# The Earliest Known Radiation of Pitheciine Primates

**DOI:** 10.1002/ajp.70040

**Published:** 2025-05-16

**Authors:** Nelson M. Novo, Gabriel M. Martin, Laureano R. González Ruiz, Marcelo F. Tejedor

**Affiliations:** ^1^ Instituto Patagónico de Geología y Paleontología (CCT CONICET—CENPAT) Puerto Madryn Argentina; ^2^ Centro de Investigación Esquel de Montaña y Estepa Patagónica (CIEMEP, CONICET—UNPSJB) Esquel Chubut Argentina; ^3^ Departamento de Ciencias de la Tierra Universidad de Zaragoza Zaragoza Spain; ^4^ Research Associate Gothenburg Global Biodiversity Centre Göteborg Sweden

**Keywords:** Miocene, parsimony, Patagonia, phylogeny, Pitheciinae, Platyrrhini

## Abstract

Two of the more interesting and controversial platyrrhine primate taxa from the Miocene of Patagonia are *Soriacebus* and *Mazzonicebus*. Although they are known basically from isolated teeth and partial mandibles and maxillae, their morphology is highly distinctive. Opinions about their phylogenetic relationships differ widely. We interpret these fossils as belonging to the lineage of the anatomically derived, living pitheciine seed‐predators; others hold the view that they are stem platyrrhines with convergent adaptations with pitheciines (with the single exception of *Proteropithecia* among the Patagonian forms), somewhat distant relatives converging coincidently with pitheciines. Here we tested these hypotheses in two ways: (1) by summarizing a character analysis of taxonomically informative traits; (2) we implemented “blind” parsimony analyses using the software package TNT, including a combined matrix of both morphological and molecular data, and replication studies of other matrices. We make some criticisms on the applied methodology of Parsimony in our analysis. *Soriacebus* and *Mazzonicebus* resulted sister‐taxa nested deeply within the pitheciid clade; thus, and according to our inferences, they are not stem platyrrhines. Most of the differences separating them from the younger and uniformly recognized pitheciine fossils *Proteropithecia*, *Nuciruptor* and *Cebupithecia* are explained as being of more primitive character states; the vast majority of resemblances and their broader functional patterns are definitively pitheciine, as typified by the living pitheciines (sakis and uakaris). We therefore found that none of the Miocene Patagonian genera treated here can be reliably interpreted as stem platyrrhines. Rather, they tend to ratify the Long Lineage Hypothesis.

## Summary

- The pitheciine primates include the living sakis and uakaris, as well as their fossil relatives since the Early Miocene.
- Morphology and molecules support the view that Patagonian pitheciines are part of the crown Platyrrini.

## Graphical abstract

Right mandible and dentition of the holotype of *Soriacebus ameghinorum* (MACN Pv SC2), from the Early Miocene of Patagonia, compared with the living *Cacajao calvus* (FMNH 88813, cast), both representing extinct and extant morphologies of the Pitheciinae: Procumbent and styliform incisors and projecting canines represent a morphological complex for sclerocarpic harvesting, a key dietary adaptation of pitheciines. As summarized in the present work, the morphology of the anterior dentition shows a remarkable adaptation shared by both species, being Soriacebus, as well as *Mazzonicebus* and *Proteropithecia*, early representatives of the subfamily.

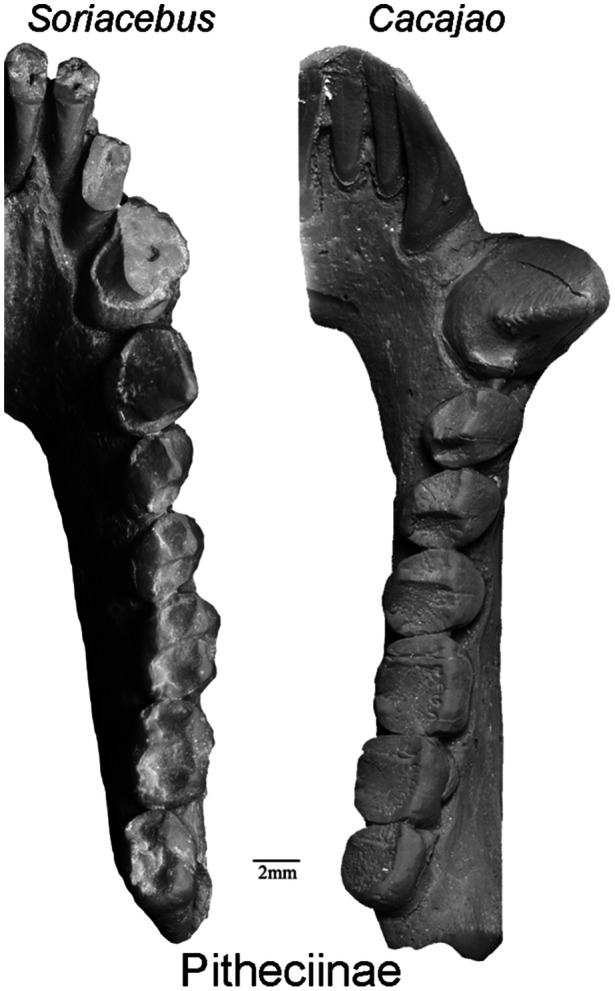

**Abbreviations**
CH: Chubut
MACN Pv: Museo Argentino de Ciencias Naturales (Buenos Aires, Argentina), Vertebrate Paleontology collection
MLP: Museo de La Plata, Buenos Aires, Argentina
MPEF‐PV: Museo Egidio Feruglio, Vertebrate Paleontology Collection. Trelew, Chubut Province, Argentina
MPM PV: Museo Regional Provincial Padre Manuel Jesús Molina, Vertebrate Paleontology Collection, Río Gallegos, Argentina
SC: Santa Cruz

## 1 Introduction

^40^Ar/^39^Ar dating at Pinturas Formation indicates an age of ~17 Ma, including the primate levels (see Fleagle et al. 2012; Perkins et al. 2012). The named primate species from Pinturas are *Soriacebus ameghinorum, Soriacebus adrianae, Carlocebus carmenensis*, and *Carlocebus intermedius* (Fleagle et al. 1987; Fleagle 1990). The most controversial primate from Pinturas has always been *Soriacebus*, represented by several dental and mandibular specimens that provided rather complete information about its unusual morphology. Nevertheless, there has been no consensus regarding the systematic position of *Soriacebus*, and different interpretations were offered since it was first described.

The original description of *Soriacebus* by Fleagle et al. (1987) presented a mixed picture of morphological resemblances. They pointed out that in anterior dentition and mandibular shape *Soriacebus* more closely resembles the living pitheciines (*Pithecia, Chiropotes, Cacajao*), while the lower premolars and molars resemble those of extant callitrichines (*Callimico*, *Leontopithecus*, *Callithrix*, *Cebuella*) for their narrowness and morphology of the molar trigonids. Additionally, Fleagle et al. (1987) drew attention to the unique condition of *Soriacebus* among platyrrhines in having three‐rooted upper premolars, while noting that the presence of a small hypocone on P4 is shared with living *Callicebus* and the extinct *Carlocebus* from Pinturas Formation. Following character analysis, Rosenberger et al. (1990) included *Soriacebus* in the Tribe Pitheciini (later elevated to subfamily rank), together with the living *Pithecia*, *Chiropotes* and *Cacajao*, as well as the extinct *Cebupithecia* from the Middle Miocene of La Venta (Colombia) (*Nuciruptor* was another Laventan pitheciine described later by Meldrum and Kay in 1997), based on several traits characterizing the dentition and mandible. Among the major shared derived features they discussed are the morphology and size of the lower incisors and the robustness of the projecting canine, which is associated with a massive and tall p2. In addition, the exaggerated posterior mandibular depth was identified as a similarity jointly shared with the living pitheciines and *Callicebus*, which is grouped within the pitheciids as well.

Kay (1990) suggested, alternatively, that *Soriacebus* is an early offshoot of platyrrhines unrelated to pitheciids, and especially emphasized the distinct morphology of its cheek teeth, which he and coworkers considered more primitive for lacking a diastema between lower incisors and canine, having reduced and compressed premolar metaconids, the m1 metaconid is placed lingually and quite distal from the protoconid, hypoconulids on m1‐2, and the presence of a discrete fovea on m1‐2. As evidence of its primitiveness in several dental characteristics, this author compared *Soriacebus* with *Apidium* and *Aegyptopithecus* from the Early Oligocene of Fayum, Egypt. This first assessment was followed by a revised, broadly based parsimony study that elaborated on this view and was based also an enlarged fossil record. There, Kay (1990) claimed *Soriacebus* was not related to pitheciids, atelids or cebids, the three major monophyletic groups of crown platyrrhines, but was instead part of a stem‐group of platyrrhines that included several other Miocene and Oligocene genera, the Stem Hypothesis (SH), that is here treated as Hypothesis 1 (Kay et al. 2008 *et seq*).

Subsequently, Kay (2010) described a new platyrrhine genus and species from the Early Miocene at the locality of Gran Barranca, in southern Chubut Province. The age of those primates was estimated by the ^40^Ar/^39^Ar method to *ca*. 20 Ma (Ré et al. 2010). The new taxon was named *Mazzonicebus almendrae*, and it was shown to have clear affinities with *Soriacebus*. Based on their similarities, Kay (2010) named the subfamily Soriacebinae, raising the rank of Tribe Soriacebini (Rosenberger et al. 1990), to include exclusively *Soriacebus* and *Mazzonicebus*, now severed from the pitheciids and regarded as a stem platyrrhines. *Mazzonicebus* is among the earliest records of Patagonian primates (see also Kramarz et al. 2012, for possibly slightly older unnamed primate specimens). Subsequently, a new maxillary specimen of *Mazzonicebus* was described later on from the Early Miocene at La Estrella (Sarmiento Formation, Chubut, Argentina) providing detailed information on the morphology of upper molars (Novo et al. 2017).

In addition, Kay and colleagues (Kay et al. 2008; Kay 2015) have concluded that all but one of the pre‐middle Miocene platyrrhines, as well as the sub‐recent Caribbean forms are also stem platyrrhines. The exception is *Proteropithecia*, from Cañadón del Tordillo, Middle Miocene of Neuquén Province (Kay et al. 1998). The alleged stem genera include, in order of age, *Branisella*, from the Late Oligocene of Bolivia; *Chilecebus* from the Early Miocene of central Chile (Flynn et al. 1995); the aforementioned *Mazzonicebus, Tremacebus* and *Dolichocebus*, also from the Early Miocene; *Carlocebus* and *Soriacebus* from younger levels of the Early Miocene; then *Homunculus* (which Kay et al. 2008; Kay 2010, 2015 synonymized with *Killikaike*), also from younger levels of the Early Miocene; as well as the Pleistocene‐Holocene Caribbean genera *Xenothrix, Antillothrix* and *Paralouatta* (a second species of *Paralouatta*, *P. marianae*, is from the Early Miocene). This analysis was strongly criticized by some authors (e.g., Rosenberger 2010; Rosenberger and Tejedor 2013, 2023; Tejedor 2013; Novo 2015; Tejedor and Novo 2017; it is noted that Beck et al. (2023), however, considered the Greater Antilles primates *Xenothrix*, *Antillothrix* and *Paralouatta* as closer to *Callicebus* instead of stem genera) for its fundamentals and conclusions, which proposed massive convergence as the explanation for the resemblances that *Soriacebus* and *Mazzonicebus* shares with modern, seed‐predaceous pitheciines. Opposed to SH, based on the morphological characteristics of the dentition and mandible, these authors proposed that *Soriacebus* and its close relative *Mazzonicebus* are part of the Family Pitheciidae, and therefore also part of the crown Platyrrhini; this is named the “Long‐Lineages Hypothesis” (LLH), here treated as Hypothesis 2 (see Rosenberger et al. 2009; Rosenberger 2010; Rosenberger and Tejedor 2013, 2023; Tejedor 2013; Tejedor and Novo 2017; Silvestro et al. 2019).

The controversies around the phylogenetic position of *Soriacebus* and *Mazzonicebus* led by the authors cited in the previous paragraph, do not affect the interpretation of *Proteropithecia*; the latter is widely accepted as a Middle Miocene pitheciine.

Marivaux et al. (2016) described *Canaanimico amazonensis* from the Late Oligocene of Chambira Formation at Contamana, Peruvian Amazonia, and considered this new taxon related to *Soriacebus* as part of a stem group cited in previous literature by Kay and colleagues listed above. A microwear analysis in two upper molars of *Canaanimico* resulted in an inferred diet with a high percentage of hard‐object feeding, as expected for the Patagonian *Soriacebus* judging for its frontal dentition, thus meaning that both genera may have shared a similar dietary pattern (Marivaux et al. 2016).

In sum, the objective of this contribution is testing the hypotheses of the fossils' phylogenetic affinities by two approaches: The first is rooted in a character analysis following the arguments used by Rosenberger et al. (1990, 2009; see also Rosenberger 1979), and in our subsequent studies of platyrrhine and pitheciid evolution (e.g., Tejedor and Rosenberger 2008; Tejedor 2013; Rosenberger and Tejedor 2013, 2023; Novo 2015), and the second employs the algorithmic, parsimony‐based method TNT (Goloboff et al. 2008), as a complementary approach to the comparative analyses.

All the specimens discussed along this text that come from the Pinturas Formation (late–Early Miocene) were collected during joint paleontological expeditions in Argentine Patagonia, by the Museo Argentino de Ciencias Naturales (Buenos Aires), the Stony Brook University (New York, USA), Laboratorio de Investigaciones en Evolución y Biodiversidad (Universidad Nacional de la Patagonia “San Juan Bosco”, Sede Esquel, Argentina), and Instituto Patagónico de Geología y Paleontología (CCT CONICET ‐ CENPAT, Puerto Madryn, Argentina).

## 2 Methods

Data for this review are based on specimens in museum collections and information published in the literature. No living animals were investigated directly for this study. The research adhered to the American Society of Primatologists Principles for the Ethical Treatment of Nonhuman Primates.

The subfamily name Homunculinae instead of Callicebinae is here used (see also Rosenberger et al. 1990, for a previously suggested tribal rank) for reasons of taxonomic priority since Ameghino (1894) was the first to use a family‐level rank placing *Homunculus* (as “Homunculidae”) in classification, and that *nomen* becomes the formal name for a suprageneric category that includes the genus *Homunculus*.

To evaluate the relationships of the Patagonian primates among the Platyrrhini, a cladistics analysis using dental, cranial, and postcranial material, as well as DNA sequences, was performed.

### 2.1 Morphological Data

Our character analysis of the extinct and extant groups follows conventional procedures to assess homology and morphocline polarity (e.g., Rosenberger 1979, 2002; Rosenberger and Strier 1989, and references therein). Parsimony analysis was performed using the software TNT 1.1 (Goloboff et al. 2008). We employed the morphological characters and character states listed by Marivaux et al. (2016) (original matrix from Kay et al. 2008; Kay 2015; Kay et al. 2019, added Marivaux et al.´s characters), for which we have checked and modified the scoring according to our criteria, we corrected some errors in the list and definitions of characters (see Supporting Information, Appendix S1). We also used the list of taxa (a total of 47; 19 living and 28 extinct) presented by Marivaux et al. (2016), adding some fossil taxa in this study: *Talahpithecus parvus*, from the Late? Eocene of Libya, North Africa (Jaeger et al. 2010), *Ucayalipithecus perdita*, from the earliest Oligocene of Perú (Seiffert et al. 2020; Campbell et al. 2021), *Parvimico materdei* from the Early Miocene of Perú (Kay et al. 2019), *Qatrania wingi* from the Early Oligocene of Egypt, North Africa (Simons and Kay 1983), *Miocallicebus villaviejai* (Takai et al. 2001) and *Laventiana annectens* (Rosenberger et al. 1991), both from Middle Miocene of La Venta, Colombia; *Insulacebus toussaintiana* from late Quaternary of Haiti (Cooke et al. 2011). Finally, and remarkably, we have added *Killikaike blakei*, from the late‐Early Miocene of Santa Cruz Formation in southeastern Patagonia (Tejedor et al. 2006), here considered as a genus distinct from *Homunculus*. We also used information provided by Ni et al. (2019) to complete missing data on the morphology of *Chilecebus*.

The purpose of the employed methodology was to attempt to replicate the same matrix used repeatedly in several phylogenetic studies of fossil platyrrhines, avoiding introducing too many substantial changes. We added taxa described after 2016, and some other taxa that were not included previously, corrected unforced errors, and recoded some character states that in our opinion were not well coded. We also use a different method to combine the morphological data with the molecular data, as explained below.

The morphological matrix included 416 characters and 55 taxa (47 in Marivaux et al. 2016). All characters were equally weighted and treated as unordered in our test to minimize the number of postulated evolutionary transformations. The results were based on a heuristic search of 1000 Wagner tree replicates followed by tree‐bisection‐reconnection (TBR).

The list of characters is provided in Supporting Information, Appendix S2, and the morphological matrix in Appendix S3.

### 2.2 Molecular Data

Eighteen living taxa have been used in the present analysis, the same included in the morphological matrix. Sixteen taxa correspond to species representing most genera from different platyrrhine subfamilies. The remaining genera are *Hylobates, Miopithecus* and *Presbytis* as outgroup.

We used 24 DNA nuclear sequences as in Perelman et al. (2011), and five DNA mitochondrial sequences (Arnason et al. 2000; Hodgson et al. 2009; Schrago et al. 2012; Finstermeier et al. 2013; Menezes et al. 2013; Janiak et al. 2022), as well as more recently obtained mitochondrial and nuclear sequences from the Jamaican subfossil *Xenothrix* (Woods et al. 2018) that were selected from GenBank (http://www.ncbi.nlm.nih.gov/genbank). All gene fragments were edited with BioEdit (Hall 1999) and aligned with Clustal W (Thompson et al. 1994; implemented in BioEdit), using default parameters. Finally, the genes were concatenated with SequenceMatrix v1.7 (Vaidya et al. 2011). Thus, a matrix of 20350 pb molecular characters only was obtained. In Supporting Information, Appendix S4 shows the access numbers to GenBank and the composition of the molecular matrix.

### 2.3 Combined Data

With combined morphological and molecular databases, we build a general matrix. The resulting matrix includes 20766 characters and 55 taxa (Supporting Information, Appendix S5). This method is different in contrast to the methodology used in the original matrix (Kay et al. 2008) and in Marivaux et al. (2016). These authors employed phylogenetic arrangements involving living taxa based on the monophyly obtained from molecular data. This means that a “forced” monophyly was used between living taxa (molecular scaffold): “*We established a ‘molecular scaffold’ upon which to superimpose the character distributions using the ‘Constraints Backbone’ option of PAUP. Under the ‘backbone’ constraint, extinct taxa are unconstrained and can move about on the molecular phylogenetic scaffold*. (Kay et al. 2008).” Our analysis favors a synergistic interaction between morphological and molecular data in a single matrix (Hermsen and Hendricks 2008; Beck et al. 2023).

## 3 Results

### 3.1 Morphological Background

To appreciate the weight of the morphological results of our character analysis, it is necessary to understand the dental anatomy of pitheciines and how it has been interpreted. Our summary then provides the context for interpreting the characters and affinities of the relevant fossils, which are addressed afterwards.

*The living pitheciines*. It is universally agreed that *Pithecia*, *Chiropotes* and *Cacajao* are the living representatives of a clade now classified within the Subfamily Pitheciinae because they share a strong suite of characters supporting their monophyly (e.g., Hershkovitz 1985, 1987a, 1987b; Rosenberger 1979; Ford 1986; Kay 1990; Kay et al. 1998; Horovitz 1999; Kay et al. 2008). This view is held by all active workers and had not been challenged for much of the 20th century. Nor have the essential characters supporting the hypothesis been debated.

**Figure 1:**
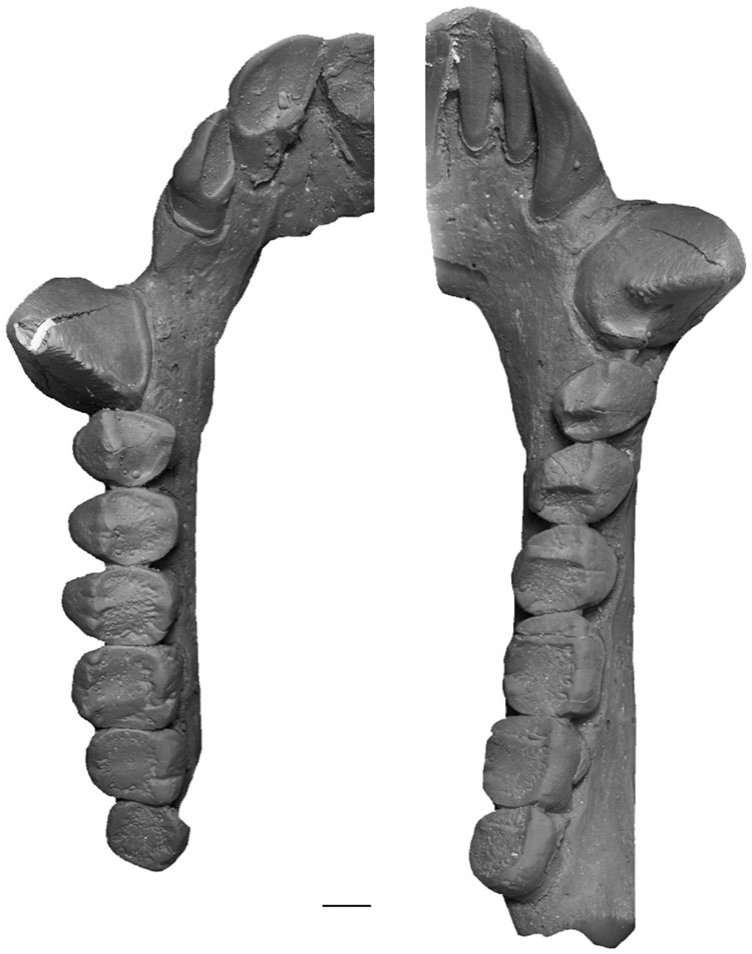
Dentition of living pitheciines. Occlusal view of the right upper (left) and right lower dentition (right) of *Cacajao calvus* (FMNH 88813, cast). Scale bar = 2 mm.

Living Pitheciinae dental anatomy is quite distinctive from the remaining platyrrhines (see Figure 1). The pattern includes tall and styliform lower incisors; the root/crown transition of i1‐2 is continuous at the cervix with no basal enlargement, and no lingual cingulum or lingual tubercle; i2 is much larger than i1. The upper incisors are extremely procumbent compared with any other platyrrhines. I1 is much larger and more spatulate than I2 and both teeth present a strong lingual cingulum. An exaggerated lingual tubercle is seen in I1. The incisor complex is also unusual in that the lower lateral incisors occlude with the upper central ones as the apical edge of the lower incisor battery tapers to form a wedge. There is a large diastema between both lower and upper lateral incisors and their adjacent canines. The extremely robust and projecting canines, most evident in *Cacajao* and *Chiropotes*, are laterally splayed. They exhibit an unusual triangular cross‐section, with a sharp entocristid defining an apex on the lingual side.

The uniqueness of this dental complex extends beyond the anterior teeth. The unicusped p2 of living pitheciines is huge and projecting, and strongly occludes between the upper canine and P2, leaving a broad wear facet on the posterolingual side of the upper canine. This arrangement is certainly an adaptation for splitting and opening hard fruits (Van Roosmalen et al. 1988; Kinzey 1992; Rosenberger 1992), like the casings of woody legumes. The p3‐4 are broader and bicuspid with separated protoconid and metaconid, and a well developed trigonid and talonid; p4 is even more molarized with a large talonid basin. In contrast to other platyrrhines, the lower molars of living pitheciines are extremely flat, quadrangular in outline with essentially undifferentiated cusps and poorly developed crests. The subtle cusps are placed marginally and they are unelevated. The trigonid is mesiodistally short and bordered by a discrete, transverse distal wall. Molar enamel is crenulate until worn and the talonid shows a distal extension posterior to entoconid and hypoconid. Upper premolars are broad and quadrate, with expanded central fovea basins in P3‐4. Upper molars present a prominent talon expansion that incorporates a large hypocone region, producing a nearly quadrate crown outline. The lingual cingulum in upper premolars and molars is basically absent. It is generally thought that the crown morphology of the cheek teeth is functionally advantageous in crushing and grinding actions, especially suitable to reducing seeds (e.g., Rosenberger and Kinzey 1976; Kinzey 1992; Rosenberger 1992; Ledogar et al. 2013).

**Figure 2:**
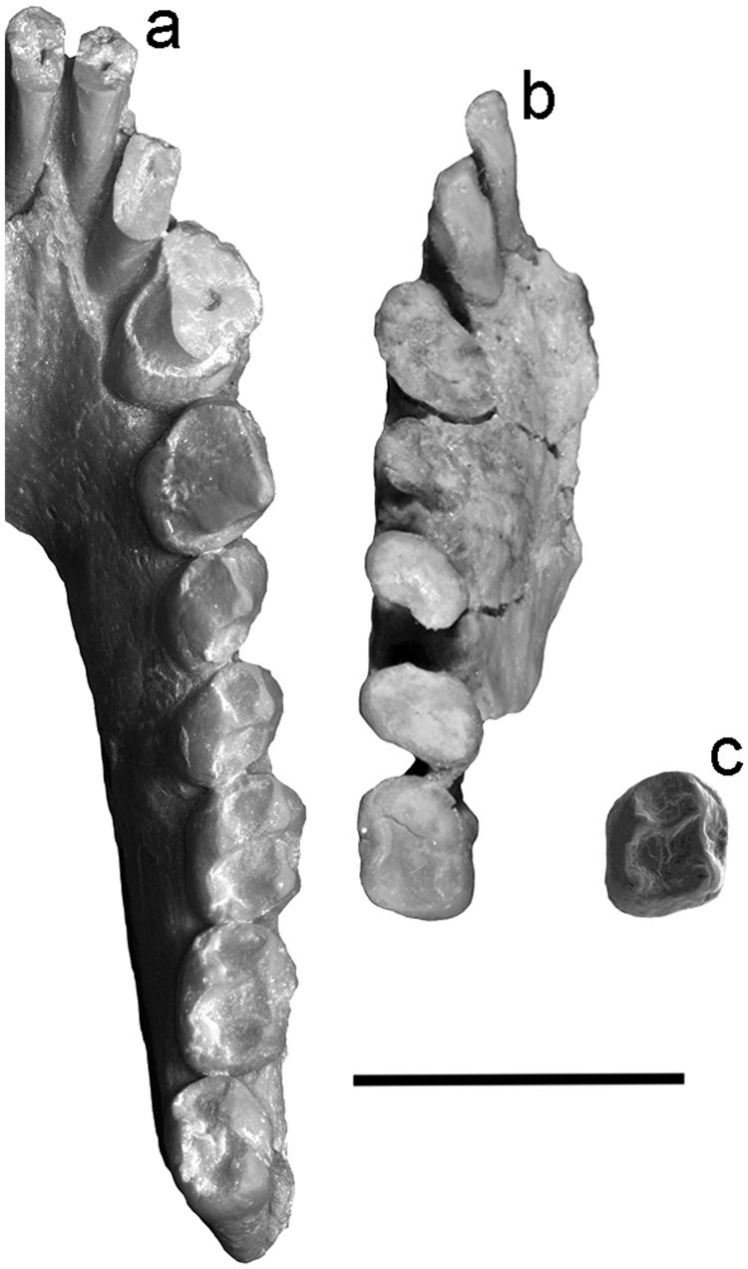
Occlusal views of (a) MACN Pv SC2 (cast), holotype of *Soriacebus ameghinorum*; (b) MPEF PV 6752, holotype of *Mazzonicebus almendrae*; and (c) MLP 91‐IX‐1‐125 (modified from Kay et al. 1998), holotype of *Proteropithecia neuquenensis*. Scale bar = 1 cm.

*The genus Soriacebus*. Fleagle et al. (1987), and Fleagle (1990) fully described dental and mandibular characters of *Soriacebus ameghinorum* and *S. adrianae*, species of almost identical morphology distinguished only by size, with the latter being smaller. Subsequent findings increased the morphological information of the genus (Tejedor 2005a, 2005b; Novo and Fleagle 2015; Novo et al. 2021). The holotype of *S. ameghinorum* (a left mandible with the whole dentition, MACN‐SC 2; Figure 2a) has a V‐shaped mandible, a massive and relatively deep symphysis, and a posteriorly deep mandibular corpus. An interesting complex of characters is seen in the anterior lower dentition, which is proportionally large in comparison with the posterior teeth. The holotype preserves the lower incisors roots and bases of the crowns which are mesiodistally compressed and procumbent. The i1 is placed in advance of i2. The base of the right lower canine is robust with a subtriangular cross‐section, and there is no diastema between the lower canine and i2. The canine cross‐section is not exactly oval because the distolingual part of the base of the crown in the holotype is considerably narrower than the buccal side. Judging from the base of the broken crown, the canine appears to be less everted than in living pitheciines.

As to the lower post‐canines, most noteworthy is the huge, unicusped p2, absolutely and relatively larger than those of the living pitheciines, especially with reference to the size of p3‐4. The lower premolars present an inflated buccal sidewall, especially at the base. The p3 is buccolingually more compressed than p4 and exhibits a small, transversely aligned metaconid and a large, taller protoconid. On p4, the protoconid and metaconid are of similar height and development and the occlusal surface is reduced, making the talonid slightly larger than the trigonid. A remarkable feature, not seen elsewhere among platyrrhines, is the mesiodistal elongation of the lower premolars and molars, an important point that has stymied some interpretations and will be discussed further below. Lower molar crowns of *Soriacebus* have well developed trigonids, slightly elevated especially on m1, with an oblique distal wall, as the metaconid is situated more distally than the protoconid. The trigonid is closed and mesially inclined. A reduced entoconid is separated from the posterior talonid crest by a small sulcus, and a very small hypoconulid is usually placed slightly lingual on the distal margin of the talonid on m1 and m2.

**Figure 3:**
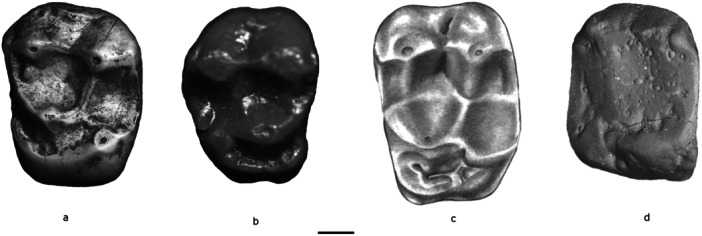
Occlusal views of the upper M1s of *Mazzonicebus almendrae* (a, MPEF‐PV 7063), *Soriacebus ameghinorum* (b, MACN Pv SC67, cast), *Cebupithecia sarmientoi* (c, UCMP 38762), and *Cacajao calvus* (d, FMNH 88813, cast). Scale bar = 1 mm.

Upper incisors are not known but the upper canine is buccolingually compressed and projects well beyond the level of the premolars. The crown has a relatively deep mesial groove. The single‐cusped upper P2 is triangular in occlusal outline. P3 and P4 are larger than P2, and both are molarized in having a strong lingual cingulum and a distinct hypocone. As mentioned above, the upper premolars differ from all extant and living platyrrhines (excepting *Paralouatta*) in having three roots. The upper molars have a strong lingual cingulum and moderately developed hypocone (Figure 3b). However, the talon basin is broad as noted by Rosenberger et al. (1990), which is related to the presence of well developed talonid cusps in the lower molars, including distinct hypoconid and entoconid.

*Mazzonicebus almendrae*. The dental morphology of *Mazzonicebus* is not well known due to the condition of the specimens. Thus, Kay (2010) in its initial description of *Mazzonicebus* focused on diagnosing the genus and establishing its affinities in connection with *Soriacebus*, a genus he had already interpreted as a stem platyrrhine. Later, Novo et al. (2017) described a new maxillary specimen from La Estrella that provided insightful information on the upper molars series. Here we present a broader perspective to assess its position relative to other platyrrhines, partly because the status of *Soriacebus* has been questioned.

Like the living pitheciines, *Mazzonicebus* also exhibits specialized incisors and relatively robust and projecting canines in a manner that can only be compared favorably with the unique complex that characterizes the modern pitheciine seed predators as described above. The holotype (MPEF PV 6752; Figure 2b) is a left mandible with part of the symphysis and left i1‐m1. The crowns of these teeth are completely worn, showing almost no details of their occlusal morphology. However, it is clear from what remains that this specimen has compact, styliform and high‐crowned incisors with mesiodistally compressed crowns and roots. The partial i1 resembles *Soriacebus* although it is somewhat smaller in caliber. The i2 has a weak lingual cingulum and well developed lingual enamel. Another specimen, MPEF PV 5351, is a symphysis showing the same characters as in the holotype. It presents partial alveoli for the incisors and a complete right canine. No diastema separates the lateral incisor root from the canine root. The canine is smaller than *Soriacebus*, relative to m1 size, and has an oval cross‐section and lingual cingulum; however, it is relatively robust. The canine entocristid is not sharp as in living pitheciines, but more discontinuous and rounded. The single‐rooted p2 resembles *Soriacebus*, being unicuspid, compressed laterally, but less projecting than in *Soriacebus*.

**Figure 4:**
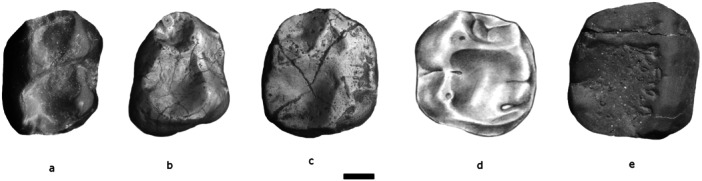
Occlusal views of the lower m1 of *Soriacebus ameghinorum* (a, in holotype, cast), *Mazzonicebus almendrae* (b, MPEF‐PV 5348), *Proteropithecia neuquenensis* (c, MLP 91‐IX‐1‐127), *Cebupithecia sarmientoi* (d, UCMP 38762), and *Cacajao calvus* (e, FMNH 88813, cast). Scale bar = 1 mm.

The lower molars of *Mazzonicebus* are represented by a highly worn m1 that is part of the holotype (MPEF‐PV 6752), and two additional isolated specimens, MPEF‐PV 6450, possibly m2, and MPEF‐PV 5348, here identified as m1 (Figure 4b). (In the Appendix 15.1 of Kay (2010), MPEF‐PV 5348 is identified as m2. Also, MPEF‐PV 6450 is the same tooth as MPEF‐PV 6484 in Kay (2010), but the number was replaced by the MPEF curator because there is another specimen with that designation in the catalog.) Both these molars exhibit a similar morphological pattern, although MPEF‐PV 5348 has smaller features. For example, the trigonid basin is very small, constricted and buccolingually narrow when compared with the broader talonid. In general appearance, however, the molars of *Mazzonicebus* are quadrangular in crown outline. The trigonid is taller than the talonid and the talonid is relatively broad, differing from *Soriacebus* where these crown components are of similar width. The *Mazzonicebus* metaconid is positioned distolingually to the protoconid as in *Soriacebus*. The distal wall of the trigonid is not interrupted by a notch. The entoconid is moderate in size and positioned distolingually to the tiny hypoconulid. MPEF‐PV 5348 and MPEF‐PV 6450 have weak and incomplete buccal cingula and exhibit a distally expanded posthypocristid and postentocristid, thus forming a distal talonid expansion as seen in the living pitheciines. Such expansion is more developed in MPEF‐PV 6450, wherein the posthypocristid and postentocristid is well extended distally. Both molars have a premetacristid projecting mesially followed by a small groove that separates a small paraconid.

**Figure 5:**
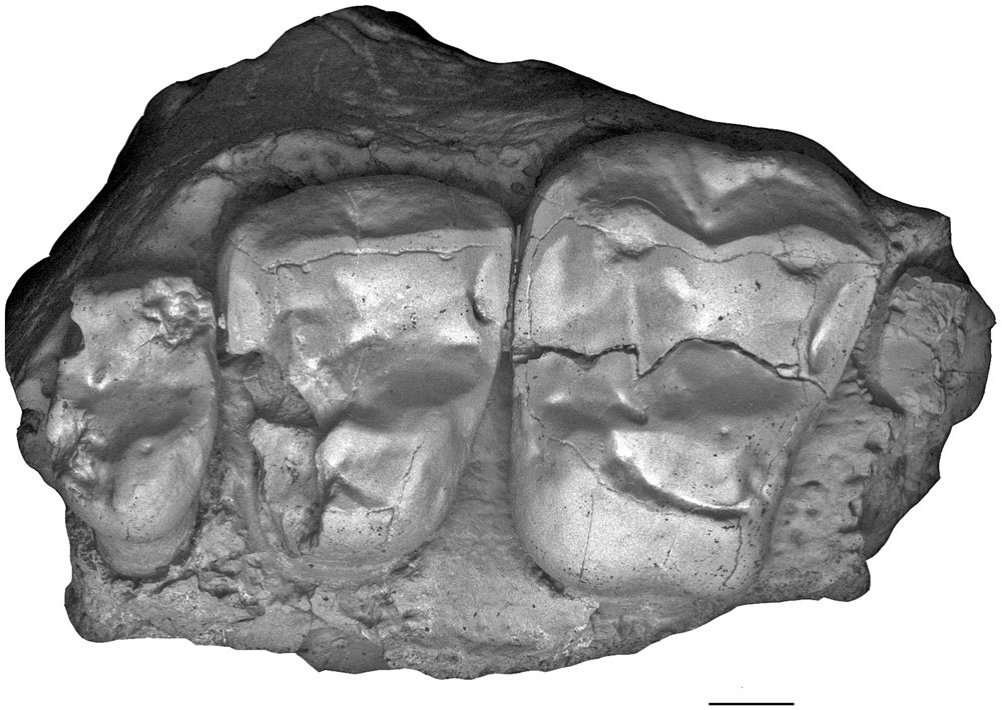
Right upper molar series of *Mazzonicebus almendrae* (MPEF‐PV 10970); modified from Novo et al. 2017). Scale bar = 1 mm.

*Mazzonicebus* is represented by few upper teeth. An isolated left upper canine, MLP 82‐V‐2‐77, strongly resembles *Soriacebus* in apparently having a projecting crown, with no basal enlargement and a deep mesial groove. The upper molars of *Mazzonicebus* show moderately developed cusps and crests (Figure 3a). M1 is larger than M2 with trapezoidal occlusal outline, being lingually narrower (see Figure 5). The four main cusps are well developed, including a moderate hypocone connected to the short, distally oriented postprotocrista by a prehypocrista. There is a moderate lingual cingulum which is not expanded as a precingulum. The mesial fovea is present. The uppers also have a distally expanded talon, as in *Soriacebus* and *Cebupithecia* (see below). The metacone of M2 is more lingually placed with respect to the paracone. The only M3 assigned to *Mazzonicebus* is present in the maxillary fragment MPEF PV 10970, from La Estrella, and is approximately one half the size of M2; it is distally damaged, broken at the metacone area. Most of the hypocone and talon are lost, as well as the tip of the paracone. However, in the middle of the distal side the enamel is complete and shows that the tooth was mesiodistally narrow, somewhat oval in shape. There is no lingual cingulum around the protocone, from which a very short and rounded prehypocrista runs distolingually. Other aspects of this M3 are difficult to describe due to its damage.

A few specimens from other localities may also pertain to *Mazzonicebus*. Hershkovitz (1981) described MLP 69‐III‐12‐I, an anterior mandibular fragment from Gran Barranca with the right p4 in situ that he attributed to *Homunculus* sp. Fleagle (1990) held the view that it could pertain to *Soriacebus* cf*. ameghinorum*, mentioning especially the broad buccal expansion, long trigonid, reduced occlusal surface of p4 and the mandible with a posteriorly deep profile. Additionally, the metaconid in p4 is close to the protoconid but distolingually placed. The trigonid is lingually open, and the mandible is buccolingually deep and thin with a robust symphysis; the dental arcade may have been rather V‐shaped. Since this specimen is from Gran Barranca, and the buccal expansion is indeed a strong character seen in *Soriacebus* as well, its attribution to *Mazzonicebus* may be correct, as Kay (2010) suggested. The caveat is that p4 of *Mazzonicebus* is otherwise unknown among the material described by Kay (2010), so direct comparisons with the type series are not possible.

In northern Chubut province, at the locality of Sacanana, MACN‐CH 354 is a left mandibular fragment with broken p4 and complete m1 that was first assigned to *Tremacebus* (Fleagle and Bown 1983), only known by an edentulous cranium. Fleagle (1990) changed his view and observed that the large trigonid and expanded buccal side of p4 is comparable to *Soriacebus*. However, Kay (2010) allocated the specimen to *Mazzonicebus*.

*Proteropithecia neuquenensis*. This is a poorly represented pitheciine from the Middle Miocene Collón Curá Formation, in the Argentine province of Neuquén (Pardiñas 1991; Kay et al. 1998; Novo et al. 2025). *Proteropithecia* exhibits a number of pitheciine‐like features that are certainly synapomorphic. Among them are the styliform and tall lower incisors with no lingual heel and the structure of the lower molars, which are quadrate with a mesiodistally short trigonid, as well as a short and straight cristid obliqua on the talonid (Figure 2c), as shown by its holotype MLP 91‐IX‐1‐125. However, in contrast to *Cebupithecia* (see below; Figure 4d) and modern pitheciines (Figure 4e), the molar cusps are more conical and better developed, the crown basins more restricted and there is an incomplete buccal cingulum. Upper and lower canines exhibit an oval cross‐sectional shape with tall, projecting crowns. The material recovered is too scarce and fragmentary to elucidate more about the dental morphology of *Proteropithecia*, but the above mentioned characteristics clearly indicate pitheciine affinities, evidently with several morphological features that are almost certainly more primitive than the states seen in living pitheciines.

**Figure 6:**
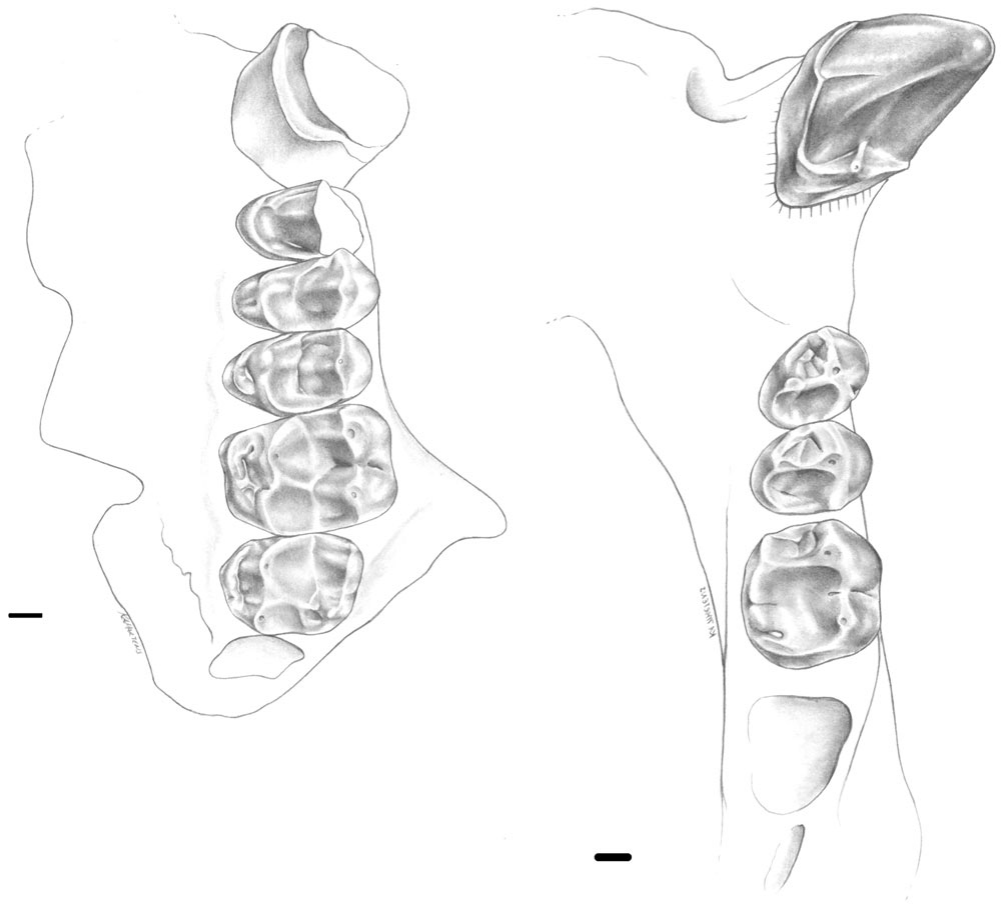
*Cebupithecia sarmientoi*, upper left (left) and lower right (right) dentitions in occlusal views. Scale bar = 1 mm. (Courtesy of Alfred L. Rosenberger).

*Other fossil pitheciines*. There are some additional Miocene relatives of *Soriacebus, Mazzonicebus, Proteropithecia*, and living pitheciines. *Cebupithecia* and *Nuciruptor* are widely accepted as extinct members of the Pitheciinae. *Cebupithecia sarmientoi* (Figure 6) comes from Middle Miocene deposits of Villavieja Fm, in La Venta, Colombia (Stirton 1951; Setoguchi et al. 1987; Meldrum et al. 1990; Meldrum and Kay 1997; Muñoz‐Saba 2018). This genus has been classically recognized as an extinct pitheciine based on characters such as the everted lower canines with triangular cross‐section and sharp entocristid, the expanded talonids of p3‐4, tall, narrow and compact incisors (indicated and inferred from preserved roots and alveoli), short trigonids and relatively low cusp relief in lower molars, upper molars with relatively low and marginal cusps and a broad talon basin. *Cebupithecia* differs from the living pitheciines especially in the absence of a diastema between i2‐c1, in having a well developed lingual cingulum in upper premolars and molars, and in the presence of smooth instead of crenulated enamel on the molars, although this may be a function of tooth wear in the type specimen.

*Nuciruptor rubricae* also comes from the Middle Miocene deposits of Villavieja Fm in La Venta (Meldrum and Kay 1997), while other attributed specimens came from La Victoria Fm (see also Muñoz‐Saba 2018); *Nuciruptor* appears to be more primitive than *Cebupithecia* in several characters. It has relatively smaller canines that are vertically projecting rather than splayed sideways and the lower canine lacks a sharp entocristid. The p2 is neither robust nor projecting and in p3‐4 the talonid is shorter than the trigonid, also in contrast to living pitheciines. However, *Nuciruptor* resembles *Pithecia, Chiropotes, Cacajao* and the extinct *Soriacebus, Mazzonicebus, Proteropithecia*, and *Cebupithecia* in the lower incisor complex, short trigonids in the lower molars, relatively low cusps and a deep mandible. There has been no disagreements about the affinities of *Nuciruptor*, which implies that the majority of its morphological difference relative to the anatomically “modern” pitheciines (i.e., sakis and uakaris) anatomy are recognized as primitive retentions of this clade.

And a more recently reported taxon related to *Soriacebus* is *Canaanimico amazonensis*, from the late Oligocene of Perú (Marivaux et al. 2016), is based on two upper molars (holotype MUSM‐2499), one of them fragmentary. The morphology resembles *Soriacebus* in general outline, the position of the hypocone and cuspules on the lingual cingulum, although *Soriacebus* has a more expanded talon. It may be an earlier representative of the radiation that led to *Soriacebus* in Patagonia, as part of the early diversification of pitheciids. However, Marivaux et al. (2016) considered *Canaanimico* as part of a stem group, an alleged monophyletic family “Homunculidae” (as Kay et al. 2008 did) joining the Patagonian primates as a whole (excepting *Proteropithecia*).

### 3.2 Parsimony

#### 3.2.1 Data From a Molecular Tree

**Figure 7:**
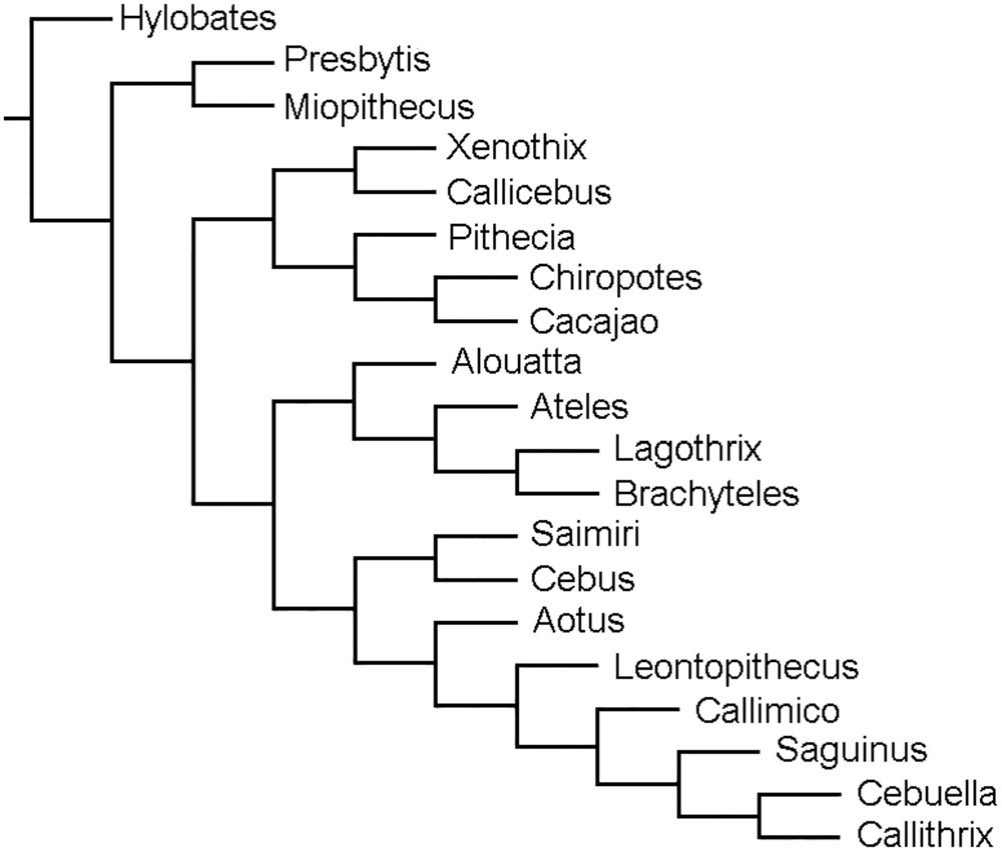
Tree resulted from the analysis of molecular data of living taxa, and the DNA obtained from *Xenothrix*.

As explained above on the analysis of molecular data from living taxa, and the DNA obtained from *Xenothrix*, our result yielded one equally parsimonious tree of 15136 steps with a CI of 0.471 and RI of 0.235 showing three distinct clades (Figure 7). More basally, the family Pitheciidae (*Pithecia, Chiropotes, Cacajao* and *Callicebus*) related to *Xenothrix*, as in Woods et al. (2018). The remaining two clades correspond to the family Atelidae (*Alouatta, Ateles, Brachyteles* and *Lagothrix*), and Cebidae (*Saimiri, Cebus, Aotus* (but see discussion on the position of *Aotus*), *Leontopithecus, Callimico, Saguinus, Cebuella* and *Callithrix*). This resulting tree is coincident with Woods et al. (2018) and Beck et al. (2023).

#### 3.2.2 Data From a Combined Tree

**Figure 8:**
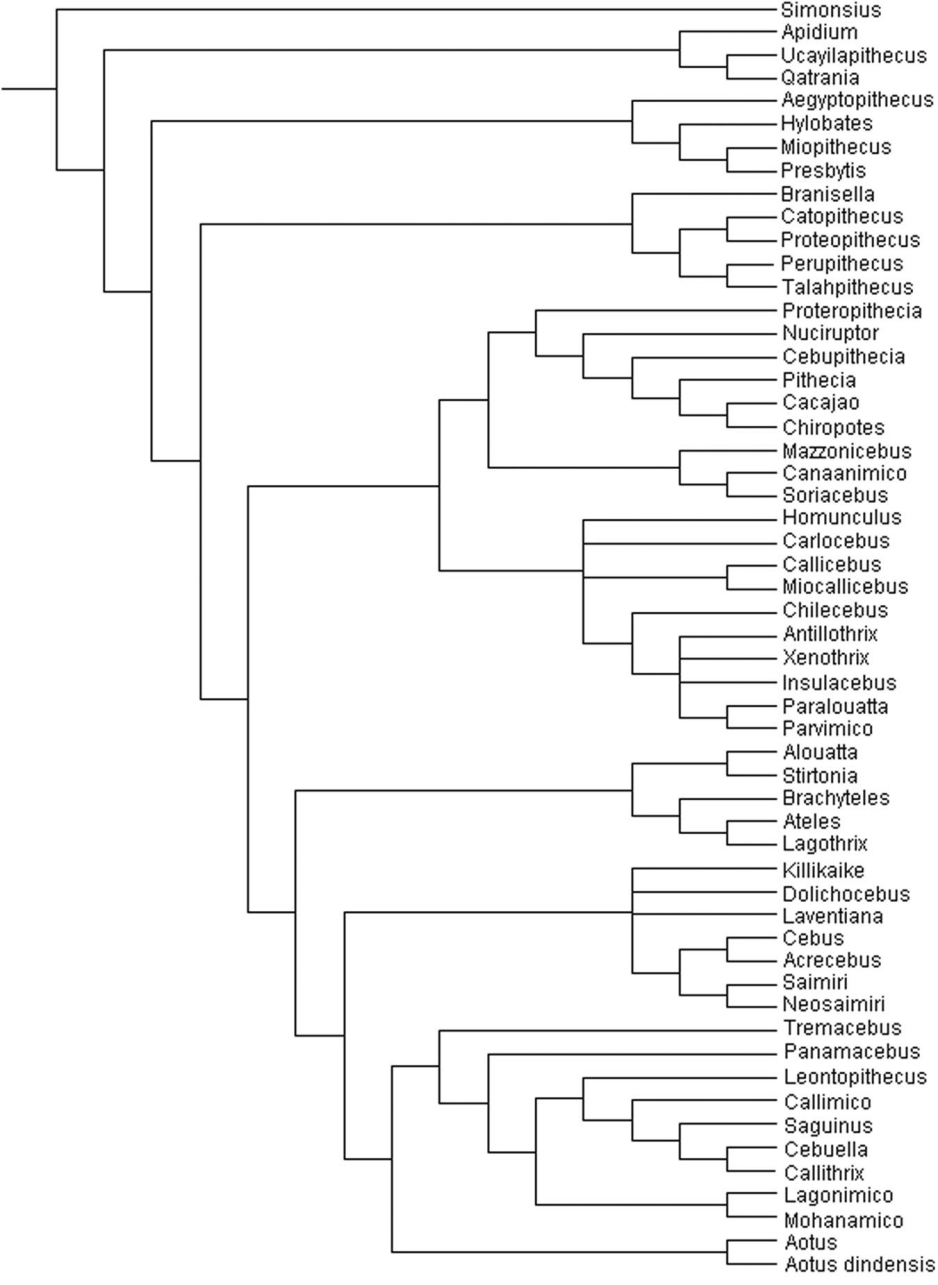
Strict consensus tree obtained from combined molecular and morphological data.

As a result of the combination of morphological and molecular data, our analysis yielded ten equally parsimonious trees of 16840 steps with a CI of 0.516 and RI of 0.431. The strict consensus tree (see Figure 8) shows the fossil platyrrhines (excepting *Perupithecus*, *Branisella*, and *Ucayalipithecus*, the latter a parapithecid) as part of the crown Platyrrhini. The position of *Branisella* is consistent with that mentioned by Takai et al. (2000), among others (Miller and Simons 1997), who recognized resemblances shared with the Fayum's *Proteopithecus*. These Salla primates have been a matter of controversy over the years due to their odd morphology, which is not easily comparable to any of the living and extinct clades, with the exception of some phenetic resemblances with callitrichines (Takai et al. 2000; Tejedor 2013).

According to the consensus tree, what is shown as crown Platyrrhini is formed by 3 or 4 clades (depending on how it is considered at the family or subfamily level). If we follow the above, we consider the first two clades as a single family Pitheciidae, composed of the subfamily Homunculinae, which includes *Homunculus, Carlocebus, Miocallicebus* and *Callicebus*, and associated with Caribbean primates (*Insulacebus, Xenotrix, Antillotrix* and *Paralouatta*), *Parvimico* and *Chilecebus*; and the other subfamily would be Pitheciinae, composed of *Mazzonicebus, Soriacebus, Canaanimico, Proteropithecia, Nuciruptor, Cebupithecia, Pithecia, Chiropotes* and *Cacajao*. The third clade is the family Atelidae formed by *Stirtonia, Alouatta, Brachyteles, Lagothrix* and *Ateles*. The fourth and last clade could be defined as the family Cebidae formed by the strongly supported Callitrichinae, the living *Cebuella, Callithrix, Saguinus*, also including the Laventan fossils *Mohanamico* and *Lagonimico*. *Panamacebus* is positioned basal to this clade, and perhaps could be considered at least as a stem Callitrichinae. Basally related to *Panamacebus* and between Callitrichinae and Cebinae is the clade formed by *Aotus*, *Aotus dindensis* and *Tremacebus*. The other cebid subfamily, Cebinae, includes the sister clade between living *Cebus* and the fossil *Acrecebus* related to the sister clade between the living *Saimiri* and the fossil *Neosaimiri*; basally related are the fossils *Laventiana, Dolichocebus* and *Killikaike*, all in a polytomy.

Therefore, the parsimony results are partially consistent with the character analysis: *Soriacebus*, *Mazzonicebus* and *Proteropithecia*, as well as *Carlocebus* and *Homunculus* are placed within the Pitheciidae, differing from Kay et al. (2008) and Kay (2015) that considered all but *Proteropithecia* as a monophyletic stem platyrrhine group. *Soriacebus* and *Canaanimico* are sister‐taxa and both are closely related to *Mazzonicebus* as part of the pitheciines, bracketed by genera most closely related to the modern sakis and uakaris, the two Colombian forms *Nuciruptor* and *Cebupithecia*, adding *Proteropithecia* from Patagonia. An ambiguous result is the position of *Aotus* and *Tremacebus* at the base of Cebidae, since morphologically they are strongly related to pitheciids, as clearly demonstrated by Rosenberger and Tejedor 2013, 2023); discussion of this inconsistency will be provided below.

## 4 Discussion

### 4.1 Patagonian and Living Pitheciines United

The empirical evidence presents an impressive suite of morphological characters shared by *Soriacebus, Mazzonicebus* and the living and fossil pitheciines when analyzed by comparative methods, and coincident with the relationships retrieved by the algorithm. Both assessments place these two Patagonian genera among pitheciines based on the amount of shared characters as an adaptive response to seed predation. These results expand our morphological and adaptive knowledge of the group by revealing mosaics that include more primitive traits in Patagonian forms, some of them shared with the Middle Miocene tropical genera *Cebupithecia* and *Nuciruptor*. This strengthens the hypothesis that the shared derived features of the pitheciine anterior tooth complex were among the first characters that evolved to produce the seed predator dietary niche (Kinzey 1992; Rosenberger 1992). A key point: Crucial features of the complex can be observed empirically in *Soriacebus* and *Mazzonicebus*. Yet it has been argued (Kay et al. 2008; Kay 2010, 2015) that *Soriacebus* and *Mazzonicebus* are not cladistically pitheciines or even pitheciids. It thus becomes important to consider exactly where, morphologically, these Patagonian forms differ from the others with respect to characters for which there is no morphological, functional‐adaptive or phylogenetic controversy.

For example, noteworthy among the unique pitheciine characters is the anterior tooth complex. The lower incisors of *Soriacebus* and *Mazzonicebus* show the same styliform, tall crowns, and the robust and projecting lower canines of *Soriacebus* tend to be somewhat triangular in cross‐section (see Tejedor 2005b), likely anticipating the sharp canine entocristid. No other platyrrhines have morphologically comparable canines, and for instance no other platyrrhines combine such canines with the same incisor anatomy as seen in pitheciines. While the lower canines are not laterally splayed in *Soriacebus* and *Mazzonicebus*, this is easily interpreted as a more primitive character state; it would not be expected that an early branching pitheciine presents precisely the same combinations of traits seen in more advanced pitheciines, as defined by their position in the cladogram, as well as other features of the dentition that are more advanced. For instance, the splayed canine pattern is also lacking in *Nuciruptor*, as mentioned above, but the genus is universally regarded as pitheciine.

As to the cheek teeth, the upper molars of *Soriacebus* and *Mazzonicebus* resemble *Cebupithecia* in the structure of the trigon and lingual cingulum, thus providing a morphocline linking the early and middle Miocene and recent genera in features whose polarities are fairly well established. The moderate hypoconne of *Soriacebus* is placed close to the protocone from the point where the lingual cingulum extends forward and forms a rounded edge mesiolingual to the protocone. This is quite evident on M1of *Soriacebus* and, to a lesser degree, in *Mazzonicebus*, making this structure virtually the same as is found in *Cebupithecia*. It only differs in that the talon of *Cebupithecia* is larger, expanded distally behind the hypocone, and the protocone and hypocone are more separated each other. This gives the molar a highly quadrate aspect, which is also quite unlike other platyrrhines. But anatomically, the crown morphology of *Soriacebus* and *Mazzonicebus* seems to be a good model of a more primitive condition. Also, although the talon of P4 is wider in *Cebupithecia* than in *Soriacebus* and *Mazzonicebus*, P2 (which is functionally incorporated with the canine into the occlusal complex) shows almost the same triangular shape in *Soriacebus* and living pitheciines.

An interesting problem known since its initial description (Fleagle et al. 1987) is that *Soriacebus* has a peculiarly shaped lower premolar and molar morphology. In crown view, these teeth are elongate with large trigonids that Fleagle et al. (1987) interpreted as a resemblance to callitrichines, in contrast to the evolutionary trend towards extreme reduction of trigonids in all other pitheciines. This pattern of elongation of p3‐4 is unique and not shared with any other platyrrhine, including *Mazzonicebus*. However, the trigonid/talonid proportions of p3‐4 are similar in *Cebupithecia* and *Soriacebus*, being shorter in *Cebupithecia*, with a long preprotocristid on p3 and a lingually inclined trigonid. This similarity is probably homologous and the premolars of *Soriacebus* may have evolved their elongate shape secondarily. *Soriacebus* has also large talonids in m1‐3, with a tiny hypoconulid frequently seen in m1‐2, even when the trigonid is large. The condition seen in callitrichines, where the trigonid is also large in lower molars, is quite different from *Soriacebus* because marmosets and tamarins have reduced talonids and talonid cusps, which is consistent with a soft diet that doesn't need a strong crushing surface in molars, like in pitheciines. Thus, there are no evident homologous similarities shared by callitrichines and *Soriacebus*. This view emphasizes the hypothesis that these traits in *Soriacebus* are most probably autopomorphic. On the other hand, the unusually elongate lower molars of the holotype of *Soriacebus ameghinorum* may be an individual variation with respect to other *Soriacebus* specimens (see Tejedor 2005a), where the trigonid:talonid breath appears subequal.

### 4.2 *Proteropithecia* and “the Others”

What does *Proteropithecia* mean in the Patagonian radiation? The SH has a consistently weak argument regarding adaptive explanations for the primate diversity in Patagonia as an evolving community: It is that all but one genus are alleged to be a stem group. The only Patagonian crown platyrrhine accepted for the SH (and for instance by most scholars; but see Beck et al. 2023) is *Proteropithecia* (Kay et al. 1998). Why *Proteropithecia* ‐a pitheciine‐ would be part of an alleged primate community of stem platyrrhines considered to be monophyletic by the SH?

Results from phylogenetic hypothesis presented by Kay et al. (2008 et seq), and Kay (2015) indicated the isolation of *Proteropithecia* as a separate evolutionary event, especially separated from *Soriacebus* and *Mazzonicebus* with similar adaptations in the anterior dentition precluding the sclerocarpic harvesting of living pitheciines discussed above. We think that additional explanations other than replicated cladistic analyses are necessary as fundamental reasons for not accepting that the adaptive thinking integrated as an ecophylogenetic approach may explain the relative coexistence of taxa with the same combination of characters, and thus expected to be part of the same clade. The most reasonable improvement for such evolutionary thinking may be that a tribe Soriacebini (i.e., *Soriacebus* + *Mazzonicebus*) and *Proteropithecia* emerged as primitive sclerocarpic harvesters, being *Proteropithecia* the oldest record of a second tribe Pitheciini, both included in the subfamily Pitheciinae. We are not saying that *Proteropithecia* is not clearly more advanced than *Soriacebus* and, to a lesser degree, *Mazzonicebus*, but they share the same adaptations for a unique feeding strategy as seed predators.

In a recently published phylogenetic hypothesis, Beck et al. (2023) ran a combined maximum parsimony molecular scaffold and undated Bayesian total evidence analyses that placed *Proteropithecia* outside the crown Platyrrhini, but grouped together with *Soriacebus*, *Mazzonicebus* and *Canaanimico;* this clade is located in a large polytomy with the rest of the clades of living and fossil platyrrhines. Beck et al. (2023) suggested that since *Proteropithecia* is not within the pitheciines, then it is not part of the crown Platyrrhini. However, we believe that their results are not conclusive having such a large polytomy in their analysis. On the other hand, these results favor the monophyletic status of the whole clade formed by *Soriacebus*, *Mazzonicebus, Canaanimico* and *Proteropithecia*, as we have mentioned throughout this study although under the LLH. Additionally, Beck et al. (2023) presented results using tip‐dating Bayesian analysis of the total evidence where *Proteropithecia* appeared as an unstable taxon, and therefore it was pruned to increase the resolution of such analysis (Beck et al. 2023). As a consequence, the position of *Proteropithecia* remained unresolved.

In a few analytical words interpreting the Patagonian primate adaptations and paleobiogeography, it must be said that, pertaining to the same evolutionary context although with different origin, does not appear the most suitable paleoecological scenario. This whole primate community living under shared large‐scale paleoenvironments, faunistic assemblage with particular adaptations, a geographical area clearly identified from other parts of what is today the extra‐Patagonian regions; that would make the whole phylogenetic scenario of stem and crown primates together hard to understand. Even when *Proteropithecia* is the youngest primate record in Patagonia (15.8 Ma, Collón Curá Formation, Middle Miocene, against 16.5 Ma for the Santa Cruz Formation primates), the dispersion to the south from northern latitudes for a whole primate community has surely had adaptive consequences for exploitation of new, appropriate environments under optimal climatic conditions and resources available. If “stem” platyrrhines radiated in southern South America, then the single crown taxon, *Proteropithecia*, incidentally arrived to the same habitat later on? Local speciations under strong natural selection pressures are powerful events that may have diversified a primate community in Patagonia at least since the Early Miocene. It seems that the adaptive and reproductive ability of that large group generated diverse morphologies and suprageneric groups that, attending to their morphological disparities, cannot be considered monophyletic as suggested by Kay et al. (2008 et seq) and Marivaux et al. (2016). From an evolutionary point of view, it would be unlikely that a single crown lineage represented by *Proteropithecia* could migrate to Patagonia after an alleged stem radiation was fully established there. Instead, we propose that a more favorable alternative is that the basal Patagonian primate community diversified there since the early Miocene giving origin to the group containing *Proteropithecia*; this is, the early Pitheciidae, and subsequently, the Pitheciinae.

Regarding the position of *Aotus* and its relatives, it should be noted that although our parsimony analysis places *Aotus*, *Aotus dindensis* and *Tremacebus* as a clade within the family Cebidae between the subfamilies Callitrichinae and Cebinae, when reviewing other hypotheses based not only on morphology but in ecology, social behavior and diet, *Aotus* is grouped with *Callicebus*, and by extension to *Tremacebus* and *Homunculus*, respectively, as the monophyletic sister group of pitheciines; this implies several possible explanations, as proposed by Rosenberger and Tejedor (2013, 2023).

### 4.3 Algorithms and Cladistic Relationships

The TNT algorithm, like others used in phylogeny reconstruction projects, is an optimization method whose goal is efficiently redistributing characters among taxa, so that the number of character state changes over the whole tree are minimized – the number of steps required to build the tree must be as few as possible. Thus the parsimony criterion takes precedence over any presumptions about individual homologies, analogies or polarities that were pre‐coded into the data.

An important value of these quantized methods is that it self‐assesses how well this objective performs, because with statistics like the Consistency and Retention Indices one gets a measure of how much homoplasy is produced as the tree is built. In other words, these statistics correspond with the phenetic load embedded in a tree. They demonstrate that such trees are not purely cladistic; rather, they fall somewhere along an axis whose opposite poles are cladistics and phenetic interpretations (see comments in Perez and Rosenberger 2014).

There are small but important differences in the characters and taxon compositions we employed to explore the phylogenetics of *Soriacebus* and *Mazzonicebus* within a larger platyrrhine context, and these produced different trees. This level of sensitivity, all by itself, presents an objective lesson that reminds us these routines are only meant to estimate affinities on the basis of the species plus character combinations fed into the matrix (Matthews and Rosenberger 2008). It means that to solve particular phylogenetic problems, one must select an appropriate framework of taxa and characters. Decisions as to what is, in fact, “appropriate,” should not be considered subjective, any more so than educated, non‐prejudicial decisions made in developing the experimental design of any scientific project.

As mentioned, the tree presented here lacks an adequate robustness. This is possibly due to the scarcity of characters supporting the nodes, or the abundance of lacking entries for the fossil taxa, or for both.

There are autapomorphies among platyrrhines at the generic level; however, it is difficult to identify characters in common, excepting the most derived groups such as living callitrichines, pitheciines, and atelids. This could be due to rapid cladogenetic events leading to the current diversity (Fleagle 2013).

The lack of sufficient characters defining the groups, a high intrageneric variation, taxa exhibiting generalized characters, and the presence of extinct taxa may produce weakly supported nodes, and even a slight modification in the data set (i.e., adding or deleting a single character) may change the topology of the tree. Relevant differences arise in several cases, and sometimes the position of a taxon changes into different families.

On the other hand, for groups represented uniquely by fossils with no support of molecular data, the phylogenetic analysis performs better. In both cases, morphological or molecular, it is necessary to consider that the results obtained only represent alternative phylogenetic hypotheses expressed in a tree. This means that a discussion focused on characters and interpretations should be also relevant in any work on phylogeny.

Additionally, it should be noted the high risk of nonvoluntary mistakes while building the matrix, as was mentioned for Kay et al. (2008) and Marivaux et al. (2016). Actually, some of those mistakes were corrected in the present work (Supporting Information, Appendix S1). Not only mistakes in coding the characters, but also the ambiguous interpretations and definitions of some of them, and those errors may be easily replicated with subsequent usage of the same matrix, as reflected in Marivaux et al. (2016) based on Kay et al. (2008). Therefore, the analysis of characters and states developed in our present work also allow to be tested in future works.

As an example for a phylogenetic analysis in platyrrhines that perpetuates character lists and coding, Kay et al. (2008) presented a matrix of 268 characters (85 cranial and 183 dental), and 31 taxa. Years later, Kay (2015) built a new matrix of 399 characters, the same uncorrected 268 characters from the 2008 matrix, adding postcranial characters, and those of the deciduous dentition, as well as using 44 taxa in total. Schrago et al. (2013) made a combined molecular and morphological phylogenetic analysis using the same morphological matrix of Kay et al. (2008). Bloch et al. (2016) tested the phylogenetic position of *Panamacebus* using the matrix of Kay (2015), with no changes in characters and coding. Marivaux et al. (2016) used the same matrix of Kay (2015) only revising and reinterpreting the character definitions and coding of the upper dentition, and adding *Canaanimico*, *Panamacebus*, and *Perupithecus* for their new phylogenetic analysis resulting in a matrix of 47 taxa. Kay et al. (2019) described *Parvimico* using the matrix published by Marivaux et al. (2016). Beck et al. (2023) employed the tip‐dating methodology combining molecular and morphological data using the same, unchanged morphological matrix of Kay et al. (2019). As expected for using that same matrix, their results show that all pre‐Laventan South and Central American primates fall out of the crown Platyrrhini.

### 4.4 Crown Vs Stem: Divergence Times

According to Kay (2010), his hypothesis on the role of the Patagonian primates in platyrrhine radiation would be also supported by the divergence times obtained by Barroso et al. (1997) based on molecular data, according to which *Callicebus* split from pitheciines between 13.5 and 16.7 Ma. Therefore, following the results of Barroso et al. (1997), *Mazzonicebus* and *Soriacebus* could not be pitheciines because *Mazzonicebus* is dated to 20 Ma, and *Soriacebus* to around 17 Ma. The same reasoning was followed by Kay (2015) for *Dolichocebus* to be excluded as a sister taxon of *Saimiri*. However, other molecular‐based studies published after Barroso et al. (1997) and before Kay (2010, 2015) have produced older divergence times for the pitheciines. For example, Opazo et al. (2006) interpreted the *Callicebus* split at about 20.35 Ma, the approximate age of *Mazzonicebus*. Similar results were obtained by Perelman et al. (2011) and Springer et al. (2012), with 20.24 and 19.84 Ma, respectively, for the last common ancestor between *Callicebus* and pitheciines. In a phylogenetic analysis using mitochondrial DNA sequences, Finstermeier et al. (2013) obtained divergence times similar to those of Opazo et al. (2006). The results showed an early divergence of the Pitheciidae with respect to the remaining platyrrhines around 22 Ma, and *Callicebus* split around 18 Ma. Perez et al. (2013) suggested an age of 27–31 Ma for the Last Common Ancestor (LCA) of crown platyrrhines using Bayesian analysis, and 21–29 Ma based on substitution rate corrected by generation time and body size. These analyses were based on both morphological and molecular evidence, thus questioning the previous estimations that gave a younger age for the divergence of the crown Platyrrhini, and for pitheciids in particular. Wilkinson et al. (2011) also made divergence time estimates for primates, and found a proportional discrepancy of 57% between their estimated mean divergence date for the crown platyrrhines following the SH of Kay et al. (2008), by eliminating the Patagonian taxa. When Wilkinson et al. (2011) included the Patagonian taxa into the crown Platyrrhini, the discrepancy was reduced to 26%. One molecular study purports to support the SH (Hodgson et al. 2009), but those results are also questionable in light of the more extensive analysis and approaches taken by Wilkinson et al. (2011) and Perez et al. (2013).

In general, studies with focus on divergence times obtain different results according to the calibration of their molecular clocks with the fossil record. Therefore, the use of the fossil record should be carefully considered and phylogenetically tested. Using fossils that are misplaced phylogenetically would result in an incorrect calibration. On the other hand, both results (phylogenetic position of the basal fossils and divergence times) should not fall into a circular reasoning. It is not correct to use divergence time data as evidence to justify the phylogenetic position of a given fossil taxon (Bibi 2013).

As mentioned above, Kay et al. (2019) used the same database reviewed here (matrix of Kay 2015, and Marivaux et al. 2016) to report a new taxon, *Parvimico materdei*. The authors concluded that the phylogenetic relationship of *Parvimico* is uncertain because it is located basally in the tree, closer to the outgroup (African taxa) than to platyrrhines. The authors inferred that perhaps a large number of missing entries in *Parvimico* led to this erroneous phylogenetic position, since the morphological characteristics of *Parvimico* are indeed comparable to platyrrhines. Based on the M1 morphology of *Parvimico* we also agree that it is more closely related to platyrrhines than to African taxa. Moreover, the conclusions of the authors are precisely what we emphasize in this study: A deep morphological study can often warn about problems arisen from the weak results of a phylogenetic analysis.

It is noted that Kay et al. (2008) offered an argument for their results by identifying the characters that define the clade of stem platyrrhines before the appearance of the Last Common Ancestor of living platyrrhines (LCA), with their own interpretation (Kay et al. 2008, 356). As argued in our present study, if we offer a deep analytical and comparative view of the characters, it will help in the phylogenetic knowledge of fossil primates in advance, than if we were only looking at the topology of a tree. For this reason, we show the changes made to the matrix, and provide a more detailed explanation of our interpretation of those characters that Kay et al. (2008) described to suggest the Patagonian primates as a stem group. These characters are identified with asterisk in the Supporting Information, Appendix S1.

In summary, our review of the well established character complex exhibited by living pitheciines, which are certainly homologously derived among platyrrhines as they occur nowhere else among primates, and are the bedrock of their unusual seed‐predator feeding strategy, overlaps with many features present in fossils – trait by trait. In cases where an individual morphological difference can be noted, there is little evidence or reason (see below) that would lead to an interpretation that these disparities are synapomorphically shared with any non‐pitheciine taxa. In fact, they almost always are logically seen as features more primitive than the core pitheciine characteristics. This is precisely what is to be expected in older, related fossils; it is the null hypothesis. In even rarer instances, as discussed further below, a small number of differences in forms like *Soriacebus* and *Mazzonicebus* may be autapomorphic (as suggested by Tejedor 2005a) but this working hypothesis needs to be further tested.

Based on a detailed morphological analyses and phylogenetic study, we conclude that pitheciines are formed by two different tribes following the classification of Rosenberger et al. (1990) for the then called subfamily Pitheciinae (currently Pitheciidae), adding the later described *Mazzonicebus* and *Proteropithecia* for the present work. Thus, *Mazzonicebus, Canaanimico* and *Soriacebus* form the tribe Soriacebini, and *Proteropithecia*, *Nuciruptor*, *Cebupithecia*, *Pithecia*, *Chiropotes* and *Cacajao* are members of the tribe Pitheciini, and both Pitheciini and Soriacebini are part of the subfamily Pitheciinae. The whole pitheciid clade including all Patagonian pitheciine taxa are part of the crown Platyrrhini, that is the LLH, as suggested previously by some authors (see Rosenberger et al. 2009; Rosenberger 2010; Tejedor 2008, 2013; Novo 2015; Bond et al. 2015; Tejedor and Novo 2017), thus validating Hypothesis 2 in this study. In sum, the present work supports the pitheciine status of *Soriacebus* and *Mazzonicebus*, as well as *Proteropithecia*, thus representing one of the broader radiations among platyrrhines.

## Author Contributions

**Nelson M. Novo:** conceptualization (equal), data curation (equal), formal analysis (equal), funding acquisition (equal), investigation (equal), methodology (equal), project administration (equal), resources (equal), software (lead), supervision (equal), validation (equal), visualization (equal), writing – original draft (equal), writing – review and editing (equal). **Gabriel M. Martin:** conceptualization (supporting), data curation (supporting), formal analysis (supporting), funding acquisition (supporting), investigation (supporting), methodology (supporting), project administration (supporting), writing – review and editing (supporting). **Laureano R. González Ruiz:** conceptualization (supporting), data curation (supporting), formal analysis (supporting), funding acquisition (supporting), investigation (supporting), methodology (supporting), project administration (supporting), writing – review and editing (supporting). **Marcelo F. Tejedor:** conceptualization (lead), data curation (equal), formal analysis (equal), funding acquisition (equal), investigation (lead), methodology (equal), resources (equal), software (supporting), supervision (lead), validation (lead), visualization (equal), writing – original draft (lead), writing – review and editing (lead).

## Ethics Statement

The authors have nothing to report.

## Supporting information

Appendix 1. Revision of characters and scoring in previous matrices by Kay et al. (2008) and Marivaux et al. (2016).

Appendix 2. List of morphological characters used in the present phylogenetic analysis.

Appendix 3. Matrix of morphological characters.

Appendix 4. DNA sequences of the molecular matrix.

Appendix 5. Matrix of the combined morphological and molecular characters.

## Data Availability

The authors have nothing to report.
