# Supplementary material for: The Earliest Known Radiation of Pitheciine Primates
Source: Am J Primatol. 2025 May 16;87(5):e70040. doi: 10.1002/ajp.70040 (PMC12082270; doi:10.1002/ajp.70040)
Supplement: Supplementary file 1 — Appendix 1. Revision of characters and scoring in previous matrices by Kay et al. (2008) and Marivaux et al. (2016). [file AJP-87-e70040-s005.docx]

**APPENDIX 1**

**Characters and score revisions**

This section shows the coding in the matrix of Marivaux et al (2016), and the changes made in the present work. Furthermore, as mentioned in the Discussion, Kay et al. (2008) suggested that the Patagonian primates would have split from the rest of the platyrrhines before the appearance of the last common ancestor (LCA). In their view, this would be supported by the retention of characters assignable to stem anthropoids found in combination with few characters shared with the crown Platyrrhini. Based on their results, in a mapping of synapomorphies they reconstructed the LCA of the crown Platyrrhini, and emphasized in 10 characters that would define the Patagonian primates as stem Platyrrhini. These 10 characters defined by Kay et al. (2008) are identified with asterisks:

**Character 6:** Zygomatic-facial foramen: 0= small relative to M1 breadth; 1= large relative to M1 breadth; 2= very large relative to M1 breadth.

*Alouatta* was scored 1, however, some variation in this character was found in the specimens revised, thus we changed to 1&2.

**Character 7:** Zygomatic arch position: 0= above the alveolar border of the maxilla; 1= below the alveolar border.

*Aotus* was scored 0, however, we found variation in the two character states, then we changed to 0&1.

**Character 9:** Zygomatic-parietal contact at pterion: 0 = no postorbital closure; 1 = zygomatic-parietal contact; 2 = alisphenoid-frontal contact.

*Tremacebus* was scored 2, however, there is a zygomatic-parietal contact in the type of *Tremacebus* (state 1). It is also confirmed by the CT-scan analysis of Fulwood et al. (2016). We changed by 1.

*Homunculus* was scored 2, however, there is variability among the different skulls assigned to *Homunculus.* We changed by 1&2.

This character is relevant because it was considered a synapomorphy for platyrrhines (Kay et al. 2008; Fulwood et al. 2016).

**Character 11:** Extraorbital exposure of the lacrimal: 0= lacrimal fossa is completed anteriorly by maxillary; 1= lacrimal has some facial exposure; 2= lacrimal contacts nasal (excludes maxillary-frontal contact).

*Homunculus* was scored 2, however, in the MPM-PV 3502, one of the best preserved skulls that provides more information, it is clearly seen how the lacrimal fossa is in contact with the maxillary, completing the fossa anteriorly. We changed the state to 0.

**Character 14:** Position of the infraorbital foramen relative to the Frankfurt horizontal plane: 0= posterior to P4; 1= positioned above P4 through P3; 2= positioned above P2.

*Nuciruptor* was scored as 1, however the only specimen assigned to *Nuciruptor* is a partial mandible (IGM 251074). We changed to ‘?.’

*Tremacebus* and *Homunculus* were scored as 1, however, a detailed study of the material indicated it is 2.

**Character 25:** Palate shape: 0= v-shaped (the distance between lingual surfaces of the upper canines divided by the between the lingual surfaces of the upper second molars is < 0.39); 1= intermediate (ratio values of ≥ 0.39, ≤ 0.64); 2= approaches parallel (ratio values > 0.64).

*Antillothrix* and *Homunculus* were scored with 2, and *Xenothrix* with 0. But following the measurements defined for the states, the three genera were changed to 1.

**Character 29:** Nature of contact between the lateral pterygoid plate and the bulla wall (cranial character 18 in Kay et al. 2004): 0= absent; 1= laminar; 2= abutting.

*Presbytis* was scored 0, but the best coding is 1. *Paralouatta* was scored as lacking entry; however, the material allows to code the character as 0.

**Character 30:** Extent of contact between the lateral pterygoid plate and the bulla wall (cranial character 19 in Kay et al. 2004): 0= slight; 1= or very extensive.

*Homunculus* was scored with 0, but it should be ‘?’ because there is no contact between the lateral pterygoid and the bulla wall, as is scored in character 29 with 0.

**Character 33:** Posterior palatine torus: 0= present; 1= absent.

*Simonsius, Aegyptopithecus*, *Catopithecus*, *Proteopithecus*, *Dolichocebus* and *Tremacebus* are scored 0, and the remaining taxa are scored 1 (excepting those scored with ‘?’).

Among these taxa, we checked that *Simonsius* is the only one showing posterior palatine torus (see Ross et al. 1998, Simons 2001)

About this character in *Dolichocebus*, Kay et al. (2008) wrote: “The remnants of the posterior parts of the palatal processes of the palatine bone are slightly thickened to form a weak posterior palatine torus. We score this feature as present in *Dolichocebus* in our character-taxon matrix while recognizing that its development is far less robust than in some non-anthropoid primates like *Adapis* (Ross 1994).’’ However, we don’t agree that the mentioned structure be a palatine torus; it is noted that this character, as erroneously scored, favored the basal position of *Dolichocebus* and *Tremacebus*, thus sharing a sinapomorphy with basal anthropoids such as *Catopithecus* and *Proteopithecus*. We changed the state to 1.

**Character 41:** Interorbital fenestra: 0= absent; 1= present.

*Dolichocebus* was scored with 0, but we changed to 1, as is also mentioned by Rosenberger (2019).

This character is important because the only taxon presenting an interorbital fenestra is *Saimiri*; therefore, the possible presence in *Dolichocebus* may imply a significant synapomorphy for both genera. As mentioned above, the presence of this trait in *Dolichocebus* is still controversial, but in the present study we suggest that the fenestra is present for the reasons mentioned by Rosenberger (2019, Fig. 1), this is that the CT scan images show the curved shape of the posterior-dorsal border of a thin bone that seems surrounding an interorbital fenestra, and it has a low probability of being a breakage. Kay et al. (2008) considered that it cannot be determined if the fenestra was present or not; however, they (and also consequently Marivaux et al. 2016) scored 0 (absent) instead of “?”, thus avoiding a possible relation with *Saimiri* in the resulting tree.

**Character 75:** Vascular canal connecting sigmoid sinus with subarquate fossa: 0= absent; 1= present.

Some taxa are scored with 2, a state not defined in character 75. In the present work, all taxa scored with 2 were replaced by 1.

**Character 78:** Symphyseal orientation: 0= more horizontal orientation of planum alveolare; 1= more vertically oriented relative to planum alveolare.

Surprisingly, *Soriacebus* was scored with “?”, although we changed to 0. *Cebus* was also scored “?”, and we changed to 1, as for *Lagonimico*. In both cases, the morphology is obvious.

**Character 80:** Mandibular corpus depth (cranial character 45 in Kay et al., 2004): 0= shallow; 1= deep.

*Saimiri* and *Leontopithecus* were scored with 1, but we changed to 0, being a stronger evidence the shallow mandible of *Saimiri*.

**Character 86:** Lower incisor number: 0= three; 1= two; 2= one: i1 present, i2 absent; 3= lower incisors absent.

*Antillothrix* and *Xenothrix* were scored 2, but they have 4 lower incisors (2 in each hemimandible), as in all platyrrhines. We changed into 1.

**Character 89:** i2-c1 diastema: 0= present; 1= absent.

All taxa were scored 1 (excepting those with lacking entry). However, it is noted that the diastema is present among living pitheciines. *Pithecia*, *Chiropotes*, *Cacajao* were changed to 0.

**Character 90:** i1-2 size (ratio of i1-2 area to m2 area): 0= very small (≤ 0.69); 1= moderate sized (≥ 0.70, ≤ 1.07); 2= large (> 1.07).

*Branisella* was scored 0 and *Soriacebus* with 2; however, there is no complete lower incisor that allows to estimate the area. We changed it into ‘?.’

**Character 91:** i1: i2 proportions (ratio of i1 area to i2 area): 0= i1 much smaller than i2 (< 0.71); 1= i1 smaller than i2 (≥ 0.71, < 0.78); 2= i1 almost as large as i2 (≥ 0.78, < 1.00); 3= i1 > i2 (≥ 1.01).

*Mazzonicebus* was scored 0, although the correct is 1.

**Character 92:** i1 crown width (spatulate incisors only): 0= considerably wider (mesiodistally) than root (spatulate); 1= narrow at apex, but still wider than root; 2= "styliform" (crown apex approximately the same width as the cervical margin).

*Aotus* was scored 0, but we changed into 1.

**Character 102:** Lower incisor lingual cingulum: 0= absent to weak; 1= strong but incomplete; 2= strong and complete.

*Panamacebus* was scored 2, but the lingual cingulum is incomplete and not strong; we changed into 1.

*Aotus* was scored 2, although the lingual cingulum is weak; changed to 0.

**Character 103:** i1 area to m1 area: 0= i1 very small (ratio ≤ 0.32); 1= moderately enlarged (> 0.32, ≤ 0.40); 2= very enlarged (> 0.40).

*Tremacebus* and *Xenothrix* were scored 0, but there are no lower incisors attributed to these genera. It was changed to ‘?.’

**Character 104:** Female c1 size (area relative to molars): 0= very small (c1 / m1 < 0.40); 1= moderate (≥ 0.4, < 0.80); 2= large (≥ 0.80, ≤ 1.20); 3= very large (> 1.20).

*Chilecebus* was scored 1, but there are no lower canines for *Chilecebus*. Therefore, we changed to ‘?.’

**Character 106:** Canine cross-sectional shape:0= rounded oval (MD:BL >1.00, <1.90); 1=MD compressed (ratio ≥ 1.90).

*Callithrix* and *Cebuella* are scored 2, but there is no state 2 for this character. This mistake was repeated in Kay et al. (2008, 2019), and Marivaux et al. (2016).

**Character 110:** P1/p1: 0= present; 1= absent.

*Antillothrix* was scored 0, but there are no platyrrhines with P1/p1. So we changed to 1.

*Chilecebus* was scored “?”, although it is clear in the holotype (SGOPV 3213) that P1 is absent. We changed to 1.

**Character 123:** p4 metaconid size: 0= absent or trace; 1= small; 2= large, almost as tall as protoconid.

*Panamacebus* was scored 1, however, the metaconid in p4 is as tall or even taller than the protoconid. We changed into 2.

**Character 136:** p2 buccal cingulum development: 0= absent; 1= incomplete, broken at protoconid and hypoconid; 2= complete.

*Tremacebus* was scored 0, but there are no lower teeth for *Tremacebus*. We changed into “?” (see also Character 137).

**Character 137:** Lower premolar inflation: 0= cusps marginal, not basally inflated; 1= crown surfaces constricted, cusp margins sloping.

*Tremacebus* was scored 1, but there are no lower teeth for *Tremacebus*. We changed into “?”.

A mandibular fragment with broken p4 and complete m1 (MACN Pv CH354) was collected at Sacanana locality in Chubut Province (Fleagle and Bown 1983), where the holotype of *Tremacebus* was found. It was always difficult to attribute MACN Pv CH354 to *Tremacebus* due to characters that according to the authors were more comparable to *Homunculus* (Fleagle and Bown 1983), *Soriacebus* (Fleagle 1990), or to a *Soriacebus*-like platyrrhine (Tejedor 2005). On the other hand, in the matrices of Kay et al. (2008), and Marivaux et al. (2016) there is no coding for any alleged lower dentition of *Tremacebus*, and only characters 136 and 137 are scored with no explanation. Therefore, we consider that MACN Pv CH354 is not attributable to *Tremacebus*; the most probable assignment is to a *Soriacebus*-like platyrrhine, as suggested by Tejedor (2005).

**Character 139:** p4 talonid length (ratio of midline mesiodistal length of trigonid to mesiodistal length of talonid): 0= extremely short or non-existing (tri:tal ≥ 1.61); 1= short (much shorter than trigonid) (tri:tal ≥ 1.27, < 1.61); 2= equal or slightly shorter in length to trigonid (tri:tal ≥ 0.92, < 1.27); 3= talonid longer than trigonid (tri:tal < 0.91).

*Callimico* was scored 3, although the talonid is never longer than the trigonid in the revised specimens; even more, it seems rather smaller. Following the measurements defined for this character we found variation, being in some cases extremely short, while short in others. We changed to 1&2.

*Saimiri* was scored 0, but following the measurments we changed to 1.

*Dolichocebus* was scored 0, but following the measurements we changed to 2.

*Panamacebus* was scored 0, but following the measurements we changed to 2.

*Neosaimiri* was scored 0, but following the measurements we changed to 2.

**Character 142:** p4 postmetaconid ridge: 0= weak or absent; 1= moderate; 2= very strong.

*Panamacebus* was scored with 0 (uniquely shared with *Apidium* and *Simonsius)*, but the best character state we interpret is 1.

**Character 147:** p4 to m1 area: 0= (< 0.62); 1= (≥ 0.63, ≤ 0.72); 2= (≥ 0.73, ≤ 0.82); 3= (≥ 0.83, ≤ 0.92); 4= (≥ 0.93, ≤ 1.02); 5= (≥ 1.03).

*Carlocebus* was scored ‘?’; however, it is possible to measure p4 and m1 of MACN Pv SC266, the holotype. Therefore, we changed to 1.

**Character 152:** m3 root number: 0= one; 1= two.

*Neosaimiri* was scored ‘?’, but some m3s exhibit part of the roots, thus being possible to code. We changed to 0.

Carlocebus was scored ‘?’, although the m3 has a single root, thus we changed to 0.

*Paralouatta* was scored with 2, but there is no state 2 for this character. However, m3 has two roots (see Horovitz and Macphee 1999), thus we changed to 1.

**Character 154:** m1 trigonid length: 0= m1 trigonid short on the lingual side; 1= m1 with elongate lingual side.

*Proteropithecia* and *Dolichocebus* were scored with ‘?.’ However, both have records of lower molars rather well preserved, so it was changed to 0.

**Character 155:** m3 trigonid width (based on relative buccolingual breadths): 0= much wider than talonid (> 1.20); 1= trigonid and talonid widths similar (1.20-1.05); 2= trigonid narrower than talonid (< 1.05).

*Aotus dindensis* was scored ‘?’, although m3s are known for the species to be scored. We change to 1.

**Character 156:** m1 paraconid position: 0= mesial to protoconid; 1= mesiolingual, between protoconid and metaconid; 2= mesial to metaconid but widely spaced from it; 3= twinned with metaconid.

*Dolichocebus* was scored 1, but we don’t think that the cusp that Kay et al. (2008) interpreted as a paraconid be indeed a paraconid. Instead, it may be an enamel crenulation. Due to the wear of MPEF-PV 5146, it can’t be confirmed, although it is highly possible that it is not a paraconid. We changed to ‘?.’

**Character 162:** m3 heel: 0= absent; 1= narrower than talonid; 2= approximately equal in width to talonid.

*Soriacebus* and *Homunculus* were scored 2; however, the m3 heel in both taxa is narrower than the talonid width. It was changed to 1.

**Character 165:** m1-2 cusp relief (ratio of hypoflexid height to hypoconid height, measured buccally): 0= low (< 1.20); 1= moderate (≥ 1.20, < 1.35); 2= high (> 1.35).

Based on the measurements of this character, we made several changes in scoring (see table s1).

**Character 168:** m1-2 paraconid development: 0= absent; 1= small; 2= large.

*Dolichocebus* was scored 1, but there is no paraconid in m1. We changed to 0.

**Character 175:** m1-2 postentoconid sulcus: 0= prominent; 1= shallow sulcus; 2= absent.

*Dolichocebus* was scored 0. However, we interpret the sulcus as shallow, and especially in m2, where it is slightly defined. We changed to 1.

**Characters 176 and 177:** m1 hypoconulid size: 0= large; 1= moderate; 2= small; 3= absent / m2 hypoconulid size: 0= large; 1= moderate; 2= small; 3= absent.

*Saguinus* was scored 3 in both characters, but some specimens exhibit a small hypoconulid in m1 or m2. It was changed to 2&3.

**Character 179:** m1-2 hypoconulid position: 0= twinned to entoconid; 1= near midline; 2= slightly buccal to midline.

*Dolichocebus* was scored 1; however, we consider that if a hypoconulid indeed exists, it is positioned twinned to the entoconid, as indicated in Fig. 17 of Kay et al. (2008). We changed to 0.

**Character 188:** m3 hypocristid development: 0= absent or seen only as a trace; 1= weak; 2= strong.

*Proteropithecia* was scored 1, but there is no m3 assigned to *Proteropithecia*. We changed to ‘?.’

**Character 190:** m1-2 distal fovea: 0= absent; 1= present.

*Aotus* and *Neosaimiri* were scored 0, but this character is variable, having distal fovea several specimens of *Aotus* and *Neosaimiri*. We changed to 0&1.

**Character 193:** m1 hypoflexid depth: 0= very shallow; 1= moderate; 2= deep.

*Proteropithecia* was scored 1, however the hypoflexid is very shallow like *Soriacebus* and *Mazzonicebus,* even similar to living piteccines. We changed to 0.

**Character 194:** m2 hypoflexid depth: 0= very shallow; 1= moderate; 2= deep.

*Proteropithecia* was scored 1; however the hypoflexid is very shallow like in *Soriacebus* and *Mazzonicebus*, and similar to living pitheciines. We changed to 0.

**Character 195:** Ratio of m2 length to m3 length: 0= m3 much longer than m2 (0.71-0.80); 1= m3 longer than m2 (0.81-0.90); 2= m3 equal to m2 (0.91-1.00); 3=m3 smaller than m2 (1.01-1.12); 4= m3 much smaller than m2 (≥ 1.13). Scored 5 if m3 is absent.

Based on the measurements, *Homunculus* and *Cebus* were changed (see table s1). *Callithrix* and *Callimico* were also changed, because m3 was scored in *Callithrix* and it has no m3; on the other hand, m3 was scored as absent in *Callimico*, and *Callimico* indeed has an m3.

**Character 201:** I2–C1 diastema: 0= present; 1= absent.

*Chilecebus* was scored 1, but it has diastema between I2-C. It was here changed to 0.

*Paralouatta* was scored 1, but based on the recorded specimens we cannot be sure. We changed to ‘?.’

**Character 212:** C1 cross-sectional shape (ratio of maximum length in the occlusal plane to maximum breadth in the occlusal plane at right angles to maximum length): 0= oval (≥ 1.16); 1= rounded (< 1.16).

*Aotus dindensis* was scored absent for this character; however, they did not consider the upper canine IGM-KU 98008 attributed to *A. dindensis* by Takai et al. (2009). We scored 1

**Character 213:** Upper canine occlusion: 0= C1 wears against P1-2; 1= C1 wears against P2; 2= C1 wears against P2-3; 3= C1 wears against P3.

*Aotus dindensis* was scored ‘?’, but they did not consider the upper canine IGM-KU 98008 attributed to *A. dindensis* by Takai et al. (2009). We scored 1.

**Character 214:** C1 mesial groove (females): 0= shallow or absent; 1= deep.

*Aotus dindensis* was scored ?; however, they did not consider the upper canine IGM-KU 98008 attributed to *A. dindensis* by Takai et al. (2009). We scored 1.

**Character 215:** C1 lingual cingulum: 0= weak or absent; 1= strong; 2= very strong.

*Aotus dindensis* was scored “?”, and again they did not consider the upper canine IGM-KU 98008 attributed to *A. dindensis* by Takai et al. (2009). We scored 1

*Callimico* was scored ‘?.’ We changed to 0.

**Character 216:** P2 root number: 0= one; 1= two; 2= three. If tooth is absent, character scored as ‘?.’

Several changes were introduced in the matrix for this character, mostly living taxa that were coding with lacking entries when in fact they could be scored easily (see table s1).

*Aotus dindensis* was scored ‘?’; however, they did not consider the upper premolars IGM-KU 98009 and 98010 attributed to *A. dindensis* by Takai et al. (2009). We scored 1.

**Character 219:** Ratio of P2 area to P3 area: 0= P2 very small (≤ 0.85); 1= P2 small (> 0.85, < 0.95); 2= P2 equal (≥ 0.95). If tooth is absent, character scored ‘?.’

*Aotus dindensis* was scored ‘?’. Again, P2-3 IGM-KU 98009 and 98010 are attributed to *A. dindensis* by Takai et al. (2009). We scored 1.

*Xenothrix* was scored 0&1, but there is no P2 assigned to *Xenothrix*. We changed to ‘?.’

**Character 221:** P2 occlusal shape (mesiodistal length/buccolingual breadth): 0= buccolingually broad (≤ 0.80); 1= round (> 0.80, ≤ 1.05); 2= mesiodistally elongate (> 1.05).

This character was re-defined to better show its variability, and also to eliminate measurements that may be arbitrary in several cases.

It was defined as follows**:** Occlusal outline of P2: 0= triangular; 1= suboval, with the larger axis buccolingually; 2= suboval, with the larger axis mesiodistally; 3= rounded.

**Character 222:** P3-4 trigon/talon proportions: 0= trigon and talon proportions similar; 1= trigon much shorter than talon with the protocone situated on the mesial aspect of the crown.

*Branisella* was scored 1, but the trigon is clearly shorter than the talon. We changed to 1.

**Character 227:** Premolar hypocones: 0= absent; 1= present on P4 only; 2= present on P3-4; 3= present on P2-4.

*Callicebus* was scored 1, but we observed variation in this character, such as presence or absence of the hypocone in P4. We changed to 0&1.

*Carlocebus* was scored with 1&2&3, but there is no hypocone on P2. We changed to 1&2.

**Character 235:** This character is no listed neither in Kay (2015) nor Marivaux et al. (2016), but judging for the number of characters in their respective matrices, we think that character 235 was scored there. It is possibly the character 235 of Kay et al. (2008) (P4 metacone: 0= absent; 1= present) because they show the same coding, and in general the three matrices are rather similar. We also note that character 235 replicate 224 in Kay et al. (2008).

**Character 238:** M3 root count: 0= three; 1= two; 2= one.

*Xenothrix* was scored 0, but it has no M3. We changed to ‘?.’

*Mazzonicebus* was scored with lacking entry because there was no M3 found at that time. However, Novo et al. (2017) reported a new specimen with M3, and it has one root. We changed to 2.

**Character 239:** M2 shape (ratio of buccolingual breadth / mesiodistal length): 0= very transverse (> 1.65); 1= transverse (≤ 1.65, > 1.30); 2= squared (≤ 1.30).

This character was modified following (Chaimanee et al. 2012, character 152); Cross-sectional outline of M1 shape: 0= triangular; 1= subtriangular, transverse; 2= rectangular, transverse; 3 = quadrangular), because it better represents the morphology of the upper molars.

The modification of this character, adding a state and referring to M1 and not to M2, better characterizes the morphology of the upper molars.

**Character 243:** M1-2 metaconule: 0= single (or absent); 1= double.

The character is not useful in the sample for the present work; all are scored 0. Therefore, it was deleted and replaced by character 401 from Marivaux et al. (2016): M1-2 metaconule: 0= absent to indistinct; 1= small; 2= moderate; 3= large.

*Pithecia* and *Callicebus* were scored with 1, although in a careful revision of several specimens of both genera we found that the metaconule was not present in upper molars. We changed to 0&1.

**Character 246:** M1 hypocone size: 0= large; 1= small; 2= absent or crestiform.

*Perupithecus* was scored 1, but we consider that the hypocone is crestiform without affecting the triagular shape of the M1 in *Perupithecus*, and the original character states do not reflect the real variability in hypocone development. Our suggested change is as follows:

M1 hypocone size: 0= large; 1= small; 2= absent or crestiform.

Then, *Perupithecus* was here scored 2.

*Panamacebus* was scored 1; however, we consider that *Panamacebus* has a well developed hypocone, as described in Bloch et al. (2016). We changed to 0.

**Character 247:** M2 hypocone size: 0= large; 1= small; 2= absent.

*Tremacebus* was scored 1, however, the holotype preserves the broken and worn left upper molars that show a rather large hypocone. It has to be scored as 0.

*Panamacebus* was scored 1&2, but there is just one M2 of *Panamacebus*, and the hypocone is large. We changed to 0.

**Character 248:** (Marivaux et al 2016; Character 400). M1-2 hypocone position: 0= distal, far lingual to protocone; 1= distal, slightly lingual to protocone; 2= same level (mesiodistally opposed); 3= distal, slightly buccal to protocone.

*Callicebus* and *Aotus* were scored 2; however, we interpret that the hypocone is slightly lingual to the protocone and not at the same level. We change to 1.

**Character 249:** M1-2 prehypocrista: 0= absent; 1= weak; 2= strong, reaches the postprotocrista, encloses the talon lingually.

*Mazzonicebus* was scored 0&1, however, upper molars assigned to *Mazzonicebus* have a weak prehypocrista. We changed to 1.

*Tremacebus* was scored 1, but due to the high wear of the upper molars in the holotype, it is not possible to assess the state of this character. We changed to ‘?.’

*Carlocebus* was scored 1; however, we found a high variability among all upper molars assigned to *Carlocebus*, being present the three states defined in this character. We changed to 0&1&2.

**Character 251:** M3 prehypocrista development: 0= absent; 1= weak; 2= strong, reaches to postprotocrista to enclose the talon lingually.

*Mazzonicebus* was scored ‘?’, but we changed to 0 because a new specimen having M3 was found (Novo et al. 2017).

**Character 253:** This character was eliminated from the matrix by Marivaux et al. (2016), and replaced for character 413 of the same matrix with no coding changes: Hypometaconule crista (= metacrista or crista obliqua): 0= indistinct to absent; 1= moderate (not connected to protocone); 2= well developed (connected to protocone or postprotocrista).

**Character 256:** This character was eliminated from the matrix by Marivaux et al. (2016), and replaced for character 412 of the same matrix: M1-2 hypoparacrista: 0= absent; 1= weakly developed (short); 2= well developed (high).

*Callimico* was scored with lacking entry, and was changed to 2.

**Character 257:** This character was eliminated from the matrix by Marivaux et al. (2016), and replaced for character 411 of the same matrix: M1-2 hypometacrista: 0= absent; 1= weakly developed (low and short); 2= well developed (high).

Several changes were introduced according to our concept on the hypometacrista development on M1-2.

**Character 259:** This character was eliminated from the matrix by Marivaux et al. (2016) and replaced by character 414 of the same matrix. M1-2 lingual cingulum development: 0= absent; 1= faintly visible; 2= well defined; 3= strong.

Several changes were made according to our interpretation (see table s1).

**Character 263:** M3 paraconule: 0= absent; 1= small-moderate; 2= large.

*Acrecebus* was scored 0, but there is no record of M3 for *Acrecebus*. We changed to ‘?.’

*Dolichocebus* and *Homunculus* were scored as 1, but we do not consider that the upper molars of both taxa have a paraconule. We changed to 0.

**Character 266:** M3 metacone: 0= absent or very small; 1= moderate (but

smaller than paracone); 2= large (equal to paracone).

*Mazzonicebus* was scored with ‘?’, but the new specimen (Novo et al., 2017) exhibits this character. We changed to 1.

**Character 267:** M3 hypocone: 0= absent or very small; 1= small; 2= large.

*Tremacebus* was scored 0&1, but the only M3 recorded for the genus is that of the holotype skull, broken and worn; it does not correspond a double coding. We changed to 0.

**Character 268:** This character was eliminated from the matrix by Marivaux et al. (2016) and replaced by character 423 of the same matrix) M1-3 anterior cingulum: 0= strong; 1= weak; 2= absent.

*Soriacebus* was scored 2, but the character is highly variable in the genus. We changed to 0&1&2.

*Canaanimico* was scored 2 but it has a weak anterior cingulum. We changed to 1.

*Tremacebus* was scored 1, however there is no anterior cingulum in the upper molar of *Tremacebus*. We changed to 2.

**Character 269:** M1 size relative to M3 (based on the ratio of areas of each tooth): 0= M1 ≥ 2.5 times the size of M3 (scored as “0” when M3 is absent); 1= M1 < 2.5, ≥ 1.5 times M3; 2= M1 < 1.5 times M3.

*Mazzonicebus* was scored with lacking entry, but now there is a new specimes with M3 (Novo et al. 2017). We changed to 0.

**Character 413:** This is the same character 420 in Marivaux et al. (2016). M1-3 posterior margin (waisting between buccal and lingual cusps): 0= indistinct to absent; 1= present but shallow; 2= present, deep.

*Branisella* was scored 0, but we interpret it is 1.

**Table s1 of changes in the Matrix**

| **Character** | **Marivaux et al. (2016)** | **This study** |
| --- | --- | --- |
|  | **Changes in character coding** | |
| 6 | *Alouatta*= 1 | *Alouatta* = 1&2 |
| 7 | *Aotus*= 0 | *Aotus* = 0&1 |
| 9 | *Tremacebus*= 2 | *Tremacebus* = 1 |
|  | *Homunculus*= 2 | *Homunculus* = 1&2 |
| 11 | *Homunculus* = 2 | *Homunculus* = 0 |
| 14 | *Tremacebus* = 1 | *Tremacebus* = 2 |
|  | *Homunculus* = 1 | *Homunculus* = 2 |
|  | *Nuciruptor* = 1 | *Nuciruptor* = ? |
| 25 | *Antillothrix* = 2 | *Antillothrix* = 1 |
|  | *Xenothrix* = 0 | *Xenothrix* = 1 |
|  | *Homunculus* = 2 | *Homunculus* = 1 |
| 29 | *Presbytis* = 0 | *Presbytis* = 1 |
|  | *Paralouatta* = ? | *Paralouatta* = 0 |
| 30 | *Homunculus* = 0 | *Homunculus* = ? |
| 33 | *Catopithecus* = 0 | *Catopithecus* = 1 |
|  | *Proteopithecus* = 0 | *Proteopithecus* = 1 |
|  | *Dolichocebus* = 0 | *Dolichocebus* = 1 |
|  | *Tremacebus* = 0 | *Tremacebus* = 1 |
| 41 | *Dolichocebus* = 0 | *Dolichocebus* = 1 |
| 75 | Errors in the state definition | Several taxa |
| 78 | *Branisella* = ? | *Branisella* = 0 |
|  | *Soriacebus =* ? | *Soriacebus* = 0 |
|  | *Cebus =*? | *Cebus* = 1 |
|  | *Lagonimico =*? | *Lagonimico* = 1 |
| 80 | *Saimiri* = 1 | *Saimiri* = 0 |
|  | *Leontopithecus* = 0 | *Leontopithecus* = 0 |
| 86 | *Antillothrix* = 2 | *Antillothrix* = 1 |
|  | *Xenothrix* = 2 | *Xenothrix* = 1 |
| 89 | *Pithecia* = 1 | *Pithecia* = 0 |
|  | *Cacajao* = 1 | *Cacajao* = 0 |
|  | *Chiropotes* = 1 | *Chiropotes* = 0 |
| 90 | *Branisella* = 0 | *Branisella* = ? |
| 91 | *Mazzonicebus* = 0 | *Mazzonicebus* = 1 |
| 102 | *Panamacebus =* 2 | *Panamacebus* = 1 |
|  | *Aotus =*2 | *Aotus* = 0 |
| 103 | *Tremacebus =*0 | *Tremacebus* = ? |
|  | *Xenothrix =*0 | *Xenothrix* = ? |
|  | *Cebupithecia =*0 | *Cebupithecia* = ? |
| 104 | *Chilecebus =*1 | *Chilecebus* = ? |
| 110 | *Antillothrix*= 0 | *Antillothrix* = 1 |
|  | *Chilecebus =*? | *Chilecebus* = 1 |
| 123 | *Panamacebus* = 1 | *Panamacebus* = 2 |
| 126 | *Dolichocebus =* 2 | *Dolichocebus* = 1&2 |
| 136 | *Tremacebus* = 0 | *Tremacebus* = ? |
| 137 | *Tremacebus* = 1 | *Tremacebus* = ? |
| 139 | *Callimico* = 3 | *Callimico* = 1&2 |
|  | *Saimiri* = 0 | *Saimiri* = 1 |
|  | *Dolichocebus* = 0 | *Dolichocebus* = 2 |
|  | *Panamacebus* = 0 | *Panamacebus* = 2 |
|  | *Neosaimiri* = 0 | *Neosaimiri* = 2 |
| 142 | *Panamacebus* = 0 | *Panamacebus* = 1 |
| 147 | *Carlocebus* = ? | *Carlocebus* = 1 |
| 152 | *Neosaimiri* = ? | *Neosaimiri* = 0 |
|  | *Carlocebus* = ? | *Carlocebus* = 0 |
|  | *Paralouatta* = 2 | *Paralouatta* = 1 |
| 154 | *Proteropithecia* = ? | *Proteropithecia* = 0 |
|  | *Dolichocebus* = ? | *Dolichocebus* = 0 |
| 155 | *Aotus dindensis* = ? | *Aotus dindensis* = 1 |
| 156 | *Dolichocebus* = 1 | *Dolichocebus* = ? |
| 162 | *Soriacebus* = 2 | *Soriacebus* = 1 |
|  | *Homunculus* = 2 | *Homunculus* = 1 |
| 165 | *Cebuella* = 0 | *Cebuella* = 1 |
|  | *Proteropithecia* = ? | *Proteropithecia* = 0 |
|  | *Antillothrix* = ? | *Antillothrix* = 1 |
|  | *Carlocebus* = ? | *Carlocebus* = 1 |
|  | *Homunculus* = 2 | *Homunculus* = 1 |
| 168 | *Dolichocebus* = 1 | *Dolichocebus* = 0 |
| 175 | *Dolichocebus* = 0 | *Dolichocebus* = 1 |
| 176 - 177 | *Saguinus* = 3 | *Saguinus* = 2&3 |
| 179 | *Dolichocebus* = 1 | *Dolichocebus* = 0 |
| 188 | *Proteropithecia* = 1 | *Proteropithecia* = ? |
| 190 | *Aotus* = 0 | *Aotus* = 0&1 |
| 193 | *Proteropithecia =* 1 | *Proteropithecia* = 0 |
| 194 | *Proteropithecia =* 1 | *Proteropithecia =* 0 |
| 195 | *Callithrix* = 4 | *Callithrix* = 5 |
|  | *Callimico* = 5 | *Callimico* = 4 |
|  | *Homunculus* = 2 | *Homunculus* = 3 |
|  | *Cebus* = 3 | *Cebus* = 4 |
| 201 | *Chilecebus* = 1 | *Chilecebus* = 0 |
|  | *Paralouatta* = 1 | *Paralouatta* = ? |
| 212 | *Aotus dindensis* = ? | *Aotus dindensis* = 1 |
| 213 | *Aotus dindensis* = ? | *Aotus dindensis* = 1 |
| 214 | *Aotus dindensis* = ? | *Aotus dindensis* = 1 |
| 215 | *Aotus dindensis* = ? | *Aotus dindensis* = 1 |
|  | *Callimico* = ? | *Callimico* = 0 |
| 216 | *Cacajao* = ? | *Cacajao* = 0 |
|  | *Chiropotes* = ? | *Chiropotes* = 0 |
|  | *Cebus* = ? | *Cebus =* 1 |
|  | *Lagothrix* = ? | *Lagothrix* = 0 |
|  | *Proteopithecus* = ? | *Proteopithecus* = 0 |
|  | *Aotus dindensis* = ? | *Aotus dindensis* = 0 |
| 217 | *Aotus dindensis* = ? | *Aotus dindensis* = 1 |
|  | *Tremacebus* = 0 | *Tremacebus* = 1 |
| 219 | *Aotus dindensis* = ? | *Aotus dindensis* = 1 |
|  | *Xenothrix* = 0&1 | *Xenothrix* = ? |
| 221 | *Simonsius* = 0 | *Simonsius* = 1 |
|  | *Apidium* = 0 | *Apidium* = 1 |
|  | *Cebuella* = 1 | *Cebuella* = 0 |
|  | *Callithrix* = 1 | *Callitrhix* = 0 |
|  | *Leontopithecus* = 1 | *Leontopithecus* = 0 |
|  | *Lagonimico* = 1 | *Lagonimico* = 0 |
|  | *Pithecia* = 1 | *Pithecia* = 2 |
|  | *Cacajao* = 0 | *Cacajao* = 2 |
|  | *Chiropotes* = 0 | *Chiropotes* = 2 |
|  | *Alouatta* = 1 | *Alouatta = 3* |
|  | *Brachyteles* = 1 | *Brachyteles = 3* |
|  | *Ateles* = 0 | *Ateles* = 3 |
|  | *Lagothrix* = 0 | *Lagothrix* = 2 |
|  | *Cebupithecia* = 0 | *Cebupithecia* = 1 |
|  | *Mazzonicebus* = 0&1 | *Mazzonicebus* = 1 |
|  | *Callicebus* = 0 | *Callicebus* = 1 |
|  | *Aotus* = 0 | *Aotus* = 2 |
|  | *Homunculus* = 1 | *Homunculus* = 0 |
|  | *Branisella* = 1 | *Branisella* = 0 |
| 222 | *Branisella* = 0 | *Branisella* = 1 |
| 227 | *Callicebus* = 1 | *Callicebus* = 0&1 |
|  | *Carlocebus* = 1&2&3 | *Carlocebus* = 1&2 |
| 235 | Not in the list. | Ch. 235 (Kay et al 2008) |
| 238 | *Xenothrix* = 0 | *Xenothrix* = ? |
|  | *Mazzonicebus* = ? | *Mazzonicebus* = 1 |
| 239 | *Simonsius* = 2 | *Simonsius* = 3 |
|  | *Apidium* = 1 | *Apidium* = 2 |
|  | *Cebuella* = 1 | *Cebuella* = 0 |
|  | *Callithrix* = 1 | *Callithrix* = 0 |
|  | *Leontopithecus* = 1&2 | *Leontopithecus* = 0&1 |
|  | *Pithecia* = 2 | *Pithecia* = 3 |
|  | *Cacajao* = 2 | *Cacajao* = 3 |
|  | *Chiropotes* = 2 | *Chiropotes* = 3 |
|  | *Alouatta* = 2 | *Alouatta* = 3 |
|  | *Stirtonia* = 2 | *Stirtonia* = 3 |
|  | *Brachyteles* = 2 | *Brachyteles* = 3 |
|  | *Ateles* = 2 | *Ateles* = 3 |
|  | *Lagothrix* = 2 | *Lagothrix* = 3 |
|  | *Catopithecus* = 1 | *Catopithecus* = 2 |
|  | *Proteopithecus* = 0 | *Proteopithecus* = 1 |
|  | *Homunculus* = 1 | *Homunculus* = 2 |
|  | *Carlocebus* = 1 | *Carlocebus* = 2 |
|  | *Dolichocebus* = 1 | *Dolichocebus* = 2 |
|  | *Tremacebus* = 1 | *Tremacebus* = 2 |
|  | *Acrecebus* = 2 | *Acrecebus* = ? |
| 240 | *Dolichocebus* = 2 | *Dolichocebus* = 1 |
|  | *Perupithecus* = - | *Perupithecus* = ? |
| 243 (401) | *Callicebus* = 1 | *Callicebus =* 0&1 |
|  | *Pithecia* = 1 | *Pithecia* = 0&1 |
| 246 | *Perupithecus* = 1 | *Perupithecus* = 2 |
|  | *Paanamacebus* = 1 | *Panamacebus* = 0 |
| 247 | *Tremacebus* = 1 | *Tremacebus* = 0 |
|  | *Panamacebus =* 1&2 | *Panamacebus* = 0 |
| 248 | *Callicebus* = 2 | *Callicebus* = 1 |
|  | *Aotus = 2* | *Aotus* = 1 |
| 249 | *Mazzonicebus =* 0&1 | *Mazzonicebus* = 1 |
|  | *Tremacebus* = 1 | *Tremacebus* = ? |
|  | *Carlocebus* = 1 | *Carlocebus* = 0&1&2 |
| 251 | *Mazzonicebus* = ? | *Mazzonicebus* = 0 |
| 253 | no changes | no changes |
| 254 | no changes | no changes |
| 255 | no changes | no changes |
| 256 | *Callimico* = ? | *Callimico =* 2 |
| 257 | *Cebuella* = 0 | *Cebuella =* 1 |
|  | *Callithrix* = 0 | *Callithix =* 1 |
|  | *Pithecia* = 2 | *Pithecia =* 1&2 |
|  | *Alouatta* = 0 | *Alouatta =* 1 |
|  | *Mazzonicebus* = 0&1 | *Mazzonicebus =* 1 |
|  | *Cebus* = 2 | *Cebus =* 1&2 |
|  | *Branisella* = 0 | *Branisella =* 1 |
| 259 | *Alouatta* = 0 | *Alouatta =* 0&1 |
|  | *Stirtonia* = 2 | *Stirtonia =* 1 |
|  | *Mazzonicebus* = 2&3 | *Mazzonicebus =* 2 |
|  | *Aotus* = 0 | *Aotus =* 0&1 |
|  | *Cebus* = 1 | *Cebus =* 1&2&3 |
|  | *Saimiri* = 3 | *Saimiri =* 2&3 |
| 263 | *Acrecebus* = 0 | *Acrecebus* = ? |
|  | *Dolichocebus* = 1 | *Dolichocebus* = 0 |
|  | *Homunculus* = 1 | *Homunculus* = 0 |
| 266 | *Mazzonicebus* = ? | *Mazzonicebus* = 1 |
| 267 | *Tremacebus* = 0&1 | *Tremacebus* = 0 |
| 268 | *Soriacebus* = 2 | *Soriacebus* = 0&1&2 |
|  | *Cannanimico* = 2 | *Cannanimico* = 1 |
|  | *Tremacebus* = 1 | *Tremacebus* = 2 |
| 269 | *Mazzonicebus* = ? | *Mazzonicebus* = 0 |
| 353 | *Dolichocebus* = ? | *Dolichocebus* = 0 |
| 355 | *Dolichocebus* = ? | *Dolichocebus* = 1 |
| 358 | *Dolichocebus* = ? | *Dolichocebus* = 1 |
| 360 | *Dolichocebus* = ? | *Dolichocebus* = 2 |
| 362 | *Dolichocebus* = ? | *Dolichocebus* = 1 |
| 364 | *Dolichocebus* = ? | *Dolichocebus* = 2 |
| 366 | *Dolichocebus* = ? | *Dolichocebus* = 0 |
| 368 | *Dolichocebus* = ? | *Dolichocebus* = 0 |
| 370 | *Dolichocebus* = ? | *Dolichocebus* = 0 |
| 371 | *Dolichocebus* = ? | *Dolichocebus* = 0 |
| 372 | *Dolichocebus* = ? | *Dolichocebus* = 0 |
| 373 | *Dolichocebus* = ? | *Dolichocebus* = 0 |
| 375 | *Cebus* = 2 | *Cebus* = 1 |
| 401 | *Callicebus* = 1 | *Callicebus =* 0&1 |
| 413 | *Branisella* = 0 | *Branisella* = 1 |

**REFERENCES**

Bloch, J.I, Woodruff, E.D., Wood, A.R., Rincón, A.F., Harrington, A.R, Morgan, G.S., Foster, D.A., Montes, C., Jaramillo, C.A., Jud, N.A., Jones, D.S., & Macfadden, B.J. (2016). First North American fossil monkey and early Miocene tropical biotic interchange. *Nature* 533: 243-246.

Fleagle, J.G. (1990). New fossil platyrrhines from the Pinturas Formation, Southern Argentina. *Journal of Human Evolution*, 19, 61-85.

Fleagle, J.G., & Bown, T.M. (1983). New primate fossils from late Oligocene (Colhuehuapian) localities of Chubut Province, Argentina. *Folia Primatologica*, 41, 240-266.

Fulwood, E.L., Boyer, D.M., & Kay, R.F. (2016) Stem members of Platyrrhini are distinct from catarrhines in at least one derived cranial feature. *Journal of Human Evolution*, 100:16-24

Horovitz, I., MacPhee, R.D.E., 1999. The Quaternary Cuban platyrrhine *Paralouatta*

*varonai* and the origin of Antillean monkeys. *Journal of Human Evolution*, 36, 33‐68.

Kay, R.F. (2015). Biogeography in deep time – What do phylogenetics, geology, and paleoclimate tell us about early platyrrhine evolution? *Molecular Phylogenetics and Evolution*, 82, 358-374.

Kay, R.F., Williams, B.A., Ross, C.F., Takai, M.. & Shigehara, N., 2004. Anthropoid origins: a phylogenetic analysis. In: Ross, C.F., Kay, R.F. (Eds.), Anthropoid Origins: New Visions. Kluwer/Plenum, New York, pp. 91‐135.

Kay, R.F., Fleagle, J.G., Mitchell, T.R.T., Colbert, M.W., Bown, T.M., & Powers, D.W. (2008). The anatomy of *Dolichocebus gaimanensis*, a primitive platyrrhine monkey from Argentina. *Journal of Human Evolution*, 54, 323-382.

Kay, R.F., Gonzales, L.A., Salenbien, W., Cooke, S.B., Angel, L., Rigsby, C., & Baker, P.A. (2019). *Parvimico materdei* gen. et sp. nov.: A new platyrrhine from the Early Miocene of the Amazon Basin, Peru. *Journal of Human Evolution*, 134, 102628.

Marivaux, L., Adnet, S., Altamirano-Sierra, A.J., Boivin, M., Pujos, F., Ramdarshan, A., Salas-Gismondi, R., Tejada-Lara, J.V., & Antoine, P.O. (2016). Neotropics provide insights into the emergence of New World Monkeys: new dental evidence from the late Oligocene of Peruvian Amazonia. *Journal of Human Evolution*, 97, 159–175.

Novo, N.M, Tejedor, M.F., Pérez, M.E., & Krause, J.M., (2017). New primate locality from the early Miocene of Patagonia, Argentina. *American Journal of Physical Anthropology*, 164, 861–867.

Rosenberger AL (2019) *Dolichocebus gaimanensis* Is Not a Stem Platyrrhine. Folia Primatol 90:494-506.

Ross, C.F., 1994. The craniofacial evidence for anthropoid and tarsier relationships. In: Fleagle, J.G., Kay, R.F. (Eds.), Anthropoid Origins: The Fossil Evidence. Plenum Press, New York, pp. 469‐547.

Ross, C., Williams, B.A., & Kay, R.F., 1998. Phylogenetic analysis of anthropoid relationships. *Journal of Human Evolution*. 35, 221‐306.

Simons EL (2001) The cranium of *Parapithecus grangeri*, an Egyptian Oligocene anthropoidean primate. Proceedings of the National Academy of Sciences of the United States of America 98(14), 7892–7897

Takai, M., Nishimura, T., Shigehara, N., & Setoguchi, T. (2009). Meaning of the canine sexual dimorphism in fossil owl monkey *Aotus dindensis*, from the middle Miocene of La Venta, Colombia. In T. Koppe, G. Meyer, K.W. Alt (Eds), *Comparative Dental Morphology. Front Oral Biology*. Basel: Karger. 13, 55-59

Tejedor, M.F. (2005). New specimens of *Soriacebus adrianae*, with comments on pitheciin primates from the Miocene of Patagonia. *Ameghiniana*, 42(1), 249-251.
