# Supplementary material for: The Earliest Known Radiation of Pitheciine Primates
Source: Am J Primatol. 2025 May 16;87(5):e70040. doi: 10.1002/ajp.70040 (PMC12082270; doi:10.1002/ajp.70040)
Supplement: Supplementary file 2 — Appendix 2. List of morphological characters used in the present phylogenetic analysis. [file AJP-87-e70040-s003.docx]

**APPENDIX 2. List of characters.**

**Cranial characters**

*Paranasal Sinuses*

1. Ethmofrontal sinus (Type II): 0= present; 1= absent.

2. Splenofrontal sinus (Type I): 0= present; 1= absent.

3. Maxillary sinus: 0= present; 1= absent.

4. Anterior ethmoidal sinus: 0= present; 1= absent.

5. Sphenoidal sinus: 0= present; 1= absent.

*Zygomatic region*

6. Zygomatic-facial foramen: 0= small relative to M1 breadth; 1= large relative to M1 breadth; 2= very large relative to M1 breadth.

7. Zygomatic arch position: 0= above the alveolar border of the maxilla; 1= below the alveolar border.

8. Extent of inferior orbital fissure: 0= ventrolateral limit of the inferior orbital fissure does not reach the zygomatic arch; 1= the ventrolateral limit of the inferior orbital fissure reaches the zygomatic arch.

9. Zygomatic-parietal contact at pterion: 0= no postorbital closure; 1= zygomatic-parietal contact; 2= alisphenoid-frontal contact.

*Lacrimal Region*

10. Position of lacrimal foramen: 0= outside orbital margin; 1= within the orbit or on the rim.

11. Extraorbital exposure of the lacrimal: 0= lacrimal fossa is completed

anteriorly by maxillary; 1= lacrimal has some facial exposure; 2= lacrimal contacts

nasal (excludes maxillary-frontal contact).

12. Zygomatic-lacrimal contact (cranial character 26 in Kay et al., 2004):

0= present on ventral orbital rim; 1= absent on ventral orbital rim.

13. Contact between lacrimal and palatine bones (cranial character 28 in

Kay et al., 2004): 0= contact present; 1= lacrimal and palatine separated; contact

between frontal and maxilla contact (or in some taxa, by a small os planum of the

ethmoid); 2= separated by a large os planum.

*Facial region*

14. Position of the infraorbital foramen relative to the Frankfurt horizontal plane: 0= posterior to P4; 1= positioned above P4 through P3; 2= positioned above P2.

15. Angle of cranial kyphosis: 0= (≤ 140°); 1= (>140°, < 155°); 2= (≥ 155°).

16. Nasal fossa width (character 25 in Horovitz, 1999): 0= narrower than

the palate width; 1= broader than the palate width.

17. Nasal capsule: 0= *processus alaris* superior present; 1= *processus alaris* superior absent.

18. Snout length: 0= long snout; 1= short snout.

19. Maxilla depth: 0= deep; 1= shallow.

20. Inter-incisor diastema width: 0= broad and wider than that of extant haplorhines; 1= narrow, haplorhine-like.

21. Ascending wing of premaxilla: 0= narrow; 1= broad.

*Temporomandibular region*

22. Postglenoid foramen: 0= absent; 1= small; 2= large.

23. Temporomandibular joint morphology: 0= biconcave and transversely wide; 1= anteroposteriorly oriented.

24. Postglenoid process size (100 times postglenoid process length divided by prosthion-inion length): 0= weak or absent (< 0.39); 1= strong (≥ 0.39, < 0.69); 2= very strong (≥ 0.69).

*Pterygoid and palatal region*

25. Palate shape: 0= v-shaped (the distance between lingual surfaces of

the upper canines divided by the between the lingual surfaces of the upper second

molars is < 0.39); 1= intermediate (ratio values of ≥ 0.39, ≤ 0.64); 2= approaches

parallel (ratio values > 0.64).

26. Interpterygoid fossa (Du Brul, 1965): 0= deep; 1= shallow.

27. Length of medial pterygoid plate: 0= long medial pterygoid plate extending one-third to one half of the distance to the anterior surface of the bulla enclosing a large fossa between medial and lateral pterygoids; 1= short but distinct from lateral pterygoid plate for its entire dorsoventral extent. Ventrally, there is a hamular process; more dorsally the plate merges with the lateral plate or if distinct, the fossa is slit-like; 2= medial pterygoid plate entirely absent, or reduced to a low rugosity. Only the hamulus is present.

28. Encroachment of the auditory bulla on the pterygoid fossa: 0= no encroachment; 1= encroachment by the anterior accessory cavity; 2= present and formed by the tympanic cavity.

29. Nature of contact between the lateral pterygoid plate and the bulla wall: 0= absent; 1= laminar; 2= abutting.

30. Extent of contact between the lateral pterygoid plate and the bulla wall: 0= slight; 1= or very extensive.

31. Pyramidal process of palate and post-alveolar notch: 0= no post-alveolar notch between the pyramidal process and the maxillary tuberosity; 1= offset from maxillary tuberosity by a distinct post-alveolar notch.

32. Mediolateral position of pyramidal processes (100 times the ratio of inter-pyramidal breadth to outer M1 palate breadth): 0= medially placed (≤ 43); 1= intermediate (> 43, ≤ 64); 2= laterally placed (> 64).

33. Posterior palatine torus: 0= present; 1= absent.

34. Posterior nasal spine: 0= reduced or absent; 1= small but distinct; 2= robust and long.

35. Posterior extent of the turbinates: 0= extend posterior to the palatine; 1= completely anterior to the palatine.

36. Angle of the incisive canal in palate: 0= obliquely oriented with respect to the plane of the palate; 1= more closely resembles a right angle with the palate.

*Temporal fossa*

37. Temporal emissary foramen: 0= present and large; 1= small or absent.

*Nucal region*

38. Paroccipital processes: 0= forms a distinct shelf or process; 1= forms a raised ridge; 2= weak or absent.

39. Pneumatization of mastoid (cranial character 3 in Kay et al., 2004): 0= absent; 1= present.

*Orbital region*

40. Lateral cranial profile at glabella: 0= depressed; 1= flat; 2= convex.

41. Interorbital fenestra: 0= absent; 1= present.

42. Size of orbits: 0= small; 1= large; 2= extremely large.

43. Orbital convergence: 0= (< 55°); 1= (55°-65°); 2= (> 65°).

44. Interorbital breadth: 0= narrow; 1= broad; 2= extremely broad.

45. Exposure of vomer in orbit: 0= unexposed; 1= exposed.

46. Postorbital closure: 0= none; 1= postorbital bar present; 2= postorbital septum present.

47. Composition of the postorbital septum: 0= zygomatic forms most of the septum; 1= frontal forms most of the septum.

48. Position of interorbital constriction relative to olfactory tract: 0= absent; 1= present below olfactory tract.

49. Foramen rotundum: 0= superior orbital fissure transmits maxillary nerve; 1= separate foramen (f.rotundum) for maxillary nerve.

50. Metopic suture in adults: 0= unfused; 1= fused.

*Ear region*

51. Cochlear housing as exposed in middle ear: 0= singular; 1= dual.

52. Transbullar septa: 0= ventrolateral region of middle ear without septa; 1= anteroventral region with septa.

53. Transverse septum arising from the cochlear housing: 0= absent; 1= present and forming the lateral wall of an anterior accessory cavity pneumatized from the tympanic cavity; 2= present and forming the lateral wall of an anterior accessory cavity pneumatized from the epitympanic recess.

54. Extent of pneumatization of anterior accessory cavity: 0= anterior accessory cavity lies anterior to the tympanic cavity and is not trabeculated; 1= anterior accessory cavity extends medial to the tympanic cavity, and is trabeculated.

55. Presence or absence of perbullar pathway for the internal carotid artery: 0= absent; 1= present and formed exclusively by the petrosal bone.

56. Anteroposterior location of posterior carotid foramen in bulla: 0= posterior to line joining midpoints of tympanic bones; 1= anterior to this line.

57. Mediolateral position of posterior carotid foramen in bulla: 0= medial; 1= midline of the bulla; 2= lateral.

58. Ventrodorsal position of the carotid foramen in the bulla: 0= dorsal, adjacent to basioccipital or mastoid bone; 1= ventral.

59. Position of posterior carotid foramen relative to fenestra cochleae: 0= posterior; 1= ventral; 2= anterior.

60. Position of the internal carotid canal relative to the fenestra cochleae: 0= runs across ventral lip of the fenestra cochleae, shielding it from ventral view when a canal is present; 1= internal carotid canal does not shield the fenestra cochleae from ventral view.

61. Position of the portion of the internal carotid / promontory artery (or its accompanying nerves) lying on the promontorium anterior to the fenestra

cochleae: 0= on ventrolateral surface of promontorium; 1= contacting only the cupula of the cochlea.

62. Size of stapedial and promontory canals: 0= both stapedial and promontory canals are large; 1= stapedial slightly smaller than promontory; 2= stapedial highly reduced or absent altogether; 3= stapedial larger than promontory; 4= both promontory and stapedial canals absent.

63. Morphology of promontory canal, when present: 0= open trough; 1= complete canal.

64. Canal for internal carotid artery or nerves: 0= absent; 1= present.

65. Position of ventral edge of the tympanic bone: 0= intrabullar or aphaneric; 1= extrabullar or phaneric.

66. The shape of the tympanic bone: 0= ribbon-like or only slightly expanded; 1= laterally expanded into a collar or tube.

67. Morphology of annular bridge: 0= Linea semicircularis or partial anular bridge formed on the entotympanic bulla; 1= Linea semicircularis formed on the petrosal bulla; 2= a complete annular bridge present.

68. Flange of basioccipital overlapping medial bulla wall: 0= absent or minimal; 1= extensive.

69. Basioccipital stem:0= narrow; 1= broad.

70. Suprameatal foramen: 0= absent; 1= present, small and in the posterior root of the zygomatic arch; 2= present, large, and above the external auditory meatus.

71. Patent parotic fissure: 0= present; 1= absent.

72. Enclosure of intratympanic portion of facial nerve in a bony canal: 0= no canal, facial runs in a sulcus; 1= bony canal present.

73. Epitympanic crest: 0= absent; 1= present.

*Brain and internal cranial characters*

74. Tentorium cerebelli ossification: 0= absent; 1= present.

75. Vascular canal connecting sigmoid sinus with subarcuate fossa: 0= absent; 1=

present.

76. Size of olfactory bulbs: 0= large olfactory lobe; 1= moderate olfactory lobe; 2= small olfactory lobe.

77. Relative brain size: 0= small; 1= large; 2= very large.

*Mandible*

78. Symphyseal orientation: 0= more horizontal orientation of planum alveolare; 1= more vertically oriented relative to planum alveolare.

79. Lateral profile of mandible [ratio of mandible depth (measured buccally) at p2 and m2]: 0= superior and inferior border of the mandibular corpus are essentially parallel from the premolar to the mandibular angle (≤ 1.26); 1= inferior border deepens posteriorly (> 1.26, ≤ 1.72); 2= “hyper-deep” (> 1.72).

80. Mandibular corpus depth: 0= shallow; 1= deep.

81. Symphyseal fusion in young adult: 0= absent; 1= present.

82. Coronoid height relative to condyle: 0= very far above condyle; 1= above the level of condyle; 2= slightly above or equal to coronoid.

83. Condyle height relative to tooth row: 0= at level of tooth row; 1= slightly above; 2= well above tooth row.

84. Angle of the mandible: 0= hook-shaped angle; 1= moderately expanded angle; 2= extremely expanded angle.

85. Depth of the coronoid-condylar notch: 0= deep; 1= shallow.

**Permanent dentition**

*Lower incisors*

86. Lower incisor number: 0= three; 1= two; 2= one: i1 present, i2 absent; 3= lower incisors absent.

87. Lower incisor occlusal arrangement: 0= edges wear flat producing an arcuate battery from lateral perspective; 1= cusp tips staggered.

88. Lower incisor crown spacing: 0= no space; 1= spaces present between crowns.

89. i2-c1 diastema: 0= present; 1= absent.

90. i1-2 size (ratio of i1-2 area to m2 area): 0= very small (≤ 0.69); 1= moderate sized (≥ 0.70, ≤ 1.07); 2= large (> 1.07).

91. i1: i2 proportions (ratio of i1 area to i2 area): 0= i1 much smaller than i2 (< 0.71); 1= i1 smaller than i2 (≥ 0.71, < 0.78); 2= i1 almost as large as i2 (≥

0.78, < 1.00); 3= i1 > i2 (≥ 1.01).

92. i1 crown width (spatulate incisors only): 0= considerably wider (mesiodistally) than root (spatulate); 1= narrow at apex, but still wider than root; 2= "styliform" (crown apex approximately the same width as the cervical margin).

93. i2 crown cross-sectional shape (ratio of mesiodistal length to buccolingual breadth): 0= rounded oval (≥ 0.64); 1= mesiodistally compressed (< 0.64).

94. Lower incisor crown height (crown heights judged from cementoenamel junction to crown tip on the buccal surface): 0= low crowned; 1=

moderately high crowned; 2= high crowned.

95. i1-2 crown buccal outline: 0= gently curved in lateral perspective; 1= acutely curved.

96. Lower incisor roots: 0= erect or vertical; 1= slightly procumbent; 2= very procumbent.

97. Lower incisor crowns: 0= erect or vertical; 1= procumbent; 2= very procumbent.

98. Tooth comb: 0= absent; 1= with three teeth; 2= with two teeth.

99. i1 crown shape: 0= spatulate; 1= lanceolate, pointed.

100. i2 heel development (a lingual swelling at the base of crown): 0= heel absent; 1= heel present.

101. Incisor lingual enamel: 0= well developed; 1= poorly developed or absent.

102. Lower incisor lingual cingulum: 0= absent to weak; 1= strong but incomplete; 2= strong and complete.

103. i1 area to m1 area: 0= i1 very small (ratio ≤ 0.32); 1= moderately enlarged (> 0.32, ≤ 0.40); 2= very enlarged (> 0.40).

*Lower canines*

104. Female c1 size (area relative to molars): 0= very small (c1 / m1 < 0.40); 1= moderate (≥ 0.4, < 0.80); 2= large (≥ 0.80, ≤ 1.20); 3= very large (> 1.20).

105. c1 dimorphism (square root male c1 area divided by square root of female c1 area): 0= low (< 1.07); 1= moderate (≥ 1.07, < 1.17); 2= high (≥ 1.17).

106. c1 cross-sectional shape: 0= rounded oval (mesiodistal / buccolingual, > 1.00, < 1.90); 1= mesiodistally compressed (ratio ≥ 1.90).

107. c1 lingual crest development: 0= rounded; 1= sharp.

108. Canine paracristid (not scored if species has canine incorporated into a tooth comb): 0= oblique to occlusal plane; 1= nearly horizontal to occlusal plane; 2= forms part of cropping mechanism with i1-2.

109. Canine height (females): 0= low, squat; 1= narrow, short; 2= tall, at or above tooth row.

*Lower premolars*

110. P1/p1: 0= present; 1= absent.

111. p2: 0= present; 1= absent.

112. p2 roots: 0= single; 1= double.

113. p3-4 roots: 0= p3 single, p4 single; 1= p3 single, p4 double; 2= p3 double, p4 double.

114. Premolar crowding (overlapping of crowns): 0= no crowding; 1= slightly crowded; 2= very crowded.

115. p3 paraconid: 0= large; 1= small; 2= absent or extremely small.

116. p4 paraconid: 0= large; 1= small; 2= absent or extremely small.

117. p4 paraconid position: 0= mesial to protoconid; 1= mesiolingual,

between protoconid and metaconid; 2= mesial to metaconid; widely spaced from

metaconid; 3= twinned with metaconid.

118. p3-4 cristid obliqua: 0= absent; 1= weak; 2= strong.

119. p2 protoconid height and shape: 0= slender, projects above protoconids of p3-4; 1= massive, projects above protoconids of p3-4; 2= not projecting, in line with p3; 3= extremely short, shorter than p3.

120. p4 metaconid position: 0= close to protoconid; 1= widely spaced from protoconid.

121. p2 metaconid size: 0= absent or trace; 1= small; 2= large.

122. p3 metaconid size: 0= absent or trace; 1= small; 2= large.

123. p4 metaconid size: 0= absent or trace; 1= small; 2= large, almost as tall as protoconid.

124. p4 trigonid lingual wall: 0= basin closed by a premetacristid; 1= open with premetacristid absent or short.

125. p3 entoconid and lingual talonid crest: 0= absent; 1= lingual talonid crest present but an entoconid does not stand out above it; 2= entoconid is a small discrete cusp.

126. p4 entoconid and lingual talonid crest: 0= absent; 1= lingual talonid crest present but an entoconid does not stand out above it; 2= entoconid is a small discrete cusp.

127. p4 lateral and medial protocristid: 0= continuous between metaconid and protoconid; 1= discontinuous between metaconid and protoconid.

128. p3 lateral protocristid orientation: 0= transversely oriented; 1= distolingually oriented.

129. p4 lateral protocristid: 0= present; 1= absent.

130. p4 lateral protocristid orientation: 0= transversely oriented; 1= distolingually oriented.

131. p3-4 posterior trigonid wall: 0= complete (taxa without metaconids are assigned this character state); 1= deeply notched.

132. p3-4 hypoconid size: 0= large; 1= crestiform, small, or absent.

133. p3-4 hypoconid (or distal terminus of oblique cristid) position: 0= distal to protoconid; 1= distal to metaconid, or between protoconid and metaconid.

134. p4 talonid breadth: 0= narrow; 1= broad.

135. p4 hypocristid shearing development: 0= weak or absent; 1= strong.

136. p2 buccal cingulum development: 0= absent; 1= incomplete, broken at protoconid and hypoconid; 2= complete.

137. Lower premolar inflation: 0= cusps marginal, not basally inflated; 1= crown surfaces constricted, cusp margins sloping.

138. p4 exodaenodonty: 0= not exodaenodont; 1= slightly exodaenodont; 2= very exodaenodont.

139. p4 talonid length (ratio of midline mesiodistal length of trigonid to mesiodistal length of talonid): 0= extremely short or non-existent (tri:tal ≥ 1.61); 1= short (much shorter than trigonid) (tri:tal ≥ 1.27, < 1.61); 2= equal or slightly shorter in length to trigonid (tri:tal ≥ 0.92, < 1.27); 3= talonid longer than trigonid (tri:tal < 0.91).

140. p4 anterobuccal cingulum development: 0= absent or trace; 1= strong.

141. p4 postprotoconid ridge: 0= weak or absent; 1= present; 2= very strong.

142. p4 postmetaconid ridge: 0= weak or absent; 1= moderate; 2= very strong.

143. p4 paraconid height: 0= low; 1= moderate; 2= high (nearly as high as protoconid).

144. p3‐4 protoconid height: 0= p3 much lower than p4; 1= p3 equal or slightly lower than p4; 2= p3 higher than p4.

145. Ratio of p3 to p4 area: 0= (0.45-0.59); 1= (0.60-0.69); 2= (0.70-0.79); 3= (> 0.80, ≤ 1.10); 4= (> 1.10).

146. p4 mesiodistal Length / buccolingual Width: 0= (< 0.95); 1= (≥ 0.96, ≤ 1.14); 2= (≥ 1.15, < 1.20); 3= (≥ 1.21, ≤ 1.35); 4= (≥ 1.36, ≤ 1.46); 5= (≥ 1.47).

147. p4 to m1 area: 0= (< 0.62); 1= (≥ 0.63, ≤ 0.72); 2= (≥ 0.73, ≤ 0.82); 3= (≥ 0.83, ≤ 0.92); 4= (≥ 0.93, ≤ 1.02); 5= (≥ 1.03).

148. p3-4 root orientation: 0= p3-4 roots aligned mesiodistally; 1= p3 root shifted laterally, p4 mesial root aligned mesiodistally; 2= p3 roots aligned mesiodistally, p4 mesial root shifted laterally. [Score as missing if roots are singular].

*Lower Molars*

149. M3/m3: 0= present; 1= absent.

150. m1 root number: 0= one; 1= two.

151. m2 root number: 0= one; 1= two.

152. m3 root number: 0= one; 1= two.

153. m2 trigonid width (ratio of buccolingual breadths of trigonid and talonid): 0= much wider than talonid (≥ 1.11); 1= widths similar (< 1.11, > 0.90); 2= much narrower than talonid (≤ 0.90).

154. m1 trigonid length: 0= m1 trigonid short on the lingual side; 1= m1 with elongate lingual face.

155. m3 trigonid width (based on relative buccolingual breadths): 0= much wider than talonid (> 1.20); 1= trigonid and talonid widths similar (1.20-1.05); 2= trigonid narrower than talonid (< 1.05).

156. m1 paraconid position: 0= mesial to protoconid; 1= mesiolingual, between protoconid and metaconid; 2= mesial to metaconid but widely spaced from it; 3= twinned with metaconid.

157. m2 paraconid position: 0= mesial to protoconid; 1= mesiolingual, between protoconid and metaconid; 2= mesial to metaconid but widely spaced from it; 3= twinned with metaconid.

158. m3 paraconid position: 0= mesial to protoconid; 1= mesiolingual, between protoconid and metaconid; 2= mesial to metaconid but widely spaced from it; 3= twinned with metaconid.

159. m1 parastylid: 0= absent; 1= present.

160. Molar metastylid: 0= absent; 1= small; 2= large.

161. m3 hypoconulid: 0= single; 1= double.

162. m3 heel: 0= absent; 1= narrower than talonid; 2= approximately equal in width to talonid.

163. Molar occlusal enamel surface: 0= smooth; 1= slightly crenulated; 2= highly crenulated.

164. m1 trigonid height (ratio of trigonid height to talonid height): 0= trigonid higher than talonid (≥ 1.20); 1= trigonid slightly higher than talonid (< 1.20, ≥ 1.10); 2= trigonid and talonid of similar height (< 1.10).

165. m1-2 cusp relief (ratio of hypoflexid height to hypoconid height, measured buccally): 0= low (< 1.20); 1= moderate (≥ 1.20, < 1.35); 2= high (> 1.35).

166. m1 trigonid lingual configuration: 0= open; 1= closed.

167. m1 metaconid position: 0= lingual to protoconid; 1= slightly distolingual to protoconid.

168. m1-2 paraconid development: 0= absent; 1= small; 2= large.

169. m1-2 lateral protocristid orientation: 0= runs toward metaconid; 1= runs toward hypoflexid.

170. m1 distal trigonid wall: 0= complete; 1= deeply notched by protoconid/metaconid sulcus; 2= medial and lateral protocristid do not meet but

no sulcus is discerned.

171. m2 distal trigonid wall: 0= complete; 1= deeply notched by a sulcus between protoconid and metaconid; 2= medial and lateral protocristid do not meet but no sulcus is present.

172. m1 wear facet X: 0= present; 1= absent.

173. m2 wear facet X: 0= present; 1= absent.

174. m1-2 entoconid: 0= absent or very low, 1= lower than metaconid; 2= large.

175. m1-2 postentoconid sulcus: 0= prominent; 1= shallow sulcus; 2= absent.

176. m1 hypoconulid size: 0= large; 1= moderate; 2= small; 3= absent.

177. m2 hypoconulid size: 0= large; 1= moderate; 2= small; 3= absent.

178. m3 hypoconulid size: 0= large; 1= moderate; 2= small; 3= absent.

179. m1-2 hypoconulid position: 0= twinned to entoconid; 1= near midline; 2= slightly buccal to midline.

180. m1-2 cristid obliqua development: 0= weak (rounded); 1= strong (trenchant); 2= very strong (very trenchant).

181. m1 cristid obliqua orientation: 0= reaches trigonid wall at a point distal to protoconid; 1= reaches trigonid wall at a point distolingual to protoconid; 2= reaches trigonid wall at a point distal to metaconid.

182. m2 cristid obliqua orientation: 0= reaches trigonid wall at a point distal to protoconid; 1= reaches trigonid wall at a point distolingual to protoconid; 2= reaches trigonid wall at a point distal to metaconid.

183. m1 cristid obliqua terminus: 0= runs to base of trigonid; 1= runs part way up the distal trigonid wall; 2= connects with protoconid tip or protocristid; 3= connects with metaconid.

184. m2 cristid obliqua terminus: 0= runs to base of trigonid; 1= runs part way up the distal trigonid wall; 2= connects with protoconid tip or protocristid; 3= connects with metaconid.

185. m3 cristid obliqua terminus: 0= runs to base of trigonid; 1= runs part way up the distal trigonid wall; 2= connects with protoconid tip or protocristid; 3= connects with metaconid.

186. m1-2 centroconid development: 0= present; 1= absent.

187. m1-2 hypocristid development: 0= absent or seen only as a trace; 1= weak; 2= strong.

188. m3 hypocristid development: 0= absent or seen only as a trace; 1= weak; 2= strong.

189. m1-2 talonid, lingual configuration: 0= open; 1= closed, notched lingually; 2= closed, no notch.

190. m1-2 distal fovea: 0= absent; 1= present.

191. Molar cusp inflation: 0= cusps not inflated, marginally positioned; 1= very inflated.

.

192. m1-2 buccal cingulum development: 0= absent to trace; 1= partial, broken at protoconid and hypoconid; 2= complete.

193. m1 hypoflexid depth: 0= very shallow; 1= moderate; 2= deep.

194. m2 hypoflexid depth: 0= very shallow; 1= moderate; 2= deep.

195. Ratio of m2 length to m3 length: 0= m3 much longer than m2 (0.71‐0.80); 1= m3 longer than m2 (0.81-0.90); 2= m3 equal to m2 (0.91-1.00); 3= m3 smaller than m2 (1.01-1.12); 4= m3 much smaller than m2 (≥ 1.13). Score as “5” if m3 absent.

196. m1 length: 0= (< 2.5 mm); 1= (≥ 2.5, < 3.8 mm); 2= (≥ 3.8, ≤ 6.0 mm); 3= (> 6.0 mm).

197. m1 L/W: 0= (1.0-1.15); 1= (1.16-1.22); 2= (1.23-1.32); 3= (> 1.33).

198. m1-2 entoconid position relative to hypoconid: 0= transverse to hypoconid; 1= distal to hypoconid.

*Upper Incisors*

199. I1-I2 interstitial contact: 0= absent, teeth widely spaced; 1= present as narrow contact; 2= I2 tightly packed against I1, with I1 preparacrista abbreviated.

200. I1-I1 interstitial contact: 0= present; 1= absent: a wide space occurs in the midline between these teeth.

201. I2–C1 diastema: 0= present; 1= absent.

202. Ratio of I1 area to I2 area: 0= areas approximately equal (≤ 1.00); 1= I1 slightly larger than I2 (> 1.00, < 1.40); 2= I1 much larger than I2 (≥ 1.40).

203. I1 size (I1 area: M1 area): 0= small (≤ 0.50); 1= moderate (> 0.50, < 0.56); 2= large (≥ 0.56).

204. I1 occlusal shape (mesiodistal length / buccolingual breadth): 0= rounded oval (< 1.05); 1= buccolingually compressed (≥ 1.05, ≤ 1.30); 2= extremely

compressed (> 1.30).

205. I2 occlusal shape (mesiodistal length / buccolingual breadth): 0= rounded oval (≤ 1.05); 1= slightly compressed (> 1.05, < 1.30); 2= extremely compressed ≥ 1.30).

206. I1 crown shape: 0= spatulate; no apparent occlusal cusp, mesial and distal edges continuous and rounded; 1= semi-spatulate, central cusp present but blunt with discernable mesial and distal occlusal crests; 2= central occlusal cusp pointed, occlusal edges steep.

207. I1 lingual fovea: 0= simple; 1= dual with mid-crown pillar.

208. I1 occlusal edge orientation (spatulate incisors only): 0= occlusal edge orthogonal to long axis of root; 1= occlusal edge wears at a steep angle to long axis of root; 2= crown with pronounced mesial asymmetry (= mesial process) in unworn state.

209. I1-2 lingual cingulum: 0= weak, discontinuous; 1= narrow, continuous; 2= strong.

210. I1 basal lingual cusp: 0= absent; 1= present.

211. I1and I2 buccal cingulum: 0= absent; 1= present.

*Upper canines*

212. C1 cross-sectional shape (ratio of maximum length in the occlusal plane to maximum breadth in the occlusal plane at right angles to maximum length): 0= oval (≥ 1.16); 1= rounded (< 1.16).

213. Upper canine occlusion: 0= C1 wears against P1-2; 1= C1 wears against P2; 2= C1 wears against P2-3; 3= C1 wears against P3.

214. C1 mesial groove (females): 0= shallow or absent; 1= deep.

215. C1 lingual cingulum: 0= weak or absent; 1= strong; 2= very strong.

*Upper premolars*

216. P2 root number: 0= one; 1= two; 2= three. If tooth is absent, character scored as ‘?.’

217. P3 root number: 0= one; 1= two; 2= three.

218. P4 root number: 0= one; 1= two; 2= three.

219. Ratio of P2 area to P3 area: 0= P2 very small (≤ 0.85); 1= P2 small (> 0.85, < 0.95); 2= P2 equal (≥ 0.95). If tooth is absent, character scored "?".

220. Ratio of P4 area to M1 area: 0= P4 << M1 (≤ 0.66); 1= P4 < M1 (0.67-0.76); 2= P4 = M1 (0.77-1.05); 3= P4 > M1 (> 1.06).

221. (modified to Marivaux et al. 2016, Ch. 221) Occlusal outline of P2: 0= triangular; 1= suboval, with the larger axis buccolingually; 2= suboval, with the larger axis mesiodistally; 3= rounded.

222. P3-4 trigon/talon proportions: 0= trigon and talon proportions similar; 1= trigon much shorter than talon with the protocone situated on the mesial aspect of the crown.

223. P3 protocone: 0= present; 1= absent.

224. P4 metacone: 0= absent; 1= present.

225. P4 protocone: 0= low relative to paracone; 1= high relative to paracone.

226. P2 protocone: 0= present as discrete cusp; 1= absent or indistinguishable from lingual cingular ridge.

227. Premolar hypocones: 0= absent; 1= present on P4 only; 2= present on P3-4; 3= present on P2-4.

228. P4 hypocone: 0= absent or trace; 1= bump on postprotocone crista or postcingulum; 2= distinct cusp on distal margin.

229. P4 paraconule: 0= large; 1= small; 2= absent.

230. P3-4 parastyles: 0= present; 1= weak or absent.

231. P3-4 metastyles: 0= weak or absent; 1= present.

232. P3-4 postprotocrista: 0=strong, reaches the distal margin and joins the postcingulum; 1= weak, short.

233. P2-3 profile of distal crown margin: 0= convex, smoothly rounded; 1= concave, “waisted” between buccal and lingual cusps.

234. P3-4 lingual cingulum: 0= absent or weak; 1= strong.

235. (modified from character 133 to Chaimanee et al., 2012). P4 occlusal outline: 0 = triangular; 1 = suboval; 2 = squared.

236. P3-4 buccal cingulum: 0= absent or weak; 1= strong.

*Upper molars*

237. M1-2 root count: 0= three, three; 1= three, two; 2= two, two.

238. M3 root count: 0= three; 1= two; 2= one.

239. M2 shape (ratio of buccolingual breadth / mesiodistal length): 0= very transverse (> 1.65); 1= transverse (≤ 1.65, > 1.30); 2= squared (≤ 1.30).

240. Ratio of M1 area to M2 area: 0= M1 >> M2 (≥ 1.40); 1= M1 > M2 (< 1.40, > 1.0); 2= M1 ≤ M2 (≤ 1.0).

241. M1-2 Nannopithex-fold: 0= absent; 1= weak; 2= strong.

242. M1-2 pseudohypocone: 0= absent; 1= small; 2= large.

243. (Marivaux et al 2016; Character 401). M1‐2 metaconule: 0= absent to indistinct; 1= small; 2= moderate; 3= large.

244. M1-2 paraconule: 0= absent; 1= small; 2= large.

245. M1-2 preprotoconule crista: 0= absent; 1= weak; 2= strong.

246. M1 hypocone size: 0= large; 1= small; 2= absent or crestiform.

247. M2 hypocone size: 0= large; 1= small; 2= absent.

248. (Marivaux et al 2016; Character 400). M1-2 hypocone position: 0= distal, far lingual to protocone; 1= distal, slightly lingual to protocone; 2= same level (mesiodistally opposed); 3= distal, slightly buccal to protocone.

249. M1-2 prehypocrista: 0= absent; 1= weak; 2= strong, reaches to the postprotocrista, encloses the talon lingually.

250. M1-2 prehypocrista orientation: 0= buccolingually towards postprotocrista; 1= buccally towards metaconule.

251. M3 prehypocrista development: 0= absent; 1= weak; 2= strong, reaches to postprotocrista to enclose the talon lingually.

252. M1 or M2 paraconule position: 0= attached to preprotocrista; 1= not attached to preprotocrista.

253. (Marivaux et al 2016; Character 413). Hypometaconulecrista (= metacrista or crista obliqua): 0= indistinct to absent; 1= moderate (not connected to protocone); 2= welldeveloped (connected to protocone or postprotocrista).

254. (Marivaux et al 2016; Character 402). M1-2 mesostyle size: 0= absent to indistinct; 1= moderate; 2= strong.

255. (Marivaux et al 2016; Character 404). M1-2 postprotocrista development: 0= strong; 1= tiny.

256. (Marivaux et al 2016; Character 412). M1-2 hypoparacrista: 0= absent; 1= weakly developed (short); 2= well-developed (high).

257. (Marivaux et al 2016; Character 411). M1-2 hypometacrista: 0= absent; 1= weakly developed (low and short); 2= well-developed (high).

258. P4-M1-2 pericone: 0= absent; 1= small; 2= large.

259. (Marivaux et al 2016; Character 414). M1-2 lingual cingulum development: 0= absent; 1= faintly visible; 2= well-defined; 3= strong.

260. M1-2 buccal cingulum development: 0= absent; 1= weak; 2= strong.

261. M1-2 premetaconule cristae: 0= absent or weak; 1= strong.

262. M1-2 postmetaconule cristae: 0= absent or weak; 1= strong.

263. M3 paraconule: 0= absent; 1= small‐moderate; 2= large.

264. Molar protocone lingual inflation: 0= not inflated; 1= slightly inflated; 2= very inflated.

265. M2 buccal expansion of paracone: 0= no expansion; 1= expanded.

266. M3 metacone: 0= absent or very small; 1= moderate (but smaller than paracone); 2= large (equal to paracone).

267. M3 hypocone: 0= absent or very small; 1= small; 2= large.

268. (Marivaux et al 2016; Character 423). M1-3 anterior cingulum: 0= strong; 1= weak; 2= absent.

269. M1 size relative to M3 (based on the ratio of areas of each tooth): 0= M1 ≥ 2.5 times the size of M3 (scored as “0” when M3 is absent); 1= M1 < 2.5, ≥ 1.5 times M3; 2= M1 < 1.5 times M3.

**Postcranial characters**

*Humeral characters*

270. Shape of humeral trochlea: 0= cylindrical, distal edge perpendicular to humeral shaft; 1= slightly conical, distal edge slightly angled to shaft; 2= conical, distal edge steeply angled to humeral shaft.

271. Relative heights of medial and lateral edges of humeral trochlea: 0= subequal—spool-shaped; 1= medial flared relative to lateral.

272. Trochleocapitular ridge: 0= absent; 1= slightly distinct; 2= moderately distinct; 3= very distinct.

273. Waisting of the trochlea: Minimum trochlear diameter (MinTD) / Maximum trochlear diameter (MaxTD), expressed as a percentage: 0= unwaisted (> 70); 1= waisted (≤ 70).

274. Width of capitulum relative to trochlea: Ventral capitulum width / ventral trochlear width expressed as a percentage (Ford, 1994): 0= (< 100); 1= (≥ 100, < 140); 2= (≥ 140, ≤ 200); 3= > 200.

275. Entepicondylar foramen: 0= present; 1= variable; 2= absent.

276. Entepicondylar foramen position: 0= over medial epicondyle; 1= above ventral trochlea; 2= above dorsal trochlea; 3= absent.

.

277. Medial epicondyle size: 0= small; 1= prominent.

278. Dorsal position of medial epicondyle: 0= parallel; 1= slight dorsal angle; 2= large dorsal angle.

279. Shape of dorsal trochlea: 0= no pronounced lips on dorsal trochlear edges; 1= both medial and lateral edges pronounced; 2= very pronounced lateral lip.

280. Dorso-epitrochlear fossa: 0= present, strong; 1= small, shallow; 2= absent.

281. Olecranon fossa shape: 0= shallow; 1= moderate; 2= deep.

282. Supinator crest: 0= prominent (extends far proximally); 1= low (terminates close to the distal end of the bone).

283. Brachialis flange: 0= broad; 1= moderate; 2= narrow.

284. Bicipital groove: 0= shallow; 1= deep.

285. Deltopectoral crest: 0= prominent; 1= low (rounded and indistinct edge, especially proximally); 2= flattened superiorly.

286. Deltotriceps crest: 0= low; 1= prominent.

287. Medial torsion of humeral head Rotation: 0= not medially rotated; 1= medially rotated.

*Wrist characters*

288. Os Centrale: 0= small; 1= large.

289. Ulnar-pisiform articulation: 0= pisiform facet = triq; 1= pisiform facet enlarged.

Femoral characters

290. Length of femoral neck (neck length measurement number 2/BSTD expressed as a percentage: 0= short (< 75); 1= moderate (75–120); 2= long (> 120).

291. Angle of femoral neck: 0= (< 60°); 1= (60°–70°); 2= (> 70°).

292. Angle of lesser trochanter LTA: 0= medial (0–30°); 1= posterior (> 30°).

293. Size of third trochanter: 0= large (third trochanter projection index: > 25); 1= moderate (third trochanter projection index: > 10, ≤ 25); 2= crestiform or absent.

294. Knee shape (anteroposterior diameter of distalfemur / mediolateral diameter of distal femur, expressed as a percentage: 0= (> 107); 1= (107-99); 2= (< 99, ≥ 71); 3= (< 71).

295. Femoral head shape: 0= spherical; 1= semicyclindrical; 2= cylindrical.

296. Anterior extension of greater trochanter: 0= no extension; 1= extension.

297. Anterior bowing of proximal femur: 0= straight; 1= slightly bowed; 2= pronounced bowing.

298. Relative length of trochanteric fossa (intertrochanteric fossa length/BSDLT), expressed as a percentage: 0= long (> 125); 1= moderate (110–125); 2= very short (< 110).

299. Intertrochanteric crest: 0= crest absent; 1= crest present.

300. Size of lesser trochanter: 0= large; 1= intermediate; 2= small.

301. Lateral border of distal femur (i.e., the lateral rim of the patellar groove: 0= low; 1= high.

302. Crista paratrochanterica on posterior femoral neck: 0= flat; 1= low ridge or cusp; 2= high ridge.

303. Projection of the femoral head relative to the greater trochanter: 0= greater trochanter projects well above the femoral head; 1= greater trochanter is at the same level as the femoral head; 2= greater trochanter well below (distal to) the femoral head.

*Limb indices*

304. Sum of lengths of humerus and radius divided by summed lengths of femur plus tibia expressed as a percentage: 0= long hindlimb (≤ 72); 1= moderate hindlimb (≥ 73, ≤ 85); 2= short hindlimb (≥ 86).

305. Ratio of humerus length to femur length expressed as a percentage: 0= (≤ 65); 1= (≥ 66, ≤ 82); 2= (≥ 83).

*Tibial characters*

306. Contact between distal tibia and fibula: 0= absent; 1= small facet; 2= extensive facet; 3= proximal fusion (synostosis).

307. Distal tibia articulation shape: 0= square; 1= triangular.

308. Shape of distal tibial articular for talus, if ‘square’: 0= narrow articular surface (width to breadth ratio < 100); 1= wider articular surface width to breadth (≥ 100, < 130); 2= wide articular surface (> 130).

309. Medial malleolus rotation: 0= none; 1= slight;2= strong.

310. Medial malleolar articulation: 0= flat; 1= anteriorly convex; 2= all convex.

311. Shape distal tibia shaft: 0= no compression; 1= anteroposteriorly compressed.

312. Tibialis posterior groove: 0= variably distinct, on the lateral side of the medial malleolus; 1= medial to a raised crest on the posterior side of malleolus.

313. Posterior border of the trochlear facet for talus: 0= flat; 1= rounded; 2= sharp.

314. Medial malleolar height of the tibia relative to the anteroposterior diameter of distal tibial shaft, expressed as a percentage: 0= (≤ 68); 1= (> 68, ≤ 101); 2= (> 101).

*Astragalar (talar) characters*

315. Position of the groove for the tendon of m. flexor fibularis longus: 0= lateral to the posterior part of the tibiotalar joint; 1= groove is plantad and central to the facet.

316. Shape of talofibular facet: 0= steep-sided; 1= steep-sided with a plantar lip; 2= sloped obliquely.

317. Length of the talar-tibial articulation: 0= dorsoventrally deep, extends to plantar aspect of talus; 1= dorsoventrally restricted, confined to dorsal part of talus.

318. Size of the posterior trochlear shelf of talus: 0= absent or weakly developed; 1= well-developed (prominent).

319. Talar neck length (neck length / talus length) expressed as a percentage: 0= short (≤ 44); 1= moderate (≥ 45, ≤ 56); 2= long (> 56).

320. Symmetry of the lateral versus medial talar trochlea: 0= trochlea symmetric; 1= lateral trochlear rim is raised relative to medial

trochlear rim.

321. Talar cotylar fossa: 0= shallow; 1= deep, medially projecting.

322. Width of talar head (Head width / Head height, expressed as a percentage): 0= (< 115); 1= (115-127); 2= (> 127).

323. Talar neck angle: 0= (< 20°); 1= (20-30°); 2= (> 30°).

324. Talar body height (lateral body height/midtrochlear width) expressed as a percentage: 0= (< 100); 1= (100-120); 2= (> 120).

325. Talar shape (Talar width/Talar length) expressed as a percentage: 0= (≤ 60); 1= (> 60).

*Calcaneal characters*

326. Anterior calcaneal elongation. Length of calcaneus distal to talo–calcaneal facet/total calcaneal length expressed as a percentage: 0= not elongate (< 40); 1= moderately elongate (40-45); 2= long (> 45).

327. Posterior calcaneal bowing: 0= absent; 1= present.

328. Presence and location of peroneal tubercle: 0= absent; 1= located at the far anterior end of bone; 2= in the anterior half; 3= centered; 4= in the posterior half.

329. Presence of a connection between anterior and medial sustentacular facets of the calcaneus: 0= facets separate; 1= sharply angled to one another or do not connect everywhere; 2= facets broadly confluent.

330. Angle of posterior talar facet (of calcaneus): 0= (< 3°); 1= (3-8°); 2= (> 8°, ≤ 24°); 3= (> 24°).

331. Length of posterior articular facet (of calcaneus). Ratio of posterior articular facet length to maximum length of cuboid articular surface (PASL/L): 0= (< 90); 1= (90-110); 2= (111-128); 3= (> 128).

332. Breadth of posterior articular facet (of calcaneus). Ratio of posterior articular facet width (PASW) to maximum length of the cuboid articular surface, expressed as a percentage: 0= (< 70); 1= (> 70).

333. The ratio of the length of anterior calcaneus to the maximum calcaneal length, expressed as a percentage: 0= (≤ 28); 1= (> 28, ≤ 33); 2= (> 33, ≤ 48); 3= (> 48, ≤ 60); 4= (> 60).

334. Ratio of the width to length of the posterior articular facet for talus, expressed as percentage: 0 = (< 42); 1= (42-76); 2= (> 76).

335. Length of posterior calcaneus relative to maximum length of cuboid articular surface expressed as a percentage: 0= (< 61); 1= (61-107); 2= (> 107).

*Navicular characters*

336. Navicular shape. Length relative to width of the navicular, expressed as a percentage: 0 = short (< 90); 1= moderate (100-150); 2= long (> 150).

337. Naviculocuboid articulation. The naviculocuboid articulation: 0= cuboid facet on navicular contacts only the ectocuneiform; 1= cuboid facet contacts the ectocuneiform and mesocuneiform facet.

*Entocuneiform characters*

338. Shape of entocuneiform/first metatarsal articulation: 0= dorsally reduced; 1= dorsal moiety of joint enlarged relative to ventral moiety; 2= dorsal moiety greatly enlarged.

339. Lateral process of the entocuneiform: 0= small; 1= hypertrophied.

General foot characters

340. Foot axis: 0= mesaxonic; 1= paraxonic; 2= ectaxonic.

341. Toilet claw (first phalanx, hind foot): 0= absent; 1= present.

342. External thumb: 0= present; 1= reduced or absent.

343. Prehallux: 0= present; 1= absent.

344. Metatarsus length: 0= short; 1= long.

*Metatarsal characters*

345. Peroneal tubercle of the first metatarsal: 0= very large; 1= large; 2= small.

346. Hallux length: 0= short; 1= long.

Other postcranial and miscellaneous characters

347. Claws (hand): 0= absent; 1= present.

348. Lumbar vertebrae count. Number of lumbar vertebrae: 0= (> 5); 1= (≤ 5).

349. Ratio of tail length to head and body length, expressed as a percentage: 0= short (TL:HB < 73); 1= moderate (73-116); 2= long (> 116).

350. Glabrous skin on tail. Friction pads on the tail: 0= absent; 1= present.

351. Baculum: 0= absent: 1= present.

352. Scent glands on genitilia: 0= present; 1= absent.

**Deciduous dentition**

*Lower deciduous tooth characters*

353. dp2-3 root numbers: 0= single; 1= double.

354. dp2 trigonid to talonid proportions: 0= dp2 trigonid >> talonid; 1= dp2 trigonid slightly longer than talonid; 2= dp2 trigonid and talonid of similar length.

355. dp3 trigonid to talonid proportions: 0= dp3 trigonid >> talonid; 1= dp3 trigonid slightly longer than talonid; 2= dp3 trigonid and talonid of similar length.

356. dp2 protoconid projection: 0= protoconid slender, projecting; 1= protoconid robust, projecting; 2= protoconid does not project above dp3-4.

357. dp2 metaconid: 0= metaconid close to protoconid; 1= metaconid widely spaced from protoconid.

358. dp3 metaconid: 0= metaconid close to protoconid; 1= metaconid widely spaced from protoconid.

359. dp2 metaconid: 0= absent; 1= trace or small; 2= large.

360. dp3 metaconid: 0= absent; 1= trace or small; 2= large.

361. dp2 trigonid: 0= closed lingually; 1= open lingually.

362. dp3 trigonid: 0= closed lingually; 1= open lingually.

363. dp2 entoconid: 0= absent; 1= present but cristiform; 2= present as discrete cusp.

364. dp3 entoconid: 0= absent; 1= present but cristiform; 2= present as discrete cusp.

365. dp2 lateral and medial protocristids: 0= confluent; 1= separate.

366. dp3 lateral and medial protocristids: 0= confluent; 1= separate.

367. dp2 metaconid position: 0= metaconid lingual or slightly distal to protoconid; 1= metaconid far distal to protoconid.

368. dp3 metaconid position (or orientation of postmetacristid): 0= metaconid lingual or slightly distal to protoconid; 1= metaconid far distal to protoconid.

369. dp2 hypoconid size: 0= large; 1= small; 2= absent.

370. dp3 hypoconid size: 0= large; 1= small; 2= absent.

371. dp3 hypoconid position: 0= hypoconid distal to protoconid; 1= intermediate; 2= hypoconid distal to metaconid.

372. dp3 hypocristid: 0= absent; 1= weak; 2= small.

373. dp2-3 buccal cingulum: 0= absent; 1= incomplete; 2= complete.

374. dp2 shape: 0= buccolingually compressed; 1= rounded oval; 2= buccolingually broad.

375. dp4 roots: 0= one root; 1= two roots.

376. dp4 cusp relief: 0= moderate to high relief; 1= low relief.

377. dp4 trigonid to talonid width: 0= wide (trigonid mesiodistal ≥ 1.1 talonid mesiodistal length); 1= widths similar (< 1.1, > 0.95); 2= narrow (≤ 0.95).

378. dp4 trigonid: 0= open lingually; 1= closed lingually.

379. dp4 metaconid position: 0= lingual or slightly distal to protoconid; 1= far distal to protoconid.

380. dp4 paraconid: 0= absent or cristiform; 1= small discrete cusp; 2= large cusp.

381. dp4 lateral protocristid: 0= runs towards metaconid; 1= runs toward hypoflexid; 2= absent.

382. dp4 posterior trigonid wall: 0= complete; 1= sulcus between lateral and medial protocristids.

383. dp4 facet X: 0= present; 1= absent.

384. dp4 entoconid: 0= absent; 1= cristiform; 2= small discrete cusp; 3= large.

385. dp4 postentoconid sulcus: 0= present; 1= absent.

386. dp4 hypoconulid: 0= large; 1= moderate; 2= trace or absent.

387. dp4 hypoconulid: 0= twinned to entoconid; 1= slightly lingual to midline; 2= in midline.

388. dp4 cristid obliqua: 0= absent; 1= rounded; 2= trenchant.

389. dp4 cristid obliqua orientation: 0= towards protoconid; 1= between protoconid and metaconid; 2= towards metaconid.

390. dp4 cristid obliqua terminus: 0= to base of trigonid; 1= partway up trigonid; 2= to protoconid or protocristid.

391. dp4 centroconid: 0= present; 1= absent.

392. dp4 hypocristid: 0= absent; 1= weak; 2= strong.

393. dp4 buccal cingulum: 0= absent; 1= partial, broken; 2= complete.

394. dp4 talonid: 0= open lingually; 1= closed lingually.

395. dp4 hypoflexid: 0= very shallow; 1= shallow; 2= deep.

396. dp4 distal fovea: 0= absent; 1= present.

397. dp4 hypocristid accessory cusp: 0= absent; 1= present.

398. dp4 cristid obliqua: 0= straight; 1= notched.

399. dp4 trigonid mesiodistal proportions: 0= elongate relative to talonid; 1= short relative to talonid.

Upper molars (renumbered and new)

400. (Marivaux et al 2016; Character 422). M1-2 premetacrista: 0= indistinct to absent; 1= weakly developed; 2= well-developed (but well-marked notch between premetacrista and postparacrista); 3= strongly elevated (weak notch between premetacrista and postparacrista).

401. (new character) M1-2 entoprotocrista: 0 = present; 1 = absent.

402. (Marivaux et al 2016; Character 424). M1-3 anterior cingulum: 0= complete (very long), reaches the parastyle; 1= long, stop at the level of the paraconule (or where a paraconule should occur); 2= short, does not reach the paraconule (or where a paraconule should occur); 3= very short, mesiolingually limited (not extended).

403. M1-2 mesostyle position: 0= attached to ectocrista; 1= present on buccal cingulum.

404. (Marivaux et al 2016; Character 421). M1-2 postparacrista: 0= indistinct to absent; 1= weakly developed; 2= well-developed (but well-marked notch between postparacrista and premetacrista); 3= strongly elevated (weak notch between postparacrista and premetacrista).

405. M1 postprotocrista length: 0= indistinct to absent; 1= short; 2= long.

406. M2 postprotocrista length: 0= indistinct to absent; 1= short; 2= long.

407. M1 postprotocrista direction: 0= transverse, buccally directed; 1= lateral, directed toward the lingual posterior cingulum (postprotocone fold-like).

408. M2 postprotocrista direction: 0= transverse, buccally directed; 1= lateral, directed toward lingual posterior cingulum (postprotocone fold-like).

409. M1 postprotocrista terminus: 0= runs to base of metacone (with hypometacrista); 1= runs to metaconule (at the level of the small or virtual metaconule); 2= runs to posterior cingulum; 3= limited at a point distal to protocone.

410. M2 postprotocrista terminus: 0= runs to base of metacone (with hypometacrista); 1= runs to metaconule (at the level of the small or virtual metaconule); 2= runs to posterior cingulum; 3= limited at a point distal to protocone.

411. (Marivaux et al 2016; Character 418). M1-3 posterior cingulum: 0= weakly developed; 1= moderate, does not reach the metastyle; 2= connected to metastyle.

412. (Marivaux et al., 2016; Character 419). M1-2 posterior cingulum lobe (distomedial) inflation: 0= no inflation; 1= slightly inflated; 2= strongly inflated.

413. (Marivaux et al., 2016; Character 420. M1-3 posterior margin (waisting between buccal and lingual cusps): 0= indistinct to absent; 1= present but shallow; 2= present, deep.

414. (Marivaux et al., 2016; Character 415). M1-2 lingual cingulum structure: 0= mesiodistally complete; 1= broken lingually (interrupted).

415. (Marivaux et al., 2016; Character 416). M1-2 metastyle: 0= indistinct to absent; 1= moderate; 2= strong.

416. (Marivaux et al., 2016; Character 417). M1-2 parastyle: 0= indistinct to absent; 1= moderate; 2= strong.
