# Supplementary material for: The Earliest Known Radiation of Pitheciine Primates
Source: Am J Primatol. 2025 May 16;87(5):e70040. doi: 10.1002/ajp.70040 (PMC12082270; doi:10.1002/ajp.70040)
Supplement: Supplementary file 3 — Appendix 3. Matrix of morphological characters. [file AJP-87-e70040-s004.pdf]

```
xread
416 55
Simonsius
      ?????200?1?1?120?1110201110011110111111100001?201?10011100121 0?1110?
11011010000001?11?3????0??????0?????1?0002100201 2?021011112111110010 01030
00?131220111000???00010211101111 02[0
1]00211100001111110112100?????????????1??122011?0000000100001000310022 00
020?010[0 1]01002[1
2]001110000???1?????????????????????????????????????????????????????????
???????????????????????????????????????????????????????????????????1?211
00????210011
Apidium     ?????00??1???1???101??????????1[1
2]??????1??011?201?101111?0?21021110????110?0??000101111001000000000001020
120002100202 2?000111100111110100110 1000?132[1
2]20111101???00020211101111010001 10[1 2]00000111[0
1]11212100???????0??????1??[1 2]221111000031000[0 1]0010002200322001[0
1]0?100020231002110201110[0 1][1 2]02111010100 10??211010010100[1
2]1?020211001[0 1]1010100210100202 1011[1
2]?????0????0????????????????????????????????2???????????????????2?1?200
0011210001
Cebuella   11010[1 2]0011012210?1010110[0
1]1200?111011121000110201111111001210 21110?01011001201001110111011021111
10101101000021000022?12001200101000101001020 11?2212?110?10????00??0211100
00111233??11100?11?200021501010010220000000 1100010[0
1]0000010020011000 0?000000022?0???2100100100?00??2?0?30223?[0
1]?0?020??0??200[1 2]21?[0 1]1[0 1]??2112202110020????2??120100[3 4][1
2][1 2][1
2]0111?10?2100?20102010000 0??00110000??11?0001121100011 22?20011012001111?
1122102220[0 1]?0[0 1]
Callithrix  [0 1]101000011012210?101011[0 1]01200?111[0
1]1112100011020111111110012102111 0?0101100120100111011101 2[0
1]211111010120[1 2]000021000022?12000200101010 1010[0 1]102111?23[0
1]3?110?00???00??022110000110233??11100?11?20001150[0 1]0100221100000[0
1]1100012000000100200110000?00000[0 1]022?0??02100102100?0[0 1]??1?0?[2
3]0[2 3]23?[0 1]?0?020??0??20[0 1][1 2]2[0 1]?[0 1]0[0
1]??211?201110020????2??[1 2]20100[2 3][1 2]2[1 2][0
1]11111012000120102010??????????????????????????????????????????????2111
112103220100[0 1]
Saguinus   [0 1]100100011012200010101 0011[1 2]00?111[0
1]1112100011020111101110012102111 0?0101100120000112001001210000000010 2120
00021000022?100012011[0 1]10101010[0 1]001011?13[0 1]2?[0 1]10?[0
1]0????00??012100000110[1 2][2 3][2 3]?011111?11?200110[4
5]1101002111000100011000001 00000[0 1]002100[0
1]0100?000000022?0???100010210 0?0[0 1]???2?[0 1]010[1 2]1[0 1]1[0
1]10102001 0??20[0 1][1 2]2[0 1]0[0 1]0[0 1]10211[1 2][0 1]0111002[0
1]????2??12[0 1]1003[1 2]2[1 2][0 1]11[0 1
2]?10?2100?201020100002?001110000?011?000102110001022?2021111000102 1??220
1133201001
Leontopithecus   00011000110122 10?101010[0
1]00200?1111111010001102011111111001210 21110?01011001211001020110011 20000
00001021200002100002 2?1210[0
1]201101010101001020 11?1304?110?10????00??02210000[0 1]1102[2 3][2
3]?010011?11?20001051[0 1]0100201100010001100111101000 0000210001100?[0
1]00000022?0???100010 3000?00??[0 1]?[0 1]?[1 2]0[2 3]23?[1
```

2]?0?020??0????112[0 1]?[0 1]?1??2122101110020?0??200????003[1 2]2[1 2][0
1]11[0
1]1101210012010201000 20?101110000?011?0001011100011 22?2021101000101 1[0
1]?122[0 1]022211000
Lagonimico ?????000?101???????1??0[1
2]????????????1????0???20???11111???????1111???0???????001112211001120010
000010012?00021000022?10001201100010101000010 11?1????0110201???00?0022100
000111222200001201112001114110100202200000001100111001000100210011101 2100
000022?0???2001103[0
1]00000000???
??112?2
12113321[0 1]001
Callimico 10000100110122 10?1010000[0
1]1100?111011121000110201111011100121021110?01?110112110010200100100 00000
0001020[1 2]00002100002 2?1010220110101010 10000[1
2]011?1302?0110100???00?002210000011023 33?211000120200011411010020 2200000
0011001020000000000210000100110001101200??0210210210000000000?[2 3]020[0
1]?1?0?020??0??201[0 1]2[0 1]?[0 1 2]1[0 1]??210?1011100[0 2][0
1]????200120100[2 3][1 2]2[1 2]1[0
1]11?10?2000?2010201000 22?102100000?011?00010210000121202021201100111 1011
01113321[0 1]000
Mohanamico
???
??????????1011????1001??1?100000100?2?01021000022?10101201100010101000020
11?1311?0110101???00??022100000111233??10022?11?100011?130???????????????
???
???
???
Pithecia 110110001101222011010[0 1]0121100?12[0
1]1111110002002011100111001210211 10?0101101122011112211000112120220000012
101021000022?221[0
1]220220000000110003011?1301?0110101???00?0120100001111 2332?0002221002000
003200100211100011011[0 1]001111210000[0 1]121000020123100[0
1]00002200?2002[1 2]02000010[0 1]0210?30[1 2]001???02???0??200[1 2][2
3]1?011??[0 1]11110111001 1????000120100322[1
2]0111?00?2000?200010110132010210110000000111111100001312?1021101000 1111?
?3111011210001
Cacajao
1111100011012221?101010121100?121011101100200201110011100121021110?
01?110112201111221100011212022000001320102100002 2?211022022000000001100030
11?1303?0110101???00?022010001111 0233[2
3]?00022210020000032001002211000110010 10??1221010011201000 20??310000000[2
3]200?2001100000000[0 1]021[0 1]?1[0 1]201?2?[0 1 2]?021??0????1[1 2]3[0
1]?[0 1]?[0 1]??[0 1 2]11?00211001[1 2]?????00?????0[3 4]12[1 2]011[0
1]?00?2000?2000001 0?????????????????????111100101212?1021101000 1131??311
1133200?22
Mazzonicebus
???
??????????0111????10011121201100000?3?000210000?2??2?0?20?20?010101001020
11??300?011?10?1??00??020111000?11233??10011?11?101100?2[0
1]01??10?????0001?100001?1000002121010110021100000111100?2[0 1]0[1
2]102[0 1]00?001?[0
1]0???????????????????????????????????????1??????????????????????????????
???????????????????????????????????11101200130112?21?112100011[2

3]12111133210011
Chiropotes 1?1??00011012220?101000[1 2]21100?1[1
2]1111101100200201111011100121021110?01?110112201112221100011212022000001
30010210000022?2211220210000000110003011?1303?0110101??00?022010001111023
3[2
3]?0002221002000003100100221100011001010??122101000020100020??310000001[2
3]200?2001100000000100121[0 1]?1120[0 1]?2?0?021??0??201[1 2][2 3][0 1]?[0
1]21??[0 1 2]11[1 2]00111000 1?????????????00[3 4][0 1 2]2[1
2]0111?00??000??0001011???31?
?311113320 0?12
Nuciruptor
???
??????????0111????10?1??2?202200?0013?00021000022?1?101211201000001011020
11?1301?011010????00??020100001111233??00022?11?200000?200???????????????
???
???[3
4]???
?????????????????
Proteropithecia
???
???????????????????00???2120??00000?3?0002100?0????2?0??????????????01???
??????????1?10????00??120110000111233??00022?11?200100?200?????????????1?
11???
???
???
Callicebus 0001011011112220?101000111200?121[0
1]1112110021020111101110012102111 0?01011011210111022010010110100100100010
00021000022?121[0 1][1 2]20220100010100002011?1[2
3]00?0110101??00?0021100000111[1 2][2 3][2 3][1 2]1111222110 1[0 1]0[0
1]1131001002111000210 11[0 1]101110110000[0 1]121000110012100[0 1][0
1]00022000200[0 1][0 1]020000001[0 1]0010[1 2][0 1]213111[0
1]102001000201[0 1]2[0 1]000[0 1]20[1 2]11?10211001[0 1]1110100221000[2
3]12[1
2]0111100120001200020110022011211000010100001102110001222220211010000 1111
?1001133200000
Homunculus 11011000[1
2]101?220?101000111200?111?111?1100?1?201?100111???2102111 0???[0
1]1101121010 1[0 1]2?110010?10100100100?1?00021000022?121[0 1][1
2]201201000101001020?1?1300?0110100???00?10211[0 1][0 1]000111[1 2][2
3][2 3]?011011?1[1 2]?2[0
1]0011320010 0?1?10?0??0111101110000000122101001 0[0 1]12100[0 1][0 1]000[0
1]10002002202000000000?00202011110100?01?????1121?0?1??2??2??????????????
??102012011312?2??21?
012001011 1?211113321 0011
Cebus 1000[0 1]00011012200?1010[1 2]01[1 2]1000?121010121[1
2]0020120111001110012102111 0?010110012210011210100121000000001011210002 10
00022?11112201[1 2]000001010010 2011?1303?0110[0
1]00???00?0022100000111 2332?00022211020111042101002200000200110211122010 0
001[0 1]2110001022210010000121 0?2001[1 2]0[1 2 3]0000111120[0 1]?1021[0
1]12?0?020010??1[0 1][0 1][1 2][2 3][0 1 2]?[0 1]0[0 1]??[0 1 2]11[1 2][1
2]01110011????200120100[3 4][0 1 2][2 3][1 2][0
1]1111001?0001?000201000220112101200101000001111000013011202110100011 21??
2100?33210100
Aotus 10010[0 1][0 1]111012200?101010111100?1[1

2]11111211022102011110111001210211110?0101101111011102201001211010000010 01
100002100002 2?121022012010001010000 2011?1[1
2]01?0110101???00?1021100000111 2333?1112221102[0
1]000031001002221000200[0 1]1[0 1]10111[0
1]21000000210000101121000000002200?200[1 2]10[0 1]0000010020[0 1]01[0 1][1
2 3]101[0 1]1[0 1 2]10200100020[0 1][0 1 2]2[0 1]0[0 1]1[0 1]20[0 1
2]11?10211001[1 2]1110100221000[2 3][1 2]2[1 2][0
1]1111001200012000201100221102100000 1011?0011021100013 22?202110110011 11??
2220033210000
Aotus_dindensis
      ???????1?????????????????????????????????2?????????????????????????
?????????0111????1001111000000010011?00021000022?10101201201000101000020
11?1311?0110101???00?102110?0001112333?100222111100011???0?????????????11
1101?1?????????????????11??
???
???
Tremacebus
      ??0??0??1111?200?10?01001?200?1210??12100111?201?11?11100121??111??
010???1121????????????????????????????????10?????????????????????????????
????????0??
??011??????????????????122100???001?0??2?011?2????0??020?????????????????
???
??3??1111332100??
Alouatta 10000[1 2]00[1 2]1212[0 1]20110100022 1200?111[0
1]0100100021020111001110011102111 0?010110112001112221101100000000000101012
0002100002 2?1210220[1 2]201000101000000 11?13[0 1][1 2]?0111[1 2]0[1
2]???0000022[0 1]10000111[1 2][2 3][2 3][1 2]0211112121[1 2]0002213[0
1][0 1]10000000001000 1101[1 2][0 1 2]00300000[0 1 2][0 1]2100001[0
1]02320000000 1100?120[0 1]10[0 1]1000011121[0 1]?102131[0
1]?02020??0????11[2 3][0 1]?[0 1]?[0 1]??[0 1]22200011001 1???000120100[3
4]2[1 2][2 3]0[0 1]1[1
2]?00?2000?20011111012?102100000?011?0011021100012021210120120011 21?0211
1133210?21
Brachyteles 11011200[1 2]1212[1
2]20?101000211200?111101011100200201110011100121021110?01110112?01112221
1001000000000010201200021000022?121022112111001000000000 11?1[2
3]01?001?101???000002101000011 22332?2110021220000123310100000000 10?1001?00
110[0 1]300000002010002 1??3100000001 0?1?21000000000000 11210?[1
2]022310?22021??1????[0 1][1 2][2 3]0?[0 1]?0??122??0011000[1
2]????????????042220[0 1][0
1]1?00?2010?2001210100 12?002110100?011000110101000132202121201200 1121?021
1113320 0?0[1 2]
Stirtonia
      ???
?????????????1????1???????0???00?0?0?????????????????????????0???????????
????????011?2?????00??02?1100001112[2 3][2
3]?020012?12?200011?301?????????????01001[1 2][1 2]1010000011201000[0
1]1013200000001101?[1 2]20011120000111[1
2]2??
??2130212113321
0121
Ateles 110[0 1]11001111212001010001 11100?1[1
2]1101011100200201110011100121021110?01[0 1]11011220[0 1]1122[1
2]11001200000000010122100021000022?12102201101000[0 1]0100102011?130[1

2]?0000101???00?0020100000111[1 2]2[2
3]2?10012211120101132001001211010110011100011300000002100001022 3[1
2]0000000210 1?20001000000001120[0 1]?10[1 2 3]231[0 1]1[0 1
2]2021101????[0 1][1 2][2 3][0 1]?[0 1]?[0 1]??[0 1 2]1220001 1000[0
1]?01010022?100[3 4]2[1 2 3][1 2 3]011[0
1]100120101200121010032?002110100?0100101111100021112110211110001121??211
1133200?00
Lagothrix 110[0 1]020011112110?101000211[1 2]00?121[0
1]01011100210201110011100121021110?0111101122011122[1
2]110012100000000102222000210000 22?1210220120100010100001011?1301?0110101
???00?0020100000111 2332?1000221112000113200100121101011001 1?0002120000000
2100002 0?23[1 2]00000002[0 1]00?2000100000000 11210?[2 3]02231[1
2]?02021???????1[1 2]3[0 1]?[0 1]?1??[0 1 2]22200111001 1????0??2201?04[0
1][1 2 7][1
2]01111001200012001210100220012110000101100011111000013222202120111011 31?
?3011133200?00
Aegyptopithecus
      0?00?0002101?020?0011101211 00?120211?0100021?201?10011101121021110?
0101100120100102111001000010000010101200021 1?2022?1?1?0200101000101 0?1030
11?230120111101???010102110010[0 1]0010000100000111111 1[1 2]1[0
1]1300?????????????031102201?100[0 1]121210001100012001110010?000[0 1]0[1
2]10210001100021010[0 1]0011101010010???0?[1
2]2?0??0?????2?????????111011112110030131?12??0?20????0??????1?2??0?2?1?2
?0?1?1120?10211100130012101221210001?111110011110001
Catopithecus   ?????000?1???1???101110121????1[1
2]1?????11?011?201?1???11001210???10?01011?11??000002?01?0??00010000010??
12000211?201121?1?02[0
1]020100010 11?002011012312011110 11??0001001001000111111010000012220 0[0
1]1[0 1]3010100001100020003100 12?1??000?002000101100220001011 00?000[0
1]0[1 2]1022000101011101[0
1]0001110101??????1210?00?000????2?????????11?0111211???????????????????
???11211220011211011
Proteopithecus
      ?????000?1?1?2?0?10?010121100?12121???11?011?201?1?0111001210?1110?
010?1?11??100002101??????????????????2???21002221210100210101000100121021
010111010111100 1??000[0 1]01200000011111[1 2][1 2 3]00[0
1]01101222001113010???1???0?????0111012020?000000201110010111000001100?0?
001110220000000[1
2]011011121110101?0????221010???10????11??1100??101010?1201??????????????
??10[2
3]?1220011221011
Branisella
      ?????????????1???
??????????0001????1001?[1
2]?0?0100010 2?1100021000022?121012011010001011000 3001?1313?0111101???000[
0
1]021100000111111110111101122000113100?????????????0?110100010000?0020110
100011100[0 1]001110?0?[1 2][0 1]0110200000000[1
2]0??
??11[2
3]11111111211011
Canaanimico
      ???
???

???
???????????????????????0???000?0111[0 1]0??200121[2
3]000?00??1??
???213?
2111133200011
Soriacebus
???
??????????0111????1001112120110000013?00021000022?11101201201010101001020
11?1312?0110111???00?1020110000111122101001111112010003120?????????????1?
1022[1 2]100000002221010110021100000111100?2[0 1]01102000000[0 1]0[0 1
2]1??
??11[2
3]12111133200011
Hylobates
100000002101212 0?1010?0121000002111112100021020111001110112102 1111?
01011000221001221110011100000000 10[1
2]12000211?2222???1?0201101000??10?0030111242110111101???000102010011000
100001000000011111101133?0100????0?????0312?22?1?0000?0021?00?200?22000000
020?0?0002[0
1]0200?00?22222?3112311112??110?????1130?0?1??0223001110001?????112201?03
1301121?????0?????0???????????????1?1???1?1010?101100110200210011010100121
??3220000220000
Miopithecus
11111000210121 10?1010001110020021111121100200201110011101121021111?
0101100022100122111001221000000010?212000211?2222???1?0201101000??10?0030
111242010111101???00?00201001000022332?200000122000 01133?0100????0?????03
10?22?0?0000?00210000200?220000000320??000220000?01012222????2?022?????11
???????????????11?200011002??10011101010030??1121?????0?????0????????????
??21??311113210?00
Presbytis
11111000210120 10?1010001210010021111121000210201110011101121021111?
0101100022100122111001111000000010021200021 1?2222???1?0201101000??10?0030
111231110111101???000102010010000223 30?200000122000011 23?0100????0?????03
10?22?0??000?00210000200?220000000220???0???0000?00022222?20223022????011
1????1220?1?1??111100?110020?????110201?030211210?11100????0101??????????
??11??2??????200?00
Acrecebus
???
???
???
???????????????????????????001?0?0221??200120 2000?21??1??????????????????
???
??1?2?1?0?1?3220000
Cebupithecia
??0??????????1?0?10??102??????????????1??0????????0011100121021110?
??011?1???0111????10?1??21202?00?0003?21021000022?2?1?12012010000010?1020
11?1300?0110?0????00??120100?0?1?1?3???00?2??11?20100?4100??0??????????1?
11011101100000021000020?2200000000220??200010 2100?00??0?????????????????
???????????????21??10?1100?1?0??100021?0?3????111?????????????2??????????
??21[2 3]?2121133210001
Chilecebus ??????????2???0??1?1???21??????????????1?0?[1
2]?2??????????????????10??????????0????????????????????????????????1?????
???
????????????000220??2?0110201101210001 11?10101100?2100010001 0?002002202[0

1]000010001???
???1?1?1
00??33220010
Antillothrix
1101100011212120?101020211200001120111100021?201011?11101121021110?
010110112?0101????1?????????0??????02??????10000??????????????????????????
????????011010????????021110000111133??011[1 2][1
2]??1?201022320110?202000000011020[0
1]12011000100210001101221000000100?0?20001[0 1]30000010[0
1]11?11000112102020 21???10022000000 11????00110011?????????????????????????
??112?20111332
10011
Paralouatta
?00??1001??12021?101?2021?1?0?02?10?1?1000?1?2???11?111001?????110?
010???????01112221100102000000000000000?0021100002?20200110110101010000 1000
12?1313?0111111?????000201100001111332?11122212221002243211 0?2021000200?1
??[0 1][1 2][1 2]?211000100210001110[1 2]1100000000100?210002220000111[0
1]21010111121111201????????20?????0???3001110011??????0???????????????????
?00??210???2?[2
3]12111133220021
Xenothrix
??0????0?111?1?1?1010???1?????02?20??????0?1????????????????????????
??????????010112111??1??????0?0????????????1000022?0?1?22???001?01010?1000
0??1?00?111?01?????2??020100011111000?200122?11?2110115200100[1
2]??????????1??011[0
1]0?1000?00210000200?20000001120??1001?00000021??2010100011[1
2]1111200????????3???0000???
???31??30?1?3?210?21
Insulacebus
???????????????????1??
??????????0??1????1?01??0?0000?0?000??00??1000022?021022111000?0101001010
01?1201?0???110???0000021110011111133 1?1112221102000223201???20200000001?
02001001100010021000110??21000000000?0?20001030000010121??????????????????
??
???213?2001133210011
Perupithecus
??
??
??
???????????????????????0?0?000002??0???0002203000?0???1???????????????????
??
???102?12?1?1?201011
Miocallicebus
??
??
??
???????????????????????012?00000?0210??20??102000?00??0???????????????????
??
???1???1?0?1?3200000
Talahpithecus
??
??
????????????1?????????0?0???0?0??11?1?00?0???11?0000?????0????????????????
??????0?0?00???2??1?001??0?000002??0????002213000?0???1???????????????????

???
??102?12?1?1?201011
Parvimico
???
???
???
?????????????????????????1?000001?00????102002100?0???1??????????????????
???
??112121?1?3?2?0011
Killikaike
110??000?1012??0?1010???2???????11?1???20010?201?1?????????????????
?????????2????????????????????????????????10?????????????????????????????
????????0???0??????????0?
01?1?1?000??0???10100?0022100000001100?20022030000011011?????????????????
???
??111?1111133210011
Saimiri   11111000110122000101 0[1
2]0021000?1111111212102012011110111001210211 10?0101100122100112111001[1
2][0 1]1000000010112200021000022?1210220220100010100001011?1303?0110[0
1]00???00?00221100001112[2 3][2 3]20111001120200111411010022110002001 1[0
1]2[0 1][0 1]11[1 2]010000002100011012110000000 1100?200[0 2][1 2]0[2
3]00000111[1 2]010202001210202001 0001[0 1]1[0 1]210[0
1]0120211220211002011102001 10000[3 4]12[1 2
3]01111001200012000201100121112101200101000121021100013011212121120011112
?111113321000[0 1]
Dolichocebus
??0?00?0?1?12200?10?02002??00??21???121?1010?2?1??1?111001210 21110?
0101101122?0??????1?0?0?0000??001010120002100002 2?12101201201010101001020
?1?1302?011010???00??021110000111122?011012?11?1[0
1]11103110100??1?0001000?10011?00100002220110110012100000001[0 1]0002[0
1]01[0
1]020000011111???110100????????
???11
211111133210011
Laventiana
???
??????????1001????10?1??????1?0?????1?01021000022?12101201201010101000020
11?1302?011010???00??021110000111233??11112?11?210111?100????????????????
???
???
???
Panamacebus
???
????????????????????????1000?001102?1?0002?0000?2?1210?20?10?010101000010
11???1???
???????????????????????0?200000000110??200[1
2]103000?01??0???
??1
12?1111133200000
Neosaimiri
???
??????????10011???10010110000000102?12000210000 22?1210220220101010100002[
0 1]11?1301?0110100???00?00221100001112[2 3][2 3]?01101221212[0
1]011141201??20[0 1]10002001?[0 1]200120010000[0 1]02101011001 1100000[0

1][0 1]10?0?2[0 1]1[0 1][0
1]030000010010102010012102????????????????????????201110020???020????????
???11
111221133210011
Ucayalipithecus
      ???
???
????????0???1?0???00000?2?????????0?0010?0?001001010?13??0???????????????
???????????????????????????0032?0010??10?????????????????????????????????
???
???00???????0??
Qatrania
      ???
??10020?2?0?0??11?01?1?11100?1000
?????????1111?011?00000?2?????????000011100001001112113020???????????????
???????????????????????????0032?0010??10??????????????0??????????????????
???
???0????????0??
Carlocebus
      ???
??????????0?01????1?01????????0?????1?0???100002[1
2]11210120120101010?00102011?1?00?0110100???00?002111?000111[1 2][2 3][2
3]?011111112?2[0 1]0[0 1]114200?????????????1?111[1 2]1?0?0000?[1 2][1
2]21010110[0 1]221000[0 1]0001[0 1 2]00?2[0
1]021020000000100??
???
?111120011332100[0 1]1
;

cnames
{0 Ethmofrontal_sinus;
{1 Splenofrontal_sinus;
{2 Maxillary_sinus;
{3 Anterior_ethmiodal_sinus;
{4 Sphenoidal_sinus;
{5 Zygomatic-facial_foramen;
{6 Zygomatic_arch_position;
{7 Extent_of_inferior_orbital_fissure;
{8 Zygomatic-parietal_contact_at_pterion;
{9 Position_of_lacrimal_foramen;
{10 Extraorbital_exposure_of_the_lacrimal;
{11 Zygomatic-lacrimal_contact;
{12 Contact_between_lacrimal_and_palatine_bones;
{13
Position_of_the_infraorbital_foramen_relative_to_the_Frankfurt_horizontal
_plane;
{14 Angle_of_cranial_kyphosis_;
{15 Nasal_fossa_width;
{16 Nasal_capsule_;
{17 Snout_length;
{18 Maxila_depth;
{19 Inter-incisor_diastema_width_;
{20 Ascending_wing_of_premaxilla_;
{21 Postglenoid_foramen;

{22 Temporomandibular_joint_morphology;
{23 Postglenoid_process_size;
{24 Palate_shape;
{25 Interpterygoid_fossa;
{26 Length_of_medial_pterygoid_plate;
{27 Encroachment_of_the_auditory_bulla_on_the_pterygoid_fossa;
{28
Nature_of_contact_between_the_lateral_pteryfoid_plate_and_the_bulla_wall;
{29
Extent_of_contac_between_the_lateral_pterygoid_plate_and_the_bulla_wall;
{30 Pyramidal_process_of_the_palate_and_post-alveolar_notch;
{31 Mediolateral_position_of_pyramidal_processes_(;
{32 Posterior_palatine_torus;
{33 Posterior_nasal_spine;
{34 Posterior_extent_of_the_turbinates;
{35 Angle_of_the_incisive_canal_in_palate;
{36 Temporal_emissary_foramen;
{37 Paroccipital_processes;
{38 Pneumatization_of_mastoid_;
{39 Lateral_cranial_profile_at_glabella;
{40 Interorbital_fenestra;
{41 Size_of_orbits;
{42 Orbital_convergence;
{43 Interorbital_breadth;
{44 Exposure_of_vomer_in_orbit;
{45 Postorbital_closure;
{46 Composition_of_the_postorbital_septum;
{47 Position_of_interorbital_constriction_relative_to_olfactory_tract;
{48 Foramen_rotundum_;
{49 Metopic_suture_in_adults;
{50 Cochlear_housing_as_exposed_in_middle_ear;
{51 Transbullar_septa;
{52 Transverse_septum_arising_from_the_cochlear_housing;
{53 Extent_of_pneumatization_of_anterior_accessory_cavity;
{54
Presence_or_absence_of_perbullar_pathway_for_the_internal_carotid_artery;
{55 Anteroposterior_location_of_posterior_carotid_foramen_in_bulla;
{56 Mediolateral_position_of_posterior_carotid_foramen_in_bulla;
{57 Ventrodorsal_position_of_the_carotid_foramen_in_the_bulla;
{58 Position_of_posterior_carotid_foramen_relative_to_fenestra_cochleae;
{59
Position_of_the_internal_carotid_canal_relative_to_the_fenestra_cochleae;
{60 Position_of_the_portion_of_the_internal_carotid_/_promontory_artery_;
{61 Size_of_stapedial_and_promontory_canals_;
{62 Morphology_of_promontory_canal;
{63 Canal_for_internal_carotid_artery_or_nerves_;
{64 Position_of_ventral_edge_of_the_tympanic_bone;
{65 The_shape_of_the_tympanic_bone;
{66 Morphology_of_annular_bridge_;
{67 Flange_of_basioccipital;
{68 Basioccipital_stem;
{69 Suprameatal_foramen;
{70 Patent_parotic_fissure;
{71 Enclosure_of_intratympanic_portion_of_facial_nerve_in_a_bony_canal;

```
{72 Epitympanic_crest;
{73 Tentorium_cerebelli_ossification;
{74 Vascular_canal_connecting_sigmoid_sinus_with_subarcuate_fossa;
{75 Size_of_olfactory_bulbs:;
{76 Relative_brain_size:;
{77 Symphyseal_orientation;
{78 lateral_profile_of_manible;
{79 Mandibular_corpus_depth_;
{80 Symphyseal_fusion_in_young_adult;
{81 Coronoid_height_relative_to_condyle;
{82 Condyle_height_relative_to_tooth_row;
{83 Angle_of_the_mandible;
{84 Depth_of_the_coronoy-condylar_notch;
{85 Lower_incisor_number;
{86 Lower_incisor_occlusal_arrangement;
{87 Lower_incisor_spacing;
{88 _i2-c_diastema;
{89 _i1-2_size_;
{90 i1:i2_proportions;
{91 i1_scrown_width;
{92 i2_crown_cross-sectional_shape;
{93 Lower_incisor_crown_height;
{94 i1-2_crown_buccal_outline;
{95 Lower_incisor_roots_;
{96 Lower_incisor_crowns_;
{97 Tooth_comb_;
{98 i1_crown_shape_;
{99 i2_heel_development;
{100 Incisor_lingual_enamel;
{101 Lower_incisor_lingual_cingulum;
{102 i1_area_to_m1_area;
{103 Female_c1_size_;
{104 c1_dimorphism_;
{105 c1_cross_sectional_shape;
{106 c1_lingual_crest_developmentc1_lingual_crest_development;
{107 Canine_paracristid_;
{108 Canine_height_;
{109 p1/P1;
{110 p2/P2;
{111 p2_roots;
{112 p3-4_roots;
{113 Premolar_crowding;
{114 p3_paraconid;
{115 p4_paraconid;
{116 p4_paraconid_position;
{117 p3-4_cristid_oblicua;
{118 p2_protoconid_height_and_shape;
{119 p4_metaconid_position;
{120 p2_metaconid_size;
{121 p3_metaconid_size;
{122 p4_metaconid_size;
{123 p4_trigonid_-_lingual_wall;
{124 p3_entoconid_and_lingual_talonid_crest;
{125 p4_entoconid_and_lingual_talonid_crest;
```

```
{126 p4_lateral_and_medial_protocristid;
{127 p3_lateral_protocrist_orientation;
{128 p4_lateral_protocristid;
{129 p4_lateral_protocrist_orientation;
{130 p3-4_posterior_trigonid_wall;
{131 p3-4_hipoconid_size;
{132 p3-4_hypoconid_position;
{133 p4_talonid_breadth;
{134 p4_hypocristid_shearing_development;
{135 p2_buccal_cingulum_development;
{136 Lower_premolar_inflation;
{137 p4_exodaenodonty;
{138 p4_talonid_length_;
{139 p4_anterobuccal_cingulum_development;
{140 p4_postprotoconmid_ridge;
{141 p4_pstmetaconid_ridge;
{142 p4_paraconid_height;
{143 p3?4_protoconid_height;
{144 Ratio_of_p3_to_p4_area;
{145 p4_mesiodistal_Length_/_buccolingual_Width;
{146 p4:m1_area;
{147 p3-4_orientacion;
{148 m3/M3;
{149 m1_root_number;
{150 m2_root_number;
{151 m3_root_number;
{152 m2_trigonid_width_;
{153 m1_trigonid_length;
{154 m3_trigonid_width_(based_on_relative_buccolingual_breadths)_;
{155 m1_paraconid_position;
{156 m2_paraconid_position;
{157 m3_paraconid_position;
{158 m1_parastylid;
{159 Molar_metastylid;
{160 m3_hypoconulid;
{161 m3_heel;
{162 Molar_occlusal_enamel_surface;
{163 m1_trigonid_height_;
{164 m1-2_cusp_relief;
{165 m1_trigonid_lingual_configuration;
{166 m1_metaconid_position;
{167 m1?2_paraconid_development;
{168 m1?2_lateral_protocristid_orientation;
{169 m1_distal_trigonid_wall;
{170 m2_distal_trigonid_wall;
{171 m1_wear_facet;
{172 m2_wear_facet;
{173 m1-2_entoconid;
{174 m1?2_postentoconid_sulcus;
{175 m1_hypoconulid_size;
{176 m2_hypoconulid_size;
{177 m3_hypoconulid_size;
{178 m1-2_hypoconulid_position;
{179 m1-2_cristid_obliqua_development;
```

{180 m1_cristid_obliqua_orientation;
{181 m2_cristid_obli_orien;
{182 m1_cristid_obli_termin;
{183 m2_cristid_obli_termin;
{184 m3_cristid_oblicua_terminus;
{185 m1-2_centroconid_development;
{186 m1?2_hypocristid_development;
{187 m3_hypocristid_development;
{188 m1-2_talonid_lingual_configuration;
{189 m1-2_distal_fovea;
{190 Molar_cusp_inflation;
{191 m1-2_cing_bucal_development;
{192 m1_hypoflexid_depth;
{193 m2_hypoflexid_depth;
{194 Ratio_m2_length_to_m3_length;
{195 m1_length;
{196 m1L/W;
{197 m1-2_entocnid_position_relative_to_hypoconid;
{198 _I1-I2_interstitial_contact;
{199 I1-I1_interstitial_contact;
{200 I2?C1_diastema;
{201 Ratio_of_I1_area_to_I2_area;
{202 I1_size;
{203 I1_occlusal_shape;
{204 I2_occlusal_shape;
{205 I1_crown_shape;
{206 I1_lingual_fovea;
{207 I1_occlusal_edge_orientation;
{208 I1-2_lingual_cingulum;
{209 I1_basal_lingual_cusp;
{210 I1and_I2_buccal_cingulum;
{211
C1_cross?sectional_shape_(ratio_of_maximum_length_in_the_occlusal_plane_to_maximum_breadth_in_the_occlusal_plane_at_right_angles_to_maximum_length)_;
{212 Upper_canine_occlusion;
{213 C1_mesial_groove;
{214 C1_lingual_cingulum;
{215 P2_root_number;
{216 P3_root_number;
{217 P4_root_number;
{218 Ratio_of_P2_area_to_P3_area;
{219 P4:M1_area;
{220 Occlusal_outline_of_P2;
{221 P3-P4_trigonid/talonid_proportion;
{222 P3_protocone;
{223 P4_metacone;
{224 P4_protocone;
{225 P2_protocone;
{226 Premolar_Hypocone;
{227 P4_Hypocone;
{228 P4_paraconule;
{229 P3-4_Parastyles;
{230 P3-4_metastyles;

```
{231 P3-4_Postprotocrista;
{232 P2-3_profile_of_distal_crwon_margin;
{233 P3-4_lingual_cingulum;
{234 P4_occlusal_outline;
{235 P3-4_buccal_cingulum;
{236 M1-2_root;
{237 M3_root_count;
{238 M2_shape;
{239 Ratio_of_M1_area_to_M2_area;
{240 M1-2_Nannopithex_fold;
{241 M1-2_pseudohypocone;
{242 M1-2_metaconule_;
{243 M1-2_paraconule;
{244 M1-2_preprotoconulo;
{245 M1_hypocone_size;
{246 M2_Hypocone_size;
{247 M1-2_Hypocone_position;
{248 M1-2_Prehypocrista;
{249 M1-2_prehypocrista_orientation;
{250 M3_prehypocristid_development;
{251 M1_or_2_paraconule_position;
{252 Hypometaconulecrista;
{253 M1_-2_mesostyle_size;
{254 M1_2_postprotocristia_development_(404);
{255 M1-2_hypoparacrista;
{256 M1-2_hypometacrista;
{257 P4-M1-2_pericone;
{258 M1-2_cing_lingual_development;
{259 M1-2_buccal_cingulum_development;
{260 M1-2_premetaconule_crista;
{261 M1-2_postmetaconule_crista;
{262 M3_paraconule;
{263 Molar_protocone_lingual_inflation;
{264 M2_buccal_expansion_of_paracone;
{265 M3_metacone;
{266 M3_Hypocone;
{267 M1-3_anterior_cingulum;
{268 M1_size_relative_to_M3_(based_on_the_ratio_of_areas_of_each_tooth;
{269 Shape_of_humeral_trochlea;
{270 Relative_heights_of_medial_and_lateral_edges_of_humeral_trochlea;
{271 Trochleocapitular_ridge;
{272 Waisting_of_the_trochlea;
{273 Width_of_capitulum_relative_to_trochlea;
{274 Entepicondylar_foramen;
{275 Entepicondylar_foramen_position;
{276 Medial_epicondyle_size;
{277 Dorsal_position_of_medial_epicondyle;
{278 Shape_of_dorsal_trochlea;
{279 Dorso?epitrochlear_fossa;
{280 Olecranon_fossa_shape;
{281 Supinator_crest;
{282 Brachialis_flange;
{283 Bicipital_groove;
{284 Deltopectoral_crest;
```

{285 Deltotriceps_crest;
{286 Medial_torsion_of_humeral_head_Rotation;
{287 Os_Centrale;
{288 Ulnar?pisiform_articulation;
{289
Length_of_femoral_neck_(neck_length_measurement_number_2/BSTD_expressed_a
s_a_percentage;
{290 Angle_of_femoral_neck;
{291 Angle_of_lesser_trochanter_LTA;
{292
Size_of_third_trochanter:_0=_large_(third_trochanter_projection_index:_>_
25);
{293
Knee_shape_(anteroposterior_diameter_of_distalfemur_/_mediolateral_diamet
er_of_distal_femur,_expressed_as_a_percentage;
{294 Femoral_head_shape;
{295 Anterior_extension_of_greater_trochanter;
{296 Anterior_bowing_of_proximal_femur;
{297
Relative_length_of_trochanteric_fossa_(intertrochanteric_fossa_length/BSD
LT),_expressed_as_a_percentage;
{298 Intertrochanteric_crest;
{299 Size_of_lesser_trochanter;
{300 Lateral_border_of_distal_femur_;
{301 Crista_paratrochanterica_on_posterior_femoral_neck;
{302 Projection_of_the_femoral_head_relative_to_the_greater_trochanter;
{303
Sum_of_lengths_of_humerus_and_radius_divided_by_summed_lengths_of_femur_p
lus_tibia_expressed_as_a_percentage;
{304 Ratio_of_humerus_length_to_femur_length_expressed_as_a_percentage;
{305 Contact_between_distal_tibia_and_fibula;
{306 Distal_tibia_articulation_shape;
{307 Shape_of_distal_tibial_articular_for_talus,_if_?square?;
{308 Medial_malleolus_rotation;
{309 Medial_malleolar_articulation;
{310 Shape_distal_tibia_shaft;
{311 Tibialis_posterior_groove;
{312 Posterior_border_of_the_trochlear_facet_for_talus;
{313
Medial_malleolar_height_of_the_tibia_relative_to_the_anteroposterior_diam
eter_of_distal_tibial_shaft,_expressed_as_a_percentage;
{314 Position_of_the_groove_for_the_tendon_of_m._flexor_fibularis_longus;
{315 Shape_of_talofibular_facet_;
{316 Length_of_the_talar?tibial_articulation;
{317 Size_of_the_posterior_trochlear_shelf_of_talus;
{318
Talar_neck_length_(neck_length_/_talus_length)_expressed_as_a_percentage;
{319 Symmetry_of_the_lateral_versus_medial_talar_trochlea;
{320 Talar_cotylar_fossa;
{321
Width_of_talar_head_(Head_width_/_Head_height,_expressed_as_a_percentage)
_;
{322 Talar_neck_angle;
{323

Talar_body_height_(lateral_body_height/midtrochlear_width)_expressed_as_a_percentage;
{324 Talar_shape_(Talar_width/Talar_length)_expressed_as_a_percentage;
{325 Anterior_calcaneal_elongation._Length_of_calcaneus_distal_to_talo?calcaneal_facet/total_calcaneal_length_expressed_as_a_percentage;
{326 Posterior_calcaneal_bowing;
{327 Presence_and_location_of_peroneal_tubercle;
{328 Presence_of_a_connection_between_anterior_and_medial_sustentacular_facets_of_the_calcaneus;
{329 Angle_of_posterior_talar_facet_(of_calcaneus)_;
{330 Length_of_posterior_articular_facet_(of_calcaneus;
{331 Breadth_of_posterior_articular_facet_(of_calcaneus)._Ratio_of_posterior_articular_facet_width_(PASW)_to_maximum_length_of_the_cuboid_articular_surface,_expressed_as_a_percentage;
{332 The_ratio_of_the_length_of_anterior_calcaneus_to_the_maximum_calcaneal_length,_expressed_as_a_percentage;
{333 Ratio_of_the_width_to_length_of_the_posterior_articular_facet_for_talus,_expressed_as_percentage;
{334 Length_of_posterior_calcaneus_relative_to_maximum_length_of_cuboid_articular_surface_expressed_as_a_percentage;
{335 Character_336_;
{336 _Naviculocuboid_articulation._The_naviculocuboid_articulation:_0=_cuboid_facet_on_navicular_contacts_only_the_ectocuneiform;
{337 Shape_of_entocuneiform/first_metatarsal_articulation;
{338 Lateral_process_of_the_entocuneiform;
{339 Foot_axis;
{340 Toilet_claw_(first_phalanx,_hind_foot);
{341 External_thumb;
{342 Prehallux;
{343 Metatarsus_length;
{344 Peroneal_tubercle_of_the_first_metatarsal;
{345 Hallux_length;
{346 Claws_(hand)_;
{347 Lumbar_vertebrae_count._Number_of_lumbar_vertebrae;
{348 Ratio_of_tail_length_to_head_and_body_length,_expressed_as_a_percentage;
{349 Glabrous_skin_on_tail._Friction_pads_on_the_tail;
{350 Baculum;
{351 Scent_glands_on_genitilia;
{352 dp2?3_root_numbers;
{353 dp2_trigonid_to_talonid_proportions;
{354 dp3_trigonid_to_talonid_proportions;
{355 dp2_protoconid_projection;
{356 dp2_metaconid;
{357 dp3_metaconid;
{358 dp2_metaconid;
{359 dp3_metaconid;

{360 dp2_trigonid;
{361 dp3_trigonid;
{362 ._dp2_entoconid;
{363 dp3_entoconid;
{364 dp2_lateral_and_medial_protocristids;
{365 dp3_lateral_and_medial_protocristids;
{366 dp2_metaconid_position;
{367 dp3_metaconid_position_(or_orientation_of_postmetacristid)_;
{368 dp2_hypoconid_size;
{369 dp3_hypoconid_size;
{370 dp3_hypoconid_position;
{371 dp3_hypocristid;
{372 dp2?3_buccal_cingulum;
{373 dp2_shape:_0=_buccolingually_compressed;
{374 dp4_roots;
{375 dp4_cusp_relief;
{376
dp4_trigonid_to_talonid_width:_0=_wide_(trigonid_mesiodistal_?_1.1_talonid_mesiodistal_length);
{377 dp4_trigonid;
{378 dp4_metaconid_position;
{379 _dp4_paraconid;
{380 dp4_lateral_protocristid;
{381 dp4_posterior_trigonid_wall;
{382 dp4_facet_X;
{383 dp4_entoconid;
{384 dp4_postentoconid_sulcus;
{385 dp4_hypoconulid;
{386 dp4_hypoconulid_;
{387 dp4_cristid_obliqua;
{388 dp4_cristid_obliqua_orientation;
{389 dp4_cristid_obliqua_terminus;
{390 dp4_centroconid;
{391 dp4_hypocristid;
{392 dp4_buccal_cingulum;
{393 dp4_talonid;
{394 dp4_hypoflexid;
{395 dp4_distal_fovea_;
{396 dp4_hypocristid_accessory_cusp;
{397 dp4_cristid_obliqua;
{398 dp4_trigonid_mesiodistal_proportions;
{399 M1_2_premetacrista;
{400 M1_entoprotocrista;
{401 M1-3_anterior_cingulum;
{402 M1-2_mesostyle_position;
{403 M1-2_postparacrista;
{404 M1_postprotocrista_length;
{405 M2_postprotocrista_length;
{406 M1_postprotocrista_direction;
{407 M2_postprotocrista_direction;
{408 M1_postprotocrista_terminus;
{409 M2_postprotocrista_terminus;
{410 M1-3_posterior_cingulum;
{411 M1-2_posterior_cingulum_lobe;

```
{412 M1-3_posterior_margin;
{413 M1-2_lingual_cingulum_structure;
{414 M1-2_metastyle;
{415 M1-2_parastyle;
;


proc /;
comments 0
;
```
