# Supplementary material for: The Earliest Known Radiation of Pitheciine Primates
Source: Am J Primatol. 2025 May 16;87(5):e70040. doi: 10.1002/ajp.70040 (PMC12082270; doi:10.1002/ajp.70040)
Supplement: Supplementary file 4 — Appendix 4. DNA sequences of the molecular matrix. [file AJP-87-e70040-s001.pdf]

TAXA	NUCLEAR SEQUENCES																				MITOCHONDRAL SEQUENCES					REFERENCES
	ZFY	ABCA1	ADORA3	AFF2	AFF2.2	ZIC3	APP	ATXN7	AXIN1	BCHE	BCOR	BDNF	RAG1	RAG2	RPGRIP1	NEGR1	FAM123B	ERC2	CREM	FOXP1	ND1	ND2	COX1	COX2	CYTB	
Alouatta caraya	HM756997	HM765258	HM765172	HM764895	HM764810	HM756918	HM764642	HM764399	HM764314	HM764195	HM763993	HM763842	HM759098	HM758927	HM758642	HM760248	HM762089	HM762200	HM763016	HM761499	KC757384					Finstermeier et al., 2013
Ateles belzebuth	HM757002	HM765257	HM765181	HM764894	HM764818	HM756842	HM764650	HM764388	HM764323	HM764109	HM763998	HM763847	HM759107	HM758936	HM758640	HM760247	HM762098	HM762203	HM763024	HM761498	FJ785422					Finstermeier et al., 2013
Brachyteles	HM756986	HM765271	HM765156	HM764911	HM764797	HM756914			HM764298	HM764186	HM763977		HM759083	HM758911	HM758662	HM760264		HM762184	HM763030	HM761515	JX262672					Schrago et al., 2012
Cebus sp	HM757009	HM765278	HM765190	HM764920	HM764827	HM756849	HM764658	HM764408	HM764330	HM764118	HM764007	HM763853	HM759116	HM758945	HM758673	HM760273	HM762107	HM762284	HM763048		NC_002763					Arnason et al., 2000
Aotus sp	HM756999	HM765269	HM765175	HM764909	HM764813	HM756838	HM764645	HM764402	HM764317	HM764102	HM763994	HM763846	HM759102	HM758931	HM758656	HM760262	HM762092	HM762324	HM763019	HM761513	FJ785421					Hodgson et al., 2009
Saimiri sciureus		HM765401	HM765206	HM765053	HM764844	HM756867	HM764671	HM764530	HM764348	HM764132	HM764024	HM763865	HM759131	HM758963	HM758816	HM760405	HM762126	HM762269	HM763149	HM761651	FJ785425					Hodgson et al. ,2009
Saguinus sp	HM757017	HM765392	HM765198	HM765044	HM764836	HM756857	HM764665	HM764522	HM764339	HM764087	HM764016	HM763859	HM759124	HM758954	HM758806	HM760396	HM762116	HM762330	HM763147	HM761642	KC757409					Finstermeier et al., 2013
Callithrix jacchus	HM756990	HM765296	HM765164	HM764939	HM764804	HM756828	HM764635	HM764425	HM764306	HM764113	HM763985	HM763836	HM759090	HM758919	HM758694	HM760291	HM762082		XM_009000180.4	HM761540	KC757388					Finstermeier et al., 2013
Cebuella pygmaea	HM756993	HM765307	HM765167	HM764952		HM756831	HM764638	HM764433	HM764309	HM764122	HM763987	HM763838	HM759093	HM758922	HM758711	HM760303	HM762085	HM762328	HM763046	HM761552	KC757389					Finstermeier et al., 2013
Leontopithecus sp	HM756994	HM765337	HM765169	HM764985	HM764808	HM756833	HM764640	HM764463	HM764311	HM764086	HM763989	HM763840	HM759095	HM758924	HM758746	HM760338	HM762087	HM762329	HM763098	HM761582	KC757399					Finstermeier et al., 2013
Callicebus sp	HM757018	HM765286	HM765210	HM764929	HM764848	HM756869	HM764674	HM764416	HM764350	HM764111	HM764028	HM763867	HM759137	HM758966	HM758682	HM760281	HM762129	HM762289	HM763034	HM761530	FJ785423					Hodgson et al., 2009
Callimico sp	HM756988	HM765291	HM765160	HM764934	HM764800	HM756825	HM764632	HM764421	HM764302	HM764218	HM763981	AY011480	HM759087	HM758915	HM758688	HM760286	HM762079		HM763039	HM761535	NC_024628					Menezes et al., 2013
Lagothrix cana	HM756987	HM765329	HM765158	HM764978	HM764798	HM756823	HM764630	HM764460	HM764300	HM764068	HM763979	HM763832	HM759085	HM758913	HM758738	HM760330	HM762077	HM762323	HM763094	HM761579	KC757398					Finstermeier et al., 2013
Pithecia pithecia	HM757021	HM765380	HM765215	HM765029	HM764853	HM756875	HM764678	HM764507	HM764356	HM764072	HM764033	HM763869	HM759140	HM758971	HM758792	HM760382	HM762135	HM762204	HM763130	HM761627	NC_064161					Janiak et al., 2022
Cacajao sp	HM757008	HM765283	HM765187	HM764926	HM764824	HM756848	HM764655	HM764414	HM764328	HM764110	HM764004	HM763852	HM759113	HM758942	HM758678	HM760278	HM762104	HM762297	HM763032	HM761527	KC959985					Finstermeier et al., 2013
Chiropotes sp	HM757010	HM765295	HM765194	HM764938	HM764832	HM756853	HM764662		HM764335	HM764183	HM764012	HM763856	HM759120	HM758950	HM758713	HM760290	HM762112	HM762183	HM763067	HM761539	KC757393					Finstermeier et al., 2013
Hylobates lar	HM756976	HM765324	HM765143	HM764973	HM764784	HM756810	HM764617	HM764455	HM764286	HM764136	HM763965	HM763822	HM759071	HM758898	HM758733	HM760325	HM762064	HM762210	HM763091	HM761574	NC_002082					Arnason et al., 1996
Presbytis sp	HM756964	HM765375	HM765125	HM765024	HM764766	HM756792	HM764599	HM764502	HM764269	HM764081	HM763947	HM763806	HM759054	HM758880	HM758787	HM760377	HM762046	HM762250	HM763134		NC_008217					Sterner et al., 2006
Xenothrix		MK073952	MK073943				MK073953						MK073949	MK073950	MK073960	MK073959	MK073958	MK073957	MK073954	MK073945	MK073942					Woods et al., 2018
Miopithecus sp		HM765351	HM765119	HM765001	HM764759	HM756785	HM764592	HM764479	HM764262	HM764121	HM763940	HM763799	HM759047	HM758873			HM762039	HM762215	HM763117	HM761602	JQ256997					Guschanski et al., 2013
