# Supplementary material for: The Earliest Known Radiation of Pitheciine Primates
Source: Am J Primatol. 2025 May 16;87(5):e70040. doi: 10.1002/ajp.70040 (PMC12082270; doi:10.1002/ajp.70040)
Supplement: Supplementary file 5 — Appendix 5. Matrix of the combined morphological and molecular characters. [file AJP-87-e70040-s002.pdf]

```
nstates dna;
xread
'Exported by SequenceMatrix 1.7.8 on Wed Nov 03 16:09:44 ART 2021.
'
20766 55
&[num]
Simonsius
	?????200?1?1?120?1110201110011110111111100001?201?100111001210?1110?
11011010000001?11?3????0??????0?????1?0002100201 2?02101112111110010010 30
00?1312201110 00???0001021110111102[0
1]002111000011111011210 0???????????????1??122011?0000000100001000310022 00
020?010[0 1]01002[1
2]001110000???1?????????????????????????????????????????????????????????
???????????????????????????????????????????????????????????????????1?211
00--??210011
Apidium    ?????00??1???1???101???????????1[1
2]??????1??011?201?101111?0?2102111 0????110?0??000101111001000000000001020
120002100202 2?0001111001111101001101000?132[1
2]20111101???0002021110111101000110[1 2]00000111[0
1]11212100???????0??????1??[1 2]221111000031000[0 1]001000220032 2001[0
1]0?100020231002110201110[0 1][1 2]0211101010010??211010010100[1
2]1?020211001[0 1]10101002101002021011[1
2]?????0????0????????????????????????????????2???????????????????2??1-
2000011210001
Cebuella   11010[1 2]0011012210?1010110[0
1]1200?11101112100011020111111100121021110?01011001201001110111011021111
10101101000021000022?12001200101000101001020 11?2212?110?10???00??0211100
00111233??11100?11?20002150101001022000000001100010[0
1]000001002001100 00?000000022-0???2100100100?00??2?0?30223?[0
1]?0?020??0??200[1 2]21?[0 1]1[0 1]??21122021100 20????2??120100[3 4][1
2][1 2][1
2]0111?10?2100?201020100000??00110000??11?00011211000112 2?2001101200 1111-
1122102220[0 1]-0[0 1]
Callithrix [0 1]101000011012210?101011[0 1]01200?111[0
1]111210001102011111111001210211 10?0101100120100111011101 2[0
1]211111010120[1 2]000021000022?12000200101010101 0[0 1]102111?23[0
1]3?110?00????00??0221100001102 33??11100?11?20001150[0 1]010022110000 00[0
1]110001200000010020011000 0?00000[0 1]022-0??02100102100?0[0 1]??1?0?[2
3]0[2 3]23?[0 1]?0?020??0??20[0 1][1 2]2[0 1]?[0 1]0[0
1]??211?201110020????2??[1 2]20100[2 3][1 2]2[1 2][0
1]1111101200012010201 0??????????????????????????????????????????????2111
112103220100[0 1]
Saguinus   [0 1]10010001101220001010 10011[1 2]00?111[0
1]11121000110201110111001210211 10?0101100120000112001001210000000010212 0
0002100002 2?100012011[0 1]10101010[0 1]001011?13[0 1]2?[0 1]10?[0
1]0????00??012100000110[1 2][2 3][2 3]?011111?11?200110[4
5]1101002111000100011000001 00000[0 1]002100[0 1]0100?000000022-
0???100010210 0?0[0 1]??2?[0 1]010[1 2]1[0 1]1[0 1]101020010??20[0 1][1
2]2[0 1]0[0 1]0[0 1]10211[1 2][0 1]0111002[0 1]????2??12[0 1]1003[1 2]2[1
2][0 1]11[0 1
2]?10?2100?20102010000 2?001110000?011?000102110001022?2021111000 1021--
220113320 1001
Leontopithecus   0001100011012210?101010[0
1]00200?11111101000110201111111100121021110?01011001211001020110011 20000
```

00001021200002100002 2?1210[0
1]20110101010100102011?1304?110?10????00??02210000[0 1]1102[2 3][2
3]?010011?11?20001051[0 1]010020110001000110011110100000021000110 0?[0
1]00000022-0???1000103000?00??[0 1]?[0 1]?[1 2]0[2 3]23?[1
2]?0?020??0????112[0 1]?[0 1]?1??2122101110020?0??200????003[1 2]2[1 2][0
1]11[0
1]11012100120102010002 0?101110000?011?0001011100011 22?2021101000101 1[0
1]-122[0 1]022211000
Lagonimico ?????000?101????????1??0[1
2]????????????1????0???20???11111??????1111???0???????00111221001120010
000010012?00021000022?100012011000101010000101 1?1????0110201???00?0022100
000111222200001201112001114110100202200000000110011100100010021001110121 00
000022-0???2001103[0
1]00000000???
??112-
212113321[0 1]001
Callimico 100001001101221 0?1010000[0
1]1100?1110111210001102011110111001210211 10?01?1101121100102001001000000 0
0001020[1 2]00002100002 2?10102201101010101 0000[1
2]011?1302?0110100??00?00221000001102333?211000120200011411010020220000 00
0011001020000000000210000100110001101200-?02102102100000000 00?[2 3]020[0
1]?1?0?020??0??201[0 1]2[0 1]?[0 1 2]1[0 1]??210?1011100[0 2][0
1]????200120100[2 3][1 2]2[1 2]1[0
1]11?10?2000?20102010002 2?102100000?011?00010210000121202021201100111101 1
01113321[0 1]000
Mohanamico
???
??????????1011????1001??1?10000100?2?01021000022?101012011000101010000 20
11?1311?0110101???00??02210000011123 3??10022?11?100011?130???????????????
???
???
???
Pithecia 11011000110122201101 0[0 1]0121100?12[0
1]1111110002002011100111001210211 10?010110112201111221100011212022000001 2
10102100002 2?221[0
1]22022000000011000301 1?1301?0110101???00?0120100001111 2332?000222100200 0
00320010021110001101 1[0 1]001111210000[0 1]12100002012310 0[0
1]00002200?2002[1 2]02000010[0 1]0210?30[1 2]001????02???0??200[1 2][2
3]1?011??[0 1]11110111001 1????000120100322[1
2]0111?00?2000?2000101101320102101100000001111111000013 12?102110100011 11-
-3111011210001
Cacajao
111110001101222 1?10101012110 0?1210111011002002011100111001210211 10?
01?110112201111221100011212022000001320102100002 2?2110220220000000110003 0
11?1303?0110101???00?0220100011110233[2
3]?0002221002000003200100221100011001010??12210100112010002 0??310000000[2
3]200?2001100000000[0 1]021[0 1]?1[0 1]201?2?[0 1 2]?021??0????1[1 2]3[0
1]?[0 1]?[0 1]??[0 1 2]11?00211001[1 2]?????00?????0[3 4]12[1 2]011[0
1]?00?2000?20000010????????????????????????1111001012 12?10211010001131--
3111133200-22
Mazzonicebus
???
??????????0111????10011121201100000?3?000210000?2??2?0?20?20?010101001020
11??300?011?10?1??00??020111000?11233??10011?11?101100?2[0

1]01??10?????0001?100001?1000002121010110021100000111100?2[0 1]0[1
2]102[0 1]00?001?[0
1]0???????????????????????????????????????1??????????????????????????????
???????????????????????????????????1110120013011 2?21?112100011[2
3]1211113321001 1
Chiropotes 1?1??00011012220?101000[1 2]21100?1[1
2]1111101100200201111011100121021110?01?1101122011122211000112120220000 01
3001021000022?2211220210000000110003011?1303?0110101??00?0220100011110 23
3[2
3]?0002221002000003100100221100011001010??12210100002010002 0??310000001[2
3]200?20011000000100121[0 1]?1120[0 1]?2?0?021??0??201[1 2][2 3][0 1]?[0
1]21??[0 1 2]11[1 2]001110001?????????????00[3 4][0 1 2]2[1
2]0111?00??000??0001011??31-
-3111133200-12
Nuciruptor
      ???
??????????0111????10?1??2?202200?0013?00021000022?1?101211201000001011020
11?1301?011010????00??020100001111233??00022?11?200000?200???????????????
???
???[3
4]???
?????????????????
Proteropithecia
      ???
???????????????????00???2120??00000?3?0002100?0????2?0??????????????01???
??????????1?10????00??120110000111233??00022?11?200100?200?????????????1?
11???
???
???
Callicebus 0001011011112220?101000111200?121[0
1]1112110021020111101110012102111 0?010110112101110220100101101001001000 10
00021000022?121[0 1][1 2]20222010001010000 2011?1[2
3]00?0110101???00?0021100000111[1 2][2 3][2 3][1 2]11112221101[0 1]0[0
1]1131001002111000 21011[0 1]101110110000[0 1]121000110012100[0 1][0
1]00022000200[0 1][0 1]020000001[0 1]0010[1 2][0 1]213111[0
1]102001000201[0 1]2[0 1]000[0 1]20[1 2]11?10211001[0 1]1110100221000[2
3]12[1
2]0111100120001200020110022011211000010100001102110001222202110100001111
-1001133200000
Homunculus 11011000[1
2]101?220?101000111200?111?111?1100?1?201?100111???21021110???[0
1]11011210101[0 1]2?110010?10100100100?1?00021000022?121[0 1][1
2]201201000101001020?1?1300?0110100???00?10211[0 1][0 1]000111[1 2][2
3][2 3]?011011?1[1 2]?2[0
1]00113200100?1?10?0??01111011100000001221010010[0 1]12100[0 1][0 1]000[0
1]10002002202000000000?00202011110100?01?????1121?0?1??2??2??????????????
???102012011312?2?21?
0120010111-2111133210011
Cebus 1000[0 1]00011012200?1010[1 2]01[1 2]1000?121010121[1
2]0020120111001110012102111 0?010110012210011210100121000000000101121000210
00022?11112201[1 2]0000010100102011?1303?0110[0
1]00???00?0022100000111233 2?0002221102011104210100220000020011021112201 00
001[0 1]21100010222100100001210?2001[1 2]0[1 2 3]0000111120[0 1]?1021[0
1]12?0?020010??1[0 1][0 1][1 2][2 3][0 1 2]?[0 1]0[0 1]??[0 1 2]11[1 2][1

2]01110011????200120100[3 4][0 1 2][2 3][1 2][0
1]1111001?0001?000201000220112101200101000001111000013011202110100011 21--
2100?33210100
Aotus 10010[0 1][0 1]111012200?10101011110 0?1[1
2]1111121102210201111011100121021110?0101101111011102201001211010000001001
1000021000022?1210220120100010100002011?1[1
2]01?0110101???00?10211000001112333?1112221102[0
1]00003100100222100020 0[0 1]1[0 1]10111[0
1]21000000210000101121000000002200?200[1 2]10[0 1]0000010020[0 1]01[0 1][1
2 3]101[0 1]1[0 1 2]10200100020[0 1][0 1 2]2[0 1]0[0 1]1[0 1]20[0 1
2]11?10211001[1 2]1110100221000[2 3][1 2]2[1 2][0
1]11110012000120002011002211021000001011?00110211000132 2?2021101100 1111--
2220033210000
Aotus_dindensis
???????1?????????????????????????????????2?????????????????????????
??????????0111????1001111000000010011?00021000022?10101201201000101000020
11?1311?0110101???00?102110?0001112333?100222111100011???0?????????????11
1101?1?????????????????11??
???
???
Tremacebus
??0??0??1111?200?10?01001?200?1210??12100111?201?11?11100121??111??
010???1121????????????????????????????????10?????????????????????????????
????????0??
??011??????????????????122100???001?0??2?011?2????0??020?????????????????
???
??3??1111332100??
Alouatta   10000[1 2]00[1 2]1212[0 1]20110100 0221200?111[0
1]0100100021020111001110011102111 0?0101101120011122211011000000000001 01012
0002100002 2?1210220[1 2]2010001010000001 1?13[0 1][1 2]?0111[1 2]0[1
2]???0000022[0 1]10000111[1 2][2 3][2 3][1 2]0211112121[1 2]0002213[0
1][0 1]10000000000100 01101[1 2][0 1 2]00300000[0 1 2][0 1]2100001[0
1]0232000000001100?120[0 1]10[0 1]1000011121[0 1]?102131[0
1]?02020??0???11[2 3][0 1]?[0 1]?[0 1]??[0 1]2220001100 11????000120100[3
4]2[1 2][2 3]0[0 1]1[1
2]?00?2000?200111111012?102100000?011?001102110001202121012012001121-
02111133210-21
Brachyteles 11011200[1 2]1212[1
2]20?101000211200?1111010111002002011100111001210211 10?011110112?01112221
1001000000000001020120002100002 2?1210221121110010000000011?1[2
3]01?001?101???0000021010000112 2332?211002122000012331010000000 10?1001?00
110[0 1]300000002010002 1??3100000001 0?1?21000000000000 11210?[1
2]022310?22021??1????[0 1][1 2][2 3]0?[0 1]?0??122??0011000[1
2]????????????042220[0 1][0
1]1?00?2010?200121010012?002110100?0110001101010001322021212012001121-
02111133200-0[1 2]
Stirtonia
???
?????????????1????1???????0???00?0?0?????????????????????????0???????????
????????011?2?????00??02?1100001112[2 3][2
3]?020012?12?200011?301?????????????01001[1 2][1 2]1010000011201000[0
1]10132000000001101?[1 2]20011120000111[1
2]2??

??2130212113321
0121
Ateles   110[0 1]11001111212001010001111 00?1[1
2]1101011100200201110011100121021110?01[0 1]11011220[0 1]1122[1
2]11001200000000010122100021000022?12102201101000[0 1]0100102011?130[1
2]?0000101???00?0020100000111[1 2]2[2
3]2?1001221112010113200100121101011001110001130000000021000010223[1
2]0000000210 1?20001000000001120[0 1]?10[1 2 3]231[0 1]1[0 1
2]2021101????[0 1][1 2][2 3][0 1]?[0 1]?[0 1]??[0 1 2]12200011000[0
1]?01010022?100[3 4]2[1 2 3][1 2 3]011[0
1]1001201012001210100 32?002110100?0100101111100021112110211110001121--
2111133200-00
Lagothrix 110[0 1]020011112110?101000211[1 2]00?121[0
1]0101110021020111001110012102111 0?0111101122011122[1
2]110012100000000102222000210000 22?12102201201000101000 01011?1301?0110101
???00?0020100000111 2332?10002211120001132001001211010110011?0002120000000
2100002 0?23[1 2]00000002[0 1]00?20001000000000 11210?[2 3]02231[1
2]?02021???????1[1 2]3[0 1]?[0 1]?1??[0 1 2]22200111001 1????0??2201?04[0
1][1 2 7][1
2]0111100120001200121010022001211000010110001111100001322220212011101131-
-3011133200-00
Aegyptopithecus
      0?00?0002101?020?0011101211 00?120211?0100021?201?10011101121021110?
01011001201001021110010000100000101012000211?2022?1?1?020010100010 10?1030
11?2301201111 01???010102110010[0 1]00100001000001111111[1 2]1[0
1]1300??????????????031102201?100[0 1]121210001100012001110010?000[0 1]0[1
2]10210001100021010[0 1]0011101010010???0?[1
2]2?0??0?????2??????????1110111121100301 31?12??0?20????0??????1?2??0?2?1?2
?0?1?1120?10211100130012101221210001?111110011110001
Catopithecus   ?????000?1???1???10111 0121????1[1
2]1?????11?011?201?1???1100 1210???10?01011?11??000002?01?0??00010000010??
12000211?201121?1?02[0
1]0201000101 1?0020110123120111101 1??0001001001000111111101 00000122200[0
1]1[0 1]30101000011000200031 0012?1??000?0020001011002200 0101100?000[0
1]0[1 2]10220001010111 01[0
1]0001110101??????1210?00?000????2???????????11?0111211????????????????????
???11211220011211011
Proteopithecus
      ?????000?1?1?2?0?10?010121100?12121???11?011?201?1?0111001210?1110?
010?1?11??100002101????????????????????2???21002221210100210101000100121021
01011101011 11001??000[0 1]0120000001 1111[1 2][1 2 3]00[0
1]01101222001113010???1???0?????0111012020?000000201110010111000001100?0?
0011102200 00000[1
2]0110111211 10101?0???221010???10????11??1100??101010?1201??????????????????
??10[2 3]-
1220011221011
Branisella
      ?????????????1???
??????????0001????1001?[1
2]?0?01000102?11000210000 22?12101201101000 1011000 3001?1313?0111101???000[
0
1]02110000011111111011110112200011 3100?????????????0?11010001000 0?0020110
100011100[0 1]001110?0?[1 2][0 1]01102000000 00[1
2]0??

???11[2
3]11111111211011
Canaanimico
??
??
??
???????????????????????0???000?0111[0 1]0?-200121[2
3]000?00??1???
??213?
2111133200011
Soriacebus
??
??????????0111????1001112120110000013?00021000022?11101201201010101001020
11?1312?0110111???00?1020110000111122101001111112010003120??????????????1?
1022[1 2]1000000022210101100211000001111100?2[0 1]01102000000[0 1]0[0 1
2]1???
???11[2
3]12111133200011
Hylobates
100000002101212 0?1010?012100000211111210002102011100111011210 21111?
010110002210012211100111000000010[1
2]120000211?2222???1?0201101000??10?003011124211011 1101???000102010011000
1000010000001111101133?0100????0?????0312?22?1?0000?0021?00?200?22000000
020-0?0002[0
1]0200?00?22222?3112311112??110?????1130?0?1??0223001110001????112201?03
1301121?????0?????0???????????????1?1???1?1010?1011001102002100110101 00121
--3220000220000
Miopithecus
11111000210121 10?1010001110020021111121100200201110011101121021111?
0101100022100122111001221000000010?212000211?2222???1?0201101000??10?0030
1112420101111 01???00?0020100100002 2332?200000122000011 33?0100????0?????03
10?22?0?0000?0021000020 0?2200000000320??000220000?01012222????2?022????11
????????????????11?200011002??10011101010030??1121?????0?????0????????????
???21--3111133210-00
Presbytis
11111000210120 10?1010001210010021111121000210201110011101121021111?
0101100022100122111001110000000100212000211?2222???1?0201101000??10?0030
1112311101111 01???0001020100100002 2330?200000122000011 23?0100????0?????03
10?22?0??000?0021000020 0?2200000000220???0???0000?00022222?20223022????011
1????1220?1?1??111100?110020????110201?0302111210?11100????0101?????????
???11--2??????200-00
Acrecebus
??
??
??
???????????????????????????001?0?0221??2001202000?21??1???????????????????
??
???1?2?1?0?1?3220000
Cebupithecia
??0??????????1?0?10??102???????????????1??0????????0011100121021110?
??011?1???0111????10?1??21202?00?0003?2102100002 2?2?1?12012010000010?1020
11?1300?0110?0????00??120100?0?1?1?3???00?2??11?20100?4100??0???????????1?
11011101100000021000020?22000000000220??2000102100?00??0??????????????????

???????????????21??10?1100?1?0??100021?0?3????111?????????????2??????????
??21[2 3]-2121133210001
Chilecebus ??????????2???0??1?1???21??????????????1?0?[1
2]?2??????????????????10??????????0????????????????????????????????1?????
???
????????????000220??2?011020110121000111?10101100?21000100010?002002202[0
1]000010001??
??1?1-
100--33220010
Antillothrix
1101100011212120?101020211200001120111100021?201011?11101121021110?
010110112?0101????1?????????0??????02?????10000??????????????????????????
????????011010????????021110000111133??011[1 2][1
2]??1?201022320110?202000000011020[0
1]12011000100210001101221000000100?0?20001[0 1]30000010[0
1]11?11000112102020 21???10022000001 1????000110011????????????????????????
???112-
2011133210011
Paralouatta
?00??1001??12021?101?2021?1?0?02?10?1?1000?1?2???11?111001?????110?
010???????011122211001020000000000000?0021100002?202001101101010100001000
12?1313?0111111?????000201100001111332?111222122210022432110?2021000200?1
??[0 1][1 2][1 2]?211000100210001110[1 2]1100000000100?210002220000111[0
1]21010111121111201????????20?????0???3001110011??????0??????????????????
?00??210??2?[2
3]12111133220021
Xenothrix
??0????0?111?1?1?1010???1?????02?20??????0?1???????????????????????
??????????010112111??1??????0?0???????????1000022?0?1?22???001?01010?1000
0??1?00?111?01?????2??02010001111000?200122?11?2110115200100[1
2]??????????1??011[0
1]0?1000?00210000200?200000001120??1001?00000021??2010100011[1
2]1111200????????3???0000??
??31?-30?1?3?210-21
Insulacebus
???????????????????1???
??????????0??1????1?01??0?0000?0?000??00??1000022?021022111000?0101001010
01?1201?0???110???00000211100111111331?111222110200022320 1???20200000001?
02001001100010021000110??21000000000?0?20001030000010121?????????????????
???
??213-2001133210011
Perupithecus
???
???
???
???????????????????????0?0?000002?-0-?-
0002203000?0???1???
???
?102-12?1?1?201011
Miocallicebus
???
???
???
???????????????????????012?00000?0210??20??102000?00??0??????????????????

???
??1??-1?0?1?3200000
Talahpithecus
???
???
????????????1?????????0?0???0?0??11?1?00?0???11?0000?????0???????????????
??????0?0?00???2??1?001??0?000002?-
0????002213000?0???1???
???
?????102-12?1?1?201011
Parvimico
???
???
???
?????????????????????????1?000001?00????102002100?0???1??????????????????
???
??112121?1?3?2?0011
Killikaike
110??000?1012??0?1010???2???????11?1???20010?201?1?????????????????
?????????2????????????????????????????????10?????????????????????????????
????????0???0??????????0?
01?1?1?000??0???10100?0022100000001100?20022030000011011?????????????????
???
??111-1111133210011
Saimiri 11111000110122000101 0[1
2]0021000?111111121210201201111011100121021110?0101100122100112111001[1
2][0 1]1000000010112200021000022?1210220220100010100001011?1303?0110[0
1]00???00?0022110000111 2[2 3][2 3]20111001120200111411010022110002 0011[0
1]2[0 1][0 1]11[1 2]01000000210001101211000000001100?200[0 2][1 2]0[2
3]00000111[1 2]010202001210202001 0001[0 1]1[0 1]210[0
1]01202112202110020111020011 0000[3 4]12[1 2
3]01111001200012000201100121112101200101000121021100013011212121120011112
-111113321000[0 1]
Dolichocebus
??0?00?0?1?12200?10?02002??00??21???121?1010?2?1??1?11100121021110?
0101101122?0??????1?0?0?0000??0010101200021000022?12101201201010101001020
?1?1302?011010???00??021110000111 22?011012?11?1[0
1]11103110100??1?0001000?10011?00100002220110110012100000001[0 1]0002[0
1]01[0
1]02000001111??110100????????
???11
211111133210011
Laventiana
???
??????????1001????10?1??????1?0?????1?01021000022?12101201201010101000020
11?1302?011010???00??021110000111233??11112?11?210111?100????????????????
???
???
???
Panamacebus
???
????????????????????????1000?001102?1?0002?0000?2?1210?20?10?010101000010
11???1???
???????????????????????0?200000000110?-200[1

2]103000?01??0??
???1
12-1111133200000
Neosaimiri
	??
?????????10011???1001011000000102?12000210000022?1210220220101010100002[
0 1]11?1301?0110100???00?00221100001112[2 3][2 3]?01101221212[0
1]011141201??20[0 1]10002001?[0 1]200120010000[0 1]021010110011100000[0
1][0 1]10?0?2[0 1]1[0 1][0
1]030000010010102010012102???????????????????????20111020???020?????????
??11
111221133210011
Ucayalipithecus
	??
??
????????0???1?0???00000?2?????????0?0010?0?001001010?13??0??????????????
???????????????????????????0032?0010??10????????????????????????????????
??
??00???????0??
Qatrania
	??
???10020?2?0?0??11?01?1?11100?1000
?????????1111?011?00000?2?????????000011100001001112113020??????????????
???????????????????????????0032?0010??10?????????????0??????????????????
??
??0????????0??
Carlocebus
	??
??????????0?01????1?01????????0?????1?0???100002[1
2]112101201201010101?00102011?1?00?0110100???00?002111?000111[1 2][2 3][2
3]?011111112?2[0 1]0[0 1]114200?????????????????1?111[1 2]1?0?0000?[1 2][1
2]21010110[0 1]221000[0 1]0001[0 1 2]00?2[0
1]02102000000100??
??
?111120011332100[0 1]1

&[dna]
Alouatta
??CCTTTCATGCCG
GTTCCCTTTCCGTCATGAGCATGGACTACATGGTGTACTTCAGCTTCTTCACCTGGATTTTCATTCCCCTGGT
CATCATGTGTGC--AATCTATGTTGACATCTTTTACAT-
CATCCGGAACAAACTCAGTCAGAACTTCTCTAACTCCAAAGAGACAGGTGCATT---
TTATGGACGGGAGTTCAAGACGGCGAAGTCCCTGTTTCTGGTGCTTTTCTTGTT-
GCTTTGTCGTGGCTGCCTTTGTCCATCATCAACTGCGTCACCTACTTTCA-------------TGGCGAG-
TACCACAGCTTGTGCTGTACTTGG-
CATCCTGC??ATGTTCATAAAC
CGCTGATTATTTTCAACTAACCATAAAGATATCGGAACACTATATTTACTATTTGGCGCATGGGCAGGGGCAG
TAGGAACAGCCCTAAGTCTCCTAATTCGAACAGAACTCGGTCAGCCTGGAAGTTTATTAGAAGACGACCATAT
TTATAACGTTATCGTTACCTCTCACGCATTTATCATAATTTTCTTCATAGT---
AATGCCAATTATAATCGGGGGCTTTGG---
AAACTGACTTGTCCCCCTAATAATTGGTTCCCCCGATATAGCATTCCCTCGAATAAATAATATAAGCTTCTGA
CTTCTACCCCCA--TCCCTTCTTCTACTACTCG-
CATCCTCAACCCTAGAAGCCGGAGCCGGTACTGGTTGAACAGTCTACCCCCCTCTAGCGGGAAATATATCACA
TCCAGGAGCCTCTGTAGACCTCACTATTTTTTCACTCCACCTGGCTGGTATTTCTTCCATTCTAGGGGCTATT

AATTTTATCACAACAATTATTAATACAAAACCCCCAGCCATAACCCAGTATCAAACACCTTTATTTGTCTGAT
CTGTCCTTATTACAGCAGTCTTACTACTTCTGTCTCTTCCAGTTCTGGCTGCCGGAATTACCATATTACTAAC
AGACC--
GCAATCTTAATACTACATTCTTCGATCCTGCTGGTGGTGGGGACCCTATTCTATACCAGCACCTGTTCTGATT
TTTTGGACATCCTGAAGTATATATTCTCATTCTTCCAGGCTTCGGAATAATTTCACATATTGTAACATATTAC
TCTAACAAAAAAGAACCATTCGGGTATATGGGGATAGTATGAGCCATAATATCAATTGGTTTCCTGGGGTTTA
TTGTATGAGCCCACCACATATT-
TACTGTGGGCATAGACGTAGACACACGTGCATACTTCACATCAGCCACTATAATTATTGCCATTCCTACAGGA
GTAAAAGTATTTAGTTGATTAGCTACACTGCACGGCGGCAATGTCAAATGGTCTCCCGCAATACTATGAGCTC
TGGGCTTTATCTTCCTTTTTACCGTAGGTGGACTAACAGGAATTGTATTAGCCAACTCATCATTAGACAT-
CGTCCTACATGACACATACTATGTAGTAGCCCACTTTCATTACGTTCTATCCATAGGAGCAG-
TATTCGCCATTATGGGAGGTTTCATCCACTGATTCCCATTGTTCTCTGGTTATACTCTCGACCAAACCTATGC
TAAAATTCACTT-
TACCATTATATTTGTAGGCGTAAATCTAACCTTTTTCCCACAACACTTTCTTGGCCTATCTGGAATGCCCCGA
CGGTATTCAGATTACCCTGATGCATACACTACATGAAATATCGTATCATCTGTAGGCTCATTTATTTCATTAA
CAGCAGTTATCCTAATAATTTTTATAATTTGAGAAGCCTTTTCCTCAAAACGAAAAGTCTTGACCGTTGAACA
ACTAACTACCAATCTAGAATGACTCCATGGCTGCCCTCCTCCTTATCACACATTTGAAGAGCCTACCTATGTA
AAATCTT??????????????????????????????ATGGCACACCCAGCCCAACTAGGCCTACAAAACGCTA
CATCCCCTATCATAGAAGAACTTATCGCTTTCCACGACCATGCCCTTATAATTATTTTCCTAATTAGCTCACT
AGTACTATATGTTATTTCCCTAATGCTCACAACTAAATTAACCCACACCAGCACAATAAATGCCCAAGAAATT
GAAATAATTTGAACCATTCTACCTGCAATTATCCTTATTATAATTGCTCTTCCATCCCTACGCATCTTATATA
TAACAGATGAATTTAACAAACCCTACTTAACTCTTAAAGCCATTGGCCACCAATGATACTGAAGTTACGAATA
TTCAGACTATGAGGACCTAGCCTTCGACTCCTACATCACTCCTACATACTTTCTCGAACCTGGGGAATTCCGA
CTCCTTGAAGTAGACAACCGAACAACTCTACCCATAGAAGCAGATATCCGTATACTAATCTCATCACAAGACG
TATTGCACTCATGAGCCGTCCCATCACTAGGCGTTAAAGCAGATGCAATCCCTGGACGCTTAAATCAAGCTAT
ACTGGCCTCCATACGACCAGGCCTATTTTACGGGCAATGCTCAGAAATTTGCGGATCAAACCATAGCTTTATG
CCCATTGTCCTAGAATTTATTTATTTCCAAGACTTCGAAGTGTGAGCCTCATACTTATACATCGTAT??????
ATGACTACCCCCCGCAAGACACATCCACTAACAAAAATCATTAACAACTCATTCATTGATCTCCCCACACCAT
CCAACATTTCCGCCTGATGAAATTTCGGCTCACTCCTAGGTATTTGCCTAATTATCCAAATCACTACAGGTCT
ATTCCTAGCCATACATTATACACCAGACACTTCAACTGCCTTTTCCTCAGTCGCCCACATCACCCGAGACGTC
AACTACGGCTGAATAATCCGCTACCTACACGCCAACGGCGCTTCCATATTCTTCATCTGCCTATTCCTCCACA
TTGGCCGAGGCTTATATTATGGGTCATTCCTTTTTCTGAAGACCTGAAACGTCGGTATTATCCTCCTACTCAC
AACCATAGCCACAGCATTCATAGGCTACGTCCTCCCATGAGGCCAAATATCATTCTGAGGGGCCACAGTAATT
ACAAACCTTCTGTCAGCCATCCCATACATCGGATCTGACCTCGTACAATGAATCTGAGGTGGGTTCTCAGTAG
ATAAAGCCACCCTCACACGATTTTTCACCTTTCACTTTATTTTACCCTTTATCATTGCTGCCCTAGCAACTAT
CCACCTCTTGTTTCTGCATGAGACAGGATCAAGTAACCCATCAGGAATGGCATCAGACCTCGACAAAATCACA
TTTCACCCCTACTACACAACCAAAGACATCCTAGGCCTAATTATTCTCCTCCTATGTCTAATAAGCCTAACCC
TATTTTCACCTGACCTTCTAACCGACCCAGATAATTACACACTAGCTAACCCCCTCAACACCCCACCCCACAT
TAAACCAGAATGATACTTCCTATTTACATACGCAATCCTACGATCTATCCCTAACAAATTAGGAGGCGTCCTA
GCCCTAATACTCTCCATCCTAATTCTTATAATTATCCCCACCCTCCACCTATCAAAGCAACAAAGCATAGGAT
TCCGACCTATCACCCAAATCCTATTCTGAACCCTAGTAGCCGACCTATTCACACTCACATGAATCGGGAGCCA
ACCAGTTGAATACCCCTTCATAACTATCGGCCAAACCGCATCCATCATATACTTTCTAATTATCATTACACTT
ATTCCCCTCTCCGCCCTAATTGAAAACAAATTACTTAAATGATAA?TTAGGTTAAATTTATTCAAGGGAATGA
GAATTTATGGGTATAACTGTTAGCTGGAAAGCCCTTCCTCACCTCCAATCCTCCTTTCAAACTACATTTCC-
TCCTGAAATTTCCCTGTGGGATGAAATGTTCAATTATTAGTCAATCAGTC----
TCTTATACGTTATCTGGCTTTAC-TAT----
GCCCTTGATAGTTCTTGTAAAATAATCTAATATTGTGTTGTTTTGGTATCTTTGTTTTGGAAATGGAAGCATT
TTGTATGAATCACCTCTTTAGCTGTGAGGAGTTGAATTTTCTTAGTTCTGCTTGCCTAATGACCAGGAATAAA
--TACATCACAGTCTCTAACCCCACTTTTT-
GGTGGAAAGAAAATCTAAAAGTAGCAACATTTAATGGAACAAAATATCAATAATCAAAGAAACTCCCCTGTGA
AACTGTAATATTATTTT-
AAAATCAACATTGTTCTAAGGTAGCTGACATGTGTGATTTTTCTATTTTGCCTTTTCACTGACAATATACCCA
TAAATCT-
TGGTGGTGATTTTCTCTCCAAAGTTAATTTCTGGGCCATTGGTGACTGCACTATCTATTGTTCTAAACTGTTG

TCTTAATTGCATTCTTTAAAAATGTAATATTGAAGGCACATAATATTGAAGATGACTCCAGGATGAACCCTGA
GTTCGCAGACCGAA-
TAAAACAGCTCGATAAAGAGGCGTCTTACTACCGTGATGAATGTGGCAAGGCCCAAGCAGAAGTGGACCGGTT
GCTGGAGA??????????????????????????CAAGTACTTTGATCTTTCTTGGAGCAGGTTCCTTCAAAA
CGCTTCTCCAAAGGACGGAAGTCCCTCCAGTCTGGCTCGCTGCTCGCAGTGGTGGGGAAAGCTGAGGTAATTC
CCCGGTGGGAAATCTGTGAGGCCCGTGAGGCCCCTGATACCCCGCCACCAGGCCCATCACTGATGGCCCTAAG
GGCCTCATCTGATACTCTAAGACGGGCTTATCCTGTCGGATCTGGGCCTCTCGGACTTGAGCCTCTCTAGAAC
GAGCCTCTCGGGCCTGGGCATCTCGGGTTCTGGCCTCCCTGGCATGACTTTCCCAAGTGTGGGCCTCCCTG--
GCATAGGCTTCCTTACCCTGAGCCTCTCGAGTATAGGCCTCCCTGGTGTGGGCCTCCCTGGCATGAGCTTCTC
GTGCACGTGCCTCCCGGGCCTCAAGCTGCTCCCGCCGAAGCTCCCAATACAACAACTGTTTCTGGATGGTCA-
CTAGCCGTTCCTCCTCTGTCTCCATTGCCCCAGGAGGCCGGGAGGAAGAAAAGGGCTCAAAGTTCAAGAAGGG
CTCAAACATCTCAGAGCTTCGACCATGGAGGTCATAAAGGCAGTCATCCCCAGGTGGAGAGTTCTCAAGACTG
TCATCTGGCTCATAGAACTCATATAGGGCATCTCCACTGTAGCTGTCTCGGGGCAGACAATCCCTGCGGACAA
GCCCCAGGGCCTCACCTGA??????????????????????????????TTGATGAACTAAAAATGGTTCCTG
AAGGTCAGATTCATGTACAGTGAATGTTCTCCACTGTTGTGGTAGGCATGTTTCATGTGTGGGGACATTGGTG
TGCAAGTTTCATGTGTCTGGAAAACACTAGCTAGAACATTCGAAAAATTTCATTAACCCAGCTCTGGAAGGTC
ATCTATGAATTCTTCCTATTCCAGTGGCCTTTT-
AAGTGTAGATGTAGATACACCGTAATTTAAAACAGATGGGCATGTCTGAATATTTTCATATGCAAAATGGGAC
AGATGTATATGAATGGATAAATTATCCTTTTAAAAAGATACTTTGTAGCACAAAATATCACCAATGACTGGAA
CAGTGAACCTGCATTCAGTCTAGGGCAAACACCAGGAAGACACTATCAAAACATCTAA-
CTATCTATAAACTAATTCCAATTAGTCTACTTTGTCTTCATACAGAAAAAA--TTTTT-GATTTTTAAA---
TACATATTGGGTGCCAATCCATATCCTTT-
GAGCTGCTAAAGCCAGCATATCACAATAGAAGTTTACTTTTCC??????????????GTGTTTATAATTAATT
TACTCCTGCTAATTATCCCTGCCTTAATTGCCATAGCATTTTTAACACTCATAGAACGGAAGATCTTGGGCTA
TATACAATTTCGAAAGGGCCCTAACATAGTGGGCCCCTACGGAGTACTTCAACCATTCGCGGATGCAATAAAA
CTTTTTACAAAAGAACCACTACTACCCACCACATCTACTACAACCCTATATGTGATTGCCCCAACCCTAGCCC
TCTCCATCGCCCTCCTTATATGAAGCCCTCTTCCCATACCATATCCCCTAATTAACTTCAACCTAGGCCTCCT
ATTTATATTAGCAACATCAAGCCTAGCCGTCTATTCAATCTTATGGTCCGGATGAGCATCCAACTCAAACTAC
GCACTAATCGGCGCATTACGAGCAGTAGCCCAAACAATTTCATACGAAGTCACCCTAGCTATCATTCTACTAT
CAACCCTACTAATAAGCGGCTCATTCAACTTACATTCACTTATCTCAACACAAGAACACTCCTGACTCTTATT
ACCATCATGACCTTTAGCAATAATATGATATATTTCCACACTAGCAGAAACCAATCGAGCTCCCTTCGACCTA
ACAGAAGGCGAATCAGAACTAGTTTCAGGATTCAACATTGAATATGCCGCAGGTTCATTTGCTCTCTTCTTTA
TAGCAGAATATATAAACATTATTATAATAAACGCCCTAACCACCACCGTCTTCTTAGCTACACCCTATAGTAT
AAACCTACCAGAACTCTATACAATAAACTTTATAACCAAAGCCCTTCTATTAACTATTTTATTTTTATGAATT
CGAACATCATATCCTCGATTCCGCTATGACCAACTAATACACCTTCTATGAAAAAACTTTTTACCCCTCACAC
TAGCACTATGCATATGATATGTCTCAATACCATCTCTAATATCTGGCATCCCACCACAAACATA?ATCAACCC
TCTAGCCCACCTTATTATTTCCCTCACTGTCCTAGCAGGAACCGTAATTACAATATTAAGCTCACACTGATTC
CTAATCTGAGTAGGTCTAGAATTAAATATACTAGCCATCGTACCCTTGCTAGCCAAAAACACAAACCCCCGCT
CCACAGAAGCAGCCACCAAATATTTTCTAACACAAGCAACCGCATCCATACTCCTACTAATAACCATTTACCT
AAATAATCTATTTTCCGGACAATGAACAATTTATCCATCCCTAAATCAAACCCTCGCTACAATAATATTTATT
GCCCTAACAATAAAATTAGGGATAGCCCCTCTTCACTTCTGACTACCAGAAGTAACCCAAGGCATCCCTCTAA
TCCCTGCCATACTTATTCTCACATGACAAAAACTAGCCCCCTTATCTATTATACTCCAAATCTTCCCATCAAT
TGACACCAATACCCTACTAACAATCTCAATCTTATCTATCATAATTGGCAGCTGAGGCGGACTTAACCAAACA
CAATTACGTAAAATTCTAGCTTACTCCTCAATCACCCATATAGGATGAATAATAGCAGTACTACATTACGACC
CAAATATTACAATCCTGACTCTACTCATTTACATCCTTCTAACAATCTCCACATTCATAACCTTTTACCTAAA
CTCAAACATAACAACTCTATCGCTATCACACACCTGAAATAAACTTACATGAGTAATACCTATAATCCCATTA
ATAATGATATCCCTAGGGGGCCTACCCCCACTAACAGGCTTTTCCCCCAAATGAGCAATCATACAAGAACTTA
CAAAAAGTAATAACCTTATTATTCCCCTCACTATAGCCATACTCACGCTAATAAATCTATACTTCTACATACG
CCTGACGTACTCCATCTCAATAACAATATTCCCCACATCCAACAACACAAAAATTAACTGACAACTAAAACAT
ATAAAACTCTCACCACTTCTCCCCCCACTCATAATTTCTTCCACCCTTCTATTACCCTCAACCCCACTAATAC
TAATAACTT????????GGCAGCATAAATAATAAAGCATGTTACTTAAGCAATTTTACATTTACTGTATTAGT
AAGTAAATGCAATGGCCACTCTTATTCTCAAACAATAAAAACTACCCAGCCTTTTCATGAAAGAT-
GATTGCAATTTCCATTGGCCTGTGTATGCAATAAAAAG---
CCATAACTCATCAAAATTTAGCTATTTTCTCTTAAGATA--

GAAGAGCATTTTGCTTGAACATTGAACATGACATTAAATATT-
CATGAAACTGAATAGAGTTTAAGTACTAAG---
TAAAACTCTCAAATTCTTTTCAACTAGATTGAAATTCTGTTTGAACTAGGTAAAAACTACCGCTAAGCTGAAG
AGGTGTTCAAAAAC-
ATCGTAAAGAGATTGTACAATACTTTCAAAGTGCTACAGTTATTTGGGATAGACTTTTCTGAAAAGCCTGTTC
CTCCTTTGAAATTGATTCATGATAACAGCCTCCATTTGGGTCAAACAGTATTTAACCACTTGTAAA-
TTTTAATT-
AAATGATTTGTCACTTTGAACACATAGTTT??
???????AAGGAGTCTTGTGATGGAATGGGAGATGTGAGTGAGAAGCATGGGAGTGGGCCAGTAGTCCCGGAA
AAGGCAGTCCGTTTTTCATTCACAGTCATGAAAATTACTATAGCCCTCAACTCTCAGAACCTGAAAGTGTTTG
AAGAAACCAAACCTAACTCTGAACTGTGTTGCAAGCCATTGTGCCTTATGCTGGCAGATGAGTCTGACCACGA
GACCCTGACTGCCATCCTGAGTCCTCTCATTGCCGAGAGGGAGGCCATGAAGAGCAGTGAATTAATGCTTGAG
ATGGGAGGCATTCTCCGGACTTTCAAGTTCATCTTCAGGGGCACCGGATATGATGAAAAACTTGTACGGGAAG
TGGAAGGCCTTGAGGCTTCTGGCTCAGTCTACATCTGTACTCTTTGTGATGCCACCCGTCTGGAAGCCTCTCA
AAATCTTGTCTTCCACTCTATAACCAGAAGTCATGCCGAGAACCTGGAACGTTATGAGGTCTGGCGTTCCAAC
CCTTACCATGAGTCTGTGGAAGAACTGCGGGATCGGGTGAAAGGGGTCTCAGCCAAACCTTTCATTGAGACAG
TCCCTTCCATAGACGCGCTCCACTGTGACATTGGCAATGCGGCTGAGTTCTACAAGATCTTCCAGCTAGAGAT
AGGGGAGGTGTATAAGAATCCCAGTGCTTCCAAAGAGGAAAGGAAAAGGTGGCAGGCCACATTGGACAAGCAT
CTCCGGAAGAAGATGAACCTCAAACCAATCATGAGGATGAATGGCAACTTTGCCAGGAAGCTCATGACCAAAG
AGACTGTGGATGCGGTTTGTGAGTTAATTCCTTCTGAAGAGAGACACGAGGCTCTGAGGGAGCTGATGGATCT
TTACCTGAAGATGAAACCAGTATGGCGGTCATCATGCCCTGCTAAAGA--GTGCCC-
AGAATCCCTCTGCCAGTACAGTTTCAATTCACAGCGTTTTGCTGAGCTCCTTTCTACCAAGTTCAAGTATAGG
TATGAGGGGAAAATCACCAATTATTTTCACAAAA????????????????CACCCGACAGTTGTCATTATGTT
CGGAAACACTGCCGGCCCATGGAGATGAGGATAAAGTGGACATTTCTCTGCTGCATCAGGGTGAGAATCTTTT
TGAACTGCACATCCACCAGGCCTTCCTGACATCTGCCGCCCTGGCTCAGGCTGGAGACACCCAACCTACCACT
TTCTGCACCTATTCCTTCTATGACTTTGAAACCCATTGTACCCCATTATCTGTGGGGCCACAGCCCCTCTACG
ACTTCACCTCCCAGTATGTGGTGGAGACAGACTCCCTTTTCTTACACTACCTTCAAGGGGCTTCAGCCCGTCT
TGATCTCCACCAGGCTGTGGCCAGTGAGCACAACACTCTTGCTGCAGGATGGATTTGCTTTGACAGGGTGCTA
GAGACTGTGGAGAAAGTCCATGGCTTGGCCACACTGATTGGTAAGTGCTGTCAGCTTCCCGCAGCTCCTA-
GCACCAATGCAGAATTTCCCAAACACTGTAGCTGCTTTTCTGGTCGTTCCTTATTTTCTTCCTTTTTCTTTGT
TCTTGATTCTTGCCACACCATCTGTATTCTCCATGCTTTCA-
TACTTGCTGCTGCCCAGGAGCTGGTGGAGAAGAGTTTGGGGTTCTAGAGTACTGGATGAGGCTGCGTTTCCCA
ATAAAACCCAGCCTACAGGCATGCAATAAACGAAA??????????????AGCACATGCACATTCAAAGGCAGTT
TGGAGTCTGAAAAGCATCAATACATCATCCATGGAGGGAAAACCCCAAACAATGAGCTTTCAGATAAGATTTA
TGTCATGTCTGTTGTTTCCAAGAGCAACAAGAAGGTTACTTTTCGCTGCACAGAGAAAGACTTGGTAGGAGAT
GTTCCTGAAGCCAGATATGGTCATTCCATTAATGTGGTATATAGCCGAGGGAAAAGTATGGGTGTTCTTTTTG
GAGGACGTTCATACATGCCTTCTACCCACAGAACCACAGAAAAATGGAATAGTGTAGCTGACTGCCTGCCCCA
TGTTTTCCTGGTGGATTTTGAATTTGGGTGTGCTACATCATACATTCTTCCAGAACTTCAGGATGGGCTATCT
TTTCATGTCTCTATTGCCAAAAATGACACCATCTATATTTTAGGAGGACATTCACTTGCCAATAATATCCGCC
CTGCCAACCTGTACAGAATAAGGGTCGACCTTCCTCTGGGTAGCCCAGCTGT-
AAATTGCACAGTCTTGCCAGGAGGAATCTCTGTCTCCAGTGCAATCCTGACTCAAACTAACAATGATGAATTT
GTTATTGTTGGTGGATATCAGCTTGAAAATCAAAAAAGAATGATTTGCAACATCATCTCTTTAGAGGAAAACA
GGATAGAAATTCGTGAGATGGAGACCCCAGATTGACCCAGACATA?????CGTGTCTCAGCATCTTCCTGCTC
TCCACCACACTGAGCACACGATGATCCAGTGCTCCACAAACATATGTAGAATGGAGGTAAAACCTCAGGGATA
AGAAAAGATAATTGAAGGGCCGGGCGCAGTGGTTCATGCCTGTAATCCCAGCACT-
TAGGAGGCCGAGGTGAGTGGATCACAAGGTCAGGAGTTCAAGACCAGCCTGGCCAAGATGGTGAAAACTCATC
TCTACTAAAAATA---------------------------------------CAACAATT-----
AGCTTGG---
--------------------CAGGGTGGTGGGCACCTGTAATCCCAGCTACTTGGGAGGCTGAAGCAG-
TGAATTGCTTCAAC-
TGGGAGGCAGAGGTTGCAGTGAGCCGAGATCATGCCACTGCACTCCAGCCTGGACAACA-
GAGTGAGACTCCATCTCAA---

---AAAA-

TAATAATAAAAAGAAAAGATAGTTAAAGCAGATTCACCACAGTAACAAACCAGCCAAGAAACTGCACTGAAAG
TAAAGGTTT-
GGGTGACAAAATTTCCAACTGTGTTCAAAGAAACATAAGTTCCAGCAGGAGTGCACGTAATCTGATTTT-
CTTCAAAGC--
CTGTCTAATTTGTAGGGAAGGACATCCATTTTCATTAGCTTCATGTGAGCTAGAGAGAATCCATGATTGAAGC
ACTAAAATCCCTGGTAAACATGCCAGCAAGCAAGGGCACCTTCCCCAGAGGCTCAACCTAGAATATAGGACTG
TCGGCATTGGTAAGGAGCCTTGAACTCTAAACCCATGGGCTCAAGTCAGGAAGCTCAGGAAGCCTATCAACAC
CGGGA??
???
???
???
???
???
?????????????????????CAAAGATCTTTATTGCCATAAAGCAGAGTAACT-
GTCTTTGACTTGGACCTTGTTTACTGTGAACTCTAATC-AAGGCAGGACATTCAGCAGTTCTACTTA-
CTGCACTGTGGACTTGTGGAAAGGACACTTGTGA---------
ATCCAGTTTGGATTCGTGTTTGAAATTGAATTGCGAATGGTGTTCCAGGATGTGGAATACAGCTGTGATCACA
CTTGCCTACC-
GAGCTTACTTTGATCTGTTTGTCAATAGCATGCAAAAAAATGCTTTGTCTGCCCTTTGCTTCTGCTTTTTTCA
GGGAAGCTGCCAAAGAATGTCGACGTCGAAAGAAAGAATACGTAAAATGTCTGGAGAGCCGAGTTGCAGTGCT
GGAAGTCCAGAACAAGAAACTGATAGCGGAACTTGAAACCTT???????????????????????????????
???
???
???
???
???
???
???
???
???
??????????????????????????ACGTGTGGATTCCAGCACCTAAACCAAAAAATGCCACTGTAATGATA
TGGCACTCATGGTGGTGGTTTTCAAACTGGAACATCATCCTTACATGTTTATGATGGCAAGTTTCTTGCTCGG
GTCGAAAGAGTTATTGTAGTGTCAATGAACTACAGGGTGGGTGCCCTAGGATTCTTAGCTTTGCCAGGAAATC
CTGAGGCTCCAGGAAACATGGGTTTATTTGATCAACGGTTGGCTCTTCAGTGGGTTCAAAAAAATATAGCAGC
CTTTGGTGGAAATCCTAAAAGTGTGACTCTTTTTGGAGAAAGTGCAGGAGCAGCTTCAGTTAGCCTGCATTTG
CTTTCTCCTGGAAGCCATTCCTTGTTCACCAGAGCCATTCTGCAAAGTGGATCCTCTAATGCTCCCTGGGCAG
TAACATCTCTTTATGAAGATAGGAACAGAACGTTGGCCTTAGCTAAATTTACTGGTTGCTCTAGAGAAA-
TGAGACTGAAATAGTCAAATGTCTTCAAAACAAAGATCCCCAAGAAATTCTTCTGAATGAAGCATTTGTTGTC
CCCTATGGAACTCTCTTGTCAGTAAACTTTGGTCCAACAGTGGATGGTGATTTTCTCACTGATATGCCAGACA
TATTACTTGAACTTGGACAATTTAAAAAAACCGAGATTTTGGTGGGTGTTAATAAAGATGAAGGGACAGCTTT
TTTAGTCTATGGTGCTCCTGGCTTCAGCAAAGATAACAATAGTATCATAACTAGAAAAGAATTTCAGGAAGGT
TTAAAAATATTTTTTCCAGGAGTGAGTGAGTTTGGAAAGGAATCCATCCTTTTTCATTACACAGACTGGGTAG
ATGATCAGAGACCTGAAAACTACCGTGAGGCCTTGGATGATGTTGTTGGGGATTACAATATTATATGCCCGGC
??TTGTGCT
AATTGTGAGAAAATATGCTACTTTGATTAAACTAGAATGAAGGAGATAAAAAGACACCGTGGCTCAACTTGTA
ATAAAGAGCAGCTCATCCTCACAGCTTTTTGATTGTTGCTATAGTTATTT---
ATTCAACATGGGATGCACAGTACAGTTAACGTTTCATTACCAGTAGCCTGTTGCGTGCTTATTGTTAAAAGGA
ATTTAACATACAAGTTTGTGTATGGATTGTTCGCCTAATTCCGATTCCTCGTGCTGACTGGCATGAGACAGGG
AGCTGGATTGTATTAAACTGGTTTTATTGTGATGTGACTATGATGGATGTGACGAGGGCCTCTATTATTCGCT
GGGGAGTGTGGCCGTTAATGAAGCCATTAGCTGAGCTAAGGCAAGTGAAGAAAATCGGGAAATTACCATTATT
CTGAAGCCATTAAGTTGTAATCCGCCACTTTCTCTCCTTATTCAAAAAGGCATCTTCTTTTTAA?????????
?????????ATGGTTATTTCATACTTCGGTTGCATGAAGGCTGCCCCCATGAAAGAAGCAAACGTCCGAGGAC
AAGGTGGCTTGGCCTACCCAGGTGTGCGGACCCATGGGACTCTGGAGAGCGTGAATGGGCCCAAGGCAGGTTC
AAGAGGCCTGACGTCATCGTTGGCTGACACTTTCGAACATGTGATAGAAGAGCTCTTGGATGAGGACCAGAAA

GTTCGGCCCCATGAAGAAAACAATAAGGACGCAGACTTGTACACCTCCAGGGTGATGCTCAGTAGTCAAGTGC
CTTTGGAGCCTCCTCTTCTCTTTCTGCTGGAGGAATACAAAAATTACCTGGATGCTGCAAACATGTCCATGAG
GGTCCGGCGCCACTCTGACCCTGCCCGCCGAGGGGAGCTGAGCGTGTGTGACAGTATTAGTGAGTGGGTAACG
GCGGCAGACAAAAAGACTGCAGTGGACATGTCGGGCGGGACGGTCACAGTCCTTGAAAAGGTCCCTGTATCGA
AAGGCCAACTGAAGCAATACTTCTACGAGACCAAGTGCAATCCCATGGGTTACACAAAAGAAGG?????????
??AGC-
GATGCAGAATTAGACCC---
GCCACAGCAGCCTCTGAAGTTGGACAGCAAAACCATTGCTTCACTACCCGTCGGTGTCCATTTATAGAATAAT
GTGGAAAGAAACAAACCCCTCTGTCTTATGATTTATTCATTATCGCCTTTTGACAGCTGTGCTGTAACACAAG
TAGATGCCTGAACTTGAATTAATC-ACAGATCAGTAATGTATTCTCTCTCTCTCTCTCTCTCTCTCT------
--------ACATTT-GGTCTCTACATACATTTATTAATGGGTTTTGTGTACTGTAAAGAATTTAGCTGTTTC-
AAACCTAGTGCATGA-
ATAGATTCTCTCCTGATTATTTATCACATAGCCCCTTAGCCAGTTGTATATTATTCTTGTGGTTTGTGACCCA
ACTAAGTCCTACTTTAAATATGCTTTAA-GAATCGATGGGGGA---
TGCTTCACGTGAACGTGGGAGTTTAGCTGCTTCTCTTGCCTAAGTATTCCTTTCCT-
GATCACTATGCATTTTAAAGTTAAA-CATTTTTAAGTATTTCAGATGCTTTAGAGAGATTTTTTTCC-
CCATGGTTGCATTTTACTGTACAGATTGCTGCTTCTGCTCTA????????????????????????????????
???GGAGAGCACTAAGCCACTTCCAGCCCTGCTGT
GAACCAGGACTGTAGGAGCCATCTTTTTCATGTGGTCAGTTTTGGAAGCATCTACATCCACCACTTTAGAAGA
CAAGTCTAGTGGCTTATCTGTGACGTCTTTGGTAACGGTCTGCTTCTCCAACAGAGGAGGTGAGCTGCCGTCT
TTTCTGTCTTGAACTGCTGTCTTCCGGGCATGCCCGGGCACTGGCTGGGCACCTTCGCCCCCTTCCAGAGCCT
TGGGATACTTGCCGTTGGAGAGCCTGGCTGCGGGGAACTCGCTGCTGACTGTCATGTATGGCTTCGACAGGGC
AACTGAAGGAGAGGTGGAGATCCTGGCATAGTGCTTATGGAACTCCGAGTAGGTATCTGCAGCAGGCTGGGTG
GGAAGGTGGACCCGGGGTGAGGGCCGAGGCGAGGGGGGCAACAGGAGAGCTGTGTCCCCCGGCAAGCCACTGG
TGACTGCCTTGGCAGAGGGAACCCTGGGCTGTTTACTGTTCTGGATGTGAGGATAGGCATGGGAATCAACGGG
ATTCCCAGGGCTGACCCCCATCTTCCACGGGAGGCTTTTGTCTGCGCAATGGACAAGAGGCGGGATGGCTGGG
GAGGCCGAAGGCGTCGAGAGCCTCATGGGTGATGTTAAGGACGATGGGATGTGGGGACTGACGTAGTGAGGTG
GTGGCAGGTAGAGAAAGCGCTCCCCATTGGTGCAGACTGGAGAATACAGCGGCTGGGCCAAGCTGTAGGACTG
CTG???????????TGCCAGGACCTTTGTAATTCAAGTGCTTTTTACAGTAGTAGCAAATGTTATATAAGCCC
AAACATGGAGCA---
TATTTGGAGTTGGTGGGCAGACCCAAATCATGATGCAAGCCTGCAGTAGCAAGTGTGCAATGAAGCACAACCA
GATTCTGTGAATGGAAGGTAGCGAGACGCCTCTCCAAAGCAGCCCTTCTGCGCAGGAGCTGAAAGCTCCACTG
GGTAAGTTTTCTGAGGCAGTTTTCAGAGGCTGGGCTTGGCTCAGGGCCCGGCTCTGGATGAGCCTGTGGACGG
TGGGGTGGGTAGGTCTTTGCTAACCTTACATAGAACCAAGCAGAGTTTGGAAGAGTTTGTGCAGGCCTCTTTC
CTCCTGGCGTTTGTTTTGCCCTGCCAGCGGGCCAGGAGGG-
CTGCGCTGCTCAAGTAGTGGAGGGAGGGAGCCTGCAGAGCTAGGAGATCAGAGTGGGTAATCGCAGCCACAGG
GATTAGCTGCTGCAAGCTGTCCACAGGAGCCATTCTGAAATGTCATTTGAGTGATCTGGC?????????????
????????????CAGCTGTCTCCAGGAGCAGCTTCTGAGGGAGTCTTCCAGGAGGAGCAGTGTCTCCGCCATC
GTCCTCGTCCACGTCCTGGTCACATTTCCATTCCTCATCTTCGTTTAAAGTCGGCAGGTATCCAGATATGCCC
TTCCCTGTCCCTGACCCAGAGCTCTGGTCACTACAAACCTTCGGGCTCTCCGAGCCTGTCCTCGTATATTCCA
AATAAATATCAGACTTAAGGAAGGAGGGGTAGGTGTTTTCCTCCATAGTGGCCTGGATTTCTGTCTGGGCCTG
GTCAAACATGGCAGGATCGATCAGCTGCTTCATGATGCAGCCCTTTATGAAGCTCTTGGTGGCCGGCTTGGTC
TGCCGGGACACGATGCCATTGTTATCAAGGATGTACTTTCGGTAGATGGCTCTGGCCAGCTTCAACCTCTTCT
CCTCATTCGAGTCACASGGCTCCAGCTTCCTGAAGCCAGTGCAGGCAAACCAGAAGTCCAGCAAGTCAGCACA
GCCCTCCTGCTTCAGGAAAGTCCTGAACAGGCTTATCCCATCTTGGTCATCCAGCAGCGAGTGCAGTGACTCA
GCCCACTTCAAGTACGGTGGGGTGGGGGAGGCACTGCCCTCGGGCTCATACCCCAGGTCCAGATCTGAGCGTC
TCGGAGTGGCGGTCGAAGTCTCACCTTTAATGCCAACACCTTTCCCGGAGCAGAAACTGTAGCTGGCGGGCCT
CGGGTCTGTGGACACCAGTTCTCCCTCCTCACCAGGCACTGGGGGTCGGGGAGCATCTTCGGTGAAACTTGCT
CCAAGGTCCAAGGGGAAACCCTGCTCCTGGACATTCATTTGGGACTCTGTGCGTCAATGCACAATGAGCGCT
GCACCCTAATACATCAGTACTTAACAGCTCCAAAGTCAATCAATCTGTCCTGTTGAAACCAT????????AAA
TGTTAGCTAATTTTCATTGAACAA-A--
ATTACATGGATAAACTTATGACAGCGTGAAACAGGTATAATTTACAGTGGAGCAGGATAAGT-
ACTTAGGTTATTTATAGGACAAGTTATTTTTCTGTGTTGTTTTG----CTTCTTTG-
CATATTAAAATTTATTTATAGGTGTAGTTAAGCTTT--ACTATTAGTTAGCATAAATTTGGCTCGGAGTCA--

--
CCAGAGCAGAAATGTTGGAGTTCATTTTTATGAGTTATTTTCTACACATTTCTAAATTGATGTAGCTTTGTCA
CTGCTTGATTGGTTTTTTTT---
AAACTGGAGGGGGTGAAATTAGTTTATACTGAATCAAACCAGTTGCTGTTAATTACTAACACTGAAAATGTTA
ATTACATTACTAACACTGAAAAGAAGTAAAAAGAATGTTAATAACTTAGGAGTATTTGGTTAGTTA-
TGTTCAGAACACAAGCTTTAAATTAAT-GAGGAAACCA---------
GAAGTTTGATTTAAGCACTCATACTCCTTTATTTTCCTTTGTTAGCAATAATTATTGGCCCTGATGGCCATCC
TTTGACTGTTTATCCTTGTATGATTTGTGGGAAGAAGTTTAAGTCGAGAGGTTTTTTGAAAAGACACATGAAA
AACCATCCCGAACATCTTGCCAAGAAGAAGTACCACTGTACTGACTGTCATTACACTACCAACAAGAAGATAA
GTTTACACAACCACCTGGAAAGCCACAAACTGACCAGCAAGGCAGAGAAGGCCATTGAATGTGATGAGTGTGG
GAAGCATTTTTCTCATGCAGGGGCTTTGTGTACTCACAAAATGGTGCACAAAGAAAAAGGAGCCAACAAAATG
CACAAGTGTAAATTCTGTGAATACGAGACAGCTGAACCAGG?????????????????????????????????
????????????????????????????????TCTGCAAACAGTAAAGATACCACTAAAACCCCTTCTGCAGT
TCAAACTAGCACCAGCCACAACCCTGGACTTCCTCCTAATTTTAACGAATGGTACGTCTGAGGACAAACACAA
ACCCTGTTAACTATAGAATGGACCAAATGCATTTTTAGAAGAAAACTGAGACCAATCAGATGGAAATGGAGTT
TTAAGGCAAGAGGCCATATATAGGGCTACATCTTGTTAATTGCAATTGTCCAGGAAGGTTTTGGGCAATATCC
AAAAGTAGCCATGCCCTTTTCTCAGGATTAGAAAATATGTTTTGGTGTTTGAAGGATTTTTT--
ACAAAATCTTTACACTGCTTTTTCTTCCCCTTCCTCTTGCTCTCTGCATACCCCATTCTTAAACTCCTGCAAT
TCATTTTAACACTTGTCCTGTTTCTTGAGAGGAAGTTATAGAAGGCTTGTTGGTGGTGGTGATGTTAAACTGA
TGGAAATTCTTTTTCGCCTTAGTG???
?
Aotus
TTACCCCCATGTTTGGCTGGAACATGAAGCTGACCTCAGAGTACTACAGAAATGTCACCTTCCTTTCATGCCG
GTTCCCTT-
CCGTCATGAGCATGGACTACATGGTGTATTTCAGCTTCTTCACCTGGATTTTCATTCCCCTGGTCATCATGTG
TGC--GATCTATCTCGACATCTTTTACAT-
CATCCGGAACAAACTCAGACAGAACTTCTCTGACTCCAAAGAGACAGGTGCATT---
TTATGGACGGGAGTTCAAGACGGCGAAGTCCCTGTTTCTGGTGCTTTTCTTGTTTGCTTTGTCATGGCTGCCT
TTGTCCATCATCAACTGCATCACCTACTTTCA-------------
TGGTGAGGTACCACAGCTTGTGCTGTACTTGGGCATCCTGCTGTCCCATGCCAACTCCATGATGAACCCTATC
?????????????????????ATGTTCATAAACCGCTGATTATTTTCAACCAACCATAAAGACATCGGAACAC
TATATCTATTATTTGGTGCTTGAGCGGGGGCAGTGGGAACAGCCCTAAGCCTTTTAATTCGAGCAGAATTAGG
TCAACCAGGAAGTCTGATGGAAGATGATCATGTATACAACGTTATTGTTACTGCCCACGCATTCATTATAATT
TTCTTTATAGT---AATACCGATTATAATCGGGGGATTTGG---
AAACTGACTAGTTCCCCTAATAATTGGTGCCCCAGACATAGCATTCCCCCGAATAAATAATATAAGCTTCTGA
CTTCTACCCCCA--TCTCTACTGCTCTTACTTG-
CATCCTCAACTCTAGAAGCCGGCGCAGGAACCGGCTGAACAGTCTATCCACCCTTAGCAGGAAATATATCACA
CCCAGGAGCCTCTGTAGATCTAACTATTTTCTCACTTCATCTGGCAGGTATTTCTTCAATTCTAGGAGCCATT
AACTTTATTACAACAATCATTAATATAAAGCCCCCAGCCATGACCCAATACCAAACACCCCTTTTTGTCTGAT
CCGTCCTTATCACAGCAGTCCTCTTACTCCTCTCTCTTCCAGTCCTAGCCGCCGGAATTACTATACTGCTCAC
CGACC--
GTAACCTTAATACTACTTTCTTTGATCCTGCCGGTGGCGGCGACCCTATTCTATATCAACACCTATTTTGATT
CTTCGGACACCCTGAAGTATATATTCTTATTCTACCGGGCTTTGGAATAATTTCACATATCGTAACATATTAC
TCCAACAAAAAAGAACCATTTGGGTATATAGGAATAGTCTGAGCTATAATATCCATTGGTTTCCTAGGATTTA
TTGTATGAGCCCATCACATATT-
TACAGTAGGAATAGATGTTGATACACGTGCATATTTTACATCAGCCACTATAATCATTGCTATCCCTACCGGA
GTAAAGGTTTTCAGCTGACTAGCTACGCTGCATGGTGGCAACATTAAATGGTCTCCCGCAATACTATGGGCCC
TAGGCTTTATTTTTCTCTTCACTGTGGGTGGACTAACAGGAATTGTGCTAGCCAACTCATCATTAGATAT-
TGTTCTACATGATACATATTATGTAGTAGCCCATTTCCACTACGTCTTATCCATAGGAGCAG-
TATTCGCTATCATGGGTGGCTTTATTCACTGATTCCCATTATTCTCAGGCTATACACTTAACCAAACCTATGC
CAAAATTCACTT-
TACCATTATATTCGTAGGTGTAAACTTAACCTTCTTCCCGCAACATTTCCTTGGTCTATCCGGAATACCTCGA
CGATACTCAGACTATCCCGACGCCTATACCACATGAAATATTATTTCATCTGTAGGCTCATTCATCTCTCTAA
CAGCAGTAATCTTAATGATCTTTATAATTTGAGAAGCTTTCTCCTCAAAACGAAAAGTTTCAACTATTGAACA

ACTATCTACTAATCTAGAGTGACTTTACGGCTGCCCTCCTCCTTATCACACATTTGAAGAGTCCACTTATGTA
AAAACTCTAGCC????????????????????????ATGGCAACACCAGCTCAACTAGGCCTACAAAACGCTA
CGTCTCCTATTATAGAAGAACTTATCGCTTTTCACGACCATGCCCTTATAATTATTTTCTTAATCAGCTCACT
AGTCCTATATATTATCTCCCTAATACTTACTACAAAATTAACCCACACCAGCACCATAAACGCCCAGGAAATC
GAAATAATCTGAACTATTCTGCCCGCTATCATTCTTATTATAATTGCTCTCCCATCCCTACGTATCCTATATA
TAACAGACGAATTTAATAAACCCTACCTTACCCTTAAAGCAATTGGCCATCAATGATACTGAAGCTATGAATA
CTCAGACTATGAAGATCTAGCATTTGATTCCTATATTACGCCAACCTACTTCCTTGAACCTGGCGAATTTCGA
CTTCTCGAAGTAGATAACCGGACAACCCTCCCAATAGAAGCAGATATTCGTATACTAATTACATCACAAGACG
TCTTACACTCATGAGCTGTTCCATCGTTAGGTGTTAAAACAGACGCAATCCCCGGACGCTTAAATCAAGCTAT
ACTGGCCTCTATACGCCCAGGCCTATTCTATGGACAGTGCTCAGAAATCTGCGGATCAAACCATAGCTTCATA
CCTATCGTTCTAGAATTTATCTACTTCCAAGACTTCGAAGTATGAGCTTCATA????????????????????
ATGACTTCTCCCCGCAAAACACACCCACTAACAAAGATCATTAACGAATCATTCATTGATCTACCCACACCAC
CCAACATTTCCTCCTGATGAAATTTTGGCTCACTCTTAGGCATTTGCCTAATTATTCAAATCACCACCGGCCT
GTTCTTAGCTATACACTACACACCAGATACCTCAACCGCCTTCTCCTCCGTCGCCCATATCACCCGAGACGTC
AACTATGGCTGAATAATTCGCTACATACATGCCAACGGTGCTTCCATATTCTTCGTATGCCTTTTTCTCCACA
TTGGTCGAGGACTTTACTATGGATCCTTCCTTTCTCTGAAGACTTGAAATATCGGTACCATCCTACTACTTAC
AACCATAGCCACAGCATTCATAGGCTATGTTCTTCCATGAGGCCAAATATCATTCTGAGGGGCTACAGTAATT
ACAAATCTTATATCAGCCATTCCCTATATCGGATCTGACCTTGTACAATGAATTTGAGGTGGCTTCTCAGTAG
ATAAAGCCACTCTCACACGATTCTTTACTTTTCACTTTATCTTACCCTTCATTATCGCAGCCCTAGCAACTAT
CCATCTATTATTTCTGCATGAAACAGGATCAAGTAACCCATCAGGAATAACATCTGACCCCGACAAAATCACA
TTTCACCCCTATTATACAGCTAAAGACATTCTAGGGTTAATCTTTCTTCTCTTATCCCTAATAAGCCTAACCC
TATTTATACCCGACCTTTTAACCGACCCAGATAATTATACACTGGCTAATCCCCTCAACACTCCACCCCACAT
CAAGCCAGAGTGATATTTTCTATTTGCATACGCAATCCTACGATCTATCCCTAATAAACTTGGAGGAGTTCTA
GCCCTAGTACTTTCTATTTTAATTCTAATAGTTATCCCTATACTACATCTCTCCAAACAACAAAGCATAATAT
TTCGACCCATCACTCAAATTCTATTCTGAACTCTAGTAGCTGACCTACTAACTCTCACATGAATTGGAGGCCA
ACCGGTTGAATACCCCTTCGTAACCATTGGCCAAACCGCATCCATTACATACTTCTTCATTATTATTATCCTA
ATGCCCCTTTCCGCCTCAATCGAAAATATATTACTTAAATGATAA?TTAGGTTAAATTTGTTCAAGGGAGTGA
GAATTTATGGGTATAACTGTTAGCTGGAAAGCCCTTCCTCACCTCCAATCCTCCTTTCAAACTACATTTCC-
TCCTGAAATTTCTCTGTGGGATGAAATGTTCAATTATTAGTCAATCAATC----
TCTTATACATTATCTGGCTTTACATAT----
GCCCTTGATAGTTCTTGGAAAATAATCCAATATTGTGTTGTTTTGGCGTCTTTGTATTGGAAATGGAAGCATT
TTGTATGAATCACCTCTTTAACTGTGAGGAGTTGAATTGTCTTAGTTCTGCTTGCCTAATGACTGGGAATAAA
--GACATCACAGTGTCTAACCCCACTTTTT-
GGTGGAAAGAAAATCTAAAAGTAGCAACATTTAATGGAACAAAATATCAATAATCAAAGAAACTCCCCTGTGA
AACTGTAATATTATTTT-
AAAATCAACATTGTTCTAAGGTAGCTGAAATGTGTGATTTTTCTATTTTGCCTTTTCACTGACAATATACCCA
TAAATCTTTGGTGGTGATTTTCTCTCCAAAGTTAATTTGTGGGCCATTAGTGACTGCACTATGTATTGTTCTA
AATTGTTGTCTTAATTGCATTCTTTAAAAATGTAATGTTGAAGGCACATAATATTGAAGATGACTCCAGGATG
AACCCTGAGTTTGCAGACCGAA-
TAAAACAGCTCGATAAAGAGGCATCTTACTACCGTGATGAGTGTGGCAAGGCCCAAGCGGAAGTGGACCGGTT
GCTGGAGA??????????GAAGAGCTGCATCAGGCAAGTACTTTGATCTTTCTTGGAGCAGGTTCCTTCAAAA
CGCTTCTCCAGAGGACGGAAGTCCCTCCAGTCTGGCTCGCTGCTTGCAGTGGTGGGGAAAGCTGAGGTAATTC
CCCGGTGGGAAATCTGTGAGGCCCCTGATACCCCTG--------
CCACCAGGCCCATCACTGATGGGCCTAAGGGCCTCATCTGATACTCTAAGACGGGCTTCTCCTGTCGGATCTG
GGCCTCTCGGACTTGAGCCTCTCTAGAACGAGCCTCTCGGGCCTGGACTTCTCGGGTTCTGGCCTCCCTGGCA
TGACCTTCCCAAGTGTGGGCCTCCCTG--
GCATAGGCTTCCTTGCCATGAGCCTCTCGAGTATAGGCCTCCCTGGTGTGGGCCTCCCTGGCCTGAGTTTCTC
GGGCACGTGCCTCCCGGGCCTCAAGCTGCTCCCGCCGAAGCTCCCAATACAACAACTGTTTCTGGATGGTCA-
CTAGCCGTTCCTCCTCTGTCTCCATTGCCCCAGGTGGCCGGGAGGAAGAAAAGGGCTCAAAGTTCAAGAAGGG
GTCAAACATCTCAGAGCTTCGACCATGGAGGTCATAAAGGCAGTCATCCCCAGGTGGAGAGTTCTCAAGACTG
TCATCTGGCTCATAGAACTCATATAGGGCATCTCCACTGTAGCTGTCTCGGGGCAGACAATCCCTGCGGACAA
GCCCCA??????????????????????????????AGCATCACTAATTTTGATGAACTAAAAATGGTTCCTG
AAGGTCAGATTCATGTACAGTGAGTGTTCTCCACTGTTGTGGTGGGCATGTTTCATGTGTGGGGATGTTGGTG
TGCAAGTTTCGTGTGTTTGGAAAACACTAGCTAGAACATTTGAAAAATTTCATTAACCCAGCTCTGGAAGGTC

ATCTATGAATTCTTCCTATTCCAGTGGCCTTTT-
AAGTGTAGATGTAGATACACTGTAATTTAAAACAGATGGGCATGTCTGAATATTTTCATATGCAAAATGGGAC
AGATGTATATGAATGGATAAATTATCCTTTTAAAAAGATACTTTGTAGCACGAAGTATCACCAATGATTGGAA
GAGTGAACCTGCGTTCAGTCTAGGGCAAACACCAGGAAGACATTATCAAAACATCTAA-
CTATCTACAAACTAATTCCAATTAGTCTACTTTGTCTTCATACAGAAAAAA-CTTTTTTGATTTTTAAA---
TATGTATTGGGTGCCAATCCGTATCCTTT-
GAGCTGCTAAAGCCAGCATATCACAATAGAAGTTTACTTTTCCTTGAGAGTTGCAT?GTGTTCATAATTAATT
TACTTCTACTAGTCATCCCCGCTCTAATTGCCATAGCATTTCTAACACTTATAGAACGAAAAATTTTAGGCTA
CATACAATTTCGCAAAGGTCCTAACATCGTAGGCCCCTTACGGTTTACTTCAACCAATCGCAGATGCAATAAAA
CTCTTTACAAAAGAACCCCTACTTCCCACTACATCTGCCTCAACACTATATGTAACTGCCCCAACCCTAGCTC
TTTCCATTGCACTCCTCATATGAACCCCTCTCCCTATACCACACCCTTTAATTAACTTTAATTTAGGCCTTCT
ATTTATACTAGCCACGTCAAGCCTAGCCGTATATTCAATTCTATGATCTGGATGAGCATCCAACTCAAACTAT
GCACTCATCGGAGCACTACGAGCCGTAGCCCAAACAATCTCATACGAAGTTACCCTAGCCATCATTCTACTAT
CTGTCCTACTAATAAGCGGCTCATTCAACCTACACTCACTCATTGTTACACAAGAACACTCCTGACTACTACT
TCCATCATGACCCCTAGCCATAATATGATTTATTTCTACATTAGCAGAAACCAATCGAGCCCCCTTTGATTTA
ACAGAAGGCGAATCAGAACTAGTTTCAGGCTTCAACATTGAATATGCTGCAGGCTCATTCGCCCTATTTTTA
TAGCAGAATATATAAATATTATTATAATAAATGCCCTAACCACCACTATTTTCCTAGCAACATCCCACAACAT
TACTATACCAGAACTTTACACAATTAACTTTGTAGCCAAAACCGTTCTATTAACCTCCCTATTTCTATGAATC
CGAACAGCATACCCCCGATTCCGATACGACCAACTAATACATCTTCTATGAAAAAAATTTTTACCACTTACAT
TAGCACTATGCATGTGATATATTTCAATACCCATCCTAATATCTGGCATTCCACCCCAAACAT??ATTAATCC
TCTAGCCTACCTCGTTATTTCCTTTACTATCCTAATGGGAACCTTAATCACAACTCTAAGCTCACATTGATTT
CTGATTTGAATAGGCCTAGAATTAAATATACTAGCTATTGTACCAATTCTAGCCAAAAACACAAGTCCCCGCT
CCACAGAAGCATCCACTAAATATTTCTTAATGCAAGCAACCGCATCCATAATTCTCCTAATAGCTATCTTCCT
CAATACCCTATTTTCCGGACAATGAACAATTAACCCATCCCTTAACAAAATTTTGTCCACAATAATATTAATT
GCCCTAGTAATAAAACTAGGAATAGCCCCCCTTCACTTCTGACTTCCAGAAGTAACTCAAGGCATTCCTCTAA
TACCCGCTATACTTATTCTTACATGACAAAAATTAGCCCCCATATCAATTATTCTTCAAATCTTCCCACCGAT
AAACCTAAATACTCTATTAATAATCTCAATCTTATCAATTATAGCCGGCAGCTGAGGGGGACTTAATCAAACA
CAGCTACGCAAGATCATAGCCTACTCATCAATTACCCACATAGGATGAATAATGGCAGTACTATACTATGACC
CAAACATTACTATTCTAAGCTTAATCATTTACATTTTCCTAACAATCTCCACCTTTATAATATTTTACATAAA
TTCAAACCTAACAACCCTCTCACTATCACACTCCTGAAATAAACTCACATGAATAATACCCATAATTCCACTA
ATAATAATATCCTTAGGAGGTCTACCCCCACTAACAGGTTTCTCTCCCAAATGAGCTATCATACAAGAACTTA
CAAAAAACGACAACCTCATTTTTCCCCTCACCATAGCAATACTAACATTAATAAATTTATATTTCTACATACG
ACTAACATACTCCATTTCAATAACAATGTTTCCCTCATCCAATAACACAAAAATCAACTGACAACTAAAATAT
ATAAAATTAACACCACTACTGTCTCCTCTCATAGTATCCTCTACCTTCCTTCTACCCGTAACACCATTAATAC
TAATAGTAT?????GTTGGCAGCATAAATAATAAAGCATGTTACTTAAGCAATTTTACATTTACTGTATTAGT
AAGTAAATGCAATGGCCACTCTTATTCTCAAACAATAAAAACTACCCAGCCTTTTCATGAAAGAT-
GATTGCAATTTCCATTGGCCTGTATATGCAATAAAAA----
CCATAACTCATCAAAATTTTAGCTATTTTCTCTTAAGATA--
GAAGAGCGTTTTGCTTGAACATTGAACGTGACATTAAATATT-
CATGAAACTAAATAGAGTTTAAGTACCAAG---
TAAAACTCTCAAATTCTTTTCAACTAGATTGAAATTCTGTTTGAACCAGGTAAAAACTACCGCTAACCTGAAG
AGGTGTTCAAAAAC-
ATCGTAAAGAGATTGTACAATACATTCAAAGTGCTACAGTTATTTGGGATAGATTTTTCTGAAAAGCCTGTTC
CTCCTTTGAAATTGATTCATGATAACAGCCTCCATTTGGGTCAAACAGTATTTAACCGCTTGTAAA-
TTTTAATT-
AAATGATTTGTCACTTTGAACACATAGTTTGTCCCCAAGATCTTGAGGATTACTTGAATGGCCCCTTCACTGT
GGTTGTGAAGGAGTCTTGTGATGGAATGGGAGATGTGAGTGAGAAGCATGGGAGTGGCCAGTAGTCCCGGAA
AAGGCAGTCCGTTTTTCATTCACAATCATGAAAATTACTATAGTCCACAACTCTCAGAACATGAAAGTGTTTG
AAGAAGCCAAACCTAACTCTGAATTATGTTGCAAGCCATTGTGCCTTATGCTGGCAGATGAATCTGACCACGA
GACCCTGACTGCCATCCTGAGTCCTCTCATTGCCGAGAGGGAGGCCATGAAGAGCAGTGAATTAATGCTTGAG
ATGGGAGGCATTCTCCGGACTTTCAAGTTCACCTTCAGGGGCACTGGATATGATGAAAAACTTGTGCGGGAAG
TGGAAGGCCTTGAGGCTTCTGGCTCAGTCTACATCTGTACTCTTTGTGATGCCACCCGTCTGGAAGCCTCTCA
AAATCTTGTCTTCCACTCTATAACCAGAAGCCATGCCGAGAACCTGGAACGTTATGAGGTCTGGCGTTCCAAC
CCTTACCATGAGTCTGTGGAAGAACTGCGGGATCGGGTGAAAGGGGTCTCAGCCAAACCTTTCATTGAGACAG

TCCCTTCCATAGACGCACTCCACTGTGACATTGGCAATGCGGCTGAGTTCTACAAGATCTTCCAGCTAGAGAT
AGGGGAGGTGTATAAGAATCCCAATGCTTCCAAAGAGGAAAGGAAAAGGTGGCAGGCCGCATTGGACAAGCAT
CTCCGGAAGAAGATGAACCTCAAACCAATCATGAGGATGAATGGCAACTTTGCCAGGAAGCTCATGACCAAAG
AGACTGTGGATGCAGTTTGTGAGTTAATTCCTTCTGAGGAGAGGCACGAGGCTCTGAGGGAGCTGATGGATCT
TTACCTGAAGATGAAACCAGTATGGCGGTCATCATGCCCTGCTAAAGA--GTGCCC-
AGAATCCCTCTGCCAGTACAGTTTCAATTCACAGCGTTTTGCTGAGCTCCTTTCTACCAAGTTCAAGTATAGG
TATGAGGGGAAAATCACCAATTATTTTCACAAAATAGATGTTGCTTATGGCACCCGACAGTTGTCATTATGTT
TGGAAACACTGCCGGCCCATGGAGATGAGGATAAAGTGGACATTTCTCTGCTGCATCAGGGTGAGAATCTTTT
CGAACTGCACATCCACCAGGCCTTCCTGACATCTACCGCCCTGGCTCAGGCTGGAGACACCCAACCTACCACT
TTCTGTACCTATTCCTTCTATGACTTTGAAACCCATTGTACCCCATTGTCTGTGGGGCCACAGCCCCTCTATG
ACTTCACCTCCCAGTATGTGGTGGAGACAGACTCCCTTTTCTTACACTACCTTCAAGGGGCTTCAGCCCGGCT
TGATCTCCACCAGGCTGTGGCCAGTGAGCACAACACTCTTGCTGCAGGATGGATTTGCTTTGACAGGGTGCTA
GAGACTGTGGAGAAAGTCCATGGCTTGGCCACACTGATTGGTAAGTGCTGTTAGCTTCCTGCAGCTCCCT-
GCACCAATGCAGAATTTCCCAAACTCTATAACTGCTTTTCTGGTCATTTCCTATTTTCTTCCTTTTTCTTTGT
TCTTGATCATTGCCGCACCATCTGTATTCTCCATGATTTCA-
CACTTGCTGCTGCCCAGGAGCTGGTGGAGAAGAGTTTGGGGTTCTAGAGTACTGGATGAGGCTGCATTTCCCC
ATAAAACCCAGCCTACAGGCCTGCAATAAACGAAAGAAAGCCCAGGTA?AGCACTTGCGCATCAAAGGCAGCT
TGGAGTCTGAAAAGCATCAATACATAATCCATGGAGGGAAAACACCAAACAATGAGCTTTCAGATAAGATTTA
TGTCATGTCTGTTGTTTCCAAGAACAACAAGAAGGTTACTTTTCGCTGCACGGAGAAAGACTTGGTAGGAGAT
GTTCCTGAAGCCAGATATGGTCATTCCATTAATGTGGTATATAGCCGAGGGAAAAGTATGGGTGTTCTTTTTG
GAGGACGCTCATATATGCCTTCCACTCACAGAACCACAGAAAAATGGAATAGTGTAGCTGACTGCCTGCCCCA
TGTTTTCCTGGTGGATTTTGAATTCGGGTGTGCTACATCATACATTCTTCCAGAACTTCAGGATGGGCTATCT
TTTCATGTCTCTATTGCCAAAAATGACACCATCTATATTTTAGGAGGACATTCACTTGCCAATAATATCCGCC
CTGCCAATCTGTACAGAATAAGGGTTGATCTTCCCCTGGGTAGCCCAGCTGTTGAATTGCACAGTCTTGCCAG
GAGGAATCTCTGTCTCCAGTGCAATCCTGACTCAAATTAACAATGATGAATTTGTTATTGTTGGTGGATATCA
GCTTGAAAATCAAAAAAGAATGATCTGCAACATCATCTCTTTAGAGGAAAACAAGATAGAAATTCGTGAGATG
GAGACCCCGGATTGACCCAGACATA?????TGTGTCTCAGCATCTTCCTGCTCTCCACCACACTGAGCCCAAG
ATGACCCAGTGCTCCACAAACGTATGTAGAATGCAAGTAAAACCCCAGGGATCAGAAAAGATAATTAAAGGGC
CGGGCGCGGTGGCTCACGCCTGTAATCCCAGCACTTTGGGAGGCCGAAGCGGGTGGATCACGAGGTCAAGAGA
TCGAGACCATCCTGGTCAACATGGTGAAACCCCGTCTCTACTAAAAATACAAAAAAATTGGCCGGGCGCGGTG
GCTGAAGCCTGTAATCCCAGCACTTTGGGAGGCTGAGGCGGGTGGATCACGAGGTTAAGAGATCGAGACCATC
CTGGTCAACATGGTGAAACCCCGTCTCTACTAATACAAAAAATTAGCTGGGCATGGTGGCGCGTGCCTGTAAT
CCCAGCTACTCAGGAGGCTGAAGCAGGAGAATTGCCTGAACCCAGGAGGCGGAGGCTGCGGTGAGCCAAGATC
GCGCCATTGCACTCCAGCCTGGGTAACAAGAGCGAAACTCCGTCTCAAAAAAAAAAAAATATATATATATACA
ACAATTAGCTGGGTGTGGCGGTGGGTGCCTGTAGTCACAGTTACTTGGGAGGCTGAAGCAGAGAATTGCTTGA
ACCTGGGAGGCAGAGGTTACAGTGAGCTGAGATCACGCCACTGCTGTCCAGCCTGGACAACAGAGTGAGACTC
CATCTCAAAAAATAATAATAAAAAGAAAAGATTGTTAAAGCAGATTCACTACAGTAACAAACCAGCCAAGAAA
TTGCACTGAAAGTAAAGGTTT-
GGGTGACAAAATTTCCAACTATGTTCAAAGAAACGTAAGTTCCAGCAGGAGTGCACGTAATCTGATTTT-
CTTCAAAGC--
CTCTCTAATTTGTAGGGAAGGACATTCATTTTCATTAACTTCATGTGAGCTAGAGAGAATCCATG-
CTGAAGCACTAGAACCCCTGGTAAACATGCCAGCAAGCAAGGGCACCTCCCCCAGAGGCTCAACCTAGAATAT
AAGACTGTCAGCATTGGTAAGGAGCCTTGAACTCTAAACCCATAGGCTCAAGTCAAGAA-
CTCAGGAAGCCTATCAACACCGGGATACAATTTTGCCACCTTAATACTTTATTCCCTTTACT???????????
???
???
???
???
???
???CAAAGATCTTTATTGCCATAAAGCAGAGTAAC
T-GTCTTTGACTTGGACCTTGTTTACTGTGAACTCTAATC-AAGGCAGGACATGCAGCAGTTCTAATGA-
CTGCACTGTGGACGTGTGGAAAGGACCCTTGTGA---------
ATCCAGTTTGGATTAGTGTTTGAAATTGAATTGAAAATGTTGTTCCCAGATGTGGAATGCAGCCGTGATCACC
CTTGCCTACC-GAGCTTACTTTGATCTGTTTGTCAGTAGCATGCAAAAAA-

TGCTTTGTTTGCCCTTTGCTTCTGCTTTTTTCAGGGAAGCTGCCAAAGAATGTCGACGTCGAAAGAAAGAATA
CGTAAAATGTCTGGAGAGCCGAGTCGCAGTGCTGGAAGTCCAGAACAAGAAGCTTATAGAGGAACTTGAAACC
TT???
???
???
???
???
???
???
???
???
??TGTTTATATCTAAATGTGTGGATTCCA
GCACCTAAACCAAAAAATGCCACTGTAATGATATGG-
ATTTATGGCGGTGGTTTTCAAACTGGAACATCATCCTTACATGTTTATGATGGCAAGTTTCTTGCTCGGGTCG
AAAGAGTTATTGTAGTGTCAATGAACTACAGGGTGGGTGCTCTAGGATTCTTAGCTCTGCCAGGAAATCCTGA
GGCTCCAGGAAACATGGGTTTATTTGATCAACGGTTGGCTCTTCAGTGGGTTCAAAAAAATATAGCAGCCTTT
GGTGGAAATCCTAAAAGTGTAACTCTTTTTGGAGAAAGTGCAGGAGCAGCTTCAGTTAGTCTGCATTTGCTTT
CTCCTGGAAGCCATTCCTTGTTCACCAGAGCCATTCTGCAAAGTGGATCCTCTAATGCTCCATGGGCAGTAAC
ATCTCTTTATGAAGCTAGGAACAGAACGTTGGCCTTAGCTAAATTTACTGGTTGCTCTAGAGAAAATGAGACT
GAAATAGTCAAATGCCTTCAAAACAAAGATCCCCAAGAAATTCTTCTGAATGAAGCATTTGTTGTCCCCTACG
GAACTCTCTTGTCAGSAAMCTTTGGTCCAACAGTGGATGGTGATTTTCTCACTGACATGCCAGACATACTACT
TGAACTTGGACAATTTAAAAAAACCCAGATTTTGGTGGGTGTTAATAAAGATGAAGGGACAGCTTTTTTAGTC
TATGGTGCTCCTGGCTTCAGCAAAGATAACAATAGTATCATAACTAGAAAAGAATTTCAGGAAGGTTTAAAAA
TATTTTTTCCAGGAGTGAGTGAGTTTGGAAAGGAATCTATCCTTTTTCATTACACAGACTGGGTAGATGATCA
GAGACCTGAAAACTACCGTGAGGCCTTGGATGATGTTGTTGGGGATTACAATATTATATGCCCGGCCCTGGAG
TTTACGAAGAAGTGCTCAGAATGGGGAAACAATGCCTTTTTCTACTATTTTGAACACCGTTGTGCTAATTGTG
AGAAAATATGCTACTTTGATCAAACTAGAATGAAGGAAATAAAAAGACACTGTGGCTCAACTTGTAATAAAGA
GCAGCTCATCCTCACAGCTTTTTGATTGTTGCTATAGTTATCT---
ATTCAACATGGGATGCACAATGCAGTTAACGTTTCATTACCAGTAGCCTGTTGCGTGCTTATTGTTAAAAGGA
ATTTAACATACAAGTTTTTGTATGGATTGTTCGCCTAATTCCGATTCCTCGTGCTGACTGGCATGAGACAGGG
AGCTGGATTGTATTAAACTGGTTTGATTGTGATGTGACTATGATGGATGTGACAAGGGCCTCTATTATTCGCT
GGGGAGTGTGGCCGTTAATGAAGCCATTAGCTGAGCTAAGGCAAGTGAGGAAAATAGGGAAATTACCATTATT
CTGAAGCCATTAAGTTGTAATCCACCACTTTCTCTCCTTATTCAAAAAGGCATCTTCTTTTTAAGGCCTCCCT
?????TACTATGGTTATTTCATACTTCGGTTGCATGAAGGCTGCCCCCATGAAAGAAGCAAACATCCGAGGAC
AAGGTGGCTTGGCCTACCCAGGTTTGCGGACCCATGGGACTCTGGAGAGCGTGAATGGGCCCAAGGCAGGTTC
AAGAGGCCTGACGTCATCGTTGGCTGACACTTTCGAACATGTGATAGAAGAGCTGTTGGATGAGGACCAGAAA
GTTCAGCCCCATGAAGAAAACAATAAGGACGCAGACTTGTACACGTCCAGGGTGATGCTCAGTAGTCAAGTGC
CTTTGGAGCCTCCTCTTCTCTTTCTGCTGGAGGAATACAAAAATTACCTGGATGCTGCAAACATGTCCATGAG
GGTCCGGCGCCACTCTGACCCTGCCCGCCGAGGGGAGCTGAGCGTGTGTGACAGTATTAGTGAGTGGGTAACG
GCGGCAGACAAAAAGACTGCAGTGGACATGTCGGGCGGGACGGTCACAGTCCTTGAAAAGGTCCCTGTATCGA
AAGGCCAACTGAAGCAATACTTCTACGAGACCAAGTGCAATCCCATGGGTTACACAAAAGAAGGCTGCAGGGG
CATAGACA??TTCTTTGAGCAGATGCA
GAACTAGACCCCC-
GCCACAGCAGCCTCTGAAGTTGGACAGCAAAACCATTGCTTCACTACCCATCGGTGTCCATTTATAGAATAAT
GTGGAAAGAAACGAACCCCTCTGTCTTATGATTTACTCATTATCGCCTTTTGACAGCTGTGCTGTGACACAAG
TAGATGCCTGAACTTGAATTAATCCACAGATCAGTAATGTATTCTCTCTCTCTCT------TT----------
--------ACATTTTGGTCTCTACACTACATTATTAATGGGTTTTGTGTACTGTAAAGAATTTAGCTGTTTC-
AAACCTAGTGCATGA-
ATAGATTCTCTCCTGATTATTTATCACATAGCCCCTTAGCCAGTTGTATATTATTCTTGTGGTTTGTGACCCA
ACTAAGTCCTACTTTAAATATGCTTTAA-GAATCGATGGGGGA---
TGCTTCACGTGAACGTGGGAGTTTAGCTGCTTCTCTTGCCTAAGTATTCCTTTCCT-
GATCACTATGCATTTTAAAGTTAAA-CATTTTTAAGTATTTCAGATGCTTTAGAGAGATTTTTTTCC---
ATGATTGCATTTTACTGTACA-
ATTGCTGCTTCTGCTATATTTGTGATATAGGAATTAAGAGGAT??????????????????????????????

??????????????TCCGGAGAGCACTAAGCCACTTCCAGCCCTGCTGTGAACCAGGACAGTAGGAGCCATTT
TTTTCATGTGGTCAGCTTTGGAAGGATCTACATCCACCACTTTAGAAGACAAGTCTAGTGGCTTATCTGTGAC
GTCTTTGGTAACGGTCTGCTTCTCCAACAGAGGAGGTGAGCTGCCGTCTTTTCTGTCTTGAACCGCTGTTTTC
CGGGAATGCCCGGGCACTGGCTGGGCACCTTCGCCCCCTTCCGGAGCCTTGGGATACTTGCCGTTGGAGAGCC
TGGCCGCGGGGAATTCGCTGCTGACTGTCATGTATGGCTTTGACAGGGCAACTGAAGGAGAGGTGGAGATCCT
GGCATAGTGCTTGTGGAACTCCGAGTAGGTGTCTGCAGCAGGCTGGGTGGGAAGGTGGACCCGGGGTGAGGGC
CGAGGCGAAGGGGGCAACAGGAGAGCCGTGTCCCCCGGCAGGCCACTGGTGACTGCCTTGGCAGAGGGAACCC
TGGGCTGTTTACTGTTCTGGATGTGAGGATAGGCATGGGAATCAACAGGATTCCCAGGGCTGACCCCCATCTT
CCATGGGAGGCTTTTGTCTGCGCAATGGACAAGAGGTGGGATGGCTGGGGAGGCCGAAGGCATCGAGAGCCTC
ATGGGTGATGCCAAGGACGATGGGATGTGGGGACTGACGTAGTGAGGTGGTGGCAGATAGAGAAAGCGCTCCC
CGTTGGTGCAGACTGGAGAATACAGCGGCTGGGCCAAGCTGTAGGACTGCTGAGGTAGCAAGGTGCCAGGACC
TTTGTAATTCAAGTGCTTTTTACAGTAGTAGCAAATGTTACATAAGCCCAAACATGGAGCA---
TATTTGGAGTTGGTGGGCAGACCCAAATCATGATGCAAGCCTGCAGTAGCAAGTGTGCAATGAAGCACAACCA
AATTCTGTGAATGGAAGGTAGCGAGACGCCTCTCCAAAGCAGCCCTTATGCGCAGGAGCTGAAAGCTCCACCG
GGTAAGTTTTCTGAGGCAGTTTTCAGAGGCTGGACTTGGCTCAGGGCCCAGCTCTGGATGAGCCTGTGGACGG
TGGGGT----AGGTCTTTGCTAACCTTACATAGAACCAAGGAG--
TTTGGAAGAGTTTGTGCAGGCCTCTTTCCTCCTCGTGTTTGTCTTGCCCTGCCAGCAGGCCAGGAGGG-
TTGCGCTGCTCAAGTAGTGGAGGGAGGGAGCCTGCAGAGCTAGGGGATCAGAGTGGGTAATTGCAGCCACAGG
GATTAGCTGCTGCAAGCTGTCCACAGGAGCCATTCTGAAATGTCATTTGAGTGATCTGGCCGTCTACTGGAGG
AGGACCTCGGGGCAGCTGTCTCCAGGAGCAGCTTCTGAGGGAGTCTTCCAGGAGGAACAGTGTCTCTGCCATC
GTCCTCGTCCACGTCCTGGTCACATTTCCATTCCTCATCTTCGTTTAAAGTCGGCAGGTATCCAGATATGCCC
TTCCCTGTCCCTGACCCAGAGCTCTGGTCACTACAGACCTTCGGGCTCTCTGAGCCTGTCCTCGTATATTCCA
AATAAATATCAGACTTAAGGAAGGAGGGGTAGGTGTTTTCCTCCATAGTGGCCTGGATTTCTGTCTGGGCCTG
GTCAAACATGGCAGGATCGATCAGCTGCTTCATGATGCAGCCCTTTATGAAGCTCCTGGTGGCCGGCTTGGTC
TGCCGGGACACGATGCCATTGTTATCAAGGATGTACTTTCGGTAGATGGCTCTGGCCAGCTTCAGTCTCTTCT
CCTCATTGGAGTCACAGGGCTCCAGCTTCCTGAAGCCAGTGCAGGCAAACCAGAAGTCCAGCAAGTCAGCACA
GCCCTCCTGCTTCAGGAAAGTCCTGAACAGGCTTATCCCATCTTGATCATCCAGCAGGGAGTGCAGTGACTCA
GCCCACTTCAGGTACGGCGGGGTAGGGGAGGCACTGCCCTCAGGCTCATACCCCAGGTCCAGATCCGAGCGCC
TCGGAGTGGCGGTCGAAGTCTCACCTTTAATGCCAACACCTTTCCCGGAGCAGAAACTGTAGCTGGCGGGCCT
CGGGTCTGTGGACACCAGTTCTCCCTCCTCACCAGGCACTGGGGGTCGGGGAGCATCTTCGGTGAAACTTGCT
CCAAGGTCCAAGGGGAAACCCTGCTCCTGGACATTCATTTGGGACTCTGTGCGCCAATGCACAATGAGCGCT
GCACCCTAACACATCAGTACTTAACAGCTCCAAAGTCAATCAATCTGTCCTGTTGAAACCAT?????CTAAAA
CGTTAGCTAATTTTAAGTGAATGTTA--
ATTACATGGATCGGCTTATGGCAGCATGAAACAGGCGTAATTTACAGTGGAGCAGGATAAGT-
ACTTAGGTTATTTATAGGACAGATTATTTTTCTGTGTTGTTTTG----CATCTTTG-
CATATTAAAATTTATTTACAGGTGTAGTTAAGCTTT--ACTATTAGTTAGCATAAATTTGGCTTGGTGTCA--
--
CCAAAGCAGAAATGTTGGACTTCATTTTTATGAGTTATTTCCTACACATTTCTAAATTGATGTAGCTTTGTCA
CTGCTTGA------------------------------------
GTTTATACTGAATCAAACCAGTTTCTCTTA-TTAGTA-------------------
CATTACTAAAATTGAAAAGAAGTAAAAGGAATGTTAATAACTTAGGAGTATTTGCGTAGTTA-
TGTTCAGAACACAAGCTTTAAATTAGT-GAGGAAGCCA---------
GAAGTTTGATTTAAGCACTCATACTCCTTTATTTTCCTTTGTTAGCAATAATTATTGGCCCTGATGGCCATCC
TTTGACTGTTTATCCTTGTATGATGTGTGAGAAGAAGTTTAAGTCGAGAGGTTTTTTGAAAAGACACATGAAA
AACCATCCCGAACATCTTGCCAAGAAGAAGTACCACTGTACTGACTGTGATTACACTACCAACAAGAAGATAA
GTTTACACAACCACCTGGAGAGCCACAAACTGACCTCCAAGGCAGAGAAGGCCATTGAATGTGATGAGTGTGG
GAAGTATTTTTCTCATGCAGGGGCTTTGTTTACTCACAAAATGGTGCACAAAGAAAAAGGAGCCAACAAAATG
CACAAGTGTAAATTCTGTGAATACGAGACAGCTGAACAAGG????????????????????????????????
?????????????????????????TATAGCTTCTGCAAACAGTAAAGATACCACTAAAACCCCTTCTGCAGT
TCAAACTAGCACCAGCCACAACCCTGGACTTCCTCCTAATTTTAACGAATGGTACGTCTGAGGACAAACACAA
ACCCTGTTAACTATAGAATGGACCAAATGCATTTTTAAAAGAAAACTGAGACCAATCAGATGGAAATGGAGTT
TTAAGGCAAGAGGCCATATATAGGGCTACATCTTGTTAATTGCAATTGTCCAGGAAGGTTTTGGGCAAGATCC
AAAAGTAGCCATGCCCTTTTCTCAGGATTAGAAAATATGTTTTGGCGTTCGAAGGATTTTTT--
ACAAAATCTTTACACTGCTTTTTCTTCCCCTTCCTCTTGCTCTCTGCACACCCCATTCCTAAACTCCTGCAAT

TCATTTTAACACTTGTCCTGCTTCTTGAGAGGAAGTTATAGAAGGCTTGTTGGTGGTGGTGATGTTAAACTGA
TGGAAATTCTTTTTCGCCTTAGTGGTGATTGTTTAAACTCTCACAGTCTTAAACCGTGCCAAAGTCCTGTTAT
A
Ateles
?TACCCCCATGTTTGGCTGGAACATGAAGCTGACCTCAGAGCACTACAGAAATGTCACCTTCCTTTCATGCCG
GTTCCCTT-
CCGTCATGAGCATGGACTACATGGTATACTTCAGCTTCTTCACCTGGATTTTCATTCCCCTGGTCATCATGTG
TGC--AATCTATCTTGACATCTTTTACAT-
CATCCGGAACAAACTCAGTCAGAACTTCGCTAACTCCAAAGAGACAGGTGCATT---
TTACGGACGGGAGTTCAAGACGGCGAAGTCCCTGTTTCTGGTGCTTTTCTTGTTTGCTTTGTCATGGCTGCCT
TTGTCCATCATCAACTGCATCACCTACTTTCA--------------
TGGTGAGGTCCCACAGCTTGTGCTGTACTTGGGCATCCTGCTGTCCCATGCCAACTCCATGATGAACCCTATC
?????????????????????ATGTTCATAACTCGCTGATTATTCTCAACCAACCATAAAGACATCGGAACAC
TATACCTACTATTTGGCGCATGAGCAGGGGCAGTAGGAACAGCCCTAAGCCTCCTAATTCGAGCGGAACTCGG
TCAACCAGGGAGTCTAATAGAAGACGATCATGTTTATAATGTAATCGTTACCTCCCATGCATTTATCATAATT
TTCTTTATAGT---CATGCCAATTATAATTGGAGGCTTTGG---
AAACTGACTTGTTCCCTTAATAATTGGTGCCCCCGACATAGCATTTCCCCGAATAAATAATATAAGCTTCTGA
CTTCTACCCCCA--TCCCTCTTACTTCTACTCG-
CATCATCAACCCTAGAGGCCGGCGCCGGTACTGGCTGAACAGTTTACCCACCCTTAGCAGGAAATATATCACA
CCCAGGAGCCTCTGTAGATCTGACTATTTTTTCACTCCACCTAGCAGGTGTTTCTTCCATTTTAGGGGCCATT
AACTTTATTACAACAATTATTAATATAAAACCCCCAGCCATAACCCAATACCAAACACCTCTCTTTGTCTGAT
CAGTCCTTATTACAGCAGTTCTCCTACTTCTATCCCTTCCAGTTCTAGCTGCTGGAATTACAATATTATTAAC
TGACC--
GCAATCTTAATACTACCTTTTTTGACCCCGCTGGTGGAGGAGACCCAATCTTATATCAACATTTATTCTGATT
CTTTGGACACCCTGAAGTATATATTCTCATTCTCCCTGGATTTGGAATAATTTCACATATTGTAACATATTAC
TCTAACAAAAAAGAACCATTCGGTTATATAGGAATGGTATGAGCCATAATATCTATTGGCTTCCTAGGATTTA
TCGTATGAGCTCACCATATATT-
TACTGTAGGAATAGATGTAGACACACGTGCATACTTCACATCAGCTACTATAATTATTGCTATTCCCACTGGA
GTAAAAGTATTTAGCTGATTAGCCACACTACACGGTGGTAACATCAAATGATCTCCTGCAATGCTATGAGCTC
TGGGTTTTATTTTCCTCTTTACTGTAGGCGGACTTACAGGAATTGTACTAGCCAACTCATCACTAGACAT-
TGTCCTGCACGATACATACTACGTAGTAGCCCACTTTCACTATGTCTTATCTATGGGGGCCG-
TATTCGCTATCATAGGGGGCTTTATTCACTGATTTCCACTATTCTCAGGCTATACACTCGACCACACCTACGC
TAAAATTCATTT-
CACCGTCATATTCGTAGGCGTAAATATAACCTTCTTCCCACAACACTTTCTCGGTCTATCTGGGATACCCCGA
CGATACTCAGATTACCCCGATGCATATACCACATGAAATATCATCTCATCCGTAGGCTCATTCATCTCACTAA
CAGCAGTCATCCTAATAATTTTTATAATCTGAGAAGCCTTCTCCTCAAAACGAAAAGTCTTAGCCATCGAACA
ACTATTCACTAACCTAGAATGACTCTATGGATGTCCTCCTCCCTATCACACATTTGAAGAGGCTACATATGTA
AAGTCTTTAAAC?????????????????????????ATGGCCCACCCAGCTCAACTAGGATTACAAAATGCTA
CATCACCCATCATAGAAGAACTTATCGCTTTCCACGACCATACTCTTATAATTATTTTTCTAATTAGCTCGCT
AGTATTATATATTATCTCCATAATACTTACTACAAAACTAACCCATACCAGTACCATAAATGCCCAAGAAATC
GAAATAATCTGAACTATTCTACCCGCAATTATCCTTATCATAATCGCCCTCCCATCCCTACGTATTTTATATA
TAACAGACGAATTTAACAAGCCCTACCTAACCCTCAAAGCAATCGGCCACCAGTGATACTGAAGCTACGAGTA
CTCAGACTATGTGGATTTAGCCTTTGACTCTTATATTATACCTACATACTTTCTTGAACCTGGTGAATTCCGA
CTTCTTGAAGTAGATAACCGAACAACCCTACCAATAGAAGCAGACATCCGCATATTAATCTCATCACAAGACG
TCTTACACTCATGAGCTGTCCCATCGCTAGGTGTTAAAGCAGATGCAATTCCAGGACGCCTAAACCAAGCCAT
ACTAGCCTCTATACGTCCAGGCCTGTTTTACGGACAGTGTTCAGAAATCTGCGGGTCAAATCACAGCTTTATA
CCTATTGTCCTAGAATTCATCTACTTCCAAGATTTCGAAGTATGAGCCTCATA????????????????????
ATGACCACCCCTCGCAAAACACACCCACTAGCAAAAATTATTAATAACTCACTCATTGACTTACCTTCACCAT
CCAACATCTCTGCCTGATGAAATTTTGGCTCACTTCTTGGCATCTGCTTAATTATTCAAATCACCACAGGCCT
ATTCCTAGCCATACACTACACACCAGATACTTCAACCGCTTTTTCCTCAGTTGCCCACATTACCCGAGACGTC
AACTACGGTTGAATAGTCCGCTACCTACACGCAAATGGTGCCTCCATATTCTTTATCTGCCTCTTTCTGCATA
TCGGCCGAGGCTTATATTACGGATCTTTCCTCTCTCTAAAGACCTGGAATATCGGTGTTATCCTACTACTTAC
AACCATAGCCACAGCATTCATAGGTTATGTCCTCCCATGAGGCCAAATATCATTCTGAGGGGCCACAGTAATT
ACAAACCTCCTATCAGCCATCCCCTACATTGGGTCCAATATCGTACAATGAGTCTGAGGTGGCTTTTCAGTAG

ACAAAGCTACCCTTACACGATTCTTTACCTTTCACTTTATCCTACCCTTTATTATTGCAGCCCTAGCAACTAT
CCACCTCTTGTTTCTGCATGACACAGGATCAAGTAACCCGTCAGGAATGGCATCAGACCCCGACAAAATCACA
TTCCACCCCTACTACACAATCAAAGACATCCTAGGTTTAATCCTTCTTCTCCTATGCTTAATAAGCTTAACCT
TATTCTCACCCGACCTTCTAACTGACCCAGACAATTATACGCTAGCCAACCCTCTCAACACCCCACCCCATAT
CAAGCCAGAATGATACTTTCTATTTGCATACGCAATCTTACGATCTATTCCCAACAAGCTAGGAGGCGTTCTA
GCCCTAGTTCTATCCATCCTAATCTTAATAATTATTCCTACTCTACACCTATCCAAGCAACAAACCATAAAAT
TTCGACCCATCACCCAAATCCTATTCTGGGCCCTAGTAGCTGATCTACTCACACTAACATGAATTGGAGGTCA
ACCAGTCGAATACCCTTTCATAGCTATCGGCCAAACTGCATCAATCATATACTTTCTTACTATTATTACACTC
ATCCCCTTATCCACTTTAATTGAAAACAAACTACTTAAATGGTAA?TTAGGTTAAATTTATTCAAGGGAATGA
GAATTTATGGGTATAACTGTTGGCTGGAAAGCCCTTCCTCACCTCCAATCCTCCTTTCAAACTACATTTCC-
TCCTGAAATTTCTCTGTGGGATGAAATGTTCAATTATTAGTCAATCAGTC----
TCTTATACGTTATCTGGCTTTACATAT----
GCCCTTGATAGTTCTTGGAAAATAATCCAATATTGTGTTGTTTTGGTGTCTTTGTTTTGGAAATGGAAGCATT
TTGTATGAATCACCTCTTTAACTGTGAGGAGTTGAATTGTCTTAGTTCTGCTTGCCTAATGACCAGAAATAAA
--GACATCACAGTCTCTAACCCCACTTTTT-
GGTGGAAAGAAAATCTAAAAGTAGCAACATTTAATGGAACAAAATATCAATAATCAAAGAAACTCCCCTGTGA
AACCGTAATATTATTTT-
AAAATCAACATTGTTCTAAGGTAGCTGAAATGTGTGATTTTTCTATTTTGCCTTTTCACTGACAATATACCCA
TAAATCT-
TGGTGGTGATTTTCTCTCCAAAGTTAATTTCTGGGCCATTGGTGACTGCACTATCTATTGTTCTAAATTGTTG
TCTTAATTGCATTCTTTAAAAATGTAATGTTGAAGGCACATAATATTGAAGATGACTCCAGGATGAACCCTGA
GTTCGCAGACCGAA-
TAAAACAACTCGATAAAGAGGCGTCTTACTACCGTGATGAGTGTGGCAAGGCCCAAGCGGAAGTGGACCGGTT
GCTGGAGA?????????GGAAGAGCTGCATCAGGCAAGTACTTTGATCTTTCTTGGAGCAGGTTCCTTCAAAA
CGCTTCTCCAGAGGACGGAAGTCCCTCCAATCTGGCTCGCTGCTCGCAGTGGTGGGGAAAGCTGAGGTAATTC
CCCGGTGGGAAATCTGCGAGGCCCCTGATACCCCTG--------
CCACCAGGCCCATCACTGACGGGCCTAAGGGCCTCATCTGATACTCTAAGACGGGCTTCTCCTGTCGGATCTG
GGCCTCTCGGACTTGAGCCTCTCTAGAACGAGCCTCTCGGGCCTGGGCATCTCGGGTTCTGGCCTCCCTGGCA
TGACCTTCCCAAGTGTGGGCCTCCCTG--
GCATAGGCTTCCTTGCCATGAGCCTCTCGAGTATAGGCCTCCCTGGTGTGGGCCTCCCTGGCATGAGCTTCTC
GGGCACGTGTCTCCCGGGCCTCAAGCTGCTCCCGCCGAAGCTCCCAATACAACAACTGTTTCTGGATGGTCA-
CTAGCCGTTCCTCCTCTGTCTCCATTGCCCCAGGTGGCCGGGAGGAAGAAAAGGGCTCAAAGTTCAAGAAGGG
GTCAAACATCTCAGAGCTTCGACCATGGAGGTCATAAAGGCAGTCATCCCCAGGTGGAGAGTTCTCAAGACTG
TCATCTGGCTCATAGAATTCATATAGGGCATCTCCACTGTAGCTGTCTCGGGGCAGACAATCCCTGCGGACAA
GCCCCAGGGCCTCACCTGAATCATCCTCAA??????AGCATCACTAATTTTGATGAACTAAAAATGGTTCCTG
AAGGTCAGATTCATGTACAGTGAATGTTCTCCACTGTTGTGGTAGGCATGTTTCATGTGTGGGGATGTTGGTG
TGCAAGTTTCATGTGTTTGGAAAACACTAGCTAGAACATTCGAAAAATTTCATTAACCCAGCTCTGGAAGGTC
ATCTATGAATTCTTCCTATTCCAGTGGCCTTTT-
AAGTGTAGATGTAGATACACTGTAATTTAAAACAGATGGGCATGTCTGAATATTTTTGTATGTAAAATGGGAC
AGATGTATATGAATGGATAAATTATCCTTTTAAAAAGATACTTTGTAGCACGAAATATCACCAATGACTG-
AACAGTGAACCTGCATTCAGTCTAGGGCAAACACCAGGAAGACATTATCAAAACATCTAA-
CTATCTATAAACTAATTCCAATTAGTCTACTTCGTCTTCATACAGAAAAAA--TTTTT-GATTTTTAAA---
TATGTATTGGGTGCCAATCCGTATCCTTT-
GAGCTGCTAAAGCCAGCATATCACAATAGAAGTTTACTTTTCCTTG???????????ATGTTCATAGTAAATT
TACTCCTACTTATTATCCCCGCCCTAATTGCTATAGCATTCTTAACACTTATAGAACGAAAAATCTTAGGTTA
TATACAATTTCGCAAAGGTCCTAACATCGTAGGCCCCTATGGGGTACTTCAACCAATTGCTGATGCAATAAAA
CTCTTCACAAAAGAACCCCTACTACCCACCACATCTGCTTCCACCCTATATATAACCGCCCCAGCCTTAGCCT
TATCAATCGCTCTTCTTATATGAAGCCCCCTTCCAATACCACACCCCCTAATTAACTTCAACCTAGGCCTCTT
ATTTATATTAGCAACATCAAGTCTAGCCGTATATTCAATTTTGTGATCCGGATGAGCATCCAACTCAAACTAC
GCACTAATCGGCGCATTACGAGCAGTAGCCCAAACAATCTCATACGAAGTCACTCTAGCCATCATCCTACTAT
CAACCCTACTAATAAGCGGCTCATTTAACTTACAATCACTTATCACAACACAAGAACACTCCTGACTTTTACT
ACCATCATGACCCATAGCAATAATATGATTCATTTCTACACTAGCAGAAACCAATCGAGCCCCCTTCGATCTA
ACAGAAGGCGAATCAGAACTAGTTTCAGGATTCAACATTGAATATGCCGCAGGCTCATTTGCCCTATTCTTTA
TAGCAGAATATATAAATATTATCATAATAAATGCCCTAACAACTACCATTTTCCTAGCCATACCTTACAATAT

AGCCTCATCAGAACTCTATACAATAAATTTTATAACTAAAACCCTCCTATTAACCACCCTATTTTTATGAATT
CGAACAGCATACCCTCGATTTCGCTATGACCAATTAATACATCTCCTATGAAAAAAATTTTTACCACTTACAC
TAGCACTATGTATGTGATATATTTCCATACCAACCCTGACATCTGGAATTCCACCCCAAACAT??ATTAACCC
CCTAGCTCATCTCATTATTTCCCTCACTATTATGATGGGAACTATAATCACAATTCTAAGCTCACACTGATTC
CTCATTTGAATGGGCTTAGAATTAAATATACTAGCCATTGTACCAGTACTAATAAAAAATTCAAGCCCCCGCT
CCACAGAAGCAGCCACTAAATATTTTCTTACACAAGCAACCGCATCCATAATTCTACTAATAGCCATTTATCT
CAACAACCTATTTTCCGGACAATGAACAATTAATTCATCCCTAGACCAAACTCTAACCACAATAACACTAATT
GCCCTAACAATAAAACTAGGAATAGCCCCCTTCCACTTTTGACTCCCAGAAGTAACCCAAGGCACTCCTCTAA
TCCCTACCATAATCATTCTAACATGACAAAAACTAGCCCCCTTGTCAATTATACTACAAATCTTCTCATCCAT
CAACATTAATATCCTCTTAACAATATCAATCCTATCTATTATAATCGGAAGCTGAGGGGGACTAAATCAAACA
CAACTACGCAAAATCCTAGCTTATTCCTCAATCACCTACATAGGATGAATGACGGCAGTACTATATCATGACC
CAAATATCACCACTTTAACCCTACTCATCTATATTTTCCTAACAATCTCTACACTCATAACCTTTTATCTAAA
CTCAAATATAACCACCCTATCACTATCACACACCTGAAACAAAATTACATGAATAATGCCCGCAATTCCATTA
ATAATGATATCGCTAGGAGGTTTACCCCCACTAACAGGATTTTCCCCTAAATTTGCTATTATACAAGAACTCA
CAAAAAATAATAACATTATTATTCCCCTTACTATAGCTATACTAACACTAATAAACCTATACTTCTACATACG
CCTAACTTACGCCATCTCAATGACAATATTTCCCACATCCAACAGCACAAAAATTAATTGACAACTAAAGTAT
ATAAAATCCACACCACTTCTCTCCCCACTTATAACATCATCCACACTTCTACTTCCCGTAACCCCACTAATAC
TAATAGTCT?????GTTGGCAGCATAAATAATAAAGCATGTTACTTAAGCAATTTTACATTTACTGTATTAGT
AAGTAAATGCAATGGCCACTCTTATTCTCAAACAATAAAAACTACCCAGCCTTTTCATGAAAGAT-
GATTGCAATTTCCATTGGCCTGTATATGCAATAAAAAG---
CCATAACTCATCAAAATTTTAGCTATTTTCTCTTAAGATA--
GAAGAGCATTTTGCTTGAACATTGAACGTGACATTAAATATT-
CATGAAACTGAATAGAGTTTAAGTACTAAG---
TAAAACTCTCAAATTCTTTTCAACTAGACKGAAATTCTGTTTGAACCAGGTAAAAACTACCGCTAAGCTGAAG
AGGTGTTCAAAAAC-
ATCGTAAAGAGATTGTACAATATGTTCAAAGTGCTACAGTTATTTGGGATAGATTTTTCTGAAAAGCCTGTTC
CTCCTTTGAAATTGATTCATGATAACAGCCTCCATTTGGGTCAAACAGTATTTAACCGCTTGTAAA-
TTTTAATT-
AAATGATTTGTCACTTTGAACACATAGTTTGTCCCCAAGACCTTGAGGATTACTTGAATGGGCCCTTCACTGT
GGTTGTGAAGGAGTCTTGTGATGGAATGGGAGATGTGAGTGAGAAGCATGGGAGTGGGCCAGTAGTCCCGGAA
AAGGCAGTCCGTTTTTCATTCACAATCATGAAAATTACTATAGCCCACAACTCTCAGAACCTGAAAGTGTTTG
AAGAAACCAAACCTAATTCTGAACTGTGTTGCAAGCCATTGTGCCTTATGCTGGCAGATGAGTCTGACCACGA
GACCCTGACTGCCATCCTGAGTCCTCTCATTGCCGAGAGGGAGGCCATGAAGAGCAGTGAATTAATGCTTGAG
ATGGGAGGCATTCTCCGGACTTTCAAGTTCATCTTCAGGGGCACTGGATATGATGAAAAGCTTGTACGGGAAG
TGGAAGGCCTTGAAGCTTCTGGCTCAGTCTACATCTGTACTCTTTGTGATGCCACCCGTCTGGAAGCCTCTCA
AAATCTTGTCTTCCACTCTATAACCAGAAGCCATGCCGAGAACCTGGAACGTTATGAGGTCTGGCGTTCCAAC
CCTTACCATGAGTCTGTGGAAGAACTGCGGGATCGGGTGAAAGGGGTCTCAGCCAAACCTTTCATTGAGACAG
TCCCTTCCATAGATGCACTCCACTGTGACATTGGCAATGCAGCTGAGTTCTACAAGATCTTCCAGCTAGAGAT
AGGGGAGGTGTATAAGAATCCCAATGCTTCCAAAGAGGAAAGGAAAAGGTGGCAGGCCACATTGGACAAGCAT
CTCCGGAAGAAGATGAACCTCAAACCAATCATGAGGATGAATGGCAACTTTGCCAGGAAGCTCATGACCAAAG
AGACTGTGGATGCGGTTTGTGAGTTAATTCCTTCTGAGGAGAGGCACGAGGCTCTGAGGGAGCTGATGGATCT
TTACCTGAAGATGAAACCAGTATGGCGATCATCATGCCCTGCTCAAGA--GTGCCC-
AGAATCCCTCTGCCAGTACAGTTTCAATTCACAGCGTTTTGCTGAGCTCCTTTCTACCAAGTTCAAGTATAGG
TATGAGGGGAAAATCACCAATTATTTTCACAAAA???????????TATGGCACCCGACAGTTGTCATTATGTT
TGGAAACACTGCCGGCCCATGGAGATGAGGATAAAGTGGACGTTTCTCTGCTGCATCAGGGTGAGAATCTTTT
TGAACTGCACATCCACCAGGCCTTCCTGACATCTGCCGCCCTGGCTCAGGCTGGAGACGCCCAACCTACCACT
TTCTGCACCTATTCCTTCTATGACTTTGAAACCCATTGTACCCCATTATCTGTGGGGCCACAGCCCCTCTACG
ACTTCACCTCCCAGTATGTGGTGGAGACAGACTCCCTTTTCTTACACTACCTTCAAGGGGCTTCAGCCCGGCT
TGATCTCCACCAGGCCGTGGCCAGTGAGCACAACACTCTTGCTGCAGGATGGATTTGCTTTGACAGGGTGCTA
GAGACTGTGGAGAAAGTCCATGGCTTGGCCACACTGATGGGTAAGTGCTGTCGGCTTCCCGCAGCTCCTA-
GCACCAATGCAGAATTTCCCAAACTCGATAGCTGCTTTTCTGGTCGTTTCTTATTTTCTTCCTTTTTCTTTGT
TCTTGATCCTTGCCACACCATCTGTATTCTCCATGCTTTCA-
TACTTGCTGCTGCCCAGGAGCTGGTGGAGAAGAGTTTGGGGTTCTAGAGTACTGGATGAGGTTGCGTTTCCCA
ATAAAACCCAGCCTACAGGCATGCAATAAACGAAAGAA???????????????????????????????????

??????????????????????????GTTCATGGAGGGAAAACACCAAACAATGAGCTTTCAGATAAGATTTA
TGTCATGTCTGTTGTTTCCAAGAACAACAAGAAGGTTACTTTTCGCTGCACAGAGAAAGACTTGGTAGGAGAT
GTTCCTGAAGCCAGATATGGTCATTCCATTAATGTGGTATATAGCCGAGGGAAAAGTATGGGTGTTCTTTTTG
GAGGACGCTCATACATGCCTTCTACCCACAGAACCACAGAAAAATGGAATAGTGTAGCTGACTGCCTGCCCCA
TGTTTTCCTGGTGGATTTTGAATTTGGGTGTGCTACATCATACATTCTTCCAGAACTTCAGGATGGGCTATCT
TTTCATGTCTCTATTGCCAAAAATGACACCATCTATATTTTAGGAGGACATTCACTTGCCAATAATATCCGCC
CTGCCAACCTATACAGAATAAGGGTCGATCTTCCCCTGGGTAGCCCAGCTGT-
GAATTGCACAGTCTTGCCAGGAGGAATCTCTGTCTCCAGTGCA??????????????????????????????
???
??TGTGTMTCAGAATCTTCCTGCTC
TCCACCACACTGAGCCCACCATGACCCAGTGCTCCACAAACATATGTAGAATGGAAGTAAAACCTCAGGGATA
AGAAAAGATAATTAAAGGGCTGGGCGCGGTGGTTCATGCCTGTAATCCCAGCACT-
TAGGAGGCTGAGGCRAGTGGATCACAAGGTCCGGAGTTCAAGACCAGCCTGGCCAATATGGTGAAAACCCATC
TCTACTAAAAATA---CAACAATT-----
AGCTGAG--
---------------------CGGGGTGGTGGGCACCTGTAATCCCAGCTACTTGGGAGGCTGAAGCAG-
AGAATTGCTTGAACCTGGGAGGCAGAGGTTGCACTGAGCAGAGATCATGCCACTGCACTCCAGCCTGGACAAC
A-GAGTGAGACTCCATCTCAA--

---AAAA-
TAATAATAAAAAGAAAAGATAGTTAAAGCAGATTCAATACAGTAACAAACCAGCCAAGAAATTGCAGTGAAAG
TAAAGGTTT-
GGGTGACAAAATTTCCAACTGTGTTCAAAGAAACATAAGTTCCAGCAGGAGTGCACGTAATCTGATTTT-
CTTTAAAGY--
CTCTCTAATTTGTAGGGAAGGACATCCATTTTCATTAGCTTCATGTGAGCTAGAGAGAATCCATGAYTGAAGC
GCTAAAACCCCTGGTAAACATGCCAGCAAGCAAGGGCACCTTCCCCAGAGGMTCAACCTAGAAYATAAGACTG
TCGGCATTGGTAAGRAGCCTTGAACTCTAA-CCCATAGGCTCAAGTCAGGAA-
CTCAGGAAGCCTATCACCACCGGGATACAATTTTGCCACCTTAACACTTTATTCCCTTTACTTGTGCCACACT
???
???
???
???
???
????????????????????????????????CAAGGCCCTCAAAGATCTTTATTGCCATAAAGCAGAGTAAC
T-GTCTTTGACTTGGACCTTGTTTACTGTGAACTCTAATC-
AAGGCAGGACATTCAGCAGTTCTTCTTAACTGCACTGTGGACTTGTGGAAAGGACACTTGTGA---------
ATCCAGTTTGGATGCGTGTTTGAAATTGAATTGCAAATGGTGTTCCAGGATGTGGAATGCAGCCGTGATCACA
CTTGCCTACC-GAGCTTCCTTTGATCTGTTTGTCAATAGCATGCAAAAAA-
TGCTTTGTCTGCCCTTTGCTTCTGCTTTTTTCAGGGAAGCTGCCAAGGAATGTCGACGTCGAAAGAAAGAATA
CGTAAAGTGTCTGGAGAGCCGAGTTGCAGTACTGGAAGTCCAGAACAAGAAACTTATAGAGGAACTTGAAACC
TTGAAAGACATT???
???
???
???
???
???
???
???
???
???
??TGTTTATATCTAAATGTGTGGATTCCA
GCACCTAAACCAAAAAATGCCACTGTCATGATATGG-
ATTTATGGTGGTGGTTTTCAAACCGGAACATCATCCTTACATGTTTATGATGGCAAGTTTCTTGCTCGGGTCG
AAAGAATTATTGTAGTGTCAATGAACTACAGGGTGGGTGCCCTAGGATTCTTAGCTTTGCCAGGAAATCCTGA
GGCTCCAGGAAACATRGGTTTATTTGATCAACGGTTGGCTCTTCAGTGGGTTCAAAAAAATATAGCAGCCTTT

GGTGGAAATCCTAAAAGTGTGACTCTTTTTGGAGAAAGTGCAGGAGCAGCTTCAGTTAGTCTGCATTTGCTTT
CTCCTGGAAGCCATTCCTTGTTCACCAGAGCCATTCTGCAAAGTGGATCCTCTAATGCTCCTTGGGCAGTAAC
ATCTCTTTATGAAGCTAGGAACAGAAYGTTGGCCTTAGCTAAATTTACTGGTTGCTCTAGAGAAAATGAGACT
GAAATAGTCAAATGYCTTCAAAACAAAGATCCCCAAGAAATTCTTCTGAATGAAGCATTTGTTGTCCCCTACG
GAACTCTCTTGTCAGTAAACTTTGGTCCAACAGTGGATGGTGATTTTCTCACTGACATGCCAGACATATTACT
TGAACTTGGACAATTTAAAAAAACCGAGATTTTGGTGGGTGTTAATAAAGATGAAGGGACAGCTTTTTTAGTC
TATGGTGCTCCTGGCTTCAGCAAAGATAACAATAGTATCATAACTAGAAAAGAATTTCAGGAAGGTTTAAAAA
TATTTTTTCCAGGAGTGAGTGAGTTTGGAAAAGAATCCATCCTTTTTCATTACACAGACTGGGTAGATGATCA
GAGACCTGAAAACTACCGTGAGGCCTTGGATGACGTTGTTGGGGATTACAATATTATCTGCCCGGCCTTGGAG
TTCACCAAGAAGTTCTCAGAATGGGGAAACAATGCCTTTTTCTACTATTTTGAACACCGTTGTGCTAATTGTG
AGAAAATATGCTACTTTGATTAAACTAGAATGAAGGAAATAAAAAGACACTGTGGCTCAACTTGTAATAAAGA
GCAGCTCATCCTCACAGCTTTTTGATTGTTGCTATAGTTATCT---
ATTCAACATGGGATGCACAATACAGTTAACGTTTCATTACCAGTAGCCTGTTGCGTGCTTATTGTTAGAAGGA
ATTTAACATACAAGTTTGTGTATGGATTGTTCGCCTAATTCCGATTCCTCGTGCTGACTGGCATGAGACAGGG
AGCTGGATTGTATTAAACTGGTTTTATTGGAATGTGACTATGATGGATGTGACAAGGGCCTCTATTATTCGCT
GGGGAGTGTGACCGTTAATGAAGCCATTAGCTGAGCTAAGGCAAGTGAAGAAAATAGGGAAATTACCATTATT
CTGAAGCCATTAAGTTGTAATCCACCACTTTCTCTCCTTATTCAAAAAGGCATCTTCTTTTTAAGGCCTCCCT
???
????????????CCTACCCAGGTGTGCGGACCCATGGGACTCTGGAGAGCGTGAATGGGCCCAAGGCAGGTTC
AAGAGGCCTGACATCATCGTTGGCTGACACTTTCGAACATGTGATAGAAGAGCTATTGGATGAGGACCAGAAA
GTTCGGCCCCATGAAGAAAACAATAAGGACGCAGACTTGTACACGTCCAGGGTGATGCTCAGTAGTCAAGTGC
CTTTGGAGCCTCCTCTTCTCTTTCTGCTGGAGGAATACAAAAATTACCTGGATGCTGCAAACATGTCCATGAG
GGTCCGGCGCCACTCTGACCCTGCCCGCCGAGGGGAGCTAAGCGTATGTGACAGTATTAGTGAGTGGGTAACG
GCGGCAGACAAAAAGACTGCAGTGGACATGTCGGGCGGACGGT??????????????????????????????
???
???TTCTTTGAGCAGATGCA
GAACTAGACCCCC-
GCCACAGCAGCCTCTGAAGTTGGACAGCAAAACCATTGCTTCACTACCCGTCGGTGTCCATTTATAGAATAAT
GTGGAAAGAAACAAACCCCTCTGTCTTATGATTTACTCATTATCGCCTTTTGACAGCTGTGCTGTAACACAAG
TAGATGCCTGAACTTGAATTAATCCACAGATCAGTAATCTATTCTCTCTCTCTCT------TT----------
--------ACATTTTGGTCTCTACACTACATTATTAATGGGTTTTGTGTACTGTAAAGAATTTAGCTGTTTC-
AAACCTAGTGCATGA-
ATAGATTCTCTCCTGATTATTTATCACATAGCCCCTTAGCCAGTTGTATATTATTCTTGTGGTTTGTGACCCA
ACTAAGTCCTACTTTAAATATGCTTTAA-GAATCGATGGGGGA---
TGCTTCACGTGAACGTGGGAGTTTAGCTGCTTCTCTTGCCTAAGTATTCCTTTCCT-
GATCACTATGCATTTTAAAGTTAAA-
CATTTTTAAGTATTTCAGATGCTTCAGAGAGATTTTTTTTTTCCATGATTGCATTTTACTGTACAGATTGCTG
CTTCTGCTCTATTTGTGATATAGGAATTAAGAGGAT?????????????????????????????????????
???????TCCGGAGAGCACTAAGCCACTTCCAGCCCTGCTGTGAACCAGGACTGTAGGAGCCATCTTTTTCAT
ATGGTCAGCTTTGGAAGCATCTACATCCACCACTTTAGAAGACAAGTCTAGTGGCTTATCTGTGACGTCTTTG
GTAACGGTCTGCTTCTCCAACAGAGGAGGTGAGCTGCCGTCTTTTCTGTCTTGAACTGCTGTCTTCCGGGCAT
GCCCGGGCACTGGCTGGGCACCTTCGCCCCCTTCCAGAGCCTTGGGATACTTGCCGTTGGAGAGCCTGGCCAC
CGGGAACTCGCTGCTGACTGTCATGTATGGTTTCGACAGGGCAACTGAAGGAGAGGTGGAGATCCTGGCATAG
TGCTTGTGGAACTCTGAGTAGGTATCTGCAGCAGGCTGGGTGGGAAGGTGGACCCGGGGTGAGGGCCGAGGCG
ACGGGGGCAACAGGAGAGCTGTGTCCCCCGGCAGGCCACTGGTGACTGCCTTGGCAGAGGGAACCCTGGGCTG
TTTACTGTTCTGGATGTGAGGATAGGCATGGGAATCAACGGGATTCCCAGGGCTGACCCCCATCTTCCACGGG
AGGCTTTTGTCTGCGCAATGGACAAGAGGCGGGATGGCTGGGGAGGCCGAAGGCGTTGAGAGCCTCATGGGTG
ATGCCAAGGACGATGGGATGTGGGGACTGACGTAGTGAGGTGGTGGCAGGTAGAGAAAGCGCTCCCCGTTGGT
GCAGACTGGAGAATACAGCGGCTGGGCCAAGCTGTAGGACTGCTGAGGT???????TGCCAGGACCTTTGTAA
TTCAAGTGCTTTTTACAGTAGTAGCAAATGTTATATAAGCCCAAACATGGAGCA---
TATTTGGAGTTGGTGGGCAGACCCAAATCATGATGCAAGCCTCCAGTAGCAAGTGTGCAATGAAGCACAACCA
AATTCTGTGAATGGAAGGTAGCGAGACGCCTCTCCAAAGCAGCCCTTCTGCGCAGGAGCTGAAAGCTCCACCG
GGTAAGTTTTCTGAGGCAGTTTTCAGAGGCTGGGCTTGGCTCAGGGCCCAGCTCTGGATGAGCCTGTGGACGG
TGGGGTGGGTAGGTCTTTGCTAACCTTACATAGAACCAAGCAAAGTTTGGAAGAGTTTGTGCGGGCCTCTTTC

CTCCTGGTGTTTGTTTTGCCCTGCCAGCAGGCCAGGAGGG-
CTGCGCTGCTCAAGTAGTGGAGGGAGGGAGCCTGCAGAGCTAGGGGATCAGAGTGGGTAATCGCAGCCACAGG
GATTAGCTGCTGCAAGCTGTCCACAGGAGCCATTCTGAAATGTCATTTGAGTGATCTGGCCGTCTACTGGAGG
AGGCCCTCGGGGCAGCTGTCTCCAGAAGCAGCTTCTGAGGGAGTCTTCCAGGAGGAGCAGCCTCTCCGCCATC
GTCCTCGTCCACGTCCTGGTCACATTTCCATTCCTCATCTTCGTTTAAAGTCGGCAGGTATCCAGATATCCCC
TTCCCTGTCCCTGACCCAGAGCTCTGGTCACTACAGACCTTCGGGCTCTCCGAGCCTGTCCGCGTATATTCCA
AATAAATATCAGACTTAAGGAAGGAGGGGTAGGTGTTTTCCTCCATAGTGGCCTGGATTTCTGTCTGGGCCTG
GTCAAACATGGCAGGATCGATCAGCTGCTTCATGATGCAGCCCTTTATGAAGCTCTTGGTGGCCGGCTTGGTC
TGCCGGGACACAATGCCATTGTTATCGAGGATGTACTTTCGGTAGATGGCTCTGGCCAGCTTCAGCCTCTTCT
CCTCATTCGAGTCACAGGGCTCCAGCTTCCTGAAGCCAGTGCAGGCAAACCAGAAGTCCAGCAAGTCAGCACA
GCCCTCCTGCTTCAGGAAAGTCCTGAACAGGCTTATCCCATCTTGATCATCCAGCAGGGAGTGCAGTGACTCA
GCCCACTTCAAGTACGGTGGGGTGGGGGAGGCACTGCCCTCAGGCTCATACCCCAGGTCCAGATCCGAGCGTC
TCGGAGTGGCGGTCGAAGTCTCACCTTTAATGCCAACACCTTTCCCGGAGCAGAAACTGTAGCTGGCGGGCCT
CGGGTCTGTGGACACCAGTTCTCCCTCCTCACCAGGCACTGGGGGTCGGGGAGCATCTTCGGTGAAACTTGCT
CCAAGGTCCAAGGGGAAACCCTGCTCCTGGACATTCATTTGGGACTCTGTGCGTCAATGCACAATGAGCGCT
GCACCCTAATACATCAGTACTTAACAGCTCCAAAGTCAATCAATCTGTCCTGTTGAAACCAT?????CTAAAA
TGTTAACTAATTTTAAGTGAACAA-A--
ATTACATGGATAAACTTATGGCAGCGTGAAATAGGTATAATTTACAGTGGAGCAGGATAAGT-
ACTTAGGTTATTTATAGGACAGATTATTTTTTTGTGTTGTTTTG----CTTCTTTG-
CATATTAAAATTTATTTATAGGCGTAGTTAAGCTTT--ACTATTAGTTAGCATAAATTTGGCTTGGAGTGA--
--
CCAAAGCACAAATGTTGGACTTCATTTTTATGAGTTATTTCCTACACATTTCTAAATTGATGTAGCTTTGTCA
CTGCTTGATTTTTTTTTTTTT---
TAACTGGAGGGGGTGAAATTAGTTTATACCGCATCAAACCAGTTGCTCTTAATTAGTA---------------
----CATTACCAACATTGAAAAGAAGTAAAAGGAATGTTAATAACTTAGGAGTATTTGCGTAGTTA-
GTTTCAGAACACAAGCCTTAAATTAAT-GAGGAATGCG---------
GAAGTTTGATTTAAGCACTCATACTCCTTTATTTTCCTTTGTTAGCAATAATTATTGGCCCTGATGGCCATCC
TTTGACTGTTTATCCTTGTATGATTTGTGGGAAGAAGTTTAAGTCGAGAGGTTTTTTGAAAAGACACATGAAA
AACCATCCCGAACATCTTGCCAAGAAGAAGTACCACTGTACTGACTGTGATTACACTACCAACAAGAAGATAA
GTTTACACAACCACCTGGAAAGCCACAAACTGACCAGCAAGGCAGAGAAGGCCATTGAATGTGAGGAGTGTGG
AAAGCATTTTTCTCATGCAGGGGCTTTGTTTACTCACAAAATGGTGCACAAAGAAAAAGGAGCCAACAAAATG
CACAAGTGTAAATTCTGTGAATACGAGACAGCTGAACCGGG????????????????????????????????
??????????????????ACCCGCTATAGCTTCTGCAAACAGTAAAGATACCACTAAAACCCCTTCTGCAGT
TCAAACTAGCACCAGCCACAACCCTGGACTTCCTCCTAATTTTAACGAATGGTACGTCTGAGGACAAACACAA
ACCCTGTTAACTATAGAATGGACCAAATGCATTTTTAAAAGAAAACTGAGACCAATCAGATGGAAATGGAGTT
TTAAGGCAAGAGGCCATATATAGGGCTAAATCTTGTTAATTGCAATTGTCCAGGAAGGTTTTGGGCAAGATCC
AAAAGTAGCCATGCCCTTTTCTCAGGATTAGAAAATATGTTTTGGCGTTTGAAGGATTTTTT--
AAAAAATCTTTACACTGCTTTTTCTTCCCCTTTCTCTTGCTCTCTGCACACCCCATTCTTAAACTCCTGCAAT
TCATTTTAACACTTGTCCTGTTTCTTGAGAGGAAGTTATAGAAGGCTTGTTGGTGGTGGTGATGTTAAACTGA
TGGAAATTCTTTTTCGCCTTAGTGGTGATTGTTTAAACTCTCACAGTCTTAAACCGTGCCAAAGTCCTGTTAT
?
Brachyteles
???TACTACAGAAATGTCACCTTCCTTTCATGCCG
GTTCCCTT-
CCGTCATGAGCATGGACTACATGGTATACTTCAGCTTCTTCACCTGGATTTTCATTCCCCTGGTCATCATGTG
TGC--AATCTATCTTGACATCTTTTACAT-
CATCCGGAACAAACTCAGTCAGAACTTCTCTAACTCCAAAGAGACAGGTGCATT---
TTACGGACGGGAGTTCAAGACGGCGAAGTCCCTGTTTCTGGTGCTCTTCTTGTTTGCTTTGTCATGGCTGCCT
TTGTCCATCATCAACTGCATCACCTACTTTCA--------------TGGTGAG-
TCCCACAGCTTGTGCTGTACTT??AT
TTTACCTCTACCTATGTTCATAAACCGCTGATTATTTTCAACCAACCACAAAGACATCGGAACACTATACCTG
CTATTTGGCGCATGAGCAGGAGCAGTAGGAACAGCCCTAAGTCTCCTAATTCGAGCGGAACTCGGTCAACCAG
GAAGTCTAATAGAAGACGACCATATTTATAATGTTATTGTCACCTCCCACGCATTTATCATAATCTTCTTCAT
AGT---AATGCCAATTATAATCGGGGGATTTGG---

AAACTGACTTGTCCCACTAATAATTGGCGCCCCCGACATGGCATTCCCACGAATAAATAATATAAGCTTCTGA
CTTCTACCTCCA--TCCCTTCTACTTCTACTAG-
CATCCTCAACCTTAGAAGCTGGCGCGGGGACTGGCTGAACAGTTTACCCACCTCTAGCTGGAAATATGTCGCA
CCCAGGAGCCTCTGTAGATCTGACTATTTTTTCACTCCACCTAGCAGGTGTATCCTCTATCCTAGGGGCTATT
AACTTCATTACAACAATCATCAACATAAAACCTCCAGCCATGACTCAGTACCAGACACCTCTATTTGTCTGAT
CCGTACTTATTACAGCAGTTCTCCTACTTCTATCCCTTCCAGTATTAGCTGCCGGAATTACCATACTATTAAC
TGATC--
GCAATCTCAACACTACTTTTTTTGACCCCGCTGGTGGTGGAGACCCAATCTTGTACCAACACTTATTCTGATT
TTTTGGGCATCCCGAAGTATATATCCTCATCCTACCTGGCTTCGGAATAATCTCACATATTGTAACATACTAC
TCTAACAAAAAAGAACCATTCGGATATATAGGAATGGTGTGAGCCATAATATCTATTGGTTTCCTAGGTTTTA
TCGTATGAGCCCACCATATATT-
CACCGTAGGTATAGACGTAGACACACGAGCATACTTCACATCAGCTACCATAATTATTGCTATCCCTACTGGA
GTAAAAGTATTCAGCTGACTAGCCACACTGCACGGCGGCAATATTAAATGATCTCCCGCAATACTATGGGCTC
TGGGCTTTATCTTTCTTTTTACCGTAGGCGGGCTTACAGGAATTGTATTAGCCAACTCATCATTAGATAT-
CGTCCTGCACGACACATACTATGTAGTAGCCCACTTCCACTATGTCTTATCCATAGGGGCCG-
TATTTGCTATTATAGGAGGCTTTATTCACTGATTTCCTCTATTCTCGGGCTACACACTCGACCAAACCTATGC
TAAAATTCATTT-
TACCGTTATATTTGTAGGTGTCAACATAACCTTCTTCCCACAACACTTTCTTGGTTTATCTGGAATACCACGA
CGATATTCAGACTACCCTGATGCATACACTACATGAAATATCATCTCATCCGTAGGCTCATTCATCTCATTAA
CAGCAGTTATTCTAATAATTTTCATAATTTGAGAGGCCTTCTCCTCAAAACGAAAAGTTTTAATAGTTGAACA
ACTATCCACCAACCTAGAGTGACTCTACGGATGCCCCCCTCCTTACCACACATTTGAAGAGTCTACATATGTG
AAATCTTTAAGCGAAAAAGGAAGG????????????ATGGCACACCCAGCCCAACTAGGTTTACAAAACGCCA
CATCTCCAATCATAGAAGAACTTATCGCCTTCCACGACCATACCCTTATAATTATTTTCCTAATTAGCTCACT
AGTATTATATATTATTTCCATAATACTTACTACAAAACTCACCCACACCAGTACCATGAATGCCCAAGAGATT
GAAATCATCTGAACCATCCTACCTGCAATCATTCTTATTATAATTGCCCTGCCATCCTTACGTATTCTATATA
TAACAGATGAATTTAATAAACCCTACCTAACTCTTAAGGCAATCGGTCACCAATGGTACTGGAGCTACGAATA
CTCAGATTATGTAGATCTAGCCTTTGACTCTTACATTATACCTACATACTTTCTTGAACCTGGCGAATTTCGA
CTTCTAGAAGTAGACAATCGAACAACCTTACCCATAGAGGCAGACATCCGTATATTAATCTCATCACAAGATG
TACTACACTCATGAGCTGTCCCATCATTAGGTGTTAAAGCAGATGCTATCCCCGGACGCCTAAACCAAGCCAT
ACTAGCCTCTATACGACCAGGCCTATTTTACGGACAATGCTCAGAAATTTGCGGATCAAACCATAGCTTTATA
CCAATCGTCCTAGAATTCATCTATTTCCAAGATTTCGAAGTATGAGCCTCATACTTATATATTGTATATCCTA
ATGACCACCCCTCGCAAAACACATCCACTAGCAAAAATCATCAATAACTCACTAATCGACCTACCCTCACCAT
CCAATATCTCTGCCTGATGAAATTTCGGCTCACTACTAGGCATCTGCTTAGTCATACAAATTGCCACAGGCCT
ATTCCTAGCCATACACTACACACCAGATACCTCAACTGCTTTCTCCTCAGTCGCCCATATCACCCGGGACGTT
AACTACGGCTGAATAATCCGCTACCTACACGCCAATGGCGCTTCCATATTCTTTATCTGCCTCTTCCTCCATG
TAGGCCGAGGCATGTACTACGGATCCTTCCTTTCCCTGAAGACCTGAAACATCGGTATTATCCTACTGCTTAT
AACAATAGCCACAGCATTCATAGGGTACGTACTCCCGTGAGGCCAAATATCATTTTGAGGGGCTACAGTAATC
ACAAACCTCCTATCAGCCATTCCCTACATTGGATCAGACCTCGTACAATGAATCTGAGGCGGCTTCTCGGTAG
ATAAAGCCACACTCACACGATTTTTTACCTTCCACTTTATTCTACCCTTTATTATCGCAGCCCTAGTGACTAT
CCACCTCTTGTTTCTGCATGACACAGGATCAAGTAACCCATCAGGAATAACATCAGACCCTGACAAAATCACA
TTCCACCCCTACTACACAATCAAAGACATCTTAGGCTTTATCTTCCTTATCCTATGCCTGATAAGCCTGACCT
TATTCTCACCTGACTTTCTAACCGACCCAGACAATTACACACTAGCCAACCCCCTCAACACCCCACCTCACAT
CAAACCAGAATGATACTTCCTATTCGCATACGCAATCCTACGATCTATCCCTAACAAACTGGGAGGAGTCCTA
GCCTTAGCACTATCTATCCTAATCCTAGTAACACTACCTACTCTACATATATCCAAGCAACAAAGCATAAAAT
TCCGACCTATTGCCCAATTTTTATTCTGAGCCCTAGCAGCCGACCTTCTAACACTCACATGAATCGGAGGACA
ACCAGTTGAATACCCATTTGTAGCCATCGGCCAAACCGCATCAGTAATATACTTTCTTATTATCATCACACTT
ATTCCCCTCTCTACCCTAATTGAGAATAAACTACTTAAGTGATAG?????????AATTTATTCAAGGGAATGA
GAATTTATGGGTATAACTGTTGGCTGGAAAGCCCTTCCTCACCTCCAATCCTCCTTTCAAACTACATTTCC-
TCCTGAAATTTCTCTGTGGGATGAAATGTTCAATTATTAGTCAATCAGTC----
TCTTATACGTTATCTGGCTTTACATAT----
GCCCTTGATAGTTCTTGGAAAATAACCCAATATTGTGTTGTTTTGGTGTCTTTGTTTTGGAAATGGAAGCATT
TTGTATGAATCACCTCTTTAACTGTGAGGAGTTGAATTGTCTTAGTTCTGCTTGCCTAATGACCAGAAATAAA
--GACATCACAGTCTCTAACCCCACTTTTT-
GGTGGAAAGAAAATCTAAAAGTAGCAACATTTAATGGAACAAATTATCAATAATCAAAGAAACTCCCCTGTGA

AACCGTAATATTATTTT-
AAAATCAACATTGTTCTAAGGTAGCTGAAATGTGTGATTTTTCTATTTTGCCTTTTCACTGACAATATACCCA
TAAATCT-
TGGTGGTGATTTTCTCTCCAAAGTTAATTTCTGGACCATTGGTGACTGCACTATCTATTGTTCTAAATTGTTG
TCTTAATTGCATTCTTTAAAAATGTAATGTT-
AAGGCACATAATATTGAAGATGACTCCAGGATGAACCCTGAGTTCGCAGACCGAA-
TAAAACAGCTCGATAAAGAGGCATCTTACTACCGTGATGAGTGTGGCAAGGCCCAAGCGGAAGT?????????
???
???
???
???
???
???
???
???
???
???
???TTGATGAACTAAAAATGGTTCCTG
AAGGTCAGACTCATGTACAGTGAATGTTCTCCACTGTTGTGGTAGGCATGTTTCATGTGTGGGGATGTTGGTG
TGCAAGTTTCATGTGTTTGGAAAACACTAGCTAGAACATTTGAAAAATTTCATTAACCCAGCTCTGGAAGGTC
ATCTATGAATTCTTCCTATTCCAGTGGCCTTTT-
AAGTGTAGATGTAGATACACTGTAATTTAAAACAGATGGGCATGTCTGAATATTTTCATATGCAAAATGGGAC
AGATGTATATGAATGGATAAATTATCCTTTTAAAAAGATACTTTGTAGCACGAAATACCACCAATGACTGGAA
CAGTGAACCTGCATTCAGTGTAGGGCAAACACCAGGAAGACATTATCAAAACATCTAA-
CTATCTATAAACTAATTCCAATTAGTCTACTTTGTCTTCATACAGAAAAAA-ATTTTT-GATTTTTAAA---
TATGCATTGGGTGCCAATCCGTACCCTCT-
GAGCTGCTAAGGCCAGCATATCACAATAGAAGTTTACTTTTCC??????????????GTGTTTATAATCAATT
TATTGCTATTAATTATCCCTGCCCTAATCGCTATAGCATTCTTAACACTCATAGAACGAAAAATCCTAGGCTA
CATACAATTTCGCAAAGGCCCTAACATTGTAGGCCCCTACGGAACGCTACAACCAATCGCCGACGCAATAAAG
CTCTTTACAAAAGAACCCCTACTACCCTCTACATCCGCCACAACCCTATATATAACTGCCCCAACCCTAGCCC
TCTCCATCGCTCTCCTCCTATGAAGCCCCCTCCCAATACCACACCCCCTAATTAACTTTAACCTAGGCCTCCT
ATTTATATTAGCAACATCAAGCCTGGCCGTATATTCAATTCTATGGTCCGGATGAGCATCCAACTCAAACTAC
GCACTAATCGGAGCATTACGAGCAGTAGCCCAAACAATCTCATATGAAGTAACCCTAGCTATTATTCTGCTAT
CAACCCTACTAATAAGTGGCTCATTCAACCTACGTTCACTAATTACAACACAAGAACACTCATGACTTTTATT
ACCATCATGGCCTCTAGCAATAATATGGTTCACCTCCACACTAGCAGAAACTAACCGAGCCCCATTTGATCTC
ACAGAAGGCGAGTCAGAACTCGTCTCGGGGTTTAACATTGAATATGCTGCAGGCTCATTCGCCCTATTTTTCA
TAGCAGAGTACATAAATATTATTATAATAAATGCCCTAACCACCACTATCTTTTTAGCCACACCCTACAACAT
AACAGCATCAGAATTCTATACAATAAATTTTATAGTCAAAACCCTCCTATTGACCACCCTATTTCTATGAATC
CGAACAGCATACCCTCGATTTCGCTACGACCAACTAATACACCTTCTATGAAAAAACTTCTTACCACTTACGC
TAGCACTATGTATATGATACATTTCAATACCAACCCTAATATCCGGCATCCCACCCCAAACATA?ATTAACCC
ACCAGCCCATCTCATTATTTCCCTCACCGTCATAATAGGAACCATAATCACAATCCTAAGTTCACATTGATTC
CTCATCTGGATAGGCCTAGAATTAAACATATTAGCTATCGTACCCATACTAATAAAAAATACAAGCCCTCGCT
CTACAGAAGCAGCCACTAAGTATTTTATAACACAAGCAACTGCATCTATAATACTACTAATAGCCATCTACCT
CAACAACCTATTTTCCGGACAATGAACAATTAACCCATCCTTAAACCAAGTGCTAGCTACAATAATATTAATT
GCCCTAACAATAAAATTAGGAATAGCCCCCCTACACTTCTGACTCCCAGAAGTAACCCAAGGTACCCCCCTAA
TTCCCGCCATACTTATTCTCACATGACAAAAACTAGCCCCCCTATCAATCATATTACAAATCTTCCCATCAAT
CAACATATATATCCTCTTGACAATCTCAATTCTATCTATCATGATCGGAAGCTGGGGAGGACTAAACCAAACA
CAACTGCGCAAAATCCTGGCCTATTCATCAATCACCCATATAGGATGAATAATAGCAGTACTATACTACGACC
CCAACATTACCACCCTAGCCCTACTAATTTACATCTTATTAACAATCTCCACATTCATAACCTTCTATTTAAG
CTCAAACATAACAACCCTATCACTATCCCATACCTGAAATAAAATCACATGAATTATACCCATAATTCCACTA
ATAATAATATCACTAGGAGGCCTGCCCCCATTAACAGGTTTCTCTCCTAAATTTGCTATCATACAAGAACTCA
CAAAAAACAATAATCTCATCATACCTGTTACCATAGCCATACTAACACTAATTAACCTATACTTTTATATACG
CCTAACATATGCCATCTCAATAACAATATTCCCCACATCTAACAACACAAAAATTAACTGACAACTAAAACAC
ATAAAGCCAGCTCCATTTCTCCCACCACTAATAGTCTCCTCCACCTTCCTACTACCCATAACCCCATTAATAC

TAATAATTT???????TGGCAGCATAAATAATAAAGCATGTTACTTAAGCAATTTTACATTTACTGTATTAGT
AAGTAAATGCAATGGCCACTCTTATTCTCAAACAATAAAAACTACCCAGCCTTTTCATGAAAGAT-
GATTGCAATTTCCATTGGCCTGTATATGCAATAAAAAG---
CCATAACTCATCAAAATTTTAGCTATTTTCTCTTAAGATA--
GAAGAGCATTTTGCTTGAACATTGAACGTGACATTAAATATT-
CATGAAACTGAATAGAGTTTAAGTACTAAG---
TAAAACTCTCAAATTCTTTTCAACTAGATTGAAATTCTGTTTGAACCAGGTAAAAACTACCGCTAAGCTGAAG
AGGTGTTCAAAAAC-
ATCGTAAAGAGATTGTACAATACGTTCAAAGTGCTACAGTTATTTGGGATAGATTTTTCTGAAAAGCCTGTTC
CTCCTTTGAAATTGATTCATGATAACAGCCTCCATTTGGGTCAAACAGTATTTAACCGCTTGTAAG-
TTTTAATT-
AAATGATTTGTCACTTTGAACACATAGTTT???????????CTTGAGGATTACTTGAATGGGCCCTTCACTGT
GGTTGTGAAGGAGTCTTGTGATGGAATGGGAGATGTGAGTGAGAAGCATGGGAGTGGGCCAGTAGTCCCGGAA
AAGGCAGTCCGTTTTTCATTCACAATCATGAAAATTACTATAGCCCACAACTCTCAGAACCTGAAAGTGTTTG
AAGAAACCAAACCTAATTCTGAACTGTGTTGCAAGCCATTGTGCCTTATGCTGGCAGATGAGTCTGACCACGA
GACCCTGACTGCCATCCTGAGTCCTCTCATTGCCGAGAGGGAGGCCATGAAGAGCAGTGAATTAATGCTTGAG
ATGGGAGGCATTCTCCGGACTTTCAAGTTCATTTTCAGGGGCACTGGATATGATGAAAAGCTTGTACGGGAAG
TGGAAGGCCTTGAAGCTTCTGGCTCAGTCTACATCTGTACTCTTTGTGATGCCACCCGTCTGGAAGCCTCTCA
AAATCTTGTCTTCCACTCTATAACCAGAAGCCATGCCGAGAACCTGGAACGTTATGAGGTCTGGCGTTCCAAC
CCTTACCATGAGTCTGTGGAAGAACTGCGGGATCGGGTGAAAGGGGTCTCAGCCAAACCTTTCATTGAGACAG
TCCCTTCCATAGATGCACTCCACTGTGACATTGGCAATGCAGCTGAGTTCTACAAGATCTTCCAGCTAGAGAT
AGGGGAGGTGTATAAGAATCCCAATGTTTCCAAAGAGGAAAGGAAAAGGTGGCAGGCCACATTGGACAAGCAT
CTCCGGAAGAAGATGAACCTCAAACCAATCATGAGGATGAATGGCAACTTTGCCAGGAAGCTCATGACCAAAG
AGACTGTGGATGCGGTTTGTGAGTTAATTCCTTCTGAGGAGAGGCACGAGGCTCTGAGGGAGCTGATGGATCT
TTACCTGAAGATGAAACCAGTATGGCGATCATCATGCCCTGCTAAAGA--GTGCCC-
AGAATCCCTCTGCCAGTACAGTTTCAATTCACAGCGTTTTGCTGAGCTCCTTTCTACCAAGTTCAAGTATAGG
TATGAGGGGAAAATCACCAAT????????????????????????????GCACCCGACAGTTGTCATTATGTT
TGGAAACACTGCCGGCCCATGGAGATGAGGATAAAGTGGACATTTCTCTGCTGCATCAGGGTGAGAATCTTTT
TGAACTGCACATCCACCAGGCCTTCCTGACATCTGCCGCCCTGGCTCAGGCTGGAGACACCCAACCTACCACT
TTCTGCACCTATTCTTTCTATGACTTTGAAACCCATTGTACCCCATTATCTGTGGGRCCACAGCCCCTCTACG
ACTTCACCTCCCAGTATGTGGTGGAGACAGACTCCCTTTTCTTACACTACCTTCAAGSGGCTTCAGCCCGGCT
TGATCTCCACCAGGCTGTGGCCAGTGAGCACAACACTCTTGCTGCAGGATGGATTTGCTTTGACAGGGTGCTA
GAGACTGTGGAGAAAGTCCATGGCTTGGCCACACTGATTGGTAAGTGCTGTCGGCTTCCCACAGCTCCTA-
GCACCAATGCAGAATTTCCCAAACTCGATAGCTGCTTTTCTGGTCGTTTCTTATTTTCTTCCTTTTTCTTTGT
TCTTGATCCTTGCCACACCATCTGTATTCTCCATGCTTTCA-
TACTTGCTGCTGCTCAGGAGCTGGTGGAGAAGAGTTTGGGGTTCTAGAGTACTGGATGAGGTTGCGTTTCCCA
ATAAAACCCAGCCTACAGGCATGCAATAAACGAA??????????????AGCACTTGCACATTCAAAGGCAGCT
TGGAGTCTGAAAAGTATCAATACATCATCCATGGAGGGAAAACACCAAACAATGAGCTTTCAGATAAGATTTA
TGTCATGTCTGTTGTTTCCAAGAACAACAAGAAGGTTACTTTTCGCTGCACAGAGAAAGACTTGGTAGGAGAT
GTTCCTGAAGCCAGATATGGTCATTCCATTAATGTGGTATATAGCCGAGGGAAAAGTATGGGTGTTCTTTTTG
GAGGACGCTCATACATGCCTTCTACCCACAGAACCACAGAAAAATGGAATAGTGTAGCTGACTGCCTGCCCCA
TGTTTTCCTGGTGGATTTTGAATTTGGTTGTGCTACATCATACATTCTTCCAGAACTTCAGGATGGGCTATCT
TTTCATGTCTCTATTGCCAAAAATGACACCATCTATATTTAGGAGGACATTCACTTGCCAATAATATCCGCC
CTGCCAACCTGTACAGAATAAGGGTCGATCTTCCCCTGGGTAGCCCAGCTGT-
GAATTGCACAGTCTTGCCAGGAGGAATCTCTGTCTCCAGTGCAATCCTGACTCAAACTAACAATGATGAATTT
GTTATTGTTGGTGGATATCAGCTTGAAAATCAAAAAAGAATGATCTGCAACATCATCTCTTTAGAGGAAAACA
GGATAGAAATTCGTGAGATGGAGACCCCAGATTGACCCAGACATAGCCGATGTGTCTCAGAATCTTCCTGCTC
TCCACCACACTGAGCCCACCATGACCCAGTGCTCCACAAACATATGTAGAATGGAAGTAAAACCTCAGGGATA
AGAAAAGGTAATTAAAGGGCTGGGCGCGGTGGTTCATGCCTGTAATCCCAGCACT-
TAGGAGGCCGAGGCGAGTGGATCACAAGGTCAGGAGTTCAAGACCAGCCTGGCCAAGATGGTGAAAACCCATC
TCTACTAAAAATA---CAACAATT-----
AGCTGGG---
---------------------CGGGGTGGTGGGCGCCTGTAATCCCAGCTCCTTGGGAGGCTGAAGCAG-
AGAATTGCTTGAACCTGGGAGGCAGAGGTTGCACTGAGCCGAGATCACGCCACTGCACTCCAGCCTGGACAAC

```
A-GAATGAGACTCCATCTCAA----------------------------------------------------
-------------------------------------------------------------------------
-----------------------------------------------------AAAA-
TAATAATAAAAAGAAAAGATAGTTAAAGCAGATTCAATACAGTAACAAACCAGCCAAGAAATTGCAGTGAAAG
TAAAGGTTT-
GGATGACAAAATTTCCAACTGTGTTCAAAGAAACATAAGTTCCAGCAGGAGTGAACGTAATCTGATTTT-
CTTCAAAGC--
CTCTCTAATTTGTAGGGAAGGACATCCATTTTCATTAGCTTCATGTGAGCTAGAGAGAATCCATGATTGAAGT
GCTAAAACCCCTGGTAAACATGACAGCAAGCAAGGGCACCTTCCCCAGAGGCTCAACCTAGAATATAAGACTG
TCGGCATTGGTAAGGAGCCTTGAACTCTAAACCCATAGGCTCAAGTCAGGAA-
CTCAGGAAGCCTATCACCACCGGGATACAATTTTGCCACCTT???????????????????????????????
?????????????????????????????????????????????????????????????????????????
?????????????????????????????????????????????????????????????????????????
?????????????????????????????????????????????????????????????????????????
?????????????????????????????????????????????????????????????????????????
?????????????????????????????????????????????????????????????????????????
?????????????????????????????????????????CAAAGATCTTTATTGCCATAAAGCAGAGTAAC
T-GTCTTTGACTTGGACCTTGTTTACTGTGAACTCTAATC-AAGGCAGGACACTGAGCAGTTCTACTTA-
CTGCACTGTGGACTTGTGGAAAGGACACTTGTGA---------
ATCCAGTTTGGATGCGTGTTTGAAATTGAATTGCGAATGGTGTTCCAGGATGTGGAATGCAGCCGTGATCGCA
CTTGCCTACC-GAGCTTCCTTTGATCTGTTTGTCAATAGCATGCAAAAAA-
TGCTTTGTCTGCCCTTTGCTTCTGCTTTTTTCAGGGAAGCTGCCAAGGAATGTCGACGTCGAAAGAAAGAATA
CGTAAAGTGTCTGGAGAGCCGAGTTGCAGTACTGGAAGTCCAGAACAAGAAACTTATAGAGGAACTGGAAACC
TT???????????????????????????????????????????????????????????????????????
?????????????????????????????????????????????????????????????????????????
?????????????????????????????????????????????????????????????????????????
?????????????????????????????????????????????????????????????????????????
?????????????????????????????????????????????????????????????????????????
?????????????????????????????????????????????????????????????????????????
?????????????????????????????????????????????????????????????????????????
?????????????????????????????????????????????????????????????????????????
?????????????????????????????????????????????????????????????????????????
???????????????????????????????????????????????????ATATCTAAATGTGTGGATTCCA
GCACCTAAACCAAAAAATGCCACTGTAATGATATGG-
ATTTATGGTGGTGGTTTTCAAACTGGAACATCATCCTTACATGTTTATGATGGCAAGTTTCTTGCTCGGGTCG
AAAGAGTTATTGTAGTGTCAATGAACTACAGGGTGGGTGCCCTAGGATTCTTAGCTTTGCCAGGAAATCCTGA
GGCTCCAGGAAACATGGGTTTATTTGATCAACGGTTGGCTCTTCAGTGGATTCAAAAAAATATAGCAGCCTTT
GGTGGAAATCCTAAAAGTGTGACTCTTTTTGGAGAAAGTGCAGGAGCAGCTTCAGTTAGTCTGCATTTGCTTT
CTCCTGGAAGTCATTCCTTGTTCACCAGAGCCATTCTGCAAAGTGGATCCTCTAATGCTCCCTGGGCAGTAAC
ATCTCTTTATGAAGCTAGGAACAGAACGTTGGCCTTAGCTAAATTTACTGGTTGCTCTAGAGAAAATGAGACT
GAAATAGTCAAATGTCTTCAAAACAAAGATCCCCAAGAAATTCTTCTGAATGAAGCATTTGTTGTCCCCTACG
GAACTCTCTTGTCAGTAAACTTTGGTCCAACAGTGGATGGTGATTTTCTCACTGACATGCCAGACATATTACT
TGAACTTGGACAATTTAAAAAAACCGAGATTTTGGTGGGTGTTAATAAAGATGAAGGGACAGCTTTTTTAGTC
TATGGTGCTCCTGGCTTCAGCAAAGATAACAATAGTATCATAACTAGAAAAGAATTTCAGGAAGGTTTAAAAA
TATTTTTTCCAGGAGTGAGTGAGTTTGGAAAAGAATCCATCCTTTTTCATTACACAGACTGGGTAGATGATCA
GAGACCTGAAAACTACCGTGAGGCCTTGGATGATGTTGTTGGGGATTACAATATTATATGCCCGGCCTTGGAG
TTTACCAAGAAGTTCTCAGAATGGGAAACAATGCCTTTTTCTACTATTTGA???????TTGTGCTAATTGTG
AGAAAATATGCTACTTTGATTAAACTAGAATGAAGGAAATAAAAAGACACTGTGGCTCAACTTGTAATAAAGA
GCAGCTTATCCTCACAGCTTTTTGATTGTTGCTATAGTTATCT---
ATTCAACATGGGATGCACAATCCAGTTAACGTTTCATTACCAGTAGCCTGTTGCGTGCTTATTGTTAAAAGGA
ATTTGACATACAAGTTTGTGTATGGATTGTTCGCCTAATTCCGATTCCTCGTGCTGACTGGCATGAGACAGGG
AGCTGGATTGTATTAAACTGGTTTTATTGTGATGTGACTATGATGGATGTGACAAGGGCCTCTATTATTCGCT
GGGGAGTGTGGCCGTTAATGAAGCCATTAGCTGAGCTAAGGCAAGTGAAGAAAATAGGGAAATTACCATTATT
CTGAAGCCATTAAGTTGTAATCCACCACTTTCTCTCCTTATTCAAAAGGCATCTTCTTTTTAA?????????
```

???
???
???
???
???
???
???
???
???
???
???
???
???
???
???
???
???
???
????????????????????????????????TAAGCCACTTCCAGCCCTGCTGTGAACCAGGACTGTAGGAG
CCATTTTTTTCATGTGGTCAGCTTTGGAAGCATCTACATCCACCACTTTAGAAGACAAGTCTAGTGGCTTATC
TGTGACGTCTTTGGTAACGGTCTGCTTCTCCAACAGAGGAGGTGAGCTGCCGTCTTTTCTGTCTTGAACTGCT
GTCTTCCGGGCATGCCCGGGCACTGGCTGGGCACCTTCGCCCCCTTCCAGAGCCTTGGGATACTTGCCGTTGG
AGAGCCTGGCCGCGGGGAACTCGCTGCTGACTGTCATGTATGGCTTCGACAGGGCAACTGAAGGAGAGGTGGA
GATCCTGGCATAGTGCTTGTGGAACTCCGAGTAGGTATCTGCAGCAGGCTGGGTGGGAAGGTGGACCCGGGGT
GAGGGCCGAGGTGAGGGGGGCAACAGGAGAGCTGTGTCCCCCGGCAGGCCACTGGTGACTGCCTTGGCAGAGG
GAACCCTGGGCTGTTTACTGTTCTGGATGTGAGGATAGGCGTGGGAATCAACGGGATTCCCAGGGCTGACCCC
CATCTTCCACGGGAGGCTTTTGTCTGCGCAATGGACAAGAG-CGGGATGGCTGGGGAAGCCGAAG-
CGTCGAGAGCCTCATGGGTGATGCCA--GACGATGGGATGTGGGGACTGACGTAGTGAGGTGGTGGCAG-
TAGAGAAAGCGCTCCCCGTTGGTGCAGACTGGAGAATACAGCGGCTGGGCCAAGCTGTAG?????????????
???
???
???
???
???
???
???
??CAGCTGTCTCCAG
AAGCAGCTTCTGAGGGAGTCTTCCAGGAGGAGCAGTCTCTCTGCCATCGTCCTCGTCCACGTCCTGGTCACAT
TTCCATTCCTCATCTTCGTTTAAAGTCGGCAGGTATCCAGATATCCCCTTCCCTGTCCCTGACCCAGAGCTCT
GGTCACTACAGACCTTCGGGCTCTCCGAGCCTGTCCTCGTATATTCCAAATAAATATCWGACTTAAGGAAGGA
GGGGTAGGTGTTTTCCTCCATAGTGGCCTGGATTTCTGTCTGGGCCTGGTCAAACATGGCAGGATCGATCAGC
TGCTTCATGATGCAGCCCTTTATGAAGCTCTTGGTGGCCGGCTTGGTCTGCCGGGACACGATGCCATTGTTAT
CAAGGATGTACTTTCGGTAGATGGCTCTGGCCAGCTTCAGCCTCTTCTCCTCATTCGAGTCACAGGGCTCCAG
CTTCCTGAAGCCAGTGCAGGCAAACCAGAAGTCCAGCAAGTCAGCACAGCCCTCCTGCTTCAGGAAAGTCCTG
AACAGGCTTATTCCATCTTGATCATCCAGCAGGGAGTGCAGTGACTCAGCCCACTTCAAGTACGGTGGGGTGG
GGGAGGCACTGCCCTCAGGCTCATACCCCAGGTCCAGATCCGAGCGTCTCGGAGTGGCAGTCGAAGTCTCACC
TTTAATGCCAACACCTTTCCCGGAGCAGAAACTGTAGCTGGCGGGCCTCGGGTCTGTGGACACCAGTTCTCCC
TCCTCACCAGGCACTGGGGGTCGGGGAGCATCTTCGGTGAAACTTGCTCCAAGGTCCAAGGGGAAACCCTGCT
CCTGGACATTCATTTTGGGACT???
??CTAAAATGTTAACTAATTTTAAGTGAACAA-
A--ATTACATGGATAAACTTATGGCAGCGTGAAACAGGTATAATTTACAGTGGAGCAGGATAAGT-
ACTTAGGTTATTTATAGGACAGATTATTTTTTTGTGTTGTTTTG----CTTCTTTG-
CATATTAAAATTTATTTATAGGTATAGTTAAGCTTT--ACTATTAGTTAGCATAAATTTGGCTTGGAGTCA--
--

CCAAAGCAGAAATGTTGGACTTCATTTTTATGAGTTATTTCCTACACATTTCTAAATTGATGTAGCTTTGTCA
CTGCTTGATTGTTTTTTTTT---
AAACTGGAGGGGGTGAAATTAGTTTATACTGAGTCAAACCAGTTGCTCTTAATTAGCA---------------
----CATTGCCAACATTGAAAAGAAGTAAAAGGAATGTTAATAACTTAGGAGTATTTGCGTAGTTA-
TGTTCAGAACACAAGCTTTAAATTAAT-GAGGAATCCA---------
GAAGTTTGATTTAAGCACTCATGGTCCTTTATTTTCCTTTGTTAGCAATAATTATTGGCCCTGATGGCCATCC
TTTGACTGTTTATCCTTGTATGATTTGTGGGAAGAAGTTTAAGTCGAGAGGTTTTTTGAAAAGACACATGAAA
AACCATCCCGAACATCTTACCAAGA---
AGTACCACTGTACTGACTGTGATTACACTACCAACAAGAAGATAAGTTTACACAACCACCTGGAAAGCCACAA
ACTGACCAGCAAGGCAGAGAAGGCCGTTGAATGTGATGAGTGTGCGAAGCATTTTTCTCATGCAGGGGCTTTG
TTTACTGACAAAATGGTGCACAAAGAAAAAGGAGCCAACAAAATGCACAAGTGTAAATTCTGTGAATACGAGA
CAGCTGAACCAGG???
????TCTGCAAACAGTAAAGATACCACTAAAACCCCTTCTGCAGTTCAAACTAGCACCAGCCACAACCCTGGA
CTTCCTCCTAATTTTAACGAATGGTACGTCTGAGGACAAACACAAACCCTGTTAACTATAGAATGGACCAAAT
GCATTTTTAAAAGAAAACTGAGACCAATCAGATGGAAATGGAGTTTTAAGGCAAGAGGCCATATATAGGGCTA
CATCTTGTTAATTGCAATTGTCCAGGAAGGTTTTGGGCAAGATCCAAAAGTAGCTATGCCCTTTTCTCAGGAT
TAGAAAATATGTTTTGGCGTTTGAAGGATTTTTT--
ACAAAATCTTTACACTGCTTTTTCTTCCCCTTCCTCTTGCTCTCTGCACACCCCATTCTTAAACTCCTGCAAT
TCATTTTAACACTTATCCTGTTTCTTGAGAGGAAGTTATAGAAGGCTTGTTGGTGGTGGTGATGTTAAACTGA
TGGAAATTCTTTTTCGCC???
?
Cacajao
?TACCCCCATGTTTGGCTGGAACATGAAGCTGACCTCAGAGTACTACAGAAATGTCACCTTCATTTCATGCCG
GTTCCCTT-
CCGTCATGAGCATGGACTACATGGTATACTTCAGCTTCTTCACCTGGATTTTCATTCCCCTGGTCATCATGTG
TGC--AATCTATCTTGACATCTTTTACAT-
CATCCGGAACAAACTCAGTCAGAACTTCTCTAACTCCAAAGAGACAGGTGCATT---
TTATGGACGGGAGTTCAAGACGGCGAAGTCCCTGTTTCTGGTGCTTTTCCTGTTTGCTTTGTCATGGCTGCCT
TTATCCATCATCAACTGCATCACCTACTTTCA--------------
TGGTGAGGTACCACAGCTTGTGCTGTACTTGGGCATCCTGCTGTCCCATGCCAACTCCATGATGAACCCTATC
?????????????????????ATGTTCATAAATCGCTGACTATTTTCAACCAACCACAAAGACATTGGAACAC
TATACTTATTGTTCGGTGCCTGAGCAGGAGCAGTGGGAACAGCTCTAAGCCTCCTGATCCGAGCAGAATTAGG
CCAACCAGGAAGTCTAATAGAAGATGACCATGTCTATAACGTCATTGTTACCTCTCACGCATTTATTATAATT
TTCTTCATAGT---AATGCCAATTATAATTGGCGGGTTCGG---
AAACTGACTCGTCCCCCTTATAATTGGCGCCCCTGACATAGCATTCCCCCGAATAAACAACATAAGCTTTTGA
CTACTTCCCCCA--TCACTTCTTCTTCTACTTG-
CATCATCAACTCTAGAAGCAGGTGCTGGAACTGGTTGAACTGTATATCCACCACTAGCGGGAAACATGTCACA
CCCGGGAGCCTCCGTAGACCTAACTATTTTTTCACTCCACTTAGCAGGTATTTCCTCTATCCTAGGGGCCATT
AACTTTATTACAACAATTATTAACATAAAACCCCCAGCTATAACCCAGTATCAAACACCTCTATTCGTCTGAT
CTGTACTTATTACAGCTGTTCTCCTCCTTCTCTCCCTCCCAGTCCTAGCTGCCGGAATTACCATACTATTAAC
CGATC--
GAAACCTTAACACTACTTTTTTTGACCCTGCTGGTGGCGGAGACCCAATTCTGTATCAGCACTTGTTTTGATT
TTTTGGACATCCCGAAGTATATATTCTTATTCTACCAGGCTTTGGAATAATTTCACATATTGTAACATACTAT
TCTAATAAAAAAGAACCATTCGGTTATATAGGAATAGTATGAGCTATAATGTCCATTGGTTTTCTAGGATTTA
TCGTATGAGCTCACCATATATT-
TACAGTAGGAATAGATGTAGACACCCGCGCATACTTTACATCTGCCACTATAATTATTGCTATCCCCACTGGA
GTCAAAGTCTTTAGTTGATTAGCTACATTGCATGGCGGCAACATCAAATGATCCCCCGCAATATTATGAGCCC
TAGGTTTTATTTTTCTTTTTACCGTTGGCGGATTAACAGGGATTGTTCTAGCCAACTCATCCCTAGACAT-
TGTCTTACACGATACATACTATGTAGTTGCCCACTTCCATTACGTTCTGTCCATAGGAGCAG-
TATTTGCCATCATGGGGGGATTTATTCACTGGTTTCCTCTATTCTCAGGCTACACTCTTGACCAAACTTATGC
AAAAATTCACTT-
TACTACCATATTTGTAGGCGTAAATATAACTTTTTTCCCACAACATTTCCTCGGCCTATCAGGCATGCCACGG
CGTTACTCAGATTACCCTGACGCCTATACTACATGAAATATCATTTCATCTGTGGGCTCATTTATTTCACTAA
CAGCAGTAATTCTAATAATCTTCATAATCTGAGAAGCCTTTTCCTCAAAACGTAAAGTCCTAACTATTGAACA

ACCTCACACTAATCTAGAATGACTCTACGGCTGCCCTCCTCCTTACCACACATTTGAAGAAGCTACCTACGTA
AAATTACTAAACGAAAAAGGAAGG????????????ATGGCACACCCAGCCCAGCTAGGCCTGCAAAACGCCA
CATCCCCTATTATAGAAGAACTAATTGCCTTTCATGACCACACTCTCATAATCATTTTCCTAATTAGCTCACT
AGTTCTATACACCATCTCCTTAATACTTACCACAAAATTAACCCACACTAGCACTATAAACGCCCAAGAAATT
GAAATAATCTGAACTATTCTACCCGCCATTATTCTCGTCATAATTGCTCTTCCATCCCTACGCATCCTCTACA
TAACAGATGAATTTAACAAACCATATTTAACTCTCAAAGCAATTGGTCACCAATGGTACTGAAGCTACGAGTA
CTCAGACTACGAGGACTTGGCCTTTGATTCTTATATTGTACCTACATACTTTCTCGAGCCCGGCGAATTTCGA
CTTCTTGAAGTAGACAACCGAACAACACTACCAATAGAAGCAGATATTCGTATGCTAATCTCATCACAAGATG
TACTACACTCATGAGCTGTCCCATCACTAGGCATTAAGGCAGATGCAATTCCTGGACGTTTAAACCAAGCTAT
AGTAGCCTCTATGCGACCAGGCTTATTCTATGGGCAGTGTTCAGAAATTTGTGGATCAAATCACAGCTTTATA
CCTATCGTACTAGAATTCATTTACTTCCAAGACTTTGAAGTATGAGCATCATACTTATATATTGTAT??????
ATGGCAGCCCCCCGCAAAACACATCCACTAATAAAAATTATCAACAGCTCATTCATTGACCTTCCCACACCAC
CCAATATCTCGTCCTGATGAAACTTCGGCTCACTTCTAGGCATCTGCCTAATTATTCAAATCATCACAGGCCT
ATTCTTAGCTATACATTACACATCAGATTCTTCCACTGCTTTCTCCTCAGTCGCCCATATCACCCGAGATGTA
AACTACGGCTGAATAGTGCGCTACCTACACGCCAACGGCGCCTCCCTATTCTTCATATGTCTATTTCTTCACA
TTGGACGGGGCATGTACTACGGATCCTTTCTCTTTCTAAATACATGAAACATTGGCATCATTTTATTACTTAC
AACTATAGCCACAGCATTTATAGGCTATGTACTCCCATGAGGCCAAATATCCTTCTGAGGAGCCACAGTAATC
ACAAACCTTCTATCAGCTATCCCATATATTGGACCTGACTTAGTACAATGAATCTGAGGTGGCTTTTCCGTTG
ATAAAGCCACCCTAACTCGATTTTTTACCTTCCACTTTATCTTGCCCTTCATTATTGCAGCCCTAGCATCTAT
TCACCTTCTGTTTCTGCATGACACAGGTTCAAATAATCCATCAGGACTGACATCTGATCCAGACAAAGTCTCA
TTCCACCCATACTATACAATCAAAGATATCTTAGGCTTAGTTTTCCTGCTTCTACTTTTAATAAGCCTAACCC
TATTTATACCCGATCTTTTAACAGACCCAGACAACTACACCCCAGCTAACCCCCTAAACACCCCACCACATAT
TAAACCAGAATGGTACTTTCTATTTGCATACGCAATTCTACGATCCATTCCCAATAAACTAGGAGGCGTCTTA
GCCCTTCTTCTATCCATCCTAATCCTAACAATTATCCCTATAATACATACATCCAAACAGCAAAGTATAACAT
TCCGACCCATTAGCCAGAGCCTATTCTGAGCTCTAGTAGCTGACCTATTTACACTCACATGAATTGGAGGCCA
GCCAGTTGAGTACCCTTTTATTAGCATTGGCCAAACCGCATCCACCCTATACTTTCTTATTATTTC---
TATTACTCCCCTACTCGCCCTAATTGAAAACAAACTACTTAAATGGTAG?TTAGGTTAAATTTATTCAAAGGA
ATGAGAATTTATGGGTATAGCTGTTAGCTGGAAAGCCCTTCCTCACCTCCAATCCTCCTTTCAAACTACATTT
CC-TCCTGAAATTTCTCTGTGGGATGAAATGTTCAATTATTAGTCAATCA----
GTCTGTTATACGTTATCTGGCTTTACATAT----
GCCCTTGATAGTTCTTGGAAAATAATCCAATATTGTGTTGTTTTGGTGTCTTCGTAYTGGAGATGGAAGCATT
TTGTATGAATCACCTCTTTAA-
TGTGAGGAGTTGAATTGTCTTAGTTCTGCTTGCCTAATGACCAGGAATAAAAAGACATCACAGTCTCTAACCC
CACTTTTT-
GGTGGAAAGAAAATCTAAAAGTAACAACATTTAAGGGAACAAAATATCAAKAATCAAAGAAACTCCCCTGTGA
AACTGTAATATTATTTA-
AAAATCAACATTGTTCTAAGGTAGCTGAAATGTGTGATTTTTCTATTTTGCCTTTTCACTGACAATATACCCA
TAAATCT-
TGGTGGTGATTTTCTCTCCAAAGTTAATTTCTGGGCCATTGGTGACTACACTATCTATTGTTCTAAATTGTTG
TCTTAATTGCATTCTTTAAAAATGTAATGTTGAAGGCACATAATATTGAAGATGACTCCAGGATGAACCCTGA
GTTTGCAGACCGAA-
TAAAACAGCTCGATAAAGAGGCGTCTTACTACCGTGATGAGTGTGGCAAGGCCCAAGCGGAAGTGGACCGGTT
GCTGGAGA?????????GGAAGAGCTGCATCAAGCAAGTACTTTGATCTTTCTTGGAGCAGGTTCCTTCAAAA
CGCTTCTCCAGAGGACGAAAGTCCCTCCAGTCTGGCTCGCTGCTCGCAGTGGTGGGGAAAGCTGAGGTAATTC
CCCGGTGGGAAATCTGCGAGGCCCCTTTTACCCCTG--------
CCACCAGGCCCATCACTGATGGGCCTAAGGGCCTCATCTGATACTCTAAGACAGGCTTCTCCTGTCGGATCTG
GGCCTCTCGGACTTGAGACTCTCTAGAACAAGCCTCTCGGGCCTGGACTTCTCGGGTTCTGGCCTCCCTGGCA
TGACCTTCCCAAGTGTGGGCCTCTCTG--
GCATAGGCTTCCTTGCCACGAGCTTCTCGAGTATAGGCCTCCCTGGTGTGGGCCTCCCTGGCCTGAGCGTCTC
GGGCACGTGCCTCCCGGGCCTCAAGCTGCTCCCGCCGAAGCTCCCAATACAACAACTGTTTCTGGATGGTCA-
CTAGCCGTTCCTCCTCTGTCTCCATTGCCCCAGGTGGCCGGGAGGAAGAAAAGGGCTCAAAGTTCACGAAGGG
GTCAAACATCTCAGAGCTTCTACCATGGAGGTCATAAAGGCAGTCATCCCCAGGTGGAGAGTTCTCAAGACTG
TCATCTGGCTCATAGAACTCATATAGGGCATCTCCACTGTAGCTGTCTCGGGGCAGACAATCCCTACGGACAA
GCCCCAGGGCCTCACCTGAATCATCCTCAA??????AGCATCACTAATTTTGATGAACTAAAAATGGTTCCTG

AAGGTCAGATTCATGTACAATGAATGTTCTCCACTGTTGTGGTGGACATGTTTCATGTGTGGGGATGTTGGTG
TGCAAGTTTCATGTGTTTGGAAAACACTAGCTAGAACATTCAAAAAATTTCATTAACCCAGCTCTGGAAGGTC
ATCTATGAATTCTTCCTATTCCAGTGGCCTTTT-
AAGTGTAGATGTAGATACACTGTAATTTAAAACAGATGGGCATGTCTGAATATTTTCATATGCAAAATGGGAC
AGATGTATATGAATGGATAAATTATCCTTTTAAAAAGATACTTTGTAGCACGAAATATCACCAATGATTGGAA
CAGTGAACCTGCATTCAGTCTAAGGCAAACACCAGGAAGACATTATCAAAACATCTAA-
CTAACTACAAACCAATTCCAATTAGTCTACTTTGTCTTCATACAGAAAAAA-TTTTTT-GATTTTTAAA---
TACGTATTGGGTGCCAATCCGTATCCTCT-
GAGCTGCTAAAGCCAGCATATCACAATAGAAGTTTACTTTTCCTTGAGAGTTGCATCATGCCTATAATTAACC
TCCTACTACTAATTATCCCCGCTCTCATCGCTATAGCATTCCTAACCCTCATAGAACGAAAAATCCTGGGCTA
CATACAACTCCGCAAAGGCCCTAATATCGTAGGCCCCTACGGACTGCTTCAACCAATTGCAGACGCAATAAAA
CTATTCACAAAAGAACCCCTTATACCCACCACATCTACTGTAACTCTTTATGTCGCAGCCCCAGCCCTAGCTC
TCTCCATTGCTCTCCTCTTATGAGCCCCCCTCCCCATACCATATTCCCTAATTAATCTAAACCTGGGCTTACT
ATTTATATTAGCAATATCAAGCTTAGCTGTTTACTCAATTTTATGGTCAGGATGAGCATCCAACTCAAACTAC
GCACTAATTGGTGCACTACGAGCCGTAGCCCAAACAATCTCGTATGAAGTAACATTAGCCATTATTCTTCTAT
CAGTACTACTTATAAGTGGCTCATTCAACATTCACTCGCTAATCACAACACAAGAACACTCCTGACTTCTACT
ATCATCATGACCTCTAGCTACAATATGATTTATTTCAACATTAGCTGAAACCAACCGAGCCCCATTTGACCTA
ACAGAAGGTGAATCAGAACTAGTCTCAGGCTTTAACATCGAATACGCTGCAGGCTCATTTGCCCTATTCTTCA
TAGCAGAATACATAAACATTATTATTATAAACGCCCTAACTACCACTATCTTTCTAGCAGCACCACATACCCC
AACCATGCCAGAACTATACTCAATTTATTTCATAACTAAAACTCTAATACTAACTGCCCTATTCCTATGGATT
CGAACAGCGTACCCCCGACTCCGTTATGATCAACTAATACACCTTCTATGAAAAAAATTCTTACCCCTTACAC
TAGCACTATGTATGTGATATATCTCAATACCAACCCTAACATCTAGTGTTCCACCCCAAACATA?ATTAATCC
CCTAGCACATCTCACCATCTCTTTTACAATCCTAACAGGAACAATAATTGCAATCCTAAGCTCACACTGATTC
TTCATCTGAATAGGCCTAGAACTAAATATATTATCCATTATTCCAATTCTAGCCAAAAGCACAAACCCCCGCT
CCACAGAAGCAGCCACTAAATATTTTCTCACCCAAGCAACCGCATCCATAATTCTACTAATAGCTATTATTCT
AAACAATCTATTATATGGACAATGAACAATTAACCCATCCTTAAATCAAATTTTATCCACAATAATACTAATC
GCTCTCACAATAAAATTAGGCATAGCTCCTCTTCACTTCTGACTCCCCGAAGTAGTTCAAGGTATCCCCTTAA
TCCCAGCCATACTTATCCTCACATGACAAAAATTAGCCCCAATATCAATTATTATCCAAATTTTCCCATCAAT
TAACACAAATCTCATCCTAATAATCTCAATCCTATCTATTATAGTTGGCAGTTGAGGAGGACTAAACCAAACA
CAACTACGTAAAATTTTAGCCTACTCATCAATTACCCACATGGGCTGAATAATAGCTGTCCTATACTACAACC
CAAACATTACTATCCTTAGCTTACTCATCTATCTATTTCTAACAATTTCCACATTTATAGTTTTTTACCTAAA
CTCAAATGTAACAACCCTGTCAATATCACATACCTGGAATAAACTTACATGAGTAGTACCTATAACTACATTA
ATAATATTATCCCTAGGAGGCCTACCCCCACTAACAGGTTTCTCTCCTAAATGAGCTATTATACAAGAACTGA
CAAAAAATAATAACCTTATTATCCCTCTCGTTATAGCTATACTAACACTAACAAATTTATATTTTTACATACG
CCTCACATATTCTATCTCAATAACAATATTTCCTACATCTAACAGTACAAAAATCAACTGACAATTAAAATAT
ATAAAACCATCGCCACTTCTCCCCCTACTTATAATAGCTTCTACCTCCCTTCTACCCCTAACCCCATTAATAC
TAGCAAACT?????GTTGGCAGCATAAATAATAAAGCATGTTACTTAAGCAATTTTACATTTACTGTATTAGT
AAGTAAATGCAATGGCCACTCTTATTCTCAAACAATAAAAACTACCCAGCCTTTTCATGAAAGAT-
GATTGCAATTTCCATTGGCCTGTATATGCAATAAAAAG---
CCATAACTCATCAAAATTTTAGCTATTTTCTCTTAAGATA--
GAAGAGCATTTTGCTTGAACATTGAACGTGACATTAAATATT-
CATGAAACTGAATAGAGTTTAAGTACTAAG---
TAAAACTCTCAAATTCTTTTCAACTAGATTGAAATTCTGTTTGTACCAGGTAAAAACTGCCGCTAACCTGAAG
AGGTGTTCAAAAAC-
ATCGTAAAGAGTTTGTACAGTATGTTCAAAGTGCTACAGTTATTTGGGATAGATTTTTCTGAAAAGCCTGTTC
CTCCTTTGAAATTGATTCATGATAACAGCCTCCATTTGGGTCAAACAGTATTTAACCGCTTGTAAA-
TTTTAATT-
AAATGATTTGTCACTTTGAACACATAGTTTGTCCCCAAGACCTTGAGGATTACTTGAATGGCCCCTTCACTGT
GGTTGTGAAGGAGTCTTGTGATGGAATGGGAGATGTGAGTGAGAAGCATGGGAGTGGGCCAGTAGTCCCGGAA
AAGGCAGTCCGTTTTTCATTCACAATCATGAAAATTACTATAGCCCACGACTCTCAGAACATGAAAGTGTTTG
AAGAAGCCAAACCTAACTCTGAACTGTGTTGTAAGCCATTGTGCCTTATGCTGGCAGATGAGTCTGACCATGA
GACCCTGACTGCCATCCTGAGTCCTCTCATTGCTGAGAGGGAGGCCATGAAGAGCAGTGAATTAATGCTTGAG
ATGGGAGGCATTCTCCGGACTTTCAAGTTCATCTTCAGGGGTACTGGATATGATGAAAAACTTGTACGGGAAG
TGGAAGGCCTTGAGGCTTCTGGCTCAGTCTACATCTGTACTCTTTGTGATGCCACCCGTCTGGAAGCCTCTCA

AAATCTTGTCTTCCACTCTATAACCAGAAGCCATACCGAGAACCTAGAACGTTATGAGGTCTGGCGTTCCAAC
CCTTACCATGAGTCTGTGGAAGAACTGCGGGATCGGGTGAAAGGGGTCTCAGCCAAACCTTTCATTGAGACAG
TCCCTTCCATAGACGCACTCCACTGTGACATTGGCAATGCGGCTGAGTTCTACAAGATCTTCCAGCTAGAGAT
AGGGGAGGTGTATAAGAATCCCAATGCTTCCAAAGAGGAAAGGAAAAGGTGGCAGGCCACATTGGACAAGCAT
CTCCGGAAGAAGATGAACCTCAAACCAATCATGAGGATGAATGGCAACTTTGCCAGGAAGCTCATGACCAAAG
AGACTGTGGATGTGGTTTGTGAGTTAATTCCTTCTGAGGAGAGGCATGATGCTCTGAGGGAGCTGATGGATCT
TTACCTGAAGATGAAACCAGTATGGCGGTCATCATGCCCTGCTAAAGA--GTGCCC-
AGAATCCCTCTGCCAGTACAGTTTCAATTCACAGCGTTTTGCTGAGCTCCTTTCTACCAAGTTCAAGTATAGG
TATGAAGGGAAAATCACCAATTATTTTCACAAAATAGATGTTGCTTATGGCACCCGACAGTTGTCATTATGTT
TGGAGACACTGCCAGCCCATGGAGATGAGGATAAAGTGGACATTTCTCTGCTGCATCAGGGTGAGAATCTTTT
CGAACTGCACATCCACCAGGCCTTCCTGACATCTGCCGCCCTGGCTCAGGCTGGAGACACCCAACCTACCACA
TTCTGCACCTATTCCTTCTATGACTTTGAAACCCATTGTACCCCACTGTCTGTAGGGCCACAGCCCCTCTATG
ACTTCACCTCCCAGTATGTGGTAGAGACAGACTCCCTTTTCTTACACTACCTTCAAGGGGCTTCAGCCCGGCT
TGATCTCCACCAGGCTGTGGCCAGTGAGCACAACACTCTTGCTGCAGGATGGATTTGCTTTGACAGGGTGCTA
GAGACTGTGGAGAAAGTCCATGGCTTGGCCACACTGATTGGTAAGTGCTGTCAGCTTCCTGCAGCTCCTA-
GCACCAATGCAGAATTTCCCAAACTCTATAGCTGCTTTTCTGGTCGTTTCTTATTTTCTTCCTTTCTCTTTGT
TCTTGATCCTTGCCACACCATCTGTATCCTCCATGCTTTCG-
TACTTGCTGCTGCCCAGGAGCTGGTGGAGAAGAGTTTGGGGTTCTAGAGTACTGGATGAGGCTGCGTTTCCCC
ATAAAACCCAGCCTACAGGCATGCAATAAACGAAAGAAAGCCCAGG???????????????????????????
????????????????????????????TTCATGGAGGGAAAACACCAAACAATGAGCTTTCAGATAAGATTTA
TGTCATGTCTGTTGTTTCCAAGAACAACAAAAAGGTTACTTTTCGCTGCACGGAGAAAGACTTGGTAGGAGAT
GTTCCTGAAGCCAGATATGGTCATTCCATTAATGTGGTATATAGCCGGGGGAAAAGTATGGGTGTTCTTTTTG
GAGGACGCTCATACATGCCTTCTACCCACAGAACCACAGAAAAAATGGAATAGCGTAGCTGACTGCCTGCCCCA
TGTTTTCCTGGTGGATTTTGAATTCGGGTGTGCTACATCATACATTCTTCCAGAACTTCAGGATGGGCTATCT
TTTCACGTCTCTATTGCCAAAAATGACACCATCTATATTTAGGAGGACATTCACTTGCCAATAATATCCGCC
CTGCCAACCTGTACAGAATAAGGGTTGATCTTCCCCTGGGTAGCCCAGCTGT-
GAATTGCACAGTCTTGCCAGGAGGAATCTCTGTCTCCAGTGCA????????????????????????????????
???
???TGTGTCTCAGCATCTTCCTGCTC
CCCACCACACTGAGCCCACGATGACCCAGTGCTCCACAAACATATGTAGAATGGAAGTAAAACCTCAGGGATA
AGAAAAGATAATTAAAGGGCCGGGTGCGGTGGTTCATGCCTGTAATCCC-------
TAGGAGGCTGAGGCGAGTGGATCACAAGGTCAAGAGTTCAAGATCAGCCCAGCCAAGATGGTGAAAACCCGTC
TCTACTAAAAATA---------------------------------------CAACAATT-----
AGCTGGG---
---------------------CGTGGTGGTGGGCACCTGTAATCCCAGCTACTTGGGAGCCTGAAGCAG-
AGAACTGCTTGAACCTGGGAGGCAGAGGTTGCAGTGAGCCGAGATCACGCCACTGCACTCCAGCCTGGACAAC
A-GAGTGAGACTCCATCTCAA---

---AAAA-
TAATAATAAAAAGAAAAGATAGTTAAAGAAGATTCACTACAGTAAGAAACCAGCCAAGAAATTGCACTGAAAG
TAAAGGTTT-
GGGTGACAAAATTTCCAACTGTGTTCCAAGAAACATAAGTTCCAGCAGGAGTGCACTTAATCCGATTTT-
CTTCAAAGC--
CTCTCTAATTTGTAGGGAAGGACATTCATTTTCATTAGCTTCATGTGAGCTAGAGAGACTCCATGATTGAAGC
GCTAGAACCCCTGGTAAACATGCCAGCAAGCAAGGGCACCTTCCCCAGAGGCTCAAACTAGAATATAAAACTG
TTGGCATTGGTAAGGAGCCTTGAACTCTAAACCCATAGGCTCAAGTCAGGAA-
CTCAGGAAGCCTGTCAACACCGGGATACAATTTTGCCACCTTAACACTCTATTCCCTTTACTTGTGCCAAACT
???
???
???
???
???
???CAAAGATCTTTACTGCCATAAAGCAGAGTACC
T-GTCTTTGACTTGGACCTTGTTTACTGTGAACTCTAATC-AAGGCAGGACATGCAGCAGTTCTACTTA-

CCGCACTGTGGACGTGTG---------------A---------
ATCCAGTTGGGATTAGTGTTTGAAATTGAATGGCGAATGTTGTTCCAGGATGTGGAGTGCAGCCGTGATCACA
CTTGCCTACC-GAGCTTACTTTGAACTATTTGTCAATAGCATGCAAAAAA-
TGCTGTGTCTGCCCTTTGCTTCTGCTTTTTCCAGGGAAGCTGCCAAAGAGTGTCGACGTCGAAAGAAAGAATA
CGTAAAATGTCTGGAGAGCCGAGTTGCAGTGCTGGAAGTCCAGAACAAGAAGCTTATAGAGGAACTTGAAACC
TT??
??
??
??
??
??
??
??
??
???TGTTTGTATCTAAATGTGTGGATTCCA
GCACCTAAACCAAAAAATGCCACTGTAATGATATGG-
ATTTATGGTGGTGGTTTTCAAACTGGAACATCATCTTTACATGTTTATGATGGCAAGTTTCTTGCTCGGGTCG
AAAGAGTTATTGTAGTGTCAATGAACTACAGAGTGGGTGCCCTAGGATTCTTAGCTTTGCCAGGAAATCCTGA
GGCTCCAGGAAACATGGGTTTATTTGATCAACGGTTGGCTCTTCAGTGGGTTCAAAAAAATATAGCAGCTTTT
GGTGGAAATCCTAAAAGTGTAACCCTTTTTGGAGAAAGTGCAGGAGCAGCTTCAGTGAGTCTGCATTTGCTTT
CTCCTGGAAGCCATTCGTTGTTCACCAGAGCCATTCTGCAAAGTGGATCCTCTAATGCTCCTTGGGCAGTAAC
ATCTCTTTATGAAGCTAGGAACAGAACGTTGGCCTTAGCTAAATTTACTGGTTGCTCTAGAGAAAATGAGACT
GAAATAGTCAAATGCCTTCAAAACAAAGATCCCCAAGAAATTCTTCTGAATGAAGCATTTGTTGTCCCCTACG
GAACTCTCTTGTCAGTAAACTTTGGTCCAACAGTGGATGGTGATTTTCTCACTGACATGCCAGACATATTACT
TGAACTTGGACAATTTAAAAAAACCCAGATTTTGGTAGGTGTTAATAAAGATGAAGGGACAGCTTTTTTAGTC
TATGGTGCTCCTGGCTTCAGCAAAGATAACAATAGTGTCATAACTAGAAAAGAATTTCAGGAAGGTTTAAAAA
TATTTTTTCCAGGAGTGAGTGAGTTTGGAAAGGAATCCATCCTTTTTCATTACACAGACTGGGTAGATGACCA
GAGACCTGAAAACTACCGTGAGGCCTTGGATGATGTTGTTGGGGATTACAATATCATATGCCCGGCCTTGGAG
TTTACCAAGAAGTTCTCAGAATGGGGAAATAATGCCTTTTTCTACTATTTTGAACACCGTTGTGCTAATTGTG
AGAAAATATGCTACTTTGATCAAACTAGAATGAAGGAAATAAAAAGACACTGTGGATCAACTTGTGATAAAGA
GCAGCTCATCCTCACAGCTTTTTGATTGTTGCTATAGTTATCT---
ATTCAACATGGGATGCACAATACAGTTAACATTTCATTACCAGTAGCCTGTTGCGTGCTTATTGTTAAAAGGA
ATTTAACATACAAGTTTTTGTATGGACTGTTCGCCTAATTCCGATTCCTCGTGCTGACTGGCATGAGACAGGG
AGCTGGATTGTATTAAACTGGTTTTATTGTGATGTGACTATGATGGATGTGACAAGGGCCTCTATTATTCACT
GGGGAGTGTGGCCGTTAATGAAGCCATTAGCTGAGCTAAGGCAAGTGAAGAAAATAGGGAAATTACCATTATT
CTGAAGCCATTAAGTTGTAATCCACCACTTTCTCTCCTTATTCAAAAAGGCATCTTCTTTTTAAGGCCTCCCT
??
?????GGCTTGGCCTACCCAGGTATGCGGACCCATGGGACTCTGGAGAGCGTGAATGGGCCCAAGGCAGGTTC
AAGAGGCCTGACGTCGTCGTTAGCTGACACTTTTGAACATGTGATAGAAGAGCTGTTGGATGAGGACCAGAAA
GTTCGGCCCCATGAAGAAAACAATAAGGACGCAGACTTGTACACGTCCAGGGTGATGCTCAGTAGTCAAGTGC
CTTTGGAGCCTCCTCTTCTCTTTCTGCTGGAGGAATACAAAAATTACCTGGATGCTGCAAACATGTCCATGAG
GGTCCGGCGCCACTCTGACCCTGCCCGCCGAGGGGAGCTAAGCGTGTGTGACAGTATTAGTGAGTGGGTAACG
GCGGCAGACAAAAAGACTGCAGTGGACATGTCGGGCGGACGGTCACAGT?????????????????????
??
???TTCTTTGAGCAGATGCA
GAACTAGACCCCC-
GCCACGGCAGCCTCTGAAGTTGGACAGCAAAACCATTGCTTCACTACCCATCGGTGTCCATTTACAGAATAAT
GTGGAAAGAAACAAACCCCTCTGTCTTATGATTTACTCATTATCGCCTTTTGACAGCTGTGCTGTAACACAAG
TAGATGCCTGAACTTGAATTAATCCACAGATCAGTAATGTATTCTCTCACTCTCTCTCT--TT----------
--------ACATTTTGGTCTCTACACTACATTATTAATGGGTTTTGTGTACTGTAAAGAATTTAGCTGTTTC-
AAACGTAGTGCATGA-
ATAGATTCTCTCCTGATTATTTATCACATAGCCCCTTAGCCAGTTGTATATTATTCTTGTGGTTTGTGACCCA
ACTAAGTCCTACTTTAAATATGCTTTAA-GAATCGATGGGGGA---
TGCTTCACGTGAACGTGGGAGTTTAGCTGCTTCTCTTGCCTAAGTATTCCTTTCCT-

GATCACTATGCATTTTAAAGTTAAAACATTTTTAAGTATTTCAGATACTTYAGAGAGATTTTTTTTTTCCATG
ATTGCATTTTACTGTACAGATTGCTGCTTCTGCTATATTTGTGATATAGGAATTAAGAGGAT???????????
??????????????????????????????????TCCGGAGAGCACTAAGCCACTTCCAGCCCTGCTGTGAACC
AGGACCGTCGGAGCCATCTTTTTCATGTGGTCAGCTTTGGAAGCATCTACATCCACCACTTTAGAAGACAAGT
CTAGTGGCTTATCTGTGACGTCTTTGCTAACGGTCTGCTTCTCCAACAGAGGAGGTGAGCTGCCGTCTTTTCT
GTCTTGAACCGCTGTCTTCCGGGCATGCCCAGGCACTGGCTGGGCACCTTCGCCCCCTTCCGGAGCCTTGGGA
TACTTGCCGTTGGAGAGCCTGGCCGCGGGGAACTCGCTGCTGACTGTCATGTATGGCTTGGACAGGGCAACTG
AAGGAGAGGTGGAGATCCTGGCATAGTGCTTGTGGAACTCCGAGTAGGTGTCTGCAGTAGGCTGGGTGGGAAG
GTGGACCCGGGGTGAGGGCCGAGGCGAGGGGGGCAACAGGAGAGCCGTGTCCCCCGGCAGGCCACTGGTGACT
GCCTTGGCAGAGGGAACCCTAGGCTGTTTACTGTTCTGGATGTGAGGATAGGCATGGGAATCAACGGGATTCC
CAGGGCTGACCCCCATCTTCCACGGGAGGCTTTTGTCTGCGCAATGGACAAGAGGCGGGATGGCTGGGGAGGC
CGAAGGCGTCGAGAGCCTCATGGGTGATGCCAAGGACGACGGGATGTGGGGACTGACGTAGTGAGGTGGTGGC
AGGTAGAGAAAGCGCTCCCCGTTGGTGCAGACTGGAGAATACAGAGGCTGGGCCAAGCTGTAGGACTGCTG??
?????????TGCCAGGACCTTTGTAATTCAAGTGCTTTTTACAGTAGTAGCAAATGTTACATAAGCCCAAACA
TGGAGCA---
TATTTGGAGTTGGTGGGCAGACCCAAATCATGATGCAAGCCTGCAGTAGCAAGTGTGCAATGAAGCACAACCA
AATTCTGTGAATGGAAGGTAGCGAGACGCCTCTCCAAAGCAGCCCTTCTGCGCAGGAGCTGAGAGCTCCACCG
GGTAAGTTTTCTGAGGCAGTTTTCAGAGGCTGGGCTTGGCTCAGGGCCCAGCTCTGGATGAGCCTGTGGACGG
TGGGGT----
AGGTCTTTGCTAACCTTACCTAGAACCACGCAGAGTTTGGAAGAGTTTGTGCAGGCCTCTTTCCTCCTGGCGT
TTGTCTTGCCCTGCCAGCAGGCCAGGAGGG-
CTGCGCYGCTCAAGTAGTGAAGGGAGGGAGCCTGCAGAGCTAGGGGATCAGAGTAGGTAATCGCAGCCACAGG
GATTAGCTGCTGCAAGCTGTCCACAGGAGCCATTCTGAAATGTCATTTGAGTGATCTGGCCGTCTACTGGAGG
AGGCCCTCGGGGCAGCTGTCTCCAGGAGCAGCTTCTGAGGGAGTCTTCCGGGAGGAGCAGTGTCTCTGCCATC
GTCCTCGTCCACGTCCTGGTCACATTTCCATTCCTCATCTTCATTTAAAGTCGGCAGGTATCCAGATAGGCCC
TTCCCTGTCCCTGACCCAGAGCTCTGGTCACTACAGACCTTCGGGCTCTCCGAGCCTGTCCTCGTATATTCCA
AATAAATATCAGACTTRAGGAAGGAGGGGTAGGTGTTTTCCTCCATAGTGGCCTGGATTTCTGTCTGGGCCTG
GTCAAACATGGCAGGATCGATCAGCTGCTTCATGATGCAGCCCTTTATGAAGCTCTTGGTGGCCGGCTTGGTC
TGCCGGGACACGATGCCATTGTTATCAAGGATGTACTTTCGGTAGATGGCTCTGGCCAGCTTCAGCCTCTTCT
CCTCGTTGGAGTCACAGGGCTCCAGCTTCCTGAAGCCAGTGCAGGCAAACCAGAAGTCCAGCAGGTCAGCACA
GCCCTCCTGCTTCAGGAAAGTCCTGAACAGGCTTATCCCATCTTGATCATCCAGCAGGGAGTGCAGTGACTCA
GCCCACTTCAAGTACGGTGGGGTGGGGGAGGCACTGCCCTCAGGCTCATACCCCAGGTCCAGATCCGAGCGCC
TCGGAGTGGCGGTCGAAGTCTCACCTTTAATGCCAATACCTTTCCCGGAGCAGAAACTGTAGCTGGCGGGCCT
CGGGTCTGTGGACACCAGTTCTCCCTCCTCACCAGGCACTGGGGGTCGGGGAGCATCTTCGGTGAAACTTGCT
CCAAGGTCCAAGGGGAAACCCTGCTCCTGGACATTCATTTGGGACTCTGTGCGTCAATGCACAATGAGCGCT
GCACCCTAATACATCAGTACTTA-
CAGCTCCAAAGTCAATCAATCTGTCCTGTTGAAACCAT?????CTGAAGTGTTAGCTAATTTTAAGTGAACAA
-A--ATTACATGGATCAACTTTTGGCAGCATAAAACAGGTATAATTTACAGTGGAGCAGGCTAAGT-
ACTTAGGTTATTTATAGGACAGATTACTTTTCTGTGTTGTTTTG----CTTCTTTG-
CATATTAAAGTTTATTTATAGGTGTAGTTAAGCTTT--ACTATTAGTTACCATAAATTTGGCTTGGAGTCA--
--
CCAAAGCAGAAATATTAGACTTCATTTTTATGAGTTATTTCCTACACATTTCTAAATTGATGTAGCTTTGTCA
CTGCTTGATTGATTTTTTTTT--
AAACTGGAGGGGGGTGAAATTAGTTTATACTGAATCAAACCAGTTTCTCTTAATTAGTA---------------
----CATTATTAACACTGAAAAGAAGTAAAAGGAATGTTAATAACTTAGGAGTATTTGCGTAGTTA-
TGTTTAGAACACAAGCTTTAAATTAGT-GAGGAAACCA---------
GAAGTTTGATTTAAGCACTCATACTCCTTTATTTTCCTTTGTTAGCAATAATTATTGGCCCTGATGGCCATCC
TTTGACTGTTTATCCTTGTATGATTTGTGGGAAGAAGTTTAAGTCAAGAGGTTTTTTGAAAAGACACATGAAA
AACCATCCCGAACATCTTGCCAAGAAG---
TACCACTGTACTGACTGTGATTACACTACCAACAAGAAGATAAGTTTACACAACCACCTGGAAAGCCACAAAC
TGACCAGCAAGGCAGAGAAGGCCATTGTATGTGATGAGCGTGGGAAGCATTTTTCTCATGCAGGGGCTTTGTT
TACTCACAAAATGGTGCACAAGGAAAAGGGAGTCAGCAAAATGCACAAGTGTAAATTCTGTGAATACGAGACA
GCTGAA???CCCGCTATAG
CTTCTGCAAACAGTAAAGATACCACTAAAACCCCTTCTGCAGTTCAAACTAGCACCAGCCACAACCCTGGACT

TCCTCCTAATTTTAACGAATGGTACGTCTGAGGACAAACACAAACCCTGTTAACTATAGAATGGACCAAATGC
ATTTTTAAAAGAAAACTGAGACCAATCAGATGGAAATGGAGTTTTAAGGCAAGAGGCCATATATAGGGCTACG
TCTTGTTAATTGCAGTTGTCCAGGAAGGTTTTGGGCAAGATCCAAAAGTAGCCATGCCCTTTTCTCAGGATTA
GAAAATATGTTTTGGCGTTTGAAGGATTTTTTTACAAAATCTTTACATTGCTTTTTCTTCCCCTTCCTCTTG
CTCTCTACACACCCCATTCCTAAACTCCTGCAATTCATTTGAACACTTGTCCTGTTTCTTGAGAGGAAGTTAT
AGAAGGCTTGTTGG???
???????????????????????????????????
Callicebus
?TACCCCCATGTTTGGCTGGAACATGAAGCTGACCTCAGAGGACTACAGAAATGTCACCTTCCTTTCATGCCG
GTTCCCTT-
CCGTCATGAGCATGGACTACATGGTATACTTCAGCTTCTTCACCTGGATTTGCATTCCCCTGGTCATCATGTG
TGC--AATCTATCTTGACATCTTTTACAT-
CATCCGGAACAAACTCAGTCAGAACTTCTCTAACTCCAAAGAGACGGGTGCATT---
TTATGGACGGGAGTTCAAGACGGCGAAGTCCCTGTTTCTGGTGCTTTTCTTGTTTGCGTTGTCATGGCTGCCT
TTATCCATCATCAACTGCATCACCTACTTTCA--------------
TGGTGAAGTACCACAGCTTGTGCTGTACCTGGGCATCCTGCTGTCCCATGCCAACTCCATGATGAACCCTATC
?????????????????????ATGTTCATAAATCGCTGACTATTTTCAACCAACCATAAAGACATCGGAACGC
TCTACCTTCTATTCGGCGCCTGAGCAGGGGCAGTCGGAACCGCCCTAAGCCTCCTAATTCGAGCGGAACTAGG
GCAACCTGGGAGTCTAATAGAAGATGATCACGTCTACAACGTTATTGTAACCTCTCACGCATTTATTATAATC
TTCTTTATAGT---TATGCCAATCATAATTGGGGGTTTTGG---
AAACTGACTAGTGCCCCTTATAATTGGTGCGCCTGATATAGCATTTCCCCGAATAAACAATATAAGCTTCTGA
CTCCTCCCCCCG--TCACTCCTCCTGCTACTTG-
CATCATCAACTCTAGAGGCCGGCGCAGGAACTGGCTGAACAGTTTACCCGCCCCTGGCCGGAAATATGTCTCA
TCCAGGAGCCTCAGTAGACTTAACTATCTTCTCGCTACACCTGGCAGGTGTCTCCTCCATTCTAGGAGCTATT
AATTTTATTACTACAATTATTAATATAAAACCCCCAGCTATAACCCAATACCAAACTCCTCTTTTTGTATGAT
CAGTTCTCATCACAGCAGTTCTTCTTCTCCTTTCTCTCCCTGTCCTAGCTGCTGGAATTACTATACTACTAAC
AGATC--
GCAATCTTAATACTACCTTCTTCGACCCAGCCGGTGGGGGTGACCCAATTTTATATCAGCACCTATTTTGATT
CTTTGGGCACCCTGAAGTGTATATTCTCATTCTACCCGGCTTCGGAATAATTTCCCACATCGTAACATACTAC
TCCAATAAAAAAGAACCATTTGGGTACATAGGAATAGTATGAGCTATAATATCTATTGGCTTTCTAGGATTTA
TTGTATGGGCACACCATATATT-
TACAGTAGGAATAGATGTAGATACCCGTGCATATTTTACATCAGCCACTATAATCATTGCTATCCCCACCGGA
GTAAAAGTCTTCAGCTGACTGGCCACACTGCACGGCGGTAACATTAAATGGTCCCCTGCAATACTATGAGCCC
TAGGCTTCATTTTCCTTTTTACTGTAGGTGGACTGACCGGGATTGTATTAGCCAACTCATCATTAGACAT-
TGTTTTACATGATACATACTATGTAGTAGCCCACTTTCACTATGTGCTATCGATAGGGGCAG-
TATTTGCTATTATAGGAGGCTTTATTCACTGATTCCCACTATTCTCTGGCTACACCCTCGACCAAACTTACGC
TAAAATTCACTT-
CAGTATTATATTTGTAGGCGTAAATATAACCTTCTTTCCCCAACATTTCCTCGGTCTATCCGGTATACCTCGA
CGCTACTCAGACTACCCTGATGCCTACACAACATGAAATATTGTATCATCTGTAGGTTCATTTATCTCACTAA
CAGCAGTTATCTTAATAATCTTTATAATTTGAGAGGCTTTCTCCTCAAAACGGAAAGTCATAATTGTTGAACA
ACTATCCTCCAACCTAGAATGACTTTACGGCTGCCCCCCTCCCTACCATACATTTGAAGAAGCTACTTATGTA
AAAACCCTCAAC?????????????????????????ATGGCCCACCCAGCCCAACTTGGCCTACAAAATGCAG
CATCCCCTATTATAGAAGAACTAATCGCCTTTCATGACCATGCCCTAATAATTATCTTTCTTATTAGCTCTCT
AGTTCTATATATTATTTCCCTAATGCTTACCACAAAACTTACACATACCAGCACAATAAATGCCCAAGAAATT
GAAATTATCTGAACCATTCTACCTGCCATTATCCTTATTATAATTGCCCTTCCATCCTTACGTATCTTGTACA
TAACAGACGAGTTCAACAAACCATATCTAACCCTTAAAGCTATTGGCCACCAATGATACTGAAGCTACGAATA
CTCGGACTATGAAGACCTGGCCTTTGACTCCTATATTATGCCCACATATTTCCTTGAGCCTGGTGAATTCCGA
CTTCTTGAGGTAGATAACCGCACTACCTTACCAATAGAAGCAGACATCCGCATATTAATTTCATCACAAGACG
TACTACACTCATGAGCCGTACCATCACTAGGCGTTAAAGCAGATGCAATCCCTGGACGCTTAAACCAAGCTAT
AGTAGCCTCAATACGGCCAGGTCTCTTCTATGGACAGTGCTCAGAAATTTGTGGGTCAAATCACAGCTTTATA
CCTATCGTTCTAGAGTTTATTTATTTCCAAGACTTTGAAGTCTGAGCCTCTTA????????????????????
ATGACCATTACCCGCAAAACACACCCACTAGCAAAAATTATTAACAACTCATTCATTGACCTACCCACACCAT
CCAATATCTCATCCTGATGGAACTTCGGCTCACTCCTAGGTATCTGCCTCATTACCCAGATTGCCACCGGCCT
ATTTCTAGCCATACATTACACGCCAGACACCTCCACCGCTTTCTCCTCAGTAGCCCACATTACACGAGATGTA

AACTACGGCTGAATTATCCGTTACCTACACGCTAACGGCGCCTCTATATTTTTCATCTGCCTATTCCTTCACA
TTGGACGAGGCCTGTATTACGGGTCATTTCTCTTTCTGAAGACCTGAAATGTCGGTATTATTCTACTACTGGC
ATCCATAGCCACAGCATTTATAGGGTACGTACTTCCATGGGGCCAAATATCTTTCTGAGGCGCCACAGTAATT
ACAAATCTTCTATCAGCTATTCCCTACATCGGATCAGACCTTGTACAATGGATTTGAGGAGGCTTTTCCGTAG
ACAAAGCTACACTCACACGATTCTTTACCTTCCACTTTATCTTACCCTTTATTATTGCAGCCCTAGCTGCTAT
CCACCTTTTATTTCTGCATGACACAGGCTCCAATAACCCATCAGGATTAGTATCCAACTCCGACAAAATTGTA
TTCCATCCCTATTACACAATTAAAGATATTCTAGGACTAATCTTTCTCCTCCTCCTCCTAATAAGTTTAACTC
TATTTACACCTGACCTTCTAACTGACCCAGATAACTATACCCTAGCTAACCCCCTTAACACTCCACCCCATAT
TAAGCCAGAGTGATACTTCTTATTTGCATACGCCATTCTACGATCCATCCCCAATAAGCTAGGAGGTGTTCTA
GCCCTTATTTTATCCATCTTAATCCTAGCAGTTATTCCAATAACCCATCTATCTAAACAACAAAGCATAATAT
TCCGACCAATTGGTCAAAGCCTATTCTGAACTCTAGTGGCCACTCTACTTACACTGACATGAATTGGAGGTCA
ACCAGTAGAATACCCCTTCGTAGCTATTGGCCAAATAGCATCCATTATATACTTTCTTACTATTATTACCCTC
ATTCCTCTATCCTCCCTAATTGAAAATAAACTACTCAAATGGTAA?TTAGGTTAAATTTATTCAAAGGAATGA
GAATTTATGGGTATAACTGTTAGCTGGAAAGCCCTTCCTCACCTCCAATCCTCCTTTCAAACTACATTTCC-
TCCTGAAATTTCTCTGTGGGATGAAATGTTCAATTATTAGTTAATCAATC----
TCTCATATATTATCTGGCTTTACATAT----
GCCCTTGATCGTTCTTGGAAAATAATCCARTATTGTGTTGTTTTGGTGTCTTTGTATTGGAAATGGAAGCATT
TTGTATGAATCACCTCTTTAAGTGTGAGGAGTTGGATTGTCTTAGTTCGGCTTGCCTAATGACCAGGAATAAA
--GATATCACAGTCTCTAATCCCACTTTTT-
GGTGGAAAGAAAATCTAAAAGTAACAACATTTAAGGGAACAAAGTATCAATAATCAAAGAAACTCCCCTGTGA
AACTGTAATATTATTTT-
AAAATCGACATTGTTCTAAGGTAGCTGAAATGTGTGATTTTTCTATTTTGCCTTTTCACTGACAATATACCCA
TAAATCT-
TGGTGGTGATTTTCTCTCCAAAGTTAATTTCTGGACCATTGGTGACTGCACTGTCTATTGTTCTAAATTGTTG
TCTTAATTGCATTCTTTAAAAATGTAATGTTGAAGGCACATAATATTGAAGATGACTCCAGGATGAACCCTGA
GTTCGCAGACCGAA-
TAAAACAGCTCGATAAAGAGGCGTCTTACTACCGTGATGAGTGTGGCAAGGCCCAAGCAGAAGTGGACCGGTT
GCTGGAGA?????????GGAAGAGCTGCATCAGGCAAGTACTTTGATCTTTCTTGGAGCAGGTTCCTTCAAAA
CGCTTCTCCAGAGGACGGAAGTCCCTCCAGTCTGGCTCGCTGCTCGCAGTGGTGGGGAAAGCTGAGGTAATTC
CCCGGTGGGAAATCTGCGAGGCCCCTGATACCCCTG--------
CCACCAGGCCCATCACTGATGGGCCTAAGGGCCTCATCTGATACTCTAAGACGGGCTTCTCCTGTCGGATCTG
GGCCTCTCGGACTTGAAACTCTCTAGAACGAGCCTCTCGGGCCTGGGCTTCTCGGGTTCTGGCCTCCCTGGCA
TGACCTTCCCAAGTGTGGGCCTCCCTG--
GCATAGGCTTCCTTGCCATGAGCCTCTCGAGTATAGGCCTCCCTGGTGTGGGCCTCCCTGGCCTGAGCTTCTC
GGGCACGTGCCTCCCGGGCCTCAAGCTGCTCCCGCCGAAGCTCCCAATACAACAACTGTTTCTGGATGGTCA-
CTAGCCGTTCCTCCTCTGTCTCCATTGCCCCAGGTGGCCGGGAGGAAGAAAAGGGCTCAAAGTTCAAGAAGGG
GTCAAACATCTCAGAGCTTCGACCGTGGAGGTCATAAAGGCAGTCATCCTCAGGTGGAGAGTTCTCAAGACTG
TCATCTGGCTCATAGAACTCATATAGGGCATCTCCACTGTAGCTGTCTCGGGGCAGACAATCCCTGCGGACAA
GCCCCAGGGCCTCACCTGAATCATCCTCAA?????????????CTAATTTTGATGAACTAAAAATGGTTCCTG
AAGGTCAGATTCATGTACAGTGAATGTTCTCCACTGTTGTGGTGGGCATGTTTCATGCGTGGGGATGTTGGTG
TGCAAGTTTCGTGTGTTTGGAAAACACTAGCTAGAACATTCGAAAAATTTCATTAACCCAGCTCTGGAAGGTC
ATCTATGAATTCTTCCTATTCCAGTGGCCTTTT-
AAATGTAGATGTAGATACACTGTAATTTAAAACAGATGGGCATGTCTCAGTATTTTCATATGCAAAATGGGAC
AGATGTATATGAATGGATAAATTATCCTTTTAAAAAGATACTTTGTAGCACGAAATATCACCAATGATTGGAA
CAGTGAACCTGCATTCAGTCTAGGGCAAACACCAGGGAGACATTATCAAAACATCTAAACTATCCACAAACTA
ATTCCAATTAGTCTACTTTGTCTTGATACAGAAAAAA-CCTTTTTGATTTTTAAA---
TATGTATTGGGTGCCAATCCATATCCTTT-
GAGCTGCTAAAGCCAGCATATCACAATAGAAGTTTACTTTTCCTTGAGAGT????????????ATAATCAATT
TACTACTACTTATTGTCCCAGCCCTCATCGCCATAGCATTTCTTACACTCACAGAACGAAAAATCCTGGGCTA
CATACAATTCCGTAAAGGCCCTAACATTGTTGGTCCCTATGGAGTTCTTCAACCTATTGCAGACGCGATAAAA
CTCTTTACAAAAGAACCTCTACTTCCCACAGCATCTACCACAATTCTATACGTAATTGCCCCAACCTTAGCAC
TCTCCATTGCCCTCCTCCTATGAGCCCCCTTACCTATACCCCTCCCCCTAATCAATTTTAATTTAGGCCTCTT
ATTCATACTGGCAATCTCAAGTCTAGCTGTATACTCAATCTTATGGTCCGGATGAGCATCTAACTCAAAATAT
GCACTAATAGGCGCATTACGAGCCGTAGCCCAAACAATCTCATATGAAGTTACACTAGCTATTATTCTCCTCT

CCGTCCTACTAATAAGCGGCTCATTCAATCTACACTCACTCATTACAACGCAAGAACACTTATGACTCCTTCT
TCCATCATGACCTCTCGCCATAATATGATTCATTTCAACATTAGCCGAAACCAACCGAGCCCCCTTTGACCTA
ACAGAAGGCGAATCAGAACTAGTCTCAGGGTTCAACATCGAATACGCCGCAGGCTCATTTGCCCTATTTTTTA
TAGCAGAATACATAAATATTATTATAATAAATGCCCTAACCACCACCATCTTTTTAGCAACACCCCACACACT
ACTTACCCCAGAATTATATACAGCAAACTTTATAACTAAAACACTACTACTAACCGCACTGTTCTTATGAATT
CGAACAGCATACCCCCGATTCCGTTATGACCAACTCATATATCTCCTATGAAAAAAATTCTTACCCCTCACAT
TGGCACTATGTATATGATATATCTCAATACCAATTTTTACATCCGCCGTACCACCCCAAACAT??ATTAATCC
CCCAGCCCAACTCGTTATTCTATTTACAATTTTAACAGGAACTATAATTACAATCCTAAGCTCACACTGATTT
CTTATCTGAGTGGGACTAGAACTAAACATGCTAGCTATCGTACCAGTACTAGCTAAAAGTACAAACCCCCGCT
CCACAGAAGCAGCTACAAAATATTTCCTAGTTCAAGCAACCGCATCCATACTTCTCCTAATAACCATCTTTAT
AAACTATATATTCTCCGGGCAATGATCAATCAACCCACCCACAAGCCAATTCTTATCTACTACAATATTAATT
GCCTTAACAATAAAACTAGGCATAGCCCCTCTTCACTTCTGACTTCCAGAAGTTACCCAAGGCATTCCCCTCA
TCCCCGCCATAATCATTCTCACATGACAAAAACTAGCCCCCATTTCAATTATGTTTCAAATCTTCCCCTCCAT
TAACATAAATATCCTCATAGCAATTTCCATAATATCAATTATAATTGGCAGCTGGGGTGGACTAAACCAAACA
CAGTTACGCAAAATCCTAGCCTACTCATCAATTACTCATATAGGATGAATAATAGCAGTTCTACATTATAATC
CAAACATCACCATTCTCAGCCTATTTATCTATCTACTCCTAACAATCTCCATCTTTATAACTTTTTACCTAAA
TTCAAACACAACAACTCTATCCATATCCCACACCTGAAATAAATTTACATGAACAATACCTGTTATACCACTA
ATAATATTATCTCTTGGAGGCCTACCCCCACTCACAGGCTTCTCCCCTAAATGAGTTATTCTACAGGAATTCA
CAAACAATAACAGCCTTGCCATCCCTCTCACCATAGCCATACTAACACTAGTAAACTTATATTTTTACTTACG
CCTAACATACTCTATTTCAGTGACTATATTTCCCACATCCAACAACGCAAAAATTAACTGACAACTAAAATAT
ATAAAACCAGCCCCCCTCCTTACCCCACTTATAGTCTCCTCCACCCTCCTTCTACCTTTAACTCCACTAATAC
TATTAACCT?????GTTGGCAGCATAAATAATAAAGCATGTTACTTAAGCAATTTTACATTTACTGTATTAGT
AAGTAAATGCAATGGCCACTCTTATTCTCAAACAATAAAAACTACCCAGCCTTTTCATGAAAGAT-
GATTGCAATTTCCATTGGCCTGTATATGCAATAAAAAG---
CCATAACTCATCAAAATTTTAGCTATTTTCTCTTAAGATA--
GAAGAGCATTTTGCTTGAACATTGAACGTGACATTAAATATT-
CATGAAACTGAATAGAGTTTAAGTACTAAG---
TAAAACTCTCAAATTCTTTTCAACTGGATTGAAATTCTGTTTGAACCAGGTAAAAACTGCCGCTAATCTGAAG
AGGTGTTCAAAAAC-
AGCGTAATGAGTTTGTATAATATGTTCAAAGTGCTACAGTTATTTGGGATAGATTTTTCTGAAAAGCCTGTTC
CTCCTTTGAAATTGATTCATGATAACAGCCTCCATTTGGGTCAAACAGTATTTAACCGCTTGTAAA-
TTTTAATT-
AAATGATTTGTCAGTTTGAACACATAGTTTGT?CCCAAGACCTTGAGGATTACTTAAATGGCCCCTTCACTGT
GATTGTGAAGGAGTCTTGTGATGGAATGGGAGATGTGAGTGAGAAGCATGGGAGTGGGCCAGTAGTCCCAGAA
AAGGCAGTCCGTTTTTCATTCACAATCATGAAAATTACTATAACCCACAACTCTCAGAACATGAAAGTGTTTG
AAGAAGCCAAACCTAACTCTGAACTGTGTTGCAAGCCATTGTGCCTTATGCTGGCAGATGAGTCTGACCACGA
GACCCTGACTGCCATCCTGAGTCCTCTCATTGCTGAGAGGGAGGCCATGAAGAGCAGTGAATTAATGCTTGAG
ATGGGAGGCATTCTACGGACTTTCAAGTTCATCTTCAGGGGCACTGGATATGATGAAAAACTTGTGCGGGAAG
TGGAAGGCCTTGAGGCTTCTGGCTCAGTCTACATCTGTACTCTTTGTGATGCTACCCGTCTGGAAGCCTCTCA
AAATCTTGTCTTCCACTCTATAACCAGAAGCCATGCAGAGAACCTGGAACGTTATGAAGTCTGGCGTTCCAAC
CCTTACCATGAGTCTGTGGAAGAACTGCGGGATCGGGTGAAAGGGGTCTCAGCCAAACCTTTCATTGAGACAG
TCCCTTCCATAGATGCCCTCCACTGTGACATTGGCAATGCGGCTGAGTTCTACAAGATCTTCCAGCTAGAGAT
AGGGGAGGTGTATAAGAATCCCAACGCTTCCAAAGAGGAAAGGAAAAGGTGGCAGACCACACTGGACAAGCAT
CTCCGGAAAAAGATGAACCTCAAACCAATCATGAGGATGAATGGCAACTTTGCCAGGAAGCTCATGACCAAAG
AGACTGTGGATGCGGTTTGTGAGTTAATTCCTTCTGAGGAGAGGCACGAGGCTCTGAGGGAGCTGATGGATCT
TTACCTGAAGATGAAACCAGTATGGCGGTCATCATGCCCGGCTAAAGA--ATGCCC-
AGAATCCCTCTGCCAGTACAGTTTCAATTCACAGCGTTTTGCTGAGCTCCTTTCTACCAAGTTCAAGTATAGA
TATGAGGGGAAAATCACCAATTATTTTCACAAAA???????????????CACCCGACAGTTGTCATTGTGTT
TGGAAACACTGCCGGCCCTTGGAGATGAGGATAAAGTGGACATTTCTCTGCTGCATCAGGGTGAGAATCTTTT
CGAACTGCACATCCACCAGGCCTTCCTGACATCTGCCGCCCTGGCTCAGGCTGGAGACACCCAACCTACCACT
TTCTGCACCTATTCCTTCTATGATTTTGAAACCCATTGTACTCCACTGTCTGTAGGGCCACAGCCCCTCTACG
ACTTCACCTCCCAGTATGTGGTGGAGACAGACTCCCTTTTCTTACACTACCTTCAAGGGGCTTCAGCCCGGCT
TGATCTCCACCAGGCTGTGGCCAGTGAGCACAACATTCTTGCTGCAGGATGGATTTGCTTTGACAGGGTGCTA
GAGACTGTGGAGAAAGTCCATGGCTTGGCCACACTGATTGGTAAGTGCTGTCGGCTTCCTGCAGCTCCTA-

GCACCACTGCAGAATTTCCCAAACTCTGTAGCTGCTTTTCTGGTTGTTTCTTACTTTGTTCCTTTTTCTTTGT
TCTTGATCCTTGCCACACCATCTGTATTCTCCATGCTTTCA-
TACTTGCTGCTGCCCAGGAGCTGGTGGAGAAGAGTTTGGGGTTCTAGAGTACTGGATGAGGCTGCGTTTCCCC
ATAAATCCCAGCCTACAGGCATGCAATAAACGAAAGAA???????????????????????????????????
???????????????????????????TTCATGGAGGGAAAACACCAAACAATGAGCTTTCAGATAAGATTTA
TGTCATGTCTGTTGTTTCCAAGAACAAYAAAAAGGTTACTTTTCGCTGCACGGAGAAAGACTTGGTAGGAGAT
GTTCCTGAAGCCAGATATGGTCATTCCATTAATGTGGTATATAGCCGAGGGAAAAGTATGGGTGTTCTTTTTG
GAGGACGCTCATACATGCCTTCTACCCACAGAACCACAGAAAAAATGGAATAGTGTAGCTGACTGCCTGCCCCA
TGTTTTCCTGGTGGATTTTGAATTCGGGTGTGCTACATCATACRTTCTTCCAGAACTTCAGGATGGGCTATCT
TTCCATGTCTCTATTGCCAAAAATGACACCATCTATATTTTAGGAGGRCATTCACTTGCCAATAATATCCGCC
CTGCCAACCTGTACAGAATAAGGGTTGATCTTCCCCTGGGTAGCCCAKCTGT-
GAATTGCACAGTCTTGCCAGGAGGAATCTCTGTCTCCAGTGCA??????????????????????????????
???
??TGTGTCTTAGTATCTTCCTGCTC
TCCACCACACTGAGCCCACGATGACCCAGTGCTCCACAAACATAYGTAGAATGGAAGTAAAACCTCAGGGATA
AGAAAAGATAATTAAAGGGCCGGGTACAGTGGTTCATGCCTGTAATCCCAGCACT-
TAGGAGGCCAAGGCGAGTGGATCACAAGGTCAGGAGTTCAAGACCAGCCTGGCCAAGATGGTGAAAACCCATC
TCTACTAAAAATA---CAA---TT-----
AGCTGGG--
---------------------CGTGGTGGTGGGCACCTGTAATCCCAGTTACTTGGGAGGCTGAAGCAG-
AGAACTGCTTGAACCTGGGAGGCAGAGGTTGCCGTGAGCCGAGATCGCGCCACTGCACTCCAGCCTGGACAAC
A-GAGTGAGACTCCATCTCAA--

---AAAA-TAAGA---
AGAAGAAAAKATAGTTAAAGAAGATTCACTGCAGTAACAAACTAGCCAAGAAATTGCACTGAAAGTAAGGGTT
T-GGGTGACAAAATTTCCAACTGTGTTCAAAGAAACATAAGTTCCAGCAGGAGTRCACTTAATCTGATTTT-
CTTCAAAGC--
CTCTCTAATTTGTAGAGAAGGACATTCATTTTCATTAGCTTCATGTGAGCTAGAGAGAATCCATGATTGAAGC
ACTAGAACCCCTGGTAAACATGCCAGCAAGCAAGGGCACCTTCCCCAGAGGCTCAAACTAGAATGTAAAACTG
TTGGCACTGGTAAGGAGCCTTGAACTCTAAACCCATAGGCTCAAGTCAGGAA-
CTCAGGAAGCCTCTCAACACTGGGATACAATTTTGCCWCCTTAACACTTTATTCCCTTTACTTGTGCCAAATT
???
???
???
???
???
???????????????????????????GAACTCAAGGCCCTCAAAAACCTTTATTGCCATAAAGCAGAGTGAC
T-GTCTTTGACTTGGACCTTGTTTACTGTGAACTCTAATC-AAAGCAGGACATGCAGCAGTTCTACTAA-
CCGCACTGTGGACGTGCGGAAAGGACGCTTGTGA---------
ATCCAGTTTGGATGAGTGTTTGAAATTGAATTGTGAATGTTGTTCCAGGATGTGGAATGCAGCCGTGATCACA
CTTGCCTACC-GAGCTTACTTTGAACTGTTTGTCAATAGCATGCAAAAAA-
TGCTTTGTCTGCCCTTTGCTTCTGCTTTTTCCAGGGAAGCYGCCAAAGAATGTCGACGTCGAAAGAAAGAATA
CGTAAAATGTCTGGAGAGCCGAGTGGCAGTGCTGGAAGTCCAGAACAAGAAGCTTATAGAGGAACTTGAAACC
TTGAAAGACATTT??
???
???
???
???
???
???
???
???
??TGTTTATATCTAAATGTGTGGATTCCA
GCACCTAAACCAAAAAATGCCACTGTAATGATATGG-

ATTTATGGTGGTGGTTTTCAAACTGGAACATCATCTTTACATGTTTATGATGGCAAGTTTCTTGCTCGGGTCG
AAAGAGTTATTGTAGTGTCAATGAACTACAGGGTGGGTGCCCTAGGATTCTTAGCTTTGCCTGGAAATCCTGA
GGCTCCAGGAAACATGGGTTTATTTGATCAACGGTTGGCTCTTCAGTGGGTTCAAAAAAATATAGCAGCCTTT
GGTGGAAATCCTMAAAGTGTAACTCTTTTTGGAGAAAGTGCAGGAGCAGCTTCAGTTAGTCTGCATTTGCTTT
CTCCTGGAAGCCATTCCTTGTTCACCAGAGCCATTCTGCAAAGTGGATCCTCTAATGCTCCTTGGGCAGTAAY
GTCTCTTTACGAAGCTAGGAACAGAACGTTGGCCTTAGCTAAATTTACTGGTTGCTCTAGAGAAAATGAGACT
GAATTAGTCAAATGCCTTCAAAACAAAGATCCCCAAGAAATTCTTCTGAATGAAGCATTTGTCGTCCCCTATG
GAACTCTCTTGTCAGTAAACTTTGGTCCAAYGGTGGATGGTGATTTTCTCACTGACATGCCAGACATATTACT
TGAACTTGGACAATTWAAAAAAACCCAGATTTTGGTAGGTGTTAATAAAGATGAAGGGACAGCTTTTTTAGTC
TATGGTGCTCCTGGCTTCAGCAAAGATAACAATAGTTTCATAACTAGAAAAGAATTTCAGGAAGGCTTAAAAA
TRTTTTTTCCAGGAGTGAGTGAGTTTGGAAAGGAATCCATTCTTTTTCATTACACAGACTGGGTAGATGATCA
GAGACCTGAAAACTACCGTGAGGCCTTGGATGATGTTGTTGGGGATTACAATATCATATGCCCGGCCTTGGAG
TTTACCAAGAAGTTCTCAGAATGGGGAAATAATGCCTTTTTCTACTATTTTGAACACCGTTGTGCTAATTGTG
AGAAAATATGCTACTTTGATCAAACTAGAATGAAGGAAATAAAAAGACACTGTGGCTCAACTTGTAATAAAGA
GCAGCTCATCCTCACAGCTTTTTGATTGTTGCTATAGTTATCT---
TTTCAACATGGGATGCACAATACAGTTAACATTTCATTACCAGTAGCCTGTTGCGTGCTTATTGTTAAAAGGA
ATTTAACATACAAGTTTTTGTATGGCTTGTTCGCCTAATTCCGATTTCTTGTGCTGACTGGCATGAGACAGGG
AGCTGGATTGTATTAAACTGGTTTTATTGTGATGTGACTATGATGGATGTGACAAGGGCCTCTATTATTCGCT
GGGGAGTGTGGCCGTTAATGAAGCCATTAGCTGAGCTAAGGCAAGTGAAGAAAATAGGGAAATTACCATTATT
CTGAAGCCATTAAGTTGTAATCCACCACTTTCTCTCCTTATTCAAAAAGGCATCTTCTTTTTAAGGCCTCCCT
??C
AAGGTGGCTTGGCCTACCCAGGTGTGCGGACCCATGGGACTCTGGAGAGCATGAATGGGCCCAAGGCAGGTTC
AAGAGGCCTGACGTCGTCGTTGGCTGACACTTTCGAACATGTGATAGAAGAGCTGTTGGATGAGGACCAGAAA
GTTCGGCCCCATGAAGAAAACAATAAGGACGCAGACTTGTACACGTCCAGGGTCATGCTCAGTAGTCAAGTGC
CTTTGGAGCCTCCTCTTCTCTTTCTGCTGGAGGAATACAAAAATTACCTGGATGCTGCAAACATGTCCATGAG
GGTCCGGCGCCACTCTGACCCTGCCCGTCGAGGGGAGCTGAGCGTGTGTGACAGTATTAGTGAGTGGGTAACG
GCGGCAGACAAAAAGACTGCAGTGGACATGTCAGGCGGGACGGTCACAGTCCTTGAAAAGGTCCCTGTATCGA
AAGGCCAACTGAAGCAATAT???
???AGCAGATGCA
GAACTAGACCCCC-
GCCACAGCAGCCTCTGAAGTTGGACAGCAAAACCATTGCTTCACTACCCATCGGTGTCCATTTATAGAATAAT
GTGGAAAGAAACAAACCCCTCTGTCTTATGATTTACTCATTATCGCCTTTTGACAGCTGTGCTGTAACACAAG
TAGATGCCTGAACTTGAATTAATCCACAGATCAGTAATGTATTCTCTCTCTCTCTCTCTCTCTCTCTCTCTCT
CTCTCTTTACATTTTGGTCTCTACACTACATTATTAATGGGTTTTGTGTACTGTAAAGAATTTAGCTGTTTC-
AAACCTAGTGCATGA-
ATAGATTCTCTCCTGATTATTTATCACATAGCCCCTTAGCCAGTTGTATATTATTCTTGTGGTTTGTGACTCA
ACTAAGTCCTACTTTAAATATGCTTTAA-GAATCGATGGGGGA---
TGCTTCACGTGAACGTGGGASTTTAGCTGCTTCTCTTGCCTAAGTATTCCTTTCCT-
GATCACTATGCATTTTAAAGTTAAA-CATTTTTAAGTATTTCAGATGCTTTAGAGAGATTTTTTTCC-C-
ATGATTGCATTTTACTGTACAGATTGCTGCTTCTGCTATATTTGTGATATAGGAATTAAGAGG??????????
??????????????????????????TAATCTCACTTCCGGAGAGCACTAAGCCACTTCCAGCCCTGCTGTGA
ACCAGCACAGTAGGAGCCATCTTTTTCATGTGGTCAGCTTTGGAAGCATCTACATCCACCACTTTAGAAGACA
AGTCTAGTGGCTTATCTGTGACGTCTTTGGTAACGGTCTGCTTCTCCAACAGAGGAGGTGAACTGCTGTCTTT
TCTGTCTTGAACTGCTGTCTTCCGGGCATGCCCGGGCACTGGCTGGACACCTTCGCCCCCTTCCGGAGCCTTG
GGATACTTGCCGTTGGAGAGCCTGGCCGCGGGGAACTCGCTGCCGACTGTCATGTATGGCTTCGACAGGGCAA
CTGAAGGAGAGGTGGAGATCCTGGCATAGTGCTTGTGGAACTCTGAGTAGGTGTCTGCAGCAGGCTGGGTGGG
AAGGTGGACCCGGGGTGAGGGCCGAGGCGAGGGGGGCAACAGGAGAGCCGTATCCCCCGGCAGGCCACTGGTG
ACTGCCTTGGCAGAGGGAACCCTGGGCTGTTTACTGTTCTGGATGTGAGGATAGGCATGGGAATCAACGGGAT
TCCCAGGGCTGACCCCCATCTTCCACGGGAGGCTTTTGTCTGCGCAATGGACAAGAGGCGGGATGGCTGGGGA
GGCCGAAGGTGTCGAGAGCCTCATGGGTGATGCCAAGGACGATGGGATGTGGGGACTGACGTAGTGAGGTGGT
GGCAGGTAGAGAAAGCGCTCCCCGTTGGTGCAGACCGGAGAATACAGCGGCTGGGCCAAGCTGTAGGACTGCT
GAGGTAGCAAG?TGCCAGGACCTTTGTAATTCAAGTGCTTTTTACAGTAGTAGCAAATGTTACATAAGCCCAA
ACATGGAGCA---
TATTTGGAGTTGGTGGGCAGACCCAAATCATGATGCAAGCCTGCAGTAGCAAGTGTGCAATGAAGCACAACCA

AATTCTGTGAATGGAAGGTAGCGAGACGCCTCTCCAAAGCAGCCCTTCTGCGCAGGAGCTGAAAGCTCCACCG
GGTAAGTTTTCTGAGGCAGTTTTCAGAGGCTGGGCTTGGCTCAGGGCCCAGCTCTGGATGAGCCTGTGGACGG
TGGGGT----
AGGTCTTTGCTAACATTACCTAGAACCAAGCAGAGTTTGGAAGAGTTTGTGCAGGCCTCTTTCCTCCTGGCGT
TTGTCTTGCCCTGCCAGCAGGCCAGGAGGG-
CTGCGCTGCTCAAGTAGTGGAGGGAGGGAGCCTGCAGAGCTAGGGGATCAGAGTAGGTAATCGCAGCCACAGG
GATTAGCTGCTGCAAGCTGTCCACAGGAGCCATTCTGAAATGTCATTTGAGTGATCTGGCCGTCTACTGGAGG
AGGCCCTCGGGGCAGCTGTCTCCAGGAGCAGCTTCTGAGGGAGTCTTCCAGGAGGAGCAGTGTCTCTGCCATC
GTCCTCGTCCACATCCTGGTCACACTTCCATTCCTCATCTTCGTTTAAAGTCGGCAGGTATCCAGATATGCCC
TTCCCTGTCCCTGACCCAGAGCTCTGGTCACTACAGACCTTTGGGCTCTCCGAGCCTGTCCTCGTATATTCCA
AATAAATATCAGACTTAAGGAAGGAGGGGTAGGTGTTCTCCTCCATAGTGGCCTGGATTTCCGTCTGGGCCTG
GTCGAACATGGCAGGATCGATCAGCTGCTTCATGATGCAGCCCTTTATGAAGCTCTTGGTGGCCAGCTTGGTC
TGCCGGGACACAATGCCGTTGTTATCAAGGATGTACTTTCGGTAGATGGCTCTGGCCAGCTTCAGCCTCTTCT
CCTCATTTGAGTCACAGGGCTCCAGCTTCCTGAAGCCAGTGCAGGCAAACCAGAAGTCCAGCAAGTCAGCACA
GCCCTCCTGCTTCAGGAAAGTCCTGAACAGGCTTATCCCATCTTGATCATCCAGCAGGGAGTGCAGGGACTCA
GCCCACTTCAAGTGCGGTGGCGTGGGGGAGGCACTGCCCTCAGGCTCATATCCCAGGTCCAGATCCGAGCGCC
TTGGAGTGGCGGTCGAAGTCTCACCTTTAAGGCCAATACCTTTCCCGGAGCAGAAACTGTAGCTGGCAGGCCT
CGGGTCTGTGGACACCAGTTCTCCCTCCTCACCAGGCACTGGGGGTCGGGGAGCATCTTCGGTGAAACTTGCT
CCGAGGTCCAAGGGGAAACCCTGCTCCTGGACATTCATTTTGGGACTCTGTGCGTCAATGCACAATGAGCGCT
GCACCCTAATACATCAGTAATTA-
CAGCTCCAAAGTCAATCAATCTGTCCTGTTGAAACCAT?????CTAAAATGTTAGCTAATTTTAAGTGAACAA
-A--ATTACATGGATCGACTTATAGCAGCGAAAAGCAGGTATAATTTACAGTGGAGCAGGATAAAT-
ACTTAGGTTATTTATAGGACAGATTACTTTTCTGTGTTGTTTTG----CTTCTTTG-
TGTATTAAAATTTATTTATAGGTGTAGTTAAGCTTT--ACTATTAGTTAGCATAGATTTGGCTTGGAGTCA--
--
CCAAAGCATAAATATTGCACTTCATTTTTATAAGTTATTTGCTACACATTTCTAAACTGATGTAGCTTTGTCA
GTGCTTGATTGATTTTTTTTTT-
AAACTGGAGGGGGTGAAGTTGGTTTATACTGAATCAAACCAGTTTCTCTTAATTAGTA---------------
----CGTTACTAACATTGAAAAGAAGTAAAAGGAATGTTAATAACTTGGGAGTATTTGCGTAGTTA-
TGTTCAGAACACAAGCTTTAAATTAGT-GAGGAAACCA---------
GAAGTTTGGTTTAAGCACTCATACTCCTTTATTTTCCTTTGTTAGCAATAATTATTGGCCCTGATGGCCATCC
TTTGACCGTTTATCCTTGTATGATTTGTGGGAAGAAGTTTAAGTCAAGAGGTTTTTTGAAAAGACACATGAAA
AACCATCCTGAACATCTTGCCAAGAAGAAGTACCACTGTACTGACTGTGATTACACTACCAACAAGAAGATAA
GTTTACACAACCACCTGGAAAGCCACAAACTGACCAGTAAGGCAGAGAAGCCCACTGAATGTGATGAGTGTGG
GAAGCATTTTTCTCATGCAGGGGCTTTGTTTACTCACAAAATGGTGCACAAGGAAAAAGGAGCCAACAAAATG
CACAAGTGTAAATTCTGTGAATACGAGACAGCTGAA?????????????????????????????????????
???AAGATACCACTAAAACCCCTTCTGCAGT
TCAAACTAGCACCAGCCACAACCCTGGACTTCCTCCTAATTTTAACGAATGGTACGTCTGAGGACAAACACAA
ACCCTGTTAACTATAGAATGGACCAAATGCATTTTTTAAAGAAAACTGAGACCAATCAGATGGAAATGGAGTT
TTAAGGCAAGAGGCCATATATAGGGCTACATCTTGTTAATTGCAATTGTCCAGGAAGGTTTTGGGCAAGATCC
AGAAGTAGCCATGCCCTTTACTCAGGATTAGAAAATATGTTTTGGTGTTCGAAGGATTTTTT--
ACAAAATCTTTACACTGCTTTTTCTTCCCCTTCCTCTTGCTCTCTGCACACCCCATTTCTAAACTCCTGCAAT
TCATTTTAACACTTGTCCTGTTTCTTGAGAGGAAGTTATAGAAGGCTTGGTGGTGGTGGTGATGTTAAACTGA
TGGAAATTCTTTTTCGCCTTAGTGGTGATTGTTTAAACTCTCACAGTCTTA??????????????????????
?
Callimico
?TACCCCCATGTTTGGCTGGAACATGAAGCTGACCTCAGAGAACTACAGAAATGTCACCTTCCTTTCATGCCG
GTTCCCTT-
CCGTCATGAGCATGGACTACATGGTATACTTCAGCTTCTTCACCTGGATTTTCATTCCCCTGGTCATCATGTG
TGC--AATCTATCTTGACATCTTTTACAT-
CATCCGGAGCAAACTCAGACAGAACTCCTCTAACTCCAAAGAGACAGGCGCATT---
TTACGGACGGGAGTTCAAGACGGCGAAGTCCCTGTTTCTGGTGCTTTTCTTGTTTGCTCTGTCATGGCTGCCT
TTGTCCATCATCAACTGCATCACCCACTTTCA-------------
TGGTGATGTACCACAGCTTGTGCTGTACTTGGGCATCCTGCTGTCCCATGCCAACTCCATGATGAACCCTATC

?????????????????????ATGTTCATAAATCGCTGATTATTTTCAACCAATCATAAAGACATCGGAACAC
TATATTTACTATTTGGCGCATGAGCTGGAGCAGTAGGAACAGCCCTAAGCCTCTTAATCCGAACAGAACTTGG
CCAACCAGGAAGCCTAATGGAAGATGACCATGTCTATAATGTTATTGTAACATCCCATGCTTTTGTTATAATC
TTTTTTATAGT---AATGCCAATTATAATTGGAGGCTTTGG---
AAACTGACTTGTTCCTCTGATAATCGGTGCTCCAGATATAGCATTCCCCCGAATAAACAATATAAGCTTCTGA
CTCCTACCACCC--TCACTCCTTCTTCTACTCG-
CATCTTCAACTCTAGAAGCCGGCGCTGGGACTGGCTGGACAGTATACCCGCCCTTAGCAGGAAACATATCGCA
CCCCGGAGCCTCTGTAGATTTAGCCATTTTTTCACTCCATCTAGCAGGTGTCTCCTCCATCCTAGGAGCTATC
AACTTCATTACAACAATTGTTAATATAAAACCCCCAGCTATAACCCAATATCAAACCCCTCTATTCGTGTGAT
CTGTTTTAATTACAGCAGTTCTACTCCTACTATCTCTCCCAGTTCTAGCCGCCGGAATTACTATACTGTTAAC
TGATC--
GTAACCTTAACACCACCTTCTTTGATCCTGCTGGTGGCGGCGATCCTATTCTATATCAACATCTATTCTGATT
CTTCGGTCACCCTGAAGTATATATTCTCATCTTACCAGGCTTTGGAATAATCTCACATATTGTAACATACTAC
TCCAATAAAAAAGAACCATTTGGTTACATGGGCATAGTTTGGGCTATAATATCTATTGGTTTCCTAGGATTTA
TTGTATGAGCTCACCATATGTT-
TACAGTAGGCATAGATGTTGATACTCGTGCATATTTTACATCAGCCACTATAATTATTGCTATCCCTACTGGA
GTAAAAGTGTTTAGTTGACTAGCTACACTACATGGCGGTAATATCAAGTGATCTCCTGCAATACTATGGGCCT
TAGGGTTTATTTTTCTTTTCACCGTAGGCGGGTTAACAGGAATTGTACTAGCCAACTCATCACTAGATAT-
TGTATTACATGATACATACTATGTAGTAGCACACTTTCACTATGTCTTATCAATAGGAGCAG-
TATTTGCTATTATAGGAGGCTTTATTCACTGATTCCCTCTATTTTCAGGGTATACACTTGATCAAACTTATGC
CAAAATCCACTT-
TACTATTATATTCGTAGGTGTAAACCTAACTTTCTTTCCACAGCACTTTCTTGGCTTATCTGGTATACCTCGA
CGATACTCAGACTATCCTGATGCATATACTACATGAAATATTGTCTCATCTGTAGGCTCATTTATCTCATTAA
CAGCAGTTATCCTAATAATTTTTATAATTTGAGAAGCTTTCTCATCAAAACGTAAAGTCTCAACCATCGAACA
ACTATCTACTAACCTAGAATGGTTATATGGCTGTCCCCCTCCCTACCACACATTCGAAGAAGCCACCTATGTG
AAAACTCTAAGCGAAAAAGGAAGG????????????ATGGCAACACCAGCCCAATTAGGTTTACAAAACGCTA
CATCCCCCATCATAGAAGAACTTATTGCCTTCCATGACCATGCCCTTATAATTATTTTTTTAATTAGCTCTTT
AGTTTTATATATTATTTCCTTAATACTTACTACAAAACTTACTCACACTAGCACCATAAATGCTCAAGAAATC
GAAATAATCTGGACCATTTTACCCGCCGTCATTCTTATTATAATTGCTCTTCCATCTTTACGCATTCTATATA
TAACAGATGAGTTCAATAAACCTTACTTAACCCTTAAAGCAATTGGTCACCAATGATATTGAAGTTATGAATA
CTCCGACTATGAAGATCTAGCATTTGACTCCTATATTACGCCAACATACTTCCTAGAGCCCGGGGAATTTCGA
CTCCTTGAGGTAGATAACCGAACAACCTTACCTATGGAAGCAGACATTCGTGTACTAATTTCATCACAAGACG
TCCTACACTCATGGGCCGTACCAGCACTAGGTGTAAAAACAGATGCTATTCCCGGACGCCTAAATCAAGCCAT
ATTAACCTCTACACGACCAGGTTTATATTACGGACAATGCTCAGAAATTTGCGGGTCTAATCACAGCTTTATG
CCTATTGTCTTAGAATTCATTTACTTCCAAGACTTCGAAGTATGAGCCTCATATCTATACATTGTAT??????
ATGACCTCTCCCCGTAAAACTCACCCGCTAGCAAAAATTATCAACGAATCATTCATTGATCTCCCTACACCAT
CAAATATCTCCTCCTGATGAAATTTTGGCTCACTCCTAGGAACCTGTCTAATTATCCAAATCACCACAGGCCT
ATTCCTAGCAATACATTACACACCAGATACCTCTACTGCCTTTTCCTCAGTTGCCCATATTACTCGAGATGTT
AATTACGGATGAATAATCCGTTACTTACATGCTAATGGTGCATCCATATTTTTCATCTGCCTCTTTCTCCACA
TTGGGCGAGGCCTATACTACGGATCATTTCTTTTTCTGAAGACTTGAAATATCGGTACTATCCTACTACTTAC
AACCATAGCCACAGCATTCATAGGCTATGTGCTTCCATGAGGACAAATATCATTCTGGGGAGCCACAGTAATT
ACTAACCTCCTATCAGCCATTCCCTACATTGGGTCCGACTTAGTCCAATGGATTTGAGGCGGATTCTCAGTAG
ACAAAGCCACCCTAACACGATTCTTTACTTTCCACTTTATCTTGCCTTTCATCATCGCTGCCTTAGCAGCTAT
TCACCTACTATTTCTGCATGAAACAGGCTCAAGTAACCCATCAGGAATAACTTCAGAACCCGACAAAATCTCA
TTTCATCCCTACTATACAACTAAAGATATTCTCGGATTGATCTTCCTTATTCTATTCTTAATAAACTTAACTC
TATTCACCCCCGATTTATTAACAGACCCGGACAATTATACACTAGCTAATCCCCTAAACACCCCACCCCACAT
CAAGCCAGAATGATACTTCCTATTCGCATATGCAATCCTACGATCTATCCCCAACAAACTAGGAGGCGTTTTA
GCCCTAATACTCTCTATCCTAATTCTAACAATTATTCCCACAATACATCTATCCAAACAGCAAAGTATAACAT
TTCGACCAATCACTCAAATCCTATTCTGAGCCCTGGTGGCCGATTTATTAACACTAACCTGAATCGGAGGCCA
ACCAGTTGAATACCCATTTATCACAATTGGTCAAACAGCATCTATTATATACTTTTTAATCATCATCACTCTT
ATCCCCCTTTCTGCTCTAATCGAAAATAAACTTCTTAAATGATAA????????????????????????????
???
???
???

???
???
???
???
???
???
???
???????????????????????????GGAAGAGCTGCATCAGGCAAGTACTTTGATCTTTCTTGGAGCAGGT
TCCTTCAAAACGCTTCTCCAGAGGACGGAAGTCCCTCCAGTCTGGCTCACTGCTCGCAGTGGTGGGGAAAGCT
GAGGTAATTCCCCGGTGGGAAATCTGCGAGGCCCCTGATACCCCTG--------
CCACCAGGCCCATCACTGATGGGCCTAAGGGCCTCATCTGATACTCTAAGATGGGTTTCTCCTGTTGGATCTG
GGCCTCTCGGACTTGAGCCTCTCTAGAACGAGCCTCTCGGGCCTGGGCTTCTCGGGTTCTGGCCTTCCTGGCA
TGACCTTCCCAAGTGTGGGCCTCCCTG--
GCATAGGCTTCCTTGCCATGAGCTTCTAGAGTATAGGCCTCCCTGGTGTGGGCCTCCCTGGCCTGGGCTTCTC
GGGCACGTGCCTCCCGGGCCTCAAGCTGCTCCCTCCGAAGCTCCCAATACAACAACTGTTTCTGGATGGTCA-
ATAGCCGTTCCTCCTCTGTCTCCATTGCCCCAGGTGGCCGGGAGGAAGAAAAGGGCTCAAAGTTCAAGAAGGG
GTCAAACATCTCAGAGCTTCGACCACAAAGGTCATAAAGGCAGTCATCCCCAGGTGGAGAGTTCTCAAGACTG
TCATCTGGCTCATAGAACTCATATAGGGCATCTCCACTGTAGCTGTCTCGGGGCAGACAATCCCTGCGGACAA
GCCCCAGGGTCTCACCTGAATCATCCTCAA???????????TCACTAATTTTGATGAACTAAAAATGGTTCCTG
AAGGTCAGATTCATGTACAGTGAATGTTCTCCACTGTTGTAGTGGGCATGTTTCATGTGTGGGGATGTTGGTG
TGCAAGTTTCGTGTGTTTGGAAAACACTAGCTAGAACATTCGAAAAATTTCATTAACCCAGCTCTGGAAGGTC
ATCTATGAATTCTTCCTATTCCAGTGGCCTTTT-
AAGTGTAGATGGAGATACACTGTAATTTAAAACAGATGGGCATGTCTGAATATTTTCATATGCAAAATGGGAC
AGATGTATATGAATGGATAAATTATCCTTTTAAAAAAATACTTTGTAGCACGAAATATCACCAATGATTGGAA
GAGTGAACCTGCATTGGGTCTAGGGCAAACACCAGGAAGACATTATCAAAACATCTAA-
CTATCTACAAACTAATTCCAATTAGTCTACTTTGTCTTCATACAGAAAAAA-CTTTTTTGATTTTTAAA---
TATGTATTGGGTGCCAATCCGTATCCTTT-
GAGCTGCTAAAGCCAGCATACCACAATAGAAGTTTACTTTTCCTTGAGAGTTG????GTGTTTATAATCAACC
TACTCCTATTAATCGTACCAGCCCTAATCGCTATAGCATTTTTAACACTTACAGAACGAAAAATCTTAGGCTA
TATACAATTTCGAAAAGGCCCCAACATCGTGGGCCCGTATGGAGTACTCCAACCAATCGCTGATGCCATAAAA
CTTTTCACAAAAGAACCCCTACTACCCACTACCTCTACTACAACCTTATATCTAACTGCCCCAACCTTAGCCC
TATCTATCGCCCTTCTTCTATGAACTCCACTCCCCATACCATACCCTCTAATCAACCTTAACTTAGGTCTACT
ATTTATCCTAGCAACATCAAGCCTAGCTGTATACTCAATTCTATGATCCGGATGAGCATCCAACTCAAACTAC
GCACTAATTGGCTCATTACGAGCCGTAGCCCAAACAATCTCATACGAAGTTACTCTCGCCATTATCCTCCTAT
CTACCCTATTAATAAGTGGCTCATTTAACCTACAAACACTCATCACAACACAAGAGCACTATTGACTTCTTCT
TCCATCATGACCCCTAGCCATAATATGATTTATCTCCACACTAGCAGAAACCAATCGAGCCCCATTTGACCTA
ACAGAAGGAGAATCAGAACTAGTCTCAGGCTTTAATATTGAATATGCCGCGGGCTCATTCGCTTTATTCTTTA
TAGCAGAGTATATAAACATTATTATAATAAACGCCCTAACTACTACTATTTTTCTAGCAACACCCTATAAAAT
AACCACATCAGAAATTTACACAATTAACTTCATAACTAAAACTCTTTTACTAACCACCCTATTTCTATGAATT
CGAACAGCATACCCCCGCTTCCGCTATGATCAATTAATATACTTATTATGAAAAAACTTTTTACCCCTTACAC
TAGCACTATGCATGTGATATATCTCAATACCTGTTCTAACATCCGGCATTCCACCCCAAACAT??ATCAACCC
CCTAGCCTATCTTATCATTTCCTTCACTATCCTAACAGGAACCATAATCACAACTTTAAGCTCACATTGATTT
CTAGCCTGAATAGGCCTAGAACTAAATATACTAGCTATCGTACCAATTCTAGCCAAAAATACAAACCCCCGAT
CTACAGAAGCATCAACCAAATATTTCCTAATTCAAGCAACAGCATCAATAATCCTCCTAATAGCTATTTTCCT
CAATAATTTAATCTCCGGACAATGAACAATCAATCCACCCGATAACCAAATCTCATCTATAATAATATTAATT
GCCCTAGTAATAAAAATAGGAATAGCCCCCCTACACTTCTGACTCCCAGAAGTAACTCAAGGAACCCCCCTAA
TCCCAGCTATAGTAATCCTCACATGACAAAAAATTGCCCCCATATCAATTATACTTCAAATTTTTCCATCAAT
AAACACTACTATTATTCTAACAATTTCAATCCTGTCAATTATAATCGGTAGCTGAGGGGGACTTAACCAAACT
CAACTCCGTAAAATTCTAGCCTACTCCTCAATTACCCACATAGGATGAATAACAACAGTATTATACTACAACC
CAAATATCACTATTTTAACCCTAATCATTTATATCTTCTTAACAACCTCTACATTCATAATTTTTTTCCTAAA
TTCAAACGTAACAACCCTATCATTATCACATACCTGAAATAAATTAACATGAGTGATACCTATCATTCCATTA
ATAATAATATCTTTAGGAGGCTTACCTCCACTAACAGGTTTTTCCCCCAAATGAGCCATTATACAAGAACTTA
CAAAAAATAATAACTTAATCATTCCACTTACAATAGCCATACTAACACTAATAAACTTATACTTCTATATACG
CCTAACATACTCTATCTCAATAACAATATTTCCCACATCTAATAACACAAAAATTAACTGACAACTTAAACAC

ATAAAACCAACCCCACTCCTACCACCACTTATGATACTCTCTACATCACTCTTACCACTAACCCCACTAATAC
TAGTGATCT?????GTTGGCAGCATAAATAATAAAGCATGTTACTTAAGCAATTTTACATTTACTGTATTAGT
AAGTAAATGCAATGGCCACTCTTATTCTCAAACAATAAAAACTACCCAGCCTTTTCATGAAAGAT-
GATTGCAATTTCCATTGGCCTGTATATGCAATAAAAAG---
CCATAACTCATCAAAATTTAGCTATTTTCTCTTAAGATA--
GAAGAGTGTTTTGCTTGAACATTGAACGTGACATTAAATATT-
CATGAAACTGAATAGAGTTTAAGTACTAAG---
TAAAACTCTCAAATTCTTTTCAACTAGATTGAAATTCTGTTTGAACCAGGTAAAAACTACCGCTAACCTGAAG
AGTTGTTCAAAAAC-
ATCGTAAAGAGATTGTACAATATGTTCAAAGTGCTACAGTTATTTGGGATAGATTTTTCTGAAAAGCCTGTTC
CTCCTTTGAAATTGATTCATGATAACAGCCTCCATTTGGGTCAAACAGTATTTAACCGCTTGTAAA-
TTTTAATT-
AAATGATTTGTCACTTTGAACACATAGTTTGTCCCCAAGACCTTGAGGATTACTTGAATGGCCCCTTCACTGT
GGTTGTGAAGGAGTCTTGTGACGGAATGGGAGATGTGAGTGAGAAGCATGGGAGTGGGCCAGTAGTCCCAGAA
AAGGCAGTCCGTTTTTCATTTACAATCATGAAAATTACTATAGCCCACAACTCTCAGAACGTGAAAGTGTTTG
AAGAAGCCAAACCTAACTCTGAACTGTGTTGCAAGCCATTGTGCCTTATGCTGGCAGATGAGTCTGACCACGA
GACCCTGACTGCCATCCTGAGTCCTCTCATTGCCGAGAGGGAGGCCATGAAGAGCAGTGAATTAATGCTTGAG
ATGGGAGGCATTCTCCGGACTTTCAAGTTCATCTTCAGGGGCACGGGATATGATGAAAAACTTGTGCGGGAAG
TGGAAGGCCTTGAGGCTTCTGGCTCAGTCTACATCTGTACTCTTTGTGATGCCACCCGTCTGGAAGCCTCTCA
AAATCTTGTCTTCCACTCTATAACCAGAAGCCATGCCGAGAACCTGGAACGTTATGAAGTCTGGCGTTCCAAC
CCTTACCACGAGTCTGTGGAAGAACTGCGGGATCGGGTGAAAGGGGTGTCAGCCAAACCTTTCATTGAGACAG
TCCCTTCCATAGATGCACTCCACTGTGACATTGGCAATGCGGCTGAGTTCTACAAGATCTTCCAGCTAGAGAT
AGGGGAGGTGTATAAGAATCCCAATGCTTCCAAAGAGGAAAGGAAAAGGTGGCAGGCCACACTGGACAAGCAT
CTCCGGAAGAAGATGAACCTCAAACCAATCATGAGGATGAATGGCAACTTTGCCAGGAAGCTCATGACCAAAG
AGACTGTAGATGCAGTTTGTGAGTTAATTCCTTCTGAGGAGAGGCACGAGGCTCTGAGGGAGCTGATGGATCT
TTACCTGAAGATGAAACCAGTATGGCGGTCATCATGCCCTGCTAAAGA--GTGCCC-
AGAATCCCTCTGCCAGTACAGTTTCAATTCACAGCGTTTTGCTGAGCTCCTTTCTACCAAGTTCAAGTATAGG
TATGAGGGGAAAATCACCAATTATTTTCACAAAA???????????????????CCGACAGTTGTCATTATGTT
TGGAAACACTGCCAGCCCATGGAGATGAGGATAAAGTGGACATTTCTCTGCTGCATCAGGGTGAGAATCTTTT
TGAACTGCACATCCACCAGGCCTTCCTGACATCTGCCGCCCTGGCTCAGGYTGGAGACATGCAACCCACCACT
TTCTGCACCTATTCCTTCTATGACTTTGAAACCCATTGTACCCCATTGTCTGTGGGGCCACAGCCCCTCTATG
ACTTCACCTCCCAGTATGTGGTGGAGACAGACTCTCTTTTCTTACACTACCTTCAAGGGGCTTCAGTCCGGCT
TGATCTCCACCAGGCCGTGGCCAGTGAGCACAACACTCTCGCTGCAGGATGGATTTACTTTGACAGGGTGCTA
GAGACTGTGGAGAAAGTCCATGGCTTGGCCACACTGATTGGTAAGTGTTGTCAGCTTCCTGCAGCTCCTA-
GCACCAATGCAGAATTTCCCAAACTCTATAGCTGCTTTTCTGGTCATTTCTTATTTTCTTCCTTTTTCTTTGT
TCTTGATCATTGCCACACCATCTGTATTCTCCATGATTTCA-
TACTTGCTGCTGCCCAGGAGCTGGTGGAGAAGAGTTTGGGGTTCTAGAGTACTGGATGAGGCTGCGTTTCCCC
ATAAAACCCAGCCTACAGGCATGCAATAAACGAAAGAA???????????????????????????????????
?????????????????????????GTTCATGGAGGGAAAACACCAAACAATGAGCTTTCAGATAAGATTTA
TGTCATGTCTGTTGTTTCCAAGAACAACAAGAAGGTTACTTTTCGCTGCACGGAGAAAGACTTGGTAGGAGAT
GTTCCTGAAGCCAGATATGGTCATTCCATTAATGTGGTATATAGCCGAGGGAAAAGTATGGGTGTTCTTTTTG
GAGGACGCTCATACATGCCTTCTACCCACAGAACCACAGAAAAAATGGAATAGTGTAGCTGACTGCCTGCCCCA
TGTTTTCCTGGTGGATTTTGAATTCGGGTGTGCTACATCATACATTCTTCCAGAACTTCAGGATGGGCTATCT
TTTCATGTCTCTATTGCCAAAAAATGACACCATCTATATTTTAGGAGGACATTCACTTGCCAATAATATCCGCC
CTGCCAACCTGTACAGGATAAGGGTTGATCTTCCCCTGGGTAGCCCAGCTGT-
GAATTGCACAGTCTTGCCAGGAGGAATCTCTGTCTCCAG???????????????????????????????????
??
???TATGTCTCTGCATCTTCCTGCTC
TCCACCACACTGAGCCCAAGATGACCCAGTGCTCCACAAACATATGTAGAATGGAAGTAAAGCCTCAGGAATA
AGAAAAGATAATTAAAGGGCCAGGCATGGTGGTTCATGCCTGTAATCCCAGCACT-
TAGGAGGCCAAGGCAAGTGGATCACAAGGTCAGGAGTTCAAGACCAGCCTGGCCAAGATGGTGAAAACCCGTC
TCTACTAAAAATG--CAACAATT-----
AGCTGGG---
--------------------TGTGGGGGTGGGCGCCTGTAATCCCAATTACTTGGGAGGCTGAAGCAG-

AGAATTGCTTGAACCTGGGAGGCAGAGGTTGCAGTGAGCCGAGATCACGCCACTGCACTCCAGCCTGGACAAC
A-GAGTAAGACTCCATCTCAA--

---AAAA-
TAATAAKAAGAAGAAAAGATAGTTAAAGCAGATTCACTATAGTAACAAACCAGCCAAGAAATTGCACTGAAAG
TAAAGGTTTTAG----
TAAAATTTCCAACTGTGTTCAAAGAAACATAAGTTCCAGCAGGAGTGCACTTAATCTGATTTT-
CTTCAAAGC--
CTCTCTAATTTGCAGGGAAAGACATCCATTTTCATTAGCTTCATGTGAGCTAGAGAGAATCCATGACTGAAGT
GCTAGAACACCTGGTAAACACGCCAGCAAGCAAGGGCACCTTCCCGAGAGGCTCAACCTAGACTATAAGGCTG
TCAGCATTGGTAAGGAGCCTTAAACTCTAAACCCATAGGCTCAAATCAGGAA-
CTCAGGAAGCCTCTCAACACCGGGATACAATTTTGCCACCTTAACGCTTTATTCCCTTTACTTGTGCCACACC
???
???
???
???
???
?????????????????????????????????AAGGCCCTCAAAGATCTTTATTGCCATAAAGCAGAGTAAC
TTGTCTTTGACTTGGACCTTGTTTACTGTGAACTCTAATC-AAGGCAGGACATGCAGCAGTTCTGCTTA-
CTGCACTGTGGACATGTGGAAGGGACACTTGTGA---------
ATCCAGTTCGGATTAGTGTTTGAGATGGAATTGCAGATGTTGTTCCCGGATGTGGAATGCAGCCGTGGTCACA
CTTGCCTAC--GAGCTTACTTTGATCTGTTTGTCAGTAGCATGCAAAAAA-
TGCTTTGTTTGCCCTTTGCTTCTGCTTTTTTCAGGGAAGCTGCCAAAGAATGTCGACGTCGAAAGAAAGAATA
CGTAAAATGCCTGGAGAGCCGAGTCGCAGTGCTGGAAGTCCAGAACAAGAAGCTTATAGAGGAACTTGAAACC
TTGAAAGACATT???
???
???
???
???
???
???
???
???
???
???
??????????????????????????????????TCGAAAGAGTTATTGTAGTGTCAATGAACTACAGGGTGG
GTGCTCTAGGATTCTTAGCTTTGCCAGGAAATCCTGAGGCTCCAGGAAACATGGGCTTATTTGATCAACGGTT
AGCTCTTCAGTGGGTTCAAAAAAATATAGCAGCCTTTGGTGGAAATCCTAAAAGTGTAACTCTTTTTGGAGAA
AGTGCAGGAGCAGCTTCAGTTAGTTTGCATTTGCTTTCTCCTGGAAGCCATTCCTTGTTCACCAGAGCCATTC
TGCAAAGTGGATCCTCTAATGCTCCTTGGGCAGTAACATCTCTTTATGAAGCTAGGAACAGAACATTGGCCTT
AGCTAAATTTACTGGTTGCTTTAGAGAAAATGAGACTGAAATAGTCAAATGCCTTCAAAACAAAGATCCCCAA
GAAATTCTTCTGAATGAAGCATTTGTTGTCCCCTACGGAACTCTCTTGTCAGTAAACTTTGGTCCAACAGTGG
ATGGTGATTTTCTCACTGACATGCCAGACATATTACTTGAACTCGGACAATTAAAAAAAAACCCAGATTTTGGT
GGGTGTTAATAAAGATGAAGGGACAGCTTTTTTAGTCTATGGTGCTCCTGGCTTCAGCAAAGATAACAATAGT
ATCATAACTAGAAAAGAATTTCAGGAAGGTTTAAAAATATTTTTTCCAGGACTGAGTGAGTTTGGAAAGGAAT
CCATCCTTTTTCATTACACAGACTGGGTAGATGATCAGAGACCTGAAA?????????????????????????
???
???????????????????????TTGTGCTAATTGTGAGAAAATATGCTACTTTGATCAAACTAGAATGAAGG
AAATAAAAAGACACTGTGGCTCAACTTGTAATAAAGAGCAGCTCATCCTCACAGCTTTTTGATTGTTGCTATA
GTTATCT---
GTTCAACATGGGATGCACAATACTGTTAACGTTTCATTACCAGTAGCCTGTTGCGTGCTTATTGTTAAAAGGA
ATTTAACATACAAGTTTTTGTATGGATTGTTCGCCTAATTCCGATTCCTCGTGCTGACTGGCATGAGACAGGG
AGCTGGATTGTATTAAACTGGTTTTATTGTGATGTGACTATGATGGATGTGACAAGGACCTCTATTATTCACT
GGGGAGTGTGGCCGTTAATGAAGCCATTAGCTGAGCTAAGGCAAGTGAAGAAAATAGGGAAATTACCATTATT

CTGAAGCCATTAAGTTGTAATCCACCACTTTCTCTCCTTATTCAAAAAGGCATCTTCTTTTTAAGGCCTCCCT
?????????????????????TACTTCGGTTGCATGAAGGCTGCCCCCATGAAAGAAGCAAACGTCCGAGGAC
AAGGTGGCTTGGCCTACCCAGGTGTGCGGACCCATGGGACTCTGGAGAGTGTGAATGGGCCCAAGGCAGGTTC
AAGAGGCCTGACGTCGTCATTGGCTGACACTTTCGAACATGTGATAGAAGAGCTGTTGGATGAGGACCAGAAA
GTTCGGCCCCATGAAGAAAACAATAAGGACGCAGACTTGTACACGTCCAGGGTGATGCTCAGTAGTCAAGTGC
CTTTGGAGCCTCCTCTTCTCTTTCTGCTGGAGGAATACAAAAATTACCTGGATGCTGCAAACATGTCCATGAG
GGTCCGGCGCCACTCTGACCCTGCCCGCCGAGGGGAGCTGAGCGTGTGTGACAGTATTAGTGAGTGGGTAACG
GCGGCAGACAAAAAGACTGCAGTGGACATGTCGGGCGGACGGTCACAGTCCTTGAAAAGGTCCCTGTATCGA
AAGGCCAACTGAAGCAATACTTCTACGAGACCAAGTGCAATCCCATGGGTTACACAAAAGAAGGCTGCAGG??
?????????????????????ACAGCAGAACGGCTATGAAAATCCAACCTACAAGTTCTTTGAGCAGATGCA
GAACTAGACCCCC-
GCCACAGCAGCCTCTGAAGTTGGACAGCAAAACCATTGCTTCACTACCCATCGGTGTCCATTCATAGAATAAC
GTGGAAAGAAACAAACCCCTCTGTCTTATGATTTACTCATTACCGCCTTTTGACAGCTGTGCTGTAACACAAG
TAGATGCCTGAACTTGAATTAATCCACAGATCAGTAATGTATT--CTCTCTCTCTCT----TT----------
--------ACATTTTGGTCTCTACACTACATTATTAATGGGTTTTGTGTACTGTAAAGAATTTAGCTGTTTC-
AAACCTAGTGCATGA-
ATAGATTCTCTCCTGATTATTTATCGCATAGCCCCTTAGCCAGTTGTATATTATTATTGTGGTTTGTGACCCA
ACTAAGTCCTACTTTAAATATGCTTTAA-GAATCGGTGGGGGA---
TGCTTCACGTGAACGTGGGAGTTTAGCTGCTTCTCTTGCCTCAGTATTCCCTTTCT-
GATCACTATGCATTTTAAAGTTAAA-CATTTTTAAGTATTTCAGATGCTTTAGAGA-TTTTTTTTTC--
CATGATTGCATTTTACTGTACAGATTGCTGCTTCTGCTATATTTGTGATATAGGAATTAAGAGGATACACGTT
TGTTTCTTCGTGCCTGTTTTATG????TAATCTCACTTCCGGAGAGCACTAAGCCACTTCCAGCCCTGCTGTG
AACCAGGACCGTAGGAGCCATCTTTTTCATGTGATCAGCTTTGGAAGAATCTACATCCACCACTTTAGAAGAC
AAGTCTAGTGGCTTATCCGTGACATCTTTGGTAACAGTCTGCTTCTCCAACAGAGGAGGTGAGCTGCCGTCTT
TTCTGTCTTGAACCGCTGTCTTCCGGGAATGCCCGGGCACTGGCTGGGCACCTTCGCCCCCTTCTGGAGCCTT
GGGATACTTGCTGTTGGAGAGCCTGGCCGCGGGGAACTCGCTGCTGACTGTCATGTATGGCTTTGACAGGGCA
ACTGAAGGAGAGGTGGAGATCCTGGCATAGTGCTTGTGGAACTCCGAGAAGGTGTCTGCAGCAGGCTGGGTGG
GAAGGTGGACCCGGGGTGAGGGCCGAGGCGAGGGGGGCAACAGGAGAGCCGTGTCCCCCGGCAGGCCACTGGT
GACTGCCTTGGCAGAGGGAACCCTGGGCTGTTTACTGTTCTGGATGTGAGGATAGGCATGGGAATCAACAGGA
TTCCCAGGGCTGACCCCCATCTTCCATGGGAGGCTTTTGTCTGCGCAATGGACAAGAGGTGGGATGGCTGGGG
AGGCCGAAGGCGTCGAGAGCCTCATCGGTGATGCCAAGGACGATGGGATGTGGGGACTGACGTAGTGAGGTGG
TGGCAGGTAGAGAAAGCGCTCCCCGTTGGTGCAGACTGGAGAATACAGCGGCTGGGCCAAGCTGTAGGACTGC
TGAGGTAGCAAGGTGCCAGGACCTTTGTAATTCAAGTGCTTTTTACAGTAGTAGCAAATGTTACATAAGCCCA
AACATGGAGCA---
TATTTGGAGTTGGTGGGCAGACCCAAATCATGATGCAAGCCTGCAGTAGCAAGTGTGCAATGAAGCACAACCA
AATTCTGTGAATGGAAGGTAGCGAGACGCCTCTCCAAAGCAGCCCTTCTGCGCAGGAGCTGAAAGCTCCACCG
GGTAAGTTTTCTGAGGCAGTTTTCAGAGGCTGGGCTTGGCTTAGGGCCCAGCTCTGGATGAGCCTGTGGACGG
TGGGGT----
AGGTCTTTGCTAACCTTACATAGAATCAAGGAGAGTTTGGAAGAGTTTGTGCAGGCCTCTTTCCTCCTGGTGT
TTGTCTTGCCCTGCCAGCAGGCCAGGAGGG-
CTGCGCTGCTCAAGTAGTGGAGGGAGGGAGCCTGCAGAGCTAGGGGATCAGAGTGGGTAATTGCAGCCACAGG
GATTAGCTGCTGCAAGCTGTCCACAGGAGCCATTCTGAAATGTCATTTGAGTGATCTGGCCGTCTACTGGAGG
AGGCCCTCGGGGCAGCTGTCTCCAGGAGCAGCTTCTGAGGGAGTCTTCCAGGAGGAGCAGTGTCTCTGCCATC
ATCCTCGTCCACGTCCTGGTCACATTTCCATTCCTCATCTTCATTCAGAGTCGGCAGGTATCCAGATATGCCC
TTCCCTGTCCCTGACCCAGAGCTCTGGTCACTACAGACCTTCGGGCTCTCCGAGCCTGTCCTCGTATATTCCA
AATAAATATCAGACTTAAGGAAGGAGGGGTAGGTGTTTTCCTCCATGGTGGCCTGGATTTCTGTCTGGGCCTG
GTCAAACATGGCAGGATCGATCAGCTGCTTCATGATGCAGCCCTTTATGAAGCTCTTGGTGGCCGGCTTGGTC
TGCCGGGACACGATGCCATTGTTATCAAGAATGTACTTTCGGTAGATGGCTCTGGCCAGCTTCAGCCTCTTCT
CCTCGTTTGAGTCACAGGGCTCCAGCTTCCTGAAGCCAGTGCAGGCAAACCAGAAGTCCAGCAAGTCAGCACA
GCCCTCCTGCTTCAGGAAAGTCCTGAACAGGCTTATCCCATCTTGATCATCCAGCAGGGAGTGCAGTGACTCA
GCCCACTTCAAGTACGGTGGGGTGGGGGAGGCACTGCCCTCAGGCTCATACCCCAGGTCCAGATCCGAGCGCC
TCGGAGTGGCGGTCGAAGTCTCACCTTTAATGCCAACACCTTTCCCGGAGCAGAAACTGTAGCTGGCAGGCCT
CAGGTCTGTGGACACCAGTTCTCCCTCCTCACCAGGCACTGGGGGTCGGGGAGCATCTTCGGTGAAACTTGCT
CCAAGGTCCAAGGGGAAACCCTGCTCCTGGACATTCATTTTGGGACTCTGTGCGTCAATGCACAACGAGCGCT

GCACCCTAATACATCAGTACTTAACAGCTCCAAAGTCAATCAATCTGTCCTGTTGAAACCAT???????????
TGTTAGCTCATTTTAAGTGAACAA-A--
ATTACATGGATCAACTGATGGCAGCATGAAACAGGCATAATTTGCAGTGCAGCAGGTTAAGT-
ACTTAGGTTATTTATAGGACAGATTATTTTTCTGTGTTGTTTTG----CGTCTTTG-
CATATTAAAATTTATTTATAGGTGTAGTTAAGCTTT--TCTATTAGTTAGCATAAATTTGGCTTGGAGTCA--
--
CCAAAGCAGAAATGTTGGACTTGATTTTTATGAGTTATTTCCTACACATTTCTAAACTGATGTAGCTTTGTCA
CTGCTTGATTGATTTTTTTTTGTAAACTGGAGGGGGTAAAATTAGTTTATATTGAATCATACCAGTTTCTCTT
AATTAGTA-------------------
CATTACTAACATTGAAAAGAAATAAAAGGAATGTTAATAACTTAGGAGTATTTGCATAGTTA-
CATTCAGAACACAAGCTTTAAATTAGT-
GAGGCAACCACTAATTTAAGAAGTTTGATTTAAGCACTCATACTCCTTTATTTTCCTTTGTTAGCAATAATTA
TTGGCCCTGATGGCCATCCTTTGACTGTTTATCCTTGTATGATTTGTGGGAAGAAGTTTAAGTCAAGAGGTTT
TTTGAAAAGACACATGAAAAACCATCCCGAGCATCTTGCCAAGAAGAAGTACCACTGTACTGACTGTGATTAC
ACTACCAACAAGAAGATAAGTTTACACAACCACCTGGAAAGCCACAAACTGACCAGCAAGGCAGAGAAGGCCA
TTGAATGTGATGAGTGTGGGAAGCATTTTTCTCATGCAGGGGCTTTGTTTACTCACAAAATGGTGCACAAAGA
AAAGAGAGCCAACAAAATGCACAAGTGTAAATT??
???????????????????????????????????????CCCGCTATAGCTTCTGCAAACAGTAAAGATACCA
CTAAAACCCCTTCTGCAGTTCAAACTAGCACCAGCCACAACCCTGGACTTCCTCCTAATTTTAACGAATGGTA
CGTCTGAGGAAAAACACAAACCCTGTTAACTATAGAATGGACCAAATGCATTTTTAAAAGAAAACTGAGACCA
ATCAGATGGAAATGGAGTTTTAAGGCAAGAGGCCATATATAGGGCTACATCTTGTTAATTGCAATTGTCCAGG
AAGGTTTTGGGCAAGATCCAGAAGTAGCCATGCCCTTTTCTCAGGATTAGAAAATATGTTTTGGCGTTTGA-
GGATTTTTT--
ACAAAATCTTTACACTGCTTTTTCTTCCCCTTCCTCTTGCTCTCTGCACACCCCATTCCTAAACTCTTGCAAT
TCAATTTAACACTTGTCCTGTTTCTTGAGAGGAAGTTATAGAAGGCTTGTTGGTGATGGTGATGTTAAACTGA
TGGAAATTCTTTTTCGCCTTAGTGGTGATTGTTTAAACTCTCACAGTCTTAAACCGTGCCAAAGTCCTGTTAA
?
Callithrix
?TACCCCCATGTTTGGCTGGAACATGAAGCTGACCTCAGAGTACTACAGAAACGTCACCTTCCTGTCATGCCG
GTTCCCTT-
CCGTCATGAGCATGGACTACATGGTATACTTCAGCTTCTTCACCTGGATTTTCATTCCTCTGGTCATCATGTG
TGC--AATCTATCTTGACATCTTTTACAT-
CATCCGGAGCAAACTCAGACAGAACTTCTCTAACTCCAAAGAGACAGGCGCATT---
TTACGGACGGGAGTTCAAGACGGCGAAGTCCCTGTTTCTGGTGCTTTTCTTGTTTGCTTTGTCATGGTTGCCT
TTGTCCATCGTCAACTGCGTCACCTACTTTCA--------------
TGGTGAGGTACCACAGCTTGTGCTGCACTTGGGCATCCTGCTGTCCCATGCCAACTCCATGATGAACCCTAT?
????????????????????ATGTTCATAAATCGATGATTATTTTCAACCAACCACAAAGACATCGGAACTC
TATACTTATTATTTGGTGCATGAGCAGGGGCAGTAGGAACAGCCCTAAGCCTCCTAATCCGAGCAGAGCTAGG
CCAACCAGGGAGCCTGATAGAAGATGATCATGTTTACAATGTTATCGTCACATCTCACGCATTTATTATAATT
TTTTTTATGGT---AATACCAATTATAATTGGGGGCTTCGG---
AAACTGACTTGTCCCCTTAATAATTGGCGCCCCAGACATAGCATTTCCCCGAATAAATAACATGAGCTTTTGA
CTCCTACCCCCC--TCACTACTTCTCCTACTAG-
CATCCTCTACCCTTGAGGCCGGTGCCGGAACTGGTTGAACAGTCTACCCGCCCTTAGCAGGTAACATATCACA
CCCAGGAGCTTCTGTAGACCTGACCATCTTTTCATTGCATCTAGCAGGTGTATCTTCCATTTTAGGGGCTATC
AACTTTATTACAACAATCATTAACATAAAACCCCCAGCCATGACCCAATACCAAACCCCCCTATTCGTATGAT
CTGTACTAATTACAGCAGTCCTCCTTCTACTTTCCCTCCCAGTTCTAGCTGCCGGAATTACTATACTACTAAC
TGACC--
GCAACCTCAACACTACCTTCTTCGACCCTGCTGGTGGCGGAGACCCAGTTCTATATCAACACCTATTCTGATT
TTTCGGCCACCCTGAAGTATATATTCTTATTCTACCAGGTTTCGGAATAATTTCGCACATTGTAACGTACTAC
TCCAATAAAAAAGAGCCATTTGGCTACATGGGCATGGTCTGAGCTATAATGTCTATTGGCTTCCTAGGCTTTA
TTGTATGAGCCCACCATATATT-
CACAGTAGGAATAGATGTAGACACTCGCGCATATTTCACATCAGCCACTATAATCATCGCAATCCCTACCGGA
GTGAAAGTATTTAGCTGGTTAGCTACGCTACACGGCGGTAATATTAAATGATCCCCCGCAATATTATGGGCCT
TAGGCTTTATCTTTCTTTTCACAGTGGGCGGGTTGACAGGAATCGTATTAGCCAACTCATCATTAGACAT-

TGTATTACACGACACATACTATGTAGTAGCACACTTCCATTATGTGTTATCCATGGGAGCAG-
TATTTGCTATTATAGGGGGCTTTATCCACTGGTTTCCACTATTTTCAGGCTATACACTTGACCAAACCTACGC
TAAAGTTCATTT-
TACTATTATATTTGTAGGCGTAAATTTAACCTTCTTTCCACAGCACTTCCTTGGTCTATCCGGAATGCCCCGA
CGATACTCAGATTACCCAGATGCGTACACAACATGAAATATCGTATCATCCGTAGGGTCCTTCATCTCATTAA
CAGCAGTTATTCTTATAATTTTCATGATTTGAGAAGCATTCTCCTCAAAACGAAAAGTCTCTACCATCGAACA
GCTATCCACCAATCTAGAGTGACTATACGGCTGCCCTCCACCCTACCACACATTTGAAGAAGCCACCTATGTA
AAAACCT?????????????????????????????ATGGCAGCACCAGCCCAATTAGGCCTACAAAACGCCG
CATCCCCAATCATAGAAGAACTTATTGCCTTCCATGACCATGCTCTAATAATCATCTTCTTAATTAGCTCACT
AGTTCTATATATCATCTCTCTAATACTTACTACAAAACTGACACACACCAGTACCATGAACGCTCAAGAAATC
GAAATGATCTGAACTATCCTACCTGCAATAATCCTCATCATAATTGCCCTTCCATCCCTACGCATTTTATATA
TAACAGACGAGTTTAATAAACCATATCTAACCCTTAAAGCAATCGGCCACCAATGATACTGAAGCTACGAATA
CTCCGACTATGAAGACTTAGCATTCGACTCCTACATCATACCAACATACTTCCTTGAACCCGGGGAATTCCGA
CTCCTTGAAGTAGACAACCGAACAACCTTACCTATGGAAGCAGATATCCGTGTATTAATTTCATCACAAGACG
TTTTACACTCATGAGCTGTACCTTCACTAGGCGTGAAAACCGATGCAATCCCCGGACGCTTGAATCAAGCCAT
AGTAGCTTCCATACGACCAGGCCTATACTACGGACAATGCTCAGAAATTTGCGGGTCTAACCACAGCTTTATG
CCCATTGTACTAGAATTTATTTATTTCCAAGATTTCGAAGTATGAGCCTCATACTTATACATTGTAT??????
ATGACCTCCCCCCGCAAAACCCACCCACTGGCAAAAATCATCAATGAATCATTCGTCGACCTCCCCACACCAT
CTAACATCTCCTCTTGGTGGAATTTTGGCTCACTTCTAGGCACCTGCCTAATTATTCAAATCACTACAGGCCT
ATTCCTAGCAATACACTACACACCAGACACCGCTACCGCCTTCTCCTCAGTTGCCCACATCACCCGAGACGTC
AACTACGGCTGAATAATCCGATATCTACATGCTAACGGCGCATCCATATTCTTTATTTGCCTGTTCCTCCACG
TCGGTCGAGGGTTATATTACGGATCGTTCCTCTTTCTGAAGACTTGAAACATCGGTACAATCTTACTACTTGC
AACCATAGCTACGGCATTCATAGGCTACGTGCTACCATGAGGTCAAATGTCATTCTGAGGCGCCACAGTAATC
ACAAACCTTCTTTCAGCCATCCCCTACATTGGATCCGACTTAGTCCAATGAATCTGAGGCGGATTTTCAGTAG
ACAAAGCCACCCTCACACGATTCTTTACCTTCCACTTTATTTTACCTTTCATCATCGCAGCCCTAGCCACCAT
TCACCTCTTGTTTCTGCATGAGACAGGTTCAAGTAATCCGTCAGGAATTACCTCTGAACCGGACAAAGTCCCC
TTCCATCCCTACTATACAACTAAAGATATCCTCGGACTGACTTTCCTATTCCTAGCCCTAACAAGCTTAACCC
TATTCACACCCGACCTATTAACTGACCCAGATAACTATACACTAGCAAACCCCCTAAACACCCCACCCCACAT
TAAACCAGAGTGGTACTTCTTATTCGCATATGCAATCCTGCGATCCATCCCTAATAAGTTAGGAGGAGTACTA
GCTTTAGCAATATCCATCCTAGTGCTAATAATTATTCCCATAACACACACATCCAAACAACAAAGCATAGCAT
TCCGACCAATTACCCAGATCATGTTCTGAACGCTAGTAGCTGACCTATTAACCCTTACATGAATTGGAGGTCA
ACCAGTAGAACACCCATTTATTGCTATTGGCCAGACAGCTTCAATCATATACTTTCTCATCATCATCACCCTA
ATCCCCCTCTCCGCTCTCATCGAAAACAAGCTACTGAAGTGATAG????????????????????????????
???
???
???
???
???
???
???
???
???
???
???????????????????????????GGAAGAGCTGCATCAGGCAAGTACTTTGATCTTTCTTGGAGCAGGT
TCCTTCAAAACGCTTCTCCAGAGGACGGAAGTCCCTCCAGTCTGGCTCACTGCTCGCAGTGGTGGGGAAAGCT
GAGGTAATTCCCCGGTGGGAAATCTGCGAGGCCCCTGATACCCCTG--------
CCACCAGGCCCATCACTGATGGGCCTAAGGGCCTCATCTGATACTCTAAGATGGGTTTCTCATGTCGGACCTG
GGCCTCTCGGACTTGAGACTCTCTAGAACGAGCCTCTCGGGCCTGGGCTTCTCGGGTTCTGGCCTCCCTGGCA
TGACCTTCCCAAGTGTGGGCCTCCCTG--
GCATAGGCTTCCTTGCCATGAGCTTCTCGAGTATAGGCCTCCCTGGTGTGGGCCTCCCTGGCCTGGGCTTCTC
GGGCACGTGCCTCCCGGGCCTCAAGCTGCTCCCTCCGAAGCTCCCAATACAACAACTGTTTCTGGATGGTCA-
ATAGCCGTTCCTCCTCTGTCTCCATTGCCCCAGGTGGCCGGGAGGAAGAAAAGGGCTCAAAGTTCAAGAAGGG
GTCAAACATCTCAGAGCTTCGACCACAAAGGTCGTAAAGGCAGTCATCCCCAGGTGGAGAGTTCTCAAGACTG
TCATCTGGCTCATAGAACTCATATAGGGCATCTCCACTGTAGCTGTCTCGGGGCAGACAATCCCTGTGGACAA

GCCCCAGGGTCTCACCTGAATCATCCTCAA??????????TCACTAATTTTGATGAACTAAAAATGGTTCTTG
AAGGTCAGATTCATGTACAGTGAATGTTCTCCACTGTTGTGGTAGGCATGTTTCATGTGTGGGGATGTTGGTG
TGCAAGTTTCGTGTGTTTGGAAAACACTAGCTAGAACATTCGAAAAATTTCATTAACCCAGCTGTGGAAGGTC
ATCTATGAATTCTTCCTATTCCAGTGGCCTTTT-
AAGTGTAGATGGAGATACACTGTAATTTAAAACAGATGGGCATATCTGAATATTTTCATATGCAAAATGGGAC
AGATGTATATGAATGGATAAATTATCCTTTTAAAAAAATACTTTGTAGCATGAAATATCACCCATGATTGGAA
GAGTGAACCTGCATTCTGTCTAGGGCAAACACCAGGAAGACATTATCAAAACATCTAA-
CTATCTACAAACTAATTTCAATTAGTCTACTTTGTCTTCATACAGAAAAAA-CTTTTTCGATTTTTAAA---
TATGTATTGGGTGCCAATCCATATCCTTT-
GAGCTGCTAAAGCCAGCATATCACAATAGAAGTTTACTTTTCCTTGAGAGTTG????GTGTTTATAGTTAACT
TAATTCTACTAATCGTACCTGCCCTAATCGCTATAGCATTCCTGACACTTATAGAACGAAAAGTCTTAGGCTA
CATACAACTCCGGAAGGGACCAAACATTGTAGGGCCGTACGGAACACTCCAACCAATCGCCGACGCCATAAAA
CTTTTCACAAAAGAACCCTTACTACCTACCACTTCTACTACAACCCTATACCTAACTGCCCCAACCTTAGCCC
TCTCCATCGCCCTCCTTCTATGAACTCCACTCCCCATACCATACCCCCTAATCAACCTAAATCTAGGCCTTCT
CTTTATCCTAGCAACATCAAGCCTAGCTGTTTATTCAATCCTATGATCCGGCTGAGCATCCAACTCAAACTAC
GCACTAATTGGCGCATTACGAGCCGTAGCACAAACAATCTCATACGAAGTCACCCTCGCCATCATCCTCCTAT
CCACCCTATTAATAAGCGGCTCATTCAATCTACAATCACTAATTACAACACAAGAACACTACTGACTTCTACT
CCCATCATGACCCCTAGCCATGATATGATTTATTTCCACATTAGCAGAAACCAACCGAGCCCCCTTTGATCTC
ACTGAGGGCGAATCAGAACTAGTTTCAGGCTTCAACATTGAGTACGCCGCAGGCTCATTTGCCCTATTCTTTA
TAGCAGAGTACATAAATATTATTATAATAAATGCCCTAACCACCACCATCTTTCTAGCAACATCATTCAACAT
AACCATACCAGAAATATATACAATCAACTTTATAACCAAAACCCTCCTACTAACCACCCTATTCCTATGAATT
CGAACAGCATACCCTCGATTCCGCTATGACCAACTAATATATCTCCTGTGAAAAAATTTCTTACCTCTCACAC
TAGCACTATGTATATGATACATTTCAGTACCCATCTTAACATCCGGCATCCCCCCACAAACATA?ATTAACCC
CCTAGCCCACCTTATCATTTCCCTAACCATCCTAACAGGAACTACAATCACTATCCTAAGCTCGCACTGATTT
CTAGCCTGAATAGGCCTAGAACTAAATATACTGGCCATCGTACCAATTTTAGCCAAAAACACAAACCCCCGGT
CAACAGAAGCCTCCACCAAATATTTTCTAATCCAAGCAACAGCATCAATAATTCTCCTAATAGCCATCTTCCT
TAACAACCTAGCCTCCGGACAATGGACAATCAACCCTCCCCATAACCAAACTCTATCTACAATAATACTAATC
GCCCTAGTAATAAAAATAGGAATAGCCCCCCTCCACTTCTGACTCCCAGAAGTAACCCAAGGAACCCCCCTAA
TCCCAGCCATAATCGTCCTCACATGACAAAAACTTGCCCCCTTGTCAATTATATTTCAAATCTTCCCATCAAC
AAACACGAGTATTATCCTAATGATCTCAATCCTATCAATTATGGCCGGCAGTTGAGGGGGGCTTAACCAAACC
CAACTACGAAAAATCCTAGCCTACTCCTCAATCACACACATAGGGTGAATAATAGCCGTACTATACTATGACC
CAAACATCACCATTCTAACACTTATTATTTATATTTTCCTAACAATCTCCACATTCATAATTTTTTACCTAAA
CTCAAATATAACAACCCTATCACTATCACATACCTGAAATAAATTAACATGAATAATACCCATAATCCCACTA
ATAATAATATCATTAGGAGGCCTACCCCCATTAACAGGATTCTCCCCTAAATGAGCCATTATACAAGAACTTA
CAAAAAACAACAACCTAATCATCCCACTCACAATAGCCATGCTAACACTAATAAACCTCTACTTCTACATGCG
CCTAACATATTCCATCTCAATAACAATATTCCCCACATCAAACAACACAAAAATTAACTGACAACTAAAGCAC
ACAAAACCAATACCACTCCTACCCCCACTCATAACCCTTTCCACATTAATACTACCGCTAACCCCACTAATAC
TCACAAACT?????GTTGGCAGCATAAATAATAAAGCATGTTACTTAAGCAATTTTACATTTACTGTATTAGT
AAGTAAATGCAATGGCCACTCTTATTCTCAAACAATAAAAACTACCCAGCCTTTTCATGAAAGAT-
GATTGCAATTTCCATTGGCCTGTATATGCAATAAAAAG---
CCATAACTCATCAAAATTTTAGCTATTTTCTCTTAAGATA--
GAAGAGCGTTTTGCTTGAATATTGAACGTGACATTAAATATT-
CATGAAACTGAATAGAGTTTAAGTACTAAG---
TAAAACTCTCAAATTCTTTTCAACTAGATTGAAATTCTGTTTGAACCAGGTAAAAACTACCGCTAACCTGAAG
AGGTGTTCAAAAAC-
ATCGTAAAGAGATTGTACAATAYGTTCRAAGTGCTACAGTTATTTGGGATAGATTTTTCTGAAAAGCCTGTTC
CTCCTTTGAAATTGATTCATGATAACAGCYTCCATTTGGGTCAAACAGTATTTAACCGCTTGTAAA-
TTTTAATT-
AAATGATTTGTCACTTTGAACACATAGTTTGT?CCCAAGATCTTGAGGATTACTTGAATGGCCCCTTCACTGT
GGTTGTGAAGGAGTCTTGTGACGGAATGGGAGATGTGAGTGAGAAGCATGGGAGTGGGCCAGTAGTCCCGGAA
AAGGCAGTCCGTTTTTCATTCACAATCATGAAAATTACTATAGCCCACAACTCTCAGAACGTGAAAGTGTTTG
AAGAAGCCAAACCTAACTCTGAACTGTGTTGCAAGCCATTGTGCCTTATGCTGGCAGATGAGTCTGACCACGA
GACCCTGACTGCCATCCTGAGTCCTCTCATTGCCGAGAGGGAGGCCATGAAGAGCAGTGAATTAATGCTTGAG
ATGGGAGGCATTCTCCGGACTTTCAAGTTCATCTTCAGGGGCACTGGATATGATGAAAAACTTGTGCGGGAAG

TGGAAGGCCTTGAGGCTTCTGGTTCAGTCTACATCTGTACTCTTTGTGATGCCACCCGTCTGGAAGCCTCTCA
AAATCTTGTCTTCCACTCTATAACCAGAAGCCATGCCGAGAACCTGGAACGTTATGAGGTCTGGCGTTCCAAC
CCTTACCACGAGTCTGTGGAAGAACTACGGGATCGGGTGAAAGGGGTCTCAGCCAAACCTTTCATTGAGACAG
TCCCTTCCATAGATGCACTCCACTGTGACATTGGCAATGCGGCTGAGTTCTATAAGATCTTCCAGCTAGAGAT
AGGGGAGGTGTATAAGAATCCCAATGCTTCCAAAGAGGAAAGGAAAAGGTGGCAGGCCACATTGGACAAGCAT
CTCCGGAAGAAGATGAACCTCAAACCAATCATGAGGATGAATGGCAACTTTGCCAGGAAGCTCATGACTAAAG
AGACTGTAGATGCAGTTTGTGAGTTAATTCCTTCTGAGGAGAGGCACGAGGCTCTGAGGGAGCTGATGGATCT
TTACCTGAAGATGAAACCAGTATGGCGGTCATCATGCCCTGCTAAAGA--GTGCCC-
AGAATCCCTCTGCCAGTACAGTTTCAATTCACAGCGTTTTGCTGAGCTCCTTTCTACCAAGTTCAAGTATAGG
TATGAGGGGAAAATCACCAATTATTTTCACAAAA????????????????CACCCGACAGTTGTCATTATGTT
TGGAAACACTGCCGGCCCATGGAGATGAGGATAAAGTGGACATTTCTCTGCTGCATCAGGGTGAGAATCTTTT
CGAACTGCACATCCACCAGGCCTTCCTGACATCTGCCGCCCTGGCTCAGGCTGGAGACATGCAACCTACCACT
TTCTGCACCTATTCCTTCTATGACTTTGAAACCCATTGTACCCCATTGTCTGTGGGGCCACAGCCCCTCTATG
ACTTCACCTCCCAGTATGTGGTGGAGACAGACTCTCTTTTCATACACTACCTTCAAGGGGCTTCAGCCCGGCT
TGATCTCCACCAGGCCGTGGCCAGTGAGCACAACACTCTTGCTGCAGGATGGATTTACTTTGACAGGGTGCTA
GAGACTGTGGAGAAAGTCCATGGCTTGGCCACACTGATTGGTAAGTGTTGTTGGCTTCCTGCAGCTCCTA-
GCACCAATGCAGAATTTCCCAAACTCTATAGCTGCTTTTCTGGTCGTTTCTTATTTTCTTCCTTTTTCTTTGT
TCTTGATCATTGCCACACCATCTGTATTCTCCATGATTTCA-
TACTTGCTGCTGCCCAGGAGCTGGTGGAGAAGAGTTTGGGGTTCTAGAGTACTGGATGAGGCTGCGTTTCCCC
ATAAAACCCAGCCTACAGGCATGCAATAAACGAAAGAA???????????????????????????????????
???????????????????????????GTTCATGGAGGGAAAACACCAAACAATGAGCTTTCAGATAAGATTTA
TGTCATGTCTGTTGTTTCCAAGAACAACAAGAAGGTTACTTTTCGCTGCACGGAGAAAGACTTGGTAGGAGAT
GTTCCTGAAGCCAGATATGGTCATTCCATTAATGTGGTATATAGCCGAGGGAAAAGTATGGGTGTTCTTTTTG
GAGGACGCTCATACATGCCTTTTACCCACAGAACCACAGAAAAATGGAATAGTGTAGCTGACTGCCTACCCCA
TGTTTTCCTGGTGGATTTTGAATTCGGGTGTGCTACATCATACATTCTTCCAGAACTTCAGGATGGGCTATCT
TTTCATGTCTCTATTGCCAAAAATGACACCATCTATATTTTAGGAGGGCATTCACTTGCCAATAATATCCGCC
CTGCCAACCTGTACAGGATAAGGGTTGATCTTCCCCTGGGTAGCCCAGCTGT-
GAATTGCACAGTCTTGCCAGGAGGAATCTCTGTCTCCAG??????????????????????????????????
???
???CGTGTCTCAGCATCTTCCTGCTC
TCCACCACACTGAGCCCAAGATGACCCAGTGCTCCACAAACATATGTAGAATGGAAGTAAAGCCTCAGGGATA
AGAAAAGATAATTAAAGGGCCAGGCATGGTGGTTCATGCCTGTAATCCCAGCACT-
TAGGAGGCCAAGGTGAGTGGATCACAAGGTCAGGAGTTCAAGACCAGCCTGGCCAAGATGGTGAAAACCCATC
TCTACTAAAAATG--CAACAGTT-----
AGCTGGG--
---------------------TGTGGTGCTGGGCGCCTGTAATCCCAATTACTTGGGAGGCTGAAGCAA-
AGAATTGCTTGAACCTGGGAGGCAGAGGTTGCAACGAGCCGAGATCATGCCACTGCACTCCAGCCTGGACAAC
A-GAATAAGACTCCATCTCAA--

---AAAA-TAATAATAAGAA---
AAGATAGTTAAAGCAGATTCACTATAGTAACAAACCAGCCAAGAAATTGCACTGAAAGTAAAGGTTTTAGGTG
ACAAAATTTCCAACTGTGTTCAAAGAAACATAAGTTCCAGCAGGAGTGCACTTAATCTGATTTT-
CTTCAAAGC--
CTCTCTAATTTGCAGGGAAAGACAACCATTTTCATTAGCTTCATGTGAGCTAGAGAGAAGCCATGACTAAAGT
GCTAAAACCCCTGGGAAACATGCCAGCAAGCAAGGGCACCTTCCCCAGATGCTCAACCTAGACTATAAGACTG
TCAGCATTGGTAAGGAGCCTTGAACTCTAAACCCATAGGCTCAAGTCAGGAA-
CTCAGGAAGCCTATCAACACCGGGATACAATTTTGCCACCTTAACACTTTATTCCCTTTACTTGTGCCACACT
ACGACGTGGGGCCGCCGCCAGCGCCGGATTGCTGGGCGGCGGCGCCGCAGCTGAGCGGCGGTCGGGCTCGGCG
TCTCCACCTCCTCGCGTCCGTAATCAGTGACGAGGTCCGCTACGTAAATCCCTTCGCGGCGGCTGCCACTGGT
GACATGCCAACTTACCAGATCCGAGCTCCTACTACTGGTTTGCCACAGGGAGTGGTGATGGCTGCGTCGCCCG
GAAGTTTGCACAGTCCCCAGCAGCTGGCAGAAGAAGCAACACGCAAACGAGAGCTGAGGCTAATGAAGAACAG
GGAAGCTGCCCGGGAGTGTCGCAGGAAGAAGAAAGAATATGTCAAATGTCTTGAAAATCGTGTGGCTGTGCTT
GAAAACCAAAACAAGACTCTCATTGAGGAACTCAAGGCCCTCAAAGATCTTTATTGCCATAAAGCAGAGTAAT
TTGTCTTTGACTTGGACCTTGTTTACTGTGAACTCTAATC-AAGGCAGGACATGCAGCAGTTCTGCTTA-

CTGCACTGTGGACATGTGGAAGGGACACTTGTGA---------
ATCCAGTTCGGATTAGTGTTTGAGATGGAATTGCAGATGTTGTTCCCGGATGTGGAATGCAGCCGTGGTCACA
CTTGCCTACA-GAGCTTACTTTGATCTGTTTGTCAGCAGCATGCAAAAAA-
TGCTTTGTTTGCCCTTTGCTTCTGCTTTTTTCAGGGAAGCTGCCAAAGAATGTCGACGTCGAAAGAAAGAATA
TGTAAAATGCCTGGAGAGCCGAGTCGCAGTGCTGGAAGTCCAGAACAAGAAGCTTATAGAGGAACTTGAAACC
TTGAAAGACATTTGTTCTCCCAAAACAAATTAGTAGAAATATTTAACTATGAACTGAGGACAGCATGTACAGT
TGCTTTTGAAGAAATACAATATACAGCTGGCAAGAACGGTGGCTTTTTTTCTTTGTATCATTCGCTTCTCGAA
TCTCTTAACATTCCTAAAATGCTTCACTGTACATAGTTAAGTCAAAGCTCTAACTTCAAATTTTTTAAAAGAC
ACAAACTGTAAAAAATGTATGTAACAGATTCTTAGAATGCAATATTTGTAAGACTTGTTCCAGTGCCACATAT
GTGCAGTTCCCATTGTCTGTGGCATGAATAGTGTCCTATGCAGTAAAATTATTTGCAGGTCTTTAAATCATTT
TAGGAAAGGATGATCAAAAATAATGCATCCAGCAGTTCAGTAAAAGGAAACCACAAAAAAAAATACCTCAGGAA
GTGATTGAGAGAAAGTCTATCTAATGACATGCCTGTATGAGAATAGTCTAGATACAAATTTATGGCATTTGCA
GATTTTTATATTAGTTACTTTGTAAAAAAAAAAAAAAAAAGATTGTATTCTGTCCTTGAATGCCATAGTCGAA
GAGAGTTTTTAATAGAACCATGTTGGTTGCATTTTGTAGTGTTCGGTGCTCATTTAAATATCTGAACATTTAC
TACAGTTTTTAACTCTATTGTGTAATATAAAGGTCTTGCAGAAGTTTGTTTATATCTAAATGTGTGGATTCCA
GCACCTAAACCAAAAAATTCCACTGTAATGATATGG-
ATTTATGGTGGTGGTTTTCAAACTGGAACATCATCCTTACATGTTTATGATGGCAAGTTTCTTGCTTGGGTCG
AAAGAGTTATTGTAGTGTCAATGAACTACAGGGTGGGTGCTCTAGGATTCTTAGCTTTGCCAGGAAATCCTGA
GGCTCCAGGAAACATGGGTTTATTTGATCAACGGTTGGCTCTTCAGTGGGTTCAAAAAAATATAGCAGCCTTT
GGTGGAAATCCTAAAAGTGTAACTCTTTTTGGAGAAAGTGCAGGAGCAGCTTCAGTTAGTTTGCATTTGCTTT
CTCCTGGAAGCCATTCCTTGTTCACCAGAGCCATTCTGCAAAGTGGATCGTCTAATGCTCCTTGGGCAGTAAC
ATCTCTTTATGAAGCTAGGAACAGAACATTGGCCTTAGCTAAATTTACTGGTTGTTTTAGAGAAAATGAGACT
GAAATAGTCAAATGCCTTCAAAACAAAGATCCCCAAGAAATTCTTCTGAATGAAGCATTTGTTGTCCCCTACG
GAACTCTCTTGTCAGTAAACTTTGGTCCAACAGTGGATGGTGATTTTCTCACTGACATGCCAGACATATTACT
TGAACTTGGACAATTTAAAAAAACCCAGATTTTGGTGGGTGTTAATAAAGATGAAGGGACAGCTTTTTTAGTC
TATGGTGCTCCTGGCTTCAGCAAAGATAATAATAGTATCATAACTAGAAAAGAATTTCAGGAAGGTTTAAAAA
TATTTTTTCCAGGAGTGAGTGAGTTTGGAAAGGAATCCATCCTTTTTCATTACACAGATTGGGTAGATGATCA
GAGGCCTGAAAACTACCGTGAGGCCTTGGATGACGTTGTCGGGGATTACAATATTATATGCCCAGCCTTGGAG
TTTACAAAGAAGTTCTCAGAATGGGGAAACAATGCCTTTTTCTACTATTTGAACACCGTTGTGCTAATTGTG
AGAAAATATGCTACTTTGATCAAACTAGAATGAAGGAAATAAAAAGACACTGTGGCTCAACTTGTAATAAAGA
GCAGCTCATCCTCACAGCTTTTTGATTGTTGCTCTAGTTATCT---
GTTCAACATGGGTTGCACAATACAGTTAACATTTCATTACCAGTAGCCTGTTGCGTGCTTATTGTTAAAAGGA
ATTTAACATACAAGTTTTTGTATGGATTGTTCGCCTAGTTCCGATTCCTCGTGCTGACTGGCATGAGACAGGG
AGCTGGATTGTATTAAACTGGTTTTATTGTGATGTGACTATGATGGATGTGACAAGGACCTCTATTATTCACT
GGGGAGTGTGGCCGTTAATGAAGCCATTAGCTGAGCTAAGGCAAGTGAAGAAAATAGGGAAATTACCATTATT
CTGAAGCCATTAAGTTGTAATCCACCACTTTCTCTCCTTATTCAAAAAGGCATCTTCTTTTTAAGGCCTCCCT
?????TACTATGGTTATTTCATACTTCGGTTGCATGAAGGCTGCCCCCATGAAAGAAGCAAACATCCGAGGAC
AAGGTGGCTTGGCCTACCCAGGTGTGCGGACCCATGGGACTCTGGAGAGTGTGAATGGGCCCAAGGCAGGTTC
AAGAAGCCTGACATCGTCATTGGCTGACACTTTCGAACATGTGATAGAAGAGCTGTTGGATGAGGACCAGAAA
GTTCGACCCCATGAAGAAAACAATAAGGACGCAGACTTGTACACGTCCAGGGTGATGCTCAGTAGTCAAGTGC
CTTTGGAGCCTCCTCTTCTCTTTCTGCTGGAGGAATACAAAAATTACCTGGATGCTGCAAACATGTCCATGAG
GGTCCGGCGCCACTCTGACCCTGCCCGCCGAGGGGAGCTGAGCGTGTGTGACAGTATTAGTGAGTGGGTAACG
GCGGCAGACAAAAAGACTGCAGTGGACATGTCGGGCGGACGGTCACAGTCCTTGAAAAGGTCCCTGTATCGA
AAGGCCAACTGAAGCAATACTTCTACGAGACCAAGTGCAATCCCATGGGTTACACAAAAGAAGGCTGCAGGGG
CATAGACAA??????????????CAGCAGAACGGCTATGAAAATCCAACCTACAAGTTCTTTGAGCAGATGCA
GAACTAGACCCCC-
GCCACAGCAGCCTCTGAAGTTGGACAGCAAAACCATTGCTTCACTACCCATCGGTGTCCATTTATAGAATAAT
GTCGAAAGAAACAAACCCCTCTGTCTTATGATTTACTCATTACCGCCTTTTGACAGCTGTGCTGTAACACAAG
TAGATGCCTGAACTTGAATTAATCCACAGATCAGTAATGTATTTTCTCTCTCTCTCT----TT----------
--------ACATTTTGGTCTCTACACTACATTATTAATGGGTTTTGTGTACTGTAAAGAATTTAGCTGTTTC-
AAACCTAGTGCATGA-
ATAGATTCTCTCCTGATTATTTATCGCATAGCCCCTTAGCCAGTTGTATATTATTCTTGTGGTTTGTGACCCA
ACTAAGTCGTACTTTAAATATGCTTTAA-
GAATCGGTGGGGGATGATGCTTCACGTGAACGTGGGAGTGTAGCTGCTTCTCTTGCCTCAGTATTCCCTTCCT

-GATCACTATGCATTTTAAAGTTAAA-CATTTTTAAGTATTTCAGATGCTTTAGAGA-ATTTTTTTCC--
CATGATTGCATTTTACTGTACAGATTGCTGCTTCTGCTATATTTGTGATATAGGAATTAAGAGGATACACGTT
TGTTTCTTCGTGCCTGTTTTATG????TAATCTCACTTCCGGAGAGCACTAAGCCACTTCCAGCCCTGCTGTG
AACCAGTACCGTAGGAGCCATCTTTTTCATGTGGTCAGCTTTGGAAGAATCTACATCCACCACTTTAGAAGAC
AAGTCTAGTGGCTTATCTGTGACGTCTTTGGTAACAGTCTGCTTCTCCAACAGAGGAGGTGAGCTGCCGTCTT
TTCTGTCTTGAACCGCTGTCTTCCGGGAATGCCCGGGCACTGGCTGGGCACCTTCGCCCCCTTCTGGAGCCTT
GGGATACTTGCCGTTGGAGAGCCTGGCCGCGGGGAACTCGCTGCTGACTGTCATGTATGGCTTTGACAGGGCA
ACTGAAGGAGAGGTGGAGATCCTGGCATAGTGCTTGTGGAACTCCGAGTAGGTGTCTGTAGCAGGCTGGGTCG
GAAGGTGGACCCGGGGTGAGGGCCGAGGCGAGGGGGGCAACAGGAGAGCCGTGTCCCCCGGCAGGCCACTGGT
GACTGCCTTGGCAGAGGGAACCCTGGGCTGTTTACTGTTCTGGATGTGAGGATAGGCATGGGAATCAACAGGA
TTCCCAGGACTGACCCCCATCTTCCATGGGAGGCTTTTGTCTGCGCAATGGACAAGAGGTGGGATGGCTGGGG
AGGCCGAAGGCGTCGAGAGCCTCATTGGTGATGCCAAGGACGAAGGGATGTGGGGACTGACATAGTGAGGTGG
TGGCAGGTAGAGAAAGCGCTCCCCGTTGGTGCAGACTGGAGAATACAGCGGCTGGGCCAAGCTGTAGGACTGC
TGAGGTAGCAAGGTGCCAGGACCTTTGTAATTCAAGTGCTTTTTACAGTAGTAGCAAATGTTACATAAGCCCA
AACATGGAGCAGCATATTTGGAGTTGGTGGGCAGACCCAAATCATGATGCAAGCCTGCAGTAGCAAGTGTGCA
ATGAAGCACAACCAAATTCTGTGAATGGAAGGTAGCGAGATGCCTCTCCAAAGCAGCCCTTCTGCGCAGGAGC
TGAAAGCTCCACCGGGTAAGTTTTCTGAGGCAGTTTTCAGAGGCTGGGCTTGGCTTAGGGCCCAGCTCTGGAT
GAGCCTGTGGACGGTGGGGT----
AGGTCTTTGCTAACCTTACATAGAACCAAGGAGAGTTTGGAAGAGTTTGTGCAGGCCTCTTTCCTCCTGGTGT
TTGTCTTGCCCTGCCAGCAGGCCAGGAGGGGCTGCGCTGCTCAAGTAGTGGAGGGAGGGAGCCTGCAGAGCTA
GGGGATCAGAGTGGGTAATTGCAGCCACGGGGATTAGCTGCTGCAAGCTGTCCACAGGAGCCATTCTGAAATG
TCATTTGAGTGATCTGGCCGTCTACTGGAGGAGGCCCTCGGGGCAGCTGTCTCCAGGAGCAGCTTCTGAGGGA
GTCTTCCAGGAGGAGCAGTGTCTCTGCCATCATCCTCGTCCACGTCCTGGTCACATTTCCATTCCTCATCTTC
ATTCAAAGTCGGCAGGTATCCAGATATGCCCTTCCCCGTCCCTGACCCAGAGCTCTGGTCGCTACAGACCTTC
GGGCTCTCCGAGCCTGTCCTCGTATATTCCAAATAAATATCAGACTTAAGGAAGGAGGGGTAGGTGTTTTCCT
CCATGGTGGCCTGGATTTCTGTCTGGGCCTGGTCAAACATGGCAGGATCGATCAGCTGCTTCATGATGCAGCC
CTTTATGAAGCTCTTGGTGGCCGGCTTGGTCTGCCGGGACACGATGCCATTGTTATCAAGAATGTACTTTCGG
TAGATGGCTCTGGCCAGCTTCAGCCTCTTCTCCTCGTTCGAGTCACAGGGCTCCAGCTTCCTGAAGCCAGTGC
AGGCAAACCAGAAGTCCAGCAAGTCAGCACAGCCCTCCTGCTTCAGGAAAGTCCTGAACAGGCTTATCCCATC
TTGATCATCCAGCAGGGAGTGCAGTGACTCAGCCCACTTCAAGTACGGTGGGGTGGGGGAGGCACTGCCCTCA
GGCTCATACCCCAGGTCCAGATCCGAGCGCCTCGGAGTGGCGGTCGAAGTCTCACCTTTAATGCCAACACCTT
TCCCGGAGCAGAAACTGTAGCTGGCAGGCCTCAGGTCTGTGGACACCAGTTCTCCCTCCTCACCAGGCACTGG
GGGTCGGGGAGCATCTTCGGTGAAACTTGCTCCAAGGTCCAAGGGGAAACCCTGCTCCTGGACATTCATTTTG
GGACTCTGTGCGTCAATGCACAACGAGCGCTGCACCCTAATACATCAGTACTTAACAGCTCCAAAGTCAATCA
ATCTGTCCTGTTGAAACCAT??????TAAAATGTTAGCTAATTTTAAGTGAACAA-A--
ATTACATGGATCAACTGATGGCAGCATCAAACAGGCATAATTTACAGTGCAGCAGGATAAGTTACTTAGGTTA
TTTATAGGACAGATTATTTTTCTGTGTTGTTTTG----CGTCTTTG-
CATATTAAAATTTATTTATAGGCATAGTTAAGCCTATAACTATTAGTTAGCATAAATTTGGCTTGGAGTCA--
--
CCAAAGCAGAAATGTTGGACTTCATTTTTATGAGTTATTTCCTACACATTTCTAAACTGATGTAGCTTTGTCA
CTGCTTGATTGATGTTTTTT---
AAACTGGAAGGGGTAAAATTAGTTTATACTGAATCATACCAGTTTCTCTTAATTAGTA---------------
----CATTACTAACATTGAAAAGAAGTAAAAGGAATGTTAATAACTTAGGAGTATTTGCATAGTTA-
CGTTCAGAACACAAGCTTTAAATTAGT-GAGGCAACTA---------
GAAGTTTGATTTAAGCACTCATACTCCTTTATTTTCCTTTGTTAGCAATAATTATTGGCCCTGATGGCCATCC
TTTGACTGTTTATCCTTGTATGATTTGTGGGAAG???????????????????????????????????????
???
???
???
???
???????????????????ACCCGCTATAGCTTCTGCAAACAGTAAAGATACCACTAAAACCCCTTCTGCAGT
TCAAACTAGCACCAGCCACAACCCTGGACTTCCTCCTAATTTTAACGAATGGTACGTCTGAGGAAAAACACAA
ACCCTGTTAACTATAGAATGGACCAAATGCATTTTTAAAAGAAAACTGAGACCAATCAGATGGAAATGGAGTT
TTAAGGCAAGAGGCCATATATAGGGCTACATCTTGTTAATTGCAATTGTCCAGGAAGGTTTCGGGCAAGATCC

AAAACTAGCCATGCCCTTTTCTCAGGATTAGAAAATATGTTTTGGCGTTTGA-GGATTTTTT--
ACAAAATCTTTACACTGCTTTTTCTTCCCCTTCCTCTTGCTCTCTGCACACCCCATTCCTAAACTCTTGCAAT
TCAATTTAACACTTGTCCTGTTTCTTGAGAGGAAGTTATAGAAGGCTTG---
GTGGTGGTGATGTTAAACTGATGGAAATTCTTTTTCGCCTTAGTGGTGATTGTTTAAACTCTCACAGTCTTAA
ACCGTGCCAAAGTCCTGTTAT?
Cebuella
TTACCCCCATGTTTGGCTGGAACATGAAGCTGACCTCAGAGTACTACAGAAACGTCACCCTCCTGTCATGCCG
GTTCCCTT-
CCGTCATGAGCATGGACTACATGGTATACTTCAGCTTCTTCACCTGGATTTTCATTCCTCTGGTCATCATGTG
TGC--AATCTATCTTGACATCTTTTACAT-
CATCCGGAGCAAACTCAGACAGAACTTCTCTAACTCCAAAGAGACAGGCGCATT---
TTACGGACGGGAGTTCAAGACGGCGAAGTCCCTGTTTCTGGTGCTTTTCTTGTTTGCTTTGTCATGGTTGCCT
TTGTCCATCGTCAACTGCGTCACCTACTTTCA--------------
TGGTGAGGTACCACAGCTTGTGCTGCACTTGGGCATCCTGCTGTCCCATGCCAACTCCATGATGAACCCTATC
?????????????????????ATGTTCATAAATCGCTGATTATTTTCAACCAATCACAAAGACATCGGAACTC
TATACTTACTATTTGGTGCATGAGCAGGAGCAGTGGGGACAGCCTTAAGCCTCCTAATCCGAGCAGAATTAGG
CCAGCCAGGAAGCCTAATGGAGGATGATCATGTTTATAATGTTATTGTTACATCTCACGCATTCATTATAATC
TTCTTCATGGT---AATACCAATCATAATTGGGGGCTTTGG---
AAACTGACTTATCCCTTTAATAATTGGCGCTCCCGATATAGCATTCCCCCGAATAAATAACATAAGCTTCTGA
CTTTTACCTCCT--TCACTCCTCCTCCTACTCG-
CATCCTCTACCCTTGAAGCTGGCGCTGGAACTGGCTGAACGGTTTACCCGCCCTTAGCAGGTAACATATCACA
CCCAGGGGCTTCTGTAGACCTAACTATTTTTTCATTACATTTAGCAGGCATCTCGTCCATCCTAGGGGCTATT
AACTTCATTACAACAATCATCAACATAAAACCCCCAGCCATAACACAATACCAAACTCCTCTATTCGTATGAT
CCGTCCTAATTACAGCAGTTCTTCTCCTACTTTCCCTACCAGTCCTAGCTGCCGGAATTACTATACTGCTAAC
TGACC--
GTAATCTCAATACTACCTTCTTCGACCCTGCTGGTGGCGGAGATCCCGTTCTATATCAACACCTGTTCTGATT
TTTGGTCACCCCGAAGTATATATTCTCATTCTACCAGGTTTCGGAATAATTTCACACATTGTAACATACTAC
TCCAATAAAAAAGAACCATTTGGCTATATAGGCATGGTCTGAGCTATAATATCCATCGGTTTCCTAGGGTTCA
TTGTATGGGCCCACCATATATT-
CACGGTAGGAATAGATGTAGACACCCGCGCATATTTCACATCAGCCACTATAATCATCGCAATCCCGACCGGG
GTAAAAGTATTTAGCTGACTAGCTACGCTACACGGCGGCAATATTAAGTGATCCCCTGCAATACTATGAGCCC
TGGGCTTTATCTTTCTTTTCACCGTGGGCGGATTGACAGGAATTGTACTAGCCAATTCATCATTAGACAT-
TGTATTACATGATACATATTATGTCGTAGCACACTTCCATTACGTGCTATCTATGGGAGCAG-
TATTCGCTATTATAGGGGGCTTTATTCACTGATTCCCACTATTTTCAGGCTACACACTCGACCAAACCTATGC
TAAAGTCCATTT-
TACTATTATATTTGTAGGGGTAAATTTAACCTTCTTCCCACAGCACTTCCTGGGCCTATCAGGAATACCTCGA
CGATACTCAGATTACCCAGATGCATACACCACATGAAACATCATCTCATCCGTAGGGTCCTTCATCTCACTAA
CAGCAGTTATCCTCATAATTTTCATGATTTGAGAAGCATTCTCCTCAAAACGAAAAGTCTCTACCATCGAACA
ACTATCTACTAACCTAGAATGGCTACATGGCTGCCCTCCACCCTACCACACATTTGAAGAAGCAACCTATGTA
AAAGCCCTGAACGAAAAAGGAAGG????????????ATGGCAGCACCAGCTCAATTAGGCCTACAAAACGCCG
CATCCCCAATCATAGAAGAACTTATCGCCTTCCATGACCACGCTCTAATAATCATTTTCTTAATTAGCTCCCT
AGTTTTATACGTTATTTCTCTGATACTCACTACAAAATTGACCCACACTAGTACCATAAATGCTCAAGAAATC
GAAATAATCTGAACTATCCTACCCGCTATAATCCTCATTATAATTGCCCTTCCATCCCTACGCATTTTATATA
TAACAGACGAATTTAATAAACCGTACCTAACTCTCAAAGCGATTGGTCACCAATGATATTGAAGCTATGAATA
CTCTGACTATGAAGACCTAGCATTCGACTCTTATATTATGCCAACATATTTCCTTGAACCCGGGGAATTTCGA
CTCCTTGAAGTGGACAACCGAACTACCCTGCCCATAGAAGCAGATATTCGCGTATTAATCTCATCACAAGACG
TTCTACACTCATGAGCTGTACCATCATTAGGTGTAAAAACAGATGCAATCCCCGGACGCTTGAATCAGGCTAT
AGTAGCTTCCATACGACCAGGCCTATACTACGGACAGTGCTCAGAAATCTGTGGATCAAACCACAGCTTCATG
CCCATTGTACTAGAATTTATCTACTTCCAAGATTTCGAAGTATGAGCCTCATACCTATACATTGTAT??????
ATGATTCCTCCCCGTAAAACCCACCCACTGGCAAAAATCATAAATGAATCATTCATTGACCTTCCCACACCAT
CCAACATCTCCTCTTGATGAAATTTTGGCTCACTCCTAGGTACCTGCCTAATTATTCAAATCACTACAGGCCT
ACTCCTAGCAATACACTACACACCAGACACAGCTACCGCCTTCTCCTCAGTCGCCCATATCACCCGAGACGTC
AATTACGGATGAATAATTCGATACTTACATGCTAATGGCGCATCCATATTTTTTATTTGCCTATTCCTCCACA
TCGGCCGAGGCTTATACTACGGATCGTTCCTTTTTCTGAAGACTTGAAACATCGGTACAATCCTACTACTTAC

AACCATAGCCACAGCATTTATAGGCTACGTACTACCATGGGGCCAAATATCATTCTGAGGGGCCACAGTAATT
ACAAATCTTCTATCAGCCATCCCCTACATTGGATCCGACCTAGTCCAATGGATCTGAGGCGGGTTTTCAGTAG
ATAAAGCCACTCTCACACGATTCTTCACCTTCCACTTTATCCTACCTTTCATTATCGCAGCCCTAGCTACCAT
CCACCTTCTATTCCTGCATGAAACAGGTTCAAGTAACCCATCAGGAATCACCTCTGAACCAGATAAAATTCCA
TTCCACCCATACTATACAACTAAAGATATCCTTGGACTTACATTCCTCCTCCTAATACTAACAAGTCTAACCC
TATTTACACCCGACCTACTAACAGACCCAGACAACTACACACTAGCAAACCCCTTAAACACCCCGCCCCACAT
CAAGCCAGAATGATACTTCCTATTTGCATATGCAATCCTACGATCCATCCCCAACAAATTAGGAGGAGTTCTA
GCTCTAGCAATGTCTATCCTAATCCTAATATTTGTCCCCATAACACACCTGTCCAAACAACAAAGCATAGCAT
TCCGACCAATTACTCAAGTCATATTCTGAACACTAGTAGCTGACCTATTCACTCTCACATGAATTGGAGGCCA
ACCAGTCGAACACCCATTCATCGCCATTGGCCAAACAGCTTCTATTATATATTTCTTATCATTATTACCTTA
ATCCCCCTTTCCTCCCTCATTGAAAACAAACTACTAAAATGATAA?TTAGGTTAAATTTATTCAAGGGAATGA
GAATTTATGGGTATAACTGTTAGCTGGAAAGCCCTTCCTCACCTCCAATCCTCCTTTCAAACTACATTTCC-
TCCTGAAATTTCTCTGTGGGATGAAATGTTCGATTATTGGTCAATCAATC----
TCTTATATGTTAYCTGGCTTTACATAT----
GCCCTTGATAGTTCTTGGAAAATGATACAATATTGTGTTGTTTCGGTGTCTTTGTATTGGAAATGGAAGCATT
TTGTATGAATCACCTCTTTAACTGTGAGGAGTTGAATTGTCTTAGTTCTGCTGGCCTAATTACCAGGAATAAA
--GACATCATAGTCTCTAACCCCACTTTTT-
GGTGGAAAGAAAATCTAAAAGTAGCAACATTTAATSGAACAAAATATCAATAATCAAAGAAACTCCCCTGTGA
CACCGTAATATTATTTT-
AAAATCAACATTGTTCTAAGGTAGCTGAAATGTGTGATTTTTCTATTTTGCCTCTTCACTGACAATATACCCA
TAAATATTTGGTGGTCATTTTCTCTCCAAAGTTAATTTCTGGGCCATTGGCGACTGCACTATCTATTGTTCTG
AATTGTTGTCTTAATTGCATTCTTTAAAAATGTAATGTTGAAGGCACATAATATTGAAGATGACTCCAGGATG
AACCCTGAGTTCGCAGACCGAA-
TAAAACAGCTTGATAAAGAGGCGTCTTACTACCGTGATGAGTGTGGCAAGGCCCAAGCGGAAGTGGACCGGTT
GCTGGAGACATCACTCTGGAAGAGCTGCATCAGGCAAGTACTTTGATCTTTCTTGGAGCAGGTTCCTTCAAAA
CGCTTTTCCAGAGGACGGAAGTCCCTCCAGTCTGGCTCACTGCTCGCAGTGGTGGGGAAAGCTGAGGTAATTC
CCCGGTGGGAAATCTGCGAGGCCCCTGATACCCCTG--------
CCACCAGGCCCATCACTGATGGGCCTAAGGGCCTCATCTGATACTCTAAGATGGGTTTCTCATGACGGGCCTG
GGCCTCTCGGACTTGAGACTCTCTAGAACGAGCCTCTCGGGCCTGGGCTTCTCGGGTTCTGGCCTCCCTGGCA
TGACCTTCCCAAGTGTGGGCCTCCCTG--
GCATAGGCTTCCTTGCCATGAGCTTCTCGAGTATAGGCCTCCCTGGTGTGGGCCTCCCTGGCCTGGGCTTCTC
GGGCACGTGCCTCCCGGGCCTCAAGCTGCTCCCTCCGAAGCTCCCAATACAACAACTGTTTCTGGATGGTCA-
ATAGCCGTTCCTCCTCTGTCTCCATTGCCCCAGGTGGCCGGGAGGAAGAAAAGGGCTCAAAGTTCAAGAAGGG
GTCAAACATCTCAGAGCTTCGACCACAAAGGTCGTAAAGGCAGTCATCCCCAGGTGGAGAGTTCTCAAGACTG
TCATCTGGCTCATAGAACTCATATAGGGCATCTCCACTGTAGCTGTCTCGGGGCAGACAATCCCTATGGACAA
GCCCCAGGGTCTCACCTGAATCATCCTCAA??????AGCATCACTAATTTTGATGAACTAAAAATGGTTCCTG
AAGGTCAGATTCATGTACAGTGAATGTTCTCCACTGTTGTGGTAGGCATGTTTCATGTGTGGGGATGTTGGTG
TGCAAGTTTCGTGTGTTTGGAAAACACTAGCTAGAACATTCGAAAAATTTCATTAACCCAGCTGTGGAAGGTC
ATCTATGAATTCTTCCTATTCCAGTGGCCTTTT-
AAGTGTAGATGGAGATACACTGTAATTTAAAACAGATGGGCATATCTGAATATTTTCATATGCAAAATGGGAC
AGATGTATATGAATGGATAAATTATCCTTTTAAAAAAATACTTTGTAGCATGAAATATCACCCATGATTGGAA
GAGTGAACCTGCATTCTGTCTAGGGCAAACACCAGGAAGACATTATCAAAACATCTAA-
CTATCTACAAACTAATTTCAATTAGTCTACTTTGTCTTCATACAGAAAAAA-CTTTTTTGATTTTTAAA---
TATGTATTGGGTGCCAATCCGTATCCTTT-
GAGCTGCTAAAGCCAGCATATCACAATAGAAGTTTACTTTTCCTTGAGAGTTGCATCGTGTTTATAATTAACT
TAATCCTACTAATCGTACCCGCCCTAATTGCTATAGCATTCCTGACACTTACAGAACGAAAAGTCTTAGGTTA
TATACAATTCCGAAAAGGACCCAACATTGTAGGACCTTACGGAATACTCCAACCAATCGCCGACGCCATAAAA
CTTTTCACAAAAGAACCCCTACTACCTACCACTTCTACTACAACTCTATACCTGACCGCCCCAACTTTAGCCC
TCTCCATCGCCCTACTTTTATGGACCCCACTCCCTATACCATACCCCCTAATAAACCTAAACCTAGGCCTCCT
ATTTATTCTAGCAACGTCAAGCCTAGCTGTTTATTCAATTTTATGGTCCGGCTGAGCATCCAACTCAAACTAT
GCACTAATCGGCGCATTACGAGCCGTAGCCCAAACAATCTCATATGAAGTAACCCTTGCCATCATTCTCCTAT
CCACTCTACTAATAAGCGGCTCATTCAATTTACAATCACTCATTACAACACAAGAACACTACTGACTCCTACT
TCCATCATGACCTCTAACCATAATATGATTTATTTCCACACTAGCAGAAACCAACCGAGCCCCGTTTGACCTA
ACAGAAGGTGAATCAGAATTAGTATCAGGCTTTAATATTGAATATGCCGCAGGCTCATTCGCCTTATTCTTTA

TAGCAGAATATATGAACATTATCATAATAAATGCCCTAACCACCACCATTTTCCTAGCAGCATCATTCAACAT
AGCCATGCCAGAAATATATACAATCAACTTTATAACTAAGACCCTCCTACTAACCACCCTATTTTTATGAATT
CGAACAGCATATCCTCGATTCCGCTATGATCAACTAATGTACCTCCTATGAAAAAATTTCTTGCCTCTCACAC
TAGCACTATGCATGTGATATATTTCAATACCCATCTTAATGTCTGGCATCCCTCCACAAACATA?ATCAACCC
CCTAGCCCACCTCATTATCTCCTTAACTATCCTAACAGGAACCACAATTACAATTCTAAGCTCGCACTGATTT
CTAGCCTGAATGGGCTTAGAACTAAACATGTTGGCCATCGTACCAATTCTAGCCAAAAGTACAAATCCCCGAT
CCACAGAAGCTTCTACCAAATATTTTCTAATCCAAGCAACAGCATCAATAATCCTCCTAATGACCATCTTCCT
CAACAACTTAACCTCCGGACAATGAACAATTAACCCACCCCATAATCAAACTTTATCAACAATAATGCTAATC
GCACTCATAATAAAAATGGGAATAGCCCCCCTACACTTCTGACTTCCAGAAGTAACCCAAGGAATTCCCCTAA
TCCCAGCCATAATTGTCCTCACATGACAAAAACTTGCTCCCTTGTCAATTATATTTCAAATCTTCCCATCAAT
AAACACGAACATTATCCTAATGGTCTCAATCCTATCAATTATAGCCGGCAGCTGAGGGGGACTAAACCAAACA
CAACTACGAAAAATCCTAGCCTACTCCTCAATTACCCATATAGGATGAATAGTAGCAGTACTACACTACAACC
CAAACATCACCATTCTAACCCTTATTATCTATATTTTCCTGACAATCTCCACATTCATAATCTTCTACCTAAA
CTCAAACATAACAACCCTATCACTATCACACACCTGAAACAAACTAACATGAATAATACCCATAATTCCACTA
ATAATGATATCATTAGGAGGCCTACCCCCACTAACAGGCTTCTCCCCTAAATGAGCTATTATACAAGAACTTA
CAAAAAATAACAGCCTAATTATCCCACTCACAATAGCCATACTAACACTAATAAACCTATATTTCTATATACG
CCTAACATATTCCATCTCAATAACAATATTTCCTACATCAAACAATACAAAAATTAACTGACAACTAAAGCAC
GTAAAACCAATGCCACTCCTACCACCACTCATAGCCTTTTCCACATTATTGCTACCTCTAACCCCACTAATAC
TTACAAACT?????GTTGGCAGCATAAATAATAAAGCATGTTACTTAAGCAATTTTACATTTACTGTATTAGT
AAGTAAATGCAATGGCCACTCTTATTCTCAAACAATAAAAACTACCCAGCCTTTTCATGAAAGAT-
GATTGCAATTTCCATTGGCCTGTATATGCAATAAAAAG---
CCATAACTCATCAAAATTTTAGCTATTTTCTCTTAAGATA--
GAAGAGCGTTTTGCTTGAATATTGAACGTGACATTAAACATT-
CATGAAACTGAATAGAGTTTAAGTACTAAG---
TAAAACTCTCAAATTCTTTTCAACTATATTGAAATTCTGTTTGAACCAGGTAAAAACTACCGCTAACCTGAAG
AGGTGTTCAAAAAC-
ATCGTAAAGAGATTGTACAATACGTTCAAAGTGCTACAGTTATTTGGGATAGATTTTTCTGAAAAGCCTGTTC
CTCCTTTGAAATTGATTCATGATAACAGCCTCTATTTGGGTCAAACAGTATTTAACCGCTTGTAAA-
TTTTAATT-
AAATGATTTGTCACTTTGAACACATAGTTTGTCCCCAAGATCTTGAGGATTACTTGAATGGCCCCTTCACTGT
GGTTGTGAAGGAGTCTTGTGACGGAATGGGAGATGTGAGTGAGAAGCATGGGAGTGGGCCCGTCGTCCCGGAA
AAGGCAGTCCGTTTTTCATTCACAATCATGAAAATTACTATAGCCCACAAGTCTCAGAACGTGAAAGTGTTTG
AAGAAGCCAAACCTAACTCTGAACTGTGTTGCAAGCCATTGTGCCTTATGCTGGCAGATGAGTCTGACCACGA
GACCCTGACTGCCATCCTGAGTCCTCTCATTGCTGAGAGGGAGGCCATGAAGAGCAGTGAATTAATGCTTGAG
ATGGGAGGCATCCTCCGGACTTTCAAGTTCATCTTCAGGGGCACTGGATATGATGAAAAACTTGTGCGGGAAG
TGGAGGGCCTTGAGGCTTCTGGTTCAGTCTACATCTGTACTCTTTGTGATGCCACCCGTCTGGACGCCTCTCA
AAATCTTGTCTTCCACTCTATAACCAGAAGCCATGCCGAGAACCTGGAACGTTATGAGGTCTGGCGTTCCAAC
CCTTACCACGAGTCTGTGGAAGAACTACGGGATCGGGTGAAAGGGGTCTCAGCCAAACCTTTCATTGAGACAG
TCCCTTCCATAGATGCACTCCACTGTGACATTGGCAATGCGGCTGAGTTCTATAAGATCTTCCAGCTAGAGAT
AGGGGAGGTGTATAAGAATCCCAATGCTTCCAAAGAGGAAAGGAAAAGGTGGCAGGCCACACTGGACAAGCAT
CTCCGGAAGAAGATGAACCTCAAACCAATCATGAGGATGAATGGCAACTTTGCCAGGAAGCTCATGACTAAAG
AGACTGTAGATGCAGTTTGTGAGTTAATTCCTTCTGAGGAGAGGCACGAGGCTCTGAGGGAGCTGATGGATCT
TTACCTGAAGATGAAACCAGTATGGCGGTCATCATGCCCTGCTAAAGA--GTGCCC-
AGAATCCCTCTGCCAGTACAGTTTCAATTCACAGCGTTTTGCTGAGCTCCTTTCTACCAAGTTCAAGTATAGG
TATGAGGGGAAAATCACCAATTATTTTCACAAAATAGATGTTGCTTATGGCACCCGACAGTTGTCATTATGTT
TGGAAACACTGCCGGCCCATGGAGATGAGGATAAAGTGGACATTTCTCTGCTGCATCAGGGTGAGAATCTTTT
CGAACTGCACATCCACCAGGCCTTCCTGACATCTGCCGCCCTGGCTCAGGCTGGAGACATGCAACCTACCACT
TTCTGCACCTATTCCTTCTATGACTTTGAAACCCATTGTACCCCATTGTCTGTGGGGCCACAGCCCCTCTATG
ACTTCACCTCCCAGTATGTGGTGGAGACAGACTCTCTTTTCATACACTACCTTCAAGGGGCTTCAGCCCGGCT
TGATCTCCACCAGGCCGTGGCCAGTGAGCACAACACTCTTGCTGCAGGATGGATTTACTTTGACAGGGTGCTA
GAGACTGTGGAGAAAGTCCATGGCTTGGCCACACTGATTGGTAAGTGTTGTCGGCTTCCTGCAGCTCCTA-
GCACCAATGCAGAATTTCCCAAACTCTATAGCTGCTTTTCTGGTCRTTTCTTATTTCTTCCTTTTTCTTTGT
TCTTGATCATTGCCACACCGTCTGTATTCTCCATGATTTCA-
TACTTGCTGCTGCCCAGGAGCTGGTGGAGAAGAGTTTGGGGTTCTAGAGTACTGGATGAGGCTGCGTTTCCCC

ATAAAACCCAGCCTACAGGCATGCAATAAACGAAAGAAAGCCCAGGTA?????????????????????????
???????????????????????????TTCATGGAGGGAAAACACCAAACAATGAGCTTTCAGATAAGATTTA
TGTCATGTCTGTTGTTTCCAAGAACAACAAGAAGGTTACTTTTCGCTGCACGGAGAAAGACTTGGTAGGAGAT
GTTCCTGAAGCCAGATATGGTCATTCCATTAATGTGGTATATAGCCGAGGGAAAAGTATGGGTGTTCTTTTTG
GAGGACGCTCATACATGCCTTTTACCCACAGAACCACAGAAAAATGGAATAGTGTAGCTGACTGCCTGCCCCA
TGTTTTCCTGGTGGATTTTGAATTCGGGTGTGCTACATCATACATTCTTCCAGAACTTCAGGATGGGCTATCT
TTTCATGTCTCTATTGCCAAAAATAACACCATCTATATTTAGGAGGGCATTCACTTGCCAATAATATCCGCC
CTGCCAACCTGTACAGGATAAGGGTTGATCTTCCCCTGGGTAGCCCAGCTGT-
GAATTGCACAGTCTTGCCAGGAGGAATCTCTGTCTCCAGTGCA??????????????????????????????
???
??CGTGTCTCAGCATCTTCCTGCTC
TCCACCACACTGAGCCCAAGATGACCCAGTGCTCCACAAACATATGTAGAATGGAAGTAAAGCCTCAGGGATA
AGAAAAGATAATTAAAGGGCCAGGCGTGGTGGTTCATGCCTGTAATCCCAGCACT-
TAGGAGGCCAAGGTGAGTGGATCACAAGGTCAGGAGTTCAAGACCAGCCTGGCCAAGATGGTGAAAACCCATC
TCTACTAAAAATG---CAACAATT-----
GGCTGGG--
---------------------TGTGGTGCTGGGCGCCTGTAATCCCAATTACTTGGGAGGCTGAAGCAA-
AGAATTGCTTGAACCTGGGAGGCAGAGGTTGCAGCGAGCAGAGATCATGCCACTGCACTCCAGCCTGGACAAC
A-GAATAAGACTCCATCTCAA--

---AAAA-TAATAAGAAGAA---
AAGATAGTTAAAGCAGATTCACTATAGTAACAAACCAGCCAAGAAATTGCACTGAAAGTAAAGGTTTTAGGTG
ACAAAATTTCCAACTGTGTTCAAAGAAACATAAGTTCCATCAGGAGTGCACTTAATCTGATTTT-
CTTCAAAGC--
CTCTCTAATTTGCAGGGAAAGACAACCATTTTCATTAGCTTCATGTGAGCTAGAGAGAATCCATGACTAAAGT
GCTAAAACCCCTGGTAAACATGCCAGCAAACAAGGGTACCTTCCCCAGATGCTCAACCTA-----
TAAGACTGTCAGCATTGGTAAGGAGCCTTGAACTCTAAACCCATAGGCTCAAGTCAGGAA-
CTCAGGAAGCCTATCAACACCGAGATACAATTTTGCCACCTTAACACTTTATTCCCTTTACTTGTGCCACACT
???
???
???
???
???
???????????????????????TTAGGAACTCAAGGCCCTCAAAGATCTTTATTGCCATAAAGCAGAGTAAT
TTGTCTTTGACTTGGACCTTGTTTACTGTGAACTCTAATC-AAGGCAGGACATGCAGCAGTTCTGCTTA-
CTGCACTGTGGACATGTGGAAGGGACACTTGTGA---------
ATCCAGTTCGGATTAGTGTTTGAGATGGAATTGCAGATGTTGTTCCCGGATGTGGAATGGAGCCGTGGTCACA
CTTGCCTACA-GAGCTTACTTTGATCTGTTTGTCAGCAGCATGCAAAAAA-
TGCTTTGTTTGCCCTTTGCTTCTGCTTTTTTCAGGGAAGCTGCCAAAGAATGTCGACGTCGAAAGAAAGAATA
TGTAAAATGCCTGGAGAGCCGAGTCGCAGTGCTGGAAGTCCAGAACAAGAAGCTTATAGAGGAACTTGAAACC
TTGAAAGACATTT??
???
???
???
???
???
???
???
???
??TGTTTATATCTAAATGTGTGGATTCCA
GCACCTAAACCAAAAAATTCCACTGTAATGATATGG-
ATTTATGGTGGTGGTTTTCAAACTGGAACATCATCCTTACATGTTTATGATGGCAAGTTTCTTGCTCGGGTCG
AAAGAGTTATTGTAGTGTCAATGAACTACAGGGTGGGTGCTCTAGGATTCTTAGCTTTGCCAGGAAATCCTGA
GGCTCCAGGAAACATGGGTTTATTTGATCAACGGTTGGCTCTTCAGTGGGTTCAAAAAAATATAGCAGCCTTT

GGTGGAAATCCTAAAAGTGTAACTCTTTTTGGAGAAAGTGCAGGAGCAGCTTCAGTTAGTTTGCATTTGCTTT
CTCCTGGAAGCCATTCCTTGTTCACCAGAGCCATTCTGCAAAGTGGATCGTCTAATGCTCCTTGGGCAGTAAC
ATCTCTTTATGAAGCTAGGAACAGAACATTGGCCTTAGCTAAATTTACTGGTTGTTTTAGAGAAAATGAGACT
GAAATAGTCAAATGCCTTCAAAACAAAGATCCCCAAGAAATTCTTCTGAATGAAGCATTTGTTGTCCCCTACG
GAACTCTCTTGTCAGTAAACTTTGGTCCAACAGTGGATGGTGATTTTCTCACTGACATGCCAGACATATTACT
TGAACTTGGACAATTAAAAAAAAACCCAGATTTTGGTGGGTGTTAATAAAGATGAAGGGACAGCTTTTTTAGTC
TATGGTGCTCCTGGCTTCAGCAAAGATAATAATAGTATCATAACTAGAAAAGAATTTCAGGAAGGTTTAAAAA
TATTTTTTCCAGGAGTGAGTGAGTTTGGAAAGGAATCCATCCTTTTTCATTACACAGACTGGGTAGATGATCA
GAGACCTGAAAACTACCGTGAGGCCTTGGATGACGTTGTCGGGGATTACAACATTATATGCCCAGCCTTGGAG
TTTACAAAGAAGTTCTCAGAATGGGGAAACAATGCCTTTTTCTACTATTTTGAACACCGTTGTGCTAATTGTG
AGAAAATATGCTACTTTGATCAAACTAGAATGAAGGAAATAAAAAGACACTGTGGCTCAACTTGTAATAAAGA
GCAGCTCATCCTCACAGCTTTTTGATTGTTGCTATAGTTATCT---
GTTCAACGTGGGATGCACAATACAGTTAA----------
CCAGTAGCCTGTTGCGTGCTTATTGTTAAAAGGAATTTAACATACAAGTTTTTGTATGGATTGTTCGCCTAAT
TCCGATTCCTCGTGCTGACTGGCATGAGACAGGGAGCTGGATTGTATTAAACTGGTTTTATTGTGATGTGACT
ATGATGGATGTGACAAGGACCTCTATTATTCACTGGGGAGTGTGGCCGTTAATGAAGCCATTAGCTGAGCTAA
GGCAAGTGAAGAAAATAGGGAAATTACCATTATTCTGAAGCCATTAAGTTGTAATCCACCACTTTCTCTCCTT
ATTCAAAAGGCATCTTCTTTTTAAGGCCTCCCT??
??TTGGCCTATCCAGGTGTGCGGACCCATGGGA
CTCTGGAGAGTGTGAATGGGCCCAAGGCAGGTTCAAGAAGCCTGACATCGTCATTAGCTGACACTTTCGAACA
TGTGATAGAAGAGCTGTTGGATGAGGACCAGAAAGTTCGACCCCATGAAGAAAACAATAAGGACGCAGACTTG
TACACGTCCAGGGTGATGCTCAGTAGTCAAGTGCCTTTGGAGCCTCCTCTTCTCTTTCTGCTGGAGGAATACA
AAAATTACCTGGATGCTGCAAACATGTCCATGAGGGTCCGGCGCCACTCTGACCCTGCCCGCCGAGGGGAGCT
GAGCGTGTGTGACAGTATTAGTGAGTGGGTAACGGCGGCAGACAAAAAGACTGCAGTGGACATGTCGGGCGGG
ACGGTCACAGTCCTTGAAAAGGTG???
???
?????????????????TTCTTTGAGCAGATGCAGAACTAGACCCCC-
GCCACAGCAGCCTCTGAAGTTGGACAGCAAAACCATTGCTTCACTACCCATCAGTGTCCATTTATAGAATAAT
GTCGAAAGAAACAAACCCCTCTGTCTTATGATTTACTCATTACCGCCTTTTGACAGCTGTGCTGTAACACAAG
TAGATGCCTGAACTTGAATTAATCCACAGATCAGTAATGTATTTTCTCTCTCTCTCT----TT----------
--------ACATTTGGTCTCTACACTACATTATTAATGGGTTTTGTGTACTGTAAAGAATTTAGCTGTTTC-
AAACCTAGTGCATGA-
ATAGATTCTCTCCTGATTATTTATCGCATAGCCCCTTAGCCAGTTGTATATTATTCTTGTGGTTTGTGACCCA
ACTAAGTCGTACTTTAAATATGCTTTAA-GAATCGGTGGGGGA---
TGCTTCACGTGAACGTGGGAGTGTAGCTGCTTCTCTTGCCTCAGTATTCCCTTCCT-
GATCACTATGCATTTTAAAGTTAAA-CATTTTTAAGTATTTCAGATGCTTTAGAGA-ATTTTTTTTC--
CATGATTGCATTTTACTGTACAGATTGCTGCTTCTGCTATATTTGTGATGTAGGAATTAAGAGGAT???????
???????????????????????????????????CTTCCGGAGAGCACTAAGCCACTTCCAGCCCTGCTGTG
AACCAGGACCGTAGGAGCCATCTTTTTCATGTGGTCAGCTTTGGAAGAATCTACATCCACCACTTTAGAAGAC
AAGTCTAGTGGCTTATCTGTGACGTCTTTGGTAACAGTCTGCTTCTCCAACAGAGGAGGTGAGCTGCCGTCTT
TTCTGTCTTGAACCGCTGTCTTCCGGGAATGCCCGGGCACTGGCTGGGCACCTTCGCCCCCTTCTGGAGCCTT
GGGATACTTGCCGTTGGAGAGCCTGGCCGCGGGGAACTCGCTGCTGACTGTCATGTATGGCTTTGACAGGGCA
ACTGAAGGAGAGGTGGAGATCCTGGCATAGTGCTTGTGGAACTCCGAGTAGGTGTCTGTAGCAGGCTGGGTCG
GAAGGTGGACCCGGGGTGAGGGCCGAGGCGAGGGGGGCAACAGGAGAGCTGTGTCCCCCGGCAGGCCACTGGT
GACTGCCTTGGCAGAGGGAACCCTGGGCTGTTTACTGTTCTGGATGTGAGGATAGGCATGGGAATCAACAGGA
TTCCCAGGACTGACCCCCATCTTCCATGGGAGGCTTTTGTCTGCGCAATGGACAAGAGGTGGGATGGCTGGGG
AGGCCGAAGGCGTCGAGAGCCTCATTGGTGATGCCAAGGACGACGGGATGTGGGGACTGACATAGTGAGGTGG
TGGCAGGTAGAGAAAGCGCTCCCCGTTGGTGCAGACTGGAG????????????????????????????????
?????????????TGCCAGGACCTTTGTAATTCAAGTGCTTTTTACAGTAGTAGCAAATGTTACATAAGCCCA
AACATGGAGCA---
TATTTGGAGTTGGTGGGCAGACCCAAATCATGATGCAAGCCTGCAGTAGCAAGTGTGCAATGAAGCACAACCA
AATTCTGTGAATGGAAGGTAGCGAGATGCCTCTCCAAAGCAGCCCTTCTGCGCAGGAGCTGAAAGCTCCACCG
GGTAAGTTTTCTGAGGCAGTTT-
CAGAGGCTGGGCTTGGCTTAGGGCCCAGCTCTGGATGAGCCTGTGGACGGTGGGGT----

AGGTCTTTGCTAACCTTACATAGAACCAAGGAGAGTTTTGAAGAGTTTGTGCAGGCCTCTTTCCTCCTGGTGT
TTGTCTTGCCCTGCCAGCAGGCCAGGAGGG-
CTGCGCTGCTCAAGTAGTGGAGGGAGGGAGCCTGCAGAGCTAGGGGATCAGAGTGGGTAATTGCAGCCACAGG
GATTAGCTGCTGCAAGCTGTCCACAGGAGCCATTCTGAAATGTCATTTGAGTGATCTGGCCGTCTACTGGAGG
AGGCCCTCGGGGCAGCTGTCTCCAGGAGCAGCTTCTGAGGGAGTCTTCCAGGAGGAGCAGTGTCTCTGCCATC
ATCCTCGTCCACATCCTGGTCACATTTCCATTCCTCATCTTCATTCAAAGTCGGCAGGTATCCAGATATGCCC
TTCCCCGTCCCTGACCCAGAGCTCTGGTCGCTACAGACCTTCGGGCTCTCCGAGCCTGTCCTCGTATATTCCA
AATAAATATCAGACTTAAGGAAGGAGGGGTAGGTGTTTTCCTCCATGGTGGCCTGGATTTCTGTCTGGGCCTG
GTCAAACATGGCAGGATCGATCAGCTGCTTCATGATGCAGCCCTTTATGAAGCTCTTGGTGGCCGGCTTGGTC
TGCCGGGACACGATGCCATTGTTATCAAGAATGTACTTTCGGTAGATGGCTCTGGCCAGCTTCAGCCTCTTCT
CCTCGTTCGAGTCACAGGGCTCCAGCTTCCTGAAGCCAGTGCAGGCAAACCAGAAGTCCAGCAAGTCAGCACA
GCCTTCCTGCTTCAGGAAAGTCCTGAACAGGCTTATCCCATCTTGATCATCCAGCAGGGAGTGCAGTGACTCA
GCCCACTTCAAGTACGGTGGGGTGGGGGAGGCACTGCCCTCAGGCTCATACCCCAGGTCCAGATCCGAGCGCC
TCGGAGTGGCGGTCGAAGTCTCACCTTTAATGCCAACACCTTTCCCGGAGCAGAAACTGTAGCTGGCAGGCCT
CAGGTCTGTGGACACCAGTTCTCCCTCCTCACCAGGCACTGGGGGTCGGGGAGCATCTTCGGTGAAACTTGCT
CCAAGGTCCAAGGGGAAACCCTGCTCCTGGACATTCATTTTGGGACTCTGTGCGTCAATGCACAACGAGCGCT
GCACCCTAATACATCAGTACTTAACAGCTCCAAAGTCAATCAATCTGTCCTGTTGAAACCAT??????TAAAA
TGTTAGCTAATTTTAAGTGAACAA-A--
ATTACATGGATCGACTGATGGCAGCATCAAACAAGCATAATTTACAGTGCAGCAGGATAAGT-
ACTTAGGTTATTTATAGGACAGATTATTTTTCTGTGTTGTTTTG----CGTCTTTG-
CATATTAAAATTTATTTATAGGCATAGTTAAGCCTATAACTATTAGTTAGCATAAATTTGGCTTGGAGTCA--
--
CCAAAGCAGAAATGTTGGACTTCATTTTTATGAGTTATTTCCTACACATTTCTAAACTGATGTAGCTTTGTCA
CTGCTTGATTGATTTTTTTT---
AAACTGGAGGGGGTAAAATTAGTTTATACTGAATCATACCAGTTTCTCTTAATTAGTA---------------
----CATTACTAACATTAAAAAGAAGTAAAAGGAATGTTAATAACTTAGGAGTATTTGCATAGTTA-
CGTTCAGAACACAAGCTTTAAATTAGT-GAGGCAACTA---------
GAAGTTTGATTTAAGCACTCATACTCCTTTATTTTCCTTTGTTAGCAATAATTATTGGCCCTGATGGCCATCC
TTTGACTGTTTATCCTTGTATGATTTGTGGGAAGAAGTTTAAGTCGAGAGGTTTTTTGAAAAGACACATGAAA
AACCATCCCGAACATCTTGCCAAGAAGAAGTACCACTGTACTGACTGTGATTACACTACCAACAAGAAGATAA
GTTTACACAACCACCTGGAAAGCCACAAACTGACCAGTAAGGCAGAGAAGACCATTGAATGTGATGAGTGTGG
GAAGCATTTTTCTCATGCAGGGGCTTTGTTTACTCACAAAATGGTGCACAAAGAAAAGGGAGCCAACAAAATG
CACAAGTGTAAATTCTGTGAATACGAGACAGCTGAACAAGG?????????????????????????????????
????????????????????CCCGCTATAGCTTCTGCAAACAGTAAAGATACCACTAAAACCCCTTCTGCAGT
TCAAACTAGCACCAGCCACAACCCTGGACTTCCTCCTAATTTTAACGAATGGTACGTCTGAGGAAAAACACAA
ACCCTGTTAACTATAGAATGGACCAAATGCATTTTTAAAAGAAAACTGAGACCAATCAGATGGAAATGGAGTT
TTAAGGCAAGAGGCCATATATAGGGCTACATCTTGTTAATTGCAATTGTCCAGGAAGGTTTCGGGCAAGATCC
AAAACTAGCCATGCCCTTTTCTCAGGATTAGAAAATATGTTTTGGCGTTTGA-GGATTTTTT--
ACAAAATCTTTACACTGCTTTTTCTTCCCCTTCCTCTTGCTCTCTGCACACCCCATTCCTAAACTCTTGCAAT
TCAATTTAACACTTGTCCTGTTTCTTGAGAGGAAGTTATAGAAGGCTTG---
GTGGTGGTGATGTTAAACTGATGGAAATTCTTTTTCGCCTTAGTGGTGATTGTTTAAACT?????????????
??????????????????????
Cebus
?TACCCCCATGTTTGGCTGGAACATGAAGCTGACCTCAGAGTACTACAGAAATGTCACCTTCCTTTCATGCCG
GTTCCCTT-
CCGTCATGAGCATGGACTACATGGTATACTTCAGCTTCTTCACCTGGATTTTCATTCCCCTGGTCATTATGTG
TGC--AATCTATGTTGACATCTTTTACAK-
CATCCGGAACAAACTCAGACAGAACTTCGCTAACTCCAAAGAGACAGGTGCATT---
TTATGGACGGGAGTTCAAGACGGCGAAGTCCCTGTTTCTGGTGCTTTTCTTGTTTGCTTTGTCATGGCTGCCT
TTGTCCATCATCAACTGCATCACCTACTTTCA--------------
TGGTGAGGTACCACAGCTTGTGCTGTACTTGGGCATCCTGCTGTCCCATGCCAACTCCATGATGAACCCTATC
?????????????????????ATGTTCATTAACCGATGATTGTTCTCCACTAATCACAAGGATATTGGTACTT
TATACTTACTATTTGGAGCATGAGCCGGTATAGTAGGCACTGCCTTGAGCCTCCTCATCCGAGCCGAACTAGG
TCAGCCCGGTACTTTACTAGGTGACGATCAAATTTATAATGTCATCGTAACCGCCCATGCTTTCGTAATAATC

TTCTTCATAGT---CATGCCCATCATAATTGGGGGCTTTGG---
AAACTGACTAGTGCCGTTAATAATTGGTGCTCCGGACATGGCATTCCCCCGAATAAATAACATGAGCTTCTGA
CTCCTTCCTCCA--TCCTTTCTTCTACTATTAG-
CATCTTCTATGGTAGAAGCAGGTGCAGGAACGGGATGAACCGTATACCCCCCACTGGCTGGCAATCTGGCCCA
TGCAGGAGCATCCGTTGACCTTACAATTTTCTCCTTACACTTAGCCGGAGTCTCTTCTATTTTAGGGGCAATT
AATTTCATCACTACTATTATCAACATAAAACCCCCTGCAATATCCCAGTATCAAACTCCCCTGTTTGTATGAT
CAGTACTAATTACAGCAGTTCTACTCTTACTATCCCTGCCTGTACTGGCTGCTGGAATTACAATACTTTTAAC
AGACC--
GGAATCTTAATACCACCATTTTTGATCCCGCTGGGGGAGGAGACCCTATCCTATATCAACACTTATTCTGATT
TTTCGGACACTTTGAAGTTTACATTCTTATTCTGCCCGGATTCCGAATATTTCTTCCAATTGTCCTTACCTAC
TCCAACAAAAAGAAGCCTTTCGGTTATATAGGATTTGTATGACCAATAAATTCCATGGGCTTCCTAGGCTTAA
TCGTATGAGCTCACCATATATT-
CACAGTAGGAATAGATCTGGATACACGCGCGTATTTTACATCAGCTACCATAATCATTGCCATCCCCACTGGG
GTCAAAGTATTTAGCTGGTTGGCTACACTACATGGAGGCAACATCAAGTGATCTCCTGCAATACTATGAGCCC
TGGGTTTTATTTTTCTCTTCACTGTGGGTGGATTGACAGGAGTTGTATTAGCTAACTCATCATTAGATAT-
TGTCCTACATGATACATACTACGTGGTAGCCCATTTCCATTACGTCTTATCAATAGGAGCAG-
TGTATGCCATTATAGGAGGCTTCCTTCACTGATTTCCATTATTTTCAGGCTATACACTCGACCAAACTTACGC
TAAAATCGACTT-
TACTATTATATTTGTAGGTGTAAACATAACCTTCTTTCCACAACACTTTCTCGGCCTATCTGGAATACCTCGA
CGGTACGAGGATTATCCAGATGCCTACACTGCATGAAATATTATCTCATCCGTAGGCTCATTTATTTCACTAA
CAGCAGTTATTCTAATAATTTTTATAATTTGAGAAGCCTTTTCTTCAAAACGAAAAGTTCGAACTATTGAACA
AATGATTACTAATCTAGAGTGATTGTATGGCTGTCCCCCTCCCTACCACACATTTGAAGAAGCTACCTACGTA
AAACTCCTAAACGAAAAAGGAAGG???????????GTGGCAACACCAGCTCAACTAGGCCTACAAAACGCTA
CATCCCCCATTATAGAAGAACTAATTGCCTTCCACGACCACACGCTTATAATTATTTTCCTAATTAGTTCATT
AGTCCTATACATTATCTCTTTAATGCTTACTACAAAACTAACCCACACCAGCACTATAAATGCCCAAGAAATC
GAAATAATCTGAACTATTCTACCCGCAATTATTCTTATTATAATTGCCCTTCCATCCCTGCGCATTTTATATA
TAACAGATGAATTCAATAAACCCTACTTAACCCTAAAAGCAATTGGCCACCAATGATATTGAAGCTATGAATA
CTCGGACTATGAAGACCTATTCTTTGACTCTTACATTATACCAACCTACTATCTCCAACCAGGCGAATTCCGA
CTTCTTGAAGTAGATAATCGAACAACCCTACCAATAGAAGCAGACATCCGTATACTAATCTCATCACAAGATG
TACTACACTCATGAGCCGTCCCATCATTAGGCGTTAAAGCAGATGCAATCCCAGGCCGATTAAACCAAGCTAT
ACTAGCCTCAATACGACCAGGACTATTTTATGGGCAATGCTCGGAAATTTGCGGGTCAAACCATAGCTTTATA
CCCATTGTTCTAGAGTTTATCTACTTCCAAGATTTTGAAGTATGGGCTTCATACTTATACATTGTAT??????
ATGACCTCTTCCCGCAAAACACATCCACTAATAAAAATTATTAATAACTCATTTATTGACCTCCCTACACCAT
CCAACATCTCCTCCTGATGAAACTTCGGATCACTTCTAGGCGCCTGCCTAATAATTCAAATCACCACAGGCCT
ATTCTTAGCAATACACTACACGCCAGACACCTCAACCGCCTTCTCCTCAGTAGCTCACATCACCCGAGATATC
AACTATGGCTGAATAATCCGCCTCCTACACGCCAATGGTGCCTCCGTATTTTTTGCATGCTTATTCCTCCACA
TCGGCCGAGGCCTCTACTACGGATCCTTTCTCCTTCTAAAGACCTGAAACATCGGTACAATCCTACTATTAAT
AACAATAGCCACAGCCTTTTTTGGCTACGCTTCACCATGTGGCCAAATTTCATTCTGAGGAGCGACAGTTATT
ACAAATCTTCTATCAGCCATCCCTTATACCGCACATAACCTTGTACAATGAATCTGAGGTGGCTTTTCAGTAG
ATAAACCCACCCTCACACGATTCTTTACCTTTCACTTTATTTACCTTTTATTATCTCAGCCCTTCCAACTAT
TCACCTTTTATTTCTACATGAAACAGGCTCAAATAATCCATCAGGAATAGCATCTAGCCCCGATAAAATTATA
TTCCATCCCTACTACACAACCAAAGATATTTTTGGATTAACCCTTCTTCTCCTACTCCTTACAAGCCTAACCC
TATTTACCCCCGACCTTTTATCTGACCCAGATAACTACACACTAGCGAACCCCCTTAATACTCCACCCCATAT
TATGCCAGAGTGATACTTTCTATTCGCATACACAATTTTACGATCTATTCCAAATAAACTAGGAGGTGTTCTA
GCTCTTCTATTATCTATTATAATCCTAACAATTATCCCTGCCACTCACCTATCCAAACATGAAAGTATAATAT
TCCGTCCAATCACCCAAATCCTATTCTGAACCCTAGCAGCCGATCTACTTACACTTACATGAATTGGAGGCCA
ACCAGTATGGTTTTCCTTTGAAGTCATCGGCCAAACCGCATCCATTACTTACTTCCTTATTATTAC---
CCTAATTCCTCTATCAGCCCAAACTGAAAATAAACTACTTAAATGGTAA?TTAGGTTAAATTTATTCAAGGAA
ATGAGAATTTATGGGTATAACTGTTAGCTGGAAAGCCCTTTCTCACCTCCAATCCTCCTTTCAAACTACATTT
CC-
TCCTGAAATTTCTCTGTGGGATGAAATGTTCAATTATTAGTCAATCAATCAATCTCTTATACATTATCTGGCT
TTACATAT----
GCCCTTGATAGTTCTTGGAAAGTAATCCAATATTGTGTTGTTTTGGTGTCTTTGTATTGGAAATGGAAGCATT
TTGTGTGAATCACCTCTTTAACTGTGAGGAGTTGAATTGTCTTAGTTCTGCTTGCCTAATAACCGGGAATAAA

--GACATCACAGTCTCTAACCCCACTTTTT-
GGTGGAAAGAAAATCTAAAAGTAGCAACTTTTAATGGAACAAAATATCAATAATCAAAGAAACTCCCCTGTGA
AACTGTAATATTATTTT-
AAAATCAACATTGTTCTAAGGTAGCTGAAATGTGTGCTTTTTCTATTTTGCCTTTTCGCTGACAATATACCCA
TAAATCTCTGGTGGTGATTTTCTCTCCAAAGTTAATTTCTGGGCCATTGGTGACTGCACTATCTATTGTTCTA
AATTGTTGTCTTAATTGCATTCTTTAAAAATGTAACGTTGAAGGCACATAATATTGAAGATGACTCCAGGATG
AACCCTGAGTTCGCAGACCGAA-
TAAAACAGCTCGATAAAGAGGCGTCTTACTACCGTGATGAGTGTGGCAAGGCCCAAGCGGAAGTGGACCGGTT
GCTGGAGA?????????GGAAGAGCTGCATCAGGCAAGTACTTTGATCTTTCTTGGAGCAGGTTCCTTCAAAA
CGCTTCTCCAGAGGACGGAAGTCCCTCCAGTCTGGCTCGCTGCTTGCAGTGGTGGGGAAAGCTGAGGTAATTC
CCCGGTGGGAAATCTGCGAGGCCCCTGATACCCCTG--------
CCACCAGGCCCATCACTGATGGGCCTAAGGGCCTCATCTGATACTCTAAGATGGGCTTCTCCTGTTGGATCTG
GGCCTCTCGGACTTGAGCCTCTCTAGAACGAGCCTCTCGGGCCTGGGCTTCTCGGGTTCTGGCCTCCCTGGCA
TGACCTTCCCAAGTGTGGACCTCCCTG--
GCATAGGCTTCCTTGCCATGAGCCTCTCGAGTATAGGCCTCCCTGGTGTGGGCCTCCCTAGCCTGAGCTTCTC
GGGCACGTGCTTCCCGGGCCTCAAGCTGCTCCCGCCGAAGCTCCCAATACAACAACTGTTTCTGGATGGTCA-
CTAGCCGTTCCTCCTCTGTCTCCATTGCCCCAGGTGGCCGGGAGGAAGAAAAGGGCTCAAAGTTCAAGAAGGG
GTCAAACATCTCAGAGCTTCGACCATGGAGGTCATAAAGGCAGTCATCCCCAGGTGGAGAGTTCTCAAGACTG
TCATCTGGCTCATAGAACTCATATAGGGCATCTCCACTGTAGCTGTCTCGGGGCAGACAATCCCTGCGGACAA
GACCCAGGG??TTGATGAACTAAAAATGGTTCCTG
AAGGTCAGATTCATGTACAGTGAATGTTCTCCACTGTTGTGGTGGGCATGTTTCATGTGTGGGGATGTTGGTG
TGCAAGTTTCGTGTGTTTGGAAAACACTAGCTAGAACATTCGAAAAATTTCATTAACCCAGCTCTGGAAGGTC
ATCTATGAATTCTTCCTATTCCAGTGGCCTTTT-
AAGTGTAGATGTAGATACACTGTAATTTAAAACAGATGGGCATGTCTGAATATTTTCATATGCAAAATGGGAC
AGATGTATATGAATGGATAAATTATCCTTTTAAAAAGATACTTTGTAGCACGAAATATCACCAATGATTGGAA
CAGTGAACCTGCATTCAGTCTAGGGCAAACACCAGGAAGACATTATCAAAACATCTAA-
CTATCTACAAACTAATTCCAATTAGTCTACTTTGTCTTCATACAGAAAAAA-CTTTTTTGATTTTTAAA---
TATGTATTGGGTGCCAATCTGTATCCTTTCGAGCTGCTAAAGCCAGCATATCACAATAGAAGTTTACTTTTCC
?????????????ATGTTTACAATTAACCTACTCCTACTAATTACCCCAGCCCTAATTGCTATAGCATTCTT
AACACTTATAGAACGAAAAATCTTAGGCTATATACAACTCCGCAAAGGCCCCAACACTGTAGGCCCATATGGA
GTACTCCAACCAATCGCCGACGCAATAAAACTCTTTACAAAAGAACCCCTACTACCCAGTGTATCCACTACAA
CCCTATATATAGCCGCCCCAACCCTAGCCCTAACCATTGCCCTTCTCCTATGAACCCCCCTCCCTATACCGTA
CTCCCTTATCAACTTCAATCTAGGTCTTTTATTTGTACTAGCAACATCAAGCCTAGCTGTCTACTCAATTCTA
TGGTCCGGATGAGCATCCAACTCAAATTACGCATTAATTGGCGCACTACGAGCCGTAGCCCAGACCATTTCAT
ACGAAGTCACCCTAGNCATTATTCTATTATCAACACTACTAATAAGCGGCTCCTTTAACCTACACTCACTAAT
TACAACACAAGAACAATCCTGACTTCTACTACCATCATGACCCCTAACCATAATATGATTTATTTCCACACTA
GCAGAAACCAATCGGGCCCCCTTTGATCTAACAGAAGGTGAATCAGAACTAGTCTCAGGCTTTAATATTGAAT
ATGCCGCAGGTTCATTCGCCCTATTTTTCATAGCAGAGTACATAAATATTATTATAATAAACGCTCTAACTAC
CACCATCTTTACAGCAACACCCTACAATATACTCATAACAGAACTTTACACTATAAACTTCATAACTAAAACA
CTTCTACTAACTATCCTATTTTTATGAATTCGCACAGCATACCCTCGTTTTCGCTACGACCAACTAATGTATC
TCCTATGAAAAAAATTTCTACCACTTACACTAGCACTATGCATATGATATATTTCAATGCCTATACTACTATC
CGGTATCCCACCCCAAACATA?ATTAACCCCTTAGCCCACCTAATTATCTCCCTGACTATTCTAATAGGAACT
ATAGTCACAACTCTAAGCTCACACTGATTCCTAGTATGGGTAGGCTTAGAACTAAATATATTAGCCATCGTAC
CCATACTCGCTAAAAACATAAGCCCTCGCTCCACAGAAGCATCCACTAAATATTTCCTAACACAAGCATCCGC
ATCCATAATCCTTCTAATAACTATTTTCCTCAATAACTTACTCTACGGACAATGAACAATTAATCCACCATCT
AATCAAATCTTATCTACAATAATACTAATTGCCCTCGTACTAAAACTAGGAATAGCACCCCTTCACTTCTGAC
TACCAGAAGTAACCCAAGGCATTCCCCTAATTCCTACTATACTTATCCTTACATGACAAAAACTAGCCCCCAT
ATCAATTATCATTCAAATTTTCCCATCCATCAACTCAAACATCCTACTAATAATCTCACTTATATCTATTATA
GCTGGCAGCTGAGGAGGACTGAACCAAACACAACTACGCAAAATTCTAGCCTACTCCTCAATTACCCACATAG
GATGAATAATAGCTGTACTATACTATAACCCAAACACTACCATCTTAAGTTTACTTATCTATATCCTCCTAAC
AATTTCCACACTTACAACCTTCTACTTAAACTCAAACACAACAACCCTATCACTATCCCATACTTGAAATAAA
CTCACATGGATAATACCAATAATTCCAATAATAATAATATCCCTAGGAGGCCTCCCCCCACTAACAGGCTTCT
CCCCCAAATGAGCTATCATACAAGAAATTACAAAAAATAACAGCCTCATTTTCCCCCTTACCATAACTATACT
CACACTAATAAACCTATATTTCTACATACGCCTAACATACTCAATTTCAATAACAATATTTCCCACATCCAAC

AACACAAAAATTAGCTGACAACTAAAATATATAAAACCAACACCACTTCTATCCCCTCTAATAATTTCTTCTA
CCTTTTTACTACCTGTAACACCACTAATACTAATAATCT??????TTGGCAGCATAAATAATAAAGCATGTTA
CTTAAGCAATTTTACATTTGCTGTATTAGTAAGTAAATGCAATGGCCACTCTTATTCTCAAACAATAAAAACT
ACCCAGCCTTTTCATGAAAGAT-GATTGCAATTTCCAWTGGCCTGTATATGCAATAAAAAG---
CCATAACTCATCAAAATTTTAGCTATTTTCTCTTAAGATA--
GAAGAGCGTTTTGCTTGAACATTGAACGTGACATTAAATATT-
CATGAAACTGCATAGAGTTTAAGTACTAAG---
TAAAACTCTCAAATTCTTTTCAACCAGATTGAAATTCTGTTTGAACCAGGTAAAAACTACCGYTAACCTGACG
AGGTGTTCAAAAAC-
ACCGTAAAGAGATTGTACAATACGTTCAAAGTGCTAGAGTTATTTGGGATAGATTTTTCTGAAAAGCCTGTTC
CTCCTTTGAAATTGATTCATGATAACAGCCTCCATTTGGGTCAAACAGTATTTAACCGCTTGTAAA-
TTTTAATT-
AAATGATTTGTCACTTTGAACACATAGTTTGTCCCCAAGACCTTGAGGATTACTTGAATGGCCCCTTCACTGT
GGTTGTGAAGGAGTCTTGTGACGGAATGGGAGATGTGAGTGAGAAGCATGGGAGTGGGCCAGTAGTCCCGGAA
AAGGCAGTCCGTTTTTCATTCACAATCATGAAAATTACTATAGCCCATAACTCTCAGAATGTGAAAGTGTTTG
AAGAAGCCAAACCTAACTCTGAACTGTGTTGCAAGCCATTGTGCCTTATGCTGGCAGATGAGTCTGACCATGA
GACCCTGACTGCCATCCTGAGTCCTCTCATTGCCGAGAGGGAGGCCATGAAAAGCAGTGAATTAATGCTTGAG
ATGGGAGGCATTCTCCGGACTTTCAAGTTCATCTTCAGGGGCACTGGATATGATGAAAAACTTGTGCGGGAAG
TGGAAGGCCTTGAGGCTTCTGGCTCAGTCTACATCTGTACCCTTTGTGATGCCACCCGTCTGGAAGCATCTCA
AAATCTTGTCTTCCACTCTATAACCAGAAGCCATGCCGAGAACCTGGAACGTTATGAGGTCTGGCGTTCCAAC
CCTTATCATGAGTCTGTGGAAGAACTGCGGGATCGGGTGAAAGGGGTCTCAGCCAAACCTTTCATTGAGACAG
TCCCTTCCATAGACGCACTCCACTGTGACATTGGCAATGCGGCTGAGTTCTACAAGATCTTCCAGCTAGAGAT
AGGGGAGGTGTATAAGAATCCCAATGCTTCCAAAGAGGAAAGGAAAAGGTGGCAGGCCACATTGGACAAGCAT
CTCCGGAAGAAGATGAACCTCAAACCAATCATGAGGATGAATGGCAACTTTGCCAGGAAGCTCATGACCAAAG
AGACTGTGGATGCGGTTTGTGAGTTAATTCCTTCCGAGGAGAGGCATGAGGCTCTGAGGGAGCTGATGGATCT
TTACCTGAAGATGAAACCAGTATGGCGGTCATCATGCCCTGCTAAAGA--GTGCCC-
AGAATCCCTGTGCCAGTACAGTTTCAATTCACAGCGTTTTGCTGAGCTCCTTTCTACCAAGTTCAAGTATAGG
TATGAGGGGAAAATCACCAATTATTTTCACAAAA??????????TTATGGCACCCGACAGTTGTCATTATGTT
TGGAAACACTGCCGGCCCATGGAGATGAGGATAAAGTGGACATTTCTCTGCTGCATCAGGGTGAGAATCTTTT
CGAACTGCACATCCACCAGGCCTTCCTGACATCTGCTGCCCTGGCTCAGGCTGGAGACACCCAACCTACCACT
TTCTGCACCTATTCCTTCTATGACTTTGAAACCCATTGTACCCCATTGTCTGTGGGGCCACAGCCCCTCTATG
ACTTCACCTCCCAGTATGTGGTGGAGACAGACTCCCTTTTCTTACACTACCTTCAAGGGGCTTCAGCCCGGCT
TGATCTCCACCAGGCTGTGGCCAGTGAGCACAACACTCTTGCTGCAGGATGGATTTGCTTTGACAGGGTGCTG
GAGACCGTGGAGAAAGTCCATGGCTTGGCCACACTGATTGGTAAGTGCTGTCGGCTTCCTGCAGCTGCTA-
GCACCAATGCAGAATTTCCCAAACTCTATAGCTGCTTTTCTGGTCATTTCTTA--------
CATTTTCTTTGTTCTTGATCATTGCCGCACCATCTGTGTTCTCCATGATTTCA-
TACTTGCTGCTGCCCAGGAGCTGGTGGAGAAGAGTTTGGGGTTCTAGAGTACTGGATGAGGCTGCGTTTCCCG
ATAAAACCCAGCCTACAGGCATGCAATAAACGAAAGA????????????????????????????????????
???????????????????????????TTCATGGAGGGAAAACACCAAACAATGAGCTTTCAGATAAGATTTA
TGTCATGTCTGTTGTTTCCAAGAACAACAAGAAGGTTACTTTTCGCTGCACGGAGAAAGACTTGGTAGGAGAT
GTTCCTGAAGCCAGATATGGTCATTCCATTAATGTGGTATATAGCCGAGGGAAAAGTATGGGTGTTCTTTTTG
GAGGACGCTCATACATGCCTTCTACCCACAGAACCACAGAAAAATGGAATAGTGTAGCTGACTGCCTGCCCCA
TGTTTTCCTGGTGGATTTTGAATTCGGGTGTGCTACATCATACATTCTTCCAGAACTTCAGGATGGGCTGTCT
TTTCATGTCTCTATTGCCAAAAATGACACCATCTATATTTTAGGAGGACATTCACTTGCCAATAATATCCGCC
CTGCCAACCTGTACAGAATAAGGGTTGATCTTCCCCTGGGTAGCCCAGCTGT-
GAATTGCACAGTCTTGCCAGGAGGAATCTCTGTCTCCAGTGCAA?????????????????????????????
???
??TGTGTCTCAGCATCTTCCTGCTC
TCCACCACACTGAGCCCAAGATGACCCAGTGTTCCACAAACATATGTAGAATGGAAGTAAAACCTCAGGGATA
AGAAAAGATAATTAAAGGGCCGGGCGCAGTGGTTCATGCCTATAATCCCAGCACT-
TAGGAGGCCAAGGCGAGTGGATCACAAGGTCAGGAGTTCAAGACCAGCCTGGCCAAGATGGTGAAAACCCGTC
TCTACTAAAAATAAAAATA-----------------------------------CAACAATT-----
AGCTGGG--
---------------------TGTGGTGGTGGGCGCCTGTAATCCCACTTACTTGGGAGGCTGAAGCAG-

AGAATTGCTTAAACCTGGGAGACAGAGGTTGCAGTGAGCCGAGATCACGCCACTGCACTCCAGCCTAGACAAC
A-GAGTCAGACTCCATCTCAA--

AAAAATAATAATAAAAAGAAAAGATAGTTAAAGCAGATTCACTATAGTAATAAACCAGCCAAGAAATTGCACT
GAAAGTAAAGGTTTTGGGTGACAACATTTCCAACTGTGTTCAGAGAAACATAAGTTCCAGCAGGAATGCACTT
AATCTGATTTT-CTTCAAAGC--
CTCTCTAATTTGTAGGGAAGGACATCCATTTTCATTAGCTTCATGTGAGCTAGAGAGAATCCATGATTGAAGT
GCTAGAACCCCTGATAAACATGCCAGCAAGCAAGGGCACCTTCCCCAGAGGCTTAACCTAGAATATAAGACTG
TCAGCATTGGTAAGGAGCCTTGAACTCTAAACCCATATGCTCAAGTCAGGAA-
CTCCGGAAGCCTATCAACACTGGGATACAATTTTGCCACCTTAACACTTTATTCCCTTTACTTGTGCCACACT
???
???
???
???
???
???
???
???
???
???
???
???
???
???
???
???
???
???
???
???
??TGTTTATAT
CTAAATGTGTGGATTCCAGCACCTAAACCAAAAAATGCCACTGTAATGATATGG-
ATTTATGGTGGTGGTTTTCAAACTGGAACATCATCCTTACATGTTTATGATGGCAAGTTTCTTGCTCGGGTCG
AAAGAGTTATTGTAGTGTCAATGAACTACAGGGTGGGTGCTCTAGGATTCTTAGCTTTGCCAGGAAATCCTGA
GGCTCCAGGAAACATGGGTTTATTTGATCAACGGTTGGCTCTTCAGTGGGTTCAAAAAAATATAGCAGCCTTT
GGTGGAAATCCTAAAAGTGTAACTCTTTTTGGAGAAAGTGCAGGAGCGGCTTCAGTTAGTCTGCATTTGCTTT
CTCCTGGAAGCCATTCCTTGTTCACCAGAGCCATTCTGCAAAGTGGATCCTCTAACGCTCCTTGGGCAGTAAC
ATCTCTTCATGAAGCTAGGAACAGAACGTTGGCCTTAGCTAAATTTACTGGTTGCTCTAGAGAAAATGAGACT
GAAATAGTCAAATGCCTTCAAAACAAAGATCCCCAAGAAATTCTTCTGAATGAAGCATTTGTTGTCCCCTACG
GAACTCTCTTGTCAGTAAACTTTGGTCCAACAGTGGATGGTGATTTTCTCGCTGACATGCCAGACATATTACT
TGAACTTGGACAATTTAAAAAAACCCAGATTTTGGTGGGTGTTAATAAAGATGAAGGGACAGCTTTTTTAGTC
TATGGTGCTCCTGGCTTCAGCAAAGATAACAATAGTATCATAACTAGAAAAGAATTTCAGGAAGGTTTAAAAA
TATTTTTTCCAGGAGTGAGTGAGTTTGGAAAGGAATCCATCCTTTTTCATTACACAGACTGGGTAGATGATCA
AAGACCTGAAAACTACCGTGAGGCCTTGGATGATGTTGTTGGGGATTACAATATTATATGCCCGGCCTTGGAG
TTTACGAAGAAGTTCTCAGAATGGGGAAACAATGCCTTTTTCTACTATTTTGAACACCG??????????????
???CTGTGGCTCAACTTGTAATAAAGA
GCAGATCATCCTCACAGCTTTTTGATTGTTGCTATAGTTATCT---
CTTCAACATGGGATGCACAATACAGTTAACGTTTCATTACCAGTAGCCTGTTGCGTGCTTATTGTTAGAAGGA
ATTTAACATACAAGTTTTTGTATGGATTAGTCACCTAATTCCGATTCCTCGTGCTGACTGGCATGAGACAGGG
AGCTGGATTGTATTAAACTAGTTTTATTGTGACGTGACTATGATGGATGTGACAAGGGTCTCTATTATTCGCT
GGGGAGTGTGGCCGTTAATGAAGCCATTAGCTGAGCTAAGGCAAGTGAAGAAAATAGGGAAATTACCATTATT
CTGAAGCCATTAAGTTGTAATCCACCACTTTC???
??AGCAAACGTCCGAGGAC

AAGGTGGCTTGGCCTACCCAGGTGTGCGGACCCACGGGACTCTGGAGAGCGTGAATGGGCCCAAGGCAGGTTC
AAGAGGCCTGACATCGTCGTTGGCTGACACTTTCGAACATGTGATAGAAGAGCTGTTGGATGAGGACCAGAAA
GTTCGGCCCCATGAAGAAAACAATAAGGACGCAGACTTGTACACGTCCAGGGTGATGCTCAGTAGTCAAGTGC
CTTTGGAGCCTCCTCTTCTCTTTCTGCTGGAGGAATACAAAAATTACCTGGATGCTGCAAACATGTCCATGAG
GGTCCGGCGTCACTCTGACCCTGCCCGCCGAGGGGAGCTGAGCGTGTGTGACAGTATTAGTGAGTGGGTAACG
GCGGCAGACAAAAAGACTGCAGTGGACATGTCGGGCGGGACGGTCACAGTCCTTGAAAAGGTCCCTGTATCGA
AAGGCCAACTGAAGCAATACTTCTACGAGACCAAGTGCAATCCCATGGGTTACACAAAAGAAGGCTGCAGGGG
CATAGACAAAAGGCACTGG???ATGCA
GAACTAGACACCC-
GCCACAGCAGCCTCTGAAGTTGGACAGCAAAACCATTGCTTCACTACCCATCGGTGTCCATTTATAGAATAAT
GTGGAAAGAAACAAACCCCTCTGTCTTATGATTTACTCATTATCGCCTTTTGACAGCTGTGCTGTAACACAAG
TAGATGCCTGAACTTGAATTAATCCACAGATCAGTAATGTATTCTCTCTGTCTCT------TT----------
--------ACATTTTGGTCTCTACACTACATTATTAATGGGTTTTGTGTACTGTAAAGAATTTAGCTGTTTC-
AAACCTAGTGCATGA-
ATAGATTCTCTCCTGATTATTTATCACATAGCTCCTTAGCCAGTTGTATATTATTCTTGTGGTTTGTGACCCC
ACTAAGTCCTACTTTAAATATGCTTTAA-GAATCGATGGGGGA---
TGCTTCACGTGAACGTGGGAGTTTAGCTGCTTCTCTTGCCTAAGTATTCCTTTCCT-
GATCACTATGCATTTTAAAGTTAAA-CATTTTTAAGTATTTCAGATGCTTTAGAGAGATTTTTTTTC--
CATGATTGCATTTTACTGTACAGATTGCTGCTTCTGCTATATTTGTGATATAGGAATTAAGAGGATACACGTT
TGTTCCTTCGTGCCTGTTTTATGTGCATAATCTCACTTCCGGAGAGCACTAAGCCACTTCCAGCCCTGCTGTG
AACAAGGACCGTAGGAGCCATCTTTTTCATGTGGTCAGCTTTGGAAGCATCTACATCCACCACTTTAGAAGAC
AAGTCTAGCGGCTTATCTGTGACGTCTTTGGTAACGGTCTGCTTCTCCAACAGAGGAGGTGAGCTGCCGTCTT
TTCTGTCTTGAACCGCTGTCTTCCGGGAATGCCCGGGCACTGGCTGGGCACCTTCGCCCCCTTCTGGAGCCTT
GGGATACTTGCCGTTGGAGAGCCTGGCCGCGGGGAACTCGCTGCTGACTGTCATGTATGGCTTTGACAGGGCA
ACTGAAGGAGAGGTGGAGATCCTGGCATAGTGCTTGTGGAACTCCGAGTAGGTGTCTGCAGCAGGCTGGGTGG
GAAGGTGGACCCGGGGTGAGGGCCGAGGCGAGGGGGGCAACAGGAGAGCCGTGTCCCCTGGCAGGCCACTGGT
GACTGCCTTGGCAGAGGGAACCCTGGGCTGTTTACTGTTCTGGATGTGAGGATAGGCATGGGAATCAACAGGA
TTCCCAGGGCTGACCCCCATCTTCCATGGGAGGCCTTTGTCTGCGCAATGGACAAGAGGTGGGATGGCTGGGG
AGGCCGAAGGCGTCGAGAGCCTCATGGGGGATGCCAAGGACGATGGGATGTGGGGACTGACGTAGTGAGGTGG
TGGCAGGTAGAGAAAGCGCTCCCCGTTGGTGCAGACTGGAGAATACAGCGGCTGGGCCAAGCTGTAGGACTGC
TGAGGTAGCAAG?TGCCAGGACCTTTGTARTTCAAGTGCTTTTTACAGTAGTAGCAAATGTTACATAAGCCCA
AACATGGAGCA---
TATTTGGAGTTGGTGGGCAGACCCAAATCATGATGCAAGCCTGCAGTAGCAAGTGTGCAATGAAGCACAACCA
AATTCTGTGAATGGAAGGAAGCGAGACGCCTCTCCAAAGCAGCCCTTCTGCGCAGGAGCTGAAAGCTCCACTG
GGTAAGTTTTCTGAGGCAGTTTTCAGAGGCTGGGCTTGGCTCAGGGCCCAGCTCTGGATGAGCCTGTGGACGG
TGGGGT----
AGGTCTTTGCTAACCTTACATAGAAGCAAGGAGAGTTTGGAAGAGTTTGTGCAGGCCTCTTTCCTCCTGGTGT
TTGTCTTGCCCTGCCAGCAGGCCAGGAGGG-
CTGTGCTGCTCAAGTAGTGGAGGGAGGGAGCCTGCAGAGCTAGGGGATCAGAGTGGGTAATTGCAACCACAGG
GATTAGCTGCTGCAAGCTGTCCACAGGAGCCATTCTGAAATGTCATTTGAGTGATCTGGCCGTCTACTGGAGG
AGGCCCTCGGGGCAGCTGTCTCCAGGAGCAGCTTCTGAGGGAGTCTTCCAGGAGGAGCAGTTTCTCTGCCATC
GTCCTCGTCCACGTCCTGGTCACATTTCCATTCCTCATCTTCGTTTAAAGTCGGCAGGTATCCAGACATGCCC
TTCCCTGTCCCTGACCCAGAGCTCTGGTCACTACAGACCTTCGGGCTCTCCGAGCCTGTCCTCGTATATTCCA
AATAAATATCAGACTTAAGGAAGGAGGGGTAGGTGTTTTCCTCCATAGTGGCCTGGATTTCTGTCTGGGCCTG
GTCAAACATGGCAGGATCGATCAGCTGCTTCATGATGCAGCCCTTGATGAAGCTCTTGGTGGCCGGCTTGGTC
TGCCGGGACACGATGCCGTTGTTATCGAGGATGTACTTTCGGTAGATGGCTCTGGCCAGCTTCAGCCTCTTCT
CCTCGTTCGAGTCACAGGGCTCCAGCTTCCTGAAGCCAGTGCAGGCAAACCAGAAGTCCAGCAAGTCAGCACA
GCCCTCCTGCTTCAGGAAAGTCCTGAACAGGCTTATCCCATCTTGATCATCCAGCAGGGAGTGCAGTGACTCA
GCCCACTTCAAGTACGGTGGGGTGGGGGAGGCACTGCCCTCAGGCTCATAGCCCAGGTCCAGATCCGAACGCC
TCGGAGTGGCGGTCGAAGTCTCACCTTTAATGCCAACACCTTTCCCGGAGCAGAAGCTGTAGCTGGCGGGCCT
TGGGTCTGTGGACACCAGTTCTCCCTCCTCACCAGGCACTGGGGGTCGGGGAGCATCCTCGGTGAAACTTGCT
CCAAGGTCCAAGGGGAAACCCTGCTCCTGGACATTCATTTGGGACTCTGTGCGTCAATGCACAATGAGCGCT
GCACCCTAATACATCAGTACTTAACAGCTCCAAAGTCAATCAATCTGTCCTGTTGAAACCAT???????????
???

????????????TAAGT-ACTTAGGTTATTTATAGGACAGATTATTTTTCTGTGTTGTTTTG----
CTTCTTTG-CATATTAATATTTATTTATAGGTGTAGTTAAGCTTT--
ATTATTAGTTAGCATAAATTTGGCTTGGAGTCA----
CCAAAGCAGAAATGTTGGATTTCATTTTTATGAGTTATTTCCTACACATTTCTAAATTGATGTAGCTTTGTCA
CTGCTTGATTGATTGTTTTT---
AAAGTGGAGGGGGTAAAATTAATTTATACTGAATCAAACCAGTTTCTCTTAATTAGTA---------------
----CATTACTAACATTGAAAAGAAGTAAAAGGAATTTTAATAACTTAGGAGTATTTGCATAGTTA-
TGTTCAGAACACAAGCTTTAAATTAGT-GAGGAAACCA---------
GAAGTTTGATTTAAGCACTCATACTCCTTTATTTTCCTTTGTTAGCAAT????????????????????????
???
???
???
???
???
??????????????????TACCCGCTATAGCTTCTGCAAACAGTAAAGATACCACTAAAACCCCTTCTGCAGT
TCAAACTAGCACCAGCCACAACCCTGGACTTCCTCCTAATTTTAACGAATGGTACGTCTGAGGACAAACACAA
ACCCTGTTAACCATAGAATGGACCAAATGCATTTTTAAAAGAAAACTGAGACCAATCAGATGGAAATGGAGTT
TTAAGGCAAGAGGCCATATATAGGGCTACATCTTGTTAATTGCAATTGTCCAGGAAGGTTTTGGGCAAGATCC
AAAAGTAGCCATGCCCTTTTCTCAGGATTAGAAAATATGTTTTGGCGTTTGAAGGATTTTTT--
ACAAAATCTTTACACTGCTTTTTCTTCCCCTTCCTCTTGCTCTCTGCACACCCCATTCCTAAACTCCTGCAAT
TCATTTTAACACTTGTCCTGTTTCTTGAGAGGAAGTTATAGAAGGCTTGTTGGTGGTGGTGATGTTAAACTGA
TGGAAATTCTTTTTCGCCTTAGTGGTGATTGTTTAAACTCTCACAGTCTTAAACCGTGCCAAAGTCCTGTTAT
A
Chiropotes ???ACTACAGAAATGTCAC-
TTCATTTCATGCCAGTTCCCTT-
CCGTCATGAGCATGGACTACATGGTATACTTCAGCTTCTTCACCTGGATTTTCATTCCCCTGGTCATCATGTG
TGC--AATCTATCTTGACATCTTTTACAT-
CATCCGGAACAAACTCAGTCAGAACTTCTCTAACTCCAAAGAGACAGGTGCATT---
TTATGGACGGGAGTTCAAGACGGCAAAGTCCCTGTTTCTGGTGCTTTTCTTGTT-
GCTTTGTCATGGCTGCCTTTATCCATCATCAACTGCATCACCTACTTTCA--------------TGGTGAG-
TACCACAGCTTGTGCTGTACT--
GGCATCCTGCTT???ATGTTCATAA
ATCGCTGATTATTTTCAACTAACCACAAAGATATTGGCACACTATATTTACTATTTGGTGCCTGAGCAGGGGC
AGTAGGGACAGCCCTAAGCCTCCTGATCCGAGCGGAATTAGGTCAGCCTGGGAGCCTAATAGAAGACGATCAT
ATCTATAACGTTATTGTTACCTCTCACGCATTTATTATAATTTTCTTCATAGT---
AATGCCAATTATAATTGGAGGATTTGG---
AAACTGACTTGTCCCTCTTATAATTGGTGCTCCCGATATAGCATTTCCCCGAATAAACAACATAAGCTTTTGG
CTTCTTCCCCCA--TCACTTCTTCTCCTACTTG-
CATCGTCGACTCTAGAAGCAGGTGCTGGAACTGGTTGAACGGTATATCCGCCACTAGCAGGAAACATATCCCA
CCCAGGAGCCTCTGTAGACTTAACTATTTTCTCACTTCACTTAGCAGGCATTTCTTCTATTCTAGGAGCTATT
AATTTTATTACAACAATTATTAATATAAAGCCCCCAGCTATAACCCAATATCAAACACCTCTATTCGTATGAT
CCGTGCTTATTACAGCTGTCCTCCTTCTTCTATCTCTTCCAGTTCTAGCTGCCGGAATTACTATGCTATTAAC
TGACC--
GAAACCTTAACACCACTTTTTTTGATCCTGCTGGTGGCGGAGACCCAATCCTGTATCAGCACTTGTTTTGATT
TTTTGGGCATCCTGAAGTATATATTCTTATTCTGCCAGGCTTTGGAATAATCTCACACATTGTAACATATTAT
TCTAACAAAAAAGAACCATTCGGATACATAGGTATAGTATGGGCTATAATGTCCATTGGTTTCCTGGGATTTA
TCGTATGAGCTCACCACATATT-
CACAGTAGGAATAGATGTAGACACCCGCGCATACTTTACATCAGCTACTATAATTATTGCTATTCCCACCGGG
GTAAAAGTCTTTAGCTGATTAGCTACATTGCATGGCGGTAACATTAAATGATCCCCCGCAATACTATGAGCCC
TAGGTTTTATTTTTCTCTTTACCGTTGGCGGATTAACTGGAATTGTTCTAGCCAACTCATCATTAGATAT-
TGTCTTACACGACACATACTATGTAGTTGCCCATTTCCATTATGTTCTGTCTATGGGAGCAG-
TATTTGCCATCATAGGGGGCTTTATCCACTGATTTCCCCTATTTTCAGGTTATACCCTTGACCAGACTTATGC
AAAAATTCACTT-
TACTACTATATTTGTAGGCGTAAATATAACCTTTTTCCCGCAGCATTTCCTCGGCCTATCAGGTATGCCACGG

CGTTACTCAGACTACCCCGACGCCTATACCACATGAAATATTATCTCATCTGTAGGCTCATTTATTTCACTAA
CAGCAGTTATTCTAATAATTTTCATAATCTGAGAGGCCTTTTCCTCAAAACGTAAAGTCTTAACCGTTGAACA
ACCTCACACTAACCTAGAATGACTTTATGGCTGCCCTCCTCCTTACCACACATTTGAAGAAGCTACCTACGTA
AAATCGCTAAGCGAAAAAGGAAGG????????????ATGGCACACCCAGCCCAATTAGGCTTACAAAATGCTG
CATCTCCTATTATAGAAGAACTGATCGCATTTCACGACCATGCTCTCATAATTATTTTCCTAATTAGCTCACT
AGTCCTTTACACCATCTCCTTAATACTTACTACAAAATTAACTCACACCAGCACTATGAACGCCCAAGAAATC
GAAATAATCTGAACTATTCTACCAGCCATTATTCTCGTTATAATTGCTCTCCCATCCCTACGTATCCTCTACA
TAACAGATGAATTTAACAAACCTTATTTGACCCTCAAAGCGATTGGTCACCAATGATACTGGAGTTACGAGTA
CTCAGACTACGAAGACTTGGCCTTCGACTCTTATATCGTACCCACATACTTCCTTGAACCCGGCGAATTTCGA
CTCCTTGAAGTAGATAACCGAACAACATTGCCAATAGAAGCAGATATTCGTATACTAATCTCGTCACAAGACG
TATTACACTCATGAGCCGTCCCATCACTAGGCATTAAAACAGACGCAATTCCCGGACGTTTAAATCAAGCCAT
AGTAGCCTCTATACGACCAGGTTTATTTTATGGGCAGTGCTCAGAAATTTGCGGATCAAACCATAGCTTCATA
CCCATCGTACTAGAATTCATTTACTTCCAAGACTTTGAAGTATGAGCATCATACTTATATATTGTAT??????
ATGACAGCTACCCGTAAAACACACCCACTAATAAAAATTGTTAACACCTCGTTCATCGATCTCCCCACACCAC
CTAATATCTCATCCTGATGAAATTTTGGCTCACTCTTAGGCATCTGCTTAATTACCCAAATCATCACAGGCCT
ATTCCTAGCTATACACTACACATCAGACTCCTCCACTGCTTTTTCCTCAGTCGCCCACATCACCCGAGATGTA
AATTACGGCTGAATAGTCCGCTACCTTCATGCTAATGGCGCTTCCCTATTCTTCATATGCCTATTTCTTCACA
TTGGACGAGGCCTATACTACGGATCCTTTCTCTTCCTAAATACATGAAACATTGGCATCATCTTATTACTCAC
AACTATAGCTACAGCATTTATAGGTTATGTACTCCCATGAGGCCAAATATCTTTCTGAGGGGCCACAGTAATT
ACAAATCTCCTATCAGCTATCCCATACATCGGACCAGATCTAGTTCAATGAATCTGAGGCGGCTTCTCCGTTG
ATAAAGCCACTCTAACCCGATTCTTCACCTTCCACTTTATTTTACCTTTTATCATTGCAGCCTTAGCATCTAT
TCACCTCCTGTTTCTGCATGACACAGGTTCAAATAACCCATCAGGACTGACATCTGACCCAGACAAAGTATCA
TTTCACCCATACTATACAATTAAAGATATCTTAGGCTTAATCTTCCTACTTCTTCTTCTAATAAGCCTAACCC
TATTTATACCCGACCTTCTAACAGATCCAGACAACTATACTCCAGCCAATCCCCTAAACACCCCACCACATAT
TAAGCCTGAGTGATATTTTCTGTTTGCATACGCAATCTTACGATCTATTCCCAACAAACTAGGAGGCGTCTTA
GCCCTCTTGCTATCTATTCTAATCCTAGCAATTATTCCCCTAATTCACCTATCCAAACAACAAAGCATAGCAT
TTCGACCTATTAGCCAAGGCCTATTCTGAACCCTAGTTGCTGATCTATTCACACTTACATGAATTGGGGGCCA
ACCAGTCGAACACCCCTTTATTTACATCGGCCAAACTGCATCCACCTTATACTTTTTTATTATTAC---
CACTATCCCCCTCCTTACTCTAATTGAAAATAAGCTACTTAAGTGATAA?TTAGGTTAAATTTATTCAAAAGA
ATGAGAATTTATGGGTATAGCTGTTAGCTGGAAAGCCCTTCCTCACCTCCAATCCTCCTTTCAAACTACATTT
CC-
TCCTGAAATTTCTCTGTGGGATGAAATGTTCAATTATTAGTCAATCAATCAGTCTGTTATACGTTATCTGGCT
TTACATATATATGCCCTTGATAGTTCTTGGAAAATAATCCAATATTGTGTTGTTTTGGTGTCTTTGTATTGGA
GATGGAAGCATTTTGTATGAATCACCTCTTTAACTGTGAGGAGTTGAATTGTCTTAGTTCTGCTTGCCTAATG
ACCAGGAATAAAAAGACATCACAGTCTCTAACCCCACTTTTT-
GGTGGAAAGAAAATCTAAAAGTAACAACATTTAAGGGAACAAAATATCAATAATCAAAGAAACTCCCCTGTGA
AACTGTAATATTATTTT-
AAAATCAGCATTGTTCTAAGGTAGCTGAAATGTGTGATTTTTCTATTTTGCCTTTTCACTGACAATATACCCA
TAAATCT-
TGGTGGTGATTTTCTCTCCAAAGTTAATTTCTGGGCCATTGGTGACTACACTATCTGTTGTTCTAAATTGTTG
TCTTAATTGCATTCTTTAAAAATGTAATGTTGAAGGCACATAATATTGAAGATGACTCCAGGATGAACCCTGA
GTTTGCAGACCGAA-
TAAAACAGCTCGATAAAGAGGCGTCTTACTACCGTGATGAGTGTGGCAAGGCCCAAGCGGAAGTGGACCGGTT
GCT?????????????????????????????????CAAGTACTTTGATCTTTCTTGGAGCAGGTTCCTTCAAAA
CGCTTCTCCAGAGGACGGAAGTCCCTCCAGTCTGGCTCGCTGCTCGCAGTGGTGGGGAAAGCTGAGGTAATTC
CCCGGTGGGAAATCTGCGAGGCCCCTTTTACCCGTG--------
CCACCAGGCCCATCACTGATGGGCCTAAGGGCCTCATCTGATACTCTAAGACGGGCTTCTCCTGTCGGATCTG
GGCCTCTCGGACTTGAGACTCTCTAGAACGAGCCTCTCGGGCCTGGGCTTCTCGGGTTCTGGCCTCCCTGACA
TGACCTTCCCAAGTGTGGGCCTCTCTG--
GCATAGGCTTCCTTGCCACAAGCCTCTCGAGTATAGGCCTCCCTGGTGTGGGCCTCCCTGGCCTGAGCGTCTC
GGGCACGTGCCTCCCGGGCCTCAAGCTGCTCCCGCCGAAGCTCCCAATACAACAACTGTTTCTGGATGGTCA-
CTAGCCGTTCCTCCTCTGTCTCCATTGCCCCAGGTGGCCGGGAGGAAGAAAAGGGCTCAAAGTTCAAGAAGGG
ATCAAACATCTCAGAGCTTCTACCATGGAGGTCATAAAGGCAGTCATCCCCAGGTGGAGAGTTCTCAAGACTG
TCATCTGGCTCATAGAACTCATATAGGGCATCTCCACTGTAGCTGTCTCGGGGCAGACAATCCCTACGGACAA

GCCCCAGGGCCTCACCTGA??????????????????????????????TTGATGAACTAAAAATGGTTCCTG
AAGGTCAGATTCATGTACAGTGAATGTTCTCCACTGTTGTGGTGGACATGTTTCATGTGTGGGGATGTTGGTG
TGCAAGTTTCATGTGTTTGGAAAACACTAGCTAGAACATTCAAAAAATTTCATTAACCCAGCTCTGGAAGGTC
ATCTATGAATTCTTCCTATTCCAGTGGCCTTTT-
AAGTGTAGATGTAGATACACTGTAATTTAAAACAGATGGGCATGTCTGAATATTTTCATATGCAAAATGGGAC
AGATGTATATGAATGGATAAATTATCCTTTTAAAAAGATACTTTGTAGCACGAAATATCACCAATGATTGGAA
CAGTGAACCTGCATTCAGTCTAAGGCAAACACCAGGAAGACATTATCAAAACATCTAA-
CTAACTACAAACCAATTCCAATTAGTCTACTTTGTCTTCATACAGAAAAAA-TTTTTTTGATTTTTAAA---
TATGTATTGGGTGCCAATCCGTATCCTCT-
GAGCTGCTAAAGCCAGCATATCACAATAGAAGTTTACTTTTCC??????????????ATGCCCATAATCAACC
TTCTACTACTAACTATTCCTATCCTAATTGCTATAGCATTCCTAACTCTCATAGAACGAAAAATCTTAGGCTA
TATACAACTTCGCAAAGGTCCTAATATCGTAGGTCCCTACGGACTGCTTCAACCAATTGCAGACGCAATAAAA
TTATTTACAAAAGAATCCCTTCTGCCCACCACATCTACTATAACTATATATATTATAGCCCCAGCCCTAGCTC
TCTCCATTGCTCTCCTCCTATGAACTCCCCTCCCTATACCATACCCCCTAGTTAACCTTAATCTAGGCTTACT
ATTTATACTAGCAATATCAAGCCTAGCTGTATACTCAATTCTATGATCCGGATGAGCATCCAACTCAAACTAC
GCACTAATTGGCGCCCTACGAGCCGTAGCCCAAACAATCTCATACGAAGTAACATTAGCCATTATTCTTCTAT
CAGTACTACTTATAAGCGGCTCATTTAATCTTCACTCACTAATTACAACACAAGAACACTCTTGACTTCTTCT
GCCATCTTGACCCCTAGCTATAATATGATTTATTTCTACATTAGCTGAAACCAACCGAGCTCCCTTTGATCTA
ACAGAAGGCGAATCAGAACTAGTTTCAGGCTTTAACATCGAATACGCTGCAGGTTCATTCGCTCTCTTTTTCA
TAGCAGAATATATAAACATTATCATAATAAATGCTCTAACTACCACTATTTTTCTAGCAACACCATACACCCC
AACTATACCAGAACTCTATTCAATTTATTTCGTAACTAAAACCCTCATGCTAACTGCCCTATTCTTATGAATT
CGAACAGCATACCCCCGACTACGTTATGATCAACTAATACATCTTCTATGAAAAAAATTCTTACCTCTCACAC
TAGCACTATGTATATGATACATCTCAATACCAACCCTAACAGCTAGTATTCCACCCCAAACATA?ATTAACCC
TCTAGCACACCTCATTACCTCCCTCACAATCCTAATAGGAACTATAATCGCAATCCTAAGCTCACACTGATTT
CTTATCTGAATAGGCCTAGAACTAAATATATTATCCATTATTCCAATACTAGCCAAAAGCATAAATCCCCGCT
CCACGGAAGCAGCCACTAAGTACTTTTTAATCCAAGCAACCGCATCTATAATTCTATTAATAGCTATTATTTT
AAACAATCTACTATATGGACAATGAACAATTAACCCATCCTTGAATCAAACTCTATCCACAATAATACTAGTT
GCCATCACAATAAAACTAGGCATAGCCCCCCTTCACTTCTGACTTCCCGAAGTAGTCCAAGGCATCCCTTTAA
TCCCAGCTATACTTATCCTTACATGACAAAAACTAGCTCCAATATCAATTATTATCCAAATCTTTCCATCTAT
CAACACAAATATTATTCTAATAACCTCAATCCTATCTATTATAATTGGCAGCTGAGGAGGACTTAACCAAACA
CAATTACGTAAAATCCTAGCCTATTCATCAATTACCCACATAGGCTGAATAATAGCTATTCTATACTACAACC
CAAACATCACTATTCTTAGCCTATTCATCTATCTACTCCTAACAACTTCCACATTCATGATTTTCTACCTAAA
CTCAAATGTAACAACCCTATCAATATCACATACTTGAAACAAGCTTACATGAATAATGCCTACAATCACATTA
ATTATATTATCCCTAGGAGGTCTACCCCCACTAACAGGCTTCTCTCCTAAATGAGCTATTATACAAGAACTAA
CAAAAAATAATAACCTTATCATTCCCCTTATTATAGCTATATTAACACTAATAAATCTGTATTTTTATATACG
CCTCACATATTCCATCTCAATAACAATATTTCCTACATCCAACAGTACAAAAATTAACTGACAACTAAAACAC
ATAAAATTATCACCTCTCCTCCCCCCACTTACAGTGTCTTCTCTATCCCTTCTTCCCCTAACTCCATTAATAC
TAGTTAGCT??
????????????TGGCCACTCTTATTCTCAAACAATAAAAACTACCCAGCCTTTTCATGAAAGAT-
GATTGCAATTTCCATTGGCCTGTATATGCAATAAAAAG---
CCATAACTCATCAAAATTTTAGCTATTTTCTCTTAAGATA--
GAAGAGCATTTTGCTTGAACATTGAACGTGACATTAAATATT-
CATGAAACTGAATAGAGTTTAAGTACTAAG---
TAAAACTCTCAAATTCTTTTCAACTAGATTGAAATTCTGTTTGTACCAGGTAAAAACTGCCGCTAACCTGAAG
AGGTGTTCAAAAAC-
ATCGTAAAGAGTTTGTACAATGTGTTCAAAGTGCTACAGTTATTTGGGATAGATTTTTCTGAAAAGCCTGTTC
CTCCTTTGAAATTGATTCATGATAACAGCCTCCATTTGGGTCAAACAGTATTTAACCGCTTGTAAA-
TTTTAATT-
AAATGATTTGTCACTTTGAACACATAGTTTGTCCCCAAGACCTTGAGGATTACTTGAACGGCCCCTTCACTGT
GGTTGTGAAGGAGTCTTGTGATGGAATGGGAGATGTGAGTGAGAAGCATGGGAGTGGGCCAGTAGTCCCAGAA
AAGGCAGTCCGTTTTTCATTCACAATCATGAAAATTACTATAGCCCACGACTCTCAGAACATGAAAGTGTTTG
AAGAAGCCAAACCTAACTCTGAGCTGTGTTGCAAGCCATTGTGCCTTATGCTGGCAGATGAGTCTGACCATGA
GACCCTGACTGCCATCCTGAGTCCTCTCATTGCCGAGAGGGAGGCCATGAAGAGCAGTGAATTAATGCTTGAG
ATGGGAGGCATTCTCCGGACTTTCAAGTTCATCTTCAGGGGCACTGGATATGATGAAAAACTTGTGCGGGAAG

TGGAAGGCCTTGAGGCTTCTGGCTCAGTCTACATCTGTACTCTTTGTGATGCCACCCGCCTGGAAGCCTCTCA
AAATCTTGTCTTCCACTCTATAACCAGAAGCCATGCCGAGAACCTGGAACGTTATGAGGTCTGGCGTTCCAAC
CCTTACCATGAGTCTGTGGAAGAACTGCGGGATCGGGTAAAAGGGGTCTCAGCCAAACCTTTCATTGAGACAG
TCCCTTCCATAGACGCACTCCACTGTGACATTGGCAATGCAGCTGAGTTCTACAAGATCTTCCAGCTAGAGAT
AGGGGAGGTGTATAAGAATCCCAATGCTTCCAAAGAGGAAAGGAAAAGGTGGCAGGCCACATTGGACAAGCAT
CTCCGGAAGAAGATGAACCTCAAACCAATCATGAGGATGAATGGCAACTTTGCCAGGAAGCTCATGACCAAAG
AGACTGTGGATGTGGTTTGTGAGTTAATTCCTTCTGAGGAGAGGCATGAGGCTCTGAGGGAGCTGATGGATCT
TTACCTGAAGATGAAACCAGTATGGCGGTCATCATGCCCTGCTAAAGA--GTGCCC-
AGAATCCCTCTGCCAGTACAGTTTCAATTCACAGCGTTTTGCTGAGCTCCTTTCTACCAAGTTCAAGTATAGG
TATGAGGGGAAAATCACCAATTATTTTCACAAAA??????????TTATGGCACCCGACAGTTGTCATTATGTT
TGGAAACACTGCCAGCCCATGGAGATGAGGATAAAGTGRACATTTCTCTGCTGCATCAGGGTGAGAATCTTTT
CGAACTGCACATCCACCAGGCCTTCCTGACATCTGCCGCCCTGGCTCAGGCTGGAGACACCCAACCTACCACA
TTCTGCACCTATTCCTTCTATGACTTTGAAACCCATTGTACCCCACTGTCTGTAGGGCCACAGCCCCTCTATG
ACTTCACCTCCCAGTATGTGGTGGAGACAGACTCCCTTTTCTTACACTACCTTCAAGGGGCTTCAGCCCGGCT
TGATCTCCACCAGGCTGTGGCCAGTGAGCACAACACTCTTGCTGCAGGATGGATTTGCTTTGACAGGGTGCTA
GAGACTGTGGAGAAAGTCCATGGCTTGGCCACACTGATTGGTAAGTGCTGTCGGCTTCCTGCAGCTCCTA-
GCACCAATGCAGAATTTCCCAAACTCTATAGCTGCTTTTCTGGTCGTTTCTTATTTTCTTCCTTTCTCTTTGT
TCTTGATCCTTGCCATACCATCTGTATCCTCCATGCTTTCA-
TACTTGCTGCTGCCCAGGAGCTGGTGGAGAAGAGTTTGGRGTTCTAGAGTACTGGATGAGGCTGCGTTTCCCC
ATAAAACCCAGCCTACAGGCATGCAATAAACGAAAGA?????????????AGCACTGCACATCAAAGGCAGCT
TGGAGTCTGAAAAACATCAATACATCATCCATGGAGGGAAAACACCTAACAATGAGCTTTCAGATAAGATTTA
TGTCATGTCTGTTGTTTCCAAGAACAACAAAAAGGTTACTTTTCGCTGCACGGAGAAAGACTTGGTAGGAGAT
GTTCCTGAAGCCAGATATGGTCATTCCATTAATGTGGTATATAGCCGAGGGAAAAGTATGGGTGTTCTTTTTG
GAGGACGCTCATACATGCCTTCTACCCACAGAACCACAGAAAAATGGAATAGCGTAGCTGACTGCCTGCCCCA
TGTTTTCCTGGTGGATTTTGAATTCGGGTGTGCTACATCATACATTCTTCCAGAACTTCAGGATGGGCTATCT
TTTCATGTCTCTATTGCCAAAAATGACACCATCTATATTTAGGAGGACATTCACTTGCCAATAATATCCGCC
CTGCCAACCTGTACAGAATAAGGGTTGATCTTCCCCTGGGTAGCCCAGCTGT-
GAATTGCACAGTCTTGCCAGGAGGAATCTCTGTCTCCAGTGCAATCCTGACTCAAACTAACAATGATGAATTT
GTTATTGTTGGTGGATATCAGCTTGAAAATCAAAAAAGAATGATCTGCAACATCATCTCTTTAGAGGAAAACA
AGATAGAAATTCGTGAGATGGAGACCCCTAGATTGACCCAGACATA????TGTGTCTCAGCATCTTCCTGCTC
CCCACCACACTGAGCCCACGATGACCCAGTGCTCCACAAACATATGTAGAATGGAAGTAAAACCTCAGG----
------GATAATTAAAGGGCCGGGTGCGGTGGTTCATGCCTATAATCCC-------
TAGGAGGCTAAGGCGAGTGCATCACAAGGTCAAGAGTTCAAGATCAGCCCGGCCAAGATGGTGAAAACCCGTC
TCTACTAAAAATA--CAACAATT-----
AGCTGGG--
---------------------CGTGGTGGTGGGCACCTGTAATCCCAGCTACTTGGGAGCCTGAAGCAG-
AGAACTGCTTGAACCTGAGAGGCAGAGGTTGCAGTGAGCCGAGATCACGCCACTGTACTCCAGCCTGGACAAC
A-GAGTGAGACTCCATCTCAA--

--AAAA-
TAATAATAAAAAGAAAAGATAGTTAAAGAAGATTCACTACAGTAAGAAACCAGCCAAGAAATTGCACTGAAAG
TAAAGGCTT-
GGGTGACAAAATTTCCAACTGTGTTCCAAGAAACATAAGTTCCAGCAGGAGTGCACTTAATCTGATTTT-
CCTCAAAGT--
CTCTCTAATTTGTAGGGAAGGACATTCATTTTCATTAGCTTCATGTGAGCTAGAAAGACTCCACGATTGAAGC
GCTAGAACCCCTGGTAAACATGCCAGCAAGCAAGGGCACCTTCCCCAGAGGCTCAAACTAGAATATAAAACTG
TTGGCATTGGTAAGGAGCCTTGAACTCTAAACCCATAGGCTCAAGTCAGGAA-
CTCAGGAAGCCTATCAACACTGGGATACAATTTTGCCACCTTAACACT---
TATCCCTTACTT???
???
???
???
???
??TTAGGAACTCAAGGCCCTCAAAGATCTT

TACTGCCATAAAGCAGAGTAACT-GTCTTTGACTTGGACCTTGTTTACTGTGAACTCTAATC-
AAGGCAGGACATGCAGCAGTTCTACTTA-CTGCACTGTGGACGTGTGGAAAGGACACTTGTGA---------
ATCCAGTTGGGATTAGTGTTTGAAACTGAATGGCGAATGTTGTTCCAGGATGTGGAATGCAGCCGTGATCACA
CTTGCCTACA-GAGCTTACTTTGAACTATTTGTCAATAGCATGCAAAAAA-
TGCTGTGTCTGCCCTTTGCTTCTGCTTTTTCCAGGGAAGCTGCCAAAGAGTGTCGACGTCGAAAGAAAGAATA
CGTAAAATGTCTGGAGAGCCGAGTTGCAGTGCTGGAAGTCCAGAACAAGAAGCTTATAGAGGAACTTGAAACC
TTGAAAGACATTT??
???
???
???
???
???
???
???
???
???GTATCTAAATGTGTGGATTCCA
GCACCTAAACCAAAAAATGCCACTGTAATGATATGG-
ATTTATGGTGGTGGTTTTCAAACTGGAACATCATCTTTACATGTTTATGATGGCAAGTTTCTTGCTCGGGTCG
AAAGAGTTATTGTAGTGTCAATGAACTACAGGGTGGGTGCCCTAGGATTCTTAGCTTTGCCAGGAAATCCTGA
GGCTCCAGGAAACATGGGTTTATTTGATCAACGGTTGGCTCTTCAGTGGGTTCAAAAAAATATAGCAGCCTTT
GGTGGAAATCCTAAAAGTGTAACTCTTTTTGGAGAAAGTGCAGGAGCAGCTTCAGTTAGTCTGCATTTGCTTT
CTCCTGGAAGCCATTCCTTGTTCACCAGAGCCATTCTGCAAAGTGGATCCTCTAATGCTCCTTGGGCAGTCAC
ATCTCTTTACGAAGCTAGGAACAGAACGTTGGCCTTAGCTAAATTTACTGGTTGCTCTAGAGAAAACGAGACT
GAAATAGTCAAATGCCTTCAAAACAAAGATCCCCAAGAAATTCTTCTGAATGAAGCATTTGTTGTCCCCTACG
GAGCTCTCTTGTCAGTAAACTTTGGTCCAACAGTGGATGGTGATTTTCTCACTGACATGCCAGACATATTACT
TGAACTTGGACAATTTAAAAAAACCCAGATTTTGGTAGGTGTTAATAAAGATGAAGGGACAGCTTTTTTAGTC
TATGGTGCTCCTGGCTTCAGCAAAGATAACAATAGTGTCATAACTAGAAAAGAATTTCAGGAAGGTTTAAAAA
TATTTTTTCCAGGAGTGAGTGAGTTTGGAAAGGAATCCATTCTTTTTCATTACACAGACTGGGTAGATGACCA
GAGACCTGAAAACTACCGTGAGGCCTTGGATGATGTTGTTGGGGATTACAATATCATATGCCCGGCCTTGGAG
TTTACCAAGAAGTTCTCAGAATGGGGAAATAATGCCTTTTTCTACTACTTGA???????TTGTGCTAATTGTG
AGAAAATATGCTACTTTGATCAAACTAGAATGAAGGAAATAAAAAGACACTGTGGATCAACTTGTGATAAAGA
GCAGCTCATCCTCACAGCTTTTTGATTGTTGCTATAGTTATCT---
ATTCAACATGGGAGGTACAATACAGTTAACATTTCATGACCAGTAGCCTGTTGCGTGCTTATTGTTAAAAGGA
ATTTAACATACAAGTTTTTGTATGGATTGTTCGCCTAATTCCGATTCCTCGTGCTGACTGGCATGAGACAGGG
AGCTGGATTGTATTAAACTGGTTTTATTGTGATGTGACTATGATGGATGTGACAAGGGCCTCTATTATTCGCT
GGGGAGTGTGGCCGTTAATGAAGCCATTAGCTGAGCTAAGGCAAGTGAAGAAAATAGGGAAATTACCATTATT
CTGAAGCCATTAAGTTGTAATCCACCACTTTCTCTCCTTATTCAAAAAGGCATCTTCTTTTTAA?????????
TTCCTTACTATGGTTATTTCATACTTCGGTTGCATGAAGGCTGCCCCCATGAAAGAAGCAAACGTCCGAGGAC
AAGGTGGCTTGGCCTACCCAGGTATGCGGACCCATGGGACTCTGGAGAGCGTGAATGGGCCCAAGGCAGGTTC
AAGAGGCCTGACGTCGTCGTTAGCTGACACTTTTGAACATGTGATAGAAGAGCTGTTGGATGAGGACCAGAAA
GTTCGGCCCCATGAAGAAAACAATAAGGACGCAGACTTGTACACGTCCAGGGTGATGCTCAGTAGTCAAGTGC
CTTTGGAGCCTCCTCTTCTCTTTCTGCTGGAGGAATACAAAAATTACCTGGATGCTGCAAACATGTCCATGAG
GGTCCGGCGCCACTCTGACCCTGCCCGCCGAGGGGAGCTAAGCGTGTGTGACAGTATTAGTGAGTGGGTAACG
GCGGCAGACAAAAAGACTGCAGTGGACATGTCGGGCGGGACGGTCACAGTCCTTGAAAAGGTCCCTGTATCAA
AAGGCCAACTGAAGCAATACTTCTACGAGACCAAGTGCAATCCCATGGGTTACACAAAAGAAGGCTGCAGGGG
CATAGACAAAAGGCTTGTGAAA??????????????????????????TCACTACAGTCTTTGAGCAGATGCA
GAACTAGACCCC--
GCCATGGCAGCCTCTGAAGTTGGACAGCAAAACCATTGCTTCACTACCCATCGGTGTCCATTTACAGAATAAT
GTGGAAAGAAACAAACCCCTCTGTCTTATGATTTACTCATTATCGCCTTTTGACAGCTGTGCTGTAACACAAG
TAGATGCCTGAACTTGAATTAATCCACAGATCAGTAATGTATTCTCTCTCTCTCTCTCTCTTT----------
--------ACATTTTGGTCTCTACACTACATTATTAATGGGTTTTGTGTACTGTAAAGAATTTAGCTGTTTC-
AAACCTAGTGCATGA-
ATAGATTCTCTCCTGATTATTTATCACATAGCCCTTTAGCCAGTTGTATATTATTCTTGTGCTTTGTGACCCA
ACTAAGTCCTACTTTAAATATGCTTTAA-GAATCGATGGGGGA---

TGCTTCACGTGAACGTGGGAGTTTAGCTGCTTCTCTTGCCTAAGTATTCCTTTCCT-
GATCACTATGCATTTTAAAGTTAAAACATTTTTAAGTATTTCAGATGCTTTAGAGAGATTTTTTTTTTCCATG
ATTGCATTTTACTGTACAGATTGCTGCTTCTGCTATATTTGTGATATAGGAATTAAGAGGATACACG??????
?????????????????????????????????????GAGAGCACTAAGCCACTTCCAGCCCTGCTGTGAACC
AGGACCGTAGGAGCCATCTTTTTCATGTGGTCAGCTTTGGAAGCATCTACATCCACCACTTTAGAAGACAAGT
CTAGTGGCTTATCTGTGACGTCTTTGCTAACGGTCTGCTTCTCCAACAGAGGAGGTGAGCTGCCGTCTTTTCT
GTCTTGAACCGCTGTCTTCCGGGCATGCCCGGGCACTGGCTGGGCACCTTCGCCCCCTTCCGGAGCCTTGGGA
TACTTGCCGTTGGAGAGCCTGGCCGCGGGGAACTCGCTGCTGACTGTCATGTATGGCTTGGACAGGGCAACTG
AAGGAGAGGTGGAGATCCTGGCATAGTGCTTGTGGAACTCCGAGTAGGTGTCTGCAGTAGGCTGGGTGGGAAG
GTGGACCCGGGGTGAGGGCCGAGGCGAGGGGGGCAAGAGGAGAGCCGTGTCCCCCGGCAGGCCACTGGTGACT
GCCTTGGCAGAGGGAACCCTAGGCTGTTTACTGTTCTGGATGTGAGGATAGGCATGGGAATCAACGGATTCC
CAGGGCTGACCCCCATCTTCCACGGGAGGCTTTTGTCTGCGCAATGGACAAGAGGCGGGATGGCTGGGGAGGC
CGAAGGCGTCGAGAGCCTCATGGGTGATGCCAAGGACGACGGGATGTGGGGACTGACGTAGTGAGGTGGTGGC
AGGTAGAGAAAGCGCTCCCCGTTGGTGCAGACTGGAGAATACAGAGGCTGGGCCAAGCTGTAGGACTGCT???
???
???
???
???
???
???
???
???
????????????????????????????????GAGCAGTGTCTCTGCCATCGTCCTCGTCCACGTCCTGGTCA
CATTTCCATTCCTCATCTTCATTTAAAGTCGGCAGGTATCCAGATATGCCCTTCCCTGTCCCTGACCCAGAGC
TCTGGTCACTACAGACCTTCGGGCTCTCCGAGCCTGTCCTCGTATATTCCAAATAAATATCAGACTTAAGGAA
GGAGGGGTAGGTGTTTTCCTCCATAGTGGCCTGGATTTCTGTCTGGGCCTGGTCAAACATGGCAGGATCGATC
AGCTGCTTCATGATGCAGCCCTTTATGAAGCTCTTGGTGGCCGGCTTGGTCTGCCGGGACACGATGCCATTGT
TGTCAAGGATGTACTTTCGGTAGATGGCTCTGGCCAGCTTCAGCCTCTTCTCTTCGTTGGAGTCACAGGGCTC
CAGCTTCCTGAAGCCGGTGCAGGCAAACCAGAAGTCCAGCAGGTCAGCACAGCCCTCCTGCTTCAGGAAAGTC
CTGAACAGGCTTATCCCATCCTGATCATCCAGCAGGGAGTGCAGTGACTCAGCCCACTTCAAGTACGGTGGGG
TGGGGGAGGCACTGCCCTCAGGCTCATACCCCAGGTCCAGATCCGAACGCCTCGGAGTGGCGGTCGAAGTCTC
ACCTTTAATGCCAATACCTTTTCCGGAGCAGAAACTGTAGCTGGCGGGCCTCGGGTCTGTGGACACCAGTTCT
CCCTCCTCACCAGGCACTGGGGGTCGGGGAGCATCTTCGGTGAAACTTGCTCCGAGGTCCAAGGGGAAACCCT
GCTCCTGGACATTCATTTTGGGACTCTGTGCGTCAATGCACAATGAGCGCTGCACCCTAATACATCAG?????
???CTAAAGTGTTAGCTAATTTTAAGTGAAC
AA-A--ATTACATGGATCAACTTTTGGCAGCATAAAAGAGGTATAATTTACAGTGGAGCAGGCTAAGT-
ACTTAGGTGATTTATAGGACAGATTACTTTTCTGTGTTGTTTTG----CTTCTTTG-
CATATTAAAGTTTATTTATAGGTGTAGTTAAGCTTT--ACTATTAGTTACCATAAATTTGGCTTGGAGTCA--
--
CCAAAGCAGAAATATTGGACTTCATTTTTATGAGTTATTTCCTACACATTTCTAAATTGATGTAGCTTTGTCA
CTGCTTGATTGATTTTTTTTT--
AAACTGGAGGGGGTGAAATTAGTTTATACTGAATCAAACCAGTTTCTCTTAATTAGTA---------------
----CATTACTAACACTGAAAAGAAGTAAAAGGAATGTTAATAACTTAGGAGTATTTGCGTAGTTA-
TGTTTAGAACACAAGCTTTAAATTAGT-GAGGAAACCA---------
GAAGTTTGATTTAAGCACTCATACTCCTTTATTTTCCTTTGTTAGCAATAATTATTGGCCCTGATGGCCATCC
TTTGACTGTTTATCCTTGTATGATTTGTGGAAGAAGTTTAAGTCGAGAGGTTTTTTGAAAAGACACATGAAA
AACCATCCCGAACATCTTGCCAAGAAG---
TACCACTGTACTGACTGTGATTACACTACCAACAAGAAGATAAGTTTACACAACCACCTGGAAAGCCACAAAC
TGACCAGCAAGGCAGAGAAGGCCATTGTTTGTGATGAGTGTGGGAAGCATTTTTCTCATGCAGGGGCTTTGTT
TACTCACAAAATGGTGCACAAGGAAAAGGGAGTCAGCAAAATGCACAAGTGTAAATTCTGTGAATACGAGACA
GCTGAA???
?TTCTGCAAACAGTAAAGATACCACTAAAACCCCTTCTGCAGTTCAAACTAGCACCAGCCACAACCCTGGACT
TCCTCCTAATTTTAACGAATGGTACGTCTGAGGACAAACACAAACCCTGTTAACTATAGAATGGACCAAATGC
ATTTTTAAAAGAAAACTGAGACCAATCAGATGGAAATGGAGTTTTAAGGCAAGAGGCCATATATAGGGCTACA

TCTTGTTAATTGCAGTTGTCCAGGAAGGTTTTGGGCAAGATCCAAAAGTAGCCATGCCCTTTTCTCAGGATTA
GAAAATATGTTTTGGCGTTTGAAGGATTTTTTT-
ACAAAATCTTTACATTGCTTTTTCTTCCCCTTCCTCTTGCTCTCTACACACCCCATTCCTAAACTCCTGCAAT
TCATTTGAACACTTGTCCTGTTTCTTGAGAGGAAGTTATAGAAGGCTTGTTGGTGGTGGTGATGTTAAACTGA
TGGAAATTCTTTTTCGCCTTAGTGGTGATTGTTTAAATTCTCACAGTCTTAAACCGTGCCAAAG?????????
?
Hylobates
?TACCCCCATGTTTGGCTGGAACATGAAACTGACCTCAGAGTACCACAGAAATATCACCTTCCTTTCATGCCA
ATTTGTTT-
CCGTCATGAGAATGGACTACATGGTATACTTCAGCTTCTTCACCTGGATTTTCATCCCCCTGGTTGTCATGTG
CGC--CATCTATCTTGACATCTTTTACAT-
CATTCGGAACAAACTCAGTCCGAACTTCTCTAACTCCAAAGAGACAGGTGCATT---
TTATGGACGGGAGTTCAAGACGGCTAAGTCCTTGTTTCTGGTTCTTTTCTTGTTTGCTCTGTCATGGCTGCCT
TTATCTATCATCAACTGCATCATCTACTTTAA--------------
TGGTGAGGTACCACAACTTGTGCTGTACATGGGCATCCTGCTGTCCCATGCCAACTCCATGATGAACCCTATC
?????????????????????ATGTTCGCCGACCGCTGGTTATTCTCCACAAACCATAAAGATATTGGAACAC
TATACTTACTATTTGGCGCATGGGCCGGGGTTCTGGGCACGGCCTTAAGCCTCCTCATTCGAGCCGAACTGGG
TCAACCCGGCAATCTCCTAGGCAATGACCATATCTATAACGTCATTGTAACGGCCCACGCATTCGTCATAATC
TTCTTCATAGT---AATACCCATCATAATTGGGGGCTTTGG---
CAACTGGCTCGTCCCTCTGATAATCGGCGCTCCCGATATGGCATTCCCTCGTATAAATAACATAAGCTTCTGA
CTTCTTCCCCCC--TCATTCCTACTGCTGCTTG-
CCTCCGCTATAGTAGAAGCCGGCGCCGGAACAGGATGAACGGTCTACCCTCCGCTGGCAGGAAACTACTCCCA
CCCAGGAGCCTCTGTCGACCTAACCATTTTTTCTCTACACCTGGCCGGAGTATCATCTATCCTAGGGGCTATT
AACTTCATTACCACAATCATCAACATAAAACCCCCAGCCATATCCCAATACCAAACACCCCTCTTTGTCTGAT
CCGTCCTAATTACAGCCGTCCTACTCCTCCTCTCCCTACCAGTCCTAGCCGCCGGCATTACTATACTACTAAC
GGACC--
GCAACCTCAACACTACTTTCTTTGACCCCGCTGGAGGAGGAGACCCTATCCTATATCAACACCTATTCTGATT
CTTCGGTCACCCCGAAGTTTATATTCTCATCCTACCAGGCTTCGGAATGATCTCACATATCGTAACACACTAC
TCAGGAAAAAAAGAACCGTTCGGATATATAGGCATAGTCTGAGCCATAATATCAATTGGCTTCCTAGGTTTCA
TTGTCTGAGCCCACCATATATT-
CACAGTAGGTATGGACGTAGACACACGAGCCTATTTCACCTCTGCCACCATAATTATCGCCATCCCCACCGGC
GTCAAGGTATTTAGCTGACTCGCCACACTCCATGGAAGCGACACCAAATGGTCCGCCGCAGTGCTCTGAGCCC
TAGGCTTCATCTTTCTCTTCACGGTAGGAGGCTTGACTGGCATCGTACTGGCAAACTCATCACTGGATAT-
TGTACTTCACGATACATATTATGTCGTAGCCCACTTCCACTACGTCTTATCCATAGGAGCCG-
TATTCGCCATCATAGGAGGCTTCGTCCACTGATTCCCCTTATTCTCGGGCTACACTTTAGATCAAACCTACGC
CAAAATTCACTT-
TGCCATTATATTTGTTGGGGTAAACTTAACCTTCTTCCCACAACACTTCCTTGGCCTCTCCGGAATACCACGA
CGTTACTCTGACTACCCCGATGCATACACTACCTGAAATATCCTATCCTCTGTAGGCTCATTTATTTCCCTAA
CAGCAGTAATACTGATAATTTTTATAATCTGAGAAGCCTTCGCTTCAAAACGAAAAATTCTAATAATCGAACA
ACCCTCCACCAACCTAGAATGGCTGTACGGATGCCCGCCGCCCTATCACACATTCGAAGAGCCCGTCTATATA
AAGCCTAGA???????????????????????????ATGGCACATGCAACTCAAGTAGGCCTACAAGACGCTA
CATCCCCTATCATAGAAGAACTAATCTCTTTCCACGACCACGCCCTTATAATCATCTTCCTCATCAGCTTCCT
AGTCCTATATGCCCTCTTCCTAACACTCACAACAAAACTAACCAACACTAACATTACGGATGCCCAAGAAATA
GAAACCGTCTGAACAATCCTGCCTGCTATTATTCTAGTCCTAATCGCCCTCCCGTCCCTCCGCATCCTTTACC
TGACAGACGAGATCAACGACCCCTCCTTTACTATCAAAGCAATCGGCCATCAATGATACTGGGCCTACGAATA
TACAGACTACGGTGGGCTGATCTTTAATTCTTACATGCTTCCACCATTATTTCTAGAACCAGGGGATCTCCGA
CTCCTTGAAGTCGATAACCGAGTGGTTCTTCCAATTGAAGCCCCTGTCCGTATAATAATTACATCACAAGACG
TCCTACACTCATGAACTGTCCCCTCCCTGGGTCTAAAAACAGACGCCATCCCAGGGCGCCTAAACCAAACCAC
ATTCACCGCTACACGCCCAGGGGTATATTACGGCCAATGCTCAGAGATCTGTGGGGCCAACCATAGCTTTATA
CCAATTGTCCTAGAACTAATTCCCTTAAAAATCTTTGAAATAGGG--
CCTGTATTCACTCTATAG????????ATGACCCCCCTGCGCAAAACTAACCCACTAATAAAACTAATCAACCA
CTCACTTATCGACCTTCCAGCCCCATCCAACATTTCTATATGATGAAACTTTGGTTCACTCCTAGGCGCCTGC
TTGATCCTCCAGATCATCACAGGATTATTTTTAGCCATACACTACACACCAGATGCCTCCACAGCTTTCTCAT
CAGTAGCTCACATCACCCGAGACGTAAACTACGGCTGAATCATCCGCTACCTTCACGCCAACGGTGCCTCAAT

ATTTTTATCTGCCTATTCCTACACATCGGCCGAGGCCTATACTACGGTTCATTCCTTTACCTAGAAACCTGA
AATATTGGCATTATCCTCCTACTCGCAACCATAGCAACAGCCTTCATGGGCTATGTCCTCCCATGAGGCCAAA
TATCCTTTTGAGGGGCCACAGTAATCACAAACCTACTATCCGCCGTCCCATACATCGGAACAGATCTAGTCCA
ATGGGTCTGAGGCGGCTACTCAGTAGATAACGCCACACTCACACGCTTTTTCACCTTTCACTTCATCCTACCT
TTCATTATCACGGCCCTAGCAGCCCTGCACCTTCTATTCCTACACGAGACAGGATCAAACAATCCCTTAGGCA
TCTCCTCCCAACCAGACAAAATCGCCTTCCACCCCTACTATACAATCAAAGACATCCTAGGACTATTTCTCCT
CCTCCTCATACTAATAAGCCTAGTACTATTCTCACCCGACCTCCTAGGCGACCCGAGCAACTATACCCAGGCT
AATCCCCTAAACACCCCTCCCCACATCAAACCCGAATGATACTTTTTATTCGCATACGCAATTCTACGGTCCG
TCCCTAATAAATTGGGAGGCGTACTAGCCCTCCTACTATCAATCCTCATCCTAGCAATAATCCCCGCACTCCA
CACAGCTAAACAGCAAAGCATGATATTTCGCCCACTAAGCCAGCTCACGTACTGACTCCTAGTAATAAACTTA
CTGATTCTCACATGAATCGGAGGACAACCGGTAAGCTACCCATTTATCACCATTGGACAAGTGGCATCCGCAC
TATACTTCACCACAATCCTAGTACTTATACCAGCCGCCTCCCTAATCGAAAACAAAATACTCAAATGAACCTT
TAGGTTAAATTTATTTAAGAGAATGAGAATTTATGGGTATAACTGTTAGCTGGAAAGCCCTTCCTCACCTCCA
AGCCTCCTTTCAAACTATATTTCC-
TGCTGAAATTTCTYAGTGGAATGACATGTTCAATYATTAGTCAATCAGTC----
TCTTATATGTTATCYGGCTTTACATAT----
GCCCTTGGTAGTTCTTGGAAAATAATCCAATATTGTGTTGTTTTGGTGTCTTTGTATTGGAAATGGAAGCATT
TTGTATGAATCACCTCCTTAACTGTGAGGAGTTGAATTGTCTTAGGTCTGCGTATCAAATGACCGGGAATAAA
--GACATCACAGTATCTAACCCTACTTTTT-
GGTGGAAAGAAAAGCTAAAAATAGCAACATTTAATGGAACAAAATATCAATAATCAAAGAAACTCCCCTGTGA
AACTCTAATATTATTTT-
AAAATCAACATTGTTCTAAGGTAGCTGAAATGTGTGATTTTTCTATTTTGCCTTTTCACTGACAATATACCCA
TAAATCT-
TGGTGGTGATTTTCTCTCCAAAGTTAATTTCTGGGCCATTGGTGACAGCATTATCTATCATTCTAAATTGTTG
TCTTAACTGCATTCTTTAAAAATGTAATATTGAAGGCACATAATATTGAAGATGACTCCAGGATGAACCCTGA
GTTTGCAGACCGAA-
TAAAACAGCTCGATAAAGAGGCGTCTTACTACCGAGACGAGTGTGGCAAGGCCCAAGCGGAAGTGGACCGGTT
GCTGGAGA????????????AGAGCTGCATCAGGCAGGTACTTTGATCTTTCTTGGAGCAGGTTCCTTCATAA
CGCTTCTCCAGAGGACGGAAGTCCCTCCAGTCTGGCTCGCTGCTTGCAGTGGTGGGGAAAGCTGAGGTAATTC
CCCGGTGGGAAATGTGAGAGGTCCCTGATACCCCTG--------
CTGCCAGGCCCATCACTGATGGGCCTAAGGGCCTCATCTGATACTCTAAGATGGGCTTCTCCTGCCGGGCCTG
GGCCTCTCGGACTTGAGTCTCTCTAGAACGAGCCTCTCGTGTCTGGGCTTCTCGGGTTCTGGCCTCCCTGCCA
TGAGCTTCCCAAGTGTGGGCCTCCCTG--GCACAGGCTTCCCTGCCATAAGCCTCTCGAGTATA---------
------
GGCCTCCCTGGCATGAGCTTCTCGGGCACGTGCCTCCTGGGCCTCAAGCTGCTCCCGCCGAAGCTCCCAATAC
AACAACTGTTTCTGGATGGTCA-
CTAGCCGTTCTTCCTCTGTCTCCATTGCCCCAGGTGGCCGGGAGGAAGAAAAGGGCTCAAAGTTTAAGAAGGG
GTCAAACATCTCAGAGCTTCGACCATGGAGGTCATAAAGGCAGTCATCTCCAGGTGGAGAGTTCTCAAGGCTG
TCATCTGGCTCATAGAACTCATATAGGGCATCTCCACTGTAGCTGTCTCGGGGTAGACAATCCCTGTGGACAA
GCCCCAGGGCCTCACCTGAATCATCCTCAAATCCAA????????????????????????????????????
????????ATTCATGTACAGTGAACCTTCTCCACTGTTGTTGTGGGCATGTTTCATGCGTGGGATGTTGATG
TGCCAGTGTCGCGTGTTTGGAAAACACTAGTTAGAACATTCGAAAAATTTCATTAACCCAGCTCTGGAAGGTC
ATCCATGAATTCTTCCTATTCCAGTGGCCTTTT-
AAGTGTAGATGTAGATACACTGTAATTTAAAACAGATGGGCATGTCTGAATATTTTCATATGCAAAATGGGAC
AGATGTATATGAATGGATAAATTATCCTTTTAAAAAGATAATTTGTAGCACGAAATATCACCAATGATTGGAA
CAGTGAACTTGCATTCAGTTTAGGGCAAACACCAGGAAGACATTATCAAAACATCTAA-
CCATCTACAAATTAATTCCAGTTAGTCTACTTTGTCTTCATACAGAAGAAA-CTTTTCGGATTTTTCAA---
TATCTATTGGGTGCCAATCGGTATCCTTT-
GAGCTGCTAAAGCCAGCGTATCACAATAGAAGTTTACTTTTCCTTGAGAGTTGCATCATGCCCATAATCAACC
TCCTGCTCCTCATCCTACCCACCCTAATCGCCATAGCATTCCTAATGTTAACCGAACGAAAAATCCTAGGCTA
CACACAACTACGCAAAGGCCCCAACATCGTAGGCCCCTATGGCCTACTACAGCCTTTCGCCGACGCAATAAAG
CTCTTCACCAAAGAACCCCTAAAACCATCTACATCAACCACCGCCCTCTATATCATCGCCCCAACCTTAGCCC
TTACCATTGCCCTCCTACTATGAACCCCCCTCCCCATACCCAACCCCCTAATCAACCTCAACTTGGGCCTCCT
ATTTATCCTGGCTACATCTAGCCTAACCGTCTACTCCATCCTATGATCAGGATGAGCATCAAACTCTAACTAC

GCCCTAATCGGCGCACTGCGAGCAGTAGCCCAAACAATTTCATATGAAGTCACTTCGGCCATTATCTTACTAT
CAGTACTACTTATGAGCGGCTCATTTAACCTCTCCACCCTCATTACAACACAAGAGCACATCTGACTGCTCCT
ACCAACATGGCCCCTAGCTATAATATGATTTATCTCTACATTAGCAGAAACCAATCGAACCCCCTTCGACCTC
ACCGAAGGAGAGTCAGAATTAGTCTCAGGATTCAATACCGAATATGCTGCCGGCCCATTCGCCCTATTCTTCA
TAGCCGAATATGTAAACATCATTATGATAAACGCCCTAACCACCATAATCTTCCTAGGTACCACACACAACGC
CCACCGCCCAGAACTTTACACCACATGCTTCACCATCAAAACCCTACTCTTAACCTCCCTATTTCTATGAATC
CGAACAACATACCCCCGATTCCGCTACGACCAACTCATGTACCTCCTATGAAAAAACTTCTTACCACTCACCC
TAACACTACTAATATGATATATTTCTCTATCCACCATAATTGCCAGCATTCCCCCACAGACCTAAATTAATCC
CCTAGCTCAACCCATCATCTACTCCACCATTTTCGCAGGTACACTCATTACCGCATCAAGCTCACACTGATTC
CTCACCTGGGTGGGATTAGAAATAAACATACTAGCCTTCATCCCAGTTCTGACAAAAAAAATAAATCCCCGCT
CCACAGAAGCTGCTATCAAATATTTCCTCGTACAAGCAACCGCATCCATAATTCTCATAATAGCCATTCTCTC
CAACAACCTACTTTCCGGGCAGTGAACTATAGCCAACATCACCAACCAATATTCATCAACAATAATATTAATA
GCCCTGGCTATAAAACTGGGAATAGCCCCCTTTCACTTCTGGGTCCCAGAAGTCACCCAGGGAACTACCCTTA
TATCCGGCCTACTCCTCCTCACATGACAAAAACTGGCCCCTATCTCAATCATATACCAAATCTTCCCAGTGGT
AAACGTAAACATCCTCCTCGCCTTTTCAATCTTATCTATCATGGTAGGCAGCTGAGGCGGACTGAACCAAACC
CAACTACGCAAAATTCTAGCATACTCCTCAATCACCCACGTAGGCTGAATAATGGCCGTACTACCATACAACC
CAGACATCACCATCTTCAACCTAATCATCTACATCGTGCTAACAACCACCGCATTCCTAGCACTCAACCTGAA
TTCCAGCACCCACAACCCTACTATTATCTCGCTCTTGAAACAAACTAACCTGATTACTGCCCCTAATCCCATCC
ACCCTATTATCACTAGGAGGCCTACCCCCACTAACCGGATTCCTACCCAAATGACTCGTAATTGAAGAACTCA
CAAAGAACGGAACACTCATTATCCCAACTGCCATAGCCATCATCACCCTTATCAACCTATACTTCTACATACG
CCTAATCTACTCCACCTCAATCACGCTGCTTCCCACATCCAACAACGTAAAAATAAAGTGACAGTTTGAAAAC
ACAAAACCCACATTTCTCCTCCCCACACTTATGACCCTCACCACTCTCCTCCTACCAATCGCCCCACTTACAT
TCCCCACCCCATAG?TTGGCAGCATAAATAATAAAGCATGTTACTTAAGCAATTTTACATTTACTGTATTAGT
AAGTAAATGCAATGGCCACTCTTATTCTCAAACAATAAAAACTACCCAGCCTTTTCATGAAAGAT-
GATTGCAATTTCCATTGGCCTGTATATGCAATAAAAAG---
CCATAACTCATCAAAATTTTAGCTATTTTCTCTTAAGATA--
GAAGAGCATTTTGCTTGAACATTGAACGTGATATTAAATATT-
CATGAAACTGAATAGAGTTTAAGTACTAAG---
TAAAACTCTCAAATTCTTTTCAACTAGATTGAAATTCTGTTTGAACCAGGTAAAAACTGCCGCTAACCTGAAG
AGGTGTTCAAAAAC-
ATCGTAAAGAGGTTGTACAATATGTTCAAAGTGCTATAGTTATTTGGGATTGATTTTTCTGAAAAGCCTGTTC
CTCCTTTGAAATTGATTCATGATAACAGCCTCCATTTGGGTCAAACAGTATTTAACCGCTTGTAAA-
TTTTAATT-
AAACGATTTGTCACTTTGAACACATAGTTTGTCCCCAAGACCTTGATGATTACCTGAATGGCCCCTTCACTGT
GGTGGTGAAGGAGTCTTGTGATGGAATGGGAGATGTGAGTGAGAAGCATGGGAGTGGGCCAGTAGTTCCAGAA
AAGGCAGTCCGTTTTTCATTCACAATCATGAAAATTACTATTGCCCACAGCTCTCAGAATGTGAAAGTATTTG
AAGAAGCCAAACCTAACTCTGAACTGTGTTGCAAGCCATTGTGCCTTATGCTGGCAGATGAGTCTGACCACGA
GACCCTAACTGCCATCCTGAGCCCTCTCATTGCTGAGAGGGAGGCCATGAAGAGCAGTGAATTAATGCTTGAG
CTGGGAGGCATTCTCCGGACTTTCAAGTTCATCTTCAGGGGCACCGGCTATGATGAAAAACTTGTGCGGGAAG
TGGAAGGCCTCGAGGCTTCTGGCTCAGTCTACATTTGTACTCTTTGTGATGCCACCCGTCTGGAAGCCTCTCA
AAATCTTGTCTTCCACTCTATAACCAGAAGCCATGCTGAGAACCTGGAACGTTATGAGGTCTGGCGTTCCAAC
CCTTACCACGAGTCTGTGGAAGAACTGCGGGATCGGGTGAAAGGGGTCTCAGCCAAACCTTTCATTGAGACAG
TCCCTTCCATAGACGCACTCCATTGTGACATTGGCAATGCAGCTGAGTTCTACAAGATCTTCCAGCTAGAGAT
AGGGGAAGTGTATAAGAATCCCAGCGCTTCCAAAGAGGAAAGGAAAAGGTGGCAGGCCACACTGGACAAGCAT
CTCCGGAAGAAGATGAACCTCAAACCAATCATGAGGATGAATGGCAACTTTGCCAGGAAGCTCATGACCAAAG
AGACTGTGGATGCAGTTTGTGAGTTAATTCCTTCTGAGGAGAGGCACGAGGCTCTGAGGGAGCTGATGGATCT
TTACCTGAAGATGAAACCAGTATGGCGGTCATCATGCCCTGCTAAAGA--GTGCCC-
AGAATCCCTCTGCCAGTACAGTTTCAATTCACAGCGTTTTGCTGAGCTCCTTTCTACCAAGTTCAAGTATAGG
TACGAGGGAAAAATCACCAATTATTTTCACAAAA???????????TATGGCACCCGACAGTTGTCATTATGTT
TGGAAACACTGCCAGCCCATGGAGATGAGGATAAAGTGGATATTTCTCTGCTGCATCAGGGTGAGAATCTTTT
TGAACTGCACATCCACCAGGCCTTCCTGACATCTGCCGCCCTAGCGCAGGCTGGAGATACCCAACCTACCACT
TTCTGCACCTATTCTTTCTATGACTTTGAAACCCACTGTACCCCATTATCTGTGGGGCCACAGCCCCTCTATG
ACTTCACCTCCCAGTATGTGATGGAGACAGATTCGCTTTTCTTACACTACCTTCAAGAGGCTTCAGCCCGGCT
TGATCTACACCAGGCTGTGGCCAGTGAACACAACACTCTTGCTGCAGGATGGATTTGCTTTGACAGGGTGCTA

```
GAGACTGTGGAGAAAGTCCATGGTTTGGCCACACTGATTGGTAAGTGCTGTTGGCTTCCTGCAGTTCCTAAGC
ACCAATGCAGAATTTCCCAAAGCCTACAGTTGCTTTTCTGGTGGTTTCTTATTTTCTTCCTTTGTCTTTGTTC
TTGATCCTTGCCACACCATCTGTATTCTCCCTGCTTTCA-
TACTTTCTGCTGCCCAGGAGCTGGTGGAGAAGAGTTCGGGGTTCTAGAGTACTGGATGAGGCTGCGTTTCCCC
ATAAAACCCAGCCTACAGGCATGCAATAAACGAAAGAAA?????????????????????????????????
?????????????????????????????ATGGAGGGAAAACACCAAACAATGAGGTTTCAGATAAGATTTA
TGTCATGTCTATTGTTTGCAAGAACAACAAAAAGGTTACTTTTCGCTGCACAGAGAAAGACTTGGTAGGAGAT
GTTCCTGAAGGCCGATATGGTCATTCCATTAATGTGGTGTATAGCCGAGGGAAAAGTATGGGTGTTCTCTTTG
GAGGACGCTCATACATGCCTTCTACCCACAGAACCACAGAAAAATGGAATAGTGTAGCTGACTGCCTGCCCCA
TGTTTTCCTGGTGGATTTTGAATTCGGGTGTGCTACATCATACATTCTTCCAGAACTTCAGGATGGGCTATCT
TTTCACGTCTCTATTGCCAAAAATGACACCATCTATATTTAGGAGGACATTCACTTGCCAATAATATCCGGC
CTGCCAACCTGTACAGAATAAGGGTTGATCTTCCCCTGGGTAGCCCAGCTGT-
GAATTGCACAGTCTTGCCAGGAGGAATCTCTGTCTCCAGTGCA?????????????????????????????
????????????????????????????????????????????????????????????????????????
????????????????????????????????????????????????????????????????????????
????????????????????????????????????????????????????????????????????????
????????????????????????????????????????????????????????????????????????
????????????????????????????????????????????????????????????????????????
????????????????????????????????????????????????????????????????????????
????????????????????????????????????????????????????????????????????????
??????????????????????????????????????????????TGTGTCKCACCATCTTCCTACTC-
TCCACTACACTGAACCCA--CCATGATGCAGTGCTCCA-CAAACATATGTAGAATGGAAGT-----------
------------------------------------------------------------------------
------------------------------------------------------------------------
------------------------------
AAAACCTCAGGGRTAAGAAAAGATAGTTAAAGCAGATTCACTACAGTAACAAACCAGTCAAGAAATTTCACTG
AAAGTAAAGGTTT-
GGGTGACAAAATTTCCAGCTGTGTTCTAAGAAACATAAGTTCAAGCAGGAGTGCATTTAATCTGATTTTTCCT
CAAAGC--
CTCTCTAATTTGTAGGGAAGGACATTCATTTTCATTAACTTCATGTGAGCTAGAGAGAATCTGTGATTGAGGC
TCTAGAACCCCTGGTAAACATGCCAGCAAGCAACGGCACCTCCCCTGGAGGTTCAACCTAGAATACAAGACTG
TCAGCATTGGTAAGGAGCCTTGAACTCTAAACCCATAGGCTCAAGTCAGGAA-
CTCAGGAGGCCTATCAACACCTGGATACAATTTTGCCACCTTAACACTTTATTCCCTTTACTTGTGCCAAACT
????????????????????????????????????????????????????????????????????????
????????????????????????????????????????????????????????????????????????
????????????????????????????????????????????????????????????????????????
????????????????????????????????????????????????????????????????????????
????????????????????????????????????????????????????????????????????????
?????????????????????????????????AGGCCCTCAAAGATCTTTATTGCCATAAAGTAGAGTAAC
T-GTCTTTGACTTGGACCTTGTTTACTGTGAACTCTAATC-AAGGCAGGAGATGCAGCAGTTCTACTTA-
TTGCCATGTGGACTTGTGGAAAGGACACGTGTGACCCTTAAGAATCCAGTTTGGATTAGTGTTTGAAATTGAA
TTGGGAATGTTGTTCCAGGATGTGGAATGCAGCCGTGATCACACTT----ACC-
GAGCTTACTTTGATCTGTTTGTCAATAGCATGCAAAAAA-
TGCTTTGTTTGCCCTTTGCTTCTGCTTTTTTCAGGGAAGCTGCCAAAGAATGTCGACGTCGAAAGAAAGAATA
TGTAAAATGTCTGGAGAGCCGAGTTGCAGTGCTGGAAGTCCAGAACAAGAAGCTTATAGAGGAGCTTGAAACC
TTGAAAGACATT????????????????????????????????????????????????????????????
????????????????????????????????????????????????????????????????????????
????????????????????????????????????????????????????????????????????????
????????????????????????????????????????????????????????????????????????
????????????????????????????????????????????????????????????????????????
????????????????????????????????????????????????????????????????????????
????????????????????????????????????????????????????????????????????????
????????????????????????????????????????????????????????????????????????
????????????????????????????????????????????????????????????????????????
```

??TGTTTATATCTAAATGTATGGATTCCA
GCACCTAAACCAAAAAATGCCACTGTACTGATATGG-
ATTTATGGTGGTGGTTTTCAAACTGGAACATCATCTTTACATGTTTATGATGGCAAGTTTCTGGCTCGGGTTG
ARAGAGTTATTGTAGTGTCAATGAACTATAGGGTGGGTGCCCTAGGATTCTTAGCTTTGCCAGGAAATCCTGA
GGCTCCAGGGAACATGGGTTTATTTGATCAACAGTTGGCTCTTCAGTGGGTTCAAAAAAATATAGCAGCCTTT
GGCGGAAATCCTAAAAGTGTAACTCTCTTTGGAGAAAGTGCAGGAGCAGCTTCAGTTAGCCTGCATTTGCTTT
CTCCTGGAAGCCATTCGTTGTTCACCAGAGCCATTCTGCAAAGTGGATCATCTAATGCTCCTTGGGCAGTAAC
ATCTCTTTATGAAGCTAGGAACAGAACGTTGACCTTAACTAAATTGACTGGTTGCTCTAGAGAGAATGAGACT
GAAATAATCAAGTGTCTTAGAAATAAAGATCCCCAGGAAATTCTTCTGAATGAAGCATYTGTGGTCCCCTATG
GGACACTTTTGTCAGTAAACTTTGGTCCAACCGTGGATGGTGATTTTCTCACTGACATGCCAGACATATTAAT
TGAACTTGGACAATTTAAAAAAACCCAGATTTTAGTGGGTGTTAATAAAGATGAAGGGACAGCTTTTTTAGTC
TATGGTGCTCCTGGCTTCAGCAAAGATAACAATAGTATCATAACTAGAAAAGAATTTCAGGAAGGTTTAAAAA
TATTTTTTCCAGGAGTGAGTGAGTTTGGAAAGGAATCCATCCTTTTTCATTACACAGACTGGGTAGATGATGA
GAGACCTGAAAACTACCGTGAGGCCTTGGATGATGTTGTTGGGGATTATAATATCATATGCCCTGCCTTGGAG
TTCACCAAGAAGTTCTCAGAATGGGGAAATAATGCCTTTTTCTACTATTTTGAACACCGTTGTGCTAATTGTG
AGAAAATATGCTACTTTGATCAAACTAGAATGAAGGAAATAAAAAGACACTGTGGCTCAACTTGTAATAAAGA
GCAGCTCATCCTCACAGCTTTGTGATTGTTGCTATAGTTATCT---
ATTCAACATGGGATGCACAATACAGTTAACGTTTCATTACCAGTAGCCTGTTGCGTGCTTATTGTTAAAAGGA
ATTTAACATACAAGTTTTTGTAAGGGTTGTTCGCCTAATTCTGATTCCTCGTGCTGACTGGCATGAGACAGGG
AGCTGGATTGTATTAAACTGGTTTTATTGTGATGTGACTGTGATGGATGTGACAAGGGCCTCTATTATTCGCT
GGGGAGTGTGGCCGTTAATGAAGCCATTAGCTGAGCTAAGGCAAGTGAAGAAAATAGGGAAATTACCACTATT
CTGAAGCCATTAAGTTGTAATCCACCACTTTCTCTCCTTACTCAAAAAGGCATCTTCTTTTTAAGGCCTC???
???
???????????CCTACCCAGGTGTGCGGACCCATGGGACTCTGGAGAGCGTGAATGGGCCCAAGGCGGGTTC
AAGAGGCCTGACAT---
CGTTGGCTGACACTTTCGAACATGTGATAGAAGAGCTGTTGGATGAGGACCAGAAAGTTCGGCCCAGTGAAGA
AAACAATAAGGACGCAGACTTGTACACGTCCAGGGTGATGCTCAGTAGTCAAGTGCCTTTGGAGCCTCCTCTT
CTCTTTCTGCTGGAGGAATACAAAAATTACCTAGATGCTGCAAACATGTCCATGAGGGTCCGGCGCCACTCTG
ACCCTGCCCGCCGAGGGGAGCTGAGCGTGTGTGACAGTATTAGYGAGTGGGTAACGGCAGCAGACAAAAAGAC
TGC??
???
??????????GAACGGCTATGAAAATCCAACCTACAAGTTCTTTGAGCAGATGCAGAACTAGACCCCC-
GCCACAGCAGCCTCTGAAGTTGGACAGCAAAACCATTGCTTCACTACCCATCGGTGTCCATTTATAGAATAAC
GTGGGAAGAAACAAACCCCT--
GTTTTATGATTTACTCATTATCACCTTTTGACAGCTGTGCTGTAACACAAGTAGATGCCTGAACTTGAATTAA
TCCACAAATCAGTAATGTATT----CTATCTCTCT----TT-----------------
ACATTTTGGTCTCTACACTACATTATTAATGGGTTTTGTGTACTGTAAAGAATTTAGCTGTATC-AAAC-
TAGTGCATGA-
ATAGATTCTCTCCTGATTATTTATCACATAGCCCCTTAGCCAGTTGTATATTATTCTTGTGGTTTGTGACCCA
ACTAAGTCCTACTTTACATATGCTTTAA-GAATCGATGGGGGA---
TGCTTCATGTGAACGTGGGAGTTCAGCTGCTTCTCTTGCCTAAGTATTCCTTTCCT-
GATCACTATGCATTTTAAAGTTAAA-CGTTTTTAAGTATTTCAGATGCTTCAGAGAGATTTTTTTTC--
CATGATTGCATTTTACTGTACAGATTGCTGCTTCTGCTATATTTGTGATATAGGAATTAAGAGGATACACGTT
TGT??????????????????????????????TCACTTCCGGAGAGCACTAAGCCACTTCCAGCCCTGCTGTG
AACCAGGACCGTGGGAGCCATCTTTTTCATGTGGTCGGCTTTGGAAGCATCTACATCCACCACTTTAGAAGAC
AAGTCAAGTGGCTTATCTGTGACGTCTTTGGTAACGGTCTGCTTCTCCAACAGAGGAGGTGAGCTGCCGTCTT
TTCTGTCTTGAACCGCTGTCTTCCGGGCATGCCCAGGCACTGGCTGGGCACCTTCGCCCCCTTCCGGAGCCTT
GGGATACTTGCCATTGGAGAGCCTGGCCGCGGGGAACTCGCTGCTAACTGTCATGTACGGCTTCGACAGGGCA
ACTGAAGGAGAGGTGGAGATCCTGGCATASTGCTTGTGGAACTCCGAGTAGGTGTCTGCAGCAGGCTGGGTGG
GAAGGTGGACTCGGGGTGACGGCCGAGGTGAAGGGGGCAACAGGAGAGCTGTGTCCCCCGACAGGCCACTGGT
GACCGCCTTGGCAGAGGGAACCCTGGGCTGCTTACTGTTCTGGATGTGAGGATAGGTGTGGGAATCAACGGGA
TTCCCAGGGCTGACGCCCATCTTCCACGGGAGGCTTTTGTCTGCGCAATGGACGAGAGGCGGGATGGCTGGGG
AGGCCGAAGGTGTCGAGAGCCTCATGGGTGATGCCAAGGACGATGGGATGTGGGGACCGACGTAGTGAGGTGG
CGGCAGGTAGAGAAAGCGCTCCCCATTGGTGCAGACTGGAGAATACAGCGGCTGGGCC???????????????

??????????????GCCAGGACCTTTGTAATTCAAGTGCTTTTTACAGTAGTAGCAAATGTTACATAAGCCCA
AACATGGAGCA---
TATTTGGAGTTGGTGGGCAGACCCAAATCATGATGCAAGCCTGCAGTAGCAAGTGTGCAATGAAGCACAACCA
AATTCTGTGAATGGAAGGTAGCAAGACGCCTCTCCAAAGCAGCCCTTCTGCGCAGGAGCTGAAAGCTCCACTG
GGTAAGTTTTCTGAGGCAGTTTTCAGAGGCTGGGCTTGGCTCAGGGCCCAGCTCTGGATGAGCCTGTGGACGG
TGGGGT----
AGGTCTTTGCTAACCTTACATAGAACCAAGCAGATTTGAGAAGAGTTTATGCAGGCCTCTTTCCTCCTGGCGT
TTGTCTTGCCCTGCCAGCAGGCCAGGAGGG-
TTCCGCTGCTCAAGTAGTGGAGGGAGGGAGCCTGCAGAACTAGGGGATCAGCGTGGGTAATCGCAGCCACAGG
GATTAGCTGCTGCAAGCTGTCCACAGAAGCCATTCTGAAATGTCATTTGAGTGATCTGGCCGTCTACTGGAGG
AGGCCCTCGGGGCAGCTGTCTCCAGGAGCAGCTTCTGAGGGAGTCTTCCGGGAGGAGCCGCGTCTCTGCTGTC
GTCCTCGTCCATGTCCTGGTCACACTTCCATTCCTCATCTTCATTTAAAGTCGGCAGGTATCCAGACATGCCC
TTCCCTGTCCCTGACCCAGAGCTCTGGTCACTACAGACTTTCGGGCTCTCCGAGCCTGTCCTCGTATATTCCA
AATAAATATCAGACTTAAGGAAGGAGGGATAGGTGTTTTCCTCCATAGTGGCCTGGATTTCTGTCTGGGCCTG
GTCAAACATGGCAGGATCGATCAGCTGCTTCATGATGCAGCCCTTTATGAAGCTCTTGGTGGCTGGCTTGGTC
TGCCGGGACACAATGCCATTGTTATCAAGAATGTACTTTCGGTAGATGGCTCTCGCCAGCTTCAGCCTCTTCT
CCTCGTTCGAGTCACAGGGCTCCAGCTTCCTGAAGCCAGTGCAGGCAAACCAGAAGTCCAGCAAGTCGGCACA
GCCCTCCTGCTTCAGGAAAGTCCTGAACAGGCTTATCCCATCTTGGTCATCCAGCAGGGAGTGCAGTGACTCA
GCCCACTTCAAGTATGGTGGGGTGGGGGAGGCACTGCCCTCAGGCTCATACCCCAGGTCCAGATCCGAGCGCC
TGGGAGTGGCCGTCGAAGTCTCACCTTTAATGCCAACGCCTTTCCCGGGGCAGAAACTGTAGCTGGCGGGCCT
CGGGTCTGCGGACACCAGTTCTCCCTCCTCACCAGGCACTGGGGGTCGGGGAGCATCTTCGGTGAAACTTGCT
CCGAGGTCCAAGGGGAAACCCTGCTCTTGGATATTCATTTGGGACTCTG--
CGTCAAGGAACAATGAGCGCTGCACCCTAATACATCAGTACTTA-
CAGGTCCAAAGTGAATCAATCTGTCCTGTTGAAACCAT???????????????????????????????????
???
???
???
????????????????????????????????????GGAAAAAGAATTAAAATCCTCTGGTKRTTTT-----
-----AGTGAAAAGG--AAAACAAATTTCTAAAATGTTAT-CTAATTTTAAGTGAACAAAAT-----------
TACATGGATCTACTTACACCAGCATAAAGCAGGTATARTTTACAGGTAAGTGGAAGAAGTACCTAGGTTATTT
ATAGGACAGATTACTATCCTATGTTTTGAATGCTTT--------GAAGTTTGATT-
AAGCACTCATAGTGCTTTCTTTTCCTTTCTTAGCAATAATTATTGGCCCTGATGGTCATCCTTTGACTGTCTA
TCCTTGCATGATTTGTGGGAAGAAGTTTAAGTCGAGAGGTTTTTTGAAAAGACACATGAAAAACCATCCTGAA
CACCTTGCCAAGAAGAAGTACCACTGTACTGACTGTGATTACACTACCAACAAGAAGATAAGTTTACATAACC
ACCTGGAGAGCCACAAGCTGACCAGCAAGGCAGAGAAGGCCATCGAATGTGATGAGTGTGGGAAGCATTTTTC
TCATGCGGGGGCTTTGTTTACTCACAAAATGGTTCACAAGGAAAAAGGGGCCAACAAAATGCACAAGTGTAAA
TTCTGTGAATACGAGACAGCTGAACAGGGGKTWWTGAATCGCCACCTCTKGGCCKYSCACAGCAAGAACTTTC
CTCATA???????????????????????????????????TAAAACCCCTTCTGCAGTTCACACTAGCAC
CAGCCACAACCCTGGACTTCCTCCTAATTTTAACGAATGGTACGTCTGAGGACAAACACAAACCCTGTTAACT
ATAGAATGGACCAAATGCATTTTTAAAAGAAAACTGAGACCAATCAGATGGAAATGGAGTTTTAAGGCAAGAG
GCCATATATAGGGCTACATCTTGTTAATTGCAATTGTCCAGGAAGGTTTTGGGCAAGATCCAAAAGTAGCCAT
GCCCTTTTCTCAGGAT---AGAAAATGTTTTGGCATTTGAAGCATTTTTT--
ACAAAATCTTTACACTACTTTTTCTTCCCCTTCCTCTTGCTCTCTGCACACCCCATTCTTAAACTCCTGCAAT
TCATTTTAACACTTGTCCTGTTTCTTGAGAGGAAGTTATAGAAGGCTTGTTGGTGGTGGTGATGTTAAACTGA
TGGAAATTCTTTTTCGCCTTAGTGGTGATTGTTTAAACTCTCACAGTCTTAAACCGTGCCAAAGTCCTGTTAT
A
Lagothrix
???TTCCTTTCATGCCG
GTTCCCTT-
CCGTCATGAGCATGGACTACATGGTATACTTCAGCTTCTTCACCTGGATTTTCATTCCCCTGGTCATCATGTG
TGC--AATCTATCTTGACATCTTTTACAT-
TATCCGGAACAAACTCAGTCAGAACTTCTCTAACTCCAAAGAGACAGGTGCATT---
TTATGGACGGGAGTTCAAGACGGCAAAGTCCCTGTTTCTGGTGCTTTTCTTGTTTGCTTTGTCATGGCTGCCT
TTGTCCATCATCAACTGCATCACCTACTTTCA--------------

TGGTGAGGTCCCACAGCTTGTGCTATACTTGGGCATCCTGCTGTCCCATGCCAACTCCATGATGAACCCTATC
?????????????????????ATGTTCATAAATCGCTGACTATTTTCAACCAACCACAAAGACATCGGAACAC
TATACCTACTATTCGGCGCATGAGCAGGGGCCGTAGGAACAGCCCTAAGTCTCTTAATTCGAGCGGAACTAGG
TCAACCCGGAAGTTTAATAGAAGACGACCATGTTTATAATGTTATCGTTACCTCCCACGCATTTATCATAATC
TTCTTCATAGT---TATACCGATTATAATTGGAGGCTTTGG---
AAACTGACTTGTCCCCCTAATAATTGGTGCCCCCGACATAGCATTCCCCCGAATAAATAATATAAGCTTCTGA
CTCCTACCTCCA--TCCCTACTACTTCTATTAG-
CATCCTCGACCTTAGAAGCCGGTGCTGGAACTGGCTGAACAGTTTACCCACCCCTAGCAGGAAATATATCACA
CCCAGGAGCTTCTGTAGATCTAACTATCTTTTCACTACACCTGGCAGGTATTTCTTCCATTCTAGGGGCCATT
AACTTCATCACAACAATTATTAATATAAAGCCCCCAGCTATAACCCAATATCAAACACCCCTGTTTGTCTGAT
CCGTCCTTATCACAGCAGTTCTGCTACTTCTATCCCTTCCAGTCTTAGCCGCCGGAATTACCATACTACTGAC
TGATC--
GTAACCTCAACACTACCTTTTTCGACCCCGCCGGTGGTGGAGACCCAGTCTTATATCAACACCTATTCTGATT
TTTTGGTCACCCTGAAGTATATATTCTTATCCTACCTGGCTTCGGAATAATCTCACATATCGTAACATATTAC
TCCAACAAAAAAGAACCATTCGGGTACATGGGAATGGTATGAGCTATGATATCTATCGGTTTCCTAGGATTTA
TCGTATGAGCTCACCATATATT-
TACAGTAGGAATAGATGTAGACACACGTGCATACTTCACATCAGCTACCATAATTATTGCCATCCCCACCGGA
GTAAAAGTATTTAGTTGATTGGCCACACTGCATGGTGGCAATATTAAATGATCCCCTGCAATACTATGAGCCC
TAGGCTTTATTTTTCTCTTTACCGTAGGAGGACTCACAGGAATTGTACTAGCCAACTCATCATTAGACAT-
TGTCCTACACGACACATACTACGTAGTAGCCCACTTTCACTATGTATTATCTATGGGGGCCG-
TGTTTGCTATCATAGGGGGCTTTATCCACTGATTCCCACTATTCTCAGGTTATACACTTAACCACACTTACGC
TAAAATTCATTT-
TACCGTTATATTTGTAGGTGTAAACATAACCTTCTTTCCACAACACTTCCTCGGTTTATCTGGAATACCCCGA
CGATACTCAGATTACCCAGATGCGTACACTACATGAAATATCATCTCATCCGTAGGCTCATTTATTTCATTAA
CAGCAGTAATCCTAATAATTTTTATAATCTGAGAGGCCTTTTCCTCAAAACGGAAAGTCCTAGCCATTGAGCA
ACTAACCACTAACCTAGAGTGACTCTATGGATGTCCCCCTCTTTATCACACATTTGAAGAATCCACATATGTA
AAAGCTCTAAGCGAAAAAAGG???????????????ATGGCCCATCCAGCCCAACTAGGTTTACAAAACGCTA
CATCTCCAATCATAGAGGAACTTATCGCTTTCCATGACCATGCCCTTATAATTATTTTCCTAATTAGCTCACT
AGTATTATATATTATCTCCCTAATACTAACTACAAAATTAACCCACACCAGTACCATAAATGCTCAAGAAATT
GAAATAGTCTGAACCATCCTACCTGCAATTATCCTAATTATGATTGCCCTCCCATCCTTACGTATCCTGTACA
TGACAGACGAATTTAACAAACCCTACTTAACACTTAAAGCAATTGGCCACCAATGATACTGAAGCTACGAATA
TTCAGACTATGTAGACTTAGCCTTCGACTCCTATATTACACCCACATACTTTCTTGAACCAGGTGAATTTCGA
CTTCTTGAAGTAGACAATCGAACAACTCTTCCAATAGAAGCAGATATCCGTATGTTAATTTCATCCCAAGATG
TCTTACACTCATGAGCTGTTCCATCACTAGGTGTCAAAACAGATGCAATCCCCGGACGCCTAAACCAAGCCAT
ATTAGCCTCTATACGACCTGGCCTGTTTTATGGACAATGCTCAGAAATCTGCGGGTCAAACCACAGCTTTATA
CCCATCGTCCTAGAATTTATCTACTTCCAAGACTTCGAAGTATGAGCCTCATACTTATACATCGTAT??????
ATGACCACCCCTCGCAAAACACACCCACTAGCAAAAATCATCAACAACTCACTCATTGACCTACCCTCACCAT
CCAATATTTCTGCTTGATGAAATTTTGGTTCACTCTTAGGCATTTGTTTAATTATTCAAATCGCCACAGGCCT
ATTCCTAGCTATACACTATACACCAGACACTTCAACCGCCTTCTCTTCAGTTGCCCATATTGCCCGAGACGTA
AACTACGGATGAATAATCCGCTACCTACACGCCAACGGTGCTTCCATATTCTTTATTTGCCTCTTCCTACACG
TCGGCCGAGGCTTATATTATGGATCCTTCCTTTCTCTGGAGACTTGAAACGTAGGTATTATCCTACTACTTAC
AACTATAGCCACAGCATTCATAGGTTACGTCCTCCCATGGGGCCAAATATCATTCTGAGGGGCTACAGTCATC
ACAAACCTTCTATCAGCCATCCCCTATATCGGGTCCAGTCTTGTAGAGTGAATCTGAGGTGGTTTCTCAGTAG
ACAAAGCCACCCTTACACGATTCTTTACTTTCCACTTTATCTTACCCTTTATTATTGCAGCCCTAGCAACTAT
TCACCTATTATTTCTGCATGACACAGGGTCAAGTAATCCATCAGGAATAACATCAGACCCAGATAAAATCACA
TTTCACCCCTACTATACAATCAAGGACATTTTTGGTTTAATTCTTCTTCTCCTATGCTTAATAAACCTAACCT
TATTCTCACCTGACCTCTTAACCGACCCGGACAATTATACATTAGCCAACCCTCTTAACACCCCACCACATAT
CAAACCAGAATGATATTTCCTATTCGCATACGCAATCTTACGGTCTATCCCTAATAAGCTTGGAGGCGTCCTG
GCCCTAGTACTATCTATCCTAATTTTAGTAGCCTTTCCTACCCTACATCTATCCAAACAACAAAGCATAAAGT
TCCGACCCATCGCCCAAATTCTATTCTGAGCCTTAGTGGCTGACCTAATCACACTCACATGAATTGGAGGCCA
ACCAGTCGAATACCCTTTCGTAACCATCGGCCAAACCGCATCAGTCATATACTTCCTAATTATTATTACGCTC
ATCCCCCTCTCTACCTTAATTGAAAACAAACTACTTAAATGGTAA?TTAGGTTAAATTTATTCAAGGGAATGA
GAATTTATGGGTATAACTGTTGGCTGGAAAGCCCTTCCTCACCTCCAATCCTCCTTTCAAACGACATTTCC-
TCCTGAAATTTCTCTGTGGGATGAAATGTTCAATTATTAGTCAATCAGTC----

TCTTACACGTTATCTGGCTTTACATAG----
GCCCTTGATAGTTCTTGGAAAATAATCCAATATTGTGTTGTTTTGGTGTCTTTGTTTTGGAAATGGAAGCATT
TTGTATGAATCACCTCTTTAACTGTGAGGAGTTGAATTGTCTTAGTTCTGCTTGCCTAATGACCRGAAATAAA
--GACATCACAGTCTCTAACCCCACTTTTT-
GGTGGAAAGAAAATCTAAAAGTAGCAACATTTAATGGAACAAAATATCAGTAATCAAAGAAACTCCCCTGTGA
AACCGTAATACTATTTT-
AAAATCAACATTATTCTAAGGTAGCTGAAATGTGTGATTTTTCTATTTTGCCTTTTCACTGACAATATACCCA
TAAATCT-
TGGTGGTGATTTTCTCTCCAAAGTTAATTTCTGGGCCATTGGTGACTGCACTATCTATTGTTCTAAATTGTTG
TCTTAATTGCATTCTTTAAAAATGTAATGTTGAAGGCACATAATATTGAAGATGACTCCAGGATGAACCCTGA
GTTCGCAGACCGAA-
TAAAACAGCTCGATAAAGAGGCGTCTTACTACCGTGATGAGTGTGGCAAGGCCCAAGCGGAAGTGGACCGGTT
GCTGGAGA??????????GAAGAGCTGCATCAGGCAAGTACTTTGATCTTTCTTGGAGCAGGTTCCTTCAAAA
CGCTTCTCCAGAGGACGGAAGTCCCTCCAGTCTGGCTCGCTGCTCGCAGTGGTGGGGAAAGCTGAGGTAATTC
CCCGGTGGGAAATCTGCGAGGCCCCTGATACCCCTG--------
CCACCAGGCCCATCACTGACGGGCCTAAGGGCCTCATCTGATACTCTAAGACGGGCTTCTCCTGTCGGATCTG
GGCCTCTCGGACTTGAGCCTCTCTAGAACGAGCCTCTCGGGCCTGGGCATCTCGGGTTCTGGCCTCCCTGGCA
TGACCTTCCCAAGTGTGGGCCTCCCTG--
GCACAGGCTTCCTTGCCATGAGCCTCTCGAGTATAGGCCTCCCTGGTGTGGGCCTCCCTGGCATGAGCTTCTC
GGGCACGTGCCTCCCGGGCCTCAAGCTGCTCCCGCCGAAGCTCCCAATACAACAACTGTTTCTGGATGGTCA-
CTAGCCGTTCCTCCTCTGTCTCCATTGCCCCAGGTGGCCGGGAGGAAGAAAAGGGCTCAAAGTTCAAGAAGGG
GTCAAACATCTCAGAGCTTCCACCATGGAGGTCATAAAGGCAGTCATCCCCAGGTGGAGAGTTCTCAAGACTG
TCATCTGGCTCATAGAACTCATATAGGGCATCTCCACTGTAGCTGTCTCGGGGCAGACAATCCCTGCGGACAA
GCCCCAGGGCCTCACCTGAATCATCCTCAA???
???????????????TACAGTGAATGTTCTCCACTGTTGTGGTAGGCATGTTTCATGTGTGGGGATGTTGGTG
TGCAAGTTTCATGTGTTTGGAAAACACTAGCTAGAACATTCGAAAAATTTCATTAACCCAGCTCTGGAAGGTC
ATCTATGAATTCTTCCTATTCCAGTGGCCTTTT-
AAGTGTAGATGTAGATACACTGTAATTTAAAACAGATGGGCATGTCTGAATATTTTCATATGCAAAATGGGAC
AGATGTATATGAATGGATAAATTATCCTTTTAAAAAGATACTTTGTAGCACGAAATATCACCAATGACTGGAA
CAGTGAACCTGCATTCAGTCTAGGGCAAACACCAGGAAGACATTATCAAAACATCTAA-
CTATCTATAAACTAATTCCAATTAGTCTACTTTGTCTTCATACAGAAAAAA---TTTTTGATTTTTAAA---
TATGTATTGGGTGCCAATCCGTATCGTTT-
GAGCTGCTAAAGCCAGCATATCACAATAGAAGTTTACTTTTCCTTGAGAGTTGCAT?GTGTTTATATTAAATC
TACTACTACTAGTCATCCCTGCCCTAATTGCTATAGCATTTTTAACACTCATAGAACGAAAAATCTTAGGCTA
CATACAATTTCGCAAAGGTCCTAATATTGTAGGCCCCTACGGCATACTACAGCCAATTGCTGATGCAATAAAA
CTCTTCACAAAAGAACCCCTACTACCCACCACATCCGCCACAACCCTGTACATAATTGCCCCAACCCTAGCTC
TATCCATCGCTCTCCTACTATGAAGCCCCCTTCCAATACCACACCCTCTAATCAATTTCAACCTAGGCCTCCT
ATTTATACTAGCAACATCAAGCCTAGCCGTATATTCAATCCTATGATCTGGATGAGCTTCCAACTCAAACTAC
GCACTAATTGGCGCATTACGAGCAGTAGCCCAAACAATCTCATATGAAGTCACCCTAGCTATTATCCTGCTAT
CAACCCTATTAATGAGTGGCTCATTCAATCTGCATTCACTCATCACAACACAAGAACACTCCTGGCTTTTATT
GCCATCATGACCTCTAGCAATAATATGATTCATTTCTACACTAGCAGAAACCAACCGAGCTCCATTCGATTTA
ACAGAAGGCGAGTCAGAACTAGTTTCAGGATTCAACATTGAATATGCTGCAGGTTCATTCGCTCTATTCTTTA
TAGCAGAATATATAAATATTATTATAATAAACGCCCTAACTACCACTATCTTTTTAGCCACACCCTACAACAT
AACTACATCAGAACTCTATACCATAAGTTTCATAATCAAAACCCTTCTATTAACTAGCCTATTTTTATGAATT
CGAACAGCATACCCTCGATTCCGCTACGATCAACTTATGCATCTCCTATGAAAAAACTTCTTGCCACTCACAC
TGGCACTATGTATATGATATATTTCAATACCAGCTCTAACATCCGGCATCCCACCCCAAACATA?ATCAACCC
TCTAGCCTACCTTATTATTTCCTTCACTATTATAATGGGAACCATAATTACAGTCCTAAGCTCACATTGATTC
CTCATCTGAATGGGCCTAGAACTAAATATGCTAGCTATCGTACCTATACTAATCAAAAACACAAGTCCCCGCT
CTACGGAAGCAGCCACCAAATATTTTCTAACACAAGCAACCGCATCCATGCTATTACTAATAGCCATTTGCCT
AAATAACCTACTCTCCGGACAATGAACAATTAATCCATCCCTTAACCAAGTTCTTGCTACAATAATGATAATT
GCCCTGATAATAAAACTAGGAATAGCCCCCCTACACTTCTGACTTCCAGAGGTAACCCAAGGCACCCCCCTAA
TCCCCGCTATACTCATTCTTACATGACAAAAACTAGCCCCCTTATCAATCATATTACAAATCTTCCCATCAAT
TAACATCTATGTACTATTGATAGCCTCAATTTTATCTATCATAGTCGGAAGCTGAGGGGGACTAAACCAAACA
CAACTACGCAAAATCCTAGCTTACTCATCAATTACCCACATAGGTTGAATGATAGCAGTACTATATTATAACC

CAAGCATCACCACCCTAACCTTACTCATCTATATTTTCCTAACAATCTCCACATTTATAACTTTCTACCTAAA
CTCAAACATAACAACACTATCACTATCACACACCTGAAACAAAATCACATGAATAATACCTATAATCCCACTA
ATAATAATATCGCTAGGAGGCTTACCCCCACTAACAGGCTTCTCCCCCAAATTCGCCATTATACAAGAACTCA
CAAAAAATAATAACCTCATCATACCTCTCACCATAGCTTTGTTAACACTAATCAACCTATACTTTTATATACG
CTTAACATACGCCATCTCAATAACAATATTCCCCACATCCAACAACACAAAAATCAACTGACAACTAAAACAT
ATAAAACCCGCACCACTTCTCCCCCCACTTATAGTATCCTCCATCCTTCTATTACCTATAACCCCATTAATAC
TAATAGCCT?????GTTGGCAGCATAAATAATAAAGCATGTTACTTAAGCAATTTTACATTTACTGTATTAGT
AAGTAAATGCAATGGCCACTCTTATTCTCAAACAATGAAAACTACCCAGCCTTTTCATGAAAGAT-
GATTGCAATTTCCATTGGCCTGTATATGCAATAAAAAG---
CCGTAACTCATCAAAATTTTAGCTATTTTCTCTTAAGATA--
GAAGAGCATTTTGCTTGAACATTGAACGTGACATTAAATATT-
CATGAAACTGAATAGAGTTTAAGTACTAAG---
TAAAACTCTCAAATTCTTTTCAACTAGATTGAAATTCTGTTTGAACCAGGTAAAAACTACCGCTAAGCTGAAG
AGGTGTTCCAAAAC-
ATCGTAAAGAGATTGTACAATACGTTCAAAGTGCTACAGTTATTTGGGATAGATTTTTCTGAAAAGCCTGTTC
CTCCTTTGAAATTGATTCATGATAACAGCCTCCATTTGGGTCAAACAGTATTTAACCGCTTGTAAA-
TTTTAATT-
AAATGATTTGTCACTTTGAACACATAGTTTGTCCCCAAGACCTTGAGGATTACTTGAATGGGCCCTTCACTGT
GGTTGTGAAGGAGTCTTGTGATGGAATGGGAGATGTGAGTGAGAAGCATGGGAGTGGGCCAGTAGTCCCAGAA
AAGGCAGTCCGTTTTTCATTCACAATCATGAAAATTACTATAGCCCACAACTCTCAGAACCTGAAAGTGTTTG
AAGAAACCAAACCTAACTCTGAACTGTGTTGCAAGCCATTGTGCCTTATGCTGGCAGATGAGTCTGACCACGA
GACCCTGACTGCCATCCTGAGTCCTCTCATTGCCGAGAGGGAGGCCATGAAGAGCAGTGAATTAATGCTTGAG
ATGGGAGGCATTCTCCGGACTTTCAAGTTCATCTTCAGGGGCACTGGATATGATGAAAAACTTGTACGAGAAG
TGGAAGGCCTTGAGGCTTCTGGCTCAGTCTACATCTGTACTCTTTGTGATGCCACCCGTCTGGAAGCCTCTCA
AAATCTTGTCTTCCACTCTATAACCAGAAGCCATGCCGAGAACCTGGAACGTTATGAGGTCTGGCGTTCCAAC
CCTTACCATGAGTCTGTGGAAGAACTGCGGGATCGGGTGAAAGGGGTCTCAGCCAAACCTTTCATTGAGACAG
TCCCTTCCATAGACGCACTCCACTGTGACATTGGCAATGCAGCTGAGTTCTACAAGATCTTCCAGCTAGAGAT
AGGGGAGGTGTACAAGAATCCAAATGCTTCCAAAGAGGAAAGGAAAAGGTGGCAGGCCACACTGGACAAGCAT
CTCCGGAAGAAGATGAACCTCAAACCAATCATGAGGATGAATGGCAACTTTGCCAGGAAGCTCATGACCAAAG
AGACTGTGGATGCGGTTTGTGAGTTAATTCCTTCTGAGGAGAGGCACGAGGCTCTGAGGGAGCTGATGGATCT
TTACCTAAAGATGAAACCAGTATGGCGATCATCATGCCCTGCTAAAGA--GTGCCC-
AGAATCCCTCTGCCAGTACAGTTTCAATTCACAGCGTTTTGCTGAGCTCCTTTCTACCAAGTTCAAGTATAGG
TATGAGGGGAAAATCACCAATTATTTTCACAAAATAGATGTTGCTTATGGCACCCGACAGTTGTCATTATGTT
TGGAAACACTGCCGGCCCATGGAGATGAGGATAAAGTGGACATTTCTCTGCTGCATCAGGGTGAGAATCTTTT
TGAACTGCACATCCACCAGGCCTTCCTGACATCTGCCGCCCTGGCTCAGGCTGGAGACACCCAACCTACCACT
TTCTGCACCTATTCTTTCTATGACTTTGAAACCCACTGTACCCCATTATCTGTGGGGCCACAGCCCCTCTACG
ACTTCACCTCCCAGTATGTGGTGGAGACAGACTCCCTTTTCTTACACTACCTTCAAGGGGCTTCAGCCCGGCT
TGATCTCCACCAGGCTGTGGCCAGTGAGCACAACACTCTTGCTGCAGGATGGATTTGCTTTGACAGGGTGCTA
GAGACTGTGGAGAAAGTCCATGGCTTGGCCACACTGATGGGTAAGTGCTGTCGGCTTCCCGCAGCTCCTA-
GCACCAATGCAGAATTTCCGAAACTCGATAGCTGCTTTTCTGGTCGTTTCTTATTTCTTCCTTTTTCTTTGT
TCTTGATCCTTGCCACACCGTCTGTATTCTCCATGCTTTCA-
TACTTGCTGCTGCCCAGGAGCTGGTGGAGAAGAGTTTGGGGTTCTAGAGTACTGGATGAGGCTGCGTTTCCCA
ATAAAACCCAGCCTACAGGCATGCAATAAACGAAAGAAAGCCCAGGTA??????????????????????????
??AAACACCAAACAATGAGCTTTCAGATAAGATTTA
TGTCATGTCTGTTGTTTCCAAGAACAACAAGAAGGTTACTTTTCGCTGCACAGAGAAAGACTTGGTAGGAGAT
GTTCCTGAAGCCAGATATGGTCATTCCATTAATGTGGTATATAGCCGAGGGAAAAGTATGGGTGTTCTTTTTG
GAGGACGCTCATACATGCCTTCTACCCACAGAACCACAGAAAAATGGAATAGTGTAGCTGACTGCCTGCCCCA
TGTTTTCCTGGTGGATTTTGAATTTGGGTGTGCTACATCATACATTCTTCCAGAACTTCAGGATGGGCTATCT
TTTCATGTCTCTATTGCCAAAAATGACACCATCTATATTTAGGAGGACATTCACTTGCCAATAATATCCGCC
CTGCCAACCTGTACAGAATAAGGGTCGATCTTCCCCTGGGTAGCCCAGCTGT-
GAATTGCACAGTCTTGCCAGGAGGAATCTCTGTCTCCAGTGCAA??????????????????????????????
??
??TGTGTCTCAGAATCTTCCTGCTC
TCCACCACACCGAGCCCACCATGACCCAGTGCTCCACAAACATATGTAGAATGGAAGTAAAACCTCAGGATA

AGAAAAGATAATTAAAGGGCTGGGCGTGGTGGTTCATGCCTGTAATACCAGCACT-
TAGGAGGCCGAGGCGAGTGGATYACAAGGTCAGGAGTTCAAGACCAGCCTGGCCAAGATGGTGAAAACCCATC
TCTACTAAAAATA---CAACAATT-----
AGCTGGG--
---------------------CGGGGTGGTGGGCACCTGTAATCCCAGCTACTTGGGAGGCTGAAGCAG-
AGAATTGCTTGAACCTAGGAGGCAGAGGTTGCACTGAGCTGAGATCACGCCACTGCACTCCAGCCTGGACAAC
A-GAGTGAGACTCCATCTCAA--

---AAAA-
TTATAATAAAAAGAAAAGATAGTTAAAGCAGATTCAATACAGTAACAAACCAGCCAAGAAATTGCAGCGAAAG
TAAAGGTTT-
GGATGACAAAATTTCCAACTGTGTTCAAAGAAACATAAGTTCCAGCAGGAGTGCACGTAATCTGATTTT-
CTTCAAAGC--
CTCTCTAATTTGTAGGGAAGGACATCCATTTTCATTAGCTTCATGTGGGCTAGAGAGAATCCATGATTGAGGC
GCTAAAACCCCTGGTAAACATGCCAGCAAGCAAGGGCACCTTCCCCAGAGGCTCAACCTAGAATATAAGACTG
TCGGCATTGGTAAGGAGCCTTGAACTCTAAACCCATAGGCTCAAATCAGGAA-
CTCAGGAAGCCTATCACCACCGGGATACAATTTTACCACCTTAACACTTTATTCCCTTTACTTGTGCCACACT
???
???
???
???
???
??????????????????????????????????AGGCCCTCAAAGATCTTTATTGCCATAAAGCAGAGTAAC
T-GTCTTTGACTTGGACCTTGTTTACTGTGAACTCTAATC-AAGGCAGGACATTCAGCAGTTCTACTTA-
CTGCACTGTGGACTTGTGGAAAGGACACTTGTGA---------
ATCCAGTTTGGATGCGTGTTTGAAATTGAATTGCGAATGGTGTTCCAGGATGTGGAATGCAGCCTTGATCACA
CTTGCCTACC-GAGCTTCCTTTGATCTGTTTGTCAATAGCATGCAAAAAA-
TGCTTTGTCTGCCCTTTGCTTCTGCTTTTTTCAGGGAAGCTGCCAAGGAATGTCGACGTCGAAAGAAAGAATA
CGTAAAGTGTCTGGAGAGCCGAGTTGCAGTACTGGAAGTCCAGAACAAGAAACTTATAGAGGAACTTGAAACC
TTGAAAGACATTT??
???
???
???
???
???
???
???
???
??TGTTTATATCTAAATGTGTGGATTCCA
GCACCTAAACCAAAAAATGCCACTGTAATGATATGG-
ATTTATGGTGGTGGTTTTCAAACTGGAACATCATCCTTACATGTTTATGATGGCAAGTTTCTTGCTCGGGTCG
AAAAAGTTATTGTAGTGTCAATGAACTACAGGGTGGGTGCCCTAGGATTCTTAGCTTTGCCAGGAAATCCTGA
GGCTCCAGGAAACATGGGTTTATTTGATCAACGGTTGGCTCTTCAGTGGGTTCAAAAAAATATAGCAGCCTTT
GGTGGAAATCCTAAAAGTGTGACTCTTTTTGGAGAAAGTGCAGGAGCAGCTTCAGTTAGTCTGCATTTGCTTT
CTCCTGGAAGCCATTCCTTGTTCACCAGAGCCATTCTGCAAAGTGGATCCTCTAATGCTCCTTGGGCAGTAAC
ATCTCTTTATGAAGCTAGGAACAGAACGTTGGCCTTAGCTAAATTTACTGGTTGCTCTAGAGAAAATGAGACT
GAAATAGTCAAATGTCTTCAAAACAAAGATCCCCAAGAAATTCTTCTGAATGAAGCATTTGTTGTCCCCTACG
GAACTCTCTTGTCAGTAAACTTTGGTCCAACAGTGGATGGTGATTTTCTCACTGACATGCCAGACATATTACT
TGAACTTGGACAATTTAAAAAAACCGAGATTTTGGTGGGTGTTAATAAAGATGAAGGGACAGCTTTTTTAGTC
TATGGTGCTCCTGGAGTCAGCAAAGATAACAATAGTATCATAACTAGAAAAGAATTTCAGGAAGGTTTAAAAA
TATTTTTTCCAGGACTGAGTGAGTTTGGAAAAGAATCCATCCTTTTTCATTACACAGACTGGGTAGATGATCA
GAGACCTGAAAACTACCGTGAGGCCTTGGATGATGTTGTTGGGGATTACAATATTATATGCCCAGCCTTGGAG
TTTA???TTGTGCTAATTGTG
AGAAAATATGCTACTTTGATTAAACTAGAATGAAGGAAATAAAAAGACACTGTGGCTCAACTTGTAATAAAGA

GCAGCTCATCCTCACAGCTTTTTGATTGTTGCTATAGTTATCT---
ATTCAACATGGGATGCACAATACAGTTAACGTTTCGTTACCAGTAGCCTGTTGCGTGCTTATTGTTAAAAGGA
ATTTAACATACAAGTTTGTGTATGGATTGTTCGCCTAATTCCGATTCCTCGTGCTGACTGGCATGAGACAGGG
AGCTGGATTGTATTAAACTGGTTTTATTGTGATGTGACTATGATGGATGTGACAAGGGCCTCTATTATTCGCT
GGGGAGTGTGGCCGTTAATGAAGCCATTAGCTGAGCTAAGGCAAGTGAAGAAAATAGGGAAATTACCATTATT
CTGAAGCCATTAAGTTGTAATCCACCACTTTCTCTCCTTATTCAAAAAGGCATCTTCTTTTTAAGGCCTCCCT
???
?????????????????CCAGGTGTGCGGACCCATGGGACTCTGGAGAGCGTGAATGGGCCCAAGGCAGGTTC
AAGAGGCCTGACGTCATCGTTGGCTGACACTTTCGAACATGTGATAGAAGAGCTATTGGATGAGGACCAGAAA
GTTCGGCCCCATGAAGAAAACAATAAGGACGCAGACTTGTACACATCCAGGGTGATGCTCAGTAGTCAAGTGC
CTTTGGAGCCTCCTCTTCTCTTTCTGCTGGAGGAATACAAAAATTACCTGGATGCTGCAAACATGTCCATGAG
GGTCCGGCGCCACTCTGACCCTGCCCGCCGAGGGGAGCTGAGCGTATGTGACAGTATTAGTGAG?????????
???
???
???TTCTTTGAGCAGATGCA
GAACTAGACCCCC-
GCCACAGCAGCCTCTGAAGTTGGACAGCAAAACCATTGCTTCACTACCCGTCGGTGTCCATTTATAGAATAAT
GTGGAAAGAAACAAACCCCTCTGTCTTATGATTTACTCATTATCGCCTTTTGACAGCTGTGCTGTAACACAAG
TAGATGCCTGAACTTGAATTAATCCACAGATCAGTAATGTATTCTCTCTCTCTCT------TT----------
--------ACATTTTGGTCTCTACACTACATTATTAATGGGTTTTGTGTACTGTAAAGAATTTAGCTGTTTC-
AAACCTAGTGCATGA-
ATAGATTCTCTCCTGATTATTTATCACATAGCCCCTTAGCCAGTTGTATATTATTCTTGTGGTTTGTGACCCA
ACTAAGTCCTACTTTAAATATGCTTTAA-GAATCGATGGGGGA---TGCTTCACGTGAACGTGGGAGTT-
AGCTGCTTCTCTTGCCTAAGTATTCCTTTCCT-GATCACTATGCATTTTAAAGTTAAA-
CATTTTTAAGTATTTCAGATGCTTTAGAGAGATTTTTTTTT-
CCATGATTGCATTTTACTGTACAGATTGCTGCTTCTGCTCTATTTGTGATATAGGAATTAAGAGGAT??????
???????????????????????????????????????TCCGGAGAGCACTAAGCCACTTCCAGCCCTGCTGT
GAACCAGGACTGTAGGAGCCATCTTTTTCATGTGGTCAGCTTTGGAAGCATCTACATCCACCACTTTAGAAGA
CAAGTCTAGTGGCTTATCTGTGACGTCTTTGGTAACGGTCTGCTTCTCCAACAGAGGAGGTGAGCTGCCGTCT
TTTCTGTCTTGAACTGCTGTCTTCCGGGCATGCCCAGGCACTGGCTGGGCACCTTCGCCCCCTTCCAGAGCCT
TGGGATACTTGCCGTTGGAGAGCCTGGCCGCGGGGAACTCGCTGCTGACTGTCATGTATGGCTTCGACAGGGC
AACTGAAGGAGAGGTGGAGATCCTGGCATAGTGCTTGTGGAACTCCGAGTAGGTATCTGCAGCAGGCTGGGTG
GGAAGGTGGACCCGGGGTGAGGGCCGAGGTGAGGGGGGCAACAGGAGAGCTGTGTCCCCCGGCAGGCCACTGG
TGACTGCCTTGGCAGAGGGAACCCTGGGCTGTTTACTGTTCTGGATGTGAGGATAGGCGTGGGAATCAACGGG
ATTCCCAGGGCTGACCCCCATCTTCCACGGGAGGCTTTTGTCTGCACAATGGACAAGAGGCGGGATGGCTGGG
GAGGCCGAAGGCGTCGAGAGCCTCATGGGTGATGCCAAGGACGATGGGATGTGGGGACTGACGTAGTGAGGTG
GTGGCAGGTAGAGAAAGCGCTCCCCGTTGGTGCAGACTGGAGAATACAGCGGCTGGGCCAAGCTGTAGGACTG
CTGAGGT???????TGCCAGGACCTTTGTAATTCAAGTGCTTTTTACAGTAGTAGCAAATGTTATATAAGCCC
AAACATGGAGCA---
TATTTGGAGTTGGTGGGCAGACCCAAATCATGATGCAAGCCTGCAGTAGCAAGTGTGCAATGAAGCACAACCA
AATTCTGTGAATGGAAGGTAGCGAGACGCCTCTCCAAAGCAGCCCTTCTGCGCAGGAGCTGAAAGCTCCACCG
GGTAAGTTTTCTGAGGCAGTTTTCAGAGGCTGGGCTTGGCTCAGGGCCCAGCTCTGGATGAGCCTGTGGACGG
TGGGGTGGGTAGGTCTTTGCTAACCTTACATAGAACCAAGCAGAGTTTGGAAGAGTTTGTGCAGGCCTCTTTC
CTCCTGGTGTTTGTTTTGCCCTGCCAGCAGGCCAGGAGGG-
CTGTGCTGCTCAAGTAGTGGAGGGAGGGAGCCTGCAGAGCTAGGGGATCAGAGTGGGTAATCGCAGCCATAGG
GATTAGCTGCTGCAAGCTGTCCACAGGAGCCATTCTGAAATGTCATTTGAGTGATCTGGCCGTCTACTGGAGG
AGGCCCTCGGGGCAGCTGTCTCCAGAAGCAGCTTCTGAGGGAGTCTTCCAGGAGGAGCAGTCTCTCTGCCATC
GTCCTCGTCCACGTCCTGGTCACATTTCCATTCCTCATCTTCGTTTAAAGTCGGCAGGTATCCAGATATCCCC
TTCCCTGTCCCTGACCCAGAGCTCTGGTCACTACAGACCTTCGGGCTCTCCGAGCCTGTCCTCGTATATTCCA
AATAAATATCAGACTTAAGGAAGGAGGGGTAGGTGTTTTCCTCCATAGTGGCCTGGATTTCTGTCTGGGCCTG
GTCAAACATGGCAGGATCGATCAGCTGCTTCATGATGCAGCCCTTTATGAAGCTCTTGGTGGCTGGCTTGGTC
TGCCGGGACACGATGCCATTGTTATCAAGGATGTACTTTCGGTAGATGGCTCTGGCCAGCTTCAGCCTCTTCT
CCTCATTCGAGTCACAGGGCTCCAGCTTCCTGAAGCCAGTGCAGGCAAACCAGAAGTCCAGCAAGTCAGCACA
GCCCTCCTGCTTCAGGAAAGTCCTGAACAGGCTTATCCCATCTTGATCATCCAGCAGGGAGTGCAGTGACTCA

GCCCACTTCAAGTACGGTGGGGTGGGGGAGGCACTGCCCTCAGGCTCATACCCCAGGTCCAGATCCGAGCGTC
TCGGAGTGGCGGTCGAAGTCTCACCTTTAATGCCAACACCTTTCCCGGAGCAGAAACTGTAGCTGGCGGGCCT
CGGGTCTGTGGACACCAGTTCTCCCTCCTCACCAGGCACTGGGGGTCGGGGAGCATCTTCGGTGAAACTTGCT
CCAAGGTCCAAGGGGAAACCCTGCTCCTGGACATTCATTTTGGGACTCTGTGCGTCAATGCACAATGAGCGCT
GCACCCTAATACATCAGTACTTAACAGCTCCAAAGTCAATCAATCTGTCCTGTTGAAACCAT????TCTAAAA
TGTTAACTAATTTTAAGTGAACGA-A--
ATTACCTGGATAAACTTATGGCAGCATGAAACAGGTATAATTTACAGTGGAGCAGGATAAGT-
ACTTAGGTTATTTATAGGACAGATT-TTTTTGTGTGTTGTTTTG----CTTCTTTG-CATATTAAAATTTAT-
---AGGTGTGGTTAAGCTTT--ACTATTAGTTAGCATAAATTTGGCTTGGAGTCA----
CCAAAGCAGAAATGTTGGACTTCATTTTTATGAGTTATTTCCTACACATTTCTAAATTGATGTAGCTTTGTCA
CTGCTTGATTGTTTTTTTTT---
AAACTGGAGGGGGTGAAATTAGTTTATACTGAGTCAAACCAGTTGCTCTTAATTAGTA---------------
----CATTACCAACATTGAAAAGAAGTAAAAGGAATGTTAATAACTTAGGAGTATTTGCGTAGTTA-
CTTTCAGAACACAAGGTTTAAATTAAT-GAGGAATCCG---------
GAAGTTTGATTTAAGCACTCATACTCCTTTATTTTCCTTTGTTAGCAATAATTATTGGCCCTGATGGCCATCC
TTTGACTGTTTATCCTTGTATGATTTGTGGAAAGAAGTTTAAGTCGAGAGGTTTTTTGAAAAGACACATGAAA
AACCATCCCGAACATCTTGCCAAGAAGAAGTACCACTGTACGGACTGTGATTACACTACCAACAAGAAGATAA
GTTTACTCAACCACCTGGAAAGCCACAAACTGACCAGCAAGGCAGAGAAGGCCATTGAATGTGATGAGTGTGG
GAAGCATTTTTCTCATGCAGGGGCTTTGTTTACTCATAAAATGGTGCACAAAGAAAAAGGAGCCAACAAAATG
CACAAGTGTAAATTCTGTGAATACGAGACAGCTGAACGAGG?????????????????????????????????
?????????????????????CCGCTATAGCTTCTGCAAACAGTAAAGATACCACTAAAACCCCTTCTGCAGT
TCAAACTAGCACCAGCCACAACCCTGGACTTCCTCCTAATTTTAACGAATGGTACGTCTGAGGACAAACACAA
ACCCTGTTAACTATAGAATGGACCAAATGCATTTTTAAAAGAAAACTGAGACCAATCAGATGGAAATGGAGTT
TTAAGGCAAGAGGCCATATATAGGGCTACATCTTGTTAATTGCAATTGTCCAGGAAGGTTTTGGGCAAGATCC
AAAAGTAGCCATGCCCTTTTCTCAGGATTAGAAAATATGTTTTGGCGTTTGAAGGATTTTTT--
ACAAAATCTTTACACTGCTTTTTCTTCCCCTTCCTCTTGCTCTCTGCACACCCCATTCTTAAATTCCTGCAAT
TCATTTTAACACTTGTCCTGTTTCTTGAGAGGAAGTTATAGAAGGCTTGTTGGTGGTGGTGATGTTAAACTGT
TGGAAATTCTTTTTCGCCTTAGTGGTGATTGTTTAAACTCTCACAGTCTTAAACCGTGCCAAAGTCCTGTTAT
A
Leontopithecus
?TACCCCCATGTTTGGCTGGAACATGAAGCTGACCTCAGAGTACTATAGAAATGTCACCTTCCTTTCATGCCG
GTTCCCTT-
CTGTCATGAGCATGGACTACATGGTATACTTCAGCTTCTTCACCTGGATTTTCATTCCCCTGGTCATCATGTG
TGC--AATCTATCTTGACATCTTTTACAT-
CATCCGGAGCAAACTCAGACAGAACTTCTCTAACTCCAAAGAGACAGGTGCATT---
TTACGGACGGGAGTTCAAGACGGCGAAGTCCCTGTTTCTGGTGCTTTTCTTGTTTGCTTTGTCATGGCTGCCT
TTGTCCATCATCAACTGCATCACCTACTTTCA--------------
TGGTGAGGTACCACAGCTTGTGCTCTACTTGGGCATCCTGCTGTCCCATGCCAACTCCATGATGAACCCTATC
????????????????????ATGTTCATAAACCGCTGACTATTTTCAACTAACCACAAAGACATTGGGACAT
TATATTTATTATTCGGTGCATGGGCGGGGGCAGTAGGTACAGCCCTAAGCCTCCTAATTCGAACAGAACTAGG
ACAACCCGGAAGCCTAATAGAAGACGACCATGTGTACAATGTTATTGTCACCGCCCACGCATTCATCATAATT
TTCTTCATAGT---AATACCAATTATGATCGGGGGTTTTGG---
AAACTGACTCATTCCTTTAATAATTGGCGCCCCCGATATAGCATTTCCTCGAATAAATAACATAAGCTTCTGA
CTTCTACCACCC--TCCCTACTTCTACTGCTTG-
CATCATCAACTTTAGAAGCCGGTGCCGGCACTGGTTGGACAGTCTACCCACCCCTAGCAGGCAATTTATCCCA
CCCAGGAGCCTCTGTAGATTTAACTATCTTTTCACTACATTTAGCAGGCATTTCTTCTATTCTTGGAGCTATT
AACTTTATTACAACAATTGTAAATATGAAACCACCAGCCATGACTCAATATCAAACTCCGTTATTTGTGTGAT
CCGTCCTAATTACCGCAGTCCTTCTTTTACTTTCTCTTCCAGTTCTAGCTGCAGGGATTACTATACTATTAAC
TGACC--
GTAATTTAAATACTACTTTCTTTGATCCTGCTGGTGGCGGCGACCCTATCCTATATCAACACTTATTCTGATT
CTTCGGTCACCCTGAAGTATACATCCTAATTTACCGGGTTTTGGAATAATCTCACATATTGTAACATACTAC
TCTAATAAAAAAGAACCCTTTGGTTATATGGGCATGGTATGAGCCATAATATCTATTGGTTTCCTAGGCTTTA
TTGTATGAGCCCATCACATATT-
TACAGTGGGAATAGACGTAGATACTCGTGCATATTTTACATCAGCTACCATAATCATTGCTATTCCCACTGGA

GTAAAAGTATTTAGCTGATTAGCCACACTGCACGGCGGTAATATCAAATGATCTCCCGCAATATTATGAGCCC
TGGGCTTTATTTTTCTTTTTACCGTGGGTGGACTAACAGGAATTGTATTAGCTAACTCATCATTAGATAT-
TGTATTACATGATACATATTATGTAGTAGCCCACTTTCACTATGTATTGTCAATAGGAGCAG-
TATTTGCCATTATAGGGGGATTTATTCACTGATTCCCGCTCTTTTCAGGCTATACTCTTGACCAAACATATGC
CAAAATTCACTT-
CACCATTATATTTGTCGGCGTAAATTTAACTTTCTTCCCACAACACTTCCTTGGCTTATCCGGAATGCCCCGA
CGATACTCAGACTACCCAGACGCATATACCACATGAAATATCGTCTCATCTATTGGTTCATTTATTTCACTTA
CAGCAGTAATCCTAATGGTTTTTATGATTTGAGAAGCCTTCTCTTCAAAACGAAAAGTCTTAGCCATTGAGCA
ACTATACACCAATCTGGAATGATTATACGGCTGCCCTCCTCCTTATCACACATTCGAAGAGGCAACTTACGTT
AAATCCTAG??????????????????????????ATAGCAACACCAGCTCAACTAGGTTTACAAAATGCCA
CATCACCTATCATAGAAGAACTTATTGCTTTCCACGACCATGCACTCATAATTATTTTCCTGATTAGTTCCTT
AGTCTTATATATTATTTCCCTTATACTTACCACAAAACTCACTCATACTAGCACCATAAATGCTCAAGAAATC
GAAATAATCTGAACCATCCTCCCTGCACTGATTCTAATTACAATTGCCCTCCCATCACTACGTATTCTATATA
TGACAGACGAATTTAACAAACCTTATTTAACCCTTAAAGCAATTGGTCACCAATGATACTGAACCTATGAATA
CTCCGACTATGAAGACTTAGTATTTGACTCTTATATCATGCCAACATATTTCCTTGAGCCAGGAGAATTTCGA
CTCCTCGAAGTTGATAACCGAACAACTTTACCTATAGAAGCGGATATTCGCATATTAATTTCATCACAAGACG
TCTTACACTCATGAGCCGTACCATCACTAGGCGTAAAAACAGATGCAATTCCTGGACGTTTAAACCAAGCCAT
ACTAGCCTCTATACGACCAGGCCTATTTTATGGACAATGCTCGGAAATTTGCGGGTCCAATCACAGCTTTATA
CCTATTGTTCTAGAATTCATTTATTTCCAAGATTTCGAAGTATGAGCTTCATACTTATATATTGTAT??????
ATGACCTCCCCTCGCAAAACCCACCCACTAGCAAAAATCATCAACGAATCATTTATTGACCTACCCACACCAT
CCAATATCTCATCCTGATGAAATTTCGGTTCACTTTTAGGCACCTGCCTAATTATTCAAATCACCACAGGCCT
ATTTCTAGCAATACACTACACACCAGATACTTCCACCGCTTTCTCCTCAGTAGCCCACATTGCCCGAGATGTT
AATTATGGATGAATAATTCGTTATTTACACGCCAACGGTGCATCCATATTCTTCATCTGCCTTTTCCTCCACA
TCGGACGAGGCCTATATTACGGATCTTTTCTTTCTCTGAAGACTTGAAATGTGGGTACAATCCTACTATTAGC
AACTATGGCCACGGCATTTATAGGTTATGTACTTCCATGGGGCCAAATATCATTCTGAGGGGCTACAGTAATT
ACAAACCTCTTATCAGCCATCCCCTACATCGGCTCCGACTTAGTGCAATGAATCTGAGGTGGATTCTCAGTAG
ATAAAGCCACCCTAACACGATTTTTTACCTTTCACTTTATCTTACCTTTCATTATCGCAGCCTTAGCAACAAT
TCACCTTCTCTTTCTGCATGAGACAGGTTCAAGTAACCCATCAGGAATAGCCTCAGAACCCGACAAAATCACA
TTTCACCCATATTATACAACCAAAGACATTCTTGGACTAATTTTTCTTCTCCTATTTCTAATAAGCCTAACAC
TATTTTTACCTGACCTCTTAACAGACCCAGATAACTACACACTAGCCAACCCCCTAAACACCCCTCCCCATAT
TAAACCAGAATGATATTTCCTATTTGCATATGCCATCCTACGATCTATCCCTAATAAATTAGGGGGAGTTCTA
GCACTTGTACTTTCTATCCTAATTCTAATAATTATTCCCATAACGCACTTATCCAAACAACAAAGCATAAAAT
TCCGACCTATTTCTCAAATCCTGTTCTGAACCCTAGTAGCCGATGTACTCAAACTTACATGAACTGGAGGCCA
GCCAGTCGAATACCCTTTTATTACTATTGGCCAAACTGCATCCATTATATATTTCCTGATTATTCTCACCCTC
ATTCCCCTCTCTGCCCTAGTCGAAAACAAACTACTTAAGTGATAA?TTAGGTTTAATTTATTCAAGGGAATGA
GAATTTATGGGTATAACTGTTAGCTGGAAAGCCCTTCATCACTTCCAATCCTCCTTTCAAATTACATTTCC-
TCCTGAAATTTCTCTGTGGGATGAAAGTTTCAATTATTAGTCAATCAATC----
TCTTGTACGTTATCTGGCTTTACATAT----
GCCCTTGATAGTTCTTGGAAAATAATCCAATATTGTGTTGTTTTGGTGTCTTTGTATTGGAAATG-
AAGCATTTTGTATGACTCACCTCTTTAACTGTGAGGAGTTGAATTGTCTTAGTTCTGCTTGCCTAATGACCAG
GAATAAA--GACATCACAGTCTCTAACCCCACTTTTT-
GGTGGAAAGAAAATCTAAAAGTAGCAACATTTAATGGAACAAAATATCAATAATCAAAGAAACTCCCCTGTGA
AACTGTAATATTATTTT-
GAAATCGACATTGTTCTAAGGTAGCTGAAATGTGTGATTTTTCTATTTTGCCTTTTCACTGACAATATACCCA
TAAATCTTTAGTGGTGATTTTCTCTCCAAAGTTAATTTCTGGGCCATTGGTGACTGCACTATCTATTGTTCTA
AATTGTTGTCTTAATTGCATTCTTTAAAAATGTAATGTTGAAGGCACATAATATTGAAGATGACTCCAGGATG
AACCCTGAGTTCGCAGACCGAA-
TAAAACAGCTCGATAAAGAGGCATCTTATTACCGTGATGAGTGTGGCAAGGCCCAAGCGGAAGTGGACCGGTT
GCTGGAGA?????????GGAAGAGCTGCATCAAGCAAGTACTTTGATCTTTCTTGGAGCAGGTTCCTTCAAAA
CGCTTCTCCAGAGGACGGAAGTCCCTCCAGTCTGGCTCACTGCTCGCAGTGGTGGGGAAAGCTGAGGTCATTC
CCCGGTGGGAAATCTGCGAGGCCCCTGATACCCCTG--------
CCACCAGGCCCATCACTGATGGGCCTAAGGGCCTCATCTGATACTCTAAGATGGGTTTCTCCTGTCGGATCTG
GGCCTCTCGGACTTGAGCCTCTTTAGAACGCGCCTCTCGGGCCTGGGCTTCTCGGGTTCTGGCCTCCCTGGCA
TGACTTTCCCAAGTGTGGGCCTCCCTG--

GCATAGGCTTCCTTGCCATGAGCTTCTCGAGTATAGGCCTCCCTGGTGTGGGCCTCCCTGGCCTGGGCTTCTC
GGGCACGTGCCTCCCGGGCCTCAAGCTGCTCCCTCCGAAGCTCCCAATACAACAACTGTTTCTGGATGGTCA-
CCAGCCGTTCCTCCTCTGTCTCCATTGCCCCAGGGGGCCGGGAGGAAGAAAAGGGCTCAAAGTTCAAGAAGGG
GTCAAACATCTCAGAGCTTCGACCACAAAGGTCATAAAGGCAGTCATCCCCAGGTGGAGAGTTCTCAAGACTG
TCATCTGGCTCATAGAACTCATATAGGGCATCTCCACTGTAGCTGTCTCGGGGCAGACAATCCCTATGG????
??
???????????????????GTGAATGTTCTCCACTGTTGTGGTGGGCATGTTTCATGTGTGGGGATGTTGGTG
TGCAAGTTTCATGTGTTTGGAAAACACTAGCTAGAACATTCGAAAAATTTCATTAACCCAGCTCTGGAAGGTC
ATCTATGAATTCTTCCTATTCCAGTGGCCTTTT-
AAGTATAGATGGAGATACACTGTAATTTAAAACAGATGGGCATGTCTGAATACTTTCATATGCAAAATGGGAC
AGATGTATATGAATGGATAAATTATCCTTTTAAAAAAATACTTTGTAGCACAAAATATCACCAATGATTGGAA
GAGTGAACCTGCATTAGGTTTAGGGCAAACACCAGGAAGACATTATCAAAACATCTAA-
CTATCTACAAACTAATTCCAATTAGTCTACTTTGTCTTCGTACAGAAAAAA-CTTTTTTGATTTTTAAA---
TATATATTGGGTGCCAATCCGTATCCTTT-
GAGCTGCTAAAGCCAGCAT???????????????????????????????????????ATGTATATAATTAATT
TACTATTACTAGTCGTACCCGCCCTAATTGCCATAGCCTTTTTAACACTCACAGAACGAAAAATCCTGGGTTA
TATACAATTCCGAAAGGGCCCTAATATTGTAGGTCCCTACGGAATACTTCAACCAATCGCTGACGCCATAAAA
CTCTTCACAAAAGAACCCTTATTACCTACCACATCCACCATAACTTTATACTTAACTGCCCCCACCCTAGCTC
TTTCCATTGCTCTTCTACTATGAACGCCACTCCCCATACCATATCCCCTAATCAACTTCAATCTTGGTCTCCT
ATTTATCCTCGCAACATCAAGCCTAGCTGTTTACTCAATTTTATGATCTGGCTGAGCATCCAACTCAAACTAC
GCACTAATTGGCGCACTACGAGCTGTAGCCCAAACAATCTCATATGAGGTTACCCTTGCCATTATCTTACTAT
CAACTCTACTAATAAGCGGCTCATTCAATCTACAATCACTTATTACCACTCAAGAACACCACTGACTTCTACT
TCCATCATGGCCCCTAGCCATAATATGATTTATTTCCACACTAGCAGAAACTAATCGAGCTCCATTCGACCTG
ACAGAAGGCGAATCAGAACTAGTATCAGGTTTCAACATTGAATACGCTGCAGGCTCATTCGCCTTATTCTTCA
TAGCAGAGTATATAAATATTATTATAATAAACGCCCTAACTACCACTATTTTCTTATCCACACCCTACAATAT
AATTATACCAGAAACATATACTATCAATTTTATAGCCAAAACCCTCCTACTAACCACCTTATTTTTATGAATT
CGAACAGCCTACCCTCGCTTCCGCTACGATCAATTAATATTCTTATTATGGAAAAACTTTTTACCACTTACAT
TAGCACTATGTATATGATATGTTTCAATGCCCATCCTAGCATCTGGCATCCCACCCCAAACATA?ATTAATCC
TCTAGCCCACCTTATCATTTCCTTCACCATTCTAACAGGGACCATAATCACAATTTTAAGCTCACATTGATTC
CTAGCCTGAATAGGCCTAGAATTAAATATACTAGCCATTGTACCAATCCTAGCCAAAAGTACTAATCCCCGCT
CCACAGAGGCATCCACCAAATATTTTTTAATTCAAGCAACAGCATCAATAATCCTATTAGTATCTATTTTCCT
CAACAATCTATTAACTCAACAATGAACAATTAATCCCCCTTATAACCAAATATTGTCCACAATAATATTTATT
GCCCTAGCAATAAAAATAGGGATAGCCCCACTTCACTTCTGATTCCCAGAAATTACCCAAGGAATCCCTCTAA
TCCCAGCTATAATTATTCTCACGTGACAAAAACTCGCCCCAATATCAATCCTCCTCCAAATTTTTCCGTCAAC
AAACCTAAACTTGATTCTAACAATCTCAGTTCTATCAGTTATAATTGGCAGCTGAGGAGGACTCAACCAAACA
CAACTCCGCAAAATCCTAGCCTATTCTTCAATTACCCACATAGGATGAATAATAGCAGTACTATATTACGACC
CTCATATTACTATATTAACTTTAATCATTTATATTTTCCTAACAATCTCTACATTAATAATCTTTTATTTAAC
CTCAAATGTAACAACCCTATCCCTATCACATACCTGAAACAAGCTAGCATGAACAATACCCATCATTCCACTA
ATAATAATATCCCTAGGAGGTCTACCCCCACTAACAGGTTTTTCCCCCAAATGAGCTATTATACAAGAACTTA
TTAAAAATGATAACTTAATTATTCCCCTTTTAATAGCTTTACTAACACTAATAAATTTATATTTTTATATACG
TTTAATATATTCCATCTCAATGACAATATTCCCAACATCAAATAACACAAAAATCAACTGGCAACTAAATTAT
ATAAAGCCAATACCACTTCTATCCCCACTTGTAGTGTCTTCTACTTGTCTTCTACCCCTAACTCCACTAATAC
TCATAATTT?????GTTGGCAGCATAAATAATAAAGCATGTTACTTAAGCAATTTTACATTTACTGTATTAGT
AAGTAAATGCAATGGCCACTCTTATTCTAAAACAATAAAAACTACCCAGCCTTTTCATGAAAGAT-
GATTGCAATTTCCATTGGCCTGTATATGCAATAAAAAG---
CCATAACTCATCAAAATTTTAGCTATTTTCTCTTAAGATA--
GAAGAGTGTTTTGCTTGAACATTGAACGTGACATTAAATATT-
CATGAAACTGAATAGAGTTTAAGTACTAAG---
TAAAACTCTCAAATTCTTTTCAACTAGATTGAAATTCTGTTTGAACCAGGTAAAAACTACCGCTAACCTTAAG
AGGTGTTCAAAAAC-
ATCATAAAGAGATTGTACAATACGTTCAAAGTGCTACAGTTATTTGGGATGGATTTTTCTGAAAAGCCTGTTC
CTCCTTCGAAATTGATTCATGATAACAGCCTCCATTTGGGTCAAACAGTATTTAACCGCTTGTAAA-
TTTTAATT-
AAATGATTTGTCACTTTGAACACATAGTTTGTCCCCAAGACCTTGAGGATTACTTGAATGGCCCTTTCACTGT

GGTTGTGAAGGAGTCTTGTGACGGAATGGGAGATGTGAGTGAGAAGCATGGGAGTGGGCCAGTAGTCCCGGAA
AAGGCAGTCCGTTTTTCATTCACAATCATGAAAATTACCATAGCCCACAACTCTCAGAACGTGAAAGTGTTTG
AAGAAGCCAAACCTAACTCTGAACTGTGTTGCAAGCCATTGTGCCTTATGCTGGCAGATGAGTCTGACCACGA
GACCCTGACTGCCATCCTGAGTCCTCTCATTGCTGAGAGGGAGGCCATGAAGAGCAGTGAATTAATGCTTGAG
ATGGGAGGCATTCTCCGGACTTTCAAGTTTATCTTCAGGGGCACTGGATATGATGAAAAACTTGTGCGGGAAG
TGGAAGGCCTTGAGGCTTCTGGCTCAGTCTACATCTGTACTCTTTGTGATGCCACCCGTCTGGAAGCCTCTCA
AAATCTTGTCTTCCACTCTATAACCAGAAGCCATGCCGAGAACCTGGAACGTTATGAAGTCTGGCGTTCCAAC
CCTTACCATGAGTCTGTGGAAGAACTGCGGGATCGGGTGAAAGGGGTCTCAGCCAAACCTTTCATTGAGACAG
TGCCTTCCATAGATGCACTCCACTGTGACATTGGCAATGCAGCTGAGTTCTACAAGATCTTCCAGCTAGAGAT
AGGGGAGGTGTATAAGAATCCCAATGCTTCCAAAGAGGAAAGGAAAAGGTGGCAGGCCACATTGGATAAGCAT
CTCCGGAAGAAGATGAACCTCAAACCAATCATGAGGATGAATGGCAACTTTGCCAGGAAGCTCATGACCAAAG
AGACTGTAGATGCAGTTTGTGAGTTAATTCCTTCTGAGGAGAGGCACGAGGCTCTGAGGGAGCTGATGGATCT
TTACCTGAAGATGAAACCAGTCTGGCGGTCATCATGCCCTGCTAAAGA--GTGCCC-
AGAATCCCTCTGCCAGTACAGTTTCAATTCACAGCGTTTTGCTGAGCTCCTTTCTACCAAGTTCAAGTATAGA
TATGAGGGGAAAATCACCAATTATTTTCACAAAA??????????????GGCACCCGACAGTTGTCATTATGTT
TGGAAACACTGCCGGCCCATGGAGATGAAGATAAAGTGGACATTTCTCTGCTGCATCAGGGTGAGAATCTTTT
CGAACTGCACATCCACCAGGCCTTCCTGACATCTGCCGCCCTGGCTCAGGCTGGAGACATGCAACCTACCACT
TTCTGCACCTATTCCTTCTATGACTTTGAAACCCATTGTACCCCATTGTCTGTGGGGCCACAGCCCCTCTATG
ACTTCACCTCCCAGTATGTGGTAGAGACAGACTCCCTTTTCTTACACTACCTTCAAGGGGCTTCAGCCCGGCT
TGATCTCCACCAGGCCGTGGCCAGTGAGCACAACACTCTTGCTGCAGGATGGATATACTTTGACAGGGTGCTA
GAGACTGTGGAGAAAGTCCATGGCTTGGCCACACTGATTGGTAAGTGCTGTCGGCTTCCTGCAGCTCCTA-
GCACCAATGCAGAATTTCCCAAACTCTA--
GCTGCTTTTCTGGTCATTTCTTATTTTCTTCCTTTTTCTTTGTTCTTGATCATTGCCACACCGTCTGTATTCT
CCATGATTTCA-
TACTTGCTGCTGCCCAGGAGCTGGTGGAGAAGAGTTTGGGGTTCTAGAGTACTGGATGAGGCTGCGTTTCCCC
ATAAAACCCAGCCTACAGGCATGCAATAAACGAAAGAA????????????????????????????????????
??????????????????????????GTTCATGGAGGGAAAACACCAAACAATGAACTTTCAGATAAAATTTA
TGTCATGTCTGTTGTTTCCAAGAACAACAAGAAGATTACTTTTCGCTGCACGGAGAAAGACTTGGTAGGAGAT
GTTCCTGAAGCTAGATATGGTCATTCCATTAATGTGGTATATAGCCGAGGGAAAAGTATGGGTGTTCTTTTTG
GAGGACGCTCATACATGCCTTCTACCCACAGAACCACAGAAAAATGGAATAGTGTAGCTGACTGCCTGCCCCA
TGTTTTCCTGGTGGATTTTGAATTCGGGTGTGCTACATCATACATTCTTCCAGAACTTCAGGATGGGCTATCT
TTTCATGTCTCTATTGCCAAAAATGACACCATCTATATTTTAGGAGGACATTCACTTGCCAGTAATATCCGCC
CTGCCAACCTGTACAGGATAAGGGTTGATCTTCCCCTGGGTAGCCCAGCTGT-
GAATTGCACAGTCTTGCCAGGAGGAATCTCTGTCTCCAG???????????????????????????????????
???
??TGCGTCTCAGCATCCTCCTGCTC
TCCACCACACTGAGCCCAAGATGACCCAGTGCTCCACAAACATATGTAGAATGGAAGTAAAACCTCAGGGATA
AGAAAAGATAATTAAAGGGCCAAGCATGGTGGTTCATGCCTGTAATCCCAGCACT-
TAGGAGGCCAAGGCGAGTGGATCACAAGGTCAGGAGTTCAAGACCAGCCTGGCCAAAATAGTGAAACTCCGTC
TCTACTAAAAATA---CAACAATT-----
AGCTGGG--
---------------------TGTGGTGGTGGGCACCTGTAATCCCAGTTACTTGGGAGGCTGAAG-AG-
AGAATTGCTTGAACCTGGGAGGCAGAGGTTGCAGTGAGCCAAGATCACGCCACTGCACTCCAGCCTGGACAAC
A-GAGTAAGACTCCAACTCAA--

--AAAA-
TAAGAAGAAGAAGAAAAGATAGTTAAAGCAGATTCACTATAGTAACAAACCAGCCAAGAAATTGCACTGAAAG
TAAAGGTTTTGGGTGACAAAATTTCCAACTGTGTTCAAAGAAACATAAGTTCCAGCAGGAGTTCACTTAATCT
GATTTT-CTTCAAAGC--
CTCTCTAATTTGTAGGGAAGGACATCCATTTTCATTAGCTTCATGTGAGCTAGAGAGAATCCATGATTGAAGT
GCTAGAACCCCTGGTAAACATGCCAGCAAGCAAGGGCACCTTCCACAGAGGCTCAACCTAGACTATAAGACTG
TCAGCATTGGTAAGGAGCCTTGAACTCTAAACCCATATGTTCAAGTCAGGAA-
CTCAGGAAGCCTATCAACAGCGGGATACAATTTTGCCCCCTTAACGCTTTATTCCCTTTACTTG?????????
???

???
???
???
???
????????????????????????TAGGAACTCAAGGCCCTCAAAGATCTTTATTGCCATAAAGCAGAGTAAC
TTGTCTTTGACTTGGACCTTGTTTACTGTGAACTCTAATC-AAGGCAGGACATGCAGCAGTTCTACTTA-
CTGCACTGTGGACATGTGGAAGGGACACTTGTGA---------
ATCCAGTTTGGATTTGTGTTTGAAATTGAATTGCAGATGTTGTTCCCGGATGTGGAATGCAGCTGTGGTCACA
CTTGCCTACC-GAGCTTACTTTGATCTGTTTGTCAGTAGCATGCAAAAAA-
TGCTTTGTTTGCCCTTTGCTTCTGCTTTTTTCAGGGAAGCTGCCAAAGAATGTCGACGTCGAAAGAAAGAATA
CGTAAAATGCCTGGAGAGCCGAGTCGCGGTGCTGGAAGTCCAGAACAAGAAGCTTATAGAGGAACTTGAAACC
TTGAAAGACATTT??
???
???
???
???
???
???
???
???
??TGTTTATATCTAAATGTGTGGATTCCA
GCACCTAAACCAAAAAATGCCACTGTAATGATATGG-
ATTTATGGTGGTGGTTTTCAAACCGGAACATCATCCTTACATGTTTATGATGGCAAGTTTCTTGCTCTGGTCG
AAAGAGTTATTGTTGTGTCAATGAACTACAGGGTGGGTGCTCTAGGATTCTTAGCTTTGCCAGGAAATCCTGA
GGCTCCAGGAAACATGGGTTTATTTGATCAACAGTTGGCTCTTCAGTGGGTTCAAAAAAATATAGCAGCCTTT
GGTGGAAATCCTAAAAGTGTAACTCTTTTTGGAGAAAGTGCAGGAGCAGCTTCAGTTAGTTTGCATTTGCTTT
CTCCTGGAAGCCATTCCTTGTTCACCAGAGCCATTCTACAAAGTGGATCCTCTAATGCTCCTTGGGCAGTAAC
ATCTCTTTATGAAGCTAGGAACAGAACGTTGGCCTTAGCTAAATTTACTGGTTGCTTTAGAGAAAATGAGACT
GAAATAGTCAAATGCCTTCAAAACAAAGATCCCCAACAAATTCTTCTGAATGAAGCATTTGTTGTCCCCTACG
GAACTCTCTTGTCAGTAAACTTTGGTCCAACAGTGGATGGTGATTTTCTCACTGACATGCCAGACATATTACT
TGAACTTGGACAATTTAAAAAAACCCAGATTTTGGTGGGTGTTAATAAAGATGAAGGGACAGCTTTTTTAGTC
TATGGTGCTCCTGGCTTCAGCAAAGATAACAATAGTATCATAACTAGAAAAGAATTTCAGGAAGGTTTAAAAA
TATTTTTTCCAGGAGTGGGTGAGTTTGGAAAGGAATCCATCCTTTTTCATTACACAGACTGGGTAGATGATCA
GAGACCTGAAAACTACCGTGAGGCCTTGGATGACGTTGTCGGGGATTACAATATTATATGCCCAGCCTTGGAG
TTTACAAAGAAGTTCTCAGAATGGGGAAACAATGCCTTTTTCTACTATTTCGAACACCGTTGTGCTAATTATG
AGAAAATATGCTACTTTGATCAAACTAGAATGAAGGAAATAAAAAGACACTGTGGCTCAACTTGTAATAAAGA
GCAGCTCATCCTCACAGCTTTTTGATTGTTGCTATAGTTATCT---
GTTCAACATGGGATGCACAATACAGTTAACGTTTCATTACCAGTAGCCTGTTGCGTGCTTATTGTTAAAAAGA
ATTTAACATACAAGTTTTTGTATGGATTGTTCGCCTAATTCTGATTCCTCGCGCTGACTGGCATGAGACAGGG
AGCTGGATTGTATTAAACTGGTTTTATTGTGATGTGACTATGATGGATGTGACAAGGACCTCTATTATTCACT
GGGGAGTGTGGCCGTTAATGAAGCCATTAGCTGAGCTAAGGCAAGTGAAGAAAATAGGGAAATTACCATTATT
CTGAAGCCATTAAGTTGTAATCCACCACTTTCTCTCCTTATTCAAAAAGGCATCTTCTTTTTAAGGCCTCCCT
???CTTACTATGGTTATTTCATACTTCGGTTGCATGAAGGCTGCCCCCATGAAAGAAGCAAACGTCCGAGGAC
AAGGTGGCTTGGCCTACCCAGGTGTGCGGACCCATGGGACTCTGGAGAGTGTGAATGGGCCCAAGGCAGGTTC
AAGAGGCCTGACGTCATCATTGGCTGACACTTTCGAACATGTGATAGAAGAGCTGTTGGATGAGGATCAGAAA
GTTCGGCCCCATGAAGAAAACAATAAGGACGCAGACTTGTACACGTCCAGGGTGATGCTCAGTAGTCAAGTGC
CTTTGGAGCCTCCTCTTCTCTTTCTGCTGGAGGAATACAAAAATTACCTGGATGCTGCAAACATGTCCATGAG
GGTCCGGCGCCACTCTGACCCTGCCCGCCGAGGGGAGCTGAGCGTGTGTGACAGTATTAGTGAGTGGGTAACG
GCGGCAGACAAAAAGACTGCAGTGGACATGTCGGGCGGACGGTCACAGTCCTTGAAAAGGTCCCTGTATCGA
AAGGCCAATTGAAGCAATACTTCTACGAGACCAAGTGCAATCC??????????????????????????????
??TTCTTTGAGCAGATGCA
GAACTAGACCCCCCGCCACAGCAGCCTCTGAAGTTGGACAGCAAAACCATTGCTTCACTACCCATCGGTGTCC
ATTTATAGAATAATGTGGAAAGAAACAAACCC-
TCTGTCTTATGATTTACTCATTACCGCCTTTTGACAGCTGTGCTGTAACACAAGTAGATGCCTGAACTTGAAT

TAATCCACAGATCAGTAATGTATTCTCTCTCTCTCTCTCTCTCTCTCT-------------
ACATTTTGGTCTCTACACTACATTATTAATGGGTTTTGTGTACTGTAAAGAATTTAGCTGTTTC-
AAACCTAGTGCATGA-
ATAGATTCTCTCCTGATTATTTATCGCATAGCCCCTTAGCCAGTTGTATATTCTTCCTGTGGTTTGTGACCCA
ACTAAGTCCTACTTTAAATATGCTTTAAAGAATCGGTGGGG-A---
TGCTTCACGTGAACGTGGGAGTTTAGCTGCTTCTCTTGCCTCAGTATTCCCTTCCT-
GATCACTATGCATTTTAAAGTTAAA-CATTTTTAAGTATTTCAGATGCTTTAGAG--ATTTTTTTCC-C-
ATTATTGCATTTTATTGTACAGATTGCTGCTTCTGCTATATTTGTGATATAGGAATTAAGAGGATACACGTTT
G?????????????????????????TAATCTCACTTCCGGAGAGCACTAAGCCACTTCCAGCCCTGCTGTGA
ACCAGGACCGTAGGAGCCATCTTTTTCATGTGGTCAGCTTTGGAAGAATCTACATCCACCACTTTAGAAGATA
AGTCTAGTGGCTTATCTGTGACGTCTTTGGTAACAGTCTGCTTCTCCAACAGAGGAGGTGAGCTGCCGTCTTT
TCTGTCTTGAACCACTGTCTTCCGGGAATGCCCAGGCACTGGCTGGGCGCCTTCGCCCCCTTCTGGAGCCTTG
GGATACTTGCCACTGGAGAGCCTGGCCACGGGGAACTCGCTGCTGACTGTCATGTATGGCTTTGACAGGGCAA
CTGAAGGAGAGGTGGAGATCCTGGCATAGTGCTTGTGGAACTCCGAGTAGGTGTCTGCAGCAGGCTGGGTGGG
AAGGTGGACCCGGGGTGAGGGCCGAGGCGAGGGGGGCAACAGGAGAGCCGTGTCCCCCGGCAGGCCACTGGTG
ACTGCCTTGGCAGAGGGAACCCTGGGCTGTTTACTGTTCTGGATGTGAGGATAGGCATGGGAATCAACAGGAT
TCCCAGGGCTGACCCCCATCTTCCATGGGAGGCTTTTGTCTGCGCAATGGACAAGAGGTGGGATGGCTGGGGA
GGCCGAAGGCGTCGAAAGCCTCATTGGTGATGCTAAGGACGATGGGATGTGGGGACTGACGTAGTGAGGTGGT
GGCAGGTAGAGAAAACGCTCCCCATTGGTGCAGACTGGAGAATACAGCGGCTGGGCCAAGCTGTAGGACTGCT
GAGGTAGCAAGGTGCCAGGACCTTTGTAATTCAAGTGCTTTTTACAGTAGTAGCAAATGTTACATAAGCCCAA
ACATGGAGCA---
TATTTGGAGTTGGTGGGCAGACCCAAATCATGATGCAAGCCTGCAGTAGCAAGTGTGCAATGAAGCACAACCA
AATTCTGTGAATGGAAGGTAGCGAGACGCCTCTCCAAAGCAGCCCTTCTGCGCAGGAGCTGAAAGCTCTACCG
GGTAAGTTT-
CTGAGGCAGTTTTCAGAGGCTGGGCTTGGCTCAGGGCCCAGCTCTGGATGAGCCTGTGGATGGTGGGGT----
AGGTCTTTGCTAACCTTACATAGAACCAAGGAGAGTTTG-
AAGAGTTTGTGCAGGCCTCTTTCCTCCTGGTGTTTGTCTTGCCCTGCCAGCAGGCCAGGAGGG-
CTGCGCTTCTCAAGTAGTGGAGGGAGGGAGCCTGCAGAGCTAGGGGATCAGAGTGGGTAATTGCAGCCACAGG
GATTAGCTGCTGCAAGCTGTCCACAGGAGCCATTCTGAAATGTCATTTGAGTGATCTGGCCGTCTACTGGAGG
AGGCCCTTGGGGCAGCTGTCTCCAGGAGCAGCTTCTGAGGGAGTCTTCCAGGAGGAGCAGCGTCTCTGCCATC
GTCCTCGTCCACGTCCTGGTCACATTTCCATTCCTCATCTTCATTCAAAGTCGGCAGGTATCCAGATATGCCC
TTCCCTGTCCCTGACCCAGAGCTCTGGTCACTACAGACCTTGGGGCTCTCCGAGCCTGTCCTCGTATATTCCA
AATAAATATCAGACTTAAGGAAGGAGGGGTAGGTGTTTTCCTCCATGGTGGCCTGGATTTCTGTCTGGGCCTG
GTCAAACATAGCGGGATCGATCAGCTGCTTCATGATGCAGCCCTTTATGAAGCTCTTGGTGGCCGGCTTGGTC
TGCCGGGACACGATGCCATTGTTATCAAGAATGTACTTTCGGTAGATGGCTCTGGCCAGCTTCAGCCTCTTCT
CCTCGTTGGAGTCACAGGGCTCCAGCTTCCTGAAGCCAGTGCAGGCAAACCAGAAGTCCAGCAAGTCAGCACA
GCCCTCTTGCTTCAGGAAAGTCCTGAACAGGCTTATCCCATCTTGATCATCCAGCAGGGAGTGCAGTGACTCA
GCCCACTTCAAGTACGGTGGGGTGGGGGAGGCACTGCCCTCAGGCTCATACCCCAGGTCCAGATCCGAGCGCC
TCGGAGTGGCCGTCGAAGTCTCACCTTTAATGCCAACACCTTTCCCGGAGCAGAAACTGTAGCTGGCAGGCCT
CAGGTCTGTGGACACCAGTTCTCCCTCCTCACCAGGCACTGGGGGTCGGGGAGCATCTTCGGTGAAACTTGCT
CCAAGGTCCAAGGGGAAACCCTGCTCCTGGACATTCATTTGGGACTCTGTGCGTCAATGCACAACGAGCGCT
GCACCCTAATACATCAGTACTTAACAGCTCCAAAGTCAATCAATCTGTCCTGTTGAAACCAT????TCTAAAA
TGGTAGCTCATTTTAAGTGAACAA-A--
ATTACGTGGATCGACTGGTGGCAGCATGAAACAGGCATAATTTACAGTGCAGCAGGATAAGT-
ACTTAGGTTATTTATAGGACAGATTATTTTTCTGTGTTGTTTTG----
TGTCTTTGGCATATTAAAATTTATTTATAGGTGTAGTTAAGCTTT--
ACTATTAGTTAGCATAAATTTGGCATGGAGTCA----
CCAAAGTAGAAATGTTGGACTTCATTTTTATGAGTTATTTCCTACACATTTCTAAACTGATGTAGCTTTGTCA
CTGCTCGATTGATTTTTTTTTTTAAAGTGGAGGGGGCAAAATTAGTTCATACTGAATCATACCAGTTTCTCTT
AGTTAGTA-------------------
CATTACTAACATTGAAAAGAAGTAAAAGGAATGTTTATAACTTAAGAGTATTTGCATAGTTAACGTTCAGAAC
GCAAGCTTTAAATTAGTTGAGGAAACCA---------
GAAGCTTGATTTAAGCACTCATACGGCTTTATTTTCCTTTGTTAGCAATAATTATTGGACCTGATGGCCATCC
TTTGACTGTTTATCCTTGTATTATTTGTGGGAAGAAGTTTAAGTCAAGAGGTTTTTTGAAAAGACACATGAAA

AACCATCCCGAACATCTTGCCAAGAAGAAGTACCACTGTACTGACTGTGATTACACTACCAACAAGAAGTTAA
GTTTACACAACCACCTGGAAAGCCACAAACTGACCTGCAAGGCAGAGAAGGCCAATGAATGTGATGAGTGTGG
GAAGCATTTTTCTCATGCAGGGGCTTTGTTTACTCACAAAATGGTGCACAAAGAAAAGGGAGCCAACAAAATG
CACAAGTGTAAATTCTGTGAATACGAGACAGCTGAACAAGG??????????????????????????????????
??????????????????TACCCGCTATAGCTTCTGCAAACAGTAAAGATACCACTAAAACCCCTTCTGCAGT
TCAAACTAGCACCAGCCACAACCCTGGACTTCCTCCTAATTTTAACGAATGGTACGTCTGAGGAAAAACACAA
ACCCTGTTAACTATAGAATGGACCAAAC------------------
TGAGACCAATCAGATGGAAATGGAGTTTTAAGGCAAGAGGCCATATATAGGGCTACATCTTGTTAATTGCAAT
TGTCCAGGAAGGTTTTGGGCAAGATCCAAAAGTAGCCATGCCCTTTTCTCAGGATTAGAAAATATGTTTTGGC
GTTTGAAGGATTTTTT--
ACAAAATCTTTACACTGCTTTTTCTTCCCCTTCCTCTTGCTCTCTGCACACCCCATTCCTAAACTCCTGCAAT
TCAATTTAACACTTGTCCTGTTTCTTGAGAGGAAGTTATACAAGGCTTGTTGGTGGTGGTGATGTTAAACTGA
TGGAAATTCTTTTTCGCCTTAGTGGTAATTGTTTAAACTCTCACAGTCTTAAACCGTGCCAAAGTCCTGTTAT
?
Miopithecus
??ACCCCCATGTTTGGCTGGAACATGAAACTGACCTCAGAGTACCACAGAAATGTCACCTTCCTTTCATGCCA
ATTCCTTT-
CTGTCATGAGAATGGACTACATGGTATACTTCAGCTTCTTCACCTGGATTTTCATCCCCCTGGTAGTCATGTG
TGC--CATCTATCTTGACATCTTTTACAT-
CATCCGGAACAAACTCAGTCAGAACTTCTCTAACTCCAAAGAGACAGGTGCATT---
TTATGGACGGGAGTTCAAGACAGCTAAGTCCTTGTTTCTGGTTCTTTTCTTGTTTGCTCTGTCATGGCTGCCT
TTATCCATCATCAACTGCATCATCTACTTTAA--------------
TGGTGAGGTACCACAGCTTCTGCTGTACATGGGCATCCTGCTGTCCCATGCCAACTCCATGATGAACCCTATC
?????????????????????ATGTTTATTAACCGTTGATTCTTTTCAACAAACCACAAAGACATCGGAACTC
TGTACTTACTATTCGGTGCATGGGCTGGAGTTATAGGAACAGCTCTAAGCCTCCTCATCCGAGCTGAACTGGG
CCAACCCGGTAGTTTGTTAGGTAATGATCATATTTATAATGTTATTGTAACAGCCCACGCATTTGTCATAATT
TTCTTTATAGT---TATACCCATTATAATTGGGGGGTTCGG---
AAACTGACTAGTTCCTTTAATAATTGGTGCTCCTGACATAGCATTCCCCCGTATAAACAACATAAGCTTCTGA
CTGCTTCCTCCC--TCCTTCCTACTACTAATAG-
CATCAGCCATAGTAGAAGCTGGTGCTGGAACAGGTTGAACGGTATACCCCCCTCTAGCGGGAAATCTCTCTCA
CCCGGGAGCCTCTGTAGATTTAGTTATCTTCTCTCTCCACCTAGCAGGAATCTCCTCTATCCTAGGAGCTATT
AATTTCATCACCACCATTATTAATATAAAACCCCCCGCAATATCTCAATATCAAACCCCTTTATTTGTCTGAT
CAGTCCTAATTACAGCCATTCTCCTACTTCTTTCTTTACCAGTCCTAGCTGCCGGCATTACTATACTACTAAC
AGATC--
GCAACCTTAACACCACTTTCTTTGATCCAACTGGAGGGGGAGATCCCATCCTATACCAACACTTATTTTGATT
TTTTGGCCATCCTGAAGTCTATATCCTCATTCTCCCTGGATTTGGAATAATCTCTCACATTGTAACCTATTAT
TCTGGAAAAAAAGAACCATTTGGGTATATGGGTATGGTTTGAGCTATAATATCAATCGGGTTCTTAGGTTTTA
TTGTATGAGCCCATCACATATT-
CACAGTCGGCATAGATGTGGACACACGTGCCTATTTTACTTCTGCCACCATAATTATTGCAATCCCCACTGGA
GTTAAAGTTTTTAGCTGACTTGCCACACTTCACGGAGGCAACATCAAATGATCCCCCGCAATACTTTGAGCCC
TAGGCTTTATTTTTCTATTCACTATGGGAGGCCTCACCGGCATTATCTTAGCAAATTCATCATTAGACAT-
TGTATTACACGATACATACTATGTTGTCGCCCATTTCCATTATGTTTTGTCAATAGGAGCTG-
TCTTTGCCATTATAGGGGGCTTTATCCATTGATTCCCCTTATTTTCGGGCTACACACTAAACCAAACATGTGC
CAAGGCACACTT-
TATTATCATATTCGTGGGTGTAAATTTAACTTTCTTCCCACAACACTTTCTTGGCCTATCCGGAATACCTCGA
CGCTACTCTGATTACCCTGATGCTTACACCACATGAAACATTCTATCATCCACCGGCTCCTTTATCTCACTAG
TAGCAGTAATCCTAATAATCTACATAATCTGAGAAGCTTTTGCTTCAAAACGCAAAGTGTTAATAATTGAACA
ACCCTCAACTAACCTCGAATGGCTAAATGGCTGCCCCCCACCTCACCACACATTTGAAGAACCAGTCTATATT
AAACTGAACGAAAAAGGAGAGAGTCGAACCCCCTAAATGGCCCATCCAGTTCAACTAGGTCTACAAGATGCCA
CATCTCCCATTATAGAAGAATTAATCACTTTCCACGACTACGCCTTTATAACCATTTCTCTAATTAGTTTCCT
GGTCTTATATGCCTTATCCTCAACACTTACAACAAAACTAACCAACACCAACATCACAGATGCCCAGGAAATA
GAAACCATCTGAACTATCTTACCCGCAATTATCTTAATTCTGACTGCCCTTCCATCCCTACGCATCCTATATT
TAACAGACGAGATCAACAACCCCTCCTTTACTATTAAATCAATCGGGCACCAATGATATTGAACCTACGAATA
CACAGACTACGGAGGCCTTATTTTCAACTCCTACATATTACCTCCCCTGTTCTTAAACCCAGGAGATCTCCGA

CTTCTAGAAGTTGACAATCGAGTAGTTCTCCCAATTGAAGCTCCTGTTCGTATAATAATTACGTCTCAAGATG
TTTTACACTCATGAACCATCCCCACGCTAGGCTTGAAAACAGATGCAGTACCTGGTCGCCTAAACCAGACCAC
ATTTACCGCCATACGACCAGGCGTCTATTACGGACAATGCTCAGAAATCTGCGGCGCTAACCACAGCTTTATA
CCCATTGTCGCAGAATTAATCCCACTAAAAATTTTCGAAATAGGG--
CCCGTATTTACTCTATAA????????ATGACCCCAATACGCAAATCCAACGCAATCATAAAAATAATCAATCA
TTCCCTCATTGACCTACCCACCCCACCCAACATTTCCGTATGATGAAACTTCGGCTCACTCCTCGCATTCTGC
CTAACCCTACAGATCATCACAGGGTTATTCCTAGCAATACACTACTCACCAGACACCTCTTCTGCCTTCTCCT
CAATCGCACACACTACCCGAGATGTAAACCATGGCTGAATGATTCGCTATCTCCACGCTAATGGGGCCTCCAT
ATTCTTCATCTGCCTCTTTTTACACATAGGCCGAAACCTCTACTACGGCTCATTTCTCCTCCTAAAAACCTGA
AACACCGGCATCATACTCCTACTCACAACCATAACAACAGCCTTTATGGGTTATGTACTCCCATGAGGCCAAA
TATCATTCTGAGGGGCAACAGTAATCACAAACCTACTATCAGCCATCCCATACATCGGAACAGACCTTGTTCA
ATGAGTATGAGGAGGGTATGCCATCGACAACCCCACTCTCACACGATTCTTTACCCTACACTTCATCCTACCC
TTCATTATCGCCGCCCTAACGACCGTCCATCTGCTATTCCTACACGAAACGGGATCAAACAACCCCTGCGGAA
TTTCCTCCAACCCAGACAAAATCACCTTTCATCCCTACTATACAATCAAAGACATCCTAGGCCTAGCCATCCT
ACTCCTAATCCTAATAACACTAACCCTATTCACACCCGACCTCCTAAATGACCCAGACAACTACACCCCAGCC
AACCCACTAAACACCCCTCCACATATTAAACCAGAGTGATACTTTCTCTTCGCATATGCAATCCTACGATCCA
TCCCTAATAAACTAGGGGGCGTGCTAGCACTCTTTCTATCAATTTTTATCCTAACAATTATCCCCATACTCCA
CAAATCCAAACAACAAAGCATAATATTCCGCCCGCTTAGTCAATTCCTATTCTGATTCCTGGTTACAACACTA
CTAACCCTTACTTGAATCGGAAGTCAACCAGTAAGCCAACCTTTCATTATTATCGGCCAAATAGCATCCACAA
TATATTTCACTACAATCCTAATCTTAATACCACTAGCCTCCCTAATCGAAAATGACTTACTTAAATGAACCTT
TAGGTTAAATTTATTTAAGAGAATGAGAATTTATGGGTATAACTGTTAGCTGGAAAGCCCTTCCTCACCTCCA
AGCCTCATTTCAAACTCTGTTTCC-
TGCTGAAATTTCTCAGTGGGATGACATGTTCAATTATTAGTCAATCCGTC----
TCTTATATGTTATCTGGCTTTACATAT----
GCCCTTGATGGTTCTTGGAAGATAATCTAATATCGTGTTGTTTTGGTGTCTTTGTATTGGAAATGGAAGCATT
TTGTATGAATCACCTCCTTAACTGTGAGGAGTTGAATTGTCTTAGGTCTGCTTGTCACATGACCGGGAATAAA
--
GACATCACAGTCTCCAACCCCACTTTTTTGGTGGAAAGAAAAGCTAAAAGTAGCAACATTTAATGGAACAAAA
TATCAGTAATCGAAGAAACTCCCCTGTGAAACTCTAATATTATTTT-
AAAACCAACATTGTTCTAAGGTAGCTGAAATGTATGATTTTTCTATTTTGCCTTTTCACTGACAATATACCCA
TAAATCT-
TGGTGGTGATTTTCTTTCCAAAGTTAATTTCTGGGCCATTGTTGACAGCACTATCTATTGCTCTAAATTGTTG
TCTTAACTGCATTCTTTACAAATGTAATGTTGAAGGCACATAATATTGAAGATGACTCCAGGATGAACCCTGA
GTTTGCAGACCGAA-
TAAAACAGCTAGATAAAGAGGCGTCTTACTACCGCGACGAGTGTGGCAAGGCCCAAGCAGAAGTGGACCGGTT
GCTGGAGA?????????GGAAGAGCTGCATTAGGCAGGTACTTTGATCTTTCTTGGAGCAGGTTCCTTCATAA
CGCTTCTCAAGAGGACGGAAGTCCCTCCAGTCTGGCTCGCTGCTCGCAGTGGTAGGAAAAGCTGAGGTAATTC
CCCGGTGGGAAATCTGAGAGGTCCCTGATAGCCCTG--------
CTGCCAGGCCCATCACTGATGGGCCTAAAGGCCTCATCTGATACTCTAAGACGGGCTTCTCCTGCCGGGCCTG
GGCCTCTCGGACTTGAGTCTCTCTAGAACGAGCCTCTCGGGCCTGGGTTTCTCGGGTTCTGGCCTCCCTGCCA
CGAGCTTCCCAAGTGTGGACCTCCCTG--
GCATAGGCTTCCCTGCCATGAGACTCTCGAGTATAGGCCTCCCTGGTGTGGGCCTCCCTGGCATGAGCTTCTC
GGGCACGTGCCTCCTGGGCCTCAAGCTGCTCCCGCCGAAGCTCCCAATACAACAACTGTTTCTGGATGGTCA-
CTAGCCGTTCTTCCTCTGTCTCCATTGCCCCAGGTGGCCGGGAGGAAGAAAAGGGCTCAAAGTTTAAGAAGGG
GTCAAACATCTCAGAGCTTCGACCATGGAGGTCATAAAGGCAGTCATCTCCAGGTGGAGAGTTCTCAAGGCTG
TCATCTGGCTCATAGAACTCATATAGGGCATCTCCACTGTAACTGTCTCGGGGTAGACAATCCCTGCGGACAA
GCCCCAGGGCCTCACCTGAATCATCCTCAA??????AGCATCACTAATTTTGATGAACTAAAAATGGTTCCTG
AAGGTCAGATTCATGTACAGTGAACGTTCTCCACTGTTGTTGTGGGCATGTTTCATGTGTGCGGATGTTGATG
TGCAAGTGTCGTGTGTTTGGAAAACACTAGCTAGAACATGCGAAAAATTTCATTAACCCAGCTCTGGAAGGTC
ATCCATGAATTCTTCCTATTCCAGTGGCCTTTT-
AAGTTTAGATGTAGATACACTGTAATTTAAAACAGATGGGCATGTCTGAATATTTTCATATGCAAAATGGGAC
AGATGTATATGAATGGATAAATTATCCTTTTAAAAAGATAATTTGTAGCACGAAATATCACCAATGATTGGAA
CAGTGAACTTGCATTCAGTCTAGGGCAAACACCAGGAAGACATTATCAAAACATCTAA-
CCATCTACAAATTAATTCCAATTAGTCTACTTTGTCTTCATACAGAAGAAA-CTTTTCTGATTTTTAAA---

TATCTACTGGGTGCCAATCGGTATCCTTT-
GAGCTGCTAAAGCCAGCGTATCACAATAGAAGTTTACTTTTCCTTGAGAGTTGCATC????????????????
???
???
???
???
???
???
???
???
???
???
???
???
???
???
???
???
???
???
???
???
???
???
???
???
???
???
???
???
???
???
???
???
???
???CCCAAGACCTTGATGA
TTACCTGAATGGCCCCTTCACAGTGGTGGTGAAGGAGTCTTGTGATGGAATGGGAGATGTGAGTGAGAAGCAC
GGGAGTGGGCCAGTAGTTCCAGAAAAGGCAGTCCGTTTTTCATTCACAATCATGAAAATTACTCTCACCCACA
GCTCTCAGAATGTGAAAGTATTTGAGGAAGCCAAACCTAACTCTGAACTGTGTTGCAAGCCATTGTGCCTTAT
GCTGGCAGATGAGTCTGACCACGAGACCCTGACTGCCATCCTGAGCCCTCTCATTGCCGAGAGGGAGGCCATG
AAGAGCAGTGAATTAATGCTTGAGCTGGGAGGCATTCTACGGACTTTCAAGTTCATCTTCAGGGGTACCGGAT
ATGATGAAAAACTTGTGCGGGAAGTGGAAGGCCTTGAGGCTTCTGGCTCAGTCTACATTTGTACTCTTTGTGA
TGCCACCCGTCTAGAAGCCTCTCAAAATCTTGTCTTCCACTCTATAACCAGAAGCCATGCTGAGAACCTGGAA
CGTTATGAGGTCTGGCGTTCCAATCCTTACCATGAGTCTGTGGAAGAACTGCGGGATCGGGTGAAAGGGGTCT
CAGCCAAACCTTTCATTGAGACAGTCCCTTCCATAGACGCACTCCACTGTGACATTGGCAATGCAGCTGAGTT
CTACAAGATCTTCCAGCTGGAGATAGGGGAGGTGTATAAGAATCCCAATGCTTCCAAAGAGGAAAGGAAAAGG
TGGCAGGCCACATTGGACAAGCATCTCCGGAAGAAGATGAACCTCAAACCAATCATGAGGATGAATGGCAACT
TTGCCAGGAAGCTCATGACCAAAGAGACTGTGGATGCAGTTTGTGAGTTAATTCCCTCGGAGGAGAGGCACGA
GGCTCTGAGGGAGCTGATGGATCTTTACCTGAAGATGAAACCGGTGTGGCGGTCATCATGCCCTGCTAAAGA-
-GTGCCC-
AGAATCCCTCTGCCAGTACAGTTTCAATTCACAGCGTTTTGCTGAGCTCCTTTCTACCAAGTTCAAGTATAGG
TATGAGGGAAAAATCACCAATTATTTTCACAAAA???????????????????????????????????????
???

???
???
???
???
???
???
???
???
??ATG
GAGGGAAAACACCAAACAATGAGCTTTCAGATAAGATTTATGTCATGTCTATTGTTTGCAAGAACAACAAAAG
GGTTACTTTTCACTGCACAGAGAAAGACTTGGTAGGAGATGTTCCTGAAGCCAGATATGGTCATTCCATTAAT
GTGGTGTATAGCCGAGGGAAAAGTATGGGTGTTCTCTTTGGAGGACGCTCATACATGCCTTCTACCCACAGAA
CCACGGAAAAATGGAATAGTGTAGCTGACTGCCTGCCCCATGTTTTCCTGGTGGATTTTGAATTTGGGTGTGC
TACATCATACATTCTTCCAGAACTTCAGGATGGGCTATCTTTTCATGTCTCTATTGCCAAAAATGATACCATC
TATATTTTAGGAGGACATTCACTTGCCAATAATATCCGGCCTGCCAACCTATACAGAATAAGGGTTGATCTTC
CCTTGGGTAGCCCAGCTGT-
GAATTGCACAGTCTTGCCAGGAGGAATCTCTGTCTCCAGTGC???????????????????????????????
???
???
???
???
???
???
???
???
???
???
???
???
???
???
???
???
???
???
???
???
???
???
???AAGGCCCTCA
AAGATCTTTATTGCCATAAAGTAGAGTAACT-GTCTTTGACTTGGACCTTGTTTACTGTGAACTCTAATC-
AAGGCAGGAGATGCAGCAGTCCTACTTA-
TTGCCATGTGGACTTGTGGAAAGGACACGTGTGACCCTTAAGAATCCAGTTCGGATTAGTGTTTGAAATTGAA
TTGGGAATGTTGTTCCAGGATGTGGAATGCAGCTGTGATCACACTT----ACC-
GAGCTTACTTTGACCTGTTTGTCAATAGCATGCAAAAAA-
TGCTTTGTTTGCCCTTTGCTTCTGCTTTTTTCAGGGAAGCTGCCAAAGAATGTCGACGTCGAAAGAAAGAATA
TGTAAAATGTCTGGAGAGCCGAGTTGCAGTGCTGGAAGTCCAGAACAAGAAGCTTATAGAGGAACTTGAAACC
TTGAAAGACATTT??
???
???
???
???
???
???

??
??
???TGTTTATATCTAAATGTATGGATTCCG
GCACCTAAACCAAAAAATGCTACTGTAATGATATGG-
ATTTATGGTGGTGGTTTTCAGACTGGAACATCATCTTTATATGTTTATGATGGCAAGTTTCTGGCTCGAGTTG
AAAGAGTTATTGTCGTGTCAATGAACTATAGGGTGGGTGCTCTTGGATTCTTAGCTTTGCCAGGAAATCCTGA
GGCTCCAGGGAACATGGGTTTATTTGATCAACAGTTGGCTCTTCAGTGGGTTCAAAAAAATATAGCAGCCTTT
GGTGGAAACCCTAAAAGTGTAACTCTCTTTGGAGAAAGTGCAGGAGCAGCTTCAGTTAGCCTGCATTTGCTTT
CTCCTGGAAGCCATTCATTGTTCACCAGAGCCATTCTACAAAGTGGATCCTCTAATGCTCCTTGGGCAGTGAC
ATCTCTTTATGAAGCTAGGAACAGAACATTGACCTTGGCTAAATTGACTGGTTGCTCTAGAGATAATGAGACT
GAAATAGTCAAGTGCCTTAGAAATAAAGATCCCCACGAAATTCTTCTGAATGAAGCGTTTGTTGTCCCCTATG
GGACTCTCTTGTCAGTAAACTTCGGTCCAACCATGGATGGTGATTTTCTCACTGACATGCCAGACATATTACT
TGAACTTGGACAATTTAAAAAAACCCAGATCTTGGTGGGTGTTAATAAAGATGAAGGGACAGCTTTTTTAGTC
TATGGTGCTCCTGGCTTCAGCAAAGATAACAATAGTATCATAACTAGAAACGAATTTCAGGAAGGTTTAAAAA
TATTTTTTCCAGGCGTGAGTGAGTTTGGAAAGGAATCCATCCTTTTTCATTACACAGACTGGGTAGATGATCA
GAGACCTGAAAACTACCGTGAGGCATTGGATGATGTTGTTGGGGATTATAATATCATATGCCCTGCCTTGGAG
TTCACCAAGAAGTTCTCAGAATGGGGAAATAATGCCTTTTTCTACTATTTTGAACACCGTTGTGCTAATTGTG
AGAAAATATGCTACTTTGATCAAACTAGAATGAAGGAAATAAGAAGACACTGTGGCTCAACTTGTAATAAAGA
GCAGCTCATCCTCACAGCTTTGTGATTGTTGCTATAGTTATCT---
ATTCAACATGGGATGCACAATACAGTTAACGTTTCATTACCAGTAGCCTGTTGCGTGCTTATTGTTAAAAGGA
ATTTAACATACAAGTTTTTGTATGGGTTGTTCGCCTAATTCTGATTCCTCGTGCTGACTGGCATGAGACAGGG
AGCTGGATTGTATTAAACTGGTTTTATTGTGATGTGACTATGATGGATGTGACAAGGGCCTCTATTATTCGCT
GGGGAGTGTGGCCGTTAATGAAGCCATTAGCTGAGCTAAGGCAAGTGAAGAAAATAGGGAAATTACCACTATT
CTGAAGCCATTAAGTTGTAATCCACCACTTTCTCTCCTTACTCAAAAAGGCATCTCCTTTT-
AAGGCCT????????????????????????????????GCATGAAGGCTGCCCCCATGAAAGAAGCAAA
CATCCGAGGACAAGGTGGCTTGGCCTACCCAGGTGTGCGGACCCATGGGACTCTGGAGAGCGTGAATGGGCCC
AAGGCAGGTTCAAGAGGCCTGACAT---
CATTGGCTGACACTTTTGAACACGTGATAGAAGAGCTGTTGGATGAGGACCAGAAAGTTCGGCCCAATGAGGA
AAACAATAAGGACGCAGACTTGTACACGTCCAGGGTGATGCTCAGTAGTCAAGTGCCTTTGGAGCCTCCTCTT
CTCTTTCTGCTGGAGGAATACAAAAATTACCTGGATGCTGCAAACATGTCCATGAGGGTCCGGCGCCACTCTG
ACCCTGCCCGCCGAGGGGAGCTGAGCGTGTGTGACAGTATTAGCGAGTGGGTAACGGCGGCAGACAAAAAGAC
TGCAGTGGACATGTCGGGCGGGACGGTCACAGTCCTTGAAAAGGTCCCTGTATCGAAAGGCCAACTGAAGCAA
T???
????????????AACGGCTATGAAAATCCAACCTACAAGTTCTTTGAGCAGATGCAGAACTAGACCCCC-
GCCACAGCAGCCTCTGAAGTTGGACAGCAAAACCATTGCTTCACTACCCATCGGTGTCCATTTATAGAATACT
GTCGGAAGAAACAAACCCCTCTGTTTTATGATTTACTCATTATCGCCTTTTGACAGCTGTGCTGTAACACAAG
TAGATGCCTGAACTTGAATTAATCCACAAATCAGTAATGTATT----CTCTCTCTCT----TT----------
--------ACATTTTGGTCTCTACACTACATTATTAATGGGTTTTGTGTACTGTAAAGAATTTAGCTGTATC-
AAAC-TAGTGCATGA-
ATAGATTCTCTCCTGATTATTTATCACATAGCCCCTTAGCCAGTTGTATATTATTCTTGTGGTTTGTGACCCA
ATTAAGTCCTACTTTACATATGCTTTAA-GAATCGATGGGGGA---
TGCTTCATGTGAACGTGGGAGTTTAGCTGCTTCTCTTGCCTAAATATTCCTTTCCT-
GATCACTATGCATTTTAAAGTTAAA-CATTTTTAAGTATTTCAGATGCTTTAGAGAGATTTTTTTTTC-
CATGATTGCATTTTACTGTACAGATTGCTGCTTCTGCTATATTTGTGATATAGGAATTAAGAGGATACA????
?????????????????????????????????????GGAGAGCACTAAGCCACTTCCAGCCCTGCTGTG
AACCAGGACTGTGGGAGCCATCTTTTTCATGTGGTCAGCTTTGGAAGCATCTACATCCACCACTTTAGAAGAC
AAGTCTAGTGGCTTATCTGTGACATCTTTGGTAACGGTCTGCTTCTCCAACAGTGGAGGTGAGCTGCCGTCTT
TTCTGTCTTGAACTGCTGTCTTCCGGGCATGCCCGGGCACTGGCTGGGCACCTTCGCCCCCTTCCGGAGCCTT
GGGATACTTGCCGTTGGAGAGCCTGGCCGCGGGGAACTCGCTGCTGACTGTCATGTATGGCTTCGAGAGGGCA
ACTGAAGGAGAGGTGGAGATCCTGGCATAGTGCTTGTGGAACTCCGAGTAGGTGTCTGCAGAAGGCTGGGTGG
GAAGGTGGACTCGGGGTGAGGGCCGAGGCGAGGGAGGCAACAGGAGAGCTGTGTCCCCCGGCAGGCCACTGGT
GACCGCCTTGGCAGAGGGAACCCTGGGCTGCTTACTGTTCTGGATGTGAGGATAGGCATGGGAATCAACGGGA
TTCCCAGGGCTGACGCCCATCTTCCACGGGAGGCTTTTGTCTGCGCAATGGACGAGAGGCGGATGGCTGGGG
AGGCCGAAGGTGTCGAGAGCCTCATGGGTGATGCCAAGGACGATGGGATGTGGGGACCGACGTAGTGAGGTGG

TGGCAGGTAGAGAAAGCGCTCCCCGTTGGTGCAGACTGGAGAATACAGCGGCTGGGCCAAGCTGTAGGACTGC
TGAG??????????????GGACCTTTGTAATTCAAGTGCTTTTTACAGTAGTAGCAAATGTTACATAAGCCCA
AACATGGAGCA---
TATTTGGAGTTGGTGGGCAGACCCAAATCATGATGCAAGCCTGCAGTAGCAAGTGTGCAATGAAGCACAACCA
AATTCTGTGAATGGAAGGTAGCAGGACGCCTCTCCAAAGCAGCCCTTCTGCGCAGGAGCTGAAAGCTCCACTG
GGTAAGTTTTCTGAGGCAGTTTTCAGAGGCTGGGCTTGGCTCAGGGCCCAGCTCTGGATGAGCCTGTGGACGG
TGGGGT----
AGGTCTTTGCTAACCTTACATAGAACCAAGCAGAGTTGGGAAGAGTTTATGCAGGCCTCTTTCCTCCTGGCGT
TTGTCTTGCCCTGCCAGCAGGCCAGGAGGG-
CTGCGCTGCTCAAGTAGTGGAGGGAGGGAGCCTGCAGAGCTAGGGGATCAGCGTGGTTAATCGCAGCCACAGG
GATTAGCTGCTGCAAGCTGTCCACAGGAGCCATTCTGAAATGTCATTTGAGT????????CGTCTACTGGAGG
AGGCCCTCGGGGCAGCTGTCTCCAGGAGCAGCTTCTGAGGGAGTCTTCCGGGAGGAGCAGCCTCTCTGCCATC
GTCCTCGTCCATGTCCTGGTCACATTTCCATTCCTCATCTTCATTTAAAGTCGGCAGGTATCCAGATAGGCCC
TTCCCTGTCCCTGACCCAGAGCTCTGGTCACTACAGACTTTCGGGCTCTCCGAGCCTGTCCTCGTATATTCCA
AATAAATATCAGACTTAAGGAAAGAGGGATAGGTGTTTTCCTCCATAGTGGCCTGGATTTCTGTCTGGGCCTG
GTCAAACATGGCAGGATCGATCAGCTGCTTCATGATGCAGCCCTTTATGAAGCTCTTGGTGGCTGGCTTGGTC
TGCCGGGACACGATGCCATTGTTATCAAGGATGTACTTTCGATAGATGGCTCTCGCCAGCTTCAGCCTCTTTT
CCTCRTTCGAGTCACAGGGCTCCAGTTTCCTGAAGCCAGTGCAGGCAAACCAGAAGTCCAGCAAATCGGCACA
GCCCTCCTGCTTCAGGAAAGTCCTGAATAGGCTTATCCCATCTTGGTCATCCAGCAGGGAGTGCAGTGACTCA
GCCCACTTCAAGTATGGTGGGGTGGGGGAGGCACTGCCCTCAGGCTCATACCCCAGGTCCAGATCCGAGCGCC
TCGGAGTGGCCGTCGAAGTCTCAGCTTTAATGCCAACACCTTTCCCAGAGCAGAAACTGTAGCTGGCGGGCCT
CGGGTCTGTGGACACCAGTTCTCCCTCCTCACCAGGCACTGGGGGTCGGGGAGCATCTTCGGTGAAACTTGCT
CCGAGGTCCAAGGGGAAACCCTGCTCTTGGATATTCATTTTGGGACTCTG--
CGTCAATGAACAATGAGCGCTGCACCCTAATACATCAGTACTTA-
CGGCTCCAAAGTGAATCAATCTGTCCTGTTGAAACCAT???????????????????????????????????
???
???
???
???
???
???
???
???
???
???
???
???
???TACCCGCTATA
GCTTCTGCAAACAGTAAAGATACCACTAAAACCCCTTCTGCAGTCCAAACTAGCACCAGCCACAACCCTGGAC
TTCCTCCTAATTTTAACGAATGGTACGTCTGAGGACAAACACAAACCCTGTTAACTATAGAATGGACCAAATG
CATTTTTAAAAGAAAACTGAGACCAATCAGATGGAAATGGAGTTTTAAGGCAAGAGGCCATATATAGGGCTAC
ATCTTGTTAACTGCAATTGTCCAGGAAGGTTTTGGGCAAGATCCAAAAGTAGCCATGCCCTTTTCTCAGGATT
AGAAAATATGTTTTGGCATTTGAAGCATTTTTT--
ACAAAATCTTTACACTGCTTTTTCTTCCCCTTCCTCTTGCTGTCTGCACACCCCATTCTTAAACTCCTGCAAT
TCATTTTAACAGTTGTCCTGTTTCTCGAGAGGAAGTTATAGAAGGCTTGTTGGTGGTGGTGATGTTAAACTGA
TGGAAATTCTTTTTCGCCTTAGTGGTGATTGTTTAAACTCTCACAGTCTTAAACCGTGCCAAAGTCCTGTTAT
A
Pithecia
?TACCCCCATGTTTGGCTGGAACATGAAGCTGACCTCAGAGTACTACAGAAATGTCACCTTCGTTTCATGCCG
GTTCCCTT-
CCGTCATGAGCATGGACTACATGGTATACTTCAGCTTCTTCACCTGGATTTTCATTCCCCTGGTCATCATGTG
TGC--AATCTATCTTGACATCTTTTACAT-
CATCCGGAACAAACTCAGTCAGAACTTCTCTAACTCCAAAGAGACAGGTGCATT---
TTATGGACGGGAGTTCAAGACGGCGAAGTCCCTGTTTCTGGTGCTTTTCTTGTTTGCTTTGTCATGGCTGCCT

TTATCCATCATCAACTGCATCACCTACTTTCA--------------
TGGTGAGGTACCACAGCTTGTGCTGTACTTGGG??
???
???
??ATTAACCCCCTAGCACACCTTATTATC
TCCTTTACAATTCCAACGGGAACTATCATTACAATCCTAAGCTCACACTGATTCCTCATCTGAATAGGCCTAG
AACTAAATATACTAT--CCATGATTC--
CAATATTAGCCAAAAATACAAACCCCCGATCCACAGAAGCAGCTACTAAATATTTTCTAACCC--
AAGCAACCGCATCCATAGTCCTACTAA--TAGCCATTATTC-
TAAACAACCTATTATACGGACAATGAACAATTAACCCATCCCTAAACCCAACCTTATCAACAATTATACTTAT
CGCCTT---
GACAATAAAACTAGGAATAGCCCCCCTACACTTCTGACTCCCTGAAGTAGTCCAAGGCATCCCCCTAATCCCC
GCTATACTTATCCTCACATGACAAAAACTAGCCCCCATATCAATTATTATCCAAATCTTCCCATCAATTAACA
TAAATATTATCCTAACAATCTCAATTCTATCCATTATAATT-GGCAGCTGAGGAGGACTAAACCAAACA----
CAATTACGCAAAATCCTAGCCTACTCATCAAT-
TACACACATAGGCTGAATAACAGCAGTACTATACTATAACCCAAGCATTACCATTCTC----
AGCTTAACTATCTACCTAT-TCCTAACAATCT---
CCACATTTGTAGTTTTCTACCTAAACTCAAATATAACAACCCTGTCAATATCATACACCTGGAACAAA-----
-CTCACATGAATAGTACCTAT-AATTACATTAATAAT-
ACTATCCCTAGGAGGCCTACCCCCACTAACAGGCTT-CTCCCCTAAATG--
AGCTATCATACTAGAACTAACAAAAAACAACAA--------
CCTAATTATTCCCCTTACTATAGCTATACTAAC---------
ACTAATAAACCTATACTTTTATATACGCCTCACATACTCCATTTCA-ATAACAAT--
ATTTCCCACATCCAATAACACAAAAATTAACTGACAACTAAAATATATAAAATCAT-
CACCACTTCTCCCTATACTCATAGTATCTTCTACCCTCCTCCTACCCTTAACCCCATTAATATTAACAAGCT?
???
???
???
???
??CAAAACGCTACATCCCCAATTAT
AGAAGAACTAATTGCTTTTCATGATCATGCCCTTATAATTATTTTTCTAATCAGCTCACTAGTTCTATATACT
ATCTCCCTAATACTAACTACAAAACTAACTCATACCAGCACTATAAACGCCCAAGAAATTGAGATAATCTGAA
CTATTCTGCCTGCCATTATTCTTGTTATAATTGCTCTCCCATCCTTACGTATCCTTTACATGACAGACGAGTT
TAATAAGCCCTACTTAACCCTTAAAGCAATTGGCCACCAATGATATTGAAGCTACGAATACTCAGACTATGAA
GACCTAGCATTTGACTCTTACATTATACCCACATATTTTCTTGAGCCTGGCGAATTTCGACTTCTTGAAGTAG
ATAACCGAACAACATTACCAATAGAAGCAGACATCTGAATATTAATCTCATCACAAGATGTACTACACTCATG
AGCTGTGCCATCACTGGGCATTAAAGCAGACGCAATTCCAGGACGCTTGAATCAAGCTATAGTAGCATCCATA
CGACCAGGTTTATTT??
???ATGACTACCCCC
GCAAAACACACCCCCTAATAAAAATTATTAATAACTCGTTCATTGACCTACCTACACCACCCAATATCTCATC
CTGATGAAACTTCGGCTCGCTACTAGGCATGTGCCTAATTATCCAAATCACTACAGGCCTATTCCTAGCTATA
CACTACACATCAGACTCTTCAACCGCCTTTTCTTCAGTTGCCCACATCACCCGAGACGTTAATTACGGCTGAA
TAGTACGCTATTTGCATGCTAACGGCGCCTCACTATTCTTTATATGCTTATTCCTCCACATCGGACGGGGCCT
ATACTATGGGTCCTTTCTCTTCTTAAGCACATGAAACATTGGCATCATCTTACTACTTACAACCATGGCCACA
GCCTTCATAGGATATGTGCTCCCATGAGGCCAAATATCTTTCTGAGGGGCTACAGTAATCACAAATCTACTAT
CAGCTGTCCCCTACATTGGACCAGATCTAGTACAATGAATCTGAGGTGGCTTTTCCGTTGATAAGGCAACTCT
TACCCGATTCTTTACCTTTCACTTTATTCTACCCTTTATTATCGCAGCCCTAGCAACTATCCACCTTCTGTTT
CTGCATGACACAGGTTCAAATAACCCATCAGGACTGACATCTGACCCAGACAAAATCACATTCCACCCATACT
ACACAATCAAAGATATCCTAGGCCTAGTATTCCTACTTCTACTCCTAATAAGCCTAACCCTATTTACACCCGA
CCTCCTAACTGACCCAGACAACTACACTCCTGCTAACCCTCTAAATACCCCACCCCATATTAAACCAGAGTGA
TATTTTCTATTTGCATACGCAATTCTACGATCCATCCCTAATAAACTAGGAGGCGTACTAGCCCTACTTCTCT
CCATTCTAATCCTAGCAACCATTCCCGCAACACACCTATCCAAACAACAAAGCATACTATTCCGACCAATCAG
CCAAATTCTATTCTGAGCCCTAGTAGCTGACTTATTTACACTTACATGAATTGGAGGCCAACCAGTCGAACAC
CCCTTTATTATCATCGGCCAAACCGCATCTACCCTATACTTCTCCATTATTAC---

CCTTATCCCCCTATTCGCTCTAATTGAAAACAAACTACTCAAATGATAG?TTAGGTTAAATTTATTCAAAGGA
ATGAGAATTTATGGGTATAACTGTTAGCTGTAAAGCCCTTCCTCACCTCCAGTCCTCCTTTCAGACTRCATTT
CC-TCCTGAAATTTCTCTGTGGGATGAAATGTTCAATTATTAGTCAATCAGTC----
TCTTACATGTTATCTGGCTTTACATAT----
GCCCTTGATAGTTCTTGGAAAATAATCCAATATTGTGTTGTTTTGGTGTCTTTGTATTGGAAATGGAAGCATT
TTGTATGAATCACCTCTTTAACTGTGAGGAGTTGAATTGTTTGAGTTCTGTTTGCCTAATGACTAGGAACAAA
--GACATCACAGTCTCTAACCCCACTTTTT-
GGTGGAAAGAAAATCTAAAAGTAACAACATTTAAGGGAACAAAATATCAATAATCAAAGAAACTCCCCTGTGA
AACTGTAATATTATTTT-
AAAATCAACATTGTTCTAAGGTAGCTGAAATGTGTGATTTTTCTATTTTGCCTTTTCACTGACAATATACCCA
TAAATCT-
TGGTGGTGATTTTCTCTCCAAAGTTAATTTCTGGGCCATTGGTGACTGCACTATCTATTGTTCTAAATTGTTG
TCTTAATTGCATTCTTTAAAAATATAATGTTGAAGGCACATAATATTGAAGATGACTCCAGGATGAACCCTGA
GTTTGCAGACCGAA-
TAAAACAGCTTGATAAAGAGGCGTCTTACTACCGTGATGAGTGTGGCAAGGCCCAAGCGGAAGTGGACCGGTT
GCTGGAGA?????????GGAAGAGCTGCATCAGGCAAGTACTTTGATCTTTCTTGGAGCAGGTTCCTTCAAAA
CGCTTCTCCAGAGGACGGAAGTCCCTCCAGTCTGGCTCACTGCTCGCAGTGGTGGGGAAAGCTGAGGTAATTC
CCCGGTGGGAAATCTGCGAGGCCCCTTTTACCCCTG--------
CCACCAGGCCCATCACTGATGGGCCTAAGGGCCTCATCTGATACTCTAAGACGGGCTTCTCCTGTCGGATCTG
GGCCTCTCGGACTTGAGACTCTCTAGAACGAGCCTCTCGGGCCTGGGCTTCTCGGGTTCTGGCCTCCCTGGCA
TGACCTTCCCAAGTGTGGGCCTCTCTG--
GCATAGGCTTCCTTACCATGAGCCTCTCGAGTATAGGCCTCCCTGGTGTGGGCCTCCCTGGCCTGAGCTTCTC
GGGCACGTGCCTCCCGGGCCTCAAGCTGCTCCCTCCGAAGCTCCCAATACAACAACTGTTTCTGGATGGTCA-
CTAGCCGTTCCTCCTCTGTCTCCATTGCCCCAGGTGGCCGGGAGGAAGAAAAGGGCTCAAAGTTCAAGAAGGG
GTCAAACATCTCAGAGCTTCGACCATGGAGGTCATAAAGGCAGTCATCCCCGGGTGGAGAGTTCTCAAGACTG
TCATCTGGCTCATAGAACTCATATAGGGCATCTCCACTGTAGCTGTCTCGGGGCAGACAATCCCTACTGACAA
GCCCCAGGGCCTCACCTGAATCATCCTCAA??????AGCATCACTAATTTTGATGAACTAAAAATGGTTCCTG
AAGGTCAGATTCATGTACAGTGAATGTTCTCCACTGTTGTGGTGGACGTGTTTCATGTGTGGGGATGTTGGTG
TGCAAGTTTCGTGTGTTCGGAAAACACTAGCTAGAACATTCAAAAAATTTCATTAACCCAGCTCTGGAAGGTC
ATCTATAAATTCTTCCTATTCCAGTGGCCTTTT-
AAGTGTAGGTGTAGATACACTGTAATTTAAAACAGATGGGCATGTCTGAATATTTTCATATGCAAAATGGGAC
AGATGTATATGAATGGATAAATTATCCTTTTAAAAAGATACTTTGTAGCACGAAATATCCCCAATCATTGGAA
CAGTGAACCTGCATTCAGTCTAAGGCAAACACCAGGAAGACATTATCAAAACATCTAA-
CTAACTACAAACCAATTTCAACTAGTCTACTTTGTCTTCATACAGAAAAAA-CTTTTTGATTTTTAAA---
TATGTATTGGGTGCCAATCCGTATCCTTT-
GAGCTGCTAAAGCCAGCATATCACAATAGAAGTTTACTTTTCCTTG?????????????????ATAATTAACC
TACTATTACTAATTATCCCAGCCCTAGCAGCCATAGCATTCCTAACTCTTATAGAACGAAAAATCCTAGGCTA
CATACAACTCCGCAAAGGCCCTAACATCGTAGGCCCCTATGGCCTACTTCAACCAATCGCAGATGCAATAAAA
CTATTTACAAAAGAAACCCTTCTACCCACTACATCCACCGTAACCCTATACGTTATAGCTCCAGCCCTGGCTC
TCTCCATCGCTCTTCTCCTATGAACCCCCCTCCCTATACCTCACCCTCTAATTAACCTAAACTTAGGCCTACT
ATTTATACTGGCAATATCAAGCATAGCCGTCTATTCAATCCTATGATCCGGATGAGCATCCAATTCAAACTAC
GCACTAATTGGCGCACTACGAGCAGTAGCCCAAACAATCTCATATGAAGTTACACTAGCCATTATTCTCCTAT
CAGTACTACTAATAAGCGGCTCATTCAACCTTCACTCACTTATTACAACACAAGAACACTCTTGACTACTATT
ACCATCATGACCCTTAGCCACAATATGATTTATCTCCACACTAGCCGAAACTAACCGAGCCCCCTTTGACCTG
ACAGAAGGCGAATCAGAACTAGTCTCAGGCTTTAATATTGAATACGCCGCAGGCTCATTCGCTCTATTTTTCA
TAGCAGAATATATAAACATTATCATAATAAACGCCTTAACTACTACTATCTTCCTAGCCGCACCCCACATCCC
AACTACACCAGAACTTTATTCAATTTACTTTATAATAAAAACCCTACTACTAACTACACTATTCTTATGAATC
CGAACAGCATATCCCCGACTTCGCTATGACCAACTTATACATCTACTATGAAAAAAATTCCTACCTCTTACAC
TAGCACTATGTATATGATATATCTCAATACCAACCCTAACATCCAGTATTCCACCCCAAACAT??ATTAACCC
CCTAGCACACCTTATTATCTCCTTTACAATTCCAACGGGAACTATCATTACAATCCTAAGCTCACACTGATTC
CTCATCTGAATAGGCCTAGAACTAAATATACTATCCATGATTCCAATATTAGCCAAAAATACAAACCCCCGAT
CCACAGAAGCAGCTACTAAATATTTTCTAACCCAAGCAACCGCATCCATAGTCCTACTAATAGCCATTATTCT
AAACAACCTATTATACGGACAATGAACAATTAACCCATCCCTAAACCCAACCTTATCAACAATTATACTTATC
GCCTTGACAATAAAACTAGGAATAGCCCCCCTACACTTCTGACTCCCTGAAGTAGTCCAAGGCATCCCCCTAA

TCCCCGCTATACTTATCCTCACATGACAAAAACTAGCCCCCATATCAATTATTATCCAAATCTTCCCATCAAT
TAACATAAATATTATCCTAACAATCTCAATTCTATCCATTATAATTGGCAGCTGAGGAGGACTAAACCAAACA
CAATTACGCAAAATCCTAGCCTACTCATCAATTACACACATAGGCTGAATAACAGCAGTACTATACTATAACC
CAAGCATTACCATTCTCAGCTTAACTATCTACCTATTCCTAACAATCTCCACATTTGTAGTTTTCTACCTAAA
CTCAAATATAACAACCCTGTCAATATCATACACCTGGAACAAACTCACATGAATAGTACCTATAATTACATTA
ATAATACTATCCCTAGGAGGCCTACCCCCACTAACAGGCTTCTCCCCTAAATGAGCTATCATACTAGAACTAA
CAAAAAACAACAACCTAATTATTCCCCTTACTATAGCTATACTAACACTAATAAACCTATACTTTTATATACG
CCTCACATACTCCATTTCAATAACAATATTTCCCACATCCAATAACACAAAAATTAACTGACAACTAAAATAT
ATAAAATCATCACCACTTCTCCCTATACTCATAGTATCTTCTACCCTCCTCCTACCCTTAACCCCATTAATAT
TAACAGCT????????????????????????????????GTTACTTAAGCAATTTACATTTACTGTATTAGT
AAGTAAATGCAATGGCCACTCTTATTCTCAAACAATAAAAACTACCCAGCCTTTTCATGAAAGAT-
GATTGCAATTTCCATTGGCCTGTATATGCAATAAAAAG---
CCATAACTCATCAAAATTTTAGCTATTTTYTCTTAAGATA--
GAAGAGCATTTTGCTTGAACATTGAACGTGACATTAAATATT-
CATGAAACTGAATAGAGTTTAAGTACTAAG---
TAAAACTCTCAAATTCTTTTCAACTAGATTGAAATTCTGTTTGTACCAGGTAAAAACTGCCGCTAACCTGAAG
AGGTGTTCAAAAAT-
GTCGTAAAGAGTTTGTACAATACGTTCAAAGTGCTACAGTTATTTGGGATAGATTTTTCTGAAAAGCCTGTTC
CTCCTTTGAGATTGATTCATGATAACAGCCTCCATTTGGGTCAAACAGTATTTAACCGCTTGTAAA-
TTTTAATT-
AAATGATTTGTCACTTTGAACACATAGTTTGTCCCCAAGACCTTGAGGATTACTTGAATGGCCCCTTCACGGT
GGTTGTGAAGGAGTCTTGTGATGGAATGGGAGATGTGAGTGAGAAGCATGGGAGTGGGCCAGTAGTCCCGGAA
AAGGCAGTCCGTTTTTCATTCACAATCATGAAAATTACTATAGCCCATGACTCTCAGAACATGAAGGTGTTTG
AAGAAGCCAAACCTAACTCTGAACTGTGTTGCAAACCATTATGCCTTATGCTGGCAGATGAGTCTGATCATGA
GACCCTGACTGCCATCCTGAGTCCTCTCATTGCCGAGAGGGAGGCCATGAAGAGCAGTGAATTAATGCTTGAG
ATGGGAGGCATTCTCCGGACCTTCAAGTTCATCTTCAGGGGCACTGGATATGATGAAAAACTTGTGCGGGAAG
TGGAAGGCCTTGAGGCTTCTGGCTCAGTCTACATCTGTACTCTTTGTGATGCCACCCGTCTGGAAGCCTCTCA
AAATCTTGTCTTCCACTCTATAACCAGAAGCCATGCCGAGAACCTGGAACGTTATGAGGTCTGGCGTTCCAAC
CCTTACCATGAGTCTGTGGAAGAACTGCGGGATCGGGTGAAAGGGGTCTCAGCCAAACCTTTCATTGAGACAG
TCCCTTCCATAGACGCACTCCACTGTGACATTGGCAATGCGGCTGAGTTCTACAAGATCTTCCAGCTAGAGAT
AGGGGAGGTGTATAAGAATCCCAATGCTTCCAAAGAGGAAAGGAAAAGGTGGCAGGCCACATTGGACAAGCAT
CTCCGGAAGAAGATGAACCTCAAACCAATCATGAGGATGAATGGCAACTTTGCCAGGAAGCTCATGACCAAAG
AGACTGTGGATGTGGTTTGTGAGTTAATTCCTTCTGAGGAGAGGCACGAGGCTCTGAGGGAGCTGATGGATCT
TTACCTTTAGATGAAACCAGTATGGCGGTCATCATGCCCTGCTAAAGA--GTGCCC-
AGAATCCCTCTGCCAGTACAGTTTCAATTCACAGCGTTTTGCTGAGCTCCTTTCTACCAAGTTCAAGTATAG?
??????????????????????????????????TAGATGTTGCTTATGGCACCCGACAGTTGTCATTATGTT
TGGAAACACTACCGGCCCATGGAGATGAGGATAAAGCGGACATTTCTCTGCTGCATCAGGGTGAGAATCTTTT
CGAACTGCACATCCACCAGGCCTTCCTGACATCTGCCGCCCTGGCTCAGGCTGGAGACACCCAACCTACCACA
TTCTGCACCTATTCCTTCTATGACTTTGAAACCCATTGTACCCCACTGTCTGTAGGGCCACAGCCCCTCTATG
ACTTCACCTCCCAGTATGTGGTGGAGACAGACTCCCTTTTCTTACACTACCTTCAAGGGGCTTCAGCCCGGTT
TGATCTCCACCAGGCTGTGGCCAGTGAGCACAACACTCTTGCTGCAGGATGGATTTGCTTTGACAGGGTGCTA
GAGACTGTGGAGAAGGTCCATGGCTTGGCCACACTAATTGGTAAGTGCTGTCAGCTTCCTGCAGCTCCTA-
GCACCAATGCAGAATTTCCCAAACTCTATGGCTGCTTTTCTGGTCGTTTCTTATTTTCTTCTTTTCTCTTTGT
TCTTGATCCTTGCCACACCATCTGTATCCTCCATGCTTTCA-
TACTTGCTGCTGCCCAGGAGCTGGTGGAGAAGAGTTTGGGGTTCTAGAGTACTGGATGAGGCTGCGTTTCCCC
ATAAAACCCAGCCTACAGGCATACAATAAACGAAAGAAA??????????????????????????????????
??????????????????????????GTTCATGGAGGGAAAACACCAAACAATGAGCTTTCAGATAAGATTTA
TGTCTTGTCTGTTGTTTCCAAGAACAATAAAAAGGTTACTTTTCGCTGCACGGAGAAAGACTTGGTAGGAGAT
GTTCCTGAAGCCAGATATGGTCATTCCATTAATGTGGTATATAGCCGAGGGAAAAGTATGGGTGTTCTTTTTG
GAGGACGCTCATACATGCCTTCTACCCACAGAACCACAGAAAAATGGAATAGTGTAGCTGACTGCCTGCCCCA
TGTTTTCCTGGTGGATTTTGAATTCGGGTGTGCTACATCATACATTCTTCCAGAACTTCAGGATGGGCTATCT
TTTCATGTCTCTATTGCCAAAAATGATACCATCTATATTTTAGGAGGACATTCACTTGCCAATAATATCCGCC
CTGCCAACCTGTACAGAATAAGGGTTGATCTTCCCCTGGGTAGCCCAGCTGT-
GAATTGCACAGTCTTGCCAGGAGGAATCTCTGTCTCCAGTGCA??????????????????????????????

???
??TGTGTCTCAGCATCTTCCTGCTC
CCCACCACACTGAGCCCACGATGACCCAGTGCTCCACAAATATACGTAGAATGGAAGTAAAACCTCAGGGATA
AGAAAAGATAATTAAAGGGCCGGGTGCGGTGGTTCATGCCTGTAATCCC-------
TAGGAGGCTGAGGCGAGTGGATCACAAGGTCAGGAGTTCAAGATCAGCCTGGCCAAGATGGTGAAAACCCGTC
TCTACTAAAAATA---CAACAATT-----
AGCTGGG--
---------------------CATGGTGGTGGGCACCTGTAATCCCAGCTACTTGGGAGCCTGAAGCAG-
AGAACTGCTTGAACCTGGGAGGCAGAGGTTGCAGTGAGCCGAGATCACGCCACTGCACTCCAGCCCGGACAAC
A-GAGTGAGACTCCATCTCAA--

---AAAA-
TAATAATAAAAAGAAAAGATAGTTAAAGAAGATTCACTACAGTAACAAACCAGCCAAGAAATTGCACTGAAAG
TAAAGGTTT-
GGGTGACAAAATTTCCAACTGTGTTCCAAGAAACATAAGTTCCAGCAGGAGTGCACTTAATCTGATTTT-
CTTCAAAGTGCCTCTCTAATTTGTAGGGAAGGACATTCATTTTTGTTAGCTTCATGTGAGCTAGAGAGAATCC
ATGATTGAAGTGCTAGAACCCCTGGTAAACATGCCAGCAAGCAAGGGCACCTTCCCCAGAGGCTCAAACTAGA
ATATAAAACTGTTGGCATTGGTAAGGAGCCTTGAACTCTAAACCCATAGGCTCAAGTCAGGAA-
CTCAGGAAGCCTATCAACACCGGGATACAATTTTGCCACCTTAACACTTTATTCCCTTTACTTGTGCC?????
???
???
???
???
???
???????????????????????TTAGGAACTCAAGGCCCTCAAAGATCTTTACTGCCATAAAGCGGAGTAAC
T-GTCTTTGACTTGGACCTTGTTTACTGTGAACTCTAATC-AAGGCAGGACGTGCAGCAGTTCTACTTA-
CTGCACTGTGGACGTGTGGAAAGGACACTTGTGA---------
ATCCAGTTGGGATTAGTGTTTGAAATTGAATGGCGAATGTTGTTCCAGGATATGGAATGCAGCCGTGATCACA
CTTGCCTACC-GAGCTTACTTTGAACTATTTGTCAATAGCATGCAAAAAA-
TGCTGTGTCTGCCCTTTGCTTCTGCTTTTTCCAGGGAAGCTGCCAAAGAGTGTCGACGTCGAAAGAAAGAATA
CGTAAAATGTCTGGAGAGCCGAGTTGCAGTGCTGGAAGTCCAGAACAAGAAGCTTATAGAGCAGCTTGAAACC
TTGAAAGACATTT??
???
???
???
???
???
???
???
???
??TGTTTATATCTAAATGTGTGGATTCCA
GCACCTAAACCAAAAAATGCCACTGTAATGATATGG-
ATTTATGGTGGTGGTTTTCAAACTGGAACATCATCTTTACATGTTTATGATGGCAAGTTTCTGGCTCGGGTCG
AAAGAGTTATTGTAGTGTCAATGAACTACAGGGTGGGTGCCCTAGGATTCTTAGCTTTGCCAGGAAATCCTGA
GGCTCCAGGAAACATGGGTTTATTTGATCAACGGTTGGCTCTTCAGTGGGTTCAAAAAAATATAGCAGCCTTT
GGTGGAAATCCTAAAAGTGTAACTCTTTTTGGAGAAAGTGCAGGAGCAGCTTCAGTTAGTCTGCATTTGCTTT
CTCCTGGAAGCCATTCTTTGTTCACCAGAGCCATTCTGCAAAGTGGATCCTCCAATGCTCCTTGGGCAGTAAC
ATCTCTTTACGAAGCTAGGAACAGAACGTTGGCCTTAGCTAAATTTACTGGTTGCTCTAGAGAAAATGAGACT
GAAATAGTCAAATGCCTTCAAAACAAAGATCCCCAAGAAATTCTTCTGAATGAAGCCTTTGTTGTCCCCAACG
GAACTCTCTTGTCAGTAAACTTTGGTCCAATAGTGGATGGTGATTTTCTCACTGACATGCCAGACATATTACT
TGAACTTGGACAATTTAAAAAAACCCAGATTTTGGTAGGTGTTAATAAAGATGAAGGGACAGCTTTTTTAGTC
TATGGTGCTCCTGGCTTCAGCAAAGATAACAATAGTTTCATAACTAGAAAAGAATTTCAGGAAGGTTTAAAAA
TATTTTTTCCAGGAGTGAGTGAGCTTGGAAAGGAATCCATCCTTTTTCATTACACAGACTGGGTAGATGACCA
GAGACCTGAAAACTACCGTGAGGCCTTGGATGATGTTGTGGGGGATTACAATATCATATGCCCGGCCTTGGAG

TTTACCAAGAAGTTCTCAGAATGGGGAAATAATGCCTTTTT??????????????????TTGTGCTAATTGTG
AGAAAATATGCTACTTTGATCAAACTAGAATGAAGGAAATAAAAAGACACTGTGGCTCAACTTGTAATAAAGA
GTAGCTCATCCTCACAGCTTTTTGATTGTTGCTATAGTTATCT---
ATTCAACATGGGATGCACAATACAGTTAACACTTCATTACCAGTAGCCTGTTGCGTGCTTATTGTTAAAAGGA
ATTTAACATACAAGTTTTTGTATGGATTGTTCGCCTAATTCCGATTCCTCGTGCTGACTGGCATGAGACAGGG
AGCTGGATTGTATTAAACTGGTTTTATTGTGATGTGACTATGATGGATGTGACAAGGGCCTCTATTATTCGCT
GGGGAGTGTGGCCGTTAATGAAGCCATTAGCTGAGCTAAGGCAAGTGAAGAAAATAGGGAAATTACCATTATT
CTGAAGCCATTAAGTTGTAATCCACCACTTTCTCTCCTTATTCAAAAAGGCATCTTCTTTTTAAGGCCTCCCT
??GAC
AAGGTGGCTTGGCCTACCCAGCTATGCGGACCCATGGGACTCTGGAGAGCGTGAATGGGCCCAAGGCAGGTTC
AAGAGGCCTGACGTCGTCGTTAGCTGACACTTTTGAACATGTGATAGAAGAGCTGTTGGATGAGGACCAGAAA
GTTCGGCCCCATGAAGAAAACAATAAGGACGCAGACTTGTACACGTCCAGGGTGATGCTCAGTAGTCAAGTGC
CTTTGGAGCCTCCTCTTCTCTTTCTGCTGGAGGAATACAAAAATTACCTGGATGCTGCAAACATGTCCATGAG
GGTCCGGCGCCACTCTGACCCTGCCCGCCGAGGGGAGCTAAGTGTGTGTGACAGTATTAGTGAGTGGGTAACG
GCGGCAGACAAAAAGACTGCAGTGGACATGTCGGGCGGACGGTCACAGTCCTTGAAAAGGTCCCTGTATCGA
AAGGCCAACTGAAGCAATACTTCTACGAGACCAAGTGCAATCCCATGGGTTACACAAAAGA????????????
?????????????????????????????AACGGCTATGAAAATCCAACCTACAAGTTCTTTGAGCAGATGCA
GAACTAGACCCCC-
GCCACAGCAGCCTCTGAAGTTGGACAGCAAAACCATTGCTTCACTACCCATCGGTGTCCATTTATAGAATAAT
GTGGAAAGAAACAAACCC-
TCTGTCTTATGATTTACTCATTATCGCCTTTTGACAGCTGTGCTGTAACACAAGTAGATGCCTGAACTTGAAT
TAATCCACAGATCAGTAATGTTCTCTCTCTCTCTCTCTCTCTTT------------------
ACATTTTGGTCTCTACACTACATTATTAATGGGTTTTGTGTACTGTAAAGAATTTAGCTGTTTC-
AAACCTAGTGCATGA-
ATAGATTCTCTCCTGATTATTTATCACATAGCCCCTTAGCCAGTTGTATATTATTCTTGTGGTTTGTGACCCA
ACTAAGTCCTACTTTAAATATGCTTTAA-GAATCGATGGGGGA---
TGCTTCACGTGAACGTGGGAGTTTAGCTGCTTCTCTTGCCTAAGTATTCCTTTCCT-
GATCACTATGCATTTTAAAGTTAAA-CATTTTTAAGTATTTCAGATGCTTTAGAGAGATTTTTTTCC---
ATGATTGCATTTTACTGTACAGATTGCTGCTTCTGCTGTATTTGTGATATAGGAATTAAGAGGATACACGTTT
GGTTCTTCGTGCCTGTTTTATGTGCA??????????TCCGGAGAGCACTAAGCCACTTCCAGCCCTGCTGTGA
ACCAGGACCGTAGGAGCCATCTTTTTCATGTGGTCAGCTTTGGAAGCATCTACATCCACCACTTTAGAAGACA
AGTCTAGTGGCTTATCTGTGACGTCTTTGGTAACGGTCTGCTTCTCCAACAGAGGAGGTGAGCTGCCGTCTTT
TCTGTCTTGAACCGCTGTCTTCCGGGCATGCCCGGGCACTGGCTGGGTACCTTCGCCCCCTTCCGGAGCCTTG
GGATACTTGCCGTTGGAGAGCCTGGCCGCGGGGAACTCGCTGCTGACTGTCATGTATGGCTTGGACAGGGCAA
CTGAAGGAGAGGTGGAGATCCTGGCATAGTGCTTGTGGAACTCCGAGTAGGTGTCTGCAGTAGGCTGGGTGGG
AAGGTGGACGCGGGGTGAGGGCCGAGGCGAGGGGGGCAACAGGAGAGCCGTGTCCCCCGGCAGGCCACTGGTG
ACTGCCTTGGCAGAGGGAACCCTAGGCTGTTTACTGTTCTGGATGTGAGGATAGGCATGGGAATCAACGGGAT
TCCCAGGGCTGACCCCCATCTTCCACGGGAGGCTTTTGTCTGCGCAATGGACAAGAGGCGGGATGGCTGGGGA
GGCCGAAGGCGTCGAGAGCCTCATGGGTGATGCCAAGGACGACGGGATGTGGGGACTGACGTAGTGAGGTGGT
GGCAGGTAGAGAAAGCGCTCCCCGTTGGTGCAGACTGGAGAATACAGCGGCTGGGCCAAGCTGTAGGACTGCT
GAGGTAGCAAGGTGCCAGGACCTTTGTAATTCAAGTGCTTTTTACAGTAGTAGCAAATGTTACATAAGCCCAA
ACATAGAGCA---
TATTTGGGGTTGGTGGGCAGACCCAAATCATGATGCAAGCCTGCAGTAGCAAGTGTGCAATGAAGCACAACCA
AATTCTGTGAATGGAAGGTAGCGAGACGCCTCTCCGAAGCAGCCCTTCTGCGCAGGAGCTGAAAGCTCCACCG
GGTAAGTTTTCTGAGGCAGTTTTCAGAGGCTGGGCTTGGCTCAGGGCCCAGCTCTGGATGAGCCTGTGGACGG
TGGGGT----
AGGTCTTTGCTAACCTTACCTAGAATCAAGCAGAGTTTGGAAGAGTTTGTGCAGGCCTCTTTCCTCCTGGCGT
TTGTCTTGCCCTGCCAGCAGGCCAGGAGGG-
CTGCGCTGCTCAAGTAGTGGAGGGAGGGAGCCTGCAGAGCTAGGGGATCAGAGTAGGTAATCGCAGCCACAGG
GATTAGCTGCTGCAAGCTGTCCACAGGAGCCATTCTGAAATGTCATTTGAGTGATCTGGCCGTCTACTGGAGG
AGGCCCTCGGGGCAGCTGTCTCCAGGAGCAGCTTCTGAGGGAGTCTTCCGGGAGGAGCAGTGTCTCTGCCATC
GTCCTCGTCCACGTCCTGGTCACATTTCCATTCCTCATCTTCATTTAAAGTCGGCAGGTATCCAGATATGCCC
TTCCCTGTCCCTGACCCAGAGCTCTGGTCACTACAGACCTTCGGGCTCTCCGAGCCTGTCCTCGTATATTCCA
AATAAATATCAGACTTAAGGAAGGAGGGGTAGGTGTTTTCCTCCATAGTGGCCTGGATTTCTGTCTGGGCCTG

GTCAAACATGGCAGGATCGATCAGCTGCTTCATGATGCAGCCCTTTATGAAGCTCTTGGTGGCCGGCTTGGTC
TGCCGGGACACGATGCCATTGTTATCAAGGATGTACTTTCGGTAGATGGCTCTGGCCAGCTTCAGCCTCTTCT
CCTCGTTGGAGTCACAGGGCTCCAGCTTCCTGAAGCCAGTGCAGGCAAACCAGAAGTCCAGCAAGTCAGCACA
ACCCTCCTGCTTCAGGAAAGTCCTAAACAGGCTTATCCCATCTTGATCATCCAGCAGGGAGTGCAGTGACTCA
GCCCACTTCAAGTACGGTGGGGTAGGGGAGGCACTGCCCTCAGGCTCATACCCCAGGTCCAGATCCGAGCGCC
TCGGAGTGGCGGTCGAAGTCTCACCTTTAATGCCAATACCTTTCCCGGAGCAGAAACTGTAGCTGGCGGGCCT
CGGGTCTGTGGACACCAGTTCTCCCTCCTCACCAGGCACTGGGGGTCGGGGAGCATCTTCGGTGAAACTTGCT
CCGAGGTCCAAGGGGAAACCCTGCTCCTGGACATTCATTTTGGGACTCTGTGCGTCAATGCACAATGAGCGCT
GCACCCTAATACATCAGTACTTA-
CAGCTCCAAAGTCAATCAATCTGTCCTGTTGAAACCAT?????CTAAAGTGTTAGCTAATTTTAAGTGAACAA
-A--ATTACATGGATCGACTTTTGGCAGCATAAAACAGGTATAATTTACAGTGGAGCAAGATAAGT-
ACTTAGATTATTTATAGGACAGATTACTTTTCTGTGTTGTTTTG----CTTCTTTG-
TATATTAAAGTTTATTTATAGGTGTACTTAAGCTTT--ACTATTAG----CATAAATTTGGCTTGGAGTCA--
--
CCAAAGCAGAAATATTGGACTTCATTTTTATGAGTTATTTCCTACACATCTCTAAATTGATGTAGCTTTGTCA
CTGCTTGATTGATTTTTTTTT--
AAACTGGAGTGGGTGAAATTAGTATATACTGAATCAAACCAGTTTCTCTTAATTAGTA---------------
----CATTACTAACACTGAAAAGAAGTAAAAGGAATGTTAATAACTTGGGAGTATTTGCATAGTTA-
TGTTCAGAACACAAGCTTTAAATTAGT-GAGGAAACCA---------
GAAGTTTGATTTAAGCACTCATACTCCTTTATTTTCCTTTGTTAGCAATAATTATTGGCCCTGATGGCCATCC
TTTGACTGTTTATCCTTGTATGATTTGTGGGAAGAAGTTTAAGTCGAGAGGTTTTTTGAAAAGACACATGAAA
AATCATCCCGAACATCTTGTCAAGAAGAAGTACCACTGTACTGACTGTGATTACACTACCAACAAGAAGATAA
GTTTACACAACCACCTGGAAAGCCACAAACTGACCAGTAAGGCAGAGAAGGCCATTGAATGTGATGAGTGTGG
GAAGCATTTTTCTCATGCAGGGGCTTTGTTTACTCACAAAATGGTGCACAAGGAAAAAGGAGCCAACAAAATG
CACAAGTGTAAATTCTGTGAATACGAGACAGCTGAA?????????????????????????????????????
??????????????????TACCCGCTATAGCTTCTGCAAACAGTAAAGATACCACTAAAACCCCTTCTGCAGT
TCAAACTAGCACCAGCCACAACCCTGGACTTCCTCCTAATTTTAACGAATGGTACGTCTGAGGACAAACACAA
ACCCTGTTAACTATAGAATGGACCAAATGCATTTTTAAAAGAAAACTGAGACCAATCAGATGGAAATGGAGTT
TTAAGGCAAGAGGCCATATATAGGGCTACATCTTGTTAATTGCAGTTGTCCAGGAAGGTTTTGGGCAAGATCC
AAAAGTAGCCATGCCCTTTTCTCAGGATTAGAAAATATGTTTTGGCGTTTGAAGGATTTTTTTTACAAAATCT
TTACACTGCTTTTTCTTCCCCTTCCTCTTGCTCTCTGCACACCCCATTCCTAAACTCCTGCAATTCATTTTAA
CACTTGTCCTGTTTCTTGAGAGGAAGTTATAGAAGGCTTGTTGGTGGTGGTGATGTTAAACTGATGGAAATTC
TTTTTCGCCTTAGTGGTGATTGTTTAAACTCTCACAGTCTTAAACCGTGCCAAAGTCCTGTTATA
Presbytis
?TACCCCCATGTTTGGCTGGAACATGAAACTGACCTCAGAGTACCACAGAAATGTCACCTTCCTTTCATGCCA
ATTCCTTT-
CTGTCATAAGAATGGACTACATGGTATACTTYAGCTTCTTCACCTGGATTTTCATCCCCCTGGTTGTCATGTG
CGC--CATCTATCTTGACATCTTTTACAT-
CATCCGGAACAAACTCAGTCAGAACTTCTCTAACTCCAAAGAGACAGGTGCATT---
TTATGGACGGGAGTTCAAGACAGCTAAGTCCTTGTTTCTGGTTCTTCTCTTGTTTGCTCTGTCATGGCTGCCT
TTATCCATCATCAACTGCATCATCTACTTTAA--------------
TGGTGAGGTACCACAGCTTGTGCTGTACATGGGCATCCTGCTGTCCCATGCCAACTCCATGATGAACCCTATC
?????????????????????ATGCTCATTAACCGCTGGTTATTCTCTACAAATCATAAGGATATTGGAACTT
TATACCTATTATTTGGAGCATGAGCTGGAACTATAGGCATGGCTATAAGCCTCCTTATTCGAGCTGAGCTAGG
CCAACCCGGTAACCTACTAGGCAATGACCATATTTATAATGTTATCGTTACAGCCCATGCATTCGTTATAATC
TTCTTTATAGT---TATACCTATTATAATTGGAGGCTTCGG---
AAACTGACTAGTGCCCCTAATAATTGGTGCTCCTGACATAGCATTCCCCCGTCTAAATAACATAAGTTTCTGG
CTTCTTCCACCA--TCATTCCTACTTCTTCTCG-
CTTCGGCCATAGTAGAAGCCGGTGCCGGAACAGGTTGAACAGTCTACCCCCCTTTAGCAGGGAATTTATCTCA
CCCAGGAGCCTCCGTAGACTTAACTATCTTTTCACTTCATCTAGCAGGTATTTCTTCTATCTTGGGTGCTATT
AATTTTATCACAACCATTATCAATATAAAACCCCCTGCAATATCTCAATACCAAACCCCCTTATTTGTATGAT
CGGTCCTAATCACAGCAGTCTTACTGCTCCTATCTCTACCCGTACTAGCCGCAGGCATTACAATGCTATTAAC
AGACC--
GCAATCTCAACACTACCTTCTTTGATCCTGCCGGAGGGGGAGACCCTATCCTATATCAACACTTATTCTGATT

CTTTGGACATCCAGAAGTGTATATTCTTATTTTACCTGGCTTTGGAATAATCTCCCACATCGTAACATATTAT
TCTGGAAAAAAAGAACCATTCGGGTATATAGGTATAGTCTGAGCTATAATATCAATTGGGTTTCTAGGCTTTA
TTGTGTGAGCTCACCATATATT-
TACAGTTGGCATAGACGTAGATACACGGGCCTATTTTACCTCTGCCACTATAATTATTGCAATTCCAACTGGC
GTTAAAGTCTTTAGCTGATTAGCCACATTACACGGAGGAAATATTAAATGATCCCCCGCAATACTCTGAGCTC
TAGGCTTTATTTTCCTTTTTACTGTAGGGGGTTTAACCGGTATTGTATTAGCAAACTCCTCACTAGATAT-
CGTATTACACGATACATATTATGTGGTAGCCCATTTTCACTATGTCTTATCAATAGGGGCAG-
TCTTTGCTATTATAGGGGGTTTCATTCACTGATTTCCCCTGTTCTCAGGCTATACTCTAGACCAAGTCTACGC
TAAAGCCCATTT-
TACTATTATATTTGTAGGCGTAAATTTAACCTTCTTCCCACAACATTTTCTAGGTCTATCCGGAATACCTCGA
CGCTATTCTGACTACCCTGATGCCTATACTACATGAAACATTGTATCATCTACAGGTTCTTTTATTTCCATGG
TAGCAATACTATTAATAATTTATATAATCTGAGAAGCCTTCGCTTCAAAACGTAAAGTACTATTAATTGAACA
ACCTGCTTATAATTTAGAGTGATTACATGGCTCCCCTCCACCATACCATACATTTGAAGAACCAACATTTATC
AAAGTAAACAAAAAAGGAAGG???????????????ATGGCCCACCCAGTTCAACTAGGCCTACAAGATGCCA
CATCCCCTATTATAGAAGAATTAATTGCCTTTCACGATCACACTTTCATAATTGTGTCTCTAATTAGCTTCCT
AGTTCTATATGTTTTATCATCAGTGTTAACAACAAAATTAATTAGTACTAACATTACAGACGCTCAAGAGATA
GAGACTATTTGAACCATCTTACCCGCAATCATCCTAGTCTTAATTGCTTTACCCTCTTTACGTATCCTTTACC
TAACAGATGAGATCAACAACCCCTCATTCACTATTAAATCAATTGGACATCAGTGATACTGAACTTATGAATA
TACAGACTATGGGGGTTTAATCTTTAATTCTTATATACTCCCTTCACTATTCTTAAATCCAGGAGACTTACGA
CTCCTAGAAGTTGATAACCGAGTAGTACTGCCAATTGAAGCTCCTGTACGTATAATAATTACATCCCAAGACG
TCTTACACTCATGAACCATTCCCACACTAGGCCTAAAAACTGATGCAATTCCCGGACGCTTAAACCAAACAAC
ATTTACTGCTATACGACCAGGTGTATACTACGGACAATGCTCAGAAATCTGTGGTGCTAATCATAGTTTCATA
CCAATTGTTGCAGAATTAATCCCACTAAAAATTTTTGAAATAGGG--
CCTGTATTTACCTTATAG????????ATGACCCCCACACGTAAGTCTAACCCAATTATAAAACTAATTAACTA
CTCCCTTATTGATTTACCTACCCCATCAAACATCTCTATGTGATGAAATTTTGGCTCCCTACTAGCAACCTGC
TTAATTTTACAAATTATCACTGGCTTATTCCTAGCAATACACTATTCACCAGACACTTCTTCTGCCTTCTCCT
CAATCGCACATATCACTCGAGACGTAAACCATGGCTGAATTATCCGATATCTCCATGCTAATGGCGCTTCCAT
ATTCTTCATCTGCTTATTTCTACATGTAGGTCGAGGCCTGTATTACGGCTCATTTCTTCTCCTAGAAACCTGA
AATATTGGCATCATACTCTTATTCATAGTTATAGCAACAGCCTTCATAGGATATGTACTTCCATGAGGGCAAA
TATCATTTTGAGGTGCTACAGTAATTACAAATCTATTATCCGCAATCCCATATATCGGAATAAACCTTGTTCA
ATGAGTTTGAGGTGGATACTCCATTGATAACCCAACCCTTACACGATTTTTTACTCTCCACTTTACATTACCT
TTCATTATCACAGCCCTCACAATACTACACCTACTATTTCTACACGAAACAGGCTCAAATAATCCCTGCGGAA
TTCCCTCCGACTCCGATAAAATTCCCTTCCACCCCTACTATACTATCAAGGACATATTAGGTATAATCCTTCT
TTTCCTTATTCTAATAGTACTGGTACTATTCTCACCCGACCTTTTAAGCGACCCAGATAACTACACCCCAGCC
AACCCACTAAACACTCCATCACACATCAAGCCAGAATGATATTTCCTGTTTGCATATGCAATCCTACGATCTA
TCCCTAATAAGTTAGGGGGTGTATTAGCACTTCTCCTGTCTATTCTTATTCTAGCAGTTATTCCTCTATTACA
CAAATCTAAGCAACAAAGCATATCTTTTCGCCCACTTAGCCAATTCCTACTATGACTCCTAGTTACAATCCTA
CTAACTCTTACATGAGTTGGAAGTCAACCGGTAAACCAACCTTTCATTATAATCGGCCAAGTAGCATCCACAT
TATATTTCACCACAACCCTAATCTTAATACCACTAGCCTCCCTAATCGAAAATAATCTACTTAAAT??????T
TAGGTTCAATTTATTTTAGAGAATGAGAATTTATGGGTATAACTGTTAGCTGGAAAGCCGTTCCTCACCTCCA
AGCCTCATTTCAAACTATGTTTCC-
TGCTGAAATTTCTCAGTGGGATGACATGTTCAATTATTATTCAGTCTGTC----
TCTTATATGTTATTTGGCTTTACATAT----
GCCCTTGGTGGTTCTTGGAAGATAATCTAATATTGTGTTGTTTTGGTGTCTTTGTATTGGAAATGGAAGCATT
TTGTATGAATCACCTCCTTAACTGTGAGGAGTTAAATTGTCTTAGGTCTGCTTGTCACATGACCGGGAATAAA
--
GACATCACAGTCTCCAACCCCACTTTTTTGGTGGAAAGAAAAGCTAAAAGTAGCAACATTTAATGGAGCAAAA
TATCAGTAATCGAAGAAACTCCCCTGTGAAACTCTAATATTATTTT-
AAAACCAACATTGTTCTAAGGTAGCTGAAATGTGTGATTTTTCTATTTTGCCTTTTCACTGACAATATACCCA
TAAATCT-
TGGTGGTGATTTTCTTTCCAAAGTTAATTTCTGGGCCATTGTTGACAGCACTATCTATTGCTCTAAATTGTTG
TCTTAACTGCATTCTTTACAAATGTAATGTTGAAGGCACATAATATTGAAGATGACTCCAGGATGAACCCTGA
GTTTGCAGACCGAA-
TAAAACAGCTAGATAAAGAGGCGTCTTACTACCGCGACGAGTGTGGCAAGGCCCAAGCAGAAGTGGACCGGTT

GCTGGAGA?????????GGAAGAGCTGCATCAGGCAGGTACTTTGATCTTTCTTGGAGCAGGTTCCTTCATAA
CGCTTCTCCAGAGGACGGAAGTCCCTCCAGTCTGGCTCGCTGCTCGCAGTGGTGGGAAAAGCTGAGGTAATTC
CCCGGTGGGAAATCTGAGAGGTCCCTGATAGCCCTG--------
CTGCCAGGCCCATCACTGATGGGCCTAAGGGCCTCATCTGATACTCTAAGACGGGCTTCTCCTGCCGGGCCTG
GGCCTCTCGGACTTGAGTCTCTCTAGAACGAGCCTCTCGGGCCTGGGGTTCTCGGGTTCTGGCCTCCCTGCCA
CGAGCTTCCCAAGTGTGGGCCTCCCTG--
GCATAGGCTTCCCTGCCATGAGACTCTCGAGTATAGGCCTCCCTGGTGTGGGCCTCCCTGGCATGAGCTTCTC
GGGCACGTGCCTCCTGGGCCTCAAGCTTCTCCCGCCGAAGCTCCCAATACAACAACTGTTTCTGGATGGTCA-
CTAGCCGTTCTTCCTCTGTCTCCATTGCCCCAGGTGGCCGGGAGGAAGAAAAGGGCTCAAAGTTTAAGAAGGG
GTCAAACATCTCAGAGCTTCGACCATGGAGGTCATAAAGGCAGTCATCTCCAGGTGGAGAGTTCTCAAGGCTG
TCATCTGGCTCATAGAACTCATATAGGGCATCTCCACTGTAACTGTCTCGGGGTAGACAATCCCTGTGGACAA
GCCCCAGGGCCTCACCTGAATCATCCTCAA???
???
???
???
???
???
???
???
?????????????????????????????????ATGTTCACAATCAATCTCCTACTTATCATTTTGCCCACTA
TGGCTGCCATAGCATTTCTCACACTTACCGAACGAAAATTACTAGGTTATATACAATTACGCAAAGGGCCTAA
TATCGTAGGCCCCTATGGACTACTACAACCCTTTGCCGATGCAATAAAACTCTTCACCAAAGAACCCCTAAAA
CCCCTAACCTCCACTATCACTTTGTACATTATTGCACCAGCCTTGGCCTTTACCATTGCCCTCCTCTTATGAA
TACCTCTCCCTATACCCAATCCTCTAATCAACCTTAACCTAGGACTTTTATTTATTTTAGCCATATCCAGTTT
AACTGTCCACTCTATTCTATGATCAGGATGAGCATCAAATTCAAATTATGCATTAATTGGAGCACTACGAGCA
GTGGCCCAAACAATTTCATATGAGGTAACTATAGCCATTATTCTATTATCAATCCTATTAATAAGCGGCTCAT
TCAATCTCCATGCACTTATCACAACACAAGAAAACCTTTGACTTCTTCTACCATCATGACCTCTAGCCATAAT
ATGATTTACTTCCACATTAGCAGAAACCAATCGAGCTCCCTTTGACCTAACAGAAGGAGAATCAGAACTAGTC
TCAGGTTTTAATATTGAATATGCAGCAGGCCCATTCGCCCTTTTCTTTATGGCTGAGTATATAAATATTATCA
TGATAAATGCCCTAACAGCTACAATTTTTCTAGGTACACTATATCCAATCCACTCACCAGAGCTGTTCACAAC
ATGTTTTGTCATCAAAACACTCCTCCTAACCTCCCTATTTCTATGAATCCGAGCAACCTACCCACGATTCCGC
TATGATCAACTTATACACCTGTTATGAAAAAACTTTCTCCCCCTTACATTAGCATTTCTCATATGATACACAA
CAATACCCATTACAATCTCCGGCATTCCCCCTCAAAACTAGATTAATCCACTAGCTCAACTTATCATTTACTC
TACTATAATTATAGGTACCCTTATTACATCACTAAGCTCCCACTGATTTCTAGCCTGAGCAGGCCTAGAAATA
AATATATTAGCCTTCACTTCAATTCTAATTAAAAAAACAAATTCTCGCTCCACAGAGGCTGCTACTAAATATT
TCCTTGTACAATCCACCGCATCCATAATTCTCATGATAGCAATTATATACAATAATATATACCCAGGACAATG
AGCCATGGTCAACAACATTAACCAACTCTCATCCTTAATTATAATAATAGCCTTTGCTATAAAACTAGGAATA
GCCCCATTCCACTTCTGAGTTCCAGAAGTTACCCAAGGAACACCCCTAATACCCGGCCTGCTTCTACTTACAT
GACAAAAATTAGCCCCTATATCAATTATATACCAAATTTATCCCTCAATTAATACAAACATCCTCCTAACCCT
ATCAATCCTATCTATTATGGTGGGTAGTTGAGGAGGTCTCAACCAAACACAATTACGTAAAATCCTAGCATAT
TCTTCAATTACACATATAGGCTGAATAATAACAACACTAACATATAACCCAAACATCACAATCTTCTATTTAT
TTACATACATTATTATTACAACCACTACATTCCTAGCCCTAAACCTAAACTCAAATACCACAGCCCTAATATT
ATCACGAGCCTGAAATAAACTCACCTGGTTAATACCACTAATAGCATCCACTCTTCTATCTATAGGGGGCCTA
CCCCCATTAACCGGCTTTTTACCCAAATGAATCACCATTCTAGAACTTACAAAAAATAATAACTTTATTATGC
CTACTATAATAATCACCATAGCCCTACTCAACTTATACTTTTACTTACGCCTAGTCTATATTACCTCCATAAC
ACTACTCCCTACATCCAATAACACAAAAATAAAATGACAGTTCGAAAGTACAAAACCTATACCTCTAATAGCC
CCTCTCATCATTCTCACTACTCTCCTCCTACCAGTATCTACAACAGCCCTAAATATTCATTAGGTTGGCAGCA
TAAATAATAAAGCATGTTACTTAAGCAATTTTACATTTACTGTATTAGTAAGTAAATGCAATGGCCACTCTTA
TTCTCAAACAATAAAAACTACCCAGCCTTTTCATGAAAGAT-
GATTGCAATTTCCATTGGCCTGCATATGCAATAAAAAG---
CCATAACTCATCAAAATTTTAGCTATTTTCTCTTAAGATA--
GAAGAGCATTTTGCTTGAACATTGAACGTGATATTAAATATT-
CATGAAACTGAATAGCGTTTAAGTACTGAG---
TAAAACTCTCAAATTCTTTTCAACTAGATTGAAATTCTGTTTGAACCAGGTAAAAACTGCCGCTAACCTGAAG

AGGTGTTCAAAAAC-
ATCGTAAAGAGGTTGTACAATATGTTCAAAGTGCTATAGTTATTTGGGATTGATTTTTCTGAAAAGCCTGTTC
CTCCTTTGAAATTGATTCATGATAACAGCCTCCATTTGGGTCAAACAGTATTTAACCGCTTGTAAA-
TTTTAATT-
AAATGATTTGTCACTTTGAACACATAGTTTGTCCCCAAGACCTTGATGATTACCTGAATGGCCCCTTCACAGT
GGTGGTGAAGGAGTCTTGTGATGGAATGGGAGATGTGAGTGAGAAGCACGGGAGTGGGCCAGTAGTTCCAGAA
AAGGCAGTCCGTTTTTCATTCACAATCATGAAAATTACTATCGCCCACAGCTCTCAGAATGTGAAAGTATTTG
AGGAAGCCAAACCTAACTCTGAACTGTGTTGCAAGCCATTGTGCCTTATGCTGGCAGATGAGTCTGACCACGA
GACCCTGACTGCCATCCTGAGCCCTCTCATTGCCGAGAGGGAGGCCATGAAGAGCAGTGAATTAATGCTTGAG
CTGGGAGGCATTCTACGGACTTTCAAGTTCATCTTCAGGGGTACCGGATATGATGAAAAACTTGTGCGGGAAG
TGGAAGGCCTCGAGGCTTCTGGCTCAGTCTACATTTGTACTCTTTGTGACGCCACCCGTCTGGAAGCCTCTCA
AAATCTTGTCTTCCACTCTATAACCAGAAGCCATGCTGAGAACCTGGAACGTTATGAGGTCTGGCGTTCCAAT
CCTTACCATGAGTCTGTGGAAGAACTGCGGGATCGGGTGAAAGGGGTCTCAGCCAAACCTTTCATTGAGACAG
TCCCTTCCATAGACGCACTCCACTGTGACATTGGTAATGCAGCTGAGTTCTACAAGATCTTCCAGCTAGAGAT
AGGGGAGGTGTATAAGAATCCCAATGCTTCCAAAGAGGAAAGGAAAAGGTGGCAGGCCACACTGGACAAGCAT
CTCCGGAAGAAGATGAACCTCAAACCAATCATGAGGATGAATGGCAACTTTGCCAGGAAGCTCATGACCAAAG
AGACTGTGGATGCAGTTTGTGAGTTAATTCCTTCAGAGGAGAGGCACGAGGCTCTGAGGGAGCTGATGGATCT
TTACCTCAAGATGAAACCGGTGTGGCGGTCATCATGCCCTGCTAAAGA--GTGCCC-
AGAATCCCTCTGCCAGTACAGTTTCAATTCACAGCGTTTTGCTGAGCTCCTTTCTACCAAGTTCAAGTATAGG
TATGAGGGAAAAATCACCAATTATTTTCACAAAA???????????????????????????????????????
???
???
???
???
???
???
???
???
???
??ATG
GAGGGAAAACACCAAACAATGAGCTTTCAGATAAGATTTATGTCATGTCTATTGTTTGCAAGAACAACAAAAG
GGTTACTTTTCGCTGCACAGAGAAAGACTTGGTAGGAGATGTTCCTGAAGCCAGATATGGTCATTCCATTAAT
GTGGTGTATAGCCGAGGGAAAAGTATGGGTGTTCTATTTGGAGGACGCTCATACATGCCTTCTACCCACAGAA
CCACAGAAAAATGGAATAGTGTAGCTGACTGCCTGCCCCATGTTTTCCTGGTGGATTTTGAATTTGGGTGTGC
TACGTCATACATTCTTCCAGAACTTCAGGATGGGCTATCTTTTCATGTCTCTATTGCCAAAAATGATACCATC
TATATTTTAGGAGGACATTCACTTGCCAATAATATTCGGCCTGCCAACCTGTACAGAATAAGGGTTGATCTTC
CCTTGGGTAGCCCAGCTGT-
GAATTGCACAGTCTTGCCAGGAGGAATCTCTGTCTCCAGTGCAAG????????????????????????????
???
???
???
???
???
???
???
???TGTGTCTCACCATCTTTCTACTC-
GCCACTACACTGAGCCCA--TGATGACCCAGTGCTCCA-CAAACATGCTTAGAATGGAAGT------------
----

------------------------------AAAACCTCAGGGATAAGA-----
CAGTTAAAGCAGATTCACTACAGTAACAAACCAATCAAGAAACTGCAATGAAAGTAAAGGTTT-
GGGTGACAAAATTTCCAGCTGTGTTCTAAGAAACATAACTACAAGCAGGAGTGCATTTAATCTGATTTT-
CCTCAAAGC--
CTCTCTAATGTGTAGGGAAAGACATTCATTTTCATTAACTTCATGTGAGCTAGAGAGAATCTGTGATTGAGGC

TCTAGAACCCCTGGTAAACATGCCAGCAAGCAAGGGCACCTTCYCCGGAGGTTCAATCTAGAATGTAAGGCTG
TCAGCRTTGGTAAGGAGTCTTGAACTCTAAATCCACAGGCTCAAGTCAGGAA-
CTCAGGAAGCCTATCAACACCTGGATACAATTTTGCCACCTTAACACTTTATTCCCTTTACTTGTGCCAAACT
???
???
???
???
???
?????????????????????????????????AAGGCCCTCAAAGATCTTTATTGCCATAAAGTAGAGTAAC
T-GTCTTTGACTTGGACCTTGTTTACTGTGAACTCTAATC-AAAGCAGGAGATGCAGCAGTCCTACTTA-
TTGCCATGTGGACTTGTGGAAAGGACACGTGTGACCCTTAAGAATCCAGTTCGGATTAGTGTTTGAAATTGAA
TTGGGAATGTTGTTCCAGGATGTGGAATGCAGCTGTGATCACACTT----ACC-
GAGCTTACTTTGACCTGTTTGTCAATAGCATGCAAAAAA-
TGCTTTGTTTGCCCTTTGCTTCTGCTTTTTTCAGGGAAGCTGCCAAAGAATGTCGACGTCGAAAGAAAGAATA
TGTAAAATGTCTGGAGAGCCGAGTTGCAGTGCTGGAAGTCCAGAACAAGAAGCTTATAGAGGAACTTGAAACC
TTGAAAGACATTT??
???
???
???
???
???
???
???
???
??TGTTTATATCTAAATGTATGGATTCCG
GCACCTAAACCAAAAAATGCTACTGTAATGATATGG-
ATTTATGGTGGTGGTTTTCAAACTGGAACATCATCTTTACATGTTTATGATGGCAAGTTTCTGGCTCGGGTTG
AAAGAGTTATTGTAGTGTCAATGAACTATAGGGTGGGTGCCCTTGGATTCTTAGCTTTGCCAGGAAATCCTGA
GGCTCCAGGGAACATGGGTTTATTTGATCAACAGCTGGCTCTTCAGTGGGTTCAAAAAAATATAGCAGCCTTT
GGTGGAAATCCTAAAAGTGTAACTCTCTTTGGAGAAAGTGCAGGAGCAGCTTCAGTTAGCCTGCATTTGCTTT
CTCCTGGAAGCCATTCATTGTTCACCAGAGCCATTCTACAAAGTGGATCCTCTAATGCTCCTTGGGCAGTAAC
ATCTCCTTATGAAGCTAGGAACAGAACGTTGACCTTAGCTAAATTGACTGGTTGCTCTAGAGATAATGAGACT
GAAATAGTCAAGTGCCTTAGAAATAAAGATCCCCAAGAAATTCTTCTGAATGAAGCATTTGTTGTCCCCTATG
GGACACTCTTGTCAGTAAACTTTGGTCCAACCATGGATGGTGATTTTCTCACTGACATGCCAGACGTATTACT
TGAACTTGGACAATTTAAGAAAACCCAGATCTTGGTGGGTGTCAATAAAGATGAAGGGACAGCTTTTTTAGTC
TATGGTGCTCCTGGCTTCAGCAAAGACAACAATAGTATCATAAGTAGAAACGAATTTCAGGAAGGTTTAAAAA
TATTTTTTCCAGGTGTGAGTGAGTTTGGAAAGGAATCCATCCTTTTTCATTACACAGACTGGGTAGATGATCA
GAGACCTGAAAACTACCGTGAGGCGTTGGATGATGTTGTTGGGGATTATAATATCATATGCCCTGCCTTGGAG
TTCACCAAGAAGTTCTCAGAATGGGGAAATAATGCCTTTTTCTACTATTTTGAACACCGTTGTGCTAATTGTG
AGAAAATATGCTACTTTGATCAAACTAGAATGAAGGAAATAAAAAGACACTGTGGCTCAACTTGTAATAAAGA
GCAGCTCATCCTCACAGCTTTGTGATTGTTGCTATAGTTATCT---
ATTCAACATGGGATGCACAATACAGTTAACGTTTCATTACCAGTAGCCTGTTGCGTGCTTATTGTTAAAAGGA
ATTTAACATACAAGTTCTTGTATGGGTTGTTCACCTAATTCTGATTCCTCGTGCTGACTGGCATGAGACAGGG
AGCTGGATTGTATTAAACTGGTTTTATTGTGATGTGACTATGATGGATGTGACAAGGGCCTCTATTATTCGCT
GGGGAGTGTGGCCGTTAATGAAGCCATTAGCTGAGCTAAGGCAAGTGAAGAAAATAGGGAAATTACCACTATT
CTGAAGCCATTAAGTTGTAATCCACCACTTTCTCTCCTTACTCAAAAAGGCATCTTCTTTTTAAGGCCTCCCT
???????????GGTTATTTCATACTTTGGTTGCATGAAGGCTGCCCCCATGAAAGAAGCAAACATCCGAGGAC
AAGGTGGCTTGGCCTACCCAGGTGTGCGGACCCATGGGACTCTGGAGAGCGTGAATGGGCCCAAGGCAGGTTC
AAGAGGCCTGACAT---
CATTGGCTGACACTTTTGAACACGTGATAGAAGAGCTGTTGGATGAGGACCAGAAAGTTCGGCCCAATGAGGA
AAACAATAAGGACGCAGACTTGTACACGTCCAGGGTGATGCTCAGTAGTCAAGTGCCTTTGGAGCCTCCTCTT
CTCTTTCTGCTGGAGGAATACAAAAATTACCTGGATGCTGCAAACATGTCCATGAGGGTCCGGCGCCACTCTG
ACCCTGCCCGCCGAGGGGAGCTGAGCGTGTGTGACAGTATTAGCGAGTGGGTAACGGCGGCAGACAAAAAGAC
TGCAGTGGACATGTCGGGCGGGACGGTCACAGTCCTTGAAAAGGTCCCTGTATCGAAAGGCCAACTGAAGCAA

TACTTCTACGAGACCAAGTGCAATCCCATGGGTTACACAAAAGAAG???????????????????????????
????????????AACGGCTATGAAAATCCAACCTACAAGTTCTTTGAGCAGATGCAGAACTAGACCCCC-
GCCACAGCAGCCTCTGAAGTTGGACAGCAAAACCATTGCTTCACTACCCATCGGTGTCCATTTATAGAATACT
GTCGGAAGAAACAAACCCCTCTGTTTTATGATTTACTCATTATCGCCTTTTGACAGCTGTGCTGTAACACAAG
TAGATGCCTGAACTTGAATTAATCCACAAATCAGTAATGTATT----CTCTCTCTCT----TT----------
--------ACATTTTGGTCTCTACACTACATTATTAATGGGTTTTGTGTACTGTAAAGAATTTAGCTGTATC-
AAAC-TAGTGCATGA-
ATAGATTCTCTCCTGATTATTTATCACATAGCCCCTTAGCCAGTTGTATATTATTCTTGTGGTTTGTGACCCA
ATTAAGTCCTACTTTACATATGCTTTAA-GAATCGATGGGGGA---
TGCTTCATGTGAACGTGGGAGTTTAGCTGCTTCTCTTGCCTAAGTATTCCTTTCCT-
GATCACTATGCATTTTAAAGTTAAA-CATTTTTAAGTATTTCAGATGCTTTAGAGAGATTTTTTTTC--
CATGATTGCATTTTACTGTACAGATTGCTGCTTCTGCTATATTTGTGATATAGGAATTAAGAGGATACACGTT
TGTTTCTTTGTGCCTGTTTTATGTGCA????????????CGGAGAGCACTAAGCCACTTCCAGCCCTGCTGTG
AACCAGGACTGTGGGAGCCATCTTTTTCATGTGGTCAGCTTTGGAAGCATCTACATCCACCACTTTAGAAGAC
AAGTCTAGTGGCTTATCTGTGACGTCTTTGGTAACGGTCTGCTTCTCCAACAGAGGAGGTGAGCTGCCGTCTT
TTCTGTCTTGAACCGCTGTCTTCCGGGCATGCCCGGGCACTGGCTGGGCACCTTCGCTCCCTTCCGGAGCCTT
GGGATACTTGCCGTTGGAGAGCCTGGCCGCGGGGAACTCGCTGCTGACTGTCATGTATGGCTTCGACAGGGCA
ACTGAAGGAGAGGTGGAGATCCTGGCATAGTGCTTGTGGAACTCCGAGTAGGTGTCTGCAGAAGGCTGGGTGG
GAAGGTGGACTCGGGGTGAGGGCCGAGGCGAGGGAGGCAACAGGAGAGCTGTGTCCCCCGGCAGGCCACTGGT
GACCGCCTTGGCAGAGGGAACCCTGGGCTGCTTACTGTTCTGGATGTGAGGATAGGCGTGGGAATCAACGGGA
TTCCCAGGGCTGACGCCCATCTTCCACGGGAGGCTTTTGTCTGCGCAATGGACGAGAGGCGGGATGGCTGGGG
AGGCCGAAGGTGTCGAGAGCCTCATGGGTGATGCCAAGGATGATGGGATGTGGGGACCGACGTAGTGAGGTGG
TGGCAGGTAGAGAAATCGCTCCCCGTTGGTGCAGACTGGAGAATACAGCGGCTGGGCCAAGCTGTAGGACTGC
TG????????????TGCCAGGACCTTTGTAATTCAAGTGCTTTTTACAGTAGTAGCAAATGTTACATAAGCCCA
AACATGGAGCA---
TATTTGGAGTTGGTGGGCAGACCCAAATCATGATGCAAGCCTGCAGTAGCAAGTGTGCAATGAAGCACAACCA
AATTCTGTGAATGGAAGGTAGCAAGACGCCTCTCCAAAGCAGCCCTTCTGCGCAGGAGCTGAAAGCTCCACTG
GGTAAGTTTTCTGAGGCAGTTTTCAGAGGCTGGGCTTGGCTCAGGGCCCAGCTCTGGATGGGCCTGTGGACGG
TGGGGT----
AGGTCTTTGCTAATCTTACATAGAACCAAGCAGAGTTGGGAAGAGTTTATGCAGGCCTCTTTCCTCCTGGCGT
TTGTCTTGCCCTGCCAGCAGGCCAGGAGGG-
CTGCGCTGCTCAAGTAGTGGAGGGAGGGAGCCTGCAGAGCTAGGGGATCAGCGTGGTTAATCGCAGCCACAGG
GATTAGCTGCTGCAAGCTGTCCACAGGAGCCATTCTGAAATGTCATTTGAGTGATCTGGCCGTCTACTGGAGG
AGGCCCTCGGAGCAGCTGTCTCCAGGAGCAGCTTCTGAGGGAGTCTTCCGGGAGGAGCAGCGTCTCTACCATC
GTCCTCGTCCATGTCCTGGTCACATTTCCATTCCTCATCTTCATTTAAAGTCGGCAGGTATCCAGATATGCCC
TTCCCTGTCCCTGACCCAGAGCTCTGGTCACTACAGACTTTCGGGCTCTCCGAGCCTGTCCTCGTATATTCCA
AATAAATATCAGACTTAAGGAAAGAGGGATAGGTGTTTTCCTCCATGGTGGCCTGGATTTCTGTCTGGGCCTG
GTCAAACATGGCAGGATCGATCAGCTGCTTCATGATGCAGCCCTTTATGAAGCTCTTGGTGGCTGGCTTGGTC
TGCCGGGACACGATGCCATTGTTATCAAGGATGTACTTTCGATAGATGGCTCTTGCCAGCTTCAGCCTCTTTT
CCTCGTTCGAATCACAGGGCTCCAGCTTCCTGAAGCCAGTGCAGGCAAACCAGAAGTCCAGCAAATCGGCACA
GCCCTCCTGCTTCAGGAAAGTCCTGAACAGGCTTATCCCATCTTGGTCATCCAGCAGGGAGTGCAGTGACTCA
GCCCACTTCAAGTATGGTGGGGTGGGGGAGGCACTGCCCTCAGGCTCATACCCCAGGTCCAGATCCGAGCGCC
TCGGAGTGGCCGTCGAAGTCTCAGCTTTAATGCCAACACCTTTCCCAGAGCAGAAACTGTAGCTGGCGGGCCT
CGGGTCTGTGGACACCAGTTCTCCCTCCTCACCAGGCACTGGGGGTCGGGGAGCATCTTCGGTGAAACTTGCT
CCGAGGTCCAAGGGGAAACCCTGCTCTTGGATATTCATTTTGGGACTCTG--
CGTCAATGAACAATGAGCGCTGCACCCTAATACATCAGTACTTA-
CAGCTCCAAAGTGAATCAATCTGTCCTGTTGAAACCAT?????TCAAAATGTTACCTAACTTTAAGTGAACAA
AATTATTACATGGATCTATGTATACCAGCATAAAGCAGGTATAATTTACAGAGAAGTGGAAGAAGT-
ACCTAGGTTATTTACATGACAGATTACTATCCTGTGTTGTTCTGAATGTTTCTTTG-
CATATTAAAATTTATTTATAGGTGCAGTTAAGCTTT--ACTCTTAG----
CATATATTTGGCTTGGAGTCAGTCACCAAAGAAGAAATGC-
GGCCTTGATTTTTATGAGTTATTTGCTATACATTTCTAAATTTATGTTCTTTTATCACTGCTTGCTTGATTTA
TTTTTTTAAGCTGGAGGAG---------GTTCATACTGAACCAAACCA-CTTCTGTTGATTAGGA--------
--CATTACTATAACTCTTAACATCGAAAAGCAGTAAAAGGAATGTTAATAA-----

AAGTATTTGCCCAGTAA-TGTTCAGAACACAAGCTTTAAATTCAT-GAGGAAACCA---------
GAGGTTTGATT-
AAGCACTCATACTGCTTTCTTTTCCTTTCTTAGCAATAATTATTGGCCCTGATGGTCATCCTTTGACTGTCTA
TCCTTGCATGATTTGTGGGAAGAAGTTTAAGTCTAGAGGTTTTTTGAAAAGACATATGAAAAACCATCCCGAA
CACCTTGCCAAGAAGAAGTACCACTGTACTGACTGTGATTACACTACCAACAAGAAGATAAGTTTACATAACC
ACCTGGAGAGCCACAAGCTAACCAGCAAGGCAGAGAAGACCATTGAATGTGATGAGTGTGGGAAGCATTTTTC
TCATGCGGGGGCTTTGTTTACTCACAAAATGGTGCACAAGGAAAAAGGGGCCAACAAAATGCACAAGTGTAAA
TTCTGTGAATATGAGACAGCTGAACA???
??????TACCCGCTATAGCTTCTGCAAACAGTAAAGATACCACTAAAACCCCTTCTGCAGTTCAAACTAGCAC
CAGCCACAACCCTGGACTTCCTCCTAATTTTAACGAATGGTACGTCTGAGGACAAACACAAACCCTGTTAACT
ATAGAATGGACCAAATGCATTTTTAAAAGAAAACTGAGACCAATCAGATGGAAATGGAGTTTTAAGGCAAGAG
GCCATATATAGGGTTACATCTTGTTAATTGCAATTGTCCAGGAAGGTTTTGGGCAAGATCCAAAAGTAGCCAT
GCCCTTTTCTCAGGATTAGAAAATATGTTTTGGCATTTGAAGCATTTTTG--
ACAAAATCTTTACACTGCTTTTTCTTCCCCTTCCTCTTGCTGTCTGCACATCCCATTCTTAAACTCCTGCAAT
TCATTTTAACACTTGTCCTGTTTCTCGAGAGGAAGTTATAGAAGGCTTGTTGGTGGTGGTGATGTTAAACTGA
TGGAAATTCTTTTTCGCCTTAGTGGTGATTGTTTAAACTCTCACAGTCTTAAACCGTGCCAAAGTCCTGTTAT
A
Saguinus
?TACCCCCATGTTTGGCTGGAACATGAAGCTGACCTCAGAGTACTATAGAAATGTGACCTTTCTGTCATGCCG
GTTCCCTT-
CCGTCATGAGCATGGACTACATGGTATACTTCAGCTTCTTCACCTGGATTTTCATTCCCCTGGTCATCATGTG
TGC--AATCTATCTTGACATCTTTTACAT-
CATCCGGAACAAACTCAGACAGAACTTGTCTAACTCCAAAGAGACAGGTGCATT---
TTACGGACGGGAGTTCAAGACGGCGAAGTCCCTGTTTCTGGTGCTTTTCTTGTTTGCTTTGTCATGGCTGCCT
TTGTCCATTATCAACTGCATCACCTACTTCCA--------------
TGGCGAGGTGCCACAGCTTGTGCTGTACTTGGGTATCCTGCTGTCCCATGCCAACTCCATGATGAACCCTATC
?????????????????????ATGTTCACAAATCGCTGATTATTTTCAACCAACCACAAAGACATCGGAACAT
TATACCTACTATTTGGCGCATGAGCGGGAGCTGTGGGAACAGCCCTAAGTTTCCTTATCCGAGCAGAACTAGG
CCAACCTGGGAGCTTGTTTGAGGATGATCATGTCTATAATGTTATCGTTACATCCCACGCATTTATTATAATT
TTCTTCATAGT---TATACCCATTATAATCGGGGGTTTTGG---
CAACTGACTAATCCCCCTAATAATTGGCGCCCCAGACATAGCATTCCCTCGAATAAATAATATAAGCTTCTGA
CTTCTACCCCCA--TCACTCCTCCTTCTACTTG-
CATCCTCAACCTTAGAGGCTGGCGCTGGAACTGGCTGAACAGTATACCCACCCCTAGCAGGAAACATATCACA
TCCAGGCGCCTCTGTAGACCTAGTTATTTTCTCACTGCACCTGGCAGGTGTGTCTTCCATCTTAGGGGCTATC
AACTTCATCACCACAATCATTAACATAAAACCCCCTGCCATAACCCAGTACCAAACCCCCTTATTCGTATGAT
CCGTTTTAATCACAGCAGTTCTTCTTCTACTCTCCCTGCCTGTCTTAGCTGCCGGAATTACTATACTACTAAC
TGACC--
GAAATCTTAACACTACTTTCTTCGACCCTGCAGGCGGCGGTGATCCTATCCTGTACCAACACCTATTCTGATT
CTTTGGTCATCCCGAAGTATATATTCTTATCCTACCAGGGTTCGGAATAATTTCACACATTGTAACATACTAC
TCTAATAAAAAGAGCCGTTTGGATACATAGGCATAGTTTGAGCTATAATATCTATCGGATTTTTAGGGTTTA
TCGTATGAGCTCATCACATATT-
TACAGTCGGAATGGATGTCGACACCCGCGCATACTTCACATCAGCCACTATAATCATCGCTATTCCTACTGGG
GTGAAAGTATTTAGTTGACTAGCCACCCTACACGGCGGCAATATCAAATGATCCCCCGCAATACTATGGGCCC
TAGGCTTCATCTTCCTCTTTACCGTAGGAGGGCTGACAGGAATCGTATTAGCTAACTCATCACTGGATAT-
TGTACTACATGATACATACTATGTAGTAGCCCACTTCCACTATGTTCTATCTATGGGGGCAG-
TATTTGCCATCATAGGGGGATTTATCCACTGATTCCCACTATTCTCAGGTTACACACTTGACCAAACCTATGC
TAAAATTCACTT-
CACTATTATATTTGTAGGCGTAAACCTAACCTTTTTCCCACAACACTTCCTCGGATTATCAGGAATACCCCGA
CGATATTCAGACTACCCTGACGCCTATACTACATGAAATATCATCTCATCTGTAGGCTCATTAATCTCACTGA
CAGCAGTGATACTAATAATTTTTATAATCTGAGAAGCATTCTCTTCTAAGCGCAAAGTTTCAACCATTGAACA
ACTATCAACTAACCTAGAGTGACTACACGGATGTCCTCCTCCATTCCATACATTTGAAGAAGCCACTTATGTA
AAAGCCTTAAGCGAAAAAGGAAGG????????????ATGGCAACACCAGCCCAATTAGGCCTACAAAACGCTA
CATCCCCCATCATAGAAGAACTTATCGCCTTCCACGACCACGCTCTTATAATTATTTTCTTAGTTAGCTCACT
AGTCCTATATACTATTTCTCTAATACTTACTACTAAACTCACCCACACAAGCACCATAAATGCCCAAGAAATT

GAAACAATCTGAACCATCCTGCCCGCCCTTATCCTAATCACAATCGCCCTCCCATCCCTACGCATCCTATACA
TAACAGATGAATTCAACAAACCTTACTTAACACTTAAAGCCATCGGCCACCAATGATACTGAAGCTACGAATA
CTCCGACTACGAGGACCTATACTTTGACTCTTATATCATACCAACATACTTTCTAGAACCTGGGGAATTTCGA
CTCCTTGAAGTAGACAACCGAACAACTTTGCCAATAGAAGCAGACATTCGTATACTAATCACATCACAAGACG
TCCTACACTCCTGAGCTGTACCATCGTTAGGTGTAAAAACAGATGCAATTCCCGGACGTTTAAATCAAGCCAT
ACTAGCCTCCATACGACCAGGCCTATTTTATGGTCAATGCTCAGAAATTTGCGGATCTAACCACAGCTTTATA
CCTATTGTCCTAGAATTCATTTATTTCCAAGATTTCGAAGTATGAGCCTCATACTTATATATCGTAT??????
ATGACCTCTCCCCGCAAAACTCACCCACTGGCAAAAATTATCAATAACTCATTCATTGACCTCCCAACACCAT
CTAACATCTCTTCCTGATGAAACTTCGGCTCACTTCTAGGTACTTGCCTAATAATCCAAATCACCACAGGCTT
ATTATTAGCCATGCATTACACATCAGACACCGCCACTGCCTTCTCCTCAGTCGCTCACATTACCCGGGACGTA
AACTATGGCTGAATGATCCGCTACCTACATGCTAACGGCGCATCCATATTTTTCATCTGTATATTCCTACATA
TCGGCCGAGGCCTATACTACGGATCTTTTCTCTTTCTGAAGACCTGAGGTGTCGGTACAATCCTACTACTTAC
CACCATGGCAACCGCATTCATGGGATACGTCCTCCCATGGGGCCAAATATCATTTTGAGGGGCTACAGTTATC
ACAAACCTTCTATCTGCAATTCCATATATCGGATCTGATCTAGTCCAATGAATCTGGGGCGGATTCTCAGTAG
ACAAAGCCACCCTTACACGATTCTTCACCTTCCACTTCATCCTGCCCTTCATCATCGCAGCTCTAGCAACCAT
CCACCTTCTCTTTCTGCATGAGACAGGTTCAAGTAACCCGTCAGGAATGACATCAGAACCCGACAAAATCCCA
TTCCACCCATACTATACAATCAAAGATATCCTCGGACTAATATTCCTCCTACTTCTCCTAACAAGCCTAACTC
TGTTTTCACCAGACTTGCTAACAGACCCAGACAACTATACACTAGCCAACCCCCTAAATACCCCACCCCACAT
CAAACCTGAATGGTACTTTTTATTTGCATACGCAATCCTACGGTCCATTCCCAATAAACTAGGCGGCGTCCTA
GCCCTCCTCTTATCTATCCTCATCCTAATAGTCGTCCCCATACTACACACATCCAAACAACAAAGCATGGCAT
TCCGACCAGTTACTCAAGCCCTATTCTGAACGCTAGTAGCCGATCTGCTAACACTCACATGAATTGGAAGCCA
ACCAGTCGAATATCCATACATAACTGTTGGCCAAACAGCATCCATCATATACTTTCTTATCATCATCATCCTA
ATTCCACTTTCTGCCCTTATCGAAAATAAATTACTTAAATGATAA?TTAGATTAAATTTATTCAAGGGAATGA
GAATTTATGGGTATAACTGTTAGCTGGAAAGCCCTTCCTCACCTCCAATCCTCCTTTCAAACTACATTTCC-
TCCTAAAATTTCTCTGTGGGATGAAATGTTCAATTATTAGTCAATCAATC----
TCTTATACGTTATTTGGCTTTACATAT----
GCCCTTGATAGTTCTTGGAAAATAATCCAATATTGTGTTGTTTTGGTGTCTTTGTATTGAAAATGGAAGCATT
TTGTATGAATCACCTCTTTAACTGTGAGGAGTTGAATTGTCTTATTTCTGCTTGCCTAATGACCAGGAATAAA
--GACATCACAGTCTCTAACCCCACTTTTT-
GGTGGAAAGAAAATCTAAAAGTAGCAACATTTAATGGAACAAAATATCAATAATCTAAGAAACTCCCCTGTGA
AACTGTAATATTATTTT-
AAAATCAACATTGTTCTAAGGTAGCTGAAATGTGTGATTTTTCTATTTGGCCTTTTCACTGACAATATACCCA
TAAATCTTTGGTGGTGATTTTCTCTCCAAAGTTAATTTCTGGGCCATTGGTGACTGCACTATCTATTGTTCTA
AATTGTTGTCTTAATTGCATTCTTTAAAAATGTAATGTTGAAGGCACATAATATTGAAGATGACTCCAGGATG
AACCCTGAGTTCACAGACCGAA-
TAAAACAGCTTGATAAAGAGGCGTCTTACTACCGTGATGAGTGTGGCAAGGCCCAAGCGGAAGTGGACCGGTT
GCTGGAGA?????????GAAGAGCTGCATCAGGCAAGTACTTTGATCTTTCTTGGAGCAGGTTCCTTCAAAA
CGCTTCTCCAGAGGACGAAAGTCCCTCCAGTCTGGCTCGCTGCTTGCAGTGGTGGGGAAAGCTGAGGTAATTC
CCCGGTGGGAAATCTGCGAGGCCCCTGATACCCCGG--------
CCACCAGGCCCATCACTGATGGGCCTAAGGGCCTCATCTGATACTCTAAGATGGGTTTCTCCTGTCGGATCTG
GGCCTCTCGGACTTGAGCCTCTCTAGAACGAGCCTCTCGGGCCTGGGCTTCTCGGGTTCTGGCCTCCCTGGCA
TGACCTTCCCAAGTGTGGGCCTCCCTG--
GCATAGGCTTCCTTGCCATGAGCTTCTCGAGTGTAGGCCTCCCTGGTGTGGGCCTCCCTGGCCTGGGCTTCTC
GGGCACGTGCCTCCCGGGCCTCAAGCTGCTCCCTCCGAAGCTCCCAATACAACAACTGTTTCTGGATGGTCA-
CTAGTCGTTCCTCCTCTGTCTCCATTGCCCCAGGTGGCCGGGAGGAAGAAAAGGGCTCAAAGTTCAAGAATGG
GTCAAACATCTCAGAGTTTCGACCACGGAGGTCATAGAGGCAGTCATCCCCAGGTGGAGAGTTCTCAAGACTG
TCATCTGGCTCATAGAACTCATATAGGGCATCTCCACTGTAGCTGTCTCGGGGCAGACAATCCCTGCGGACAA
GCCCCAGGGTCTCACCTGAATCATCCTCAA??????AGCATCACTAATTTTGATGAACTAAAAATGGTTCCTG
AAGGTCAGATTCATGTACAGTGAATGTTCTCCACTGTTGTGGTGGGCATGTTTCATGTGTGGGGATGTTGGTG
TGCAAGTTTCCTGTGTTTGGAAAACACTAGCTAGAACATTCGAAAAATTTCATTAACCCAGCTATGGAAGGTC
ATCTATGAATTCTTCCTATTCCAGTGGCCTTTTTAAGTGTAGATGGAGATACACTGTAATTTAAAACAGATGG
GCATGTCTGAATATTTTCATATGCAAAATGGGACAGATGTATATGAATGGATAAATTATCCTTTTAAAAAAAT
ACTTTGTAGCACGAAATATCACCAATGATTGGAAGAGTGAACCTGCATTCGGTCTAGGGCAAACACCAGGAAG
ACATTATCAAAACATCTAA-CTATCTACAAACTAATTCCAATTAGTCTACTTTGTCTTCTTACAGCAAAAA-

CTTTTTTGATTTTTAAA---TATGCATTGGGTGCCAATCCGTATCCTTT-
GAGCTGCTAAAGCCAGCATATCACAATAGAAGTTTACTTTTCCTTGAGAGTTGCATCATGTTTATAATCAACC
TCCTCGTAGTAATCCTATCAGCTCTAGTCGCCATGGCGTTCTTAACACTCACAGAACGAAAAGTGCTAGGCTA
CATACAATTTCGAAAAGGTCCTAACATTGTAGGCCCCTACGGAACGCTCCAACCAATCGCAGATGCCATAAAA
TTATTCACAAAAGAACCTCTACTCCCTACTTCATCCACCTCAACACTATACCTAATCGCTCCTACCCTAGCCC
TCTCAATCTCCCTACTTTTATGAACACCTCTTCCCATGCCATACCCCCTAATAAACTTCAACCTAGGTCTCCT
ATTTATCCTAGCAACATCAAGTCTCGCCGTATACTCAATCCTATGGTCCGGGTGAGCATCTAACTCAAACTAT
GCACTAATTGGCGCACTACGAGCTGTAGCCCAAACAATCTCATATGAAGTTACTCTCGCTATCATCCTCCTGT
CTGTCCTACTAATAAGCGGTTCATTTAACCTTCAATCTCTCATTACTACACAAGAACACTCCTGACTCCTATT
TCCATCATGACCCCTAGCTATAATATGGTTCATCTCAACACTAGCAGAAACCAACCGAGCCCCCTTTGATTTA
ACAGAGGGCGAATCTGAACTAGTCTCAGGATTCAACATTGAATATGCCGCAGGATCATTCGCCCTATTCTTCA
TAGCAGAGTATATGAATATTATTATAATAAATGCTCTGACCACTACTATCTTCCTAGCAGCACCCCATAATAC
AGCAGCACCAGAAACCTATACAATCAATTTTATAACTAAAACTCTACTACTAACCACCCTATTCTTATGAATT
CGAACAGCATACCCTCGCTTCCGCTATGATCAATTAATACATCTACTATGAAAAAATTTTTACCACTTACAC
TAGCACTATGTATGTGATATATTTCAATACCCACCCTAACATCCGGCATTCCACCCCAAACATA?ATCAACCC
CCTAGCCCACCTCATCATCTCCTTTACCATCATAACAGGAACCGCAATCACAATCCTAAGCTCACACTGATTC
CTAATCTGAATAGGCCTAGAATTAAATATACTAGCCATTATCCCAGTATTGGCCAAAAACATAAAACCCCGAT
CCACAGAAGCATCAACTAAATATTTTTAACCCAAGCAACAGCATCATTAATTCTCCTAATAGCCATTATCCT
CAACAACTTAATTTCCGGGCAATGATCAATTAACCCTCCCC-TAAGTATACT--
ATCTACAATTATATTAATTGCCCTAACCATAAAACTGGGAATAACCCCATTCCATTTCTGACTCCCAGAAGTA
GCTCAAGGAATTCCCCTAATCCCAGCTATAATTATCCTCACATGACAAAAACTTGCCCCCATGTCAATTATGA
TTCAAATCTCTTCATCAATGAACACAAGTGTACTCCTGATAATTTCAGTTTTATCAATTATAGCCGGCAGCTG
AGGTGGACTTAACCAAACACAACTTCGCAAAATCCTAGCTTACTCCTCAATCACCCACATAGGATGAATATTA
GCAGTGCTGCACTACAGCCCAAACATCACTATTCTAACCCTAACTATCTACATTCTATTAACAACTTCTTCAT
TCTTAACCTTCTACTCAAACTCAAACGTAACAACCCTATCTCTATCACATACCTGAAATAAATTAACATGAAT
AATACCCATAATCCCACTAATAATAATATCCCTAGGAGGACTTCCTCCCCTAACAGGCTTTTCCCCTAAATGA
ACCATTATACTAGAACTTACAAAAAACAACTACCTAACACTCCCTCTCATGATAGCCTTACTAACACTAATAA
ATCTATACTTCTACATACGCCTAACATACTCCATTTCAATAACAATATTCCCAACATCTAATAATACTAAAAT
CAACTGACAACTAAAACACATTAAACCAATACCACTCCTATCCCCCCTTATAATCTCCTCCACCCTCCTCTTA
CCCCTAACCCCCCTAATACTTATAGTAT?????GTTGGCAGCATAAATAATAAAGCATGTTACTTAAGCAATT
TTACATTTACTGTATTAGTAAGTAAATGCAATGGCCACTCTTATTCTCGAACAATAAAAACTACCCAGCCTTT
TCATGAAAGAT-GATTGCAATTTCCATTGGCCTGTATATGCAATAAAAAG---
CCATAACTCATCAAAATTTTAGCTATTTTCTCTTAAGATA--
GAAGAGCGTTTTGCTTGAACATTGAACGTGACATTAAATATT-
CATGAAACTGAATAGAGTTTAAGTACTAAG---
TAAAACTCTCAAATTCTTTTCAACTAGATTGAAATTCTGTTTGAACCAGGTAAAAATTACCACTAACCTGAAG
AGGTGTTCAAAAAC-
ATCGTAAAGAGATTGTACAATACGTTCAAAGTGCTACAGTTATTTGGGATAGATTTTTCTGAAAAGCCTGTTC
CTCCTTTGAAATTGATTCATGATAACAGCCTCCATTTGGGTCAAACAGTATTTAACCGCTTGTAAA-
TTTTAATT-
AAATGATTTGTCACTTTGAACACATAGTTTGTCCCCAAGACCTTGAGGATTACTTGAATGGCCCCTTCACTGT
GGTTGTGAAGGAGTCTTGTGATGGAATGGGTGATGTGAGTGAGAAGCATGGGAGTGGGCCAGTAGTCCCGGAA
AAGGCAGTCCGTTTTTCATTCACAATCATGAAAATTACTATAGTCCACAACTCTCAGAACGTGAAAGTGTTTG
AAGAAGCCAAACCTAACTCTGAACTGTGTTGCAAGCCATTGTGCCTTATGCTGGCAGATGAGTCTGACCACGA
GACCCTGACTGCCATCCTGAGTCCTCTAATTGCCGAGAGGGAGGCCATGAAGAGCAGTGAATTAATGCTTGAG
ATGGGAGGCATTCTCCGGACTTTCAAGTTCATCTTCAGGGGCACTGGATATGATGAAAAACTTGTGCGGGAAG
TGGAAGGCCTTGAGGCTTCTGGCTCAGTCTACATCTGTACTCTTTGTGATGCCACCCGTCTGGAAGCCTCTCA
AAATCTTGTCTTCCACTCTATAACCAGAAGCCATGCCGAGAACCTGGAACGTTATGAGGTCTGGCGTTCCAAC
CCTTACCATGAGTCTGTGGAAGAACTGCGGGATCGGGTGAAAGGGGTCTCAGCCAAACCTTTCATTGAGACAG
TCCCTTCCATAGATGCACTCCACTGTGACATTGGCAATGCGGCTGAGTTCTACAAGATCTTCCATCTAGAGAT
AGGGGAGGTGTATAAGAATCCCAATGCTTCCAAAGAGGAAAGGAAAAGGTGGCAGGCCACATTGGACAAGCAT
CTCCGGAAGAAGATGAACCTCAAACCAATCATGAGGATGAATGGCAACTTTGCCAGGAAGCTCATGACCAAAG
AGACTGTAGATGCAGTTTGTGAGTTAATTCCTTCTGAGGAGAGGCACGAGGCTCTGAGGGAGCTGATGGATCT
TTACCTGAAGATGAAACCAGTATGGCGGTCATCATGCCCTGCTAAAGA--GTGCCC-

AGAATCCCTCTGCCAGTACAGTTTCAATTCACAGCGTTTTGCTGAGCTCCTTTCTACCAAGTTCAAGTATAGA
TATGAGGGGAAAATCACCAATTATTTTCACAAAATAGATGTTGCTTATGGCACCCGACAGTTGTCGTTATGTT
TGGAAACACTGCCGGCCCATGGAGATGAGGATAAAGTGGACATTTCTCTGCTGCATCGGGGTGAGAATCTTTT
CGAACTGCACATCCACCAGGCCTTCCTGACATCTGCCGCCCTGGCTCAGGCTGGAGACATGCAACCTACCACT
TTCTGCACCTATTCCTTCTATGACTTTGAAACCCATTGTACCCCATTGTCTGTGGGGCCACAGCCCCTCTATG
ACTTCACCTCCCAGTATGTGGTGGAGACAGACTCTCTTTTCTTACACTACCTTCAAGGGGCTTCAGCCCGGCT
TGATCTCCACCAGGCTGTGGCCAGTGAGCACAACACTCTTGCTGCAGGATGGATTTACTTTGACAGGGTGCTA
GAGACTGTGGAGAAAGTCCATGGCTTGGCCACACTGATTGGTAAGTGTTGTCGGCTTCCTGCAGCTCCTA-
GCACCAATGCAGAATTTCCCAAACTCTATAGCTGCTTTTCTGGTCATTTCTTATTTTCTTCCTTTTTCTTTGT
TCTTGATCATTGCCACACCGTCTATATTCTCCATGATTTCA-
TACTTGCTGCTGCCCAGGAGCTGGTGGAGAAGAGTTTGGGGTTCTAGAGTACTGGATGAGGCTGCGTTTCCCC
ATAAAACCCAGCCTACAGGCATGCAATAAACGAAGAAAGCCCAGGTA?????????????????????????
???????????????????????????TTCATGGAGGGAAAACACCAAACAATGAGCTTTCAGATAAGATTTA
TGTCATGTCTGTTGTTTCCAAGAACAACAAGAAGGTTACTTTTCGCTGCACGGAGAAAGACTTGGTAGGAGAT
GTTCCTGAAGCCAGATATGGTCATTCCATTAATGTGGTATACAGCCGAGGGAAAAGTATGGGTGTTCTTTTTG
GAGGACGCTCATACATGCCTTCTACCCACAGAACCACAGAAAAATGGAATAGTGTAGCTGACTGCCTGCCCCA
TGTTTTCCTGGTGGATTTTGAATTTGGGTGTGCTACATCATACATTCTTCCAGAACTTCAGGATGGGCTATCT
TTTCATGTCTCTATTGCCAAAAATGACACCATCTATATTTAGGAGGACATTCACTTGCCAATAATATCCGCC
CTGCCAACCTGTACAGGATAAGGGTTGATCTTCCCCTGGGTAGCCCAGCTGT-
GAATTGCACAGTCTTGCCAGGAGGAATCTCTGTCTCCAGTGCAA?????????????????????????????
???
???TGTGTCTCAGCATCTTCCTGCTC
TCCACCATACTGAGCCCAAGATGACCCAGTGCTCCACAAACATATGTAGAATGGAAGTAAAACCTCAGGGATA
AGAAAAGGTAATTAAAGGGCCAGGCGTGGTGGTTCATGCCTGTAATCCCAGCATT-
TAGGAGGCCAAGGCGAGTGGATCACAAGGTCAGGAGTTCAAGACCAGCCTGGCCCAAATGGTGAAAACCCATC
TCTACTAAAAATA---CAACAATT-----
AGCTGGA--
---------------------TGTGGTGGTGGGCACCTGTAATTCCAGTTACTTGGGAGGCTGAAGCAG-
AGAATTGCTTGAACCTGGGAGGCGGAGGTTGCAGTGAACCGAAACCACGCCACTGCACTCCAGCCTAGACAAC
A-
GAGTAAGACTCCATCTCGAGGAGGAGGAGGAGGAGGAAGAGGAAGAAGAAGAAGAAGAAGAAGAAGAAGAAGA
AGAAGAAGAAGAAGAAGAAGAAGAAGAAGAAGGAAGAAGAGGAAGAAGAAGAAGAAGAAGAAGAAGAAGAAGA
AGAAGAAG---
AAGAAGAAGAAGAAGAAGAAAAGATAGTTAAAGCAGATTCACTATAGTAACAAACCAGCCAAGAAATTACACT
GAAAGTAAAGGTTTTGGGTGACAAAATTTCCAACTGTGTTCAAAGAAACGTAAGTTCCAGCAGGAGTGCACTT
AATCTGATTTT-CTTCAAAAC--
CTCTCTAATTTGCAGGGAAAGACATCCATTTTCATTAGCTTCATGTGAGCTAGAGAGAATCCATGACTGAAGT
GCTAGAACCTCTGTTAAACACGCCAGCAAGCAAGGGCACCTTCCCCAGAGGCTCAACCTAGACTATAAAGCTG
TCAGCATTGGTAAGGAGCCTTGAACTCTAAACCCATAGACTCAAGTCAGGAA-
CTCAGGAAGCCTATCAACACTGGGATACAGTTTTGCCACCTTAACACTTTATTCCCTTTACTTGTGCCACA??
???
???
???
???
???
???????????????????????????GGAACTCAAGGCCCTCAAAGATCTTTATTGCCATAAAGCAGAGTAAC
TTGTCTTTGACTTGGATCTTGTTTACTGTGAACTCTAATC-AAGGCAGGACATGCAGCAGTTCTACTTA-
CTGCACTGTGGACATGTGGAAAGGACACTTGTGA---------
ATCCGGTTTGGATTAGTGTTGGAAATTGAATTGCAGATGTT---
CCCGGATGTGGAATGCAGCCGTGGTCACACTTGCCTACT-
GAGCTTACTTTGATCTGTTTGTCAGTAGCATGCAAAAAA-
TGCTTTGTTTGCCCTTTGCTTCTGCTTTTTTCAGGGAAGCTGCCAAAGAATGTCGACGTCGAAAGAAAGAATA
CGTAAAATGCCTGGAGAGCCGAGTCGCAGTGCTGGAAGTCCAGAACAAGAAGCTTATAGAGGAACTTGAAACC
TTGAAAGACATTT??

???
???
???
???
???
???
???
???
??TGTTTATATCTAAATGTGTGGATTCCA
GCACCTAAACCAAAAAATGCCACTGTAATGATATGG-
ATTTATGGTGGTGGTTTTCAAACTGGAACATCATCCTTAACTGTTTATGACGGCAAGTTTCTTGCTCGGGTCG
AAAGAGTTATCGTAGTGTCAATGAACTACAGGGTGGGTGCTCTAGGATTCTTAGCTTTGCCAGGAAATCCTGA
GGCTCCAGGAAACATGGGTTTATTTGATCAACGGTTGGCTCTTCAGTGGGTTCAAAAAAATATAGCAGCCTTT
GGTGGAAATCCTAAAAGTGTAACTCTTTTTGGAGAAAGTGCAGGAGCAGTTTCAGTTAGTTTGCATTTGCTTT
CTCCTGGAAGCCATTCCTTGTTCACCAGAGCCATTCTGCAAAGTGGATCCTCTAATGCTCCTTGGGCAGTAAC
ATCTCTTTATGAAGCTAGGAACAGAACGTTGGCCTTAGCTAAATTTACTGGTTGCTTTAGAGAAAATGAGACT
GAAATAGTCAAATGCCTTCAAAACAAAGATCCCCAAGAAATTATTCTGAATGAAGCATTTGTTGTCCCCTACG
GAACTCTCTTGTCAGTAAACTTTGGTCCAACAGTGGATGGTGATTTTCTCACTGACATGCCAGACATATTACT
TGAACTTGGACAATTAAAAAAAACTCAGATTTTGGTGGGTGTTAATAAAGATGAAGGGACAGCTTTTTTAGTC
TATGGTGCTCCTGGTTTCAGCAAAGATAACAATAGTATCATAACTAGAAAAGAATTTCAGGAAGGTTTAAAAA
TATTTTTTCCAGGAGTGAGTGAGTTTGGAAAGGAATCCATCCTTTTTCATTACACAGACTGGGTAGATGATCA
GAGACCTGAAAACTACCGTGAGGCCTTGGATGACGTTGTCGGGGATTACAATATTATATGCCCAGCCTTGGAG
TTTACAAAGAAGTTCTCAGAATGGGGAAACAATGCCTTTTTMTACTATTTTGAACACCGTTGTGCTAATTGTG
AGAAAATATGCTACTTTGATCAAACTAGAATGAAGGAAATAAAAAGACACTGTGGCTCAACTTGTAATAAAGA
GCAGCTCATCCTCACAGCTTTTTGATTGTTGCTATAGTTATCTTCTGCTCAACATGGGATGCACAATACAGTT
AACGTTTCATTACCAGTAGCCTGTTGCGTGCTTATTGTTAAA-
GGAATTTAACATACAAGTTTTTGTATGGATTGTTCGCCTAATTCCGATTCCTCGTGCTGACTGGCATGAGACA
GGGAGCTGGATTGTATTAAACTGGTTTTATTGTGATGTGACTATGATGGATGTGACAAGGACCTCTATTATTC
ACTGGGGAGTGTGGCCGTTAATGAAGCCATTAGCTGAGCTAAGGCAAGTGAAGAAAATAGGGAAATTACCATT
ATTCTGAAGCCATTAAGTTGTAATCCACCACTTTCTCTCCTTATTCAAAAAGGCATCTTCTTTTTAAGGCCTC
CCT??
??????????????GCCTACCCAGGTGTGCGGACCCATGGGACTCTGGAGAGTGTGAATGGGCCCAAGGCAGG
TTCAAGAGGCCTGACATCGTCATTGGCTGACACTTTCGAACATGTGATAGAAGAGCTGTTGGATGAGGACCAG
AAAGTTCGGCCCCATGAAGAAAACAATAAGGACGCAGACTTGTACACGTCCAGGGTGATGCTCAGTAGTCAAG
TGCCTTTGGAGCCTCCTCTTCTCTTTCTGCTGGAGGAATACAAAAATTACCTGGATGCTGCAAACATGTCCAT
GAGGGTGCGGCGCCACTCTGACCCTGCTCGCCGAGGGGAGCTGAGCGTGTGTGACAGTATTAGTGAGTGGGTA
ACGGCGGCAGACAAAAAGACTGC??
???
???TTCTTTGAGCAGAT
GCAGAACTAGACCCCC-
GCCGCAGCAGCCTCTGAAGTTGGACAGCAAAACCATTGCTTCACTACCCATCGGTGTCCATTTATAGAATAAT
GTGGAAAGAAACAAACCCCTCTGTCTTATGATTTACTCATTATCGCCTTTTGACAGCTGTGCTGTAACACAAG
TAGATGCCTGAACTTGAATTAATCCACAGATCAGTAATGTTTCTCTCTCTCTCTCTCTCTCTCTCTC-----
---TCTTTACATTTTGGTCTCTACACTACATTATTAATGGGTTTTGTGTACTGTAAAGAATTTAGCTGTTTC-
AAACCTAGTGCATGA-
ATAGATTCTCTCCTGATTATTTATCGCATAGCCCCTTAGCCAGTTGTATATTATTCTTGTGGTTTGTGACCCA
ACTAAGTCCTACTTTAAATATGCTTTAA-GAATCGGTGGGGGA---
TGCTTCATGTGAACGTGGGAGTTTAGCTGCTTCTCTTGCCTCAGTATTCCCTTCCT-
GATCACTATGCATTTTAAAGTTAAA-CATTTTTAAGTATTTCAGATGCTTTAGAG--ATTTTTTTCC---
ATGATTGCATTTTACTGTACAGATTGCTGCTTCTGCTATATTTGTGATATAGGAATTAAGAGGAT????????
??????????????????????????TAATCTCACTTCCGGAGAGCAGTAAGCCACTTCCAGCCCTGCTGTGA
ACCAGGACCGTAGGAGCCATCTTTTTCATGTGGTCAGCTTTGGAAGAATCTACATCCACCACTTTAGAAGACA
AGTCTAGTGGCTTATCTGTGACGTCTTTGGTAACAGTCTGCTTCTCCAACAGAGGAGGTGAGCTGCCGTCTTT
TCTGTCTTGAACCGCTGTCTTCCGGGAATGCCCGGCACTGGCTGGGCACCTTCGCCCCCTTCTGGAGCCTTG

GGATACTTGGCATTGGAGAGCCTGGCCGCGGGGAACTCGCTGCTGACTGTCATGTATGGCTTTGACAGGGCAA
CTGAAGGAGAGGTGGAGATCCTGGCATAGTGCTTGTGGAACTCTGAGTAGGTGTCTGCAGCAGGCTGGGTGGG
AAGGTGAACCCGGGGTGAGGGCCGAGGTGAGGGGGGCAACAGGAGAGCCGTGTCCCCAGGCAGGCCACTGGTG
ACTGCCTTGGCAGAGGGAACCCTGGGCTGTTTACTGTTCTGGATGTGAGGATAGGCATGGGAATCAACAGGAT
TCCCAGGGCTGACCCCCATCTTCCATGGGAGGCTTTTGTCTGCGCAATGGACAAGAGGTGGGATGGCTGGGGA
GGCCGAAGGCGTCGAGAGCCTCATCGGTGATGCCAAGGACGATGGGATGTGGGGACTGACGTAGTGAGGTGGT
GGCAGGTAGAGAAAGCGCTCCCCGTTGGTGCAGACTGGAGAATACAGCGGCTGGGCCAAGCTGTAGGACTGCT
GAGGTAGCAAGGTGCCAGGACCTTTGTAATTCAAGTGCTTTTTACAGTAGTAGCAAATGTTACATAAGCCCAA
ACATGGAGCA---
TATTTGGAGTTGGTGGGCAGACCCAAATCATGATGCAAGCCTGCAGTAGCAAGTGTGCAATGAAGCACAACCA
AATTCTGTGAATGGAAGGTAGCGAGACGCCTCTCCAAAGCAGCCCTTCTGCGCAGGAGCTGAAAGCTCCACCG
GGTAAGTTTTTTGAGGCAGTTTTCAGAGGCTGGGCTTGGCTCAGGGCCCAGCTCTGGATGAGCCTGTGGACGG
TGGGGT----
AGGTCTTTGCTAACCTTACATAGAACCAAGGAGAGTTTGGAAGAGTTTGTGCAGGCCTCTTTCCTCCTGGTGT
TTGTCTTGCCCTGCCAGCAGGCCAGGAGGG-
CTGCGCTGCTCAAGTAGTGGAGGGAGGGAGCCTGCAGAGCTAGGGGATCAGAGTGGGTAATTGCAGCCACAGG
GATTAGCTGCTGCAAGCTGTCCACCGGAGCCATTCTGAAATGTCATTTGAGTGATCTGGCCGTCTACTGGAGG
AGGCCCTCGGGGCAGCTGTCTCCAGGAGCAGCTTCTGAGGGAGTCTTCCAGGAGGAGCGGTGTCTCTGCCATC
GTCCTCGTCCACGTCCTGGTCACATTTCCATTCCTCATCTTCATTCAAAGTTGGCAGGTATCCAGAAATGCCC
TTCCCTGTCCCTGACCCAGAGCTCTGGTCACTACAGACCTTCGGGCTCTCCGAGCCTGTCCTCGTATATTCCA
AATAAATATCAGACTTAAGGAAGGAGGGGTAGGTGTTTTCCTCCATGGTGGCCTGGACTTCTGTCTGGGCCTG
GTCAAACATGGCAGGATCGATCAGCTGCTTCATGATGCAGCCCTTGATGAAGCTCTTGGTGGCCGGCTTGGTC
TGCCGGGACACGATGCCATTGTTATCAAGAATGTACTTTCGGTAGATGGCTCTGGCCAGCTTCAGCCTCTTCT
CCTCGTTCGAGTCACAGGGCTCCAGCTTCCTGAAGCCAGTGCAGGCAAACCAGAAGTCCAGCAAGTCAGCACA
GCCCTCCTGCTTCAGGAAAGTCCTGAACAGGCTTATCCCATCTTGATCATCCAGCAGGGAGTGCAGTGACTCA
GCCCACTTCAAGTACGGTGGGGTGGGGGAGGCACTGCCCTCAGGCTCATACCCCAGGTCCAGATCCGAGCGCC
TCGGAGTGGCGGTCGAAGTCTCACCTTTAATGCCAACACCTTTCCCGGAGCAGAAACTGTAGCTGGCGGGTCT
CAGGTCTGTGGACACCAGTTCTCCCTCCTCACCAGGCACTGGGGGTCGGGGAGCATCTTCGGTGAAACTTGCT
CCAAGGTCCAAGGGGAAACCCTGCTCCTGGACATTCATTTGGGACTCTGTGCGTCAATGCACAACGAGCGCT
GCACCCTAATACATCAGTACTTAACAGCTCCAAAGTCAATCAATCTGTCCTGTTGAAACCATCATTTCTAAAA
TGTTAGCTGATTTTAAGTGAACAA-A--
ATTACATGGATCGACTGATGGCAGCATGAAACAGGCATAATTTACAGTGCAGCAGGATAAAT-
ACTTAGGTTATTTATAGGACAGATTATTTTTCTGTGTTGTTTTG----CATCTTTG-
CATATTAAAATTTCTTTATAGGTGTAGTTAAGCTTT--ACTATTAG----CATAAATTTGGCTTGGAGTCA--
--
CCAAAGCAGAAATGTTGGACTTCATTTTTATGAGTTATTTCCTACACATTTCTAAACTGATGTAGCTTTGTCA
CTGCTTGATCGATTTTTTTT---
AAACTGGAGGAGGTCAAGTTCGTTTATACTGAATCATACCAGTTTCTCTTAATTAGTA---------------
----CATTACTAACATTGAAAAGAAGTAAAAGGAATGTTAATAACTTAGGAATATTTTCATAGTTA-
TGTTCAGAACACAAGCTTTAAATTAGT-GAGGAAACCA---------
GAAGTTTGATTTAAGCACTCATACTCCTTTATTTTCCTTTGTTAGCAATAATTATTGGCCCTGATGGCCATCC
TTTGACTGTTTATCCTTGTATGATTTGTGGGAAGAAGTTTAAGTCAAGAGGTTTTTTGAAAAGACACATGAAA
AACCATCCCGAACATCTTGCCAAGAAGAAGTACCACTGTACTGACTGTGATTACACTACCAACAAGAAGATAA
GTTTACACAACCACCTGGAAAGCCACAAACTGACCAGCAAGGCAGAGAAAGCCATTGAATGTGATGAGTGTGG
GAAGCATTTTTCTCATGCAGGGGCTTTGTTTACTCACAAAATGGTGCACAAAGAAAAGGCAGCCAACAAAATG
CACAAGTGTAAATTCTGTGAATACGAGACAGCCGAACAAGGG???????????????????????????????
???????????????????CCCGCTATAGCTTCTGCAAACAGTAAAGATACCACTAAAACCCCTTCTGCAGT
TCAAACTAGCACCAGCCACAACCCTGGACTTCCTCCTAATTTTAACGAATGGTACGTCTGAGGAAAAACACAA
ACCCTGTTAACCATAGAATGGACCAAACGCATTTTTAAAAGAAAACTGAGACCAATCAGATGGAAATGGAGTT
TTAAGGCAAGAGGCCATATATAGGGCTACATCTTGTTAATTGCAATTGTCCAGGAAGGTTTTGGGCAAGATCC
AAAAGTAGCCATGCCCTTTTCTCAGGATTAGAAAATATGTTTTGGCGTTTGAAGGATTTTTT--
ACAAAATCTTTACACTGCTTTTTCTTACCCTTCCTCTTGCTCTCTGCACACCCAATTCCTAAACTCCTGCATT
TCAATTTAACACTTGTTCTGTTTCTTGAGAGGAAGTTATAGAAGGCTTGTTGGTGGTGGTGATGTTAAACTGA

TGGAAATTCTTTTTCGCCTTAGTGGTGATTGTTTAAACTCTCACAGTCTTAAACCGTGCCAAAGTCCTGTTAT
A
Saimiri
?TACCCCCATGTTTGGCTGGAACATGAAGCTGACCTCAGAGTACTACAGAAATGTCACCCTCCTTTCATGCCG
GTTCCCTT-
CCGTCATGAGCATGGACTACATGGTATACTTCAGCTTCTTCACCTGGATTTTCATTCCCCTGGTCATCATGTG
TGC--AATCTATCTTGACATCTTTTACAT-
CATCCGGAACAAACTCAGACAGAACTTCCCTAACTCCAAAGAGCCGGGCGCATT---
CTATGGACGGGAGTTCAAGACGGCGAAGTCCCTGTTTCTGGTGCTTTTCTTGTTTGCTTTGTCATGGCTGCCT
TTGTCCATCATCAACTGCATCACCTACTTTCA--------------
TGGTGAGGTACCACAGCTTGTGCTGTACTTGGGCATCCTGCTGTCCCATGCCAACTCCATGATGAACCCTATC
????????????????????ATGTTTATAAGCCGCTGACTATTCTCAACTAATCACAAAGACATTGGAACGT
TATATTTATTATTTGGTGCATGAGCTGGGGCAGTAGGGACTGCCTTGAGCCTCCTGATTCGTGCAGAGCTGGG
TCAACCAGGGAGTCTCATAGAAGATGATCACATTTTCAACGTTATTGTCACCGCCCATGCATTCATTATAATT
TTCTTCATAGT---AATACCCATCATAATTGGAGGTTTTGG---
AAACTGACTCATCCCGCTAATAATTGGTGCCCCCGACATAGCATTTCCTCGAATAAATAACATAAGTTTCTGA
CTCTTACCCCCA--TCACTCCTTCTCTTACTTG-
CATCCTCAACTCTAGAAGCTGGCGCAGGGACTGGGTGAACTGTTTATCCTCCTCTAGCAGGAAATATATCACA
CCCAGGGCCCTCCGTGGATCTCACTATCTTTTCACTCCACCTGGCCGGTATTTCCTCTATTCTAGGGGCAATT
AATTTTATTACAACAATTATTAATATAAAACCACCAGCGATGAGTCAATATCAGACACCCCTATTTGTCTGAT
CTGTGTTCATTACAGCAGTCCTCCTACTCCTCTCACTCCCAGTCCTAGCTGCCGGAATTACAATACTCCTAAC
TGATC--
GCAATCTTAACACCTCCTTCTTCGACCCAGCTGGGGGAGGCGACCCTATTCTTTACCAACATTTATTCTGATT
TTTTGGACACCCTGAAGTATACATCCTCATCCTTCCTGGCTTTGGCATGATCTCCCACATTGTTACATACTAC
TCCAACAAAAAAGAACCATTCGGATATATAGGGATGGTATGAGCTATAATATCTATCGGCTTTTTAGGCTTCA
TCGTATGGGCTCACCACATATT-
CACAGTAGGAATAGATGTGGACACCCGAGCATATTTCACATCAGCCACTATAATCATCGCCATTCCCACCGGA
GTAAAAGTATTTAGCTGACTAGCTACACTGCACGGAGGAAATATCAAATGATCCGCCGCTATACTATGAGCTC
TCGGATTTATCTTTCTCTTCACTGTAGGCGGGCTAACAGGAATCGTCTTAGCTAACTCATCATTAGATAT-
CGTCTTACATGATACGTACTATGTGGTAGCTCACTTCCACTACGTCCTATCAATGGGAGCAG-
TATTTGCTATTATGGGGGGCTTTATTCACTGGTTCCCATTATTCTCGGGCTACACACTTGACCAAACCTATGC
TAAAACTCATTT-
TACCATTATATTCGTAGGCGTTAACATAACTTTCTTCCCACAACACTTTCTCGGTCTATCAGGAATGCCCCGA
CGATACTCAGACTATCCCGATGCATACACTACATGAAACATTATCTCATCTGTGGGCTCATTCATCTCATTAG
TAGCAGTAATTCTAATAATTTTTATAATTTGAGAAGCCTTCTCCTCAAAGCGAAAAGTTCTAGTTATTGAACA
AACATCTACCAATCTAGAATGACTCTACGGCTGCCCTCCCCCTTACCACACATTTGAGGAGTCTACCTATGTA
AAACTTTAG???????????????????????????ATGGCAACACCAGCCCAGCTAGGCCTACAAAACGCTA
CATCCCCTATTATAGAAGAACTTATCGCCTTTCACGACCACGCCCTCATAATTATTTTCCTAATTAGCTCACT
AGTCTTATATACTATATCTCTAATGCTTACCACAAAATTAACCCACACTAGCACTATAAATGCTCAAGAGATT
GAGATAATCTGAACCATCTTACCTGCAATTATCCTTATCATAATCGCTCTCCCATCCCTACGTATCTTATACA
TAACTGATGAGTTTATTAAGCCCTACCTGACCCTAAAAGCAATTGGCCACCAATGATATTGAAGCTATGAATA
CTCAGACTACGAAGACCTAGCCTTCGACTCCTACATTATACCAACATACTTTCTAGAACCAGGCGAATTTCGA
CTTCTTGAAGTAGATAACCGAACAACCCTCCCAATAGAAGCAGACATCCGCATATTAGTCTCATCACACGACG
TCTTACACTCATGAGCTGTCCCCTCACTGGGCGTTAAAACAGATGCAATCCCAGGACGCCTAAATCAAATTAT
ACTAGCCTCTATACGACCAGGACTATTCTATGGACAATGCTCAGAAATCTGCGGATCAAACCATAGCTTTATG
CCTATCGTCTTAGAATTTATTTACTTCCAAGATTTCGAAGTATGAGCCTCATA????????????????????
ATGACCTCACCCCGCAAAACACACCCTTTAACAAAAATCATTAATAACTCCTTTATCGACCTTCCTACACCAT
CCAACATCTCTTTCTGATGGAACTTAGGCTCCCTCTTAGGAGCCTGCCTAATTATTCAAATCGTCACAGGCCT
ATTCTTAGCCATACATTACACCCCAGACACCCAGACAGCCTTCTCCTCAGTAGCCCACATCACCCGAGACGTA
AACCACGGATGGATAATCCGCTACATGCATACCAACGGTGCCTCCATATTCTTCATATGCCTCTTCCTCCACA
TTGGACGAGGCCTCTACTACGGATCCTTTCTTTCTCGAGAAACTTGAAACATCGGTACAATTCTACTCCTCAC
AACAATAGCCACAGCATTCATGGGCTACGTTCTCCCATGAGGCCAAATATCATTATGAGGGGCTACAGTGATC
ACAAACCTCCTATCAGCCATTCCATACGTCGGATCCAGCCTCGTAGAATGAGTTTGAGGCGGCTTCTCAGTAG
ACAAAGCCACCCTCGCACGATTTTTTACCTTCCACTTTGTCTTACCCTTTACCATCGCAGCACTAGCGACTAT

TCATCTATTGTTCTTACATGAAACAGGCTCAAACAACCCGTCAGGAGTAACGTCTAACCCTGACAAAATTATA
TTTCATCCATACTATACAATTAAAGATATTCTAGGACTAACCCTTCTACTCCTACTTTTAATAAGCCTAACAC
TATTTATACCAGACCTTTTAACCGACCCAGATAATTACACACTAGCAAACCCCCTCAGCACCCCTCCCCATAT
TAAACCAGAATGATACTTCCTATTCGCATACGCAATCCTGCGATCTATCCCTAATAAGCTCGGGGGCGTACTA
GCCCTTGTACTCTCCATCCTAGTCCTTATAGCCATTCCAACCACACACCTGTCAAACCAACAAAGCATGACAT
TTCGACCAATTACCCAAATCATATTCTGAGTATTAACAGCTAACCTACTTACACTTACATGAATCGGGGGCCA
ACCAGTTGAATACCCATTTACTATCATTGGTCAAATTGCATCCATCATATATTTTCTTATTATCACTGCCCTA
ATTCCCCTCTCAGCCCTAATCGAAAACAAACTACTCAAATGGTAA?TTAGGTTAAATTTATTCAAGGGAATGA
GAATTTATGGGTATAACTGTTAGCTGGAAAGCCCTTCCTCACCTTCAATCCTCCTTTCAAACTACATTTCC-
TCCTGAAATTTCTCTGTGGGATGAAATGTTCAATTATTAGTCAATCAATC----
TCTTATACGTTATCTGGCTTTACATAT----
GCCCTTGTTAGTTCTTGGAAAATAATCCAATATCGTGTTGTTTTGGTGTCTTTGTATTGGAAATGGAAGCATT
TTGTGTGAATTACCTCTTTAACTGTGAGGAGTTGAATTGTCTTGGTTCTGCTTACCTAGTAACTGGGAATAAA
--GACATCACAGTCTCTAACCCCACTTTTT-
GGTGGAAAGAAAATCTAAAAGTAGGAACATTTAATGGAACAAAATATCAATAATTAAAGAAACTCCCCTGTGA
AACTGTAATATTATTTT-
AAAATCAACATTGTTGTAAGATAGCGGAAATGTGTGATTTTTCTATTTTGCCTTTTCACTGACAATATACCCA
TAAATCTTTGGTGGTGATTTTCTCTCCAAAGTTAATTTCTGGGCCATTGGTGACTGCACTATCTATTGTTCTA
AACTGTTGTCTTAATTGCATTCTTTAAAAATGTAATGTTGAAGGCACATAATATTGAAGATGACTCCAGGATG
AACCCTGAGTTCGCAGACCGAA-
TAAAACAGCTCGATAAAGAGGCGTCTTACTACCGTGATGAGTGTGGCAAGGCCCAGGCGGAAGTGGACCGGTT
GCTGGAGA?????????GGAAGAGCTGCATCAGGCAGGTACTTTGATCTTTCTTGGAGCAGGTTCCTTCAAAA
CGCTTCTCCAGAGGACGGAAGTCCCTCCAGTCTGGCTCACTGCTCGCAGTGGTGGGGAAAGCTGAGGTAATTC
CCCGGTGGGAAATCTGCGAGGCCCCTGATACCCCTG--------
CCACCAGGCCCATCACTGATGGGCCTAACGGCCTCATCTGATACTCTAAGATGGGCTTCTCTTGTCGGATCTG
GGCCTCTCGGACTTGAGCCTCTCTAGAACGAGCCTCTCGGACCTGGGCTTCTCGGGTTCTGGCCTCCCTGGCA
TGACCTTCCCAAGTGTGGGCCTCCCTG--
GCATAGGCTTCCTTGCCATGAACTTCTCGAGTATAGGCCTCCCTGGTGTGGGCCTCCCTAGCCTGAGCTTCTC
GGGCACGTGCTTCCCGGGCCTCAAGCTGCTCCCGACGAAGCTCCCAATACAACAACTGTTTCTGGATGGTCA-
CTAGCCGTTCCTCCTCTGTCTCCATTGCCCCAGGTGGCCGGGAGGAAGAAAAGGGCTCAAAGTTCAAGAAGGG
GTCAAACATCTCAGAGCTTCGACCATGGAGGTCATAAAGGCAGTCATCCCCAGGTGGAGAGTTCTCAAGACTG
TCATCTGGCTCATAAAACTCATATAAGGCATCTCCACTGTAGCTGTCTCGGGGCAAACAATCCCTGCGGACAA
GCCCCAGGGTCTCACCTGAATCATCCTCAA????????????ACTAATTTTGATGAACTAAAAATGGTTCCTG
AAGGTCAGATTCATGTACAGTGAATGTTCTCCACTGTTGTGGTGGGCATGTTTCATGTGTGGGGATGTTGGTG
TGCAAGTTTCGTGTGTTTGGAAAACACTAGCTAGAACATTCGAAAAATTTCATTAACCCAGCTCTGGAAGGTC
ATCTATGAATTCTTCCTATTCCAGTGGCCTTTT-
AAGTGTAGATGTAGATACAGTGTAATTTAAAACAGATGGGCATGTCTGAATATTTTCATATGCAAAATGGGAC
AGATGTATATGAATGGATAAATTATCCTTTTAAAAGGATACTTTGTAGCACGAAATATCACCAATGATTGGAA
CAGTGAACCTGCATTCAGTCTAGGGCAAACACCAGGAAGACATTATCAAAACATCTAA-
CTATCTACAAACTAATTCCAATTAGTCTACTTTGTCTTCATACAGAAAAAA-CTTTTTTGATTTTTAAA---
TATGAATTGGGTGCCAATCTGTATCCTTT-
GAGCCGCTAAAGCCAGCATATCACAATAGAAGTTTACTTTTCCTTGAGA????????GTGTTCATAATTAACC
TACTTCTACTAATTGTCCCCACCCTAATTGCCATGGCCTTCTTGACACTTACAGAACGAAAAATTTTAGGCTA
CATACAACTTCGCAAAGGCCCTAATACCATCGGCCCATATGGAATACTCCAACCAATTGCTGATGCAATAAAA
CTTTTCACCAAAGAGCCCCTACTTCCCACAACATCCACCACAACTCTATACGTAATTGCCCCTATTCTAGCCC
TTTCAGTCGCCCTTATCCTATGAACCCCGCTACCTATACCACACCCCCTCGTCAACTTCAACCTAGGCCTTTT
ATTTGTACTTGCAACATCAAGCCTAGCCGTCTATTCAATCCTATGGTCTGGGTGGGCATCCAATTCAAACTAT
GCACTAATCGGTGCACTACGAGCCGTAGCCCAAACAATCTCCTACGAAGTCACTCTAGCTATTATCCTACTAT
CAACCCTACTAATAAGCGGTTCATTCAATATCCACTCATTAATTACAACACAAGAACATTCCTGACTCTTACT
TCCAGCATGACCCCTTACTACAATATGATTTATCTCCACACTAGCAGAAACTAACCGAGCCCCCTTCGACCTA
ACAGAGGGCGAATCAGAACTAGTCTCAGGATTTAATATCGAATATGCTGCCGGCTCTTTCGCCTTATTCTTCA
TAGCAGAATATATAAATATCATTATAATAAATGCCCTAACTACTACCATCTTCATAGCAACCCCCCACAATAC
AGCCCTTCCAGAATCCTACACAGCAAACTTTATAATCAAAACTCTCCTATTAACTGCCTTATTCCTATGAGTC
CGTACCGCATACCCACGACTTCGCTACGACCAACTAATACATCTTCTATGAAAAAAGTTCCTACCACTTACAT

TAGCCCTATGTATATGATATATTTCACTACCTACCCTAACATCCAGCATCCCACCCCAAACAT??ATCAATCC
CATCGCTCACCTCTTCATTTCCCTTACAATCCTAATAGGGACAATAATTGCAATATTCAGCTCCCACTGATTC
CTAATTTGAATAGGCCTAGAACTAAATATACTAGCTATCGTACCAACACTAGCCAAGAGTACAAATCCCCGAT
CCACAGAAGCATCCACCAAATATTTTCTAGTACAAGCAACCGCATCCATAATCTTCTTAATAACTGCTCTCCT
CAACTATCTCTCCTCAGGACAATGAACAATCAATCCCTTCCTCCACCAAACCCTATCCACAACAATACTAATT
GCCCTGATAATAAAACTAGGAATAGCTCCCCTCCACTTCTGACTTCCAGAAGTAACTCAAGGCATTCCCCTAA
CCCCCACCATACTAATCCTCACATGACAGAAACTAGCCCCCATGTCAATTATTACTCAAATCTATCCCTCAAT
AAACCTAAACATCCTACTAACAATCTCAATTCTATCAATTATAGTTGGCAGCTGAGGAGGACTCAACCAAACA
CAACTGCGCAAAATCCTGGCCTACTCATCAATTACTCATATAGGGTGAATAATAGCAGTACTATTCCACGACC
CAAACATCACCGTACTAAACCTCCTAATCTACATCCTACTAACAATCCCTACACTCTTAATCTTTAACCTAAA
CTCAAACATAACAACCCTATCACTATCCCATACCTGAAATAAATTCACATGAATAATACCCATGTTCCCAATA
ATGATAATATCCCTAGGAGGCCTACCCCCACTAACAGGTTTTTCCCCTAAATGAGCTATCATACAAGAACTTA
CAAAAAACAGTGGCCCCACCCTCCCCCTCACCATCGCTATACTGACACTAATAAATCTTTACTTCTACATGCG
CCTAACATACTCCATTTCAATGACAATATTTCCTACATCCAACAACACAAAAATCAACTGACAACTAAAACAT
ATAAAACTAACACCACTTATAGCTCCACTTATAATCTCATCTATATTCTTACTTCCAACAATACCACTAATAC
TATTAATAT?????GTTGGCAGCATAAATAATAAAGCATGTTACTTAAGCAATTTTACATTTACTGTATTAGT
AAGTAAATGCAATGGCCACTCTTATTCTCAAACAATAAAAACTACCCAGCCTTTTCATGAAAGAT-
GATTGCAATTTCCATTGGCCTGTATATGCAATAAAAAG---
CCATAACTCATCAAAATTTTAGCTATTTTCTCTTAAGATA--
GAAGAGCGTTTTGCTTGAACATTGAACGTGACATTAAATATT-
CATGAAACTGAATAGAGTTTAAGTACTAAG---
TAAAACTCTCAAATTCTTTTCAACTAGATTGAAATTCTGTTTGAACCAGGTAAAAACTACCGCTAACCTGAAG
AGGTGTTCAAAAAC-
ATCGTAAAGAGATTGTACAATACGTTCAAAGTGCTACAGTTATTTGGGATAGATTTTTCTGAAAAGCCTGTTC
CTCCTTTGAAATTGATTCATGATAACAGCCTCCATTTGGGTCAAACAGTATTTAACCGCTTGTAAA-
TTTTAATT-
AAATGATTTGTCACTTTGAACACATAGTTTGTC?????????????????????????????????????
??
??AGTTTG
AAGAAGCCAAACCAAACTCTGAACTGTGTTGCAAGCCATTGTGCCTTATGCTGGCAGATGAGTCTGACCACGA
GACCCTGACTGCCATCCTGAGTCCTCTCATTGCCGAGAGGGAGGCCATGAAGAGCAGTGAATTAATGCTTGAG
ATGGGAGGCATTCTCCGGACTTTCAAGTTCATCTTCAGGGGCACTGGATATGATGAAAAACTTGTGCGGGAAG
TGGAAGGCCTTGAGGCTTCTGGCTCAGTCTACATCTGTACTCTTTGTGATGCCACCCGTCTAGAAGCCTCTCA
AAATCTTGTCTTCCACTCTATAACCAGAAGCCATGCCGAGAACCTGGAACGTTATGAAGTCTGGCGTTCCAAC
CCTTACCATGAGTCTGTGGAAGAACTGCGGGATCGGGTGAAAGGGGTCTCAGCCAAACCTTTCATTGAGACAG
TCCCTTCCATAGACGCACTCCACTGTGACATTGGCAATGCAGCTGAGTTCTACAAGATCTTCCAGCTAGAGAT
AGGGGAGGTGTATAAGAATCCCAATGCTTCCAAAGACGAAAGGAAAAGGTGGCAGGCCACATTGGACAAGCAT
CTCCGGAAGAAGATGAACCTTAAACCAATCATGAGGATGAATGGCAACTTTGCCAGGAAGCTCATGACCAAAG
AGACTGTGGATGCGGTTTGTGAGTTAATTCCTTCCGAGGAGAGGCATGAGGCTCTGAGGGAGCTGATGGATCT
TTACCTGAAGATGAAACCAGTATGGCGGTCATCATGCCCTGCTAAAGA--GTGCCC-
AGAATCCCTCTGCCAGTACAGTTTCAATTCACAGCGTTTTGCTGAGCTCCTTTCTACCAAGTTCAAGTATAGG
TATGAGGGGAAAATCACCAATTATTTTCACAAAA???????????TATGGCACCCGACAGTTGTCATTATGTT
TGGAAACACTGCCGGCCCATGGAGATGAGGATAAAGTGGACATTTCTCTGCTGCATCAGGGTGAGAATCTTTT
CGAACTGCACATCCACCAGGCCTTCCTGACATCTGCTGCCCTGGCTCAGGCTGGAGACACCCAACCTACCACT
TTCTGCACCTATTCCTTCTATGACTTTGAGACCCATTGTACCCCATTGTCTGTGGGGCCACAGCCCCTCTATG
ACTTCACCTCCCAGTATGTGGTGGAGACAGACTCCCTTTTCTTACACTACCTTCAAGGGGCTTCAGCCCGGCT
TGATCTCCACCAGGCTGTGGCCAGTGAGCACAACACTCTTGCTGCAGGATGGATTTGCTTTGACAGGGTGCTA
GAGACTGTGGAGAAAGTCCATAGCTTGGCCACACTGATTGGTAAGTGCTGTCGGCTTCCTGCAGCTCCTA-
GCACCAATGCAGAATTCCCCAAACTCTATAGCTGCTTTTCTGGTCATTTCTTATTTTCTTCCTTTTTCTTTGT
TCTTGATCATTGCCACGCCATCTGTATTCTCCATGATTTCA-
TACTTGCTGCTGCCCAGGAGCTGGTGGAGAAGAGTTTGGGGTTCTAGAGTACTGGATGAGGCTGCGTTTCCCC
ATAAAACCCAGCCTACAGGCATGCAATAAACGAAAGAA????????????????????????????????
??????????????????????????TTCATGGAGGGAAAACACCAAACAATGAGCTTTCAGATAAGATTTA
TGTCATGTCTGTTGTTTCCAAGAACAA---

GAAGGTTACTTTTCGCTGCATGGAGAAAGACTTGGTAGGAGATGTTCCTGAAGCCAGATATGGTCATTCCATT
AATGTGGTATATAGCCGAGGGAAAAGTATGGGTGTTCTTTTTGGAGGACGCTCATACATGCCTTCTACCCACA
GAACCACAGAAAAATGGAATAGTGTAGCTGACTGCCTGCCCCATGTTTTTCTGGTGGATTTTGAATTCGGGTG
TGCTACATCATACATTCTTCCAGAACTTCAGGATGGGCTATCTTTTCATGTCTCTATTGCCAAAAATGACACC
ATCTATATTTTAGGAGGACATTCACTTGCTAATAATATCCGCCCTGCCAACCTGTACAGAATAAGGGTTGATC
TTCCCCTGGGTAGCCCAGCTGT-
GAATTGCACAGTCTTGCCAGGAGGAATCTCTGTCTCCAGTGCCAA????????????????????????????
???
??TGTGTCTCAGCATCTTCCTGCTC
TCCACCACACTGAGCCCAAGATGACCCAGTGCTCCACAAACATATGTAGAATGGAAGTAAAACCTCAGGGATA
AGAAAAGATAATTAAAGGCCGGGCGCAGTGGTTCATGCCTGTAATTCCAGCACT-
TAGGAGGCCAAGGAGAGTGCATCACAAAGTCAGGAGTTCAAGACCAGCCTGGCCAAGATGGCAAAAACCCATC
TCTACTAAAAATA---CAACAATT-----
AGCTGGTGTATTTTTAC--
---------------------TGTGGTGGTGGGCACCTGTAATCCTAGTTAATTGGGAGACTGAAGCAG-
AGAATTGCTTGAACCTGGGAGGCAGAGGTTGCAGTGAGCCAAGATCATGCCACTGCACTCCAGCCTGGACAAC
A-GAGTGAGACTCCATCTCAA--

---AAAA-
TAATAATAAAAAGAAAAGATAGTTAAAGCAGATTCACTATAGTAACAAACCAGCCAAGAAACTGCACTGAAAG
TAAAGGTTTTGGGTGACAAAATTTCCAACTGTGTTCAAAGAAACGTAAGTTCCAGTAGGAGTGCACTTAATCT
GATTTT-CTTCAAAGC--
CTCTCTAATTTGTAGGGAAGGACATCCATTTTCACTAGCTTCATGTGAGCCAAAGAGAACCCACGACTAAAGT
GCTAGAACCCCTGGTAAACATGCCAGCAAGGAAGGGCACCTTCCCCAGAGGCTCAACCTAGAATATAAGAC--
--AGCATTGGTGAGGAGCCTTGAACTCTAAACCCACAGGCTCAAGTCAGGAA-
CTCAGGAAGCCTATCAACACCGGGATACAATTTTGCCACCTTAACACTTTATTCCCTTTACTTGTGCCACACT
???
???
???
???
???
??????????????????????????GGAACTCAAGGCCCTCAAAGATCTTTATTGCCATAAAGCAGAGTAAC
T-GTCTTTGACTTGGACCTTGTTTACTGTGAACTCTAATC-AAGGCAGGACATGCAGCAGTTCTAATTA-
CTGCACTGTGGACGTGTGGAAAGGACACTTGTGA---------
ATCCAGTTTGGATTAGTGTTTGAAATTGAATTGCAAATGTTGTTCCCCGATGTGGAATGCAGCCGTGATCACC
CTTGCCTACC-GAGCTTACTTTGATCTGTTTGTCAGTAGCATGCAAAAAA-
TGCTTTGTTTGCCCTTTGCTTCTGCTTTTTTCAGGGAAGCTGCCAAAGAATGTCGACGCCGAAAGAAAGAATA
CGTAAAATGTCTGGAGAGCCGAGTCGCAGTGCTGGAAGTCCAGAACAAGAAGCTTATAGAGGAACTTGAAACC
TTGAAAGACATTT??
???
???
???
???
???
???
???
???
??TGTTTATATCTAAATGTGTGGATTCCA
GCACCTAAACCAAAAAATGCCACTGTAATGATATGG-
ATTTATGGTGGTGGTTTTCAAACTGGAACATCATCCTTACATGTTTATGATGGCAAGTTTCTTGCTCGGGTCG
AAAGAGTTATTGTAGTGTCAATGAACTATAGGGTGGGTGCTCTAGGATTCTTAGCTTTGCCAGGAAATCCTGA
GGCTCCAGGAAACATGGGTTTATTTGATCAACAGTTGGCTCTTCAGTGGGTTCAAAAAAATATAGCAGCCTTT
GGTGGAAATCCTAAAAGTGTAACTCTTTTTGGAGAAAGTGCAGGAGCAGCTTCAGTTAGTCTGCATTTGCTTT
CTCCTGGAAGCCATTCCTTGTTCACCAGAGCCATTCTGCAAAGTGGTTCCTCTAATGCTCCTTGGGCAGTAAC

ATCTCTTTATGAAGCTAGGAACAGAACGTTGGCCTTAGCTAAATTTACTGGTTGCTCTAGAGAAAATGAGACT
GAAATAGTCAAATGCCTTCAAAACAAAGATCCCCAAGAAATTCTTCTGAATGAAGCATTTGTTGTTCCCTACG
GAACTCTCTTGTCAGTAAACTTTGGTCCCACAGTGGATGGTGATTTTCTCATTGACATGCCAGACATATTACT
TRAACTTGGACAATTTAAAAAAACCCAGATTTTGGTGGGTGTTAATAAAGATGAAGGGACAGCTTTTTTAGTC
TATGGGGCTCCTGGCTTCAGCAAAGATAACAATAGTATCATAACTAGAAAAGAATTTCAGGAAGGTTTAAAAA
TATTTTTTCCAGGAGTGAGTGAGTTTGGAAAGGAATCCATCCTTTTTCATTACACAGACTGGGTAGATGATCA
GAGACCTGAAAACTACCGTGAGGCCTTGGACGATGTTGTTGGGGATTACAATATTATATGTCCAGCCTTGGAG
TTTACGAAGAAGTTCTCAGAATGGGGAAACGACGCCTTTTTCTACTATTTTGAACACCGTTGTGCTAATTGTG
AGAAAATATGCTACTTTGATCAAACTAGAATGAAGGAAATAAAAAGACACTGTGGCTCAACTTGTAATAAAGA
GCAGCTCATCCTCACAGCTTTTT-ATTGTTGCTATAGTTATCT---
CTTCAACGTGGGGTGCACAATACAGTTAACGTTTCATTACCAGTAGCCTGTTACGTGCTTATTGTTAAAAGGA
ATTTAACATACAAGTTTTTGTATGAATTGTTCGCCTAATTCCGATTCCTCGTGCTGACTGGCATGAGACAGGG
AGCTGGATTGTATTAAACTGGTTTTATTGTGATGTGACTATGATGGATGTGACAAGGGCCTCTATTATTCGCT
GGGGAGTGTGGCCGTTAATGAAGCCATTAGCTGAGCTAAGGCAAGTGAAGAAAATAGGGGAATTACCATTATT
CTGAAGCCATTAAGTTGTAATCCACCACTTTCTCTCCTTATTCAAAAAGGCATCTTCTTTTTAAGGCCTCCCT
???AACGTCCGAGGAC
AAGGTGGCTTGGCCTACCCAGGTGTGCGGACCCACGGGACTCTGGAGAGCGTGAATGGGCCCAAGGCAGGTTC
AAGAGGCCTGACGTCGTCGTTGGCCGACACTTTCGAACATGTGATAGAAGAGCTGTTGGATGAGGACCAGAAA
GTTCGGCCCCATGAAGAAAACAATAAGGACGCAGACTTGTACACGTCCAGGGTGATGCTCAGTAGTCAAGTGC
CTTTGGAGCCTCCTCTTCTCTTTCTGCTGGAGGAATACAAAAATTACCTGGATGCTGCAAACATGTCCATGAG
GGTCCGGCGCCACTCTGACCCTGCCCGCCGAGGGGAGCTGAGTGTGTGTGACAGTATTAGTGAGTGGGTAACG
GCGGCAGACAAAAAGACTGCAGTGGACATGTCGGGCGGGACGGTCACAGTCCTTGAAAAGGTCCCTGTATCGA
AAGGCCAACTGAAGCAATACTTCTACGAGACCAAGTGCAA?????????????????????????????????
???TGAGCAGATGCA
GAACTAGACCCCC-
GCCACGGCAGCCTCTGAAGTTGGACAGCAAAACCATTGCTTCACTACCCATCGGTGTCCATTTATAGAATAAT
GTGGAAAGAAACAAACCCCTCTGTCTTATGATTTCCTCCTTACCGCCTTTTGACAGCTGTGCTGTAACACAAG
TAGATGCCTGAACTTGAATTAATCCACAGATCAGTAATGTATTCTCTCTGTCTCT------TT----------
--------ACATTTTGGTCTCTACACTACATTATTAATGGGTTTTGTGTACTGTAAAGAATTTAGCTGTTTC-
AAACCTAGTGCATGA-
ATAGATTCTCTCCTGATTATTTATCACATAGCCCCTTAGCCAGTTGTATATTATTCTTGTGGTTTGTGACCCA
ACTAAGTCCTACTTTAAATATGCTTTAA-GAATCGATGGGGGA---
TGCTTCACGTGAACGTGGGAGTTTAGCTGCTTCTCTTGCCTAAGTATTCCTTTCCT-
GATCACTATGCATTTTAAAGTTAAA-
CATTTTTAAGTATTTCAGATGCTTTAGAGAGATTTTTTTTTCCATGATTGCATTTTACTGTACAGATTGCTG
CTTCTGCTATATTTGTGATATAGGAATTAAGAGG???????????????????????????????????????
?????????????????????????????????CCCTGCTGTGAACCAGGACCGTAGAAGCCATCTTTTTCAT
ATGGTCAGCTTTGGAAGCATCTATATCCACCACTTTAGAAGACAAGTCTAGTGGCTTATCTGTGACATCTTTG
GTAACGGTCTGCTTCTCCAACAGAGGAGGTGAGCTGCCGTCTTTTCTGTCTTGAACCGCTGTCTTCCGGGAAT
GCCCGGGCACTGGCTGGGCACTTTCGCCCCCTTCCGGAGCCTTGGGATACTTGCCGTTGGAGAGCCTGGCCGC
GGGGAACTCACTGCTGACTGTCATGTATGGCTTTGACAGGGCAACTGAAGGAGAGGTGGAGATCCTGGCATAG
TGCTTGTGGAACTCCGAGTAGGTGTCTGCAGCAGGCTGGGTGGGAAGGTGGACCCGGGGTGAGGGCCGAGGCG
AGGGGGGCAACAGAAGAGCCGTGTCCCCCGGCAGGCCACTGGTGACTGCCTTGGCAGAGGGAACCCTGGGCTG
TTTACTGTTCTGGATATGAGGATAGGCATGGGAATCAACAGGATTCCCAGGGCTGACCCCCATCTTCCATGGG
AGGCTTTTGTCTGCGCAATGGACAAGAGGTGGGATGGCTGGGGAGGCCGAAGGCGTCGAGAGCCTCATGGGTG
ATGCCAAGGACGATGGGATGTGGGGACTGACGTAGTGAGGTGGTGGCAGGTAGAGAAAGCGCTCCCCGTTGGT
GCAGACTGGAGAATACAGCGG????????????????????????????????????TGCCAGGACCTTTGTAA
TTCAAGTGCTTTTTACAGTAGTAGCAAATGTTACATAAGCCCAAACATGGAGCA---
TATTTGGAGTTGGTGGGCAGATCCAACTCATGATGCAAGCCTGCAGTAGCAAGTGTGCAATGAAGCACAACCA
AATTCTGTGAATGGAAGGAAGCGAGACGCCTCTCCAAAGCAGCCCTTCTGCGCAGGAGCTGAAAGCTCTGCCG
GGTAAGTTTTCTGAGGCAGTTTTCAGAGGCTGGGCTTGGCTCAGGGCCCAGCTCTGGATGAGCCTGTGGGCGG
TGGGGT----
AGGTCTTTCCTAACCTTACATAGAACCAAGGAGAGTTTGGAAGAGTTCGTGCAGGCCTCTTTCCTCCTGGTGT
TTGTCTTGCCCTGCCAGCAGGCCAGGAGGG-

CTACGCTGCTCAAGTAGTGGAGGGAGGGAGCCTGCAGAGCTAGGGGATCAGAGTGGGTAATTGCAGCCACAGG
GATTAGCTGCTGCAAGCTGTCCACAGGAGCCATTCTGAAATGTCATTTGAGTGATCTGGCCGTCTACCGGAGG
AGGCCCTCGGGGCAGCTGTCTCCAGGAGCAGCTTCTGAGGGAGTCTTCCAGGAGGAGCAGTGTCTCCGCCATC
GTCCTCGTCCACATCCTGGTCACATTTCCATTCCTCATCTTCGTTTAAAGTCGGCAGGTATCCAGAAATGCCC
TTCCCTGTCCCTGACCCAGAGCTCTGGTCACTACAGACCTTTGGGCTCTCTGAGCCTGTCCTCGTATATTCCA
AATAAATATCAGACTTAAGGAAGGAGGGGTAGGTGTTTTCCTCCATAGTGGCCTGGATTTCTGTCTGGGCCTG
GTCAAACATGGCAGGATCGATCAGCTGCTTCATGATGCAGCCCTTTATGAAGCTCTTGGTGGCCGGCTTGGTC
TGCCGGGACACGATGCCATTGTTATCAAGGATGTACTTTCGGTAGATGGCTCTGGCCAGCTTCAGCCTCTTYT
CCTCGTTCGAGTCACAGGGCTCCAGCTTCCTGAAGCCAGTGCAGGCAAACCAGAAGTCCAGCAAGTCGGCACA
GCCCTCCTGCTTCAGGAAAGTCCTGAACAGGCTTATCCCATCTTGATCATCCAGCAGGGAGTGCAGTGACTCA
GCCCACTTCAAGTACGGTGGGGTGGGGGAGGCACTGCCCTCAGGCTCATACCCCAGGTCCAGATCCGAACGCC
TGGGAGTGGCGGTCGAAGTCTCACCTTTAATGCCAACACCTTTCCCGGAGCAGAAACTGTAGCTGGTGGGCCT
TGGGTCTGTGGACACCAGTTCTCCCTCCTCACCAGGCACTGGGGGTCGGGGAGCATCTTCGGTGAAACTTGCT
CCAAGGTCCAAGGGGAAACCCTGCTCCTGGACATTCATTTTGGGACTCTGTGCGTCAATGCACAATGAGCGCT
GCACCCTAATACATCAGTACTTAACAGCTCCAAAGTCAATCAATCTGTCCTGTTGAAACCAT???????????
???
???
???
???
???
???
???
???
???
???
???
???
???
?????????????TACCCGCTATAGCTTCTGCAAACAGTAAAGATACCACTAAAACCCCTTCTGCAGTTCAAA
CTAGCACCAGCCACAACCCTGGACTTCCTCCTAATTTTAACGAATGGTACGTCTGAGGACAAATACAAACCCT
GTTAACTATAGAATGGACCAAATGCATTTTTAAAAGAAAACTGAGACCAATCAGATGGAAATGGAGTTTTAAG
GCAAGAGGCCATATATAGGGCTACATCTTGTTAATTGCAATTGTCCAGGAAGGTTTTGGGCAAGATCCAAAAG
TAGCCATGCCCTTTTCTCAGGATTAGAAAATATGTTTTGGCGTTTGAAGGATTTTTT--
ACAAAATCTTAACACTGCTTTTTCTTCCCCTTCCTCTTGCTCTCTGCACACCCCATTCCTAAACTCCTGCAAT
TCATTTTAACACTTATCCTGTTTCTTGAGAGGAAGTTATAGAAGGCTTGTTGGTGGTGGTGATGTTAAACTGA
TGGAAATTCTTTTTCGCCTTAGTGGTGATTGTTTAAACTCTCACAGTCTTAAACCGTGCCAAAGTCCTGTTAT
A
Xenothrix
????????ATGTTTGGCTGGAACATGAAGCTGACCTCAGAGTCCTACAGAAATGTCACCTTCNNNTCATGCCG
GTTCCCTT-
CCGTCATGAGCATGGACAAGATGGTNTACTTCAGCTTCTTCACCTGGATTTGCATTCCCCTGGTCATCATGTN
NNNGCAATCTATCTTGACATCTTTTACATACATCATGAACAAACTCAGTCAGAACTTCTCTAACTACAAAGAG
ACGGGTGCATTTTNNNNNNGACGGGAGTTCAAGACGGCGAAGTCCCTGTTTCTGGTGCTTTTCCTGTTNNNNN
NNNNNNNNNNNNNNNNNNNNNNNNNNNNNNNNNTTATCCATCATCAACTGCATCACCTACTTTCNNNNNNNNN
NNNNNNNNNNNNNNNNNNNNNNGCTGGTGCTGTACCTGGGCATCCTGCTGTCCCATGCCAACTCC???????
????????ATGTTCGCCAACCGATGACTATTCTCAACAAATCACAAAGACATCGGAACACTGTACCTACTATT
TGGAGCATGAGCAGGAGCAGTTGGAACTGCCCTAAGCCTTCTAATTCGAGCAGAACTAGGACAACCTGGAAGT
CTAATAGAAGATGACCATGTTTATAACGTTATCGTTACCTCCCACGCATTTATTATAATCTTCTTAATAGT--
-AATGCCAATTATAATTGGGGGNTTCGG---
AAACTGACTTATCCCAATTATAATTGGTGCTCCTGATATAGCATTTCCTCGTCTAAATAATATAAGCTTCTGA
CTCCTTCCCCCA--TCACTCCTGTTACTGCTTG-
CATCTTCAACCCTAGAGGCCGGCGCAGGAACTGGCTGAACAGTATATCCACCCGTAGCTGAAAACATATCCCA
CCCAGGAGCCTCAGTTGACCTAACTATTTTCTNATTACANNTAGCAGGCATCTCCTCTATCCTAGGGGCCATC
AATTTCATCACAACAATTATCAATATAAAACCCCCTGCCATAACCCAATACCAAACGCCTCTATTCGTCTGAT

CCGTTCTAATCACAGCAGTACTTCTTCTTCTATCGCTCCCAGTTCCAGCTGCTGGAATTACTANNNNNNNNAAC
AGACC--
GTAACCTTAACACTACTTTTTTTGATCCCGCTGGCGGAGGTGACCCAATNNNATANCAACATTTATTTTGATT
CTTCGGTCACCCTGAAGTGTATATTCTTATCCTACCTGGCTTCGGAATAATTTCACATATTGTNNCATATTAT
TCCAATAAAAAAGAACCATTCGNNTACATAGGAATAGTGTNNGCAATATTATCAATTGCTATTTTAGGATTTC
TCGTGTGGGCACATCATATGTT-
TACAGTAGGAATAGATGTCGATACCCGTGCTTATTTTACAGCAGCAAGCATAATTATTGCTATCCCTACTGGA
GTAAAAGTATTTAGCTGGTTAGCCACGCTGCACGGAGGCAACATTNNNNNNNNNNNNCGCAATACTATGAGCCC
TGGGGTTCATTTTTCTCTTTACCGTAGGCGGGCTAACAGGAATCGTACTAGCCAACTCNNNNNNNNNACAT-
TGTCCTACATGGTACATNNTATGTTGTAGCCCATTTTCACTATGTANNNTCCATAGGAGCAG-
TATTTGCTATCATAGAAGACTTTATTCACTGATTCCCACTATTCTCTGGTTACACCCTCGATCANNCATATGC
TAAAATTCATTT-
CAGCATTATATTTGTAGGTGTAAACATAACCTTTTTCCCCNNNNNNNNNNNNNGGCCTATCAGGCATACCTCGA
CGTTACTCAGCCTACCCCGATGCCTACACAACATGAAATATTATCTCATCTGTAGGCTCATTCATCTCACTAA
CAGCAGTAATTTTAATAATTTTTATAATTTGAGAAGCTTTCTCTTCAAAACGGAAAGNNNNAACCATCGAACA
ACTAGCCACCAATCTAGAGTGACTTTACGGCTGCCCCCCTCCCTACCACACATTTGAAGAGGCCACCTACATT
AAAACCCTGAACGAAAAAGGAAGG????????????ATGGCCCACCCAGCCCAACCACGCCTACAAAATGCAG
CTTCCCCCATCATAGAAGAACTTATTGCTTTCCACGATCACGCCCTTATAATTATTTTCCTAATNNNNNNTCT
AGTTCTATATATTATCTCCTTAATACTAACAACAAAACCAACACACACCAGCACAATAAATGCTCAAGAAATT
GAAATAATTTGAACNNNNNNNNNCCGCCATTATTCTTATTATAATTGCCCTTCCATCCNTACGTATCCTCTACA
TAACNNACGAATTCAATAAACCCTATCTTACCCTCAAAGCCATCGGCCACCAATGATATTGAAGTTACGAATA
TACAGACTATGAAGACCTGGCCTTTGACNNNNATATTATGCCCACCTATTTTCTTGAGCCTGGTGAGTTCCGA
CTCCTCGAAGTAGATAATCGCACNNNNNNNNNNNATAGAAGCAGACATTCGTATACTAATNNNNTCACAAGACG
TACTACACTCATGAGCTGTCCCAACATTAGGCTTAAAAGCAGATGCAATTCCTGGACGCTTAAACCAAGCCAT
AGTAGCCTCAATACGACCAGGTCTTTACTATGGACAGTGCTCAGAAATTTGTGGATCTAATCATAGCTTTATA
CCTATTGTCNNNNNNTTTATTTACTTCCAAGACTTCG?????????????????????????????????????
ATGACCATTCCCCGCAAAACACACCCACTAGCAAAAATTATTAACANCTCATTTATTGATCTTCCCACACCAT
CCNNNNTCTCATACTGATGAANNNNNNNNNNNNNNNNNNAGGTATCTGCCTTATNACCCAAATCGCCACCGGCCT
ATTTCTAGCCATANNNNNNNNNNNNNNNNNACTTCAACCGCCTTCTCCTCAGTAGCTCATATTANNCGAGACGTA
AACTACGGCTGNNNNATCCGCTACCTACACGCTAACGGCGCCTCCATATTTTTTATTTGCCTCTTTATGCATG
TTGGACGGGGCTNNNNNTATGGATCTTTTCTCTTTCTGAAGACCTGAAACGTCGGTATTATCCTACTACTGAC
ATCCATAGCAACTGCTTTTATTGGTTATGTACTTCCATGGGGCCAAATGAGTTTGTGGGGCGCCACAGTAATT
ACTAATTTGATATCAGCTATTCCNNNNNNTGGATCAGACCTCGTCCAATGAATCTGAGGAGGATTCTCTGTAG
ATAAAGCCACACTCACACGATTTTTCACCTTCCACTTTATTATACCCTTTATTATTGCANCGTTAGCTGCTAT
TCACCTTTTATTTCTCCANNNNNNNNNNNNNNNNNNNNNNNNTCAGGACTAATATCTAACTCCGACAAAATTGCA
TTCCACCCCTACTATACAATTAAAGACATTCTAGGATTAGTTCTTCTTCTCCTTCTAATAAGCCTAACCC
TATTTACACCTGATCTATTAACTGNCCCAGATAACTACACCCTAGAAAANNNNNNTAACACCCCGCCCCACAT
CAANNNNNAATGATACTTCCTATTCGCATACGCCATCCTACGATCTATCCCCAATAAACTAGGAGGGGTTCTA
GCNCTTATACTCTCCATTCTAATTCTAGCAATTATTCCTATAATCCACCTGTCCAAACAACAAAGTATAATAT
TTCNACCAATCAGCCAAAGTTTATTCTGACTCCTAACAGCTATTCTACTTACACTCACATGAATTGGAGGCCA
ACCAGTTGAACACCCCTTTATTACTATTGGCCAAACAGCATCTATTATATACTTCCTCATTATTATTACCCGT
ATCCCTTTATCCTCTCTAATTGAAAATAAATTACTAAAATTATAA????????????????????????????
?????????????????????????GAAGAGCCCTTCCTCACCTCCAATCCTCCTTTCAAACTACATTTCCGC
TCTGAAATTTCTCTATGGGATGAAATGTTCAATTATTGGTNNNNNNNNNN----
NNNCATATGTTATCTGGCNNNNNNNNNN----NNNNNNGATA-
TTCTTGGAAAATAATNNNNNNNNNNTGTTGTTTTGGTGTCTTTGTATTGGAAATGGAAGCATTTTGTATGAANN
NNNNNNNNNNGTGTGAGGAGTTGGATNNNNNNNGTTCTGCATGCCTAANNNNCAGCAATAAA--
GATATCACANNNNNNNNNNNNNNNNNNN-
NNNNGAAAGAAAATCTAAAAGTAACAANNNNNAAGGGAACGAAGTATCAATAGTAAAAGAAACNNNNNNNNNN
NNNNNNNATACTATTTTTAAAATCAACATTGTTCTANNNNNNNNNNNNNNNNNNNNNTTTTTACTTTTGCCTTT
TCACTGACAATATACCNATAAATCT----
TGGTGATTTTCTCTNNNNNNNNNNNNNNNNNNNNNNNNNNNNNNNNNNNNNNNNNNNNNNNNNNNNNNNNNNNNNN
NAATTCCAATCTTTAAAAATGTAATGTTGAAGGCACATAATATTGAAGANGACTCCAGGATGAACCCNNNNNN
NNNNNNNNGAAGTAAAACAGCTCGATAAAGAGGCGNNNNNNNNNNCGTGATGAGTGTGGCAAGGCCCAAGCGGA

AGTGGACCGGT???TTTCTTGGAGCAG
GTTCCTTNAAAACGCTTCTCCAGAGGACGGAAGTCCCTCCAGTCTGGCTCGCTGCTCGCAGTGGTGGGGAAAG
CNNNNNNNATTCCTCGGTGGGAAATCTGCGAGGCCCCTGATACCCCTG--------
CCACCAGGCCCATCACTGANNNGCCTAAGGGCCTCATCTGGCACTCAAAGACGGGCTTCTCCTGTCGGNNNNN
NNNNNNNNNNNNNNNNNNNNNNNNNNNATGAGCCTCTCGGGCCTGGGCTTCTCGGGTTCTGGCCTCCCTGGCA
TGACCTTCCNNNNTGTGGGCCTCCCNNTAGCATAGGCTTCCTTGCCATGAGCCTCTCGNNNNNNGGCCTCCCT
GGTGTGGGCCTCCCTGGCCTGAGCTTCTCGGGCACGTGCCTCCCGGGCCTCAAGCTGCTCCCGCCGAAGCTCC
CAATACAACACCTGTTTCTGGATGGTCACNTAGCCGTTCCTCCTCTGTCTCCANTGCCCCAGGTGGCCGGGAG
GAAGAAAAGGGCTCAAAGTTCAAGAAGGGGNCAAACATCTCAGAGCTTCGACCATGGAGGTCATAAAGGCAGT
CATCCCCAGGTGGAGAGTTCTCNNNNNTGTCATCTGGCTCATAGAACTCATATAGGGCATCTCCACTGTAGCT
GTCTCGGGGCAGACAATCCCTGCGGACAAGCCCCAGGGCCTCACCTG??????????????????????????
??????????????????????GTTCCTGAAGGTCAGATTCATGTACGGTGNNNNNTCGCCACTGTTGTGGTG
GGCATGNNNNNNNNGTGGGGATGTTGGTGTGCNNNNNNNNNNNNNNNNNNNNNNNNNNNNNNNNNNNNNNNNN
NNNNNNNNNNNNCCCAGCTCTGGAAGGTCATCCATGNNNNNNNNNNNNNNNNNNNNNNNNNN-
NNNNNNNNNNNNNNNNNNNNNNNNNNNNNNNNNNNNTGGCCATGTCTGAATATTTTCATATGCAAAATGGGAC
AGATGTATATGAATGGNNNNNNNNNCCTGTTAAAAAGATACTTTGTAGCACGAAATNNNNNNAATGATTGGAA
CAGTGAACCTGCATTCAGNNNNNNNNNNNNNNNNCAGGAAGACATTATCGAANNNNNNNNNNNNNNNNNNNNN
NNNNNNNNNNNNCTACTTTGTCTTCATACAGAAAAAAACTTTTTGATTTTNTAAATANNNNNNNNNNNNNNNN
NNTCCGTATCCTTT-
GAGCTGCTAAAGCCAGCATANNNNNNNAGAAGTTTACTTTTC?????????????????????ATAATCAATT
TACTTCTACTTATTATCCCAGCCTTAATCGCAATAGCATTCCTAATGCTTACCGAACGAAAAATTCTAGGCTA
TATACAACTACGCAAAGGCCCCAACATTGTTGGCCCCTACGGAACACTCCAACCTATCGCAGACGCANNNNNN
NNNTNNNNNNNAAGAACCCCTACTACCCACAGCATCCACCACAACCCTATATATTATTGCNNNNNNNNNNNNT
TATCTATTGCTCTCCTCCTATGAACTCCCCTCCCTATACCTCTTCCACNNNNNNNNNNNNNNNNNNNNNNNCT
ATTTATCCTAGCAACCTCAAGCCTAGCTGTATACTCAATCTTATGATCCGGATGAGCATCCAACTCAAAATAT
GCATTAATTGGCGCATTACGAGCCGTAGCTCAAACAATCTCATATGAAGTCACCCTNGCCATTATTCTCCTCT
CTGTTCTACTAATAAGCGGCTCATTCAATCTGCACTCACTTATTACAACGCAAGAACACCTATGACTTCTTCT
ACCATCAGGACCACTTGCTATAATATGATTTATCTCTACACTAGCAGAGACCAACCGAACCCCCTTCGACCTA
ACAGAAGGCGAGTCCGAACTAGTCTCAGGCTTCAACATCGAATACGCCGCAGGCTCATTTGCCCTATTCTTCA
TAGCAGAATACATAAACATTATTATAATAAATGCTCTAACCACTACTATCTTCCTAGCAACACCCCATATNNN
NNNNNNNNNNNAACTCTACACAATAANNNNTATAACTAAAACACTTATACTAACTGCACTATTTCTATGAATT
CGATCAGCATACCCCCGATTTCGTTACGATCAACTCATACACCTCCTATGAAAAAAATTTTTACCTCTTACAC
TAGCNNNNNNNNNNNNNNNNATTTCTATACCAATCCTAATATCTGGCATCCCGCCCCAAATATA?ATTAACCC
CCTAGCCCATCTCACTATTTCCTTCACAATCCTAACAGGAANNNNNNNNNNNNNNNNNNGCTCACACTGATTC
CTCATTTGAATAGGCCTAGAATTAAATATNNNNNNNNNTGTCCCTGTATTAGCNNNNAGCACAAACCCCCGCT
CCACAGAAGCAGCCACAAAATACTTTCTAATTCAAGCAACCGCATCAATACTCCTACNNNNNNNTATCCTCAT
AAACAATNNNNNNNNNNNNNNNNNNNNNNNNNNNNNNNNNNNNNNNNNNNNNNNNNNNNNNNNNNNNNNNNNN
NNNNNNNNAACAAAATTAGGTATAGCCCCCCTGCACTTCTGACNCCCAGAAGTAACACAAGGTATTCCCCTCA
TCCCCGCTNNNNTTCTTCTCACATGACAAAAACTAGCCCCCCTATCTATTGTAATACAAATTTTTCNNNNNNN
NNNNNNNNNNNNNNNNNNNACAATCTCCATCCTATCAATTATAATTGGTAGCTGAGGCGGACTAAACCAAACA
CAACTACGTAAAATCATAGCATACTCCTCAATTACCCACATAGGATGAATAATAGCAGTCCTGCACTACAACC
CTAACATCACTATTCNNNNNNNNNNTATCTACTTATTTTTAACAATTTCCATTTTCATAACTTTTTATCTAAA
CTCAAATACAACAACTNNNNNNATATCCCACACCTGAAATAAATTCACATGAATAATACCCATTATNNNNNNN
NNNNNNNNNTCCTGAGGAGGCCTGCCTCCACTTACTGGCTTCTCCCCTAAATNNNCTGTTNNNCAAGAATTCA
CAAACAATAACAGTCTTGCTATACCCCTTGCTATNGCCNNNNTATCTCTAGTAAATTTATACTTTTACTTACA
CCTANCATACTCTATCTCTATAACAATATTTCCTACCTCCAACAACTCAAAAATCAATTGACAACTAAAATAT
ATAAAGCCATCCCCACTTCTTGCCCCACTCACAACCCTTTCCACCTTCCTTCTACCTATTACTCCANTCATAC
TAATAAACT????????????????????????????ATGTTACTTAAGCAATTTTACATTTACTGTAGCAGT
ANNNNNNTGCAATGGCCACTCTTATTCTCAAACAATAAAAACTGCCCAGCCTTTTCATGAAAGANTANTTGNN
ATTTCCATTGGCCTGTATATGCAATNNNNNNNNNGCCATAACTCATCAAAATTTAGCTATTTTCTCTTAAGAN
NTAGAAGAGCATTTTGCTTGAACATTGAACGGGANNNNNNNNNNTTCATGAAACTGAATNNNNNNNNNNNNNN
NNNAAATAAAACTCTCAAATTCTTTTCAACTAGATTGAAATGCTGTTTGAACCAGGTAANNNNNNNNNNNNNN
NNNNNNNNNNNNNNNNNNNNCAGCATAAAGAGTTTGTACAATATGTTCNNNNNNNNNNNNNNNNNNNNNNNNN
NNNNNNCTGAAAAGCCTGTTCCTCCTTTGAAATTGATTCATGATAACAGCCTCCATTTGGGTCAAACAGTANN

NNNNNNNNNNNNNNNATTTTAATNTAAATGATTTGTCACTTNNTGA???????????????????????????
????????????????????????GATTGTGAAGGAGTCGTGTGATGGAATGGGAGATGTGAGTGNGAAGCAT
GGGAGTGGGCCAGTAGTCCCAGAAAAGTCAGTCCGTTTTTCATTCACAATCATGAAAATTACTATANCCCACA
ACTCGCAGAACATGAACGTGTTTGAAGAAGCCAAACCTNNNNNNNNNNNNNNNNGCAAGCCACTGTGCCGTAT
GCTGGCAGATGAGTCTGACCACGAGACCCTGACTGCCATCCTGAGTCCTCTCATTGCCGAGAGGGAGGCCATG
AAGAGCAATGAATTAATGCTTGAGATGGGAGGCATTCCCCGGACTTTCAAGTTCATCTTCAGGGGCACCGGCT
ATGATGAAAAACTTGAGCGGGAAGTGGAAGGCCATGTGGCATCTGGCTCAGTCTGCNTCGGTACTCTNNNNNN
NNNNNNNNNNNNNGAAGCCTCTCTCAATCTTGTCTTCCACTCCATAACCAGAAGCCATACAGAGAACCTGGAA
CGTTATGAGGTCTGGCGTTCCAACCCTTNNNATGAGTCTGTGGAAGAACTGCGGGATCGGGTGAAAGGGGTCT
CAGCCAAACCTTTCANTGAGACAGTCCCTTCCATAGACGCCCTCCACTGNNNCATTGGCAATGCGGCTGAGTT
CTACAAGATCTTCCAGCTAGAGATAGGGGAGGTGCCTAAAGATCCCAATGCTTCCAAAGAGGAAAGGAAAAGG
TGGCAGGCCACACTGGACAAGCATCTCCGNNNNNNNNNNNNNNNNNAAACCAATCATGAGGATGAATGGCAACT
TTGCCAGGAAGCTCATGACCAAACAGACNNNNNNNNNNNNNNNNNNNNNNNNTTCCTTCTGAGGAGAGGCACGA
GGCTCTGAGGGAGCTGATGGATCTTTACCTGAAAATGAAACCAGTATGGCGGTCATCATGCCCTGCTAAAGAT
GGTGCCCGAGAATCCCTCTGCCAGTACAGTTTCAATTCACNNNNGTTTGCTGAGCTCCTNNNNNNNNNNNNNCA
AGTATAGATATGA??
??????????????CACTGCCGGCCCTTGGAGATGAGGATAAAGTGGACATTTCTCTGCTGCATCAGGGTGAG
AATCTTTTCGAACTGCACATCCACCAGGCCTTCCTGACATCTGCCGCCCTGGCTCAGGCTGGAGACACCCAAC
CTACCACTTTCTGCACCTATTCCTTCTATGACTTTGAAACCCACTGTACCCCACTGTCTGTAGGGCCAAAGCC
CCTCTATGACTTCACCTCTCAGTATGTGGTGGAGACAGACTCCNNNNNNNNNNNNNNNNNNTCAAGGGGCTTCA
GCCCGGCTTGATCTCCACCAGGCTGTGGCCAGTGAGCACAACACTCTTGCTGCAGGATGGATTTGCTTTGACA
GGGAGCTNNNNNNNNNNNGAGAAAGTCCATGGCTTGGCCACNNNNNNNGGTAGATGCTGTCAGCTTCCTGCAGC
TCCTA-
GCACCGCTGCAGAATTTCCCAAACTCTGNNNNNNCTTTTCTGGTTGTTTNNNNNTTTGTTCCTTTTTCTTTGT
TCTTGATCCTTGCCACNNNNNNNNNNNNTCTCCATGCTTTCATNNCTTGCTGCTGCCCAGGAGCTGGTGGAGAA
GAGTCTGGGGTTC??
???????????????????????????????????GGCAGCCTGGAGTCTGAAAAGCATCAATNNATCATCCA
TGGAGGGAAAACANNNNNNNNNNNNNNNNNNNNNNNNNNNNNNNNNNNNNNNNNNNNNNNNTTCCAAGAACAACAAA
AANNNNNNNNNNCGCTGCACGGAGAAAGACTTGGTAGGAGATGTTCCTGAAGCCAGATATGGTCATNCCANTA
ACGTGNNNNNNNNNNNNNNNNNNNNNNNTGGGTGTTCTTTTTGGAGGACGCTCATACATGCCAACTACCCACAG
AACCACAGAGAAATGGAATAGTGTAGCTGACTGCCTGCCCCATGTTTTCCTGGTGGATTTTGAATTTGGGTNN
NCTACATCATACATTCTTCCAGAACTTCAGGATGGNNNNNNNNNNNNNNNNNNCTATTGCCAAAAATGACACCA
TCTATATNTTAGGAGGACATTCACTTGCCAATAATNNNNNNNNNNNNNNNNNNNNNNNNNNNNNNGAGTTGATCT
TCCCCTGGGTAGCCGAGCNNN-
NNATTGCACAGTCTTGCCAGGAGGAATCTCTGNNNNNNNNNNNNNNNNNNNNNNNNNNNNNNNNATGATGAATTT
GTTATTGTTGGTGGATATCAGCTTGAAAATCAAAATAGANNNATCTGCAACATCATCTCTTTAGAGGAAAACA
AGATAGAAAT???
???
???
???
???
???
???
???
???
???
???
???
???
???
???
???
???

???
???
???
??A
AAAATCTTTATTGCAAAAAANNNNNNNTGACTGTCTTTGACTTGGACCNNNNNNNNNNNNNNNNNNNNNNCAA
AGCAGGACATGCAGCAGTTCTNNNAA-CCGCACTGTGGACGTGCGGAAAGNNNNNNNNNNNN--------
NNNNNNNNNNNNNNNNNNNNNNNNATTGAATTGTGAATGTTGTTCCAGGATGTGGAATGCAGCCATGATCACA
CTTGCCTACCGNNNNNNNNNNNNNNNNNTTTGTCAATAGCATGCAAAAAA-
TGCTTTGTCTGCCCTTTGCTTCTGCTTTTTCCAGGGAAGCTGCCAAAGAATGTCGACGTCGAAAGAAAGAATA
CGNNNNATGCCAGGAGAGCCGAGTGGCAGTGCTGGAAGTCCAGAACAAGAAGCTTATAGAGGAACTTGAAACC
TT???
???
???
???
???
???
???
???
???
???
???
???
???
???
???
???
???
???
???
???
???
???
???
???
???
???
???
???
???
???
???
???
???
???
???
???
???
???
???
?????ATGCAGAACTAGACCCCC-
GCCACAGCAGCCNNNNNNNNTGGACAGCAAAACCAATGCTTCACGACCCATCGGTGTCCATNNNNNNNNNNNN
NTGGAAAGAAACAAACNNNNNNGTCATATGATTTACTCATTATCGCCTTTTGACNNNNNNNNNNNNNNNNNNN
NNNNNNNNNNNNNNNNNATTAATCCACAGATCAGTAATGTATTCTCTCTCTCTCTCTCTTT----------
----

ACATTTTGATCTCTNNNNNNNNNNNNNNNNNNNNNNNNNNTACTGTAAAGAATTTAGCTGTTTCTAAACCTAG
TGCATGATATAGATTCTCTCCTGATTATTTATCANNNNNNNNNNNNNNNNNNNNNNNNNNNNNNNNNNNNNNN
NNNNNNNNNNNNNNNNNNNNNNNNNNNNNNNNNNNN-NNATCGATGGGGGA---
TGCTTCACGTGAACGTGGGAGTTTAGCTGCTTCTCTTGCCTAAGTATTCCTTTCCTTGATNNNNNNNNATTTT
AAAGTTAAACTATTTTTAAGTATTTCAGNNNNNNNNNAGAGATTTTTTTCC--
CATGANNNNNNNTTACTGTACAGATTGCTGCTTCTGCTATATTTGTGATATAGNNNNNNNNNNNNTACACGTT
TGTTTCTTCCTGC??
???
???
???
???
???
???
???
???
???
???
???
???
???
???
???
???
???
???
???
???
???
???
???
???
???
???
???
???
???
???
???
???
???
???
???
???
???
???
???
???
???
???
???
???
???
???
???

??
??
??
??
??
Acrecebus
??
??
??
??
??
??
??
??
??
??
??
??
??
??
??
??
??
??
??
??
??
??
??
??
??
??
??
??
??
??
??
??
??
??
??
??
??
??
??
??
??
??
??
??
??
??
??
??

??
??
??
??
??
??
??
??
??
??
??
??
??
??
??
??
??
??
??
??
??
??
??
??
??
??
??
??
??
??
??
??
??
??
??
??
??
??
??
??
??
??
??
??
??
??
??
??
??
??
??
??
??
??

??
??
??
??
??
??
??
??
??
??
??
??
??
??
??
??
??
??
??
??
??
??
??
??
??
??
??
??
??
??
??
??
??
??
??
??
??
??
??
??
??
??
??
??
??
??
??
??
??
??
??
??
??
??

??
??
??
??
??
??
??
??
??
??
??
??
??
??
??
??
??
??
??
??
??
??
??
??
??
??
??
??
??
??
??
??
??
??
??
??
??
??
??
??
??
??
??
??
??
??
??
??
??
??
??
??
??
??

??
??
??
??
??
??
??
??
??
??
??
??
??
??
??
??
??
??
??
??
??
??
??
??
??
??
??
??
??
??
??
??
??
??
??
??
??
??
??
??
??
??
??
??
??
??
??
??
??
??
??
??
??
??

???
???
???
???
???
???
???
???
???
???
???
???
???
???
??
Aegyptopithecus
???
???
???
???
???
???
???
???
???
???
???
???
???
???
???
???
???
???
???
???
???
???
???
???
???
???
???
???
???
???
???
???
???
???
???
???
???
???

??
??
??
??
??
??
??
??
??
??
??
??
??
??
??
??
??
??
??
??
??
??
??
??
??
??
??
??
??
??
??
??
??
??
??
??
??
??
??
??
??
??
??
??
??
??
??
??
??
??
??
??
??
??

??
??
??
??
??
??
??
??
??
??
??
??
??
??
??
??
??
??
??
??
??
??
??
??
??
??
??
??
??
??
??
??
??
??
??
??
??
??
??
??
??
??
??
??
??
??
??
??
??
??
??
??
??
??

??
??
??
??
??
??
??
??
??
??
??
??
??
??
??
??
??
??
??
??
??
??
??
??
??
??
??
??
??
??
??
??
??
??
??
??
??
??
??
??
??
??
??
??
??
??
??
??
??
??
??
??
??
??

??
??
??
??
??
??
??
??
??
??
??
??
??
??
??
??
??
??
??
??
??
??
??
??
??
??
??
??
??
??
??
??
??
??
??
??
??
??
??
??
??
??
??
??
??
??
??
??
??
??
??
??
??
??
??

???
???
???
???
???
???
???
???
???
???
???
???
???
???
???
???
???
???
???
???
???
???
???
???
??

Antillothrix

???
???
???
???
???
???
???
???
???
???
???
???
???
???
???
???
???
???
???
???
???
???
???
???
???
???
???

??
??
??
??
??
??
??
??
??
??
??
??
??
??
??
??
??
??
??
??
??
??
??
??
??
??
??
??
??
??
??
??
??
??
??
??
??
??
??
??
??
??
??
??
??
??
??
??
??
??
??
??
??
??

??
??
??
??
??
??
??
??
??
??
??
??
??
??
??
??
??
??
??
??
??
??
??
??
??
??
??
??
??
??
??
??
??
??
??
??
??
??
??
??
??
??
??
??
??
??
??
??
??
??
??
??
??
??

???
???
???
???
???
???
???
???
???
???
???
???
???
???
???
???
???
???
???
???
???
???
???
???
???
???
???
???
???
???
???
???
???
???
???
???
???
???
???
???
???
???
???
???
???
???
???
???
???
???
???
???
???
???

???
???
???
???
???
???
???
???
???
???
???
???
???
???
???
???
???
???
???
???
???
???
???
???
???
???
???
???
???
???
???
???
???
???
???
???
???
???
???
???
???
???
???
???
???
???
???
???
???
???
???
???
???
???

??
??
??
??
??
??
??
??
??
??
??
??
??
??
??
??
??
??
??
??
??
??
??
??
??
??
??
??
??
??
??
??
??
??
??
Aotus_dindensis
      ??
??
??
??
??
??
??
??
??
??
??
??
??
??
??
??
??
??

??
??
??
??
??
??
??
??
??
??
??
??
??
??
??
??
??
??
??
??
??
??
??
??
??
??
??
??
??
??
??
??
??
??
??
??
??
??
??
??
??
??
??
??
??
??
??
??
??
??
??
??
??
??
??
??

??
??
??
??
??
??
??
??
??
??
??
??
??
??
??
??
??
??
??
??
??
??
??
??
??
??
??
??
??
??
??
??
??
??
??
??
??
??
??
??
??
??
??
??
??
??
??
??
??
??
??
??
??
??

??
??
??
??
??
??
??
??
??
??
??
??
??
??
??
??
??
??
??
??
??
??
??
??
??
??
??
??
??
??
??
??
??
??
??
??
??
??
??
??
??
??
??
??
??
??
??
??
??
??
??
??
??
??

??
??
??
??
??
??
??
??
??
??
??
??
??
??
??
??
??
??
??
??
??
??
??
??
??
??
??
??
??
??
??
??
??
??
??
??
??
??
??
??
??
??
??
??
??
??
??
??
??
??
??
??
??
??

??
??
??
??
??
??
??
??
??
??
??
??
??
??
??
??
??
??
??
??
??
??
??
??
??
??
??
??
??
??
??
??
??
??
??
??
??
??
??
??
??
??
??
??
???
Apidium
??
??
??
??
??
??
??
??

??
??
??
??
??
??
??
??
??
??
??
??
??
??
??
??
??
??
??
??
??
??
??
??
??
??
??
??
??
??
??
??
??
??
??
??
??
??
??
??
??
??
??
??
??
??
??
??
??
??
??
??
??
??

??
??
??
??
??
??
??
??
??
??
??
??
??
??
??
??
??
??
??
??
??
??
??
??
??
??
??
??
??
??
??
??
??
??
??
??
??
??
??
??
??
??
??
??
??
??
??
??
??
??
??
??
??
??

??
??
??
??
??
??
??
??
??
??
??
??
??
??
??
??
??
??
??
??
??
??
??
??
??
??
??
??
??
??
??
??
??
??
??
??
??
??
??
??
??
??
??
??
??
??
??
??
??
??
??
??
??
??

??
??
??
??
??
??
??
??
??
??
??
??
??
??
??
??
??
??
??
??
??
??
??
??
??
??
??
??
??
??
??
??
??
??
??
??
??
??
??
??
??
??
??
??
??
??
??
??
??
??
??
??
??
??

??
??
??
??
??
??
??
??
??
??
??
??
??
??
??
??
??
??
??
??
??
??
??
??
??
??
??
??
??
??
??
??
??
??
??
??
??
??
??
??
??
??
??
??
??
??
??
??
??
??
??
??
??

??
???
Branisella
??
??
??
??
??
??
??
??
??
??
??
??
??
??
??
??
??
??
??
??
??
??
??
??
??
??
??
??
??
??
??
??
??
??
??
??
??
??
??
??
??
??
??
??
??
??
??
??
??
??
??
??

???
???
???
???
???
???
???
???
???
???
???
???
???
???
???
???
???
???
???
???
???
???
???
???
???
???
???
???
???
???
???
???
???
???
???
???
???
???
???
???
???
???
???
???
???
???
???
???
???
???
???
???
???
???

??
??
??
??
??
??
??
??
??
??
??
??
??
??
??
??
??
??
??
??
??
??
??
??
??
??
??
??
??
??
??
??
??
??
??
??
??
??
??
??
??
??
??
??
??
??
??
??
??
??
??
??
??
??

??
??
??
??
??
??
??
??
??
??
??
??
??
??
??
??
??
??
??
??
??
??
??
??
??
??
??
??
??
??
??
??
??
??
??
??
??
??
??
??
??
??
??
??
??
??
??
??
??
??
??
??
??
??

??
??
??
??
??
??
??
??
??
??
??
??
??
??
??
??
??
??
??
??
??
??
??
??
??
??
??
??
??
??
??
??
??
??
??
??
??
??
??
??
??
??
??
??
??
??
??
??
??
??
??
??
??
??

???
???
???
???
???
???
???
???
???
???
???
???
Canaanimico
???
???
???
???
???
???
???
???
???
???
???
???
???
???
???
???
???
???
???
???
???
???
???
???
???
???
???
???
???
???
???
???
???
???
???
???
???
???
???
???
???

??
??
??
??
??
??
??
??
??
??
??
??
??
??
??
??
??
??
??
??
??
??
??
??
??
??
??
??
??
??
??
??
??
??
??
??
??
??
??
??
??
??
??
??
??
??
??
??
??
??
??
??
??
??

??
??
??
??
??
??
??
??
??
??
??
??
??
??
??
??
??
??
??
??
??
??
??
??
??
??
??
??
??
??
??
??
??
??
??
??
??
??
??
??
??
??
??
??
??
??
??
??
??
??
??
??
??
??

??
??
??
??
??
??
??
??
??
??
??
??
??
??
??
??
??
??
??
??
??
??
??
??
??
??
??
??
??
??
??
??
??
??
??
??
??
??
??
??
??
??
??
??
??
??
??
??
??
??
??
??
??
??

??
??
??
??
??
??
??
??
??
??
??
??
??
??
??
??
??
??
??
??
??
??
??
??
??
??
??
??
??
??
??
??
??
??
??
??
??
??
??
??
??
??
??
??
??
??
??
??
??
??
??
??
??
??

??
??
??
??
??
??
??
??
??
??
??
??
??
??
??
??
??
??
??
??
??
???
Carlocebus
??
??
??
??
??
??
??
??
??
??
??
??
??
??
??
??
??
??
??
??
??
??
??
??
??
??
??
??
??
??
??

??
??
??
??
??
??
??
??
??
??
??
??
??
??
??
??
??
??
??
??
??
??
??
??
??
??
??
??
??
??
??
??
??
??
??
??
??
??
??
??
??
??
??
??
??
??
??
??
??
??
??
??
??
??

??
??
??
??
??
??
??
??
??
??
??
??
??
??
??
??
??
??
??
??
??
??
??
??
??
??
??
??
??
??
??
??
??
??
??
??
??
??
??
??
??
??
??
??
??
??
??
??
??
??
??
??
??
??

??
??
??
??
??
??
??
??
??
??
??
??
??
??
??
??
??
??
??
??
??
??
??
??
??
??
??
??
??
??
??
??
??
??
??
??
??
??
??
??
??
??
??
??
??
??
??
??
??
??
??
??
??
??

??
??
??
??
??
??
??
??
??
??
??
??
??
??
??
??
??
??
??
??
??
??
??
??
??
??
??
??
??
??
??
??
??
??
??
??
??
??
??
??
??
??
??
??
??
??
??
??
??
??
??
??
??

??
??
??
??
??
??
??
??
??
??
??
??
??
??
??
??
??
??
??
??
??
??
??
??
??
??
??
??
??
??
??
???

Catopithecus

??
??
??
??
??
??
??
??
??
??
??
??
??
??
??
??
??
??
??
??
??

??
??
??
??
??
??
??
??
??
??
??
??
??
??
??
??
??
??
??
??
??
??
??
??
??
??
??
??
??
??
??
??
??
??
??
??
??
??
??
??
??
??
??
??
??
??
??
??
??
??
??
??
??
??

??
??
??
??
??
??
??
??
??
??
??
??
??
??
??
??
??
??
??
??
??
??
??
??
??
??
??
??
??
??
??
??
??
??
??
??
??
??
??
??
??
??
??
??
??
??
??
??
??
??
??
??
??
??

??
??
??
??
??
??
??
??
??
??
??
??
??
??
??
??
??
??
??
??
??
??
??
??
??
??
??
??
??
??
??
??
??
??
??
??
??
??
??
??
??
??
??
??
??
??
??
??
??
??
??
??
??
??

??
??
??
??
??
??
??
??
??
??
??
??
??
??
??
??
??
??
??
??
??
??
??
??
??
??
??
??
??
??
??
??
??
??
??
??
??
??
??
??
??
??
??
??
??
??
??
??
??
??
??
??
??
??

??
??
??
??
??
??
??
??
??
??
??
??
??
??
??
??
??
??
??
??
??
??
??
??
??
??
??
??
??
??
??
??
??
??
??
??
??
??
??
??
??
???

Cebupithecia

??
??
??
??
??
??
??
??
??
??
??

??
??
??
??
??
??
??
??
??
??
??
??
??
??
??
??
??
??
??
??
??
??
??
??
??
??
??
??
??
??
??
??
??
??
??
??
??
??
??
??
??
??
??
??
??
??
??
??
??
??
??
??
??
??

??
??
??
??
??
??
??
??
??
??
??
??
??
??
??
??
??
??
??
??
??
??
??
??
??
??
??
??
??
??
??
??
??
??
??
??
??
??
??
??
??
??
??
??
??
??
??
??
??
??
??
??
??
??

??
??
??
??
??
??
??
??
??
??
??
??
??
??
??
??
??
??
??
??
??
??
??
??
??
??
??
??
??
??
??
??
??
??
??
??
??
??
??
??
??
??
??
??
??
??
??
??
??
??
??
??
??
??

???
???
???
???
???
???
???
???
???
???
???
???
???
???
???
???
???
???
???
???
???
???
???
???
???
???
???
???
???
???
???
???
???
???
???
???
???
???
???
???
???
???
???
???
???
???
???
???
???
???
???
???
???
???

???
???
???
???
???
???
???
???
???
???
???
???
???
???
???
???
???
???
???
???
???
???
???
???
???
???
???
???
???
???
???
???
???
???
???
???
???
???
???
???
???
???
???
???
???
???
???
???
???
???
???
??
Chilecebus
???

??
??
??
??
??
??
??
??
??
??
??
??
??
??
??
??
??
??
??
??
??
??
??
??
??
??
??
??
??
??
??
??
??
??
??
??
??
??
??
??
??
??
??
??
??
??
??
??
??
??
??
??
??
??

??
??
??
??
??
??
??
??
??
??
??
??
??
??
??
??
??
??
??
??
??
??
??
??
??
??
??
??
??
??
??
??
??
??
??
??
??
??
??
??
??
??
??
??
??
??
??
??
??
??
??
??
??
??

???
???
???
???
???
???
???
???
???
???
???
???
???
???
???
???
???
???
???
???
???
???
???
???
???
???
???
???
???
???
???
???
???
???
???
???
???
???
???
???
???
???
???
???
???
???
???
???
???
???
???
???
???
???

??
??
??
??
??
??
??
??
??
??
??
??
??
??
??
??
??
??
??
??
??
??
??
??
??
??
??
??
??
??
??
??
??
??
??
??
??
??
??
??
??
??
??
??
??
??
??
??
??
??
??
??
??
??

???
???
???
???
???
???
???
???
???
???
???
???
???
???
???
???
???
???
???
???
???
???
???
???
???
???
???
???
???
???
???
???
???
???
???
???
???
???
???
???
???
???
???
???
???
???
???
???
???
???
???
???
???
???
???
???
???
???
???

??
??
??
??
??
??
??
??

Dolichocebus

??
??
??
??
??
??
??
??
??
??
??
??
??
??
??
??
??
??
??
??
??
??
??
??
??
??
??
??
??
??
??
??
??
??
??
??
??
??
??
??
??
??
??
??
??
??
??

??
??
??
??
??
??
??
??
??
??
??
??
??
??
??
??
??
??
??
??
??
??
??
??
??
??
??
??
??
??
??
??
??
??
??
??
??
??
??
??
??
??
??
??
??
??
??
??
??
??
??
??
??
??

??
??
??
??
??
??
??
??
??
??
??
??
??
??
??
??
??
??
??
??
??
??
??
??
??
??
??
??
??
??
??
??
??
??
??
??
??
??
??
??
??
??
??
??
??
??
??
??
??
??
??
??
??
??

??
??
??
??
??
??
??
??
??
??
??
??
??
??
??
??
??
??
??
??
??
??
??
??
??
??
??
??
??
??
??
??
??
??
??
??
??
??
??
??
??
??
??
??
??
??
??
??
??
??
??
??
??
??

??
??
??
??
??
??
??
??
??
??
??
??
??
??
??
??
??
??
??
??
??
??
??
??
??
??
??
??
??
??
??
??
??
??
??
??
??
??
??
??
??
??
??
??
??
??
??
??
??
??
??
??
??
??

??
??
??
??
??
??
??
??
??
??
??
??
??
??
??
??
??
???
Homunculus
??
??
??
??
??
??
??
??
??
??
??
??
??
??
??
??
??
??
??
??
??
??
??
??
??
??
??
??
??
??
??
??
??
??
??

??
??
??
??
??
??
??
??
??
??
??
??
??
??
??
??
??
??
??
??
??
??
??
??
??
??
??
??
??
??
??
??
??
??
??
??
??
??
??
??
??
??
??
??
??
??
??
??
??
??
??
??
??
??

???
???
???
???
???
???
???
???
???
???
???
???
???
???
???
???
???
???
???
???
???
???
???
???
???
???
???
???
???
???
???
???
???
???
???
???
???
???
???
???
???
???
???
???
???
???
???
???
???
???
???
???
???
???

??
??
??
??
??
??
??
??
??
??
??
??
??
??
??
??
??
??
??
??
??
??
??
??
??
??
??
??
??
??
??
??
??
??
??
??
??
??
??
??
??
??
??
??
??
??
??
??
??
??
??
??
??
??

???
???
???
???
???
???
???
???
???
???
???
???
???
???
???
???
???
???
???
???
???
???
???
???
???
???
???
???
???
???
???
???
???
???
???
???
???
???
???
???
???
???
???
???
???
???
???
???
???
???
???
???
???
???

??
??
??
??
??
??
??
??
??
??
??
??
??
??
??
??
??
??
??
??
??
??
??
??
??
??
??
??
Killikaike
??
??
??
??
??
??
??
??
??
??
??
??
??
??
??
??
??
??
??
??
??
??
??
??
??

??
??
??
??
??
??
??
??
??
??
??
??
??
??
??
??
??
??
??
??
??
??
??
??
??
??
??
??
??
??
??
??
??
??
??
??
??
??
??
??
??
??
??
??
??
??
??
??
??
??
??
??
??
??

??
??
??
??
??
??
??
??
??
??
??
??
??
??
??
??
??
??
??
??
??
??
??
??
??
??
??
??
??
??
??
??
??
??
??
??
??
??
??
??
??
??
??
??
??
??
??
??
??
??
??
??
??
??

???
???
???
???
???
???
???
???
???
???
???
???
???
???
???
???
???
???
???
???
???
???
???
???
???
???
???
???
???
???
???
???
???
???
???
???
???
???
???
???
???
???
???
???
???
???
???
???
???
???
???
???
???
???

??
??
??
??
??
??
??
??
??
??
??
??
??
??
??
??
??
??
??
??
??
??
??
??
??
??
??
??
??
??
??
??
??
??
??
??
??
??
??
??
??
??
??
??
??
??
??
??
??
??
??
??
??
??
??
??

??
??
??
??
??
??
??
??
??
??
??
??
??
??
??
??
??
??
??
??
??
??
??
??
??
??
??
??
??
??
??
??
??
??
??
??
??
??
Lagonimico
??
??
??
??
??
??
??
??
??
??
??
??
??
??
??

??
??
??
??
??
??
??
??
??
??
??
??
??
??
??
??
??
??
??
??
??
??
??
??
??
??
??
??
??
??
??
??
??
??
??
??
??
??
??
??
??
??
??
??
??
??
??
??
??
??
??
??
??
??

??
??
??
??
??
??
??
??
??
??
??
??
??
??
??
??
??
??
??
??
??
??
??
??
??
??
??
??
??
??
??
??
??
??
??
??
??
??
??
??
??
??
??
??
??
??
??
??
??
??
??
??
??
??

???
???
???
???
???
???
???
???
???
???
???
???
???
???
???
???
???
???
???
???
???
???
???
???
???
???
???
???
???
???
???
???
???
???
???
???
???
???
???
???
???
???
???
???
???
???
???
???
???
???
???
???
???
???

??
??
??
??
??
??
??
??
??
??
??
??
??
??
??
??
??
??
??
??
??
??
??
??
??
??
??
??
??
??
??
??
??
??
??
??
??
??
??
??
??
??
??
??
??
??
??
??
??
??
??
??
??
??

???
???
???
???
???
???
???
???
???
???
???
???
???
???
???
???
???
???
???
???
???
???
???
???
???
???
???
???
???
???
???
???
???
???
???
???
???
???
???
???
???
???
???
???
???
???
???
??
Laventiana
???
???
???
???
???

??
??
??
??
??
??
??
??
??
??
??
??
??
??
??
??
??
??
??
??
??
??
??
??
??
??
??
??
??
??
??
??
??
??
??
??
??
??
??
??
??
??
??
??
??
??
??
??
??
??
??
??
??
??

??
??
??
??
??
??
??
??
??
??
??
??
??
??
??
??
??
??
??
??
??
??
??
??
??
??
??
??
??
??
??
??
??
??
??
??
??
??
??
??
??
??
??
??
??
??
??
??
??
??
??
??
??
??

??
??
??
??
??
??
??
??
??
??
??
??
??
??
??
??
??
??
??
??
??
??
??
??
??
??
??
??
??
??
??
??
??
??
??
??
??
??
??
??
??
??
??
??
??
??
??
??
??
??
??
??
??
??

???
???
???
???
???
???
???
???
???
???
???
???
???
???
???
???
???
???
???
???
???
???
???
???
???
???
???
???
???
???
???
???
???
???
???
???
???
???
???
???
???
???
???
???
???
???
???
???
???
???
???
???
???
???

??
??
??
??
??
??
??
??
??
??
??
??
??
??
??
??
??
??
??
??
??
??
??
??
??
??
??
??
??
??
??
??
??
??
??
??
??
??
??
??
??
??
??
??
??
??
??
??
??
??
??
??
??
??

??
??
??
???
Mazzonicebus
??
??
??
??
??
??
??
??
??
??
??
??
??
??
??
??
??
??
??
??
??
??
??
??
??
??
??
??
??
??
??
??
??
??
??
??
??
??
??
??
??
??
??
??
??
??
??
??
??

??
??
??
??
??
??
??
??
??
??
??
??
??
??
??
??
??
??
??
??
??
??
??
??
??
??
??
??
??
??
??
??
??
??
??
??
??
??
??
??
??
??
??
??
??
??
??
??
??
??
??
??
??
??
??
??
??
??
??
??

??
??
??
??
??
??
??
??
??
??
??
??
??
??
??
??
??
??
??
??
??
??
??
??
??
??
??
??
??
??
??
??
??
??
??
??
??
??
??
??
??
??
??
??
??
??
??
??
??
??
??
??
??
??

???
???
???
???
???
???
???
???
???
???
???
???
???
???
???
???
???
???
???
???
???
???
???
???
???
???
???
???
???
???
???
???
???
???
???
???
???
???
???
???
???
???
???
???
???
???
???
???
???
???
???
???
???
???

???
???
???
???
???
???
???
???
???
???
???
???
???
???
???
???
???
???
???
???
???
???
???
???
???
???
???
???
???
???
???
???
???
???
???
???
???
???
???
???
???
???
???
???
???
???
???
???
???
???
???
???
???
???

??
??
??
??
??
??
??
??
??
??
??
??
??
??
Miocallicebus
???
??
??
??
??
??
??
??
??
??
??
??
??
??
??
??
??
??
??
??
??
??
??
??
??
??
??
??
??
??
??
??
??
??
??
??
??
??
??

??
??
??
??
??
??
??
??
??
??
??
??
??
??
??
??
??
??
??
??
??
??
??
??
??
??
??
??
??
??
??
??
??
??
??
??
??
??
??
??
??
??
??
??
??
??
??
??
??
??
??
??
??
??

??
??
??
??
??
??
??
??
??
??
??
??
??
??
??
??
??
??
??
??
??
??
??
??
??
??
??
??
??
??
??
??
??
??
??
??
??
??
??
??
??
??
??
??
??
??
??
??
??
??
??
??
??
??

??
??
??
??
??
??
??
??
??
??
??
??
??
??
??
??
??
??
??
??
??
??
??
??
??
??
??
??
??
??
??
??
??
??
??
??
??
??
??
??
??
??
??
??
??
??
??
??
??
??
??
??
??
??

??
??
??
??
??
??
??
??
??
??
??
??
??
??
??
??
??
??
??
??
??
??
??
??
??
??
??
??
??
??
??
??
??
??
??
??
??
??
??
??
??
??
??
??
??
??
??
??
??
??
??
??
??
??

??
??
??
??
??
??
??
??
??
??
??
??
??
??
??
??
??
??
??
??
??
??
??
??
Mohanamico
??
??
??
??
??
??
??
??
??
??
??
??
??
??
??
??
??
??
??
??
??
??
??
??
??
??
??
??
??

??
??
??
??
??
??
??
??
??
??
??
??
??
??
??
??
??
??
??
??
??
??
??
??
??
??
??
??
??
??
??
??
??
??
??
??
??
??
??
??
??
??
??
??
??
??
??
??
??
??
??
??
??
??

??
??
??
??
??
??
??
??
??
??
??
??
??
??
??
??
??
??
??
??
??
??
??
??
??
??
??
??
??
??
??
??
??
??
??
??
??
??
??
??
??
??
??
??
??
??
??
??
??
??
??
??
??
??

??
??
??
??
??
??
??
??
??
??
??
??
??
??
??
??
??
??
??
??
??
??
??
??
??
??
??
??
??
??
??
??
??
??
??
??
??
??
??
??
??
??
??
??
??
??
??
??
??
??
??
??
??
??

??
??
??
??
??
??
??
??
??
??
??
??
??
??
??
??
??
??
??
??
??
??
??
??
??
??
??
??
??
??
??
??
??
??
??
??
??
??
??
??
??
??
??
??
??
??
??
??
??
??
??
??
??
??

??
??
??
??
??
??
??
??
??
??
??
??
??
??
??
??
??
??
??
??
??
??
??
??
??
??
??
??
??
??
??
??
??
???
Neosaimiri
??
??
??
??
??
??
??
??
??
??
??
??
??
??
??
??
??
??
??

??
??
??
??
??
??
??
??
??
??
??
??
??
??
??
??
??
??
??
??
??
??
??
??
??
??
??
??
??
??
??
??
??
??
??
??
??
??
??
??
??
??
??
??
??
??
??
??
??
??
??
??
??
??

??
??
??
??
??
??
??
??
??
??
??
??
??
??
??
??
??
??
??
??
??
??
??
??
??
??
??
??
??
??
??
??
??
??
??
??
??
??
??
??
??
??
??
??
??
??
??
??
??
??
??
??
??
??

??
??
??
??
??
??
??
??
??
??
??
??
??
??
??
??
??
??
??
??
??
??
??
??
??
??
??
??
??
??
??
??
??
??
??
??
??
??
??
??
??
??
??
??
??
??
??
??
??
??
??
??
??
??

??
??
??
??
??
??
??
??
??
??
??
??
??
??
??
??
??
??
??
??
??
??
??
??
??
??
??
??
??
??
??
??
??
??
??
??
??
??
??
??
??
??
??
??
??
??
??
??
??
??
??
??
??
??

???
???
???
???
???
???
???
???
???
???
???
???
???
???
???
???
???
???
???
???
???
???
???
???
???
???
???
???
???
???
???
???
???
???
???
???
???
???
???
???
???
???
???
??
Nuciruptor
???
???
???
???
???
???
???
???
???

???
???
???
???
???
???
???
???
???
???
???
???
???
???
???
???
???
???
???
???
???
???
???
???
???
???
???
???
???
???
???
???
???
???
???
???
???
???
???
???
???
???
???
???
???
???
???
???
???
???
???
???
???
???

??
??
??
??
??
??
??
??
??
??
??
??
??
??
??
??
??
??
??
??
??
??
??
??
??
??
??
??
??
??
??
??
??
??
??
??
??
??
??
??
??
??
??
??
??
??
??
??
??
??
??
??
??
??

??
??
??
??
??
??
??
??
??
??
??
??
??
??
??
??
??
??
??
??
??
??
??
??
??
??
??
??
??
??
??
??
??
??
??
??
??
??
??
??
??
??
??
??
??
??
??
??
??
??
??
??
??
??

???
???
???
???
???
???
???
???
???
???
???
???
???
???
???
???
???
???
???
???
???
???
???
???
???
???
???
???
???
???
???
???
???
???
???
???
???
???
???
???
???
???
???
???
???
???
???
???
???
???
???
???
???
???

??
??
??
??
??
??
??
??
??
??
??
??
??
??
??
??
??
??
??
??
??
??
??
??
??
??
??
??
??
??
??
??
??
??
??
??
??
??
??
??
??
??
??
??
??
??
??
??
??
??
??
??
??
??
??
??

Panamacebus
??
??
??
??
??
??
??
??
??
??
??
??
??
??
??
??
??
??
??
??
??
??
??
??
??
??
??
??
??
??
??
??
??
??
??
??
??
??
??
??
??
??
??
??
??
??
??
??
??
??
??
??
??

??
??
??
??
??
??
??
??
??
??
??
??
??
??
??
??
??
??
??
??
??
??
??
??
??
??
??
??
??
??
??
??
??
??
??
??
??
??
??
??
??
??
??
??
??
??
??
??
??
??
??
??
??
??
??
??
??

??
??
??
??
??
??
??
??
??
??
??
??
??
??
??
??
??
??
??
??
??
??
??
??
??
??
??
??
??
??
??
??
??
??
??
??
??
??
??
??
??
??
??
??
??
??
??
??
??
??
??
??
??
??

??
??
??
??
??
??
??
??
??
??
??
??
??
??
??
??
??
??
??
??
??
??
??
??
??
??
??
??
??
??
??
??
??
??
??
??
??
??
??
??
??
??
??
??
??
??
??
??
??
??
??
??
??
??

??
??
??
??
??
??
??
??
??
??
??
??
??
??
??
??
??
??
??
??
??
??
??
??
??
??
??
??
??
??
??
??
??
??
??
??
??
??
??
??
??
??
??
??
??
??
??
??
??
??
??
??
??
??
??

???
???
???
???
???
???
???
???
???
??
Paralouatta
???
???
???
???
???
???
???
???
???
???
???
???
???
???
???
???
???
???
???
???
???
???
???
???
???
???
???
???
???
???
???
???
???
???
???
???
???
???
???
???
???
???
???
???

???
???
???
???
???
???
???
???
???
???
???
???
???
???
???
???
???
???
???
???
???
???
???
???
???
???
???
???
???
???
???
???
???
???
???
???
???
???
???
???
???
???
???
???
???
???
???
???
???
???
???
???
???
???

???
???
???
???
???
???
???
???
???
???
???
???
???
???
???
???
???
???
???
???
???
???
???
???
???
???
???
???
???
???
???
???
???
???
???
???
???
???
???
???
???
???
???
???
???
???
???
???
???
???
???
???
???
???

???
???
???
???
???
???
???
???
???
???
???
???
???
???
???
???
???
???
???
???
???
???
???
???
???
???
???
???
???
???
???
???
???
???
???
???
???
???
???
???
???
???
???
???
???
???
???
???
???
???
???
???
???
???

??
??
??
??
??
??
??
??
??
??
??
??
??
??
??
??
??
??
??
??
??
??
??
??
??
??
??
??
??
??
??
??
??
??
??
??
??
??
??
??
??
??
??
??
??
??
??
??
??
??
??
??
??
??

???
???
???
???
???
???
???
???
???
???
???
???
???
???
???
???
???
???
???
???
Parvimico
???
???
???
???
???
???
???
???
???
???
???
???
???
???
???
???
???
???
???
???
???
???
???
???
???
???
???
???
???
???
???
???
???

??
??
??
??
??
??
??
??
??
??
??
??
??
??
??
??
??
??
??
??
??
??
??
??
??
??
??
??
??
??
??
??
??
??
??
??
??
??
??
??
??
??
??
??
??
??
??
??
??
??
??
??
??
??

???
???
???
???
???
???
???
???
???
???
???
???
???
???
???
???
???
???
???
???
???
???
???
???
???
???
???
???
???
???
???
???
???
???
???
???
???
???
???
???
???
???
???
???
???
???
???
???
???
???
???
???
???
???

??
??
??
??
??
??
??
??
??
??
??
??
??
??
??
??
??
??
??
??
??
??
??
??
??
??
??
??
??
??
??
??
??
??
??
??
??
??
??
??
??
??
??
??
??
??
??
??
??
??
??
??
??
??

??
??
??
??
??
??
??
??
??
??
??
??
??
??
??
??
??
??
??
??
??
??
??
??
??
??
??
??
??
??
??
??
??
??
??
??
??
??
??
??
??
??
??
??
??
??
??
??
??
??
??
??
??
??

???
???
???
???
???
???
???
???
???
???
???
???
???
???
???
???
???
???
???
???
???
???
???
???
???
???
???
???
???
???

Perupithecus

???
???
???
???
???
???
???
???
???
???
???
???
???
???
???
???
???
???
???
???
???
???

??
??
??
??
??
??
??
??
??
??
??
??
??
??
??
??
??
??
??
??
??
??
??
??
??
??
??
??
??
??
??
??
??
??
??
??
??
??
??
??
??
??
??
??
??
??
??
??
??
??
??
??
??
??

??
??
??
??
??
??
??
??
??
??
??
??
??
??
??
??
??
??
??
??
??
??
??
??
??
??
??
??
??
??
??
??
??
??
??
??
??
??
??
??
??
??
??
??
??
??
??
??
??
??
??
??
??
??

??
??
??
??
??
??
??
??
??
??
??
??
??
??
??
??
??
??
??
??
??
??
??
??
??
??
??
??
??
??
??
??
??
??
??
??
??
??
??
??
??
??
??
??
??
??
??
??
??
??
??
??
??
??

??
??
??
??
??
??
??
??
??
??
??
??
??
??
??
??
??
??
??
??
??
??
??
??
??
??
??
??
??
??
??
??
??
??
??
??
??
??
??
??
??
??
??
??
??
??
??
??
??
??
??
??
??
??

??
??
??
??
??
??
??
??
??
??
??
??
??
??
??
??
??
??
??
??
??
??
??
??
??
??
??
??
??
??
??
??
??
??
??
??
??
??
??
???

Proteopithecus

??
??
??
??
??
??
??
??
??
??
??
??
??

??
??
??
??
??
??
??
??
??
??
??
??
??
??
??
??
??
??
??
??
??
??
??
??
??
??
??
??
??
??
??
??
??
??
??
??
??
??
??
??
??
??
??
??
??
??
??
??
??
??
??
??
??
??

??
??
??
??
??
??
??
??
??
??
??
??
??
??
??
??
??
??
??
??
??
??
??
??
??
??
??
??
??
??
??
??
??
??
??
??
??
??
??
??
??
??
??
??
??
??
??
??
??
??
??
??
??
??

??
??
??
??
??
??
??
??
??
??
??
??
??
??
??
??
??
??
??
??
??
??
??
??
??
??
??
??
??
??
??
??
??
??
??
??
??
??
??
??
??
??
??
??
??
??
??
??
??
??
??
??
??
??

??
??
??
??
??
??
??
??
??
??
??
??
??
??
??
??
??
??
??
??
??
??
??
??
??
??
??
??
??
??
??
??
??
??
??
??
??
??
??
??
??
??
??
??
??
??
??
??
??
??
??
??
??
??

??
??
??
??
??
??
??
??
??
??
??
??
??
??
??
??
??
??
??
??
??
??
??
??
??
??
??
??
??
??
??
??
??
??
??
??
??
??
??
??
??
??
??
??
??
??
??
??
??
???

Proteropithecia

??
??
??

??
??
??
??
??
??
??
??
??
??
??
??
??
??
??
??
??
??
??
??
??
??
??
??
??
??
??
??
??
??
??
??
??
??
??
??
??
??
??
??
??
??
??
??
??
??
??
??
??
??
??
??
??
??

??
??
??
??
??
??
??
??
??
??
??
??
??
??
??
??
??
??
??
??
??
??
??
??
??
??
??
??
??
??
??
??
??
??
??
??
??
??
??
??
??
??
??
??
??
??
??
??
??
??
??
??
??
??
??
??

??
??
??
??
??
??
??
??
??
??
??
??
??
??
??
??
??
??
??
??
??
??
??
??
??
??
??
??
??
??
??
??
??
??
??
??
??
??
??
??
??
??
??
??
??
??
??
??
??
??
??
??
??
??

??
??
??
??
??
??
??
??
??
??
??
??
??
??
??
??
??
??
??
??
??
??
??
??
??
??
??
??
??
??
??
??
??
??
??
??
??
??
??
??
??
??
??
??
??
??
??
??
??
??
??
??
??
??

??
??
??
??
??
??
??
??
??
??
??
??
??
??
??
??
??
??
??
??
??
??
??
??
??
??
??
??
??
??
??
??
??
??
??
??
??
??
??
??
??
??
??
??
??
??
??
??
??
??
??
??
??
??

???
???
???
???
???
??
Qatrania
???
???
???
???
???
???
???
???
???
???
???
???
???
???
???
???
???
???
???
???
???
???
???
???
???
???
???
???
???
???
???
???
???
???
???
???
???
???
???
???
???
???
???
???
???
???
???
???

??
??
??
??
??
??
??
??
??
??
??
??
??
??
??
??
??
??
??
??
??
??
??
??
??
??
??
??
??
??
??
??
??
??
??
??
??
??
??
??
??
??
??
??
??
??
??
??
??
??
??
??
??
??

??
??
??
??
??
??
??
??
??
??
??
??
??
??
??
??
??
??
??
??
??
??
??
??
??
??
??
??
??
??
??
??
??
??
??
??
??
??
??
??
??
??
??
??
??
??
??
??
??
??
??
??
??
??

??
??
??
??
??
??
??
??
??
??
??
??
??
??
??
??
??
??
??
??
??
??
??
??
??
??
??
??
??
??
??
??
??
??
??
??
??
??
??
??
??
??
??
??
??
??
??
??
??
??
??
??
??
??

??
??
??
??
??
??
??
??
??
??
??
??
??
??
??
??
??
??
??
??
??
??
??
??
??
??
??
??
??
??
??
??
??
??
??
??
??
??
??
??
??
??
??
??
??
??
??
??
??
??
??
??
??
??

???
???
???
???
???
???
???
???
???
???
???
???
???
???
???
???

Simonsius

???
???
???
???
???
???
???
???
???
???
???
???
???
???
???
???
???
???
???
???
???
???
???
???
???
???
???
???
???
???
???
???
???
???
???
???
???

??
??
??
??
??
??
??
??
??
??
??
??
??
??
??
??
??
??
??
??
??
??
??
??
??
??
??
??
??
??
??
??
??
??
??
??
??
??
??
??
??
??
??
??
??
??
??
??
??
??
??
??
??
??

???
???
???
???
???
???
???
???
???
???
???
???
???
???
???
???
???
???
???
???
???
???
???
???
???
???
???
???
???
???
???
???
???
???
???
???
???
???
???
???
???
???
???
???
???
???
???
???
???
???
???
???
???
???

??
??
??
??
??
??
??
??
??
??
??
??
??
??
??
??
??
??
??
??
??
??
??
??
??
??
??
??
??
??
??
??
??
??
??
??
??
??
??
??
??
??
??
??
??
??
??
??
??
??
??
??
??
??

??
??
??
??
??
??
??
??
??
??
??
??
??
??
??
??
??
??
??
??
??
??
??
??
??
??
??
??
??
??
??
??
??
??
??
??
??
??
??
??
??
??
??
??
??
??
??
??
??
??
??
??
??
??

??
??
??
??
??
??
??
??
??
??
??
??
??
??
??
??
??
??
??
??
??
??
??
??
??
???
Soriacebus
??
??
??
??
??
??
??
??
??
??
??
??
??
??
??
??
??
??
??
??
??
??
??
??
??
??
??

??
??
??
??
??
??
??
??
??
??
??
??
??
??
??
??
??
??
??
??
??
??
??
??
??
??
??
??
??
??
??
??
??
??
??
??
??
??
??
??
??
??
??
??
??
??
??
??
??
??
??
??
??
??
??

??
??
??
??
??
??
??
??
??
??
??
??
??
??
??
??
??
??
??
??
??
??
??
??
??
??
??
??
??
??
??
??
??
??
??
??
??
??
??
??
??
??
??
??
??
??
??
??
??
??
??
??
??
??

??
??
??
??
??
??
??
??
??
??
??
??
??
??
??
??
??
??
??
??
??
??
??
??
??
??
??
??
??
??
??
??
??
??
??
??
??
??
??
??
??
??
??
??
??
??
??
??
??
??
??
??
??
??

??
??
??
??
??
??
??
??
??
??
??
??
??
??
??
??
??
??
??
??
??
??
??
??
??
??
??
??
??
??
??
??
??
??
??
??
??
??
??
??
??
??
??
??
??
??
??
??
??
??
??
??
??
??

???
???
???
???
???
???
???
???
???
???
???
???
???
???
???
???
???
???
???
???
???
???
???
???
???
???
???
???
???
???
???
???
???
???
???
???
Stirtonia
???
???
???
???
???
???
???
???
???
???
???
???
???
???
???
???
???

??
??
??
??
??
??
??
??
??
??
??
??
??
??
??
??
??
??
??
??
??
??
??
??
??
??
??
??
??
??
??
??
??
??
??
??
??
??
??
??
??
??
??
??
??
??
??
??
??
??
??
??
??

??
??
??
??
??
??
??
??
??
??
??
??
??
??
??
??
??
??
??
??
??
??
??
??
??
??
??
??
??
??
??
??
??
??
??
??
??
??
??
??
??
??
??
??
??
??
??
??
??
??
??
??
??
??
??
??
??

??
??
??
??
??
??
??
??
??
??
??
??
??
??
??
??
??
??
??
??
??
??
??
??
??
??
??
??
??
??
??
??
??
??
??
??
??
??
??
??
??
??
??
??
??
??
??
??
??
??
??
??
??
??

??
??
??
??
??
??
??
??
??
??
??
??
??
??
??
??
??
??
??
??
??
??
??
??
??
??
??
??
??
??
??
??
??
??
??
??
??
??
??
??
??
??
??
??
??
??
??
??
??
??
??
??
??
??

??
??
??
??
??
??
??
??
??
??
??
??
??
??
??
??
??
??
??
??
??
??
??
??
??
??
??
??
??
??
??
??
??
??
??
??
??
??
??
??
??
??
??
??
??
???

Talahpithecus

??
??
??
??
??
??
??

??
??
??
??
??
??
??
??
??
??
??
??
??
??
??
??
??
??
??
??
??
??
??
??
??
??
??
??
??
??
??
??
??
??
??
??
??
??
??
??
??
??
??
??
??
??
??
??
??
??
??
??
??
??
??
??
??
??
??
??

??
??
??
??
??
??
??
??
??
??
??
??
??
??
??
??
??
??
??
??
??
??
??
??
??
??
??
??
??
??
??
??
??
??
??
??
??
??
??
??
??
??
??
??
??
??
??
??
??
??
??
??
??
??

??
??
??
??
??
??
??
??
??
??
??
??
??
??
??
??
??
??
??
??
??
??
??
??
??
??
??
??
??
??
??
??
??
??
??
??
??
??
??
??
??
??
??
??
??
??
??
??
??
??
??
??
??
??

??
??
??
??
??
??
??
??
??
??
??
??
??
??
??
??
??
??
??
??
??
??
??
??
??
??
??
??
??
??
??
??
??
??
??
??
??
??
??
??
??
??
??
??
??
??
??
??
??
??
??
??
??
??

??
??
??
??
??
??
??
??
??
??
??
??
??
??
??
??
??
??
??
??
??
??
??
??
??
??
??
??
??
??
??
??
??
??
??
??
??
??
??
??
??
??
??
??
??
??
??
??
??
??
??
??
??
??

???
???
Tremacebus
???
???
???
???
???
???
???
???
???
???
???
???
???
???
???
???
???
???
???
???
???
???
???
???
???
???
???
???
???
???
???
???
???
???
???
???
???
???
???
???
???
???
???
???
???
???
???
???
???
???
???
???

??
??
??
??
??
??
??
??
??
??
??
??
??
??
??
??
??
??
??
??
??
??
??
??
??
??
??
??
??
??
??
??
??
??
??
??
??
??
??
??
??
??
??
??
??
??
??
??
??
??
??
??
??
??

??
??
??
??
??
??
??
??
??
??
??
??
??
??
??
??
??
??
??
??
??
??
??
??
??
??
??
??
??
??
??
??
??
??
??
??
??
??
??
??
??
??
??
??
??
??
??
??
??
??
??
??
??
??

??
??
??
??
??
??
??
??
??
??
??
??
??
??
??
??
??
??
??
??
??
??
??
??
??
??
??
??
??
??
??
??
??
??
??
??
??
??
??
??
??
??
??
??
??
??
??
??
??
??
??
??
??
??

??
??
??
??
??
??
??
??
??
??
??
??
??
??
??
??
??
??
??
??
??
??
??
??
??
??
??
??
??
??
??
??
??
??
??
??
??
??
??
??
??
??
??
??
??
??
??
??
??
??
??
??
??
??

??
??
??
??
??
??
??
??
??
??
??
???
Ucayalipithecus
??
??
??
??
??
??
??
??
??
??
??
??
??
??
??
??
??
??
??
??
??
??
??
??
??
??
??
??
??
??
??
??
??
??
??
??
??
??
??
??

??
??
??
??
??
??
??
??
??
??
??
??
??
??
??
??
??
??
??
??
??
??
??
??
??
??
??
??
??
??
??
??
??
??
??
??
??
??
??
??
??
??
??
??
??
??
??
??
??
??
??
??
??
??

??
??
??
??
??
??
??
??
??
??
??
??
??
??
??
??
??
??
??
??
??
??
??
??
??
??
??
??
??
??
??
??
??
??
??
??
??
??
??
??
??
??
??
??
??
??
??
??
??
??
??
??
??
??

??
??
??
??
??
??
??
??
??
??
??
??
??
??
??
??
??
??
??
??
??
??
??
??
??
??
??
??
??
??
??
??
??
??
??
??
??
??
??
??
??
??
??
??
??
??
??
??
??
??
??
??
??
??
??

??
??
??
??
??
??
??
??
??
??
??
??
??
??
??
??
??
??
??
??
??
??
??
??
??
??
??
??
??
??
??
??
??
??
??
??
??
??
??
??
??
??
??
??
??
??
??
??
??
??
??
??
??
??

???
???
???
???
???
???
???
???
???
???
???
???
???
???
???
???
???
???
???
???
???
??
Insulacebus
???
???
???
???
???
???
???
???
???
???
???
???
???
???
???
???
???
???
???
???
???
???
???
???
???
???
???
???
???
???
???

??
??
??
??
??
??
??
??
??
??
??
??
??
??
??
??
??
??
??
??
??
??
??
??
??
??
??
??
??
??
??
??
??
??
??
??
??
??
??
??
??
??
??
??
??
??
??
??
??
??
??
??
??
??

???
???
???
???
???
???
???
???
???
???
???
???
???
???
???
???
???
???
???
???
???
???
???
???
???
???
???
???
???
???
???
???
???
???
???
???
???
???
???
???
???
???
???
???
???
???
???
???
???
???
???
???
???
???
???
???

??
??
??
??
??
??
??
??
??
??
??
??
??
??
??
??
??
??
??
??
??
??
??
??
??
??
??
??
??
??
??
??
??
??
??
??
??
??
??
??
??
??
??
??
??
??
??
??
??
??
??
??
??
??

??
??
??
??
??
??
??
??
??
??
??
??
??
??
??
??
??
??
??
??
??
??
??
??
??
??
??
??
??
??
??
??
??
??
??
??
??
??
??
??
??
??
??
??
??
??
??
??
??
??
??
??
??
??

??
??
??
??
??
??
??
??
??
??
??
??
??
??
??
??
??
??
??
??
??
??
??
??
??
??
??
??
??
??
??
??

;
